# Supplementary material for: Copper-catalyzed carbo-difluoromethylation of alkenes via radical relay
Source: Nat Commun. 2021 Jun 1;12:3272. doi: 10.1038/s41467-021-23590-2 (PMC8169770; doi:10.1038/s41467-021-23590-2)
Supplement: Supplementary file 1 — Supplementary information [file 41467_2021_23590_MOESM1_ESM.pdf]

Supplementary Information

Copper-Catalyzed Carbo-Difluoromethylation of Alkenes via Radical Relay

Aijie Cai,[a] Wenhao Yan,[a] Xiaojun Zeng,[a] Samson B. Zacate,[b] Tzu-Hsuan Chao,[c] Jeanette A. Krause,[a] Mu-Jeng Cheng,[c] Wei Liu*[a]

[a]Department of Chemistry, University of Cincinnati, Cincinnati, OH, 45221, U.S.A.

[b]Department of Chemistry and Biochemistry, Miami University, Oxford, OH, 45056, U.S.A.

[c]Department of Chemistry, National Cheng Kung University, Tainan, 701, Taiwan

E-mail: liu2w2@uc.edu

Contents:

**Page S2:**	**I.**	General information
**Page S2:**	**II.**	Supplementary method 1. General procedure for Cu-catalyzed radical-relayed difluoromethylation
**Page S6:**	**III.**	Supplementary method 2. General procedure for the synthesis of redox-active esters (RAEs)
**Page S6:**	**IV.**	Supplementary method 3. General procedure for the synthesis of styrenes
**Page S7:**	**V.**	Supplementary method 4. General procedure for synthetic transformation
**Page S8:**	**VI.**	Procedure for additional experiments
**Page S20:**	**VII.**	DFT Calculations of Cu/bpy system catalyzed decarboxylative difluoromethylation of secondary cyclic RAEs
**Page S21:**	**VIII.**	Reaction optimization
**Page S25:**	**IX.**	Supplementary method 5. Synthesis and characterizations of new compounds
**Page S72:**	**X.**	Crystallographic data for radical-relayed difluoromethylation product **27**
**Page S79:**	**XI.**	Crystallographic data for radical-relayed difluoromethylation product **43**
**Page S85:**	**XII.**	Computational details
**Page S100:**	**XIII.**	NMR spectra of radical-relayed difluoromethylation products
**Page S322:**	**XIV.**	Supplementary References

I. General information

Unless otherwise noted, all difluoromethylation reactions were run under an inert atmosphere and all glassware was flame dried before use. Copper catalysts were purchased from Strem Chemicals, INC. All other purchased chemicals were of the highest purity available from Millipore-Sigma, Oakwood Chemical, AK Scientific, Fisher Scientific, or TCI America and were used without further purification. DMSO was purchased from Alfa Aaser (99.9%) and dried with 4Å molecular sieves. All the TCNHPI redox-active esters were synthesized according to the published procedures.[1-4] The alkenes were prepared according to previously reported protocols.[5-6] The aryl diazonium salts were prepared according to a reported procedure.[7] GC/MS analysis was performed on a Thermo-Fischer Scientific ISQ QD single quadrupole mass spectrometer. Flash column chromatography was performed using F60 silica gel (40-63 μm, 230-400 mesh, 60 Å) purchased from Silicycle. Analytical thin-layer chromatography (TLC) was carried out on 250 μm 60-F_{254} silica gel plates purchased from Silicycle, and visualization was affected by observation of fluorescence-quenching with ultraviolet light and staining with $KMnO_4$ or I_2. ^{1}H NMR, ^{13}C NMR and ^{19}F NMR spectra were recored at 297K on a Bruker AVANCE AV 400 (400 MHz, 101MHz and 376 MHz) spectrometer, and ^{19}F NMR was not referenced. Data is reported in ppm using $CDCl_3$ and DMSO-d_6 as solvent unless otherwise specified. Data is reported as: Chemical shifts (δ), multiplicity (s = singlet, d = doublet, t = triplet, q = quartet, m = multiplet, br = broad), coupling constants (Hz); integrated intensity. High-resolution Mass spectra were obtained from University of Cincinnati Mass Spectral Facility or the Mass Spectrometry Facility in the Department of Chemistry at Princeton University.

II. Supplementary Method 1. General procedure for Cu-catalyzed radical-relayed difluoromethylation

Alkyl TCNHPI redox esters (RAEs) as electrophiles (general procedure A):

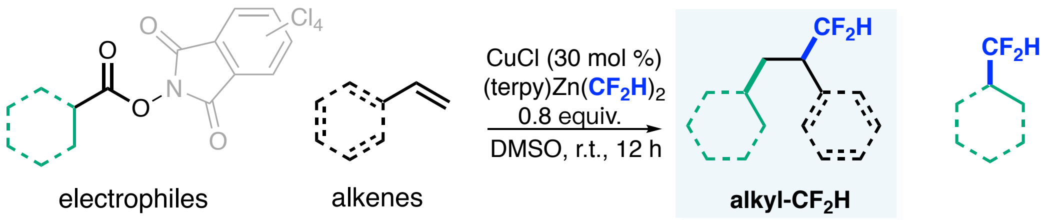

In a nitrogen-filled glovebox, to a 4 mL vial equipped with a stir bar was added terpyridine (0.8 equiv., 47 mg), $(DMPU)_2Zn(CF_2H)_2$ (0.8 equiv., 87 mg), and 800 μL DMSO. The resulting mixture was stirred at room temperature for 1 min to generate (terpy)$Zn(CF_2H)_2$ in-situ. A different 4 mL vial equipped with a stir bar was sequentially charged with CuCl (30 mol %, 7.5 mg), RAEs (0.25 mmol, 1.0 equiv.), the DMSO solution of the in-situ formed (terpy)$Zn(CF_2H)_2$, and alkene (0.75 mmol, 3.0 equiv.) in DMSO (200 μL). The resultant mixture was stirred at room temperature for 12 h. After the reaction was completed, the mixture

was diluted with EtOAc (50 mL), filtered through a short plug of Celite, and washed with H_2O (50 mL) and brine. The organic layer was combined, dried over Na_2SO_4, filtered, and then concentrated under reduced pressure. The crude difluoromethylation product was purified by flash column chromatography. (*note: generally 5-15% direct decarboxylative difluoromethylation products were observed in the crude reaction mixtures. Excess unreacted alkenes could be simply recovered (0.45-0.50 mmol) by column chromatography.*).

Alkyl pyridinium salts as electrophiles (general procedure B):

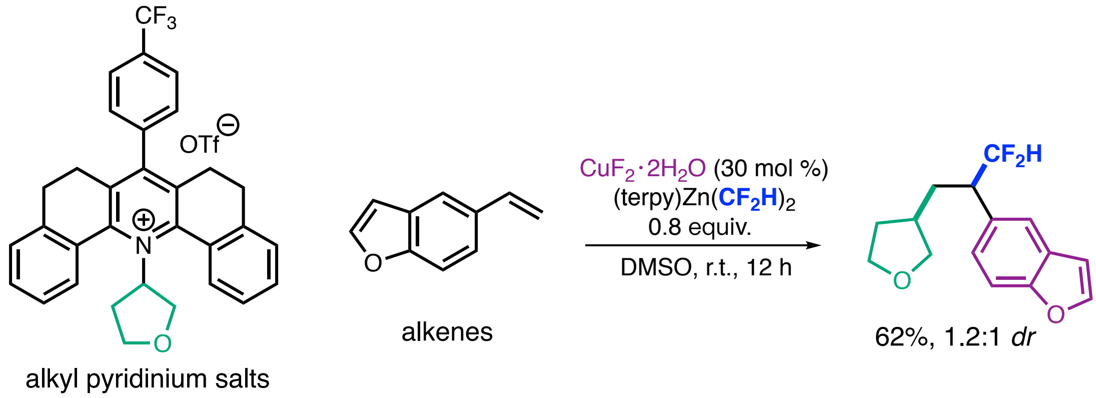

In a nitrogen-filled glovebox, to a 4 mL vial equipped with a stir bar was added terpyridine (0.8 equiv., 47 mg), $(DMPU)_2Zn(CF_2H)_2$ (0.8 equiv., 87 mg), and 800 μL DMSO. The resulting mixture was stirred at room temperature for 1 min to generate $(terpy)Zn(CF_2H)_2$ in-situ. A different 4 mL vial equipped with a stir bar was sequentially charged with CuF_2•$2H_2O$ (30 mol %, 10.0 mg), tetrahydrofuran pyridinium salt (0.25 mmol, 162 mg, 1.0 equiv.), and the DMSO solution of the in-situ formed $(terpy)Zn(CF_2H)_2$, followed by 5-vinylbenzofuran (0.75 mol, 108 mg, 3.0 equiv.) in DMSO (200 μL). The resultant mixture was stirred at room temperature for 12 h. After the reaction was completed, the mixture was diluted with EtOAc (50 mL), filtered through a short plug of Celite, and washed with H_2O (50 mL) and brine. The organic layer was combined, dried over Na_2SO_4, filtered, and then concentrated under reduced pressure. The crude mixture was purified by flash column chromatography (eluted with 5% ethyl acetate in hexane) to afford the corresponding difluoromethylated product. (*note: The use of CuCl as catalyst provided the difluoromethylated product in lower yield (54% was determined by ^{19}F NMR) compared to CuF_2•$2H_2O$ under the similar standard conditions*).

Alkyl bromide as electrophiles (general procedure C):

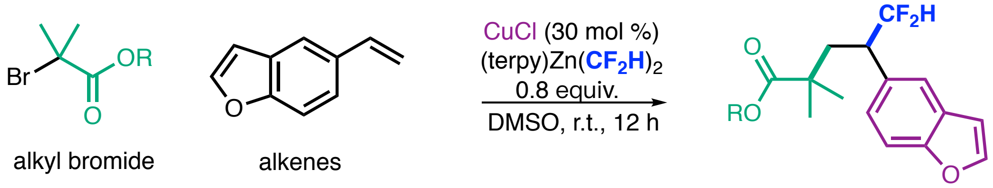

52, R = Me; 54%
53, R = ^{t}Bu; 54%

In a nitrogen-filled glovebox, to a 4 mL vial equipped with a stir bar was added terpyridine (0.8 equiv., 47 mg), $(DMPU)_2Zn(CF_2H)_2$ (0.8 equiv., 87 mg), and 800 μL DMSO. The resulting mixture was stirred at room temperature for 1 min to generate $(terpy)Zn(CF_2H)_2$ in-situ. A different 4 mL vial equipped with a stir bar was sequentially charged with CuCl (30 mol %, 7.5 mg), the DMSO solution of the in-situ formed $(terpy)Zn(CF_2H)_2$, 5-vinylbenzofuran (0.75 mol, 108 mg, 3.0 equiv.), and alkyl bromide (0.25 mmol, 1.0 equiv.) in DMSO (200 μL). The resultant mixture was stirred at room temperature for 12 h. After the reaction was completed, the mixture was diluted with EtOAc (50 mL), filtered through a short plug of Celite, and washed with H_2O (50 mL) and brine. The organic layer was combined, dried over Na_2SO_4, filtered, and then concentrated under reduced pressure. The crude difluoromethylation product was purified by flash column chromatography.

Aryl diazonium salts as electrophiles (general procedure D):

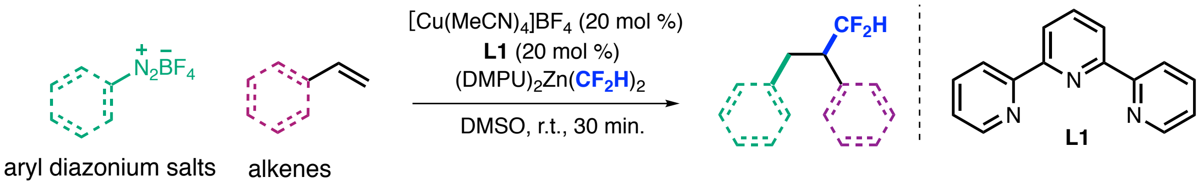

In a nitrogen-filled glovebox, a 4 mL vial equipped with a stir bar was charged with $[Cu(MeCN)_4]BF_4$ (20 mol %, 15.5 mg), terpyridine (**L1**) (20 mol %, 11.8 mg), alkenes (0.75 mmol, 3.0 equiv.) and 200 μL DMSO. A solution of diazonium salts (0.25 mmol, 1.0 equiv.) in 400 μL DMSO, and a solution of $(DMPU)_2Zn(CF_2H)_2$ (0.2 mmol, 87 mg, 0.8 equiv.) in 400 μL DMSO were slowly added to the vial with syringes at the same time over the course of 10 minutes. The resultant mixture was stirred at room temperature for 30 minutes. After the reaction was completed, the mixture was diluted with EtOAc (50 mL), filtered through a short plug of Celite, and washed with H_2O (50 mL) and brine. The organic layer was combined, dried over Na_2SO_4, filtered, and then concentrated under reduced pressure. The crude difluoromethylation product was purified by flash column chromatography.

For gram-scale synthesis:

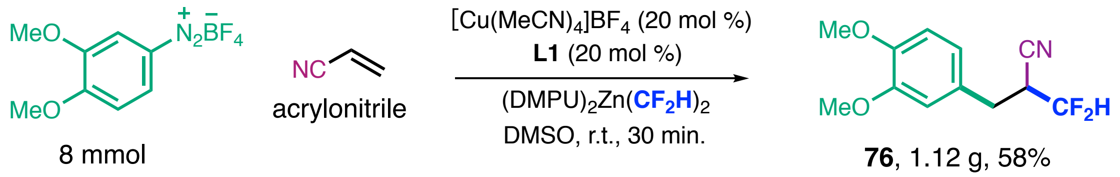

In a nitrogen-filled glovebox, a 100 mL round bottom flask equipped with a stir bar was charged with $[Cu(MeCN)_4]BF_4$ (20 mol %, 496 mg), terpyridine (**L1**) (20 mol %, 376 mg), acrylonitrile (24 mmol, 1.27g, 3.0 equiv.) and 6.4 mL DMSO. A solution of diazonium salts (8.0 mmol, 2.01g, 1.0 equiv.) in 12.8 mL DMSO and a solution of $(DMPU)_2Zn(CF_2H)_2$ (6.4 mmol, 2.78 g, 0.8 equiv.) in 12.8 mL DMSO were slowly added to the vial with syringes at the same time over the course of 1 h. The resultant mixture was stirred at room temperature for 30 min. After the reaction was completed, the mixture was diluted with EtOAc (100 mL), filtered through a short plug of Celite, and washed with H_2O (100 mL) and brine. The organic layer was combined, dried over Na_2SO_4, filtered, and then concentrated under reduced pressure. The crude difluoromethylation product was purified by flash column chromatography to give the desired product **76** (1.12g, 58% yield).

Reaction set up outside of glovebox:

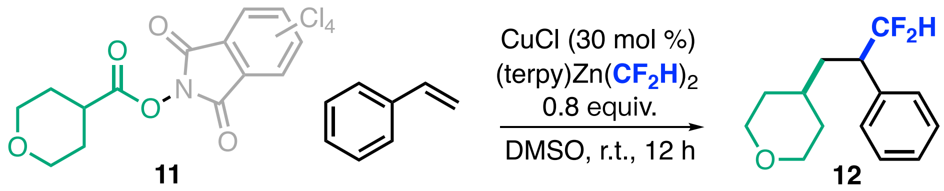

In the air, $(DMPU)_2Zn(CF_2H)_2$ was quickly dissolved in DMSO to make a stock solution of $(DMPU)_2Zn(CF_2H)_2$. The stock solution was stored in a -20 °C freezer and no significant decomposition of the solution was observed by ^{19}F NMR over the period of a month.

In the air, 4 mL oven-dried vial (A) equipped with a stir bar was charged with CuCl (30 mol %, 7.5 mg) and the RAE (**11**, 0.25 mmol, 1.0 equiv.). The vial was closed with a PTEE septum cap and wrapped with electrical tape. The vial was evacuated and back-filled with Argon on a Schlenk line (three cycles), then added DMSO (200 μL). A different vial (B) charged with terpyridine (0.8 equiv., 47 mg) was also evacuated and backfilled with Argon. The stock solution of $(DMPU)_2Zn(CF_2H)_2$ (800 μL) was then added to vial B and mixed for 1 min to generate $(terpy)Zn(CF_2H)_2$ in-situ. This solution was then added to vial A along with styrene (0.75 mmol, 3.0 equiv.) was then injected to vial A using a syringe. The resultant mixture was stirred at room temperature for 12 h. The yield of product **12** was determined by ^{19}F NMR (74 %).

III. Supplementary Method 2. General procedure for the synthesis of redox-active esters (RAEs)

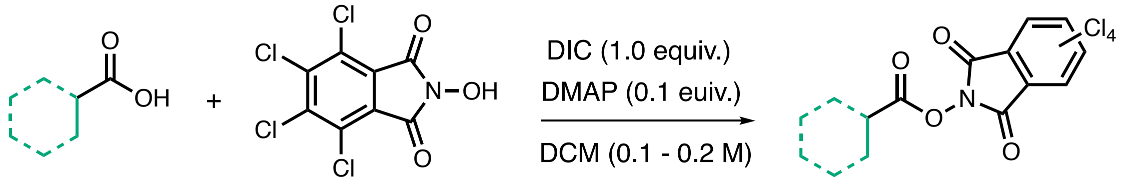

Redox-active esters were prepared according to the previously reported procedure[1-4]: A round-bottom flask or culture tube was charged with (if solid) carboxylic acid (1.0 equiv.), tetrachloro-*N*- hydroxyphthalimide (1.0 equiv.) and DMAP (0.1 equiv). Dichloromethane was added (0.1 – 0.2 M), and the mixture was stirred vigorously. Carboxylic acid (1.0 equiv) was added via syringe (if liquid). DIC (1.1 equiv) was then added dropwise via syringe, and the mixture was allowed to stir until the acid was consumed (determined by TLC). Typical reaction times were between 0.5 to 12 h. The mixture was filtered (over Celite, SiO_2, or through a fritted funnel) and rinsed with additional CH_2Cl_2/Et_2O. The organic solvent was removed under reduced pressure, and purification by column chromatography and recrystallization with CH_2Cl_2 and MeOH, if necessary, afforded the corresponding redox-active ester.

IV. Supplementary Method 3. General procedure for the synthesis of styrenes

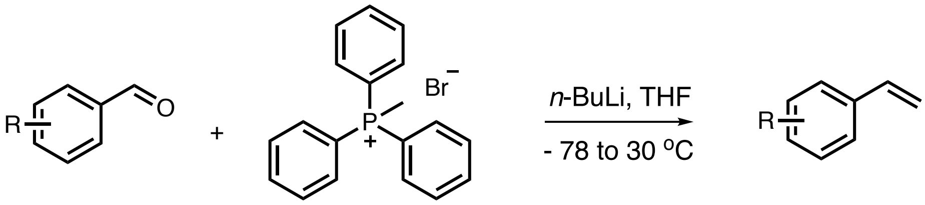

Styrenes were prepared according to the previously reported procedure[5-6]: Methyltriphenylphosphonium bromide (2.64 g, 7.4 mmol, 1.20 equiv.) was suspended in THF (12.0 mL) and *n*-BuLi (3.10 mL, 2.4 M solution in THF, 7.4 mmol, 1.20 equiv.) was added dropwise with stirring under N_2 at -78 °C. After the reaction was stirred 1h at 30 °C, aldehyde (6.20 mmol, 1.00 equiv.) in THF (4.00 mL) was added dropwise at -78 °C. Then, the reaction was stirred for 12 h at 30 °C. The reaction was quenched with H_2O (10.0 mL) and extracted 3 times with ether (10.0 mL). The combined organic layers were dried over $MgSO_4$. The filtrate was concentrated in vacuo and the residue was purified by chromatography on silica gel to afford the corresponding styrenes.

V. Supplementary Method 4. General procedure for synthetic transformation

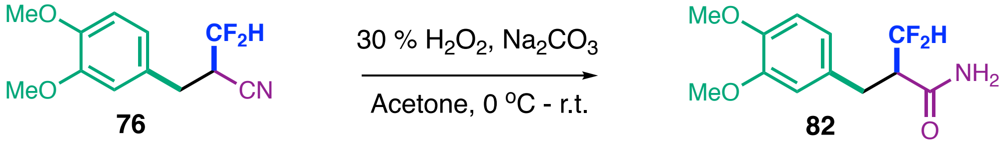

The Procedure for the preparation of 82: To a stirred solution of **76** (48.2 mg, 0.2 mmol, 1.0 equiv.) in acetone (1.5 mL) was added Na_2CO_3 (106.0 mg, 1.0 mmol, 5.0 equiv.) at room temperature. To the suspension was added aqueous H_2O_2 solution (30%, 3.1 mL) at 0 °C. The resulting suspension was stirred at room temperature. After **76** was completely consumed by TLC analysis, water was added, and the resulting mixture was extracted with EtOAc. The combined organic layers were washed with water and brine, dried over Na_2SO_4, and filtered. The filtrate was concentrated under reduced pressure, and the resulting residue was purified by flash column chromatography (EtOAc/hexanes = 1/1) to afford the desired product **82** (41.5 mg, 80%) as a white solid.

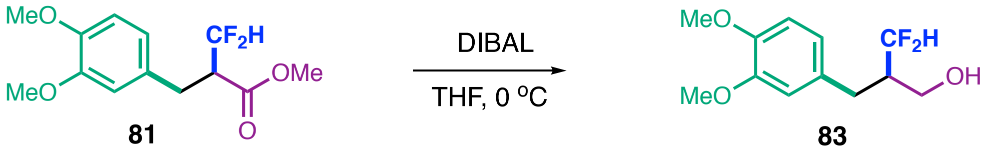

The Procedure for the preparation of 83: A solution of diisobutylaluminum hydride (1.0 M in heptane, 660 μL, 0.66 mmol, 3.3 equiv) was added to **81** (0.2 mmol, 56 mg, 1.0 equiv.) in THF (1.0 mL) at 0 °C. After 1.5 hours, water (1.5 mL) was added dropwise, and the resulting suspension was extracted with diethyl ether (3 × 10 mL). The organic layers were dried with anhydrous Na_2SO_4 and concentrated under reduced pressure. The residue was purified by flash column chromatography on silica gel (40% EtOAc in hexanes) to give **83** (42 mg, 85%) as a colorless oil.

VI. Procedure for additional experiments

1. ^{19}F NMR analyses of a mixture of $(DMPU)_2Zn(CF_2H)_2$ and terpyridine.

Conditions: $(DMPU)_2Zn(CF_2H)_2$ (0.04 mmol, 17.4 mg), DMSO-*d*$_6$ (500 μL), and terpyridine (0.04 mmol, 9.4 mg or 0.02 mmol, 4.7 mg). ^{19}F NMR directly recorded after mixing at room temperature.

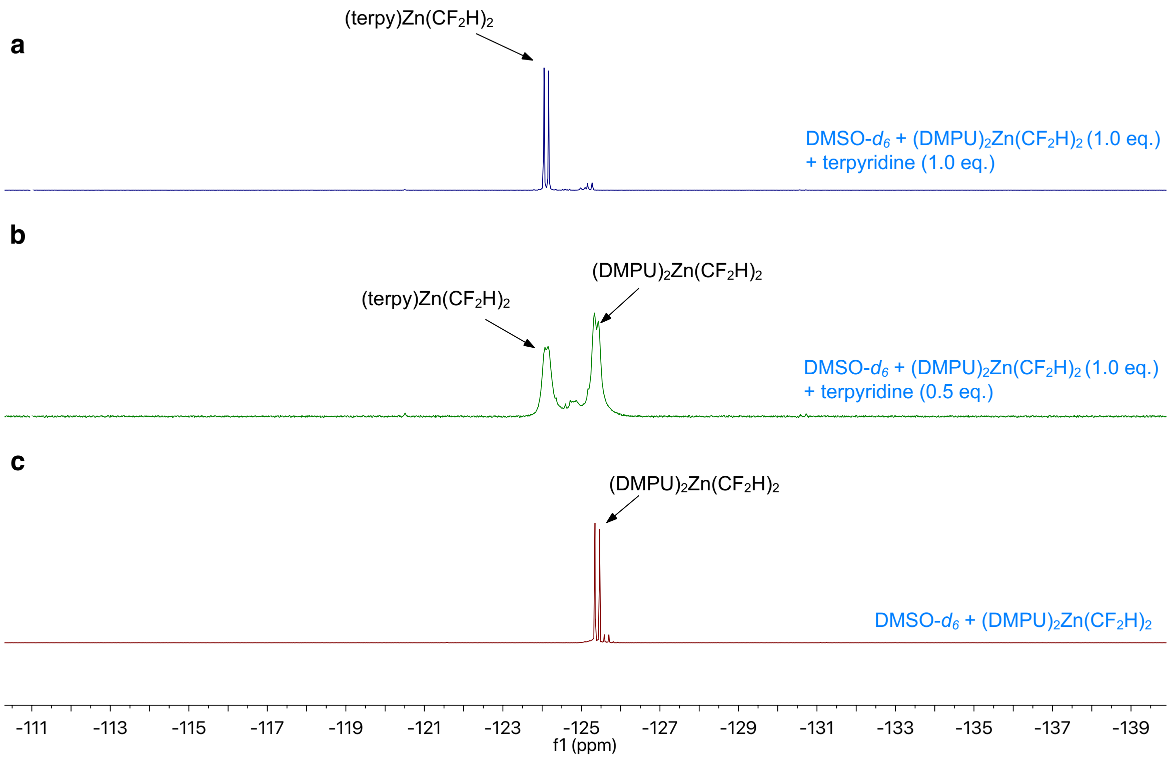

Supplementary Fig. 1. ^{19}F NMR analysis of the reaction between $(DMPU)_2Zn(CF_2H)_2$ and terpyridine. **a**. DMSO-d$_6$ (500 µL), $(DMPU)_2Zn(CF_2H)_2$ (0.04 mmol, 1.0 eq.) and terpyridine (1.0 eq.). **b**. DMSO-d$_6$ (500 µL), $(DMPU)_2Zn(CF_2H)_2$ (0.04 mmol, 1.0 eq.) and terpyridine (0.5 eq.) Note: The broadening the peaks suggests the fast equilibrium between the two species. **c**. DMSO-d$_6$ (500 µL) and $(DMPU)_2Zn(CF_2H)_2$ (0.04 mmol, 1.0 eq.).

Comment: The above spectra indicates that the terpyridine ligand coordinated with $(DMPU)_2Zn(CF_2H)_2$ reagent to form the $(terpy)Zn(CF_2H)_2$ reagent in DMSO.

2. Experimental study of Zn to Cu transmetalation.

Conditions: $(DMPU)_2Zn(CF_2H)_2$ (0.04 mmol, 17.4 mg), DMSO-d_6 (500 μL), and CuCl (0.04 mmol, 4.0 mg). **^{19}F NMR** directly recorded after mixing at room temperature.

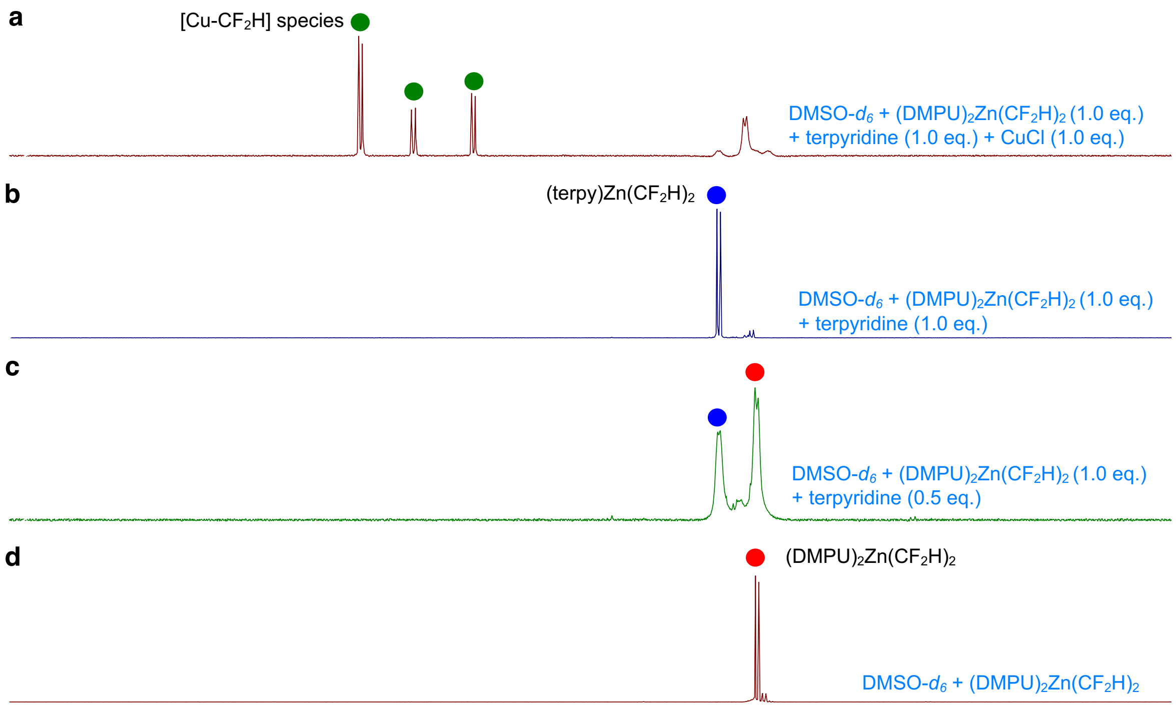

Supplementary Fig. 2. ^{19}F NMR analysis of the reaction between $(DMPU)_2Zn(CF_2H)_2$, terpy and CuCl. a. DMSO-d_6 (500 μL), $(DMPU)_2Zn(CF_2H)_2$ (0.04 mmol, 1.0 eq.) and CuCl (1.0 eq.) b. DMSO-d_6 (500 μL), $(DMPU)_2Zn(CF_2H)_2$ (0.04 mmol, 1.0 eq.) and terpyridine (1.0 eq.) c. DMSO-d_6 (500 μL), $(DMPU)_2Zn(CF_2H)_2$ (0.04 mmol, 1.0 eq.) and terpyridine (0.5 eq.) d. DMSO-d_6 (500 μL) and $(DMPU)_2Zn(CF_2H)_2$ (0.04 mmol, 1.0 eq.).

Comment: The above spectra indicates that a transmetalation of a copper(I) catalyst (CuCl) with $(DMPU)_2Zn(CF_2H)_2$ reagent to afford a reactive [Cu-CF_2H] species in DMSO.[8]

2. Radical clock experiments

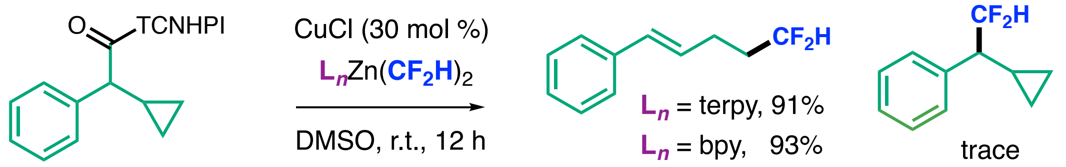

In a nitrogen-filled glovebox, to a 4 mL vial equipped with a stir bar was added terpyridine (0.8 equiv., 18.8 mg) or 2,2'-bipyridine (0.8 equiv., 12.5 mg), $(DMPU)_2Zn(CF_2H)_2$ (0.8 equiv., 35 mg), and 400 μL DMSO. The resulting mixture was stirred at room temperature for 1 min to generate $(terpy)Zn(CF_2H)_2$ or $(bpy)Zn(CF_2H)_2$ in-situ. A different 4 mL vial equipped with a stir bar was sequentially charged with CuCl (30 mol %, 3 mg), RAE (0.1 mmol, 1.0 equiv.) and the DMSO solution of the in-situ formed $(terpy)Zn(CF_2H)_2$ or $(bpy)Zn(CF_2H)_2$. The mixture was stirred at room temperature for 12 h. **Note:** *The yields were determined by ^{19}F NMR spectroscopy; 1-Fluoro-3-nitrobenzene (13.5 mg, 0.095 mmol) was added as internal standard for ^{19}F NMR analysis ($CDCl_3$).*

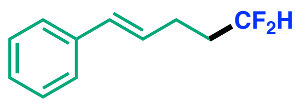

(*E*)-(5,5-difluoropent-1-yl)benzene: MS (EI): *m/z* calcd $C_{11}H_{12}F_2$ $[M]^+$ 182.1, found 182.1.

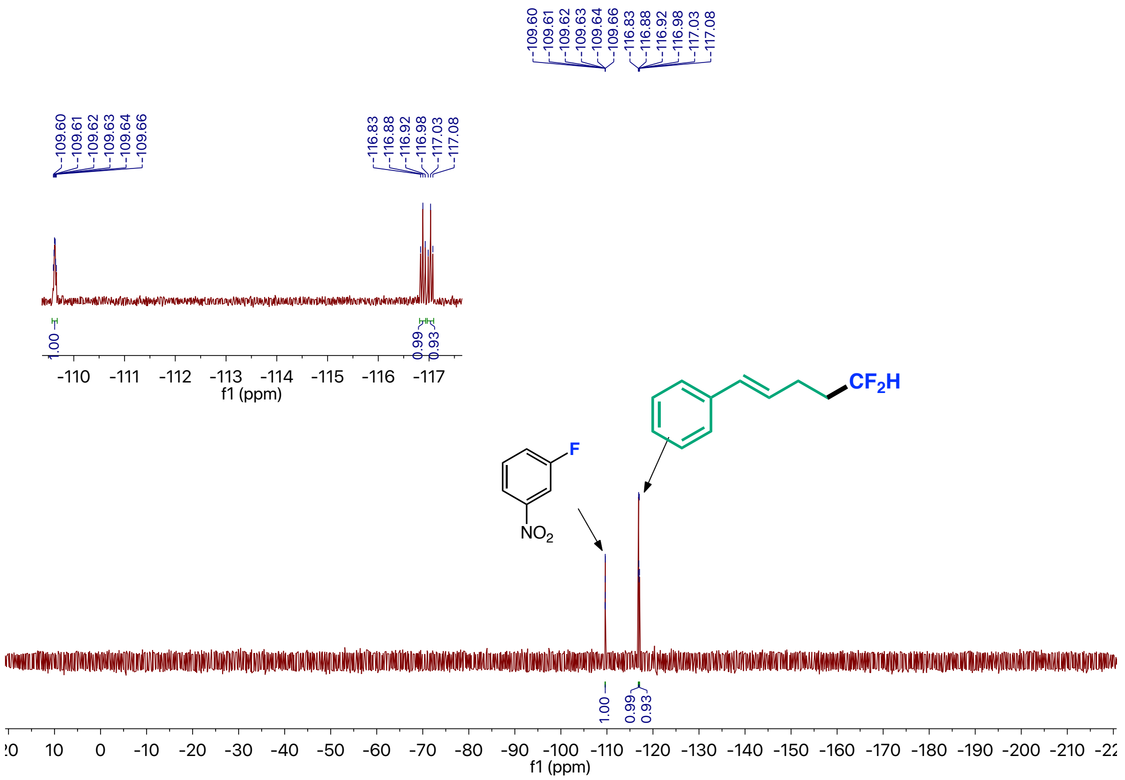

Supplementary Fig. 3. ^{19}F NMR of radical clock experiments. ^{19}F NMR (376 MHz, $CDCl_3$) δ -116.95 (dt, J = 56.4, 17.3 Hz). When using $(terpy)Zn(CF_2H)_2$ as difluoromethyl reagent: the formation of 91% rearranged product supports the intermediacy of the alkyl radicals.

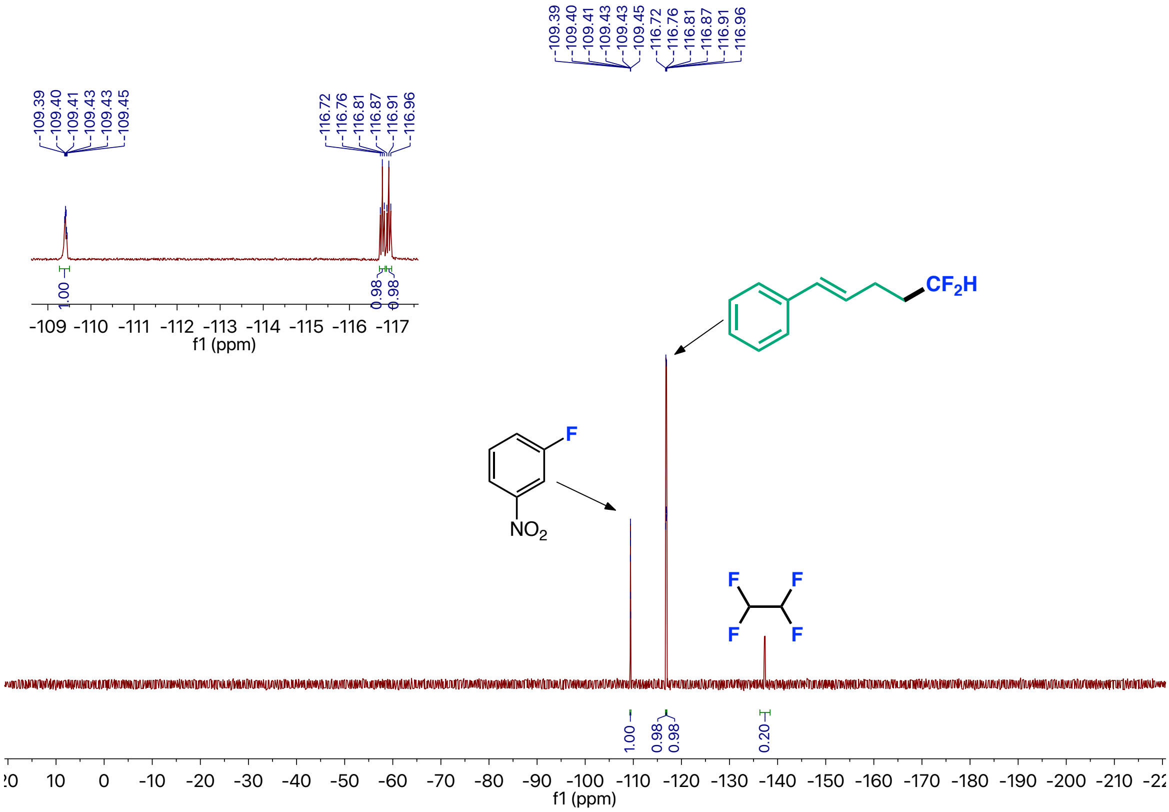

Supplementary Fig. 4. ^{19}F NMR of radical clock experiments. ^{19}F NMR (376 MHz, $CDCl_3$) δ -116.84 (dt, J = 56.4, 17.3 Hz). When using $(bpy)Zn(CF_2H)_2$ as difluoromethyl reagent: the formation of 93% rearranged product supports the intermediacy of the alkyl radicals.

3. Decarboxylative difluoromethylation of acyclic secondary TCNHPI redox-esters

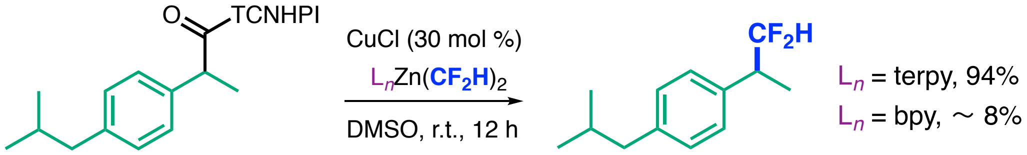

In a nitrogen-filled glovebox, to a 4 mL vial equipped with a stir bar was added terpyridine (0.8 equiv., 18.8 mg) or 2,2'-bipyridine (0.8 equiv., 12.5 mg), $(DMPU)_2Zn(CF_2H)_2$ (0.8 equiv., 35 mg), and 400 μL DMSO. The resulting mixture was stirred at room temperature for 1 min to generate $(terpy)Zn(CF_2H)_2$ or $(bpy)Zn(CF_2H)_2$ in-situ. A different 4 mL vial equipped with a stir bar was sequentially charged with CuCl (30 mol %, 3 mg), ibuprofen redox-ester (0.1 mmol, 49 mg, 1.0 equiv.) and the DMSO solution of the in-situ formed $(terpy)Zn(CF_2H)_2$ or $(bpy)Zn(CF_2H)_2$. The mixture was stirred at room temperature for 12 h.**Note:** *The yields were determined by ^{19}F NMR spectroscopy; 1-Fluoro-3-nitrobenzene (24.8 mg, 0.18 mmol) was added as internal standard for ^{19}F NMR analysis ($CDCl_3$).*

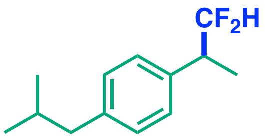

1-(1,1-difluoropropan-2-yl)-4-isobutylbenzene: *m/z* calcd $C_{13}H_{18}F_2$ $[M]^+$ 212.1, found 212.1.

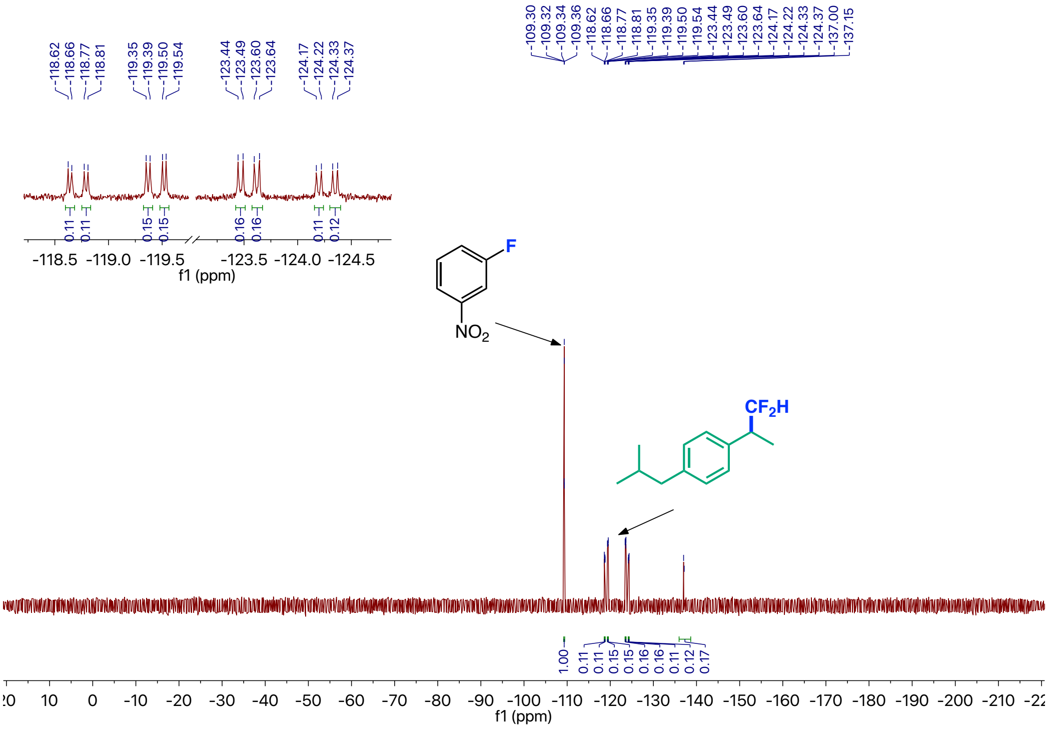

Supplementary Fig. 5. ^{19}F NMR of the difluoromethylation reaction of ibuprofen redox-ester as electrophile. ^{19}F NMR (376 MHz, $CDCl_3$) δ -119.08 (ddd, J = 274.3, 56.4, 12.9 Hz, 1F), -123.91 (ddd, J = 274.7, 56.9, 17.2 Hz, 1F). When using $(terpy)Zn(CF_2H)_2$ as difluoromethyl reagent in this reaction: the formation of desired difluoromethylation product in 94% yield.

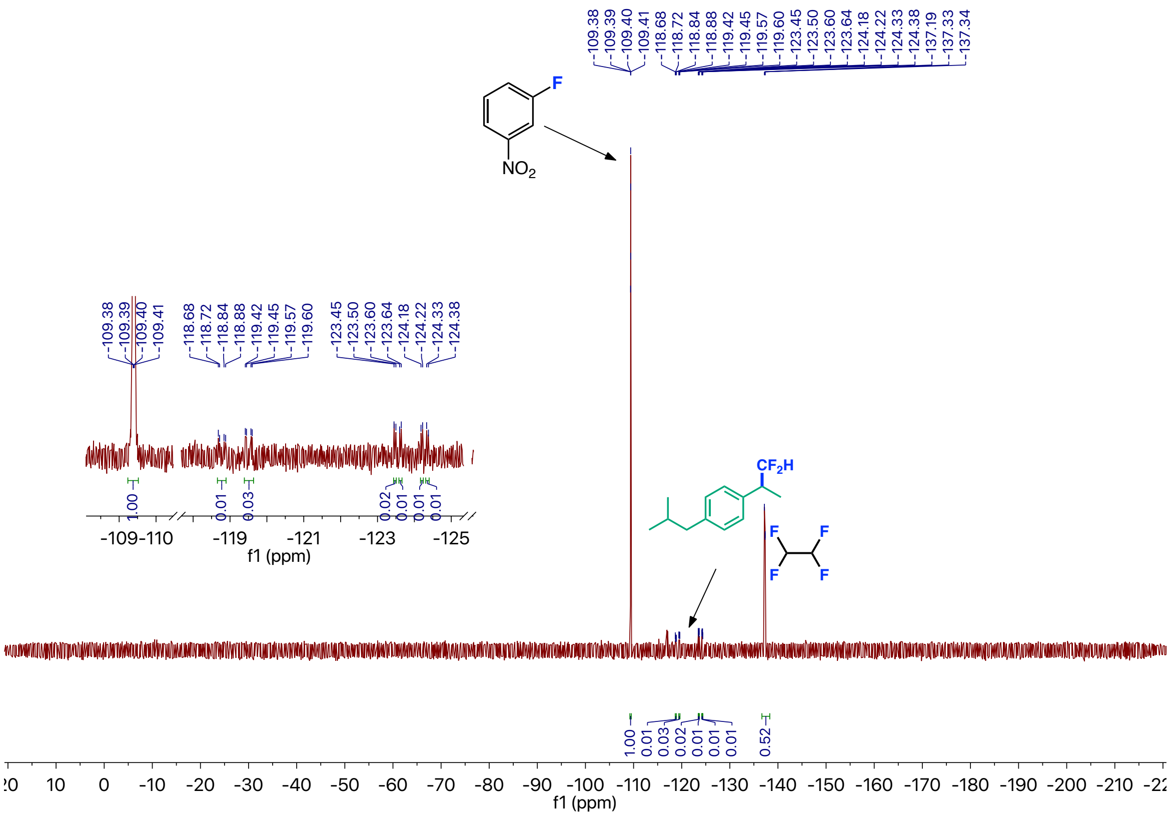

Supplementary Fig. 6. ^{19}F NMR of the difluoromethylation reaction of ibuprofen redox-ester as electrophile. When using (bpy)Zn(CF_2H)$_2$ as difluoromethyl reagent in this reaction: the formation of desired difluoromethylation product only in 8% yield. We observed the formation of 1,1,2,2-tetrafluoroethane as a major side product in the below ^{19}F NMR spectra.

The mixtures of the reaction have also been analyzed by GC-MS.

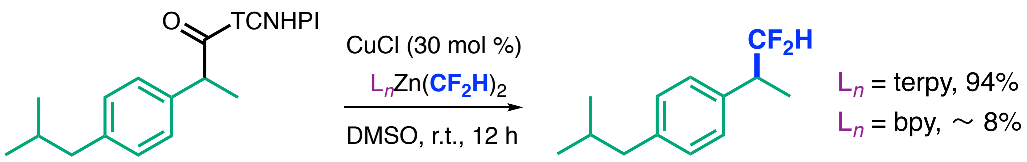

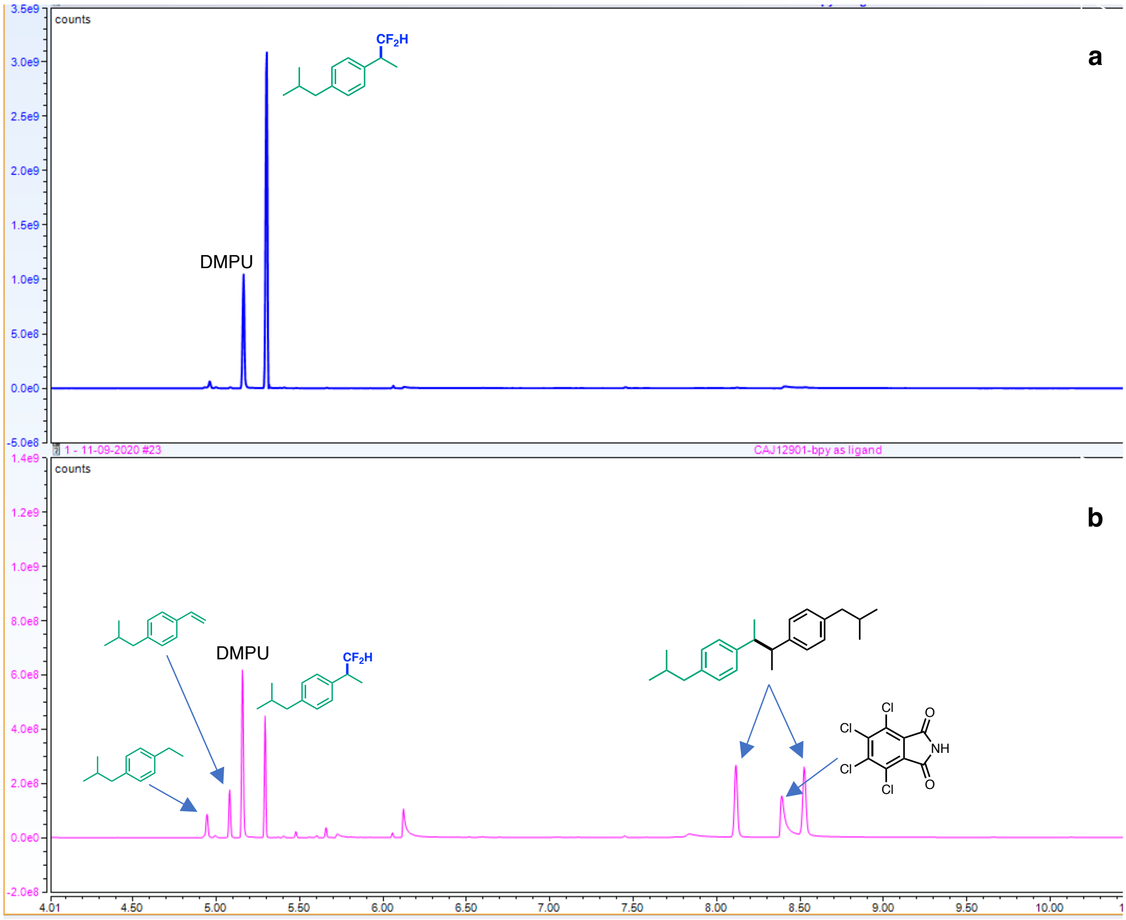

Supplementary Fig. 7. GC spectra of the difluoromethylation reaction of ibuprofen redox-ester as electrophile: a. terpyridine as ligand in the decarboxylative difluoromethylation reaction. **b**. bipyridine as ligand in the decarboxylative difluoromethylation reaction.

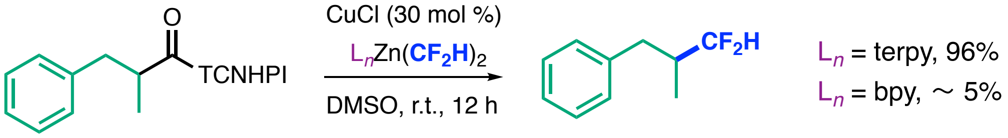

In a nitrogen-filled glovebox, to a 4 mL vial equipped with a stir bar was added terpyridine (0.8 equiv., 18.8 mg) or 2,2'-bipyridine (0.8 equiv., 12.5 mg), $(DMPU)_2Zn(CF_2H)_2$ (0.8 equiv., 35 mg). and 400 μL DMSO. The resulting mixture was stirred at room temperature for 1 min to generate $(terpy)Zn(CF_2H)_2$ or $(bpy)Zn(CF_2H)_2$ in-situ. A different 4 mL vial equipped with a stir bar was sequentially charged with CuCl (30 mol %, 3 mg), 2-benzylpropionic redox-ester (0.1 mmol, 45 mg, 1.0 equiv.) and the DMSO solution of the in-situ formed $(terpy)Zn(CF_2H)_2$ or $(bpy)Zn(CF_2H)_2$. The mixture was stirred at room temperature for 12 h. **Note:** *The yields were determined by ^{19}F NMR spectroscopy; 1-Fluoro-3-nitrobenzene (23 mg, 0.16 mmol) was added as internal standard for ^{19}F NMR analysis ($CDCl_3$).*

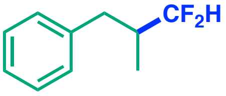

(3,3-difluoro-2-methylpropyl)benzene: *m/z* calcd $C_{10}H_{12}F_2$ $[M]^+$ 170.1, found 170.1.

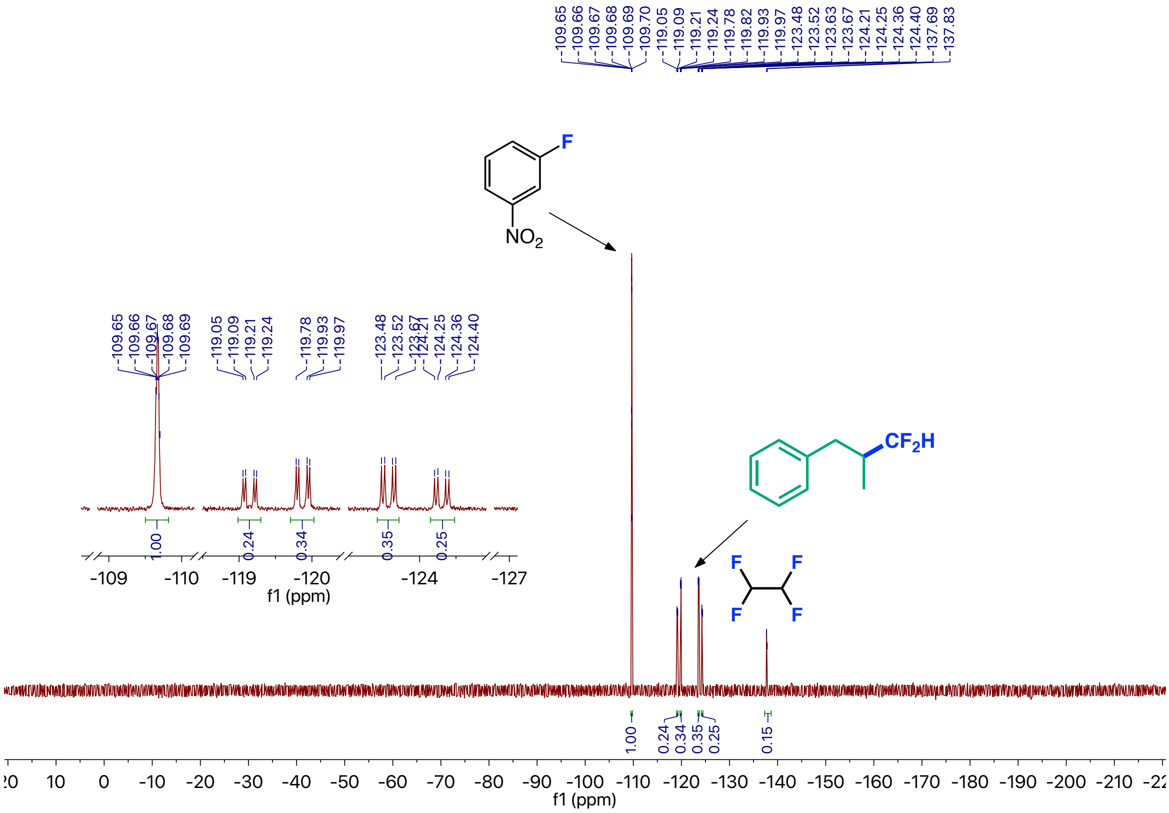

Supplementary Fig. 8. ^{19}F NMR of the difluoromethylation reaction of 2-benzylpropionic redox-ester as electrophile. ^{19}F NMR (376 MHz, $CDCl_3$) δ -119.51 (ddd, J = 274.5, 56.5, 13.2 Hz, 1F), -123.94 (ddd, J = 274.6, 56.9, 16.6 Hz, 1F). When using $(terpy)Zn(CF_2H)_2$ as difluoromethyl reagent in this reaction: the formation of desired difluoromethylation product in 96% yield.

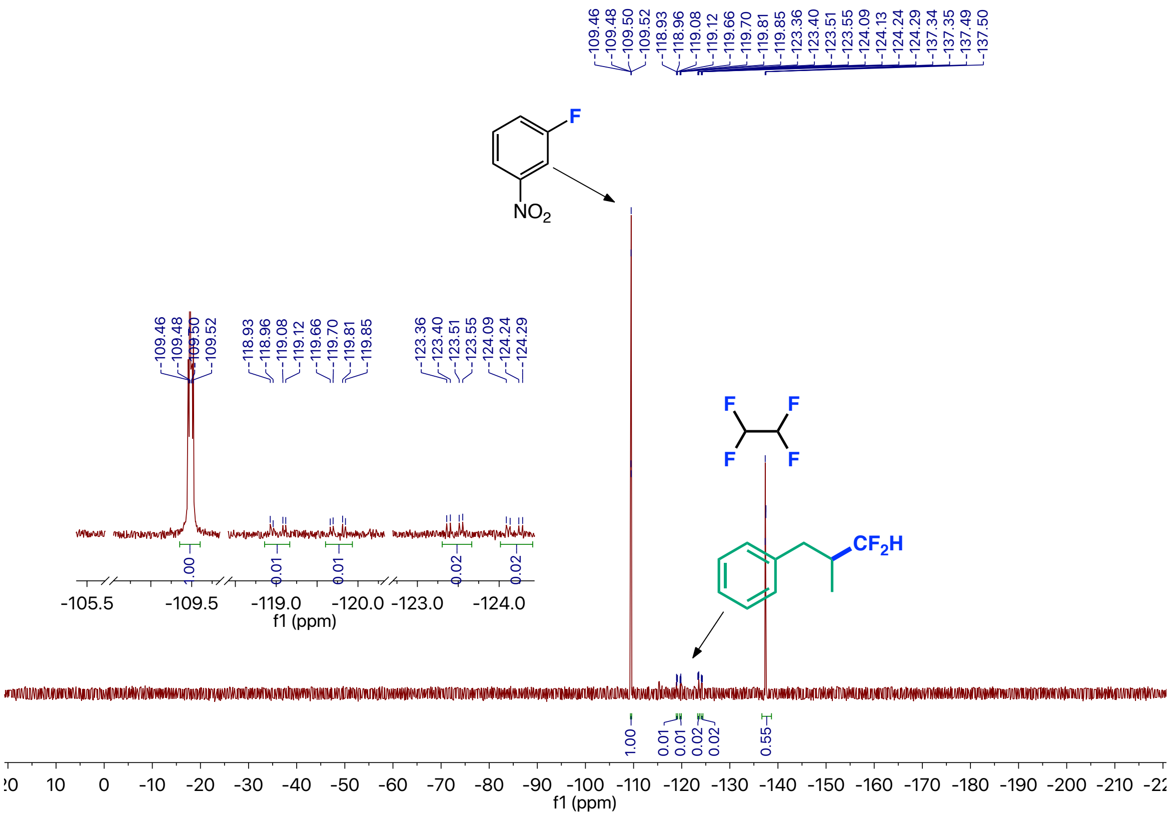

Supplementary Fig. 9. ^{19}F NMR of the difluoromethylation reaction of 2-benzylpropionic redox-ester as electrophile. When using (bpy)Zn(CF_2H)$_2$ as difluoromethyl reagent in this reaction: the formation of desired difluoromethylation product only in 5% yield. We observed the formation of 1,1,2,2-tetrafluoroethane as a major side product in this reaction from the below ^{19}F NMR spectra.

The mixtures of the reaction have also been analyzed by GC-MS.

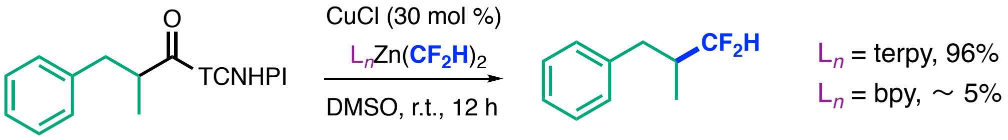

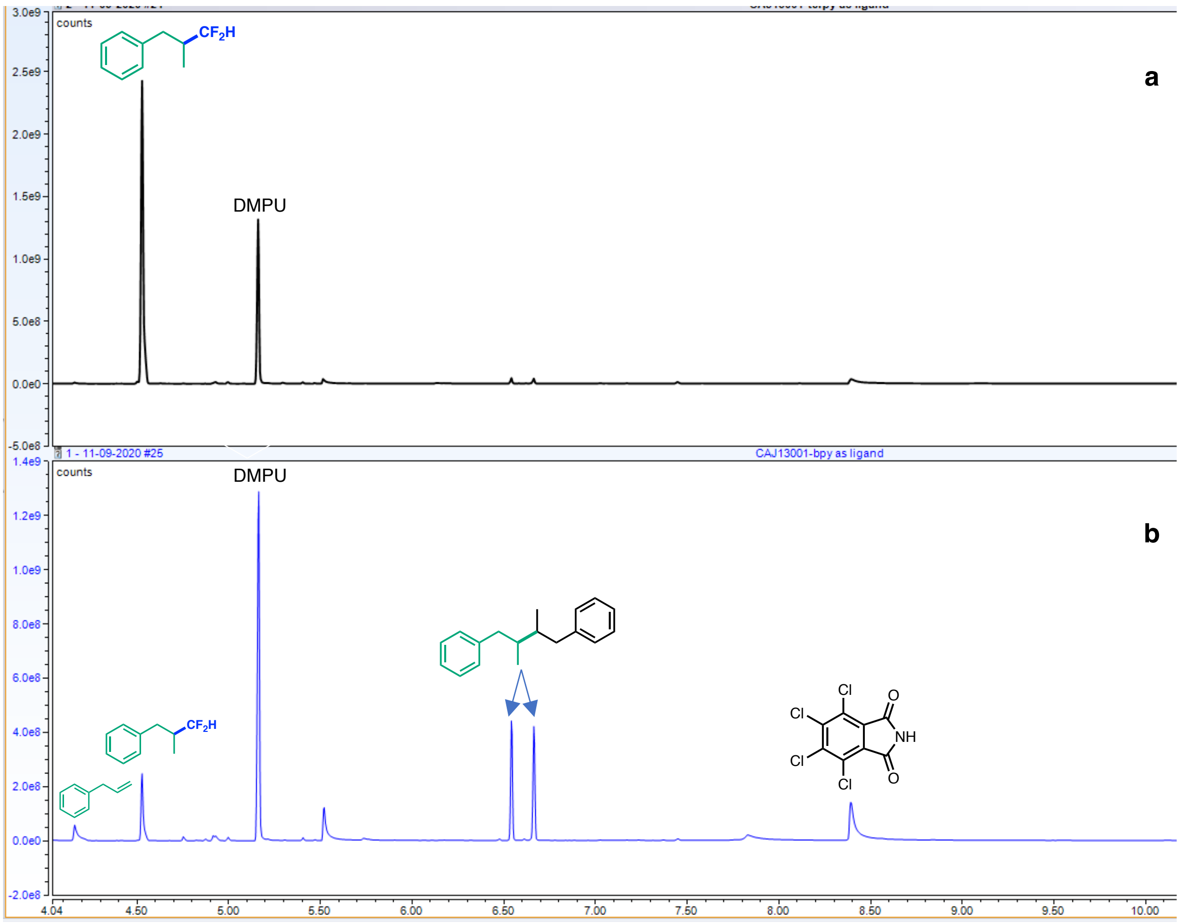

Supplementary Fig. 10. GC spectra of the difluoromethylation reaction of 2-benzylpropionic redox-ester as electrophile. **a**. terpyridine as ligand in the decarboxylative difluoromethylation reaction. **b**. bipyridine as ligand in the decarboxylative difluoromethylation reaction.

4. Radical-relayed difluoromethylation of tertiary TCNHPI redox-ester

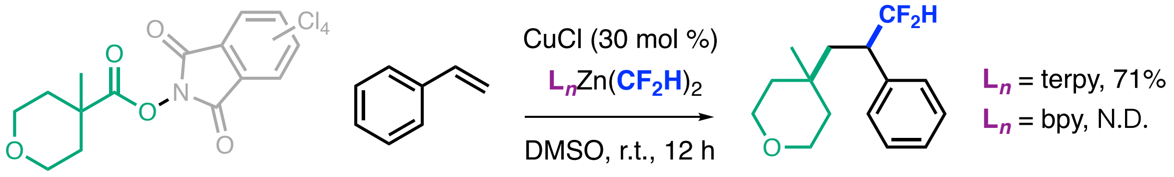

In a nitrogen-filled glovebox, to a 4 mL vial equipped with a stir bar was added terpyridine (0.8 equiv., 18.8 mg) or 2,2'-bipyridine (0.8 equiv., 12.5 mg), $(DMPU)_2Zn(CF_2H)_2$ (0.8 equiv., 35 mg), and 320 μL DMSO. The resulting mixture was stirred at room temperature for 1 min to generate $(bpy)Zn(CF_2H)_2$ in-situ. A different 4 mL vial equipped with a stir bar was sequentially charged with CuCl (30 mol %, 3 mg), RAEs (0.1 mmol, 1.0 equiv., 43 mg), the DMSO solution of the in-situ formed $(bpy)Zn(CF_2H)_2$, and alkene (0.75 mmol, 3.0 equiv., 31.2 mg) in 200 μL DMSO. The resultant mixture was stirred at room temperature for 12 h.

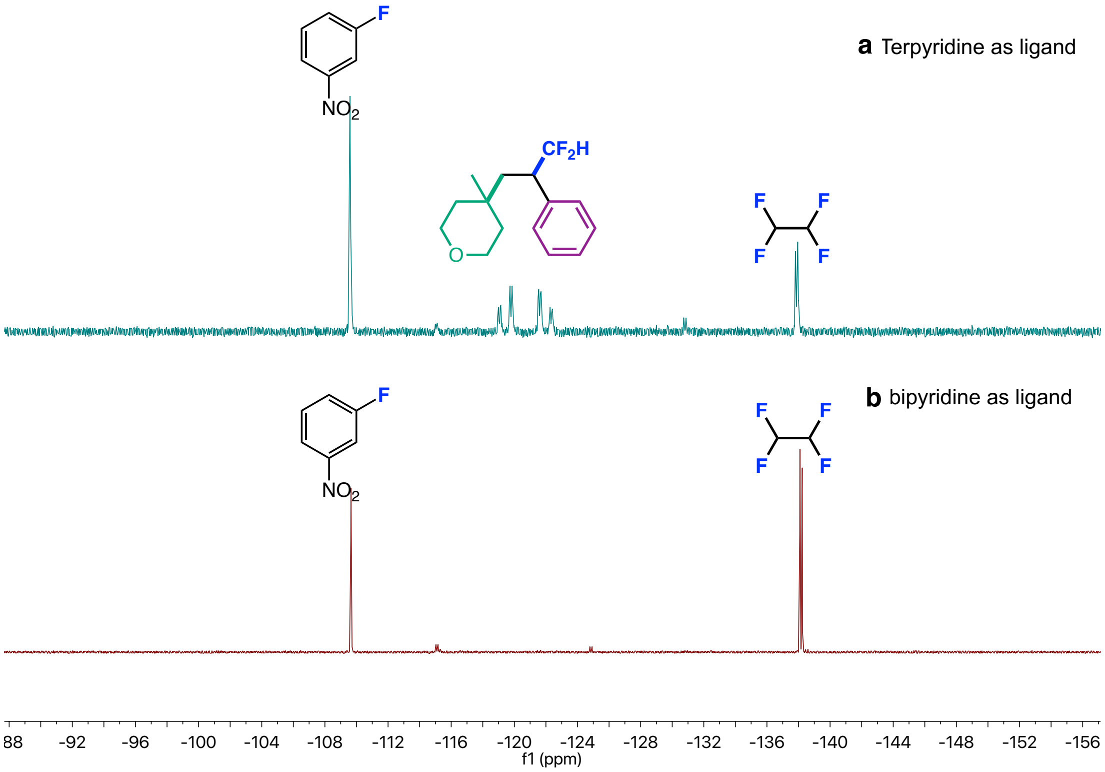

Supplementary Fig. 11. ^{19}F NMR of radical-relayed difluoromethylation of tertiary TCNHPI redox-ester. a. The use of terpyridine as ligand for radical-relayed difluoromethylation, **b.** The use of bipyridine as ligand for radical-relayed difluoromethylation, The yields were determined by ^{19}F NMR spectroscopy; 1-Fluoro-3-nitrobenzene was added as internal standard for ^{19}F NMR analysis ($CDCl_3$).

Based on the ^{19}F NMR results, we observed the formation of radical-relayed difluoromethylation product in 76% yield when using $(terpy)Zn(CF_2H)_2$ as difluoromethyl reagent. On the other hand, no desired product was formed when using $(bpy)Zn(CF_2H)_2$ as difluoromethyl reagent (for more details see below ^{19}F NMR spectra).

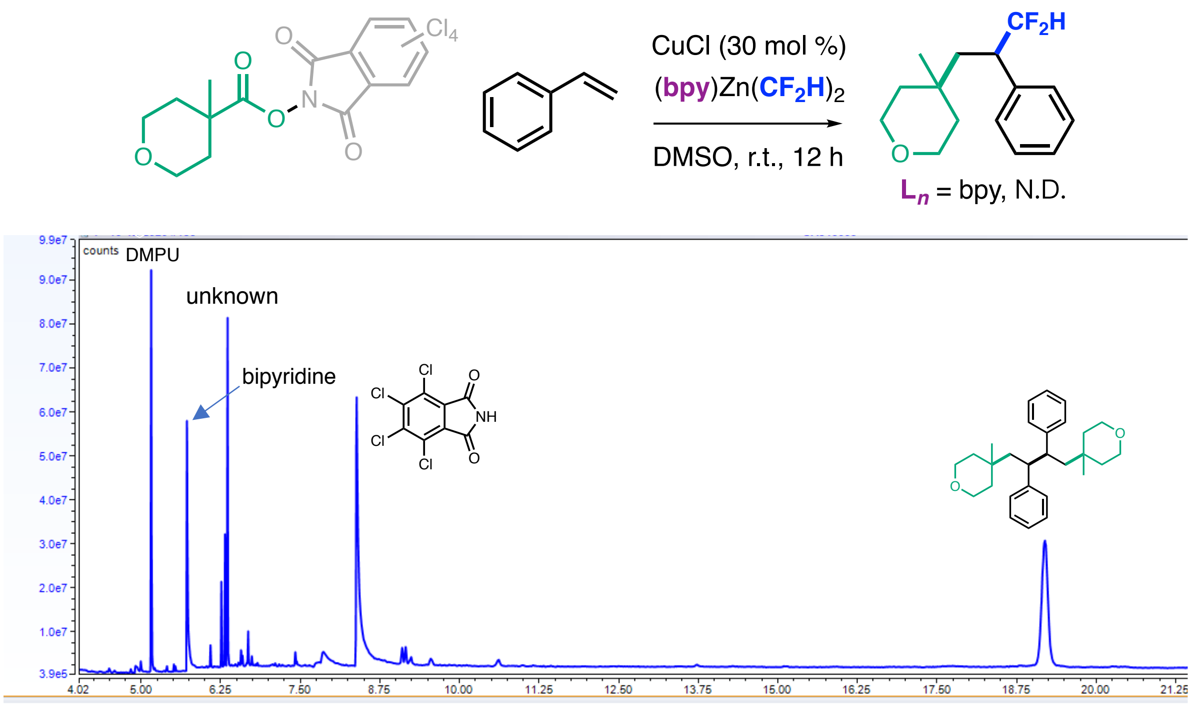

Supplementary Fig. 12. GC spectra of radical-relayed difluoromethylation using bipyridine as ligand. The GC-MS analysis confirms the formation of the dimerization product as a major side product in this reaction.

VII. DFT Calculations of Cu/bpy system catalyzed decarboxylative difluoromethylation of secondary cyclic RAEs

Our previous work has shown that copper/bipyridine system could catalyze efficient decarboxylative difluoromethylation of the RAEs derived from secondary cyclic carboxylic acids, while the RAEs derived from secondary benzylic carboxylic acids were much less effective. We attributed this result to fast combination rate of a secondary cyclic alkyl radical with **Cu-2** complex. To confirm this hypothesis, we conducted the DFT Calculation on the reaction between a cyclohexyl radical and **Cu-2**.

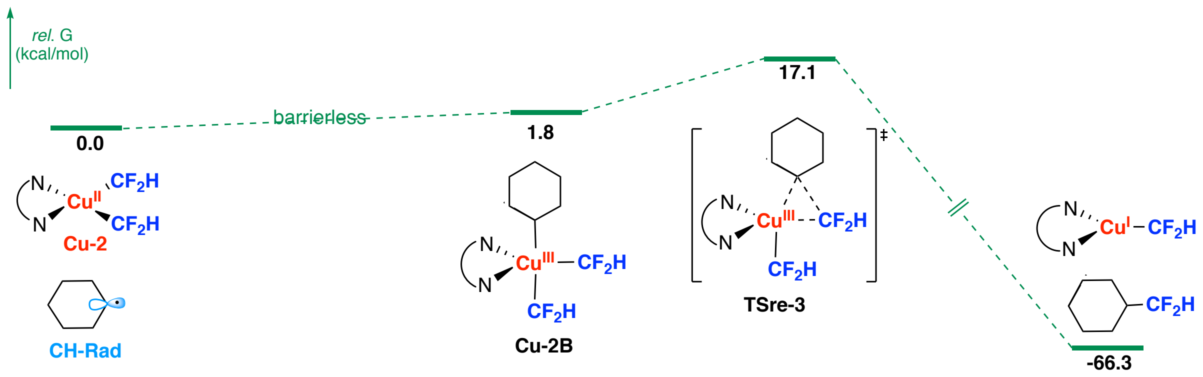

Supplementary Fig. 13. Energy profile for the recombination of a cyclohexyl radical (CH-Rad) with Cu-2. The recombination of a cyclohexyl radical with **Cu-2** was a barrierless pathway, in contrast with a high barrier required for the recombination of a secondary benzylic radical with **Cu-2**. This is consistent with our experimental results that copper/bipyridine system could catalyze the decarboxylative difluoromethylation of RAEs derived from secondary cyclic carboxylic acids, but was not effective for while the RAEs derived from secondary benzylic carboxylic acids.

VIII. Reaction optimization

Supplementary Table 1: Evaluation of different ligands, yield determined by ^{19}F NMR.[a]

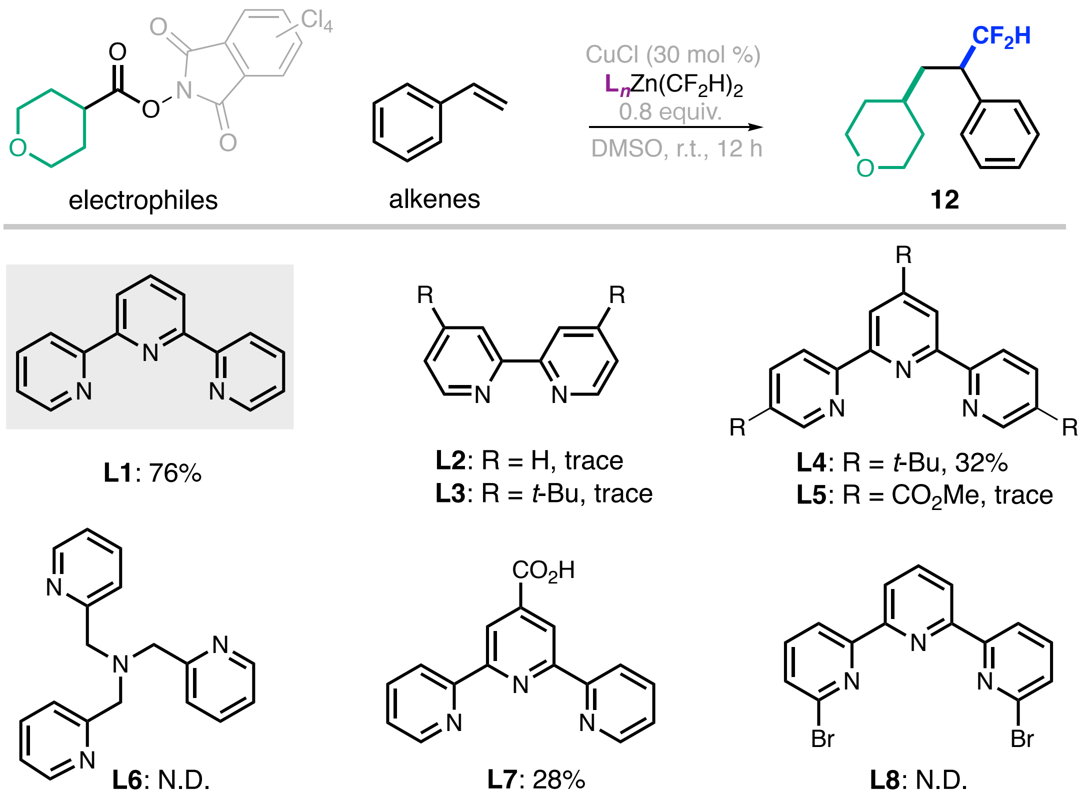

Supplementary Table 2: Evaluation of different cooper source and solvents.[a]

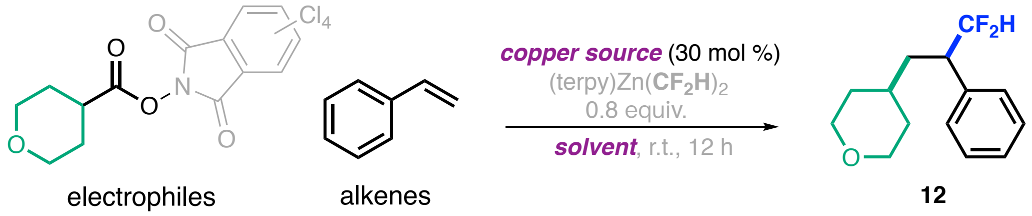

Entry	Copper source	Solvent	Yield (%)[b]
**1**	**CuCl**	**DMSO**	**76**
2	CuI	DMSO	64
3	CuBr	DMSO	62
4	CuCN	DMSO	trace
5	CuOAc	DMSO	39
6	$[Cu(OTf)]_2 \cdot C_6H_6$	DMSO	52
7	Cu(Tc)	DMSO	36
8	$Cu(OTf)_2$	DMSO	42
9	$Cu(OAc)_2$	DMSO	33
10	CuCl	DMSO/THF (3:1)	59
11	CuCl	DMSO/MeCN (3:1)	65
12	CuCl (20 mol %)	DMSO	48
13	CuCl	DMPU	54
14	CuCl	DMI	49
15	CuCl	DMF	18

[a]RAEs (0.1 mmol, 1.0 equiv.), styrene (0.3 mmol, 3.0 equiv.), (terpy)Zn(CF_2H)$_2$ (0.08 mmol, 0.8 equiv.), and [Cu] (30 mol %) in DMSO (0.4 mL). [b]Yields were determined by ^{19}F NMR using 1-fluoro-3-nitrobenzene as the internal standard.

Note: *The yields were determined by ^{19}F NMR spectroscopy; 1-Fluoro-3-nitrobenzene (26.8 mg, 0.19 mmol) was added as internal standard for ^{19}F NMR analysis (in $CDCl_3$).*

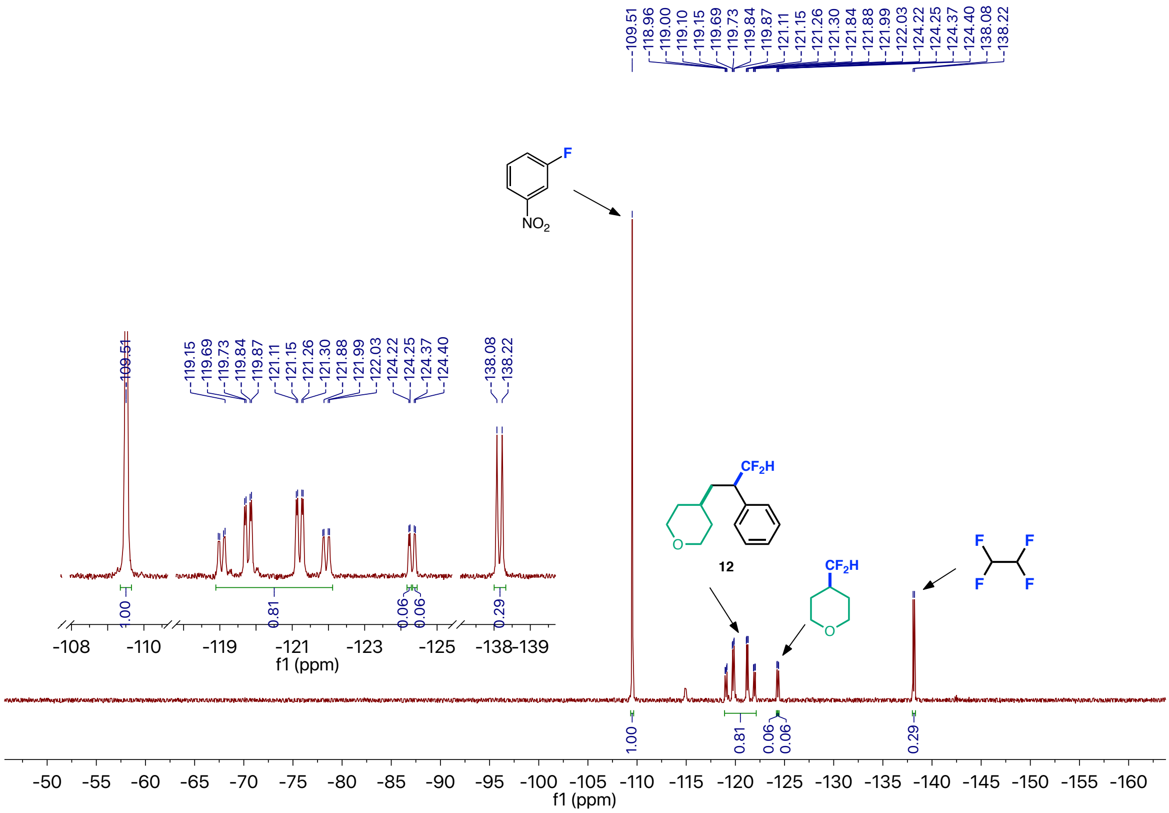

Supplementary Fig. 14. A representative ^{19}F-NMR spectra of the optimization studies (Supplementary Table 2, entry 1). ^{19}F NMR (376 MHz, $CDCl_3$) δ -119.46 (ddd, J = 274.9, 56.9, 15.5 Hz), -121.55 (ddd, J = 274.3, 56.7, 14.9 Hz), -124.33 (dd, J = 56.6, 13.2 Hz).

Supplementary Table 3: Optimization of copper-catalyzed difluoromethyl-arylation of alkenes.[a]

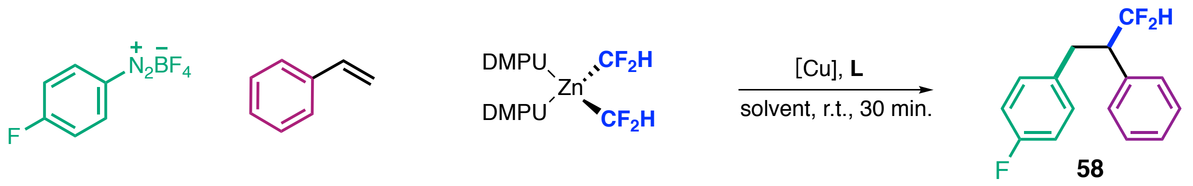

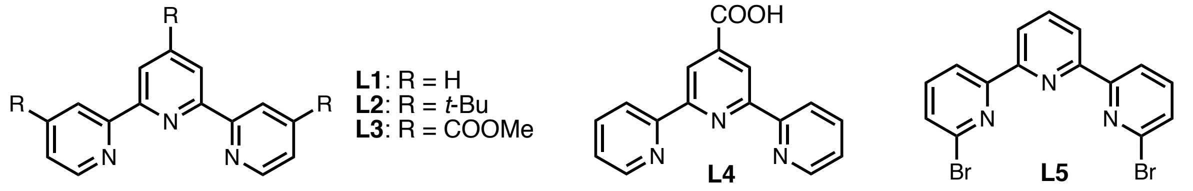

Entry	[Cu] (mol %)	**L** (mol %)	Solvent	Yield (%)[b]
1	CuCl (30)	**L1** (50)	DMSO	30
2	$[Cu(MeCN)_4]BF_4$ (30)	**L1** (50)	DMSO	39
3	CuOAc (30)	**L1** (50)	DMSO	34
4	CuI (30)	**L1** (50)	DMSO	31
5	$Cu(OTf)_2$ (30)	**L1** (50)	DMSO	30
6	$Cu(acac)_2$ (30)	**L1** (50)	DMSO	33
7	$[Cu(MeCN)_4]PF_6$ (30)	**L1** (50)	DMSO	37
8	$[Cu(MeCN)_4]BF_4$ (10)	**L1** (20)	DMSO	41
9	$[Cu(MeCN)_4]BF_4$ (10)	**L2** (20)	DMSO	28
10	$[Cu(MeCN)_4]BF_4$ (10)	**L3** (20)	DMSO	38
11	$[Cu(MeCN)_4]BF_4$ (10)	**L4** (20)	DMSO	41
12	$[Cu(MeCN)_4]BF_4$ (10)	**L5** (20)	DMSO	37
13	$[Cu(MeCN)_4]BF_4$ (10)	**L1** (10)	DMSO	35
**14**	**$[Cu(MeCN)_4]BF_4$ (20)**	**L1 (20)**	**DMSO**	**65**
15	$[Cu(MeCN)_4]BF_4$ (20)	**L1** (20)	DMI	trace
16	No	**L1** (20)	DMSO	N.D.
17	$[Cu(MeCN)_4]BF_4$ (20)	No	DMSO	21

[a]Diazonium salts (0.1 mmol, 1.0 equiv.), styrene (0.3 mmol, 3.0 equiv.), $(DMPU)_2Zn(CF_2H)_2$ (0.08 mmol, 0.8 equiv.), [Cu] (20 mol %), and **L** (20 mol %) in DMSO (0.4 mL). [b]Yields were determined by ^{19}F NMR using 1-fluoro-3-nitrobenzene as the internal standard.

IX. Supplementary Method 5. Synthesis and characterizations of new compounds

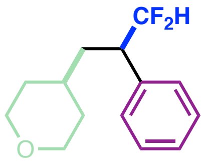

Using general procedure A. **4-(3,3-difluoro-2-phenylpropyl)tetrahydro-2H-pyran (12)**: 0.25 mmol scale, 39.5 mg, 66%, colorless oil, 5% ethyl acetate (EA) in hexane, $R_f = 0.15$.

^{1}H NMR (400 MHz, $CDCl_3$) δ 7.42 – 7.30 (m, 3H), 7.26 (d, J = 7.5 Hz, 2H), 5.83 (td, J = 56.7, 3.7 Hz, 1H), 3.92 (t, J = 12.8 Hz, 2H), 3.33 – 3.08 (m, 3H), 1.93 – 1.71 (m, 2H), 1.69 – 1.61 (m, 1H), 1.49 (d, J = 9.4 Hz, 1H), 1.40 – 1.25 (m, 3H); ^{13}C NMR (101 MHz, $CDCl_3$) δ 136.9 (t, J = 4.0 Hz), 128.9, 128.9, 127.8, 118.2 (t, J = 245.3 Hz), 67.9, 67.8, 46.9 (t, J = 19.8 Hz), 35.2 (t, J = 4.0 Hz), 33.9, 32.2, 31.8; ^{19}F NMR (376 MHz, $CDCl_3$) δ -118.49 (ddd, J = 275.5, 56.7, 14.4 Hz, 1F), -122.30 (ddd, J = 275.6, 56.9, 16.5 Hz, 1F); MS (EI): $[M]^+$ m/z calcd $C_{14}H_{18}F_2O$ 240.1, found 240.1; HRMS (m/z): (EI) $[M-HF]^+$ calcd for $C_{14}H_{17}FO$, 220.1263; found 220.1262.

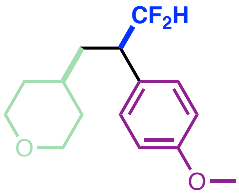

Using general procedure A. **4-(3,3-difluoro-2-(4-methoxyphenyl)propyl)tetrahydro-2H-pyran (13)**: 0.25 mmol scale, 44 mg, 65%, colorless oil, 7% EA in hexane, $R_f = 0.12$.

^{1}H NMR (400 MHz, $CDCl_3$) δ 7.15 (d, J = 8.3 Hz, 2H), 6.89 (d, J = 8.2 Hz, 2H), 5.77 (td, J = 56.9, 3.9 Hz, 1H), 3.89 (t, J = 11.8 Hz, 2H), 3.81 (s, 3H), 3.24 (q, J = 12.1 Hz, 2H), 3.14 – 2.99 (m, 1H), 1.79 – 1.68 (m, 2H), 1.61 (d, J = 12.4 Hz, 1H), 1.46 (d, J = 9.2 Hz, 1H), 1.36 – 1.20 (m, 3H); ^{13}C NMR (101 MHz, $CDCl_3$) δ 159.2, 129.9, 128.6 (t, J = 3.72 Hz), 118.3 (t, J = 243.5

Hz), 114.3, 67.9, 67.8, 55.3, 45.9 (t, J = 19.5 Hz), 35.2 (t, J = 4.0 Hz), 33.9, 32.1, 31.8; ^{19}F NMR (376 MHz, $CDCl_3$) δ -118.83 (ddd, J = 274.7, 56.8, 14.5 Hz, 1F), -122.39 (ddd, J = 274.7, 56.9, 16.3, 1F); HRMS (EI): m/z $[M]^+$ calcd for $C_{15}H_{20}F_2O_2$ 270.1431, found 270.1436.

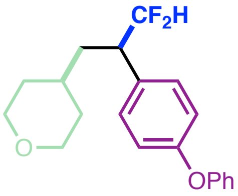

Using general procedure A. **4-(3,3-difluoro-2-(4-phenoxyphenyl)propyl)tetrahydro-2H-pyran (14):** 0.25 mmol scale, 50 mg, 60%, colorless oil, 5% EA in Hexane, R_f = 0.11.

^{1}H NMR (400 MHz, $CDCl_3$) δ 7.36 (t, J = 7.6 Hz, 2H), 7.19 (d, J = 8.2 Hz, 2H), 7.13 (t, J = 7.3 Hz, 1H), 7.01 (dd, J = 21.2, 8.1 Hz, 4H), 5.80 (td, J = 56.8, 3.5 Hz, 1H), 3.91 (t, J = 11.2 Hz, 2H), 3.27 (q, J = 12.1 Hz, 2H), 3.11 (dtt, J = 19.1, 9.1, 3.8 Hz, 1H), 1.76 (tq, J = 13.9, 7.5, 5.1 Hz, 2H), 1.62 (d, J = 12.5 Hz, 1H), 1.48 (d, J = 10.2 Hz, 1H), 1.39 – 1.22 (m, 3H); ^{13}C NMR (101 MHz, $CDCl_3$) δ 157.1, 156.9, 131.2 (t, J = 4.0 Hz), 130.2, 129.9, 123.7, 119.3, 118.8, 118.1 (t, J = 244.7 Hz), 67.9, 67.8, 46.1 (t, J = 19.9 Hz), 35.3 (t, J = 4.0 Hz), 33.9, 32.2, 31.8; ^{19}F NMR (376 MHz, $CDCl_3$) δ -118.91 (ddd, J = 275.4, 56.7, 14.7 Hz, 1F), -122.20 (ddd, J = 275.4, 56.9, 16.1 Hz, 1F); HRMS (EI): m/z calcd $C_{20}H_{22}F_2O_2$ $[M]^+$ 332.1588, found 332.1595.

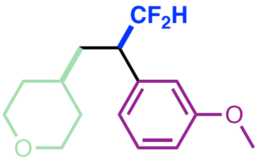

Using general procedure A. **4-(3,3-difluoro-2-(3-methoxyphenyl)propyl)tetrahydro-2H-pyran (15):** 0.25 mmol scale, 35 mg, 52%, colorless oil, 7% EA in hexane, R_f = 0.15.

^{1}H NMR (400 MHz, $CDCl_3$) δ 7.30 (t, J = 7.8 Hz, 1H), 6.85 (dd, J = 20.1, 11.7 Hz, 3H), 5.82 (td, J = 56.7, 3.9 Hz, 1H), 3.92 (t, J = 14.3 Hz, 2H), 3.84 (s, 3H), 3.27 (q, J = 11.6 Hz, 2H), 3.12 (tdd, J = 15.4, 10.4, 4.2 Hz, 1H), 1.86 – 1.71 (m, 2H), 1.66 (d, J = 12.4 Hz, 1H), 1.49 (d, J = 10.9 Hz, 1H), 1.40 – 1.23 (m, 3H); ^{13}C NMR (101 MHz, $CDCl_3$) δ 159.9, 138.5 (t, J = 4.0 Hz), 129.9, 121.3, 118.1 (t, J = 244.3 Hz), 115.1, 112.6, 67.9, 67.8, 55.3, 46.8 (t, J = 19.9 Hz), 35.2 (t, J = 4.0 Hz), 33.9, 32.2, 31.8; ^{19}F NMR (376 MHz, $CDCl_3$) δ -118.23 (ddd, J = 275.5, 56.5, 14.0 Hz, 1F), -122.27 (ddd, J = 275.5, 56.9, 16.4 Hz, 1F); HRMS (EI): m/z calcd $C_{15}H_{20}F_2O_2$ $[M]^+$ 270.1431, found 270.1438.

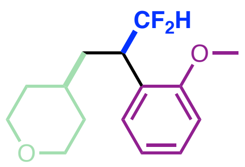

Using general procedure A. **4-(3,3-difluoro-2-(2-methoxyphenyl)propyl)tetrahydro-2H-pyran (16):** 0.25 mmol scale, 35 mg, 52%, colorless oil, 6% EA in hexane, R_f = 0.20.

^{1}H NMR (400 MHz, $CDCl_3$) δ 7.23 – 7.12 (m, 2H), 6.89 (t, J = 7.5 Hz, 1H), 6.83 (d, J = 8.2 Hz, 1H), 5.81 (td, J = 57.3, 3.7 Hz, 1H), 3.82 (t, J = 12.3 Hz, 2H), 3.76 (s, 3H), 3.70 – 3.54 (m, 1H), 3.17 (q, J = 11.8 Hz, 2H), 1.82 – 1.62 (m, 2H), 1.58 (d, J = 12.6 Hz, 1H), 1.40 (d, J = 9.6 Hz, 1H), 1.28 – 1.14 (m, 3H); ^{13}C NMR (101 MHz, $CDCl_3$) δ 157.7, 129.3, 128.6, 125.4 (t, J = 4.0 Hz), 121.0, 118.0 (t, J = 243.5 Hz), 110.9, 68.0, 68.0, 55.6, 39.5 (t, J = 19.9 Hz), 34.4 (t, J = 4.0 Hz), 33.9, 32.5, 32.1; ^{19}F NMR (376 MHz, $CDCl_3$) δ -117.08 (ddd, J = 274.0, 56.9, 11.9 Hz, 1F), -123.58 (ddd, J = 275.3, 56.8, 19.1 Hz, 1F); HRMS (ESI): m/z calcd $C_{15}H_{21}F_2O_2$ $[M+H]^+$ 271.1504, found 271.1508.

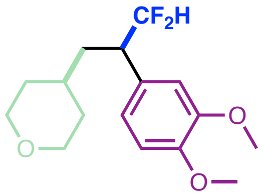

Using general procedure A. **4-(2-(3,4-dimethoxyphenyl)-3,3-difluoropropyl)tetrahydro-2H-pyran (17):** 0.25 mmol scale, 48 mg, 64%, colorless oil, 15% EA in hexane, $R_f = 0.10$.

^{1}H NMR (400 MHz, $CDCl_3$) δ 6.84 (d, J = 8.1 Hz, 1H), 6.81 – 6.64 (m, 2H), 5.78 (td, J = 56.9, 2.9 Hz, 1H), 3.87 (s, 8H), 3.24 (q, J = 11.7 Hz, 2H), 3.12 – 2.95 (m, 1H), 1.78 – 1.69 (m, 2H), 1.62 (d, J = 12.5 Hz, 1H), 1.45 (d, J = 9.8 Hz, 1H), 1.38 – 1.22 (m, 3H); ^{13}C NMR (101 MHz, $CDCl_3$) δ 149.2, 148.6, 129.2 (t, J = 4.0 Hz), 121.2, 118.2 (t, J = 244.3 Hz), 111.9, 111.4, 67.9, 67.8, 56.1, 55.9, 46.4 (t, J = 19.6 Hz), 35.2 (t, J = 4.0 Hz), 33.9, 32.2, 31.8; ^{19}F NMR (376 MHz, $CDCl_3$) δ -118.56 (ddd, J = 275.0, 56.6, 14.3 Hz, 1F), -122.45 (ddd, J = 275.1, 57.0, 16.7 Hz, 1F); HRMS (EI): m/z calcd $C_{16}H_{22}F_2O_3$ $[M]^+$ 300.1537, found 300.1538.

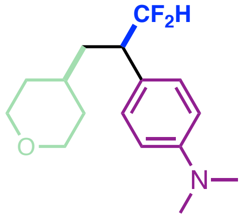

Using general procedure A. **4-(1,1-difluoro-3-(tetrahydro-2H-pyran-4-yl)propan-2-yl)-N,N-dimethylaniline (18):** 0.25 mmol scale, 39 mg, 55%, white solid, 5% EA in hexane, R_f = 0.11.

^{1}H NMR (400 MHz, $CDCl_3$) δ 7.10 (d, J = 8.4 Hz, 2H), 6.71 (d, J = 8.5 Hz, 2H), 5.76 (td, J = 57.1, 3.8 Hz, 1H), 3.89 (t, J = 13.8 Hz, 2H), 3.25 (q, J = 11.7 Hz, 2H), 3.10 – 2.94 (m, 7H), 1.82 – 1.61 (m, 3H), 1.47 (d, J = 11.0 Hz, 1H), 1.41 – 1.21(m, 3H); ^{13}C NMR (101 MHz, $CDCl_3$) δ 150.1, 129.5, 124.1 (t, J = 4.0 Hz), 118.6 (t, J = 245.0 Hz), 112.7, 68.0, 67.9, 45.8 (t, J = 19.4

Hz), 40.6, 35.1 (t, J = 4.0 Hz), 34.0, 32.2, 31.7; ^{19}F NMR (376 MHz, $CDCl_3$) δ -118.31 (ddd, J = 273.4, 57.0, 14.0 Hz, 1F), -122.69 (ddd, J = 273.4, 57.3, 17.3 Hz, 1F); HRMS (ESI): m/z calcd $C_{16}H_{23}F_2NNaO$ $[M+Na]^+$ 306.1640, found 306.1642.

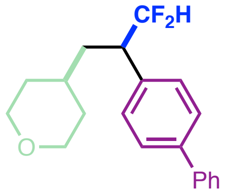

Using general procedure A. **4-(2-([1,1'-biphenyl]-4-yl)-3,3-difluoropropyl)tetrahydro-2H-pyran (19):** 0.25 mmol scale, 58 mg, 73%, colorless oil, 5% EA in hexane, R_f = 0.12.

^{1}H NMR (400 MHz, $CDCl_3$) δ 7.60 (t, J = 6.1 Hz, 4H), 7.45 (t, J = 7.2 Hz, 2H), 7.48 – 7.30 (m, 3H), 5.85 (t, J = 56.1 Hz, 1H), 3.91 (t, J = 13.5 Hz, 2H), 3.34 – 3.12 (m, 3H), 1.93 – 1.73 (m, 2H), 1.66 (d, J = 12.8 Hz, 1H), 1.50 (d, J = 10.7 Hz, 1H), 1.42 – 1.25 (m, 3H); ^{13}C NMR (101 MHz, $CDCl_3$) δ 140.7, 140.7, 135.8 (t, J = 4.0 Hz), 129.4, 128.9, 127.6, 127.6, 127.2, 118.2 (t, J = 244.0 Hz), 67.9, 67.8, 46.5 (t, J = 19.7 Hz), 35.3 (t, J = 4.0 Hz), 33.9, 32.2, 31.8; ^{19}F NMR (376 MHz, $CDCl_3$) δ -118.56 (ddd, J = 275.6, 56.6, 14.5 Hz, 1F), -122.04 (ddd, J = 275.5, 56.9, 16.3 Hz, 1F); HRMS (ESI): m/z calcd $C_{21}H_{25}F_2O$ $[M+H]^+$ 331.1868, found 331.1873.

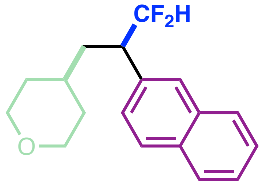

Using general procedure A. **4-(3,3-difluoro-2-(naphthalen-2-yl)propyl)tetrahydro-2H-pyran (20):** 0.25 mmol scale, 37 mg, 51%, colorless oil, 4% EA in hexane, R_f = 0.12.

^1H NMR (400 MHz, $CDCl_3$) δ 7.86 (t, J = 7.6 Hz, 3H), 7.72 (s, 1H), 7.56 – 7.45 (m, 2H), 7.39 (d, J = 8.4 Hz, 1H), 5.91 (td, J = 56.6, 2.9 Hz, 1H), 3.89 (dd, J = 19.6, 11.1 Hz, 2H), 3.39 – 3.14 (m, 3H), 1.96 (t, J = 11.7 Hz, 1H), 1.89 – 1.78 (m, 1H), 1.69 (d, J = 12.8 Hz, 1H), 1.49 (d, J = 9.1 Hz, 1H), 1.44 – 1.27 (m, 3H); ^{13}C NMR (101 MHz, $CDCl_3$) δ 134.3 (t, J = 4.0 Hz), 133.5, 133.0, 128.7, 128.2, 127.9, 127.8, 126.5, 126.2, 118.2 (t, J = 245.2 Hz) 67.9, 67.8, 47.0 (t, J = 19.8 Hz), 35.2 (t, J = 4.0 Hz), 33.9, 32.2, 31.8 (note: in aromatic region, one carbon is overlay by another one); ^{19}F NMR (376 MHz, $CDCl_3$) δ -118.27 (ddd, J = 275.9, 56.7, 14.5 Hz, 1F), -121.93 (ddd, J = 276.0, 56.8, 16.3 Hz, 1F); HRMS (EI): m/z calcd $C_{18}H_{20}F_2O$ $[M]^+$ 290.1482, found 290.1486.

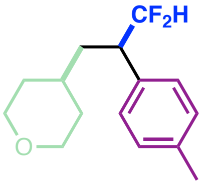

Using general procedure A. **4-(3,3-difluoro-2-(p-tolyl)propyl)tetrahydro-2H-pyran (21):** 0.25 mmol scale, 42 mg, 66%, colorless oil, 5% EA in hexane, R_f = 0.2.

^1H NMR (400 MHz, $CDCl_3$) δ 7.28 – 7.10 (m, 4H), 5.81 (td, J = 56.8, 3.8 Hz, 1H), 3.92 (t, J = 13.6 Hz, 2H), 3.33 – 3.01 (m, 3H), 2.38 (s, 3H), 1.88 – 1.70 (m, 2H), 1.65 (d, J = 12.8 Hz, 1H), 1.49 (d, J = 9.5 Hz, 1H), 1.40 – 1.21 (m, 3H); ^{13}C NMR (101 MHz, $CDCl_3$) δ 137.5, 133.7 (t, J = 4.0 Hz) , 129.6, 128.8, 118.3 (t, J = 244.9 Hz), 67.9, 67.8, 46.4 (t, J = 19.6 Hz), 35.2 (t, J = 4.0 Hz), 33.9, 32.2, 31.8, 21.2; ^{19}F NMR (376 MHz, $CDCl_3$) δ -118.51 (ddd, J = 275.2, 56.8, 14.4 Hz, 1F), -122.31 (ddd, J = 275.1, 57.0, 16.5 Hz, 1F); HRMS (EI): m/z calcd $C_{15}H_{19}FO$ $[M-HF]^+$ 234.1420, found 234.1426.

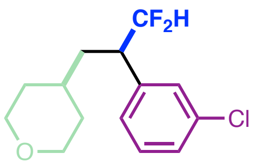

Using general procedure A. **4-(2-(3-chlorophenyl)-3,3-difluoropropyl)tetrahydro-2H-pyran (22):** 0.25 mmol scale, 30.2 mg, 44%, colorless oil, 5% EA in hexane, R_f = 0.10.

^{1}H NMR (400 MHz, $CDCl_3$) δ 7.30 (d, J = 4.7 Hz, 2H), 7.24 (s, 1H), 7.17 – 7.08 (m, 1H), 5.79 (td, J = 56.5, 3.5 Hz, 1H), 3.90 (t, J = 11.6 Hz, 2H), 3.32 – 3.01 (m, 3H), 1.85 – 1.65 (m, 2H), 1.64 – 1.58 (m, 1H), 1.48 (d, J = 8.7 Hz, 1H), 1.36 – 1.24 (m, 3H); ^{13}C NMR (101 MHz, $CDCl_3$) δ 138.8, 134.8, 130.2, 129.1, 128.1, 127.3, 117.7 (t, J = 243.6 Hz), 67.9, 67.8, 46.5 (t, J = 19.3 Hz), 35.2 (t, J = 3.0 Hz), 33.8, 32.2, 31.8; ^{19}F NMR (376 MHz, $CDCl_3$) δ -119.12 (ddd, J = 276.9, 56.3, 15.0 Hz, 1F), -121.82 (ddd, J = 276.9, 56.6, 15.5 Hz, 1F); MS (EI): m/z calcd $C_{14}H_{17}ClF_2O$ $[M]^+$ 274.1, found 274.1; HRMS (EI): m/z calcd $C_{14}H_{16}ClFO$ $[M-HF]^+$ 254.0874, found 254.0887.

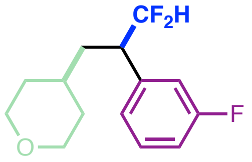

Using general procedure A. **4-(3,3-difluoro-2-(3-fluorophenyl)propyl)tetrahydro-2H-pyran (23):** 0.25 mmol scale, 37 mg, 57%, colorless oil, Hexane, R_f = 0.15.

^{1}H NMR (400 MHz, $CDCl_3$) δ 7.32 (q, J = 7.1 Hz, 1H), 7.05 – 6.92 (m, 3H), 5.80 (td, J = 56.5, 2.9 Hz, 1H), 3.90 (t, J = 11.8 Hz, 2H), 3.32 – 3.06 (m, 3H), 1.84 – 1.69 (m, 2H), 1.61 (d, J = 12.9 Hz, 1H), 1.47 (d, J = 9.0 Hz, 1H), 1.36 – 1.24 (m, 3H); ^{13}C NMR (101 MHz, $CDCl_3$) δ 163.1 (d, J = 246.9 Hz), 139.3 (t, J = 3.0 Hz), 130.4 (d, J = 8.4 Hz), 124.8 (d, J = 2.0 Hz), 117.7 (t, J = 244.6Hz), 115.9 (d, J = 21.7 Hz), 114.9 (d, J = 21.0 Hz), 67.9, 67.8, 46.6 (t, J = 20.2 Hz), 35.3 (t, J = 4.0 Hz), 33.8, 32.2, 31.8; ^{19}F NMR (376 MHz, $CDCl_3$) δ -112.50 (td, J = 9.2, 6.0 Hz, 1F), -119.09 (ddd, J = 276.9, 56.3, 14.8 Hz, 1F), -121.99 (ddd, J = 276.8, 56.6, 15.6 Hz,

1F); MS (EI): m/z calcd $C_{14}H_{17}F_3O$ $[M]^+$ 258.1, found 258.1; HRMS (EI): m/z calcd $C_{14}H_{16}F_2O$ $[M-HF]^+$ 238.1169, found 238.1184.

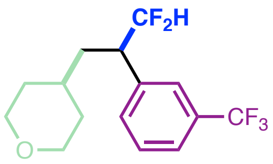

Using general procedure A.

4-(3,3-difluoro-2-(3-(trifluoromethyl)phenyl)propyl)tetrahydro-2H-pyran (24): 0.25 mmol scale, 40 mg, 52%, colorless oil, 5% EA in Hexane, R_f = 0.13.

^{1}H NMR (400 MHz, $CDCl_3$) δ 7.59 (d, J = 7.7 Hz, 1H), 7.54 – 7.34 (m, 3H), 5.83 (td, J = 56.4, 3.5 Hz, 1H), 3.90 (t, J = 11.8 Hz, 2H), 3.30 – 3.15 (m, 3H), 1.87 – 1.74 (m, 2H), 1.61 (d, J = 13.5 Hz, 1H), 1.48 (d, J = 8.9 Hz, 1H), 1.39 – 1.25 (m, 3H); ^{13}C NMR (101 MHz, $CDCl_3$) δ 137.8, 132.5, 131.3 (q, J = 32.4 Hz), 129.4, 125.7 (q, J = 3.0 Hz), 124.9 (q, J = 4.0 Hz), 124.1 (q, J = 272.8 Hz) 117.5 (t, J = 245.2 Hz), 67.9, 67.7, 46.7 (t, J = 19.9 Hz), 35.3 (t, J = 4.0 Hz), 33.8, 32.2, 31.9; ^{19}F NMR (376 MHz, $CDCl_3$) δ -62.60 (s, 3F), -119.63 (ddd, J = 277.6, 56.3, 15.5 Hz, 1F), -121.64 (ddd, J = 277.9, 56.5, 15.2 Hz, 1F); MS (EI): m/z calcd $C_{15}H_{17}F_5O$ $[M]^+$ 308.1, found 308.1; HRMS (EI): m/z calcd $C_{15}H_{16}F_4O$ $[M-HF]^+$ 288.1137, found 288.1146.

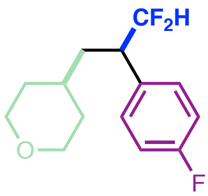

Using general procedure A. **4-(3,3-difluoro-2-(4-fluorophenyl)propyl)tetrahydro-2H-pyran (25):** 0.25 mmol scale, 32 mg, 50%, colorless oil, 4% EA in hexane, R_f = 0.15.

^{1}H NMR (400 MHz, $CDCl_3$) δ 7.20 – 7.09 (m, 2H), 6.98 (t, J = 8.1 Hz, 2H), 5.93 – 5.50 (m, 1H), 3.82 (t, J = 11.3 Hz, 2H), 3.28 – 2.94 (m, 3H), 1.78 – 1.61 (m, 2H), 1.52 (d, J = 11.3 Hz,

1H), 1.37 (t, J = 9.1 Hz, 1H), 1.29 – 1.17 (m, 3H); ^{13}C NMR (101 MHz, $CDCl_3$) δ 162.4 (d, J = 247.5 Hz), 132.4 (d, J = 4.6 Hz), 130.5 (d, J = 7.8 Hz), 117.9 (t, J = 244.2 Hz), 115. (d, J = 22.1 Hz), 67.90, 67.8, 46.1 (t, J = 20.4 Hz), 35.3 (t, J = 4.0 Hz), 33.9, 32.2, 31.8; ^{19}F NMR (376 MHz, $CDCl_3$) δ -114.71 (tt, J = 8.6, 5.3 Hz, 1F), -119.38 (ddd, J = 276.2, 56.4, 15.1 Hz, 1F), -122.09 (ddd, J = 276.1, 56.8, 15.9 Hz, 1F); HRMS (EI): m/z calcd $C_{14}H_{16}F_2O$ $[M-HF]^+$ 238.1174, found 238.1174.

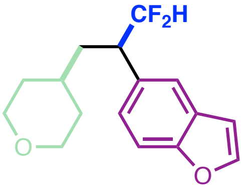

Using general procedure A. **5-(1,1-difluoro-3-(tetrahydro-2H-pyran-4-yl)propan-2-yl)benzofuran (26)**: 0.25 mmol scale, 44 mg, 63%, colorless oil, 7% EA in hexane, R_f = 0.10.

^{1}H NMR (400 MHz, $CDCl_3$) δ 7.64 (s, 1H), 7.49 (d, J = 8.6 Hz, 2H), 7.17 (d, J = 8.5 Hz, 1H), 6.76 (s, 1H), 5.84 (td, J = 56.8, 3.6 Hz, 1H), 3.99 – 3.77 (m, 2H), 3.22 (q, J = 11.4 Hz, 3H), 1.91 – 1.72 (m, 2H), 1.65 (d, J = 12.0 Hz, 1H), 1.47 (d, J = 8.6 Hz, 1H), 1.36 – 1.20 (m, 3H); ^{13}C NMR (101 MHz, $CDCl_3$) δ 154.6, 145.7, 131.2 (t, J = 4.0 Hz), 128.0, 125.0, 121.5, 118.3 (t, J = 243.3 Hz), 111.7, 106.6, 67.9, 67.8, 46.7 (t, J = 19.4 Hz), 35.6 (t, J = 3.4 Hz), 33.9, 32.13, 31.7; ^{19}F NMR (376 MHz, $CDCl_3$) δ -118.55 (ddd, J = 275.1, 56.7, 14.5 Hz, 1F), -122.21 (ddd, J = 275.2, 57.0, 16.4 Hz, 1F); HRMS (EI): m/z calcd $C_{16}H_{18}F_2O_2$ $[M]^+$ 280.1275, found 280.1273.

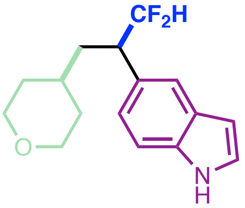

Using general procedure A. **5-(1,1-difluoro-3-(tetrahydro-2H-pyran-4-yl)propan-2-yl)-1H-indole (27):** 0.25 mmol scale, 40 mg, 57%, White solid, 15% EA in Hexane, R_f = 0.10.

^{1}H NMR (400 MHz, $CDCl_3$) δ 8.30 (s, 1H), 7.52 (s, 1H), 7.37 (d, J = 8.3 Hz, 1H), 7.22 (s, 1H), 7.07 (d, J = 8.3 Hz, 1H), 6.55 (s, 1H), 5.86 (td, J = 57.0, 3.8 Hz, 1H), 3.96 – 3.84 (m, 2H), 3.34 – 3.09 (m, 3H), 1.97 – 1.84 (m, 1H), 1.78 (ddd, J = 13.4, 8.3, 4.1 Hz, 1H), 1.69 (d, J = 12.0 Hz, 1H), 1.50 – 1.27 (m, 4H); ^{13}C NMR (101 MHz, $CDCl_3$) δ 135.4, 128.3, 128.0 (t, J = 4.0 Hz), 125.0, 122.8, 121.0, 118.8 (t, J = 244.7 Hz), 111.4, 102.6, 67.9, 67.9, 46.9 (t, J = 19.6 Hz), 35.5 (t, J = 4.0 Hz), 34.0, 32.2, 31.7; ^{19}F NMR (376 MHz, $CDCl_3$) δ -117.75 (ddd, J = 273.5, 56.8, 13.7 Hz, 1F), -122.54 (ddd, J = 273.6, 57.3, 17.3 Hz, 1F); HRMS (ESI): m/z calcd $C_{16}H_{19}F_2NNaO$ $[M+Na]^+$ 302.1327, found 302.1328.

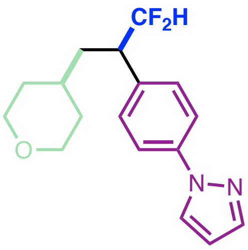

Using general procedure A. **1-(4-(1,1-difluoro-3-(tetrahydro-2H-pyran-4-yl)propan-2-yl)phenyl)-1H-pyrazole (28):** 0.25 mmol scale, 43 mg, 56%, white solid, 10% EA in hexane, R_f = 0.13.

H NMR (400 MHz, $CDCl_3$) δ 7.92 (d, J = 1.9 Hz, 1H), 7.80 – 7.57 (m, 3H), 7.33 (d, J = 8.3 Hz, 2H), 6.47 (s, 1H), 5.82 (td, J = 56.6, 3.7 Hz, 1H), 4.00 – 3.76 (m, 2H), 3.34 – 3.07 (m, 3H), 1.88 – 1.74 (m, 2H), 1.61 (d, J = 12.1 Hz, 1H), 1.46 (d, J = 8.0 Hz, 1H), 1.35 – 1.24 (m, 3H); ^{13}C NMR (101 MHz, $CDCl_3$) δ 141.3, 139.8, 134.9 (t, J = 4.0 Hz), 130.0, 126.8, 119.6, 117.9 (t, J = 244.1 Hz), 107.9, 67.9, 67.8, 46.3 (t, J = 19.7 Hz), 35.2 (t, J = 4.0 Hz), 33.9, 32.1, 31.8; ^{19}F NMR (376 MHz, $CDCl_3$) δ -119.18 (ddd, J = 276.5, 56.7, 15.1 Hz, 1F), -121.84 (ddd, J =

276.2, 56.6, 15.6 Hz, 1F); HRMS (ESI): m/z calcd $C_{17}H_{21}F_2N_2O$ $[M+H]^+$ 307.1616, found 307.1620.

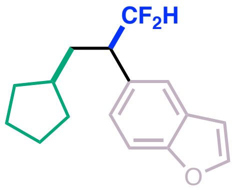

Using general procedure A. **5-(3-cyclopentyl-1,1-difluoropropan-2-yl)benzofuran (29):** 0.25 mmol scale, 43 mg, 65%, colorless oil, Hexane, $R_f = 0.20$.

^{1}H NMR (400 MHz, $CDCl_3$) δ 7.63 (s, 1H), 7.49 (d, J = 8.4 Hz, 2H), 7.19 (d, J = 8.5 Hz, 1H), 6.76 (s, 1H), 5.86 (td, J = 56.8, 3.3 Hz, 1H), 3.25 – 3.03 (m, 1H), 1.98 (td, J = 13.4, 12.2, 4.1 Hz, 1H), 1.85 – 1.71 (m, 2H), 1.68 – 1.53 (m, 4H), 1.52 – 1.40 (m, 2H), 1.20 – 1.03 (m, 2H); ^{13}C NMR (101 MHz, $CDCl_3$) δ 154.5, 145.6, 131.8 (t, J = 4.0 Hz), 127.9, 125.2, 121.6, 118.4 (t, J = 244.4 Hz), 111.5, 106.7, 49.3 (t, J = 19.6 Hz), 37.0, 35.1 (t, J = 4.0 Hz), 33.5, 31.9, 25.2, 25.2; ^{19}F NMR (376 MHz, $CDCl_3$) δ -118.72 (ddd, J = 274.9, 56.8, 15.2 Hz, 1F), -121.82 (ddd, J = 274.8, 57.0, 16.0 Hz, 1F); HRMS (EI): m/z calcd $C_{16}H_{18}F_2O$ $[M]^+$ 264.1326, found 264.1328.

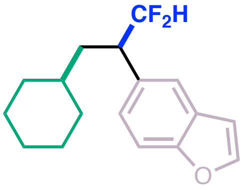

Using general procedure A. **5-(3-cyclohexyl-1,1-difluoropropan-2-yl)benzofuran (30):** 0.25 mmol scale, 36 mg, 52%, colorless oil, Hexane, $R_f = 0.20$.

^1H NMR (400 MHz, $CDCl_3$) δ 7.63 (s, 1H), 7.49 (d, J = 4.4 Hz, 2H), 7.18 (d, J = 8.6 Hz, 1H), 6.77 (s, 1H), 5.84 (td, J = 56.9, 3.8 Hz, 1H), 3.24 (tdt, J = 15.2, 9.8, 4.1 Hz, 1H), 1.84 – 1.56 (m, 7H), 1.16 – 0.83 (m, 6H); ^{13}C NMR (101 MHz, $CDCl_3$) δ 154.5, 145.5, 131.7 (t, J = 4.0 Hz), 127.9, 125.2, 121.6, 118.6 (t, J = 242.6 Hz), 111.6, 106.7, 47.0 (t, J = 19.4 Hz), 36.2 (t, J = 4.0 Hz), 34.5, 34.1, 32.2, 26.61, 26.3, 26.1; ^{19}F NMR (376 MHz, $CDCl_3$) δ -118.93 (ddd, J = 274.1, 56.8, 15.3 Hz, 1F), -121.72 (ddd, J = 274.5, 57.0, 15.9 Hz, 1F); HRMS (EI): m/z calcd $C_{17}H_{20}F_2O$ $[M]^+$ 278.1482, found 278.1487.

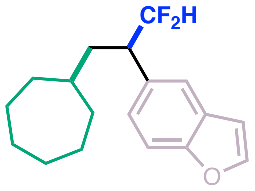

Using general procedure A. **5-(3-cycloheptyl-1,1-difluoropropan-2-yl)benzofuran (31):** 0.25 mmol scale, 45 mg, 62%, colorless oil, Hexane, R_f = 0.22.

^1H NMR (400 MHz, $CDCl_3$) δ 7.64 (s, 1H), 7.49 (d, J = 4.3 Hz, 2H), 7.18 (d, J = 8.6 Hz, 1H), 6.77 (s, 1H), 5.85 (td, J = 56.9, 3.8 Hz, 1H), 3.20 (dtq, J = 15.1, 9.5, 4.5 Hz, 1H), 1.85 – 1.70 (m, 3H), 1.64 – 1.41 (m, 7H), 1.36 – 1.12 (m, 5H); ^{13}C NMR (101 MHz, $CDCl_3$) δ 154.5, 145.5, 131.7 (t, J = 4.0 Hz), 127.9, 125.2, 121.6, 118.6 (t, J = 244.5 Hz), 111.6, 106.7, 47.7 (t, J = 19.3 Hz), 36.6 (t, J = 4.0 Hz), 35.9, 35.5, 32.9, 28.8, 28.6, 26.3, 26.0; ^{19}F NMR (376 MHz, $CDCl_3$) δ -118.73 (ddd, J = 274.4, 56.8, 15.3 Hz, 1F), -121.62 (ddd, J = 274.3, 57.0, 15.8 Hz, 1F); HRMS (EI): m/z calcd $C_{18}H_{22}F_2O$ $[M]^+$ 292.1639, found 292.1644.

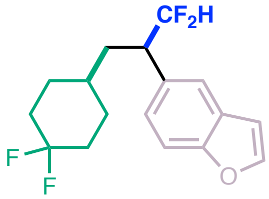

Using general procedure A. **5-(3-(4,4-difluorocyclohexyl)-1,1-difluoropropan-2-yl)benzofuran (32):** 0.25 mmol scale, 58 mg, 74%, colorless oil, 4% EA in Hexane, R_f = 0.18.

^{1}H NMR (400 MHz, $CDCl_3$) δ 7.65 (s, 1H), 7.50 (d, J = 9.2 Hz, 2H), 7.17 (d, J = 8.4 Hz, 1H), 6.77 (s, 1H), 5.84 (td, J = 56.7, 3.7 Hz, 1H), 3.34 – 3.09 (m, 1H), 2.01 (td, J = 18.6, 16.1, 8.2 Hz, 2H), 1.93 – 1.75 (m, 3H), 1.66 – 1.47 (m, 3H), 1.39 – 1.20 (m, 3H); ^{13}C NMR (101 MHz, $CDCl_3$) δ 154.6, 145.8, 131.1 (t, J = 4.0 Hz), 128.1, 125.0, 123.6 (t, J = 241.4 Hz)121.5, 118.3 (t, J = 244.5 Hz), 111.8, 106.7, 47.5 (t, J = 19.6 Hz), 34.4, 33.4 (dd, J = 20.1, 5.3 Hz), 33.2 (dd, J = 20.1, 5.3 Hz), 32.5, 30.1 (d, J = 9.4 Hz), 27.8 (d, J = 9.5 Hz); ^{19}F NMR (376 MHz, $CDCl_3$) δ -91.79 (d, J = 235.6 Hz, 1F), -102.10 (d, J = 231.8 Hz, 1F), -118.43 (ddd, J = 275.1, 56.5, 14.1 Hz, 1F), -122.40 (ddd, J = 275.3, 56.9, 16.6 Hz, 1F); HRMS (EI): m/z calcd $C_{17}H_{18}F_4O$ $[M]^+$ 314.1294, found 314.1297.

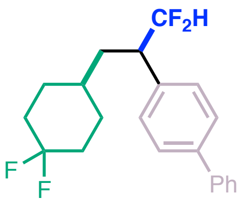

Using general procedure A. **4-(3-(4,4-difluorocyclohexyl)-1,1-difluoropropan-2-yl)-1,1'-biphenyl (33):** 0.25 mmol scale, 47 mg, 54%, colorless oil, 2% EA in Hexane, R_f = 0.16.

^{1}H NMR (400 MHz, $CDCl_3$) δ 7.66 – 7.57 (m, 4H), 7.46 (t, J = 7.5 Hz, 2H), 7.50 – 7.28 (m, 3H), 5.85 (td, J = 56.7, 3.5 Hz, 1H), 3.15 (qd, J = 15.3, 3.7 Hz, 1H), 2.10 – 1.97 (m, 2H), 1.93 – 1.76 (m, 3H), 1.73 – 1.56 (m, 3H), 1.43 – 1.27 (m, 3H); ^{13}C NMR (101 MHz, $CDCl_3$) δ 140.8, 140.6, 135.7 (t, J = 4.0 Hz), 129.3, 129.0, 127.6, 127.6, 127.2, 123.6 (t, J = 241.0 Hz), 118.1 (t, J = 244.2 Hz), 47.3 (t, J = 19.7 Hz), 34.1, 33.5 (dd, J = 19.8, 5.7 Hz), 33.2 (dd, J = 19.8, 5.3 Hz), 32.5, 30.1, 27.9; ^{19}F NMR (376 MHz, $CDCl_3$) δ -91.84 (d, J = 234.3 Hz, 1F), -102.07 (d,

J = 243.3 Hz, 1F), -118.44 (ddd, J = 275.8, 56.5, 14.2 Hz, 1F), -122.24 (ddd, J = 275.9, 56.8, 16.5 Hz, 1F); HRMS (EI): m/z calcd $C_{21}H_{22}F_4$ $[M]^+$ 350.1658, found 350.1666.

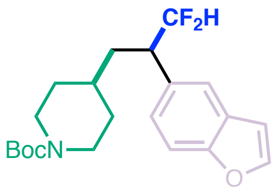

tert-butyl-4-(2-(benzofuran-5-yl)-3,3-difluoropropyl)piperidine-1-carboxylate (34): 0.25 mmol scale, 52 mg, 55%, white solid, 9% EA in Hexane, R_f = 0.15.

In a nitrogen-filled glovebox, to a 4 mL vial equipped with a stir bar was added terpyridine (1.1 equiv., 64.6 mg), $(DMPU)_2Zn(CF_2H)_2$ (0.8 equiv., 87 mg), and 800 μL DMSO. The resulting mixture was stirred at room temperature for 1 min to generate $(terpy)Zn(CF_2H)_2$ in-situ. A different 4 mL vial equipped with a stir bar was sequentially charged with CuCl (30 mol %, 7.5 mg), RAEs (0.25 mmol, 1.0 equiv.), the DMSO solution of the in-situ formed $(terpy)Zn(CF_2H)_2$, and alkene (0.75 mmol, 3.0 equiv.) in DMSO (200 μL). The resultant mixture was stirred at room temperature for 12 h. After the reaction was completed, the mixture was diluted with EtOAc (50 mL), filtered through a short plug of Celite, and washed with H_2O (50 mL) and brine. The organic layer was combined, dried over Na_2SO_4, filtered, and then concentrated under reduced pressure. The crude difluoromethylation product was purified by flash column chromatography.

^1H NMR (400 MHz, $CDCl_3$) δ 7.63 (d, J = 1.7 Hz, 1H), 7.48 (d, J = 8.4 Hz, 2H), 7.16 (d, J = 8.6 Hz, 1H), 6.76 (s, 1H), 5.82 (td, J = 56.8, 3.7 Hz, 1H), 4.01 (s, 2H), 3.34 – 3.09 (m, 1H), 2.52 (q, J = 14.9, 14.2 Hz, 2H), 1.90 – 1.68 (m, 3H), 1.51 (d, J = 12.4 Hz, 1H), 1.43 (s, 9H), 1.27 – 1.05 (m, 3H); ^{13}C NMR (101 MHz, $CDCl_3$) δ 154.9, 154.5, 145.7, 131.2 (t, J = 4.0 Hz), 128.0, 125.0, 121.5, 118.3 (t, J = 245.4 Hz), 111.7, 106.6, 79.4, 46.9 (t, J = 19.8 Hz), 43.9, 43.6,

35.2 (t, J = 3.0 Hz), 33.1, 32.7, 31.1, 28.6; ^{19}F NMR (376 MHz, $CDCl_3$) δ -118.55 (dd, J = 274.2, 53.9 Hz, 1F), -122.29 (ddd, J = 274.8, 56.8, 15.8 Hz, 1F); HRMS (ESI): m/z calcd $C_{21}H_{27}F_2NNaO_3$ $[M+Na]^+$ 402.1851, found 402.1850.

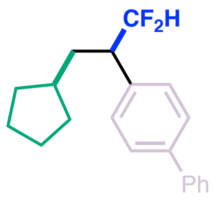

Using general procedure A. **4-(3-cyclopentyl-1,1-difluoropropan-2-yl)-1,1'-biphenyl (35):** 0.25 mmol scale, 40 mg, 53%, white solid, Hexane, R_f = 0.12.

^{1}H NMR (400 MHz, $CDCl_3$) δ 7.60 (t, J = 8.1 Hz, 4H), 7.46 (t, J = 7.5 Hz, 2H), 7.40 – 7.30 (m, 3H), 5.87 (td, J = 56.8, 3.9 Hz, 1H), 3.11 (dh, J = 15.1, 3.8 Hz, 1H), 1.98 (ddd, J = 13.4, 11.0, 4.4 Hz, 1H), 1.87 – 1.73 (m, 2H), 1.73 – 1.58 (m, 4H), 1.52 – 1.42 (m, 2H), 1.22 – 1.08 (m, 2H); ^{13}C NMR (101 MHz, $CDCl_3$) δ 140.8, 140.5, 136.3 (t, J = 4.0 Hz), 129.5, 128.9, 127.4, 127.2, 118.2 (t, J = 243.8 Hz), 49.0 (t, J = 19.8 Hz), 37.0, 34.8 (t, J = 4.0 Hz), 33.5, 31.9, 25.3, 25.2 (note: in aromatic region, one carbon is overlay by another one); ^{19}F NMR (376 MHz, $CDCl_3$) δ -118.76 (ddd, J = 275.5, 56.7, 15.3 Hz, 1F), -121.62 (ddd, J = 275.6, 56.8, 15.8 Hz, 1F); HRMS (EI): m/z calcd $C_{20}H_{22}F_2$ $[M]^+$ 300.1690, found 300.1695.

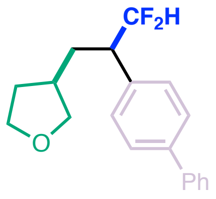

Using general procedure A.

3-((S)-2-([1,1'-biphenyl]-4-yl)-3,3-difluoropropyl)tetrahydrofuran (36): 0.25 mmol scale, 40 mg, 53%, 1.2:1 dr, white solid, 5% EA in Hexane, R_f = 0.20.

^{1}H NMR (400 MHz, $CDCl_3$) δ 7.58 (d, J = 7.7 Hz, 4H), 7.44 (t, J = 7.2 Hz, 2H), 7.33 (dd, J = 19.0, 7.4 Hz, 3H), 5.87 (td, J = 56.6, 3.9 Hz, 1H), 3.96 – 3.65 (m, 3H), 3.33 (dt, J = 58.2, 7.5 Hz, 1H), 3.18 – 2.90 (m, 1H), 2.15 – 1.86 (m, 4H), 1.59 – 1.43 (m, 1H); ^{13}C NMR (101 MHz, $CDCl_3$) δ 140.9, 140.6, 135.6, 129.3, 128.9, 127.7, 127.6, 127.2, 117.9 (t, J = 245.0 Hz), 73.5, 68.0, 49.1 (t, J = 19.9 Hz), 36.8, 32.1, 32.0 (t, J = 4.0 Hz); ^{19}F NMR (376 MHz, $CDCl_3$) δ -118.20 (dddd, J = 276.8, 69.8, 56.3, 13.9 Hz, 1F), -122.43 (dddd, J = 276.8, 56.8, 47.3, 16.7 Hz, 1F); HRMS (EI): m/z calcd $C_{19}H_{20}F_2O$ $[M]^+$ 302.1482, found 302.1477.

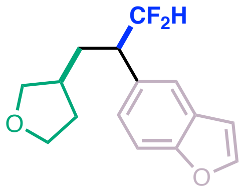

Using general procedure A or C. **5-(1,1-difluoro-3-(tetrahydrofuran-3-yl)propan-2-yl)benzofuran (37):** 0.25 mmol scale, 38 mg, 57%, 1.2 : 1 dr, colorless oil, 5% EA in Hexane, R_f = 0.16.

^{1}H NMR (400 MHz, $CDCl_3$) δ 7.64 (s, 1H), 7.49 (d, J = 7.5 Hz, 2H), 7.17 (d, J = 8.5 Hz, 1H), 6.76 (s, 1H), 5.87 (tt, J = 56.7, 4.0 Hz, 1H), 3.90 – 3.59 (m, 3H), 3.45 – 3.19 (m, 1H), 3.19 – 2.94 (m, 1H), 2.08 – 1.81 (m, 4H), 1.63 – 1.39 (m, 1H); ^{13}C NMR (101 MHz, $CDCl_3$) δ 154.6, 145.8, 131.1 (t, J = 4.0 Hz), 128.1, 125.0, 121.5, 118.1 (t, J = 244.3 Hz), 111.8, 106.6, 73.5, 68.0, 49.32 (t, J = 19.7 Hz), 36.8, 33.0, 32.4 (t, J = 4.0 Hz); ^{19}F NMR (376 MHz, $CDCl_3$) δ -122.90 (dddd, J = 276.4, 62.7, 56.5, 14.0 Hz, 1F), -127.39 (dddd, J = 276.3, 56.9, 49.4, 17.0 Hz, 1F); HRMS (EI): m/z calcd $C_{15}H_{16}F_2O_2$ $[M]^+$ 266.1118, found 266.1113.

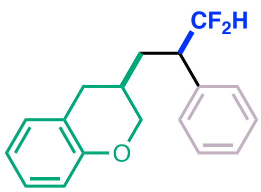

Using general procedure A. **3-(3,3-difluoro-2-phenylpropyl)chromane (38):** 0.25 mmol scale, 45 mg, 62%, 1.4:1 dr, colorless oil, 2% EA in Hexane, R_f = 0.23.

(Mixture of diastereoisomers) ^{1}H NMR (400 MHz, $CDCl_3$) δ 7.42 – 7.27 (m, 5H), 7.13 – 6.97 (m, 2H), 6.80 (ddt, J = 29.9, 16.2, 7.8 Hz, 2H), 5.86 (t, J = 56.7 Hz, 1H), 4.08 (d, J = 10.6 Hz, 1H), 3.88 – 3.68 (m, 1H), 3.34 – 3.08 (m, 1H), 2.93 – 2.71 (m, 1H), 2.58 – 2.41 (m, 1H), 2.03 – 1.73 (m, 3H); (Mixture of diastereoisomers) ^{13}C NMR (101 MHz, $CDCl_3$) δ 154.6, 136.4 (t, J = 4.0 Hz), 130.0, 129.1, 128.9, 128.11, 127.5, 121.3, 120.5, 117.9 (t, J = 243.4 Hz), 116.6, 70.8, 47.6 (t, J = 19.6 Hz), 32.2, 30.7, 30.1 (t, J = 4.0 Hz), 29.4; (Mixture of diastereoisomers) ^{19}F NMR (376 MHz, $CDCl_3$) δ -118.35 (dddd, J = 276.4, 64.0, 56.5, 13.9 Hz, 1F), -122.64 (dddd, J = 276.5, 56.8, 23.8, 16.6 Hz, 1F); HRMS (EI): m/z calcd $C_{18}H_{18}F_2O$ $[M]^+$ 288.1326, found 288.1331.

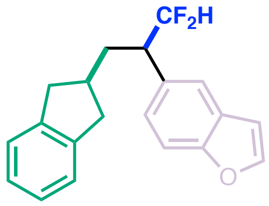

Using general procedure A. **5-(3-(2,3-dihydro-1H-inden-2-yl)-1,1-difluoropropan-2-yl)benzofuran (39):** 0.25 mmol scale, 43 mg, 55%, white solid, 2% EA in Hexane, R_f = 0.15.

^{1}H NMR (400 MHz, $CDCl_3$) δ 7.64 (s, 1H), 7.60 – 7.44 (m, 2H), 7.24 (d, J = 8.5 Hz, 1H), 7.20 – 7.08 (m, 4H), 6.78 (s, 1H), 5.91 (td, J = 56.8, 3.7 Hz, 1H), 3.24 (dtt, J = 19.4, 9.5, 4.2 Hz, 1H), 3.02 (dd, J = 15.3, 7.7 Hz, 1H), 2.91 (dd, J = 15.5, 7.8 Hz, 1H), 2.62 (ddd, J = 29.1, 15.4, 8.5 Hz, 2H), 2.30 (dp, J = 16.6, 8.3 Hz, 1H), 2.22 – 2.04 (m, 2H); ^{13}C NMR (101 MHz, $CDCl_3$)

δ 154.6, 145.7, 143.3, 142.9, 131.3 (t, J = 4.0 Hz), 128.0, 126.3, 126.3, 125.1, 124.5, 124.4, 121.6, 118.3 (t, J = 244.5 Hz), 111.7, 106.6, 49.1 (t, J = 19.6 Hz), 39.8, 38.5, 37.4, 34.5 (t, J = 4.0 Hz); ^{19}F NMR (376 MHz, $CDCl_3$) δ -118.38 (ddd, J = 275.7, 56.7, 14.8 Hz, 1F), -122.03 (ddd, J = 275.5, 56.9, 16.3 Hz, 1F); HRMS (EI): m/z calcd $C_{20}H_{18}F_2O$ $[M]^+$ 312.1324, found 312.1333.

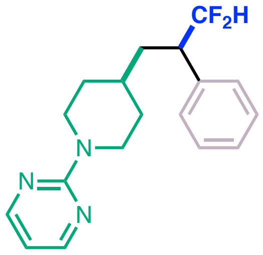

Using general procedure A. **2-(4-(3,3-difluoro-2-phenylpropyl)piperidin-1-yl)pyrimidine (40):** 0.25 mmol scale, 56 mg, 71%, white solid, 7% EA in Hexane, R_f = 0.14.

^{1}H NMR (400 MHz, $CDCl_3$) δ 8.29 (d, J = 4.6 Hz, 2H), 7.45 – 7.25 (m, 5H), 6.44 (t, J = 4.6 Hz, 1H), 5.84 (td, J = 56.7, 3.8 Hz, 1H), 4.71 (t, J = 15.1 Hz, 2H), 3.37 – 3.02 (m, 1H), 2.75 (q, J = 13.1 Hz, 2H), 1.91 – 1.73 (m, 3H), 1.66 (d, J = 12.8 Hz, 1H), 1.53 – 1.35 (m, 1H), 1.21 (dqd, J = 24.7, 12.3, 4.1 Hz, 2H); ^{13}C NMR (101 MHz, $CDCl_3$) δ 161.6, 157.8, 136.9 (t, J = 4.0 Hz), 129.0, 128.9, 127.8, 118.19 (t, J = 244.9 Hz), 109.4, 47.1 (t, J = 19.6 Hz), 44.0, 43.9, 35.0 (t, J = 4.0 Hz), 33.1, 33.0, 31.2; ^{19}F NMR (376 MHz, $CDCl_3$) δ -118.51 (ddd, J = 275.5, 56.7, 14.4 Hz, 1F), -122.30 (ddd, J = 275.6, 56.8, 16.3 Hz, 1F); HRMS (ESI): m/z calcd $C_{18}H_{22}F_2N_3$ $[M+H]^+$ 318.1776, found 318.1780.

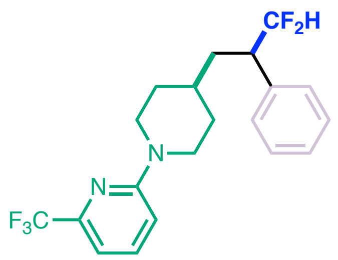

Using general procedure A. **2-(4-(3,3-difluoro-2-phenylpropyl)piperidin-1-yl)-6-(trifluoromethyl)pyridine (41):** 0.25 mmol scale, 62 mg, 65%, white solid, 2% EA in Hexane, R_f = 0.15.

^{1}H NMR (400 MHz, $CDCl_3$) δ 8.35 (s, 1H), 7.57 (dd, J = 9.1, 2.2 Hz, 1H), 7.45 – 7.25(m, 3H), 7.26 (d, J = 6.8 Hz, 2H), 6.59 (d, J = 9.1 Hz, 1H), 5.81 (td, J = 56.7, 3.9 Hz, 1H), 4.47 – 4.21 (m, 2H), 3.25 – 3.05 (m, 1H), 2.76 (qd, J = 13.2, 2.6 Hz, 2H), 1.93 – 1.70 (m, 3H), 1.65 (d, J = 12.9 Hz, 1H), 1.49 – 1.35 (m, 1H), 1.22 (dtt, J = 23.9, 11.9, 6.1 Hz, 2H); ^{13}C NMR (101 MHz, $CDCl_3$) δ 160.3, 145.9, 136.8 (t, J = 4.0 Hz), 134.5 (q, J = 3.2 Hz), 129.3, 128.9, 128.8, 127.9, 124.9 (q, J = 270.0 Hz) 118.2 (t, J = 245.2 Hz), 105.6, 47.2 (t, J = 19.7 Hz), 45.2, 45.0, 34.9 (t, J = 4.0 Hz), 33.1, 32.7, 30.9; ^{19}F NMR (376 MHz, $CDCl_3$) δ -61.06 (s, 3F), -118.35 (ddd, J = 275.9, 56.6, 14.1 Hz, 1F), -122.47 (ddd, J = 275.7, 56.8, 16.6 Hz, 1F); HRMS (ESI): m/z calcd $C_{20}H_{21}F_5N_2$ $[M]^+$ 384.1625, found 384.1634.

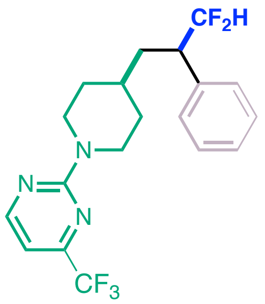

Using general procedure A. **2-(4-(3,3-difluoro-2-phenylpropyl)piperidin-1-yl)-4-(trifluoromethyl)pyrimidine (42):** 0.25 mmol scale, 58 mg, 61%, white solid, 2% EA in Hexane, R_f = 0.19.

^{1}H NMR (400 MHz, $CDCl_3$) δ 8.47 (d, J = 4.7 Hz, 1H), 7.45 – 7.28 (m, 5H), 6.71 (d, J = 4.8 Hz, 1H), 5.85 (td, J = 56.7, 3.8 Hz, 1H), 4.77 (t, J = 15.3 Hz, 2H), 3.29 – 3.08 (m, 1H), 2.79 (q, J = 14.5 Hz, 2H), 1.95 – 1.74 (m, 3H), 1.68 (d, J = 13.1 Hz, 1H), 1.52 – 1.40 (m, 1H), 1.32 – 1.15 (m, 2H); ^{13}C NMR (101 MHz, $CDCl_3$) δ 161.4, 160.1, 156.3 (q, J = 35.3 Hz), 136.8 (t, J = 4.0 Hz), 128.9, 127.9, 120.8 (q, J = 274.8 Hz), 118.2 (t, J = 245.0 Hz), 104.1, 104.0, 47.2 (t, J = 20.0 Hz), 44.0, 43.9, 34.9 (t, J = 4.0 Hz), 33.1, 32.9, 31.1; ^{19}F NMR (376 MHz, $CDCl_3$) δ -70.89 (s, 3F), -118.40 (ddd, J = 275.8, 56.8, 14.4 Hz, 1F), -122.41 (ddd, J = 275.4, 56.9, 16.6 Hz, 1F); HRMS (ESI): m/z calcd $C_{19}H_{20}F_5N_3Na$ $[M+Na]^+$ 408.1470, found 408.1472.

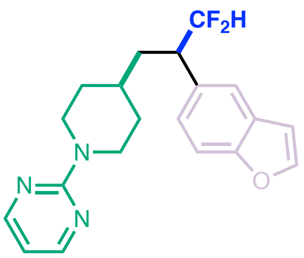

Using general procedure A. **2-(4-(2-(benzofuran-5-yl)-3,3-difluoropropyl)piperidin-1-yl)pyrimidine (43):** 0.25 mmol scale, 55 mg, 62%, colorless oil, 10% EA in Hexane, R_f = 0.14.

^{1}H NMR (400 MHz, $CDCl_3$) δ 8.26 (d, J = 4.0 Hz, 2H), 7.64 (s, 1H), 7.49 (d, J = 6.4 Hz, 2H), 7.19 (d, J = 8.5 Hz, 1H), 6.76 (s, 1H), 6.40 (t, J = 4.3 Hz, 1H), 6.01 – 5.65 (m, 1H), 4.76 – 4.56 (m, 2H), 3.25 (q, J = 15.0 Hz, 1H), 2.71 (q, J = 12.4 Hz, 2H), 1.95 – 1.72 (m, 3H), 1.63 (d, J = 12.3 Hz, 1H), 1.48 – 1.35 (m, 1H), 1.29 – 1.08 (m, 2H); ^{13}C NMR (101 MHz, $CDCl_3$) δ 161.6, 157.8, 154.6, 145.7, 131.3 (t, J = 4.0 Hz), 128.0, 125.1, 121.5, 118.4 (t, J = 244.9 Hz), 111.7, 109.4, 106.7, 47.0 (t, J = 19.7 Hz), 44.0, 43.8, 35.3 (t, J = 3.0 Hz), 33.1, 33.0, 31.1; ^{19}F NMR (376 MHz, $CDCl_3$) δ -118.56 (ddd, J = 274.8, 56.7, 14.5 Hz, 1F), -122.19 (ddd, J = 274.5, 56.9, 16.3 Hz, 1F); HRMS (ESI): m/z calcd $C_{20}H_{21}F_2N_3NaO$ $[M+Na]^+$ 380.1545, found 380.1540.

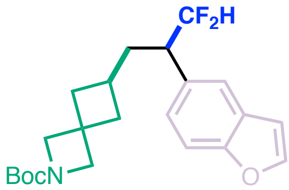

Using general procedure A. **tert-butyl-6-(2-(benzofuran-5-yl)-3,3-difluoropropyl)-2-azaspiro[3.3]heptane-2-carboxylate (44):** 0.25 mmol scale, 58 mg, 60%, colorless oil, 10% EA in Hexane, $R_f = 0.15$.

^{1}H NMR (400 MHz, $CDCl_3$) δ 7.63 (d, J = 1.8 Hz, 1H), 7.53 – 7.36 (m, 2H), 7.11 (d, J = 8.4 Hz, 1H), 6.75 (s, 1H), 5.83 (td, J = 56.8, 3.8 Hz, 1H), 3.77 (dd, J = 21.6, 4.6 Hz, 3H), 3.06 – 2.90 (m, 1H), 2.25 – 2.14 (m, 1H), 2.11 – 1.85 (m, 4H), 1.84 – 1.75 (m, 1H), 1.73 – 1.60 (m, 2H), 1.40 (s, 9H); ^{13}C NMR (101 MHz, $CDCl_3$) δ 156.3, 154.6, 145.7, 131.3 (t, J = 4.0 Hz), 127.9, 124.9, 121.4, 118.1 (t, J = 244.6 Hz), 111.7, 106.6, 79.3, 48.3 (t, J = 19.9 Hz), 39.3, 38.8, 35.7 (t, J = 3.0 Hz), 34.3, 31.9, 29.8, 28.5, 27.5; ^{19}F NMR (376 MHz, $CDCl_3$) δ -118.15 (ddd, J = 275.9, 56.8, 14.5 Hz, 1F), -121.93 – -123.04 (m, 1F); HRMS (ESI): m/z calcd $C_{22}H_{27}F_2NNaO_3$ $[M+Na]^+$ 414.1851, found 414.1850.

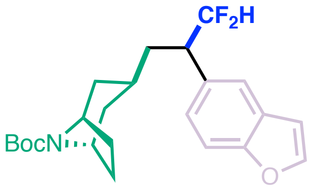

tert-butyl-3-2-(benzofuran-5-yl)-3,3-difluoropropyl)-8-azabicyclo[3.2.1]octane-8-carboxylate (45): 0.25 mmol scale, 56 mg, 55%, colorless oil, 9% EA in Hexane, $R_f = 0.12$.

In a nitrogen-filled glovebox, to a 4 mL vial equipped with a stir bar was added terpyridine (1.1 equiv., 64.6 mg), $(DMPU)_2Zn(CF_2H)_2$ (0.8 equiv., 87 mg), and 800 μL DMSO. The resulting mixture was stirred at room temperature for 1 min to generate (terpy)$Zn(CF_2H)_2$ in-situ. A different 4 mL vial equipped with a stir bar was sequentially charged with CuCl (30

mol %, 7.5 mg), RAEs (0.25 mmol, 1.0 equiv.), the DMSO solution of the in-situ formed (terpy)Zn(CF_2H)$_2$, and alkene (0.75 mmol, 3.0 equiv.) in DMSO (200 μL). The resultant mixture was stirred at room temperature for 12 h. After the reaction was completed, the mixture was diluted with EtOAc (50 mL), filtered through a short plug of Celite, and washed with H_2O (50 mL) and brine. The organic layer was combined, dried over Na_2SO_4, filtered, and then concentrated under reduced pressure. The crude difluoromethylation product was purified by flash column chromatography.

^{1}H NMR (400 MHz, $CDCl_3$) δ 7.63 (s, 1H), 7.47 (d, J = 10.4 Hz, 2H), 7.14 (d, J = 8.2 Hz, 1H), 6.75 (s, 1H), 5.81 (td, J = 56.8, 3.2 Hz, 1H), 4.20 – 4.05 (m, 2H), 3.15 (q, J = 14.9 Hz, 1H), 1.82 – 1.57 (m, 6H), 1.45 (s, 9H), 1.40 – 1.14 (m, 5H); ^{13}C NMR (101 MHz, $CDCl_3$) δ 154.5, 153.5, 145.6, 131.2, 128.0, 125.0, 121.4, 118.4 (t, J = 244.6 Hz), 111.7, 106.7, 79.2, 53.7, 53.1, 47.0 (t, J = 21.6 Hz), 38.9, 38.1, 37.4, 36.4, 35.7, 28.6, 25.4; ^{19}F NMR (376 MHz, $CDCl_3$) δ -118.44 (dddd, J = 274.6, 73.1, 56.6, 14.9 Hz, 1F), -122.23 (dtd, J = 275.2, 62.9, 57.8, 16.5 Hz, 1F); HRMS (ESI): m/z calcd $C_{23}H_{29}F_2NNaO_3$ $[M+Na]^+$ 428.2008, found 428.2007.

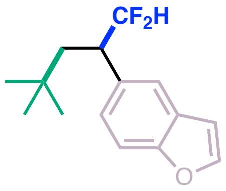

Using general procedure A. **5-(1,1-difluoro-4,4-dimethylpentan-2-yl)benzofuran (46):** 0.25 mmol scale, 42 mg, 67%, white solid, 3% EA in Hexane, R_f = 0.15.

^{1}H NMR (400 MHz, $CDCl_3$) δ 7.63 (s, 1H), 7.53 (s, 1H), 7.48 (d, J = 8.5 Hz, 1H), 7.22 (d, J = 8.5 Hz, 1H), 6.76 (s, 1H), 5.80 (td, J = 57.2, 3.2 Hz, 1H), 3.37 – 3.09 (m, 1H), 1.95 – 1.80 (m, 2H), 0.83 (s, 9H); ^{13}C NMR (101 MHz, $CDCl_3$) δ 154.5, 145.5, 133.4 (q, J = 3.0 Hz), 127.8, 125.6, 121.8, 118.5 (t, J = 245.7 Hz), 111.5, 106.7, 46.9 (t, J = 19.4 Hz), 42.1 (t, J = 3.0 Hz),

31.1, 30.1; ^{19}F NMR (376 MHz, $CDCl_3$) δ -119.17 (ddd, J = 272.4, 57.1, 15.6 Hz, 1F), -121.91 (ddd, J = 272.1, 57.0, 17.6 Hz, 1F); HRMS (EI): m/z calcd $C_{15}H_{18}F_2O$ $[M]^+$ 252.1324, found 252.1333.

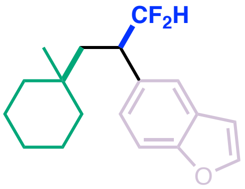

5-(1,1-difluoro-3-(1-methylcyclohexyl)propan-2-yl)benzofuran (47): 0.25 mmol scale, 52 mg, 71%, colorless oil, Hexane, R_f = 0.15.

In a nitrogen-filled glovebox, to a 4 mL vial equipped with a stir bar was added terpyridine (1.1 equiv., 64.6 mg), $(DMPU)_2Zn(CF_2H)_2$ (0.8 equiv., 87 mg), and 800 μL DMSO. The resulting mixture was stirred at room temperature for 1 min to generate (terpy)$Zn(CF_2H)_2$ in-situ. A different 4 mL vial equipped with a stir bar was sequentially charged with CuCl (30 mol %, 7.5 mg), RAEs (0.25 mmol, 1.0 equiv.), the DMSO solution of the in-situ formed (terpy)$Zn(CF_2H)_2$, and alkene (0.75 mmol, 3.0 equiv.) in DMSO (200 μL). The resultant mixture was stirred at room temperature for 12 h. After the reaction was completed, the mixture was diluted with EtOAc (50 mL), filtered through a short plug of Celite, and washed with H_2O (50 mL) and brine. The organic layer was combined, dried over Na_2SO_4, filtered, and then concentrated under reduced pressure. The crude difluoromethylation product was purified by flash column chromatography.

^1H NMR (400 MHz, $CDCl_3$) δ 7.62 (s, 1H), 7.53 (s, 1H), 7.47 (d, J = 8.5 Hz, 1H), 7.22 (d, J = 8.4 Hz, 1H), 6.75 (s, 1H), 5.79 (td, J = 57.2, 3.3 Hz, 1H), 3.31 – 3.10 (m, 1H), 1.90 (d, J = 8.3 Hz, 2H), 1.49 – 1.20 (m, 8H), 1.08 (s, 2H), 0.77 (s, 3H); ^{13}C NMR (101 MHz, $CDCl_3$) δ 154.4, 145.5, 133.7 (t, J = 4.0 Hz), 127.8, 125.6, 121.8, 118.6 (t, J = 244.9 Hz), 111.4, 106.7, 45.9 (t,

J = 19.3 Hz), 40.6, 38.4, 38.3, 33.4, 26.4, 25.3, 22.1, 21.9; ^{19}F NMR (376 MHz, $CDCl_3$) δ -119.01 (ddd, J = 271.8, 57.2, 15.7 Hz, 1F), -121.81 (ddd, J = 271.7, 57.2, 18.0 Hz, 1F); HRMS (EI): m/z calcd $C_{18}H_{22}F_2O$ $[M]^+$ 292.1639, found 292.1641.

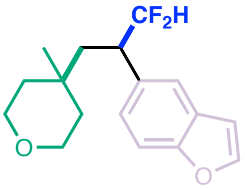

5-(1,1-difluoro-3-(4-methyltetrahydro-2H-pyran-4-yl)propan-2-yl)benzofuran (48): 0.25 mmol scale, 48 mg, 65%, colorless oil, 5% EA in Hexane, R_f = 0.12.

In a nitrogen-filled glovebox, to a 4 mL vial equipped with a stir bar was added terpyridine (1.1 equiv., 64.6 mg), $(DMPU)_2Zn(CF_2H)_2$ (0.8 equiv., 87 mg), and 800 μL DMSO. The resulting mixture was stirred at room temperature for 1 min to generate $(terpy)Zn(CF_2H)_2$ in-situ. A different 4 mL vial equipped with a stir bar was sequentially charged with CuCl (30 mol %, 7.5 mg), RAEs (0.25 mmol, 1.0 equiv.), the DMSO solution of the in-situ formed $(terpy)Zn(CF_2H)_2$, and alkene (0.75 mmol, 3.0 equiv.) in DMSO (200 μL). The resultant mixture was stirred at room temperature for 12 h. After the reaction was completed, the mixture was diluted with EtOAc (50 mL), filtered through a short plug of Celite, and washed with H_2O (50 mL) and brine. The organic layer was combined, dried over Na_2SO_4, filtered, and then concentrated under reduced pressure. The crude difluoromethylation product was purified by flash column chromatography.

^{1}H NMR (400 MHz, $CDCl_3$) δ 7.63 (s, 1H), 7.56 – 7.41 (m, 2H), 7.21 (d, J = 8.4 Hz, 1H), 6.75 (s, 1H), 5.79 (td, J = 57.1, 3.4 Hz, 1H), 3.75 – 3.66 (m, 1H), 3.65 – 3.54 (m, 1H), 3.55 – 3.35 (m, 2H), 3.32 – 3.14 (m, 1H), 2.10 – 1.87 (m, 2H), 1.58 – 1.45 (m, 1H), 1.36 – 1.22 (m, 2H), 1.05 (d, J = 13.5 Hz, 1H), 0.91 (s, 3H); ^{13}C NMR (101 MHz, $CDCl_3$) δ 154.5, 145.7, 133.2 (q,

J = 3.0 Hz), 127.9, 125.4, 121.8, 118.4 (t, J = 245.6 Hz), 111.6, 106.7, 63.9, 63.7, 45.7 (t, J = 18.8 Hz), 40.8, 38.3, 38.1, 31.3, 23.9; ^{19}F NMR (376 MHz, $CDCl_3$) δ -118.44 (ddd, J = 272.6, 57.0, 14.4 Hz, 1F), -122.66 (ddd, J = 272.6, 57.0, 18.7 Hz, 1F); HRMS (EI): m/z calcd $C_{17}H_{20}F_2O_2$ $[M]^+$ 294.1431, found 294.1439.

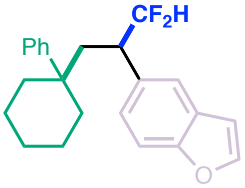

Using general procedure A. **5-(1,1-difluoro-3-(1-phenylcyclohexyl)propan-2-yl)benzofuran (49):** 0.25 mmol scale, 34 mg, 38%, colorless oil, Hexane, R_f = 0.20.

^{1}H NMR (400 MHz, $CDCl_3$) δ 7.52 (s, 1H), 7.28 (d, J = 8.5 Hz, 1H), 7.32 – 7.09 (m, 5H), 7.07 (t, J = 6.6 Hz, 1H), 6.90 (d, J = 8.4 Hz, 1H), 6.62 (s, 1H), 5.37 (td, J = 57.1, 2.8 Hz, 1H), 2.77 – 2.57 (m, 1H), 2.15 – 1.90 (m, 3H), 1.73 (d, J = 11.6 Hz, 1H), 1.65 – 1.45 (m, 2H), 1.33 – 1.08 (m, 6H); ^{13}C NMR (101 MHz, $CDCl_3$) δ 154.3, 145.6, 145.4, 132.9, 128.4, 127.6, 127.2, 125.8, 125.5, 121.8, 118.1 (t, J = 245.1 Hz), 111.2, 106.7, 46.0 (t, J = 19.3 Hz), 42.8, 41.7, 38.3, 35.7, 26.5, 22.5, 22.3; ^{19}F NMR (376 MHz, $CDCl_3$) δ -120.37 (ddd, J = 272.5, 57.0, 17.3 Hz, 1F), -121.65 (ddd, J = 272.6, 57.1, 17.3 Hz, 1F); HRMS (EI): m/z calcd $C_{23}H_{24}F_2O$ $[M]^+$ 354.1795, found 354.1806.

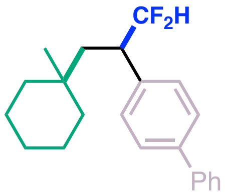

4-(1,1-difluoro-3-(1-methylcyclohexyl)propan-2-yl)-1,1'-biphenyl (50): 0.25 mmol scale, 46 mg, 56%, colorless oil, Hexane, $R_f = 0.10$.

In a nitrogen-filled glovebox, to a 4 mL vial equipped with a stir bar was added terpyridine (1.1 equiv., 64.6 mg), $(DMPU)_2Zn(CF_2H)_2$ (0.8 equiv., 87 mg), and 800 μL DMSO. The resulting mixture was stirred at room temperature for 1 min to generate $(terpy)Zn(CF_2H)_2$ in-situ. A different 4 mL vial equipped with a stir bar was sequentially charged with CuCl (30 mol %, 7.5 mg), RAEs (0.25 mmol, 1.0 equiv.), the DMSO solution of the in-situ formed $(terpy)Zn(CF_2H)_2$, and alkene (0.75 mmol, 3.0 equiv.) in DMSO (200 μL). The resultant mixture was stirred at room temperature for 12 h. After the reaction was completed, the mixture was diluted with EtOAc (50 mL), filtered through a short plug of Celite, and washed with H_2O (50 mL) and brine. The organic layer was combined, dried over Na_2SO_4, filtered, and then concentrated under reduced pressure. The crude difluoromethylation product was purified by flash column chromatography.

^{1}H NMR (400 MHz, $CDCl_3$) δ 7.63 (dd, J = 15.7, 7.9 Hz, 4H), 7.48 (t, J = 7.4 Hz, 2H), 7.40 (d, J = 8.0 Hz, 3H), 5.83 (td, J = 57.1, 3.5 Hz, 1H), 3.31 – 3.09 (m, 1H), 1.97 – 1.85 (m, 2H), 1.54 – 1.41 (m, 3H), 1.38 – 1.28 (m, 5H), 1.18 – 1.12 (m, 2H), 0.83 (s, 3H); ^{13}C NMR (101 MHz, $CDCl_3$) δ 140.8, 140.2, 138.3 (q, J = 3.0 Hz), 129.8, 128.9, 127.4, 127.3, 127.2, 118.4 (t, J = 246.3 Hz), 45.7 (t, J = 19.3 Hz), 40.2, 38.4, 38.3, 33.4, 26.4, 25.3, 22.1, 22.0; ^{19}F NMR (376 MHz, $CDCl_3$) δ -119.09 (ddd, J = 272.1, 57.0, 15.7 Hz, 1F), -121.52 (ddd, J = 272.1, 57.0, 17.5 Hz, 1F); HRMS (EI): m/z calcd $C_{22}H_{26}F_2$ $[M]^+$ 328.2003, found 328.2010.

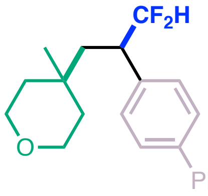

4-(2-([1,1'-biphenyl]-4-yl)-3,3-difluoropropyl)-4-methyltetrahydro-2H-pyran (51): 0.25 mmol scale, 51 mg, 62%, colorless oil, 5% EA in Hexane, $R_f = 0.15$.

In a nitrogen-filled glovebox, to a 4 mL vial equipped with a stir bar was added terpyridine (1.1 equiv., 64.6 mg), $(DMPU)_2Zn(CF_2H)_2$ (0.8 equiv., 87 mg), and 800 μL DMSO. The resulting mixture was stirred at room temperature for 1 min to generate $(terpy)Zn(CF_2H)_2$ in-situ. A different 4 mL vial equipped with a stir bar was sequentially charged with CuCl (30 mol %, 7.5 mg), RAEs (0.25 mmol, 1.0 equiv.), the DMSO solution of the in-situ formed $(terpy)Zn(CF_2H)_2$, and alkene (0.75 mmol, 3.0 equiv.) in DMSO (200 μL). The resultant mixture was stirred at room temperature for 12 h. After the reaction was completed, the mixture was diluted with EtOAc (50 mL), filtered through a short plug of Celite, and washed with H_2O (50 mL) and brine. The organic layer was combined, dried over Na_2SO_4, filtered, and then concentrated under reduced pressure. The crude difluoromethylation product was purified by flash column chromatography.

^{1}H NMR (400 MHz, $CDCl_3$) δ 7.59 (t, J = 8.6 Hz, 4H), 7.45 (t, J = 7.5 Hz, 2H), 7.36 (d, J = 7.7 Hz, 3H), 5.80 (td, J = 57.0, 3.4 Hz, 1H), 3.76 – 3.68 (m, 1H), 3.65 – 3.43 (m, 3H), 3.25 – 3.12 (m, 1H), 2.04 – 1.87 (m, 2H), 1.60 – 1.50 (m, 1H), 1.37 – 1.27 (m, 2H), 1.11 (d, J = 13.5 Hz, 1H), 0.95 (s, 3H); ^{13}C NMR (101 MHz, $CDCl_3$) δ 140.6, 140.5, 137.7 (q, J = 3.0 Hz), 129.7, 128.9, 127.5, 127.5, 127.1, 118.2 (t, J = 244.8 Hz), 63.9, 63.8, 45.5 (t, J = 19.9 Hz), 40.5, 38.2, 38.1, 31.3, 24.0; ^{19}F NMR (376 MHz, $CDCl_3$) δ -118.52 (ddd, J = 272.9, 56.9, 14.4 Hz, 1F), -122.38 (ddd, J = 272.9, 57.0, 18.3 Hz, 1F); MS (EI): m/z calcd $C_{21}H_{24}F_2O$ $[M]^+$ 330.2, found 330.2; HRMS (EI): m/z calcd $C_{21}H_{23}FO$ $[M-HF]^+$ 310.1733, found 310.1743.

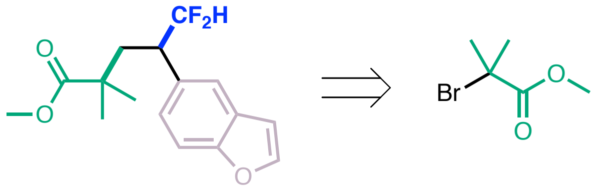

Using general procedure C.

methyl-4-(benzofuran-5-yl)-5,5-difluoro-2,2-dimethylpentanoate (52): 0.25 mmol scale, 40 mg, 54%, colorless oil, 4% EA in Hexane, $R_f = 0.24$.

^{1}H NMR (400 MHz, $CDCl_3$) δ 7.61 (s, 1H), 7.54 – 7.39 (m, 2H), 7.17 (d, J = 8.5 Hz, 1H), 6.74 (s, 1H), 5.79 (t, J = 56.8 Hz, 1H), 3.18 (s, 4H), 2.30 – 2.08 (m, 2H), 1.19 (s, 3H), 1.11 (s, 3H); ^{13}C NMR (101 MHz, $CDCl_3$) δ 177.4, 154.5, 145.6, 131.4 (t, J = 4.0 Hz), 127.8, 125.7, 122.1, 118.0 (t, J = 245.5 Hz), 111.4, 106.6, 51.5, 46.9 (t, J = 19.9 Hz), 41.5, 39.2 (t, J = 4.0 Hz), 26.8, 24.9; ^{19}F NMR (376 MHz, $CDCl_3$) δ -119.00 (ddd, J = 274.4, 56.8, 14.8 Hz, 1F), -122.83 (ddd, J = 274.1, 56.9, 18.1 Hz, 1F); HRMS (EI): m/z calcd $C_{16}H_{18}F_2O_3$ $[M]^+$ 296.1228, found 296.1224.

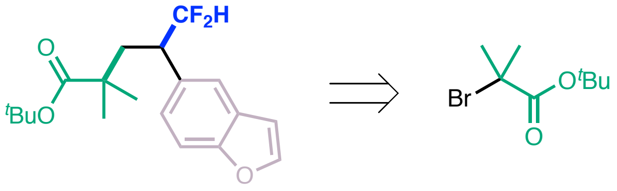

Using general procedure C.

tert-butyl-4-(benzofuran-5-yl)-5,5-difluoro-2,2-dimethylpentanoate (53): 0.25 mmol scale, 46 mg, 54%, colorless oil, 3% EA in Hexane, $R_f = 0.21$.

^{1}H NMR (400 MHz, $CDCl_3$) δ 7.61 (s, 1H), 7.55 – 7.37 (m, 2H), 7.19 (d, J = 8.5 Hz, 1H), 6.74 (s, 1H), 5.80 (td, J = 56.9, 2.3 Hz, 1H), 3.15 (q, J = 16.5 Hz, 1H), 2.26 (d, J = 14.2 Hz, 1H), 2.15 – 2.02 (m, 1H), 1.31 (s, 9H), 1.13 (s, 3H), 0.95 (s, 3H); ^{13}C NMR (101 MHz, $CDCl_3$) δ

176.6, 154.6, 145.6, 132.3 (q, J = 3.0 Hz), 127.9, 125.7, 122.0, 118.1 (t, J = 245.6 Hz), 111.5, 106.7, 80.3, 47.2 (t, J = 19.6 Hz), 42.7, 38.9 (t, J = 4.0 Hz), 27.9, 26.6, 25.7; ^{19}F NMR (376 MHz, $CDCl_3$) δ -119.44 (ddd, J = 273.4, 56.9, 15.8 Hz, 1F), -122.21 (ddd, J = 273.8, 57.0, 17.5 Hz, 1F); HRMS (EI): m/z calcd $C_{19}H_{24}F_2O_3$ $[M]^+$ 338.1694, found 338.1711.

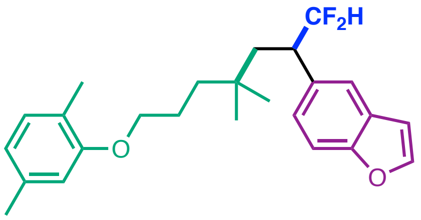

Using general procedure A. **5-(7-(2,5-dimethylphenoxy)-1,1-difluoro-4,4-dimethylheptan-2-yl)benzofuran (54):** 0.25 mmol scale, 62 mg, 62%, colorless oil, 2% EA in Hexane, R_f = 0.14.

^{1}H NMR (400 MHz, $CDCl_3$) δ 7.62 (d, J = 2.0 Hz, 1H), 7.53 (s, 1H), 7.47 (d, J = 8.5 Hz, 1H), 7.23 (d, J = 8.5 Hz, 1H), 7.01 (d, J = 7.4 Hz, 1H), 6.78 – 6.72 (m, 1H), 6.67 (d, J = 7.4 Hz, 1H), 6.55 (s, 1H), 5.80 (td, J = 57.1, 3.6 Hz, 1H), 3.76 (t, J = 6.3 Hz, 2H), 3.29 – 3.13 (m, 1H), 2.32 (s, 3H), 2.17 (s, 3H), 1.98 – 1.87 (m, 2H), 1.74 (tt, J = 12.1, 6.3 Hz, 1H), 1.67 – 1.54 (m, 1H), 1.40 (td, J = 12.9, 12.5, 4.6 Hz, 1H), 1.31 (d, J = 4.4 Hz, 1H), 0.82 (s, 6H); ^{13}C NMR (101 MHz, $CDCl_3$) δ 157.1, 154.5, 145.6, 136.6, 133.4 (q, J = 3.0 Hz), 130.4, 127.9, 125.5, 123.7, 121.8, 120.7, 118.5 (t, J = 245.7 Hz), 112.1, 111.5, 106.6, 68.5, 46.4 (t, J = 19.5 Hz), 39.7, 38.5, 33.3, 28.0, 27.8, 24.3, 21.5, 15.9; ^{19}F NMR (376 MHz, $CDCl_3$) δ -118.81 (ddd, J = 272.1, 57.0, 15.1 Hz, 1F), -122.16 (ddd, J = 272.1, 57.2, 18.1 Hz, 1F); HRMS (EI): m/z calcd $C_{25}H_{30}F_2O_2$ $[M]^+$ 400.2214, found 400.2219.

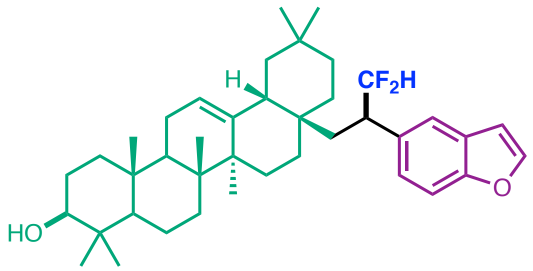

Using general procedure A. **From oleanolic acid (55):** 0.25 mmol scale, 65 mg, 43%, 1.2:1 dr, white solid, 10% EA in Hexane, R_f = 0.15.

(Mixture of diastereoisomers) ^{1}H NMR (400 MHz, $CDCl_3$) δ 7.62 (s, 1H), 7.54 – 7.40 (m, 2H), 7.19 (d, J = 8.4 Hz, 1H), 6.74 (s, 1H), 5.77 (tdd, J = 57.1, 18.8, 3.0 Hz, 1H), 5.08 (d, J = 123.3 Hz, 1H), 3.27 – 3.09 (m, 2H), 2.18 – 1.98 (m, 2H), 1.92 – 1.74 (m, 4H), 1.72 – 1.54 (m, 6H), 1.47 – 1.25 (m, 6H), 1.15 (s, 3H), 1.07 – 0.98 (m, 7H), 0.96 – 0.86 (m, 10H), 0.81 – 0.73 (m, 9H); (Mixture of diastereoisomers) ^{13}C NMR (101 MHz, $CDCl_3$) δ 154.4, 145.5, 144.4, 133.5 (t, J = 4.0 Hz), 127.7, 125.8, 122.8, 122.0, 118.8 (t, J = 245.3 Hz), 111.3, 106.7, 79.1, 55.3, 47.7, 47.6, 46.9, 45.8 (t, J = 19.1 Hz), 41.7, 40.0, 38.9, 38.8, 38.7, 37.0, 35.9, 34.6, 34.1, 33.3, 33.2, 32.7, 31.0, 28.2, 27.3, 26.3, 25.5, 24.0, 23.7, 18.5, 17.1, 15.7, 15.6; (Mixture of diastereoisomers) ^{19}F NMR (376 MHz, $CDCl_3$) δ -117.34 – -119.94 (m, 1F), -120.06 – -121.93 (m, 1F); HRMS (ESI): m/z calcd $C_{40}H_{57}F_2O_2$ $[M+H]^+$ 607.4321, found 607.4319.

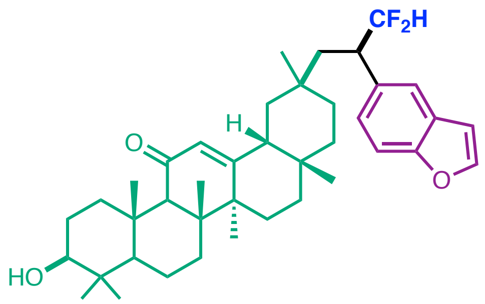

Using general procedure A. **From Glycyrrhetinic acid (56):** 0.25 mmol scale, 97 mg, 63%, white solid, 20% EA in Hexane, R_f = 0.12.

(Mixture of diastereoisomers) ^{1}H NMR (400 MHz, $CDCl_3$) δ 7.68 – 7.58 (m, 1H), 7.54 – 7.41 (m, 2H), 7.18 (t, J = 8.5 Hz, 1H), 6.74 (t, J = 9.1 Hz, 1H), 5.96 – 5.40 (m, 2H), 3.31 – 3.07 (m, 2H), 2.84 – 2.71 (m, 1H), 2.37 – 2.18 (m, 1H), 2.14 – 1.94 (m, 2H), 1.83 (s, 3H), 1.68 – 1.51 (m, 6H), 1.42 – 1.21 (m, 8H), 1.09 (dq, J = 17.8, 6.0, 3.5 Hz, 10H), 1.00 (t, J = 10.3 Hz, 5H), 0.83 – 0.73 (m, 9H); (Mixture of diastereoisomers) ^{13}C NMR (101 MHz, $CDCl_3$) δ 200.4, 170.2, 154.4, 145.6, 128.3, 128.1, 127.8, 125.4, 121.9, 118.4 (t, J = 244.6 Hz), 111.6, 106.6, 78.9, 61.9, 55.0, 47.2, 45.9, 44.1, 43.5, 39.2, 37.2, 35.9, 34.4, 34.0, 33.7, 33.2, 32.8, 32.6, 30.3, 28.7, 28.2, 27.4, 26.7, 26.4, 23.2, 21.4, 18.8, 17.6, 16.4, 15.7; (Mixture of diastereoisomers) ^{19}F NMR (376 MHz, $CDCl_3$) δ -116.50 – -119.21 (m, 1F), -121.82 – -123.99 (m, 1F); HRMS (ESI): m/z calcd $C_{40}H_{55}F_2O_3$ $[M+H]^+$ 621.4114, found 621.4115.

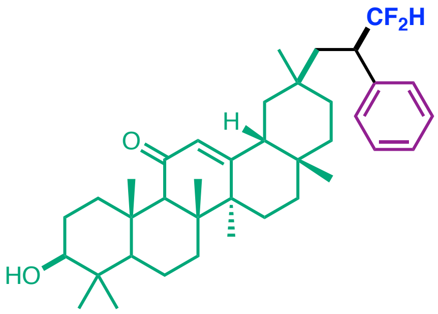

Using general procedure A. **From Glycyrrhetinic acid (57):** 0.25 mmol scale, 85 mg, 59%, white solid, 20% EA in Hexane, R_f = 0.13.

(Mixture of diastereoisomers) ^{1}H NMR (400 MHz, $CDCl_3$) δ 7.36 – 7.24 (m, 5H), 5.94 – 5.47 (m, 2H), 3.32 – 3.02 (m, 2H), 2.86 – 2.73 (m, 1H), 2.38 – 2.22 (m, 1H), 2.15 – 2.00 (m, 2H), 1.87 – 1.73 (m, 3H), 1.70 – 1.53 (m, 5H), 1.51 – 1.39 (m, 4H), 1.35 – 1.21 (m, 4H), 1.20 – 1.06 (m, 10H), 1.02 (t, J = 9.3 Hz, 5H), 0.82 (dt, J = 10.6, 5.6 Hz, 9H); (Mixture of diastereoisomers) ^{13}C NMR (101 MHz, $CDCl_3$) δ 200.2, 169.3, 129.3, 128.7, 128.4, 128.1, 127.6, 118.2 (t, J = 245.3 Hz), 78.9, 61.9, 55.0 (t, J = 4.0 Hz), 47.2, 46.7, 45.9 (t, J = 19.9 Hz), 45.5, 44.0, 43.5,

43.3, 39.2, 37.1, 36.0, 34.3, 32.8, 32.6, 30.2, 28.8, 28.2, 27.4, 26.4, 23.4, 22.0, 18.8, 18.8 (t, J = 4.0 Hz), 17.6, 16.5, 15.7; (Mixture of diastereoisomers) ^{19}F NMR (376 MHz, $CDCl_3$) δ -116.62 – -119.20 (m, 1F), -121.96 – -123.94 (m, 1F); HRMS (ESI): m/z calcd $C_{38}H_{55}F_2O_2$ $[M+H]^+$ 581.4165, found 581.4166.

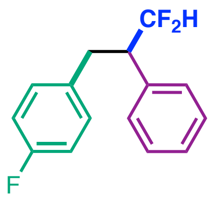

Using general procedure D. **1-(3,3-difluoro-2-phenylpropyl)-4-fluorobenzene (58):** 0.25 mmol scale, 38 mg, 61%, yellow oil, Hexane, R_f = 0.15.

^{1}H NMR (400 MHz, $CDCl_3$) δ 7.34 (t, J = 7.6 Hz, 3H), 7.23 (d, J = 7.0 Hz, 2H), 7.06 – 6.98 (m, 2H), 6.93 (t, J = 8.5 Hz, 2H), 6.14 – 5.78 (m, 1H), 3.38 – 3.24 (m, 2H), 3.11 – 2.98 (m, 1H); ^{13}C NMR (101 MHz, $CDCl_3$) δ 161.6 (d, J = 244.6 Hz), 136.2 (t, J = 4.0 Hz), 134.2 (d, J = 3.0 Hz), 130.6 (d, J = 8.0 Hz), 129.1, 128.7, 127.8, 117.38 (t, J = 244.9 Hz), 115.3 (d, J = 21.2 Hz), 52.1 (t, J = 19.8 Hz), 34.51 (dd, J = 5.3, 3.7 Hz); ^{19}F NMR (376 MHz, $CDCl_3$) δ -116.68 (h, J = 6.4, 5.7 Hz, 1F), -119.71 (ddd, J = 278.6, 56.2, 15.5 Hz, 1F), -122.89 (ddd, J = 278.7, 56.7, 15.9 Hz, 1F); HRMS (EI): m/z calcd $C_{15}H_{13}F_3$ $[M]^+$ 250.0969, found 250.0977.

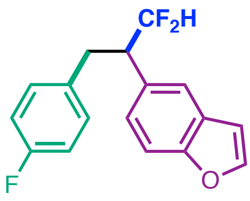

Using general procedure D. **5-(1,1-difluoro-3-(4-fluorophenyl)propan-2-yl)benzofuran (59):** 0.25 mmol scale, 52 mg, 72%, colorless oil, Hexane, R_f = 0.20.

^{1}H NMR (400 MHz, $CDCl_3$) δ 7.61 (s, 1H), 7.43 (d, J = 11.6 Hz, 2H), 7.11 (d, J = 8.4 Hz, 1H), 6.92 (dt, J = 45.7, 8.3 Hz, 4H), 6.72 (s, 1H), 5.94 (td, J = 56.6, 3.1 Hz, 1H), 3.42 – 3.23 (m, 2H), 3.04 (dd, J = 13.4, 10.1 Hz, 1H); ^{13}C NMR (101 MHz, $CDCl_3$) δ 160.6 (d, J = 244.7 Hz), 154.6, 145.6, 134.2 (d, J = 3.0 Hz), 130.6, 130.5, 127.9, 125.3, 121.7, 117.5 (t, J = 244.4 Hz), 115.3 (d, J = 21.2 Hz), 111.6, 106.7, 52.0 (t, J = 19.7 Hz), 34.8 (dd, J = 5.2, 3.6 Hz); ^{19}F NMR (376 MHz, $CDCl_3$) δ -116.74 (dq, J = 14.1, 6.1 Hz, 1F), -119.68 (ddd, J = 277.5, 56.3, 15.4 Hz, 1F), -122.99 (ddd, J = 277.5, 56.5, 16.0 Hz, 1F); HRMS (EI): m/z calcd $C_{17}H_{13}F_3O$ $[M]^+$ 290.0918, found 290.0925.

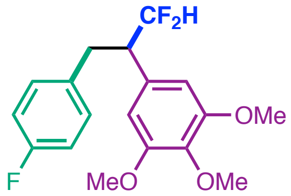

Using general procedure D. **5-(1,1-difluoro-3-(4-fluorophenyl)propan-2-yl)-1,2,3-trimethoxybenzene (60):** 0.25 mmol scale, 47 mg, 55%, colorless oil, 5% EA in Hexane, R_f = 0.14.

^{1}H NMR (400 MHz, $CDCl_3$) δ 7.05 – 6.84 (m, 2H), 6.90 (t, J = 8.7 Hz, 2H), 6.34 (s, 2H), 5.90 (td, J = 56.3, 2.6 Hz, 1H), 3.83 (s, 3H), 3.80 (s, 6H), 3.24 – 3.07 (m, 2H), 2.94 (dd, J = 13.3, 9.3 Hz, 1H); ^{13}C NMR (101 MHz, $CDCl_3$) δ 161.7 (d, J = 243.8 Hz), 153.3, 137.6, 134.1 (d, J = 3.3 Hz), 131.88 (dd, J = 4.9, 2.6 Hz), 130.6 (d, J = 8.0 Hz), 117.2 (t, J = 244.4 Hz), 115.3 (d, J = 21.3 Hz), 106.1, 61.0, 56.3, 52.21 (t, J = 19.9 Hz), 34.61 (dd, J = 5.5, 3.3 Hz); ^{19}F NMR (376 MHz, $CDCl_3$) δ -116.56 (ddd, J = 14.5, 9.1, 5.7 Hz, 1F), -119.58 (ddd, J = 278.6, 56.3, 15.5 Hz, 1F), -123.12 (ddd, J = 277.9, 56.3, 16.0 Hz, 1F); HRMS (EI): m/z calcd $C_{18}H_{19}F_3O_3$ $[M]^+$ 340.1286, found 340.1295.

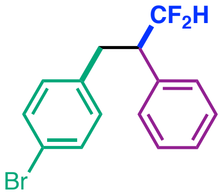

Using general procedure D. **1-bromo-4-(3,3-difluoro-2-phenylpropyl)benzene (61):** 0.25 mmol scale, 51 mg, 65%, white solid, Hexane, $R_f = 0.17$.

^{1}H NMR (400 MHz, $CDCl_3$) δ 7.28 – 7.15 (m, 5H), 7.09 (d, J = 6.7 Hz, 2H), 6.81 (d, J = 8.0 Hz, 2H), 6.00 – 5.66 (m, 1H), 3.22 – 3.09 (m, 2H), 2.94 – 2.84 (m, 1H); ^{13}C NMR (101 MHz, $CDCl_3$) δ 137.5, 136.1 (dd, J = 4.6, 2.6 Hz), 131.6, 130.9, 129.1, 128.8, 127.9, 120.4, 117.3 (t, J = 244.6 Hz), 51.8 (t, J = 19.8 Hz), 34.6 (dd, J = 5.4, 3.6 Hz); ^{19}F NMR (376 MHz, $CDCl_3$) δ -119.47 (ddd, J = 277.9, 56.4, 15.2 Hz, 1F), -123.08 (ddd, J = 278.4, 56.6, 16.4 Hz, 1F); HRMS (EI): m/z calcd $C_{15}H_{13}BrF_2$ $[M]^+$ 310.0169, found 310.0176.

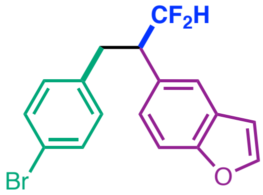

Using general procedure D. **5-(3-(4-bromophenyl)-1,1-difluoropropan-2-yl)benzofuran (62):** 0.25 mmol scale, 51 mg, 71%, colorless oil, Hexane, $R_f = 0.12$.

^{1}H NMR (400 MHz, $CDCl_3$) δ 7.54 (d, J = 2.2 Hz, 1H), 7.39 – 7.30 (m, 2H), 7.24 – 7.17 (m, 2H), 7.02 (d, J = 8.5 Hz, 1H), 6.81 (d, J = 8.0 Hz, 2H), 6.64 (d, J = 2.1 Hz, 1H), 5.86 (td, J = 56.4, 2.9 Hz, 1H), 3.33 – 3.13 (m, 2H), 2.94 (dd, J = 13.7, 10.3 Hz, 1H); ^{13}C NMR (101 MHz, $CDCl_3$) δ 154.6, 145.7, 137.6, 131.6, 130.9, 130.5, 127.9, 125.2, 121.7, 120.4, 117.5 (t, J = 245.0 Hz), 111.6, 106.7, 51.8 (t, J = 19.6 Hz), 35.0 (t, J = 4.0 Hz); ^{19}F NMR (376 MHz, $CDCl_3$)

δ -119.39 (ddd, J = 277.6, 56.3, 15.1 Hz, 1F), -123.12 (ddd, J = 277.6, 56.4, 16.4 Hz, 1F); HRMS (EI): m/z calcd $C_{17}H_{13}BrF_2O$ $[M]^+$ 350.0118, found 350.0120.

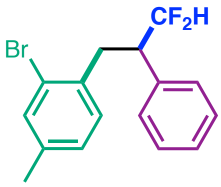

Using general procedure D. **2-bromo-1-(3,3-difluoro-2-phenylpropyl)-4-methylbenzene (63):** 0.25 mmol scale, 42 mg, 52%, colorless oil, Hexane, R_f = 0.15.

^{1}H NMR (400 MHz, $CDCl_3$) δ 7.38 – 7.20 (m, 6H), 6.89 (d, J = 7.7 Hz, 1H), 6.81 (d, J = 7.7 Hz, 1H), 6.12 – 5.77 (m, 1H), 3.55 – 3.34 (m, 2H), 3.07 (dd, J = 13.5, 8.9 Hz, 1H), 2.25 (s, 3H); ^{13}C NMR (101 MHz, $CDCl_3$) δ 138.4, 136.4 (dd, J = 4.2, 2.6 Hz), 134.5, 133.5, 131.3, 129.2, 128.6, 128.1, 127.8, 124.4, 117.4 (t, J = 244.4 Hz), 49.5 (t, J = 19.6 Hz), 35.5 (dd, J = 5.9, 3.2 Hz), 20.7; ^{19}F NMR (376 MHz, $CDCl_3$) δ -120.18 (ddd, J = 278.1, 56.3, 17.4 Hz, 1F), -122.42 (ddd, J = 277.7, 56.3, 14.7 Hz, 1F); HRMS (EI): m/z calcd $C_{16}H_{15}BrF_2$ $[M]^+$ 324.0331, found 324.0325.

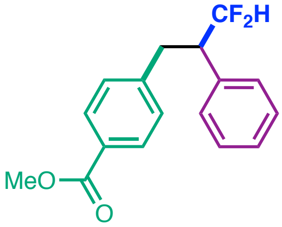

Using general procedure D. **Methyl-4-(3,3-difluoro-2-phenylpropyl)benzoate (64):** 0.25 mmol scale, 49 mg, 67%, colorless oil, 5% EA in Hexane, R_f = 0.12.

^{1}H NMR (400 MHz, $CDCl_3$) δ 7.87 (d, J = 8.1 Hz, 2H), 7.35 – 7.21 (m, 3H), 7.17 (d, J = 7.3 Hz, 2H), 7.09 (d, J = 8.1 Hz, 2H), 5.93 (td, J = 56.6, 3.1 Hz, 1H), 3.87 (s, 3H), 3.38 – 3.22 (m,

2H), 3.13 – 3.02 (m, 1H); ^{13}C NMR (101 MHz, $CDCl_3$) δ 167.1, 144.0, 135.9 (dd, J = 5.0, 2.7 Hz), 129.8, 129.2, 129.1, 128.8, 128.5, 127.9, 117.3 (t, J = 245.3 Hz), 52.1, 51.7 (t, J = 19.8 Hz), 35.2 (dd, J = 5.5, 3.5 Hz); ^{19}F NMR (376 MHz, $CDCl_3$) δ -119.23 (ddd, J = 277.7, 56.2, 14.6 Hz, 1F), -123.08 (ddd, J = 277.6, 56.3, 16.2 Hz, 1F); HRMS (EI): m/z calcd $C_{17}H_{16}F_2O_2$ $[M]^+$ 290.1118, found 290.1119.

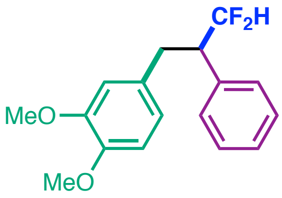

Using general procedure D. **4-(3,3-difluoro-2-phenylpropyl)-1,2-dimethoxybenzene (65):** 0.25 mmol scale, 44 mg, 60%, colorless oil, 5% EA in Hexane, R_f = 0.12.

^{1}H NMR (400 MHz, $CDCl_3$) δ 7.22 (q, J = 9.5, 8.0 Hz, 3H), 7.11 (d, J = 7.2 Hz, 2H), 6.63 (d, J = 8.1 Hz, 1H), 6.52 (d, J = 8.1 Hz, 1H), 6.34 (s, 1H), 6.01 – 5.66 (m, 1H), 3.74 (s, 3H), 3.62 (s, 3H), 3.23 – 3.07 (m, 2H), 2.96 – 2.83 (m, 1H); ^{13}C NMR (101 MHz, $CDCl_3$) δ 148.7, 147.6, 136.6 (t, J = 3.0 Hz), 130.9, 129.3, 128.6, 127.7, 121.2, 117.4 (t, J = 244.1 Hz), 112.4, 111.1, 55.9, 55.8, 52.1 (t, J = 19.8 Hz), 35.1 (t, J = 4.0 Hz); ^{19}F NMR (376 MHz, $CDCl_3$) δ -120.35 (ddd, J = 277.6, 56.3, 16.1 Hz, 1F), -122.70 (ddd, J = 277.6, 56.0, 14.6 Hz, 1F); HRMS (EI): m/z calcd $C_{17}H_{18}F_2O_2$ $[M]^+$ 292.1275, found 292.1284.

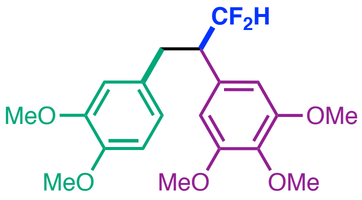

Using general procedure D. **5-(3-(3,4-dimethoxyphenyl)-1,1-difluoropropan-2-yl)-1,2,3-trimethoxybenzene (66):** 0.25 mmol scale, 62 mg, 65%, colorless oil, 15% EA in Hexane, R_f = 0.12.

^{1}H NMR (400 MHz, $CDCl_3$) δ 6.63 (d, J = 8.1 Hz, 1H), 6.51 (d, J = 8.1 Hz, 1H), 6.39 (s, 1H), 6.28 (s, 2H), 5.80 (td, J = 56.3, 2.8 Hz, 1H), 3.75 – 3.66 (m, 12H), 3.65 (s, 3H), 3.10 – 2.92 (m, 2H), 2.88 – 2.80 (m, 1H); ^{13}C NMR (101 MHz, $CDCl_3$) δ 153.3, 148.8, 147.7, 137.5, 132.3 (dd, J = 4.5, 2.8 Hz), 130.9, 121.3, 117.3 (t, J = 244.3 Hz), 112.4, 111.2, 106.3, 61.0, 56.3, 56.0, 55.8, 52.2 (t, J = 19.7 Hz), 35.2 (dd, J = 5.3, 3.0 Hz); ^{19}F NMR (376 MHz, $CDCl_3$) δ -120.40 (ddd, J = 278.6, 56.5, 16.8 Hz, 1F), -122.85 (ddd, J = 278.8, 56.5, 15.3 Hz, 1F); HRMS (EI): m/z calcd $C_{20}H_{24}F_2O_5$ $[M]^+$ 382.1592, found 382.1603.

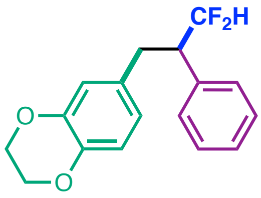

Using general procedure D.

6-(3,3-difluoro-2-phenylpropyl)-2,3-dihydrobenzo[b][1,4]dioxine (67): 0.25 mmol scale, 34 mg, 47%, colorless oil, 10% EA in Hexane, R_f = 0.13.

^{1}H NMR (400 MHz, $CDCl_3$) δ 7.35 – 7.19 (m, 5H), 6.70 (d, J = 8.2 Hz, 1H), 6.59 (s, 1H), 6.52 (d, J = 8.2 Hz, 1H), 5.89 (td, J = 56.4, 3.1 Hz, 1H), 4.20 (s, 4H), 3.32 – 3.12 (m, 2H), 2.93 (dd, J = 13.7, 9.1 Hz, 1H); ^{13}C NMR (101 MHz, $CDCl_3$) δ 143.4, 142.2, 136.5 (t, J = 4.0 Hz), 131.7, 129.2, 128.6, 127.7, 122.1, 117.8, 117.3 (t, J = 244.6 Hz), 117.2, 64.5, 64.4, 51.7 (t, J = 19.6 Hz), 34.7 (dd, J = 5.7, 3.5 Hz); ^{19}F NMR (376 MHz, $CDCl_3$) δ -121.09 (ddd, J = 277.4, 56.8, 17.3 Hz, 1F), -122.70 (ddd, J = 277.4, 56.5, 14.3 Hz, 1F); HRMS (EI): m/z calcd $C_{17}H_{16}F_2O_2$ $[M]^+$ 290.1118, found 290.1124.

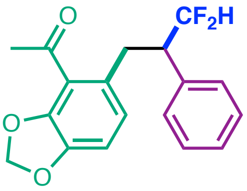

Using general procedure D. **1-(5-(3,3-difluoro-2-phenylpropyl)benzo[d][1,3]dioxol-4-yl)ethan-1-one (68):** 0.25 mmol scale, 42 mg, 53%, colorless oil, 5% EA in Hexane, $R_f = 0.15$.

^{1}H NMR (400 MHz, $CDCl_3$) δ 7.23 – 7.18 (m, 3H), 7.13 (d, J = 7.8 Hz, 2H), 7.09 (s, 1H), 6.38 (s, 1H), 6.09 – 5.78 (m, 3H), 3.46 – 3.24 (m, 2H), 3.11 (dd, J = 12.8, 8.8 Hz, 1H), 2.37 (s, 3H); ^{13}C NMR (101 MHz, $CDCl_3$) δ 199.6, 150.0, 146.1, 136.7 (t, J = 4.0 Hz), 135.8, 130.9, 129.4, 128.6, 127.6, 117.8 (t, J = 244.5 Hz), 112.4, 110.0, 101.9, 51.3 (t, J = 19.2 Hz), 34.6 (t, J = 4.5 Hz), 29.8, 29.5; ^{19}F NMR (376 MHz, $CDCl_3$) δ -120.83 (dd, J = 16.6, 7.3 Hz, 1F), -120.98 (dd, J = 16.3, 7.1 Hz, 1F); HRMS (EI): m/z calcd $C_{18}H_{16}F_2O_3$ $[M]^+$ 318.1068, found 318.1067.

Note: contaminated with 4wt.% isolable aryl difluoromethylation side product, more details see the corresponding ^{19}F NMR spectra.

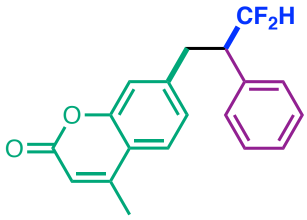

Using general procedure D. **7-(3,3-difluoro-2-phenylpropyl)-4-methyl-2H-chromen-2-one (69):** 0.25 mmol scale, 39 mg, 50%, white solid, 14% EA in Hexane, $R_f = 0.12$.

^{1}H NMR (400 MHz, $CDCl_3$) δ 7.41 (d, J = 8.5 Hz, 1H), 7.29 (d, J = 7.2 Hz, 3H), 7.18 (d, J = 7.2 Hz, 2H), 7.01 – 6.94 (m, 2H), 6.21 (s, 1H), 5.94 (td, J = 56.4, 3.0 Hz, 1H), 3.41 – 3.25 (m, 2H), 3.12 (dd, J = 13.2, 10.1 Hz, 1H), 2.37 (s, 3H); ^{13}C NMR (101 MHz, $CDCl_3$) δ 161.0, 153.5, 152.3, 143.4, 135.6 (dd, J = 5.0, 2.5 Hz), 129.0, 128.9, 128.1, 125.2, 124.6, 118.4, 117.5,

117.3 (t, J = 245.5 Hz), 114.6, 51.6 (t, J = 19.8 Hz), 34.8 (dd, J = 5.3, 3.7 Hz), 18.7; ^{19}F NMR (376 MHz, $CDCl_3$) δ -118.77 (ddd, J = 277.6, 56.2, 13.8 Hz, 1F), -123.31 (ddd, J = 277.1, 56.4, 16.8 Hz, 1F); MS (EI): m/z calcd $C_{19}H_{16}F_2O_2$ $[M]^+$ 314.1, found 314.1; HRMS (EI): m/z calcd $C_{19}H_{15}FO_2$ $[M-HF]^+$ 294.1056, found 294.1051.

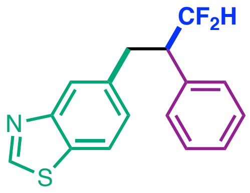

Using general procedure D. **5-(3,3-difluoro-2-phenylpropyl)benzo[d]thiazole (70):** 0.25 mmol scale, 26 mg, 36%, colorless oil, 8% EA in Hexane, R_f = 0.20.

^{1}H NMR (400 MHz, $CDCl_3$) δ 8.96 (s, 1H), 7.90 – 7.74 (m, 2H), 7.33 – 7.22 (m, 5H), 7.16 (d, J = 8.2 Hz, 1H), 5.98 (td, J = 56.2, 2.5 Hz, 1H), 3.53 – 3.33 (m, 2H), 3.23 (dd, J = 13.1, 9.5 Hz, 1H); ^{13}C NMR (101 MHz, $CDCl_3$) δ 154.4, 153.7, 137.0, 136.1 (dd, J = 4.5, 2.8 Hz), 131.9, 129.2, 128.8, 127.9, 127.0, 123.9, 121.7, 117.4 (t, J = 244.3 Hz), 52.1 (t, J = 19.8 Hz), 35.1 (dd, J = 5.5, 3.5 Hz); ^{19}F NMR (376 MHz, $CDCl_3$) δ -119.70 (ddd, J = 277.8, 56.4, 15.3 Hz, 1F), -122.92 (ddd, J = 278.1, 56.4, 15.6 Hz, 1F); HRMS (EI): m/z calcd $C_{16}H_{13}F_2NS$ $[M]^+$ 289.0737, found 289.0740.

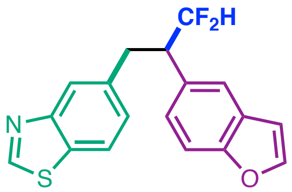

Using general procedure D. **5-(2-(benzofuran-5-yl)-3,3-difluoropropyl)benzo[d]thiazole (71):** 0.25 mmol scale, 35 mg, 42%, colorless oil, 15% EA in Hexane, R_f = 0.25.

^{1}H NMR (400 MHz, $CDCl_3$) δ 8.93 (s, 1H), 7.84 (s, 1H), 7.76 (d, J = 8.3 Hz, 1H), 7.62 – 7.57 (m, 1H), 7.48 – 7.39 (m, 2H), 7.14 (dd, J = 8.0, 3.5 Hz, 2H), 6.70 (s, 1H), 5.98 (td, J = 56.8, 2.9 Hz, 1H), 3.55. – 3.40 (m, 2H), 3.30 – 3.20 (m, 1H); ^{13}C NMR (101 MHz, $CDCl_3$) δ 154.6, 154.4, 153.7, 145.6, 137.1, 131.8, 130.5 (dd, J = 4.8, 2.8 Hz), 127.9, 127.0, 125.3, 123.9, 121.8, 121.7, 117.6 (t, J = 244.7 Hz), 111.6, 106.7, 52.0 (t, J = 19.7 Hz), 35.4 (dd, J = 5.4, 3.3 Hz); ^{19}F NMR (376 MHz, $CDCl_3$) δ -119.62 (ddd, J = 277.3, 56.4, 15.0 Hz, 1F), -122.98 (ddd, J = 277.6, 56.4, 15.7 Hz, 1F); HRMS (ESI): m/z calcd $C_{18}H_{13}F_2ONSNa$ $[M+Na]^+$ 352.0578, found 352.0579.

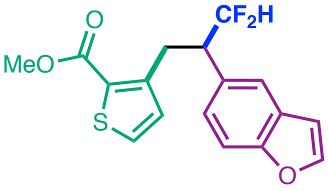

Using general procedure D. **Methyl-3-(2-(benzofuran-5-yl)-3,3-difluoropropyl)thiophene-2-carboxylate (72)**: 0.25 mmol scale, 30 mg, 36%, colorless oil, 2% EA in Hexane, R_f = 0.15.

^{1}H NMR (400 MHz, $CDCl_3$) δ 7.60 (d, J = 2.1 Hz, 1H), 7.50 – 7.39 (m, 2H), 7.25 (d, J = 5.1 Hz, 1H), 7.16 (dd, J = 8.5, 1.4 Hz, 1H), 6.72 (d, J = 1.3 Hz, 1H), 6.64 (d, J = 5.1 Hz, 1H), 5.98 (td, J = 56.4, 3.2 Hz, 1H), 3.87 (s, 3H), 3.72 (dd, J = 13.2, 5.3 Hz, 1H), 3.62 – 3.40 (m, 2H); ^{13}C NMR (101 MHz, $CDCl_3$) δ 163.0, 154.5, 147.0, 145.5, 131.3, 130.9 (t, J = 4.0 Hz), 130.4, 127.8, 127.5, 125.3, 121.7, 117.7 (t, J = 244.4 Hz), 111.4, 106.7, 52.1, 50.4 (t, J = 19.6 Hz), 29.7 (dd, J = 5.8, 3.6 Hz); ^{19}F NMR (376 MHz, $CDCl_3$) δ -120.02 (ddd, J = 276.8, 56.3, 16.8 Hz, 1F), -121.96 (ddd, J = 277.1, 56.4, 14.6 Hz, 1F); MS (EI): m/z calcd $C_{17}H_{14}F_2O_3S$ $[M]^+$ 336.1, found 336.1; HRMS (EI): m/z calcd $C_{17}H_{13}FO_3S$ $[M-HF]^+$ 316.0569, found 316.0572.

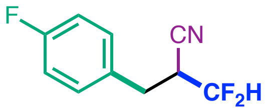

Using general procedure D. **3,3-difluoro-2-(4-fluorobenzyl)propanenitrile (73):** 0.25 mmol scale, 28 mg, 56%, colorless oil, 5% EA in Hexane, $R_f = 0.15$.

^{1}H NMR (400 MHz, $CDCl_3$) δ 7.19 (t, J = 6.8 Hz, 2H), 7.00 (t, J = 8.6 Hz, 2H), 5.79 (td, J = 54.9, 3.8 Hz, 1H), 3.16 (dtt, J = 14.6, 9.4, 5.2 Hz, 1H), 2.99 (qd, J = 14.0, 7.4 Hz, 2H); ^{13}C NMR (101 MHz, $CDCl_3$) δ 162.6 (d, J = 247.0 Hz), 130.8 (d, J = 8.2 Hz), 130.3 (d, J = 3.4 Hz), 116.3 (d, J = 21.6 Hz), 115.4 (t, J = 5.8 Hz), 112.6 (t, J = 247.7 Hz), 39.3 (t, J = 22.9 Hz), 31.5 (t, J = 3.5 Hz); ^{19}F NMR (376 MHz, $CDCl_3$) δ -114.02 (tt, J = 9.1, 5.1 Hz, 1F), -119.94 (ddd, J = 284.4, 54.9, 11.0 Hz, 1F), -121.01 (ddd, J = 284.3, 55.0, 13.6 Hz, 1F); HRMS (EI): m/z calcd $C_{10}H_8F_3N$ $[M]^+$ 199.0609, found 199.0606.

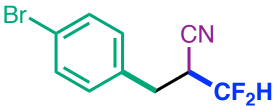

Using general procedure D. **2-(4-bromobenzyl)-3,3-difluoropropanenitrile (74):** 0.25 mmol scale, 31 mg, 48%, colorless oil, 8% EA in Hexane, $R_f = 0.20$.

^{1}H NMR (400 MHz, $CDCl_3$) δ 7.51 (d, J = 7.3 Hz, 2H), 7.17 (d, J = 7.5 Hz, 2H), 5.86 (td, J = 54.9, 2.7 Hz, 1H), 3.30 – 3.15 (m, 1H), 3.12 – 2.92 (m, 2H); ^{13}C NMR (101 MHz, $CDCl_3$) δ 133.5, 132.5, 130.9, 122.3, 115.3 (t, J = 5.7 Hz), 112.5 (t, J = 248.6 Hz), 39.1 (t, J = 23.6 Hz), 31.7 (t, J = 3.6 Hz); ^{19}F NMR (376 MHz, $CDCl_3$) δ -119.89 (ddd, J = 284.4, 54.8, 11.2 Hz, 1F), -120.91 (ddd, J = 284.0, 55.0, 13.2 Hz, 1F); HRMS (EI): m/z calcd $C_{10}H_8BrF_2N$ $[M]^+$ 258.9808, found 258.9808.

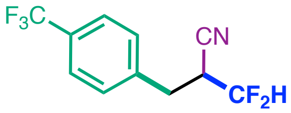

Using general procedure D. **3,3-difluoro-2-(4-(trifluoromethyl)benzyl)propanenitrile (75):** 0.25 mmol scale, 29 mg, 46%, white solid, 5% EA in Hexane, R_f = 0.13.

^{1}H NMR (400 MHz, $CDCl_3$) δ 7.65 (d, J = 8.0 Hz, 2H), 7.43 (d, J = 8.0 Hz, 2H), 6.06 – 5.74 (m, 1H), 3.40 – 3.21 (m, 1H), 3.14 (qd, J = 13.9, 7.5 Hz, 2H); ^{13}C NMR (101 MHz, $CDCl_3$) δ 138.6, 130.6 (q, J = 32.5 Hz), 129.6, 126.3 (q, J = 3.8 Hz), 124.0 (q, J = 272.3 Hz), 115.1 (t, J = 5.9 Hz), 112.5 (t, J = 248.0 Hz), 38.9 (t, J = 23.9 Hz), 31.9 (t, J = 3.6 Hz); ^{19}F NMR (376 MHz, $CDCl_3$) δ -62.71 (s, 1F), -118.34 – -122.02 (m, 2F); HRMS (EI): m/z calcd $C_{11}H_8F_5N$ $[M]^+$ 249.0577, found 249.0575.

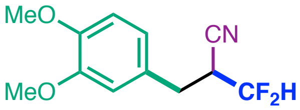

Using general procedure D. **2-(3,4-dimethoxybenzyl)-3,3-difluoropropanenitrile (76):** 0.25 mmol scale, 40 mg, 66%, colorless oil, 20% EA in Hexane, R_f = 0.12.

^{1}H NMR (400 MHz, $CDCl_3$) δ 6.84 (t, J = 6.5 Hz, 2H), 6.78 (s, 1H), 5.83 (td, J = 55.0, 3.8 Hz, 1H), 3.89 (s, 3H), 3.87 (s, 3H), 3.25 (ddp, J = 14.4, 10.0, 5.6, 5.0 Hz, 1H), 3.08 – 2.95 (m, 2H); ^{13}C NMR (101 MHz, $CDCl_3$) δ 149.4, 148.8, 126.9, 121.4, 115.7 (t, J = 5.5 Hz), 112.7 (t, J = 247.5 Hz), 112.1, 111.7, 56.1, 56.0, 39.4 (t, J = 22.6 Hz), 32.1 (t, J = 3.6 Hz); ^{19}F NMR (376 MHz, $CDCl_3$) δ -119.94 (ddd, J = 284.2, 54.9, 10.5 Hz, 1F), -121.40 (ddd, J = 284.0, 55.1, 14.4 Hz, 1F); HRMS (EI): m/z calcd $C_{12}H_{13}F_2NO_2$ $[M]^+$ 241.0914, found 241.0913.

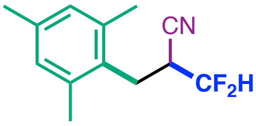

Using general procedure D. **3,3-difluoro-2-(2,4,6-trimethylbenzyl)propanenitrile (77):** 0.25 mmol scale, 23 mg, 41%, colorless oil, 10% EA in Hexane, R_f = 0.10.

^{1}H NMR (400 MHz, $CDCl_3$) δ 6.90 (s, 2H), 6.06 – 5.74 (m, 1H), 3.26 – 3.15 (m, 2H), 3.15 – 3.00 (m, 1H), 2.35 (s, 6H), 2.27 (s, 3H); ^{13}C NMR (101 MHz, $CDCl_3$) δ 137.4, 136.7, 129.9, 128.6, 115.8 (t, J = 5.5 Hz), 112.9 (t, J = 247.7 Hz), 110.5, 37.1 (t, J = 22.8 Hz), 25.3 (t, J = 3.3 Hz), 21.0, 20.0; ^{19}F NMR (376 MHz, $CDCl_3$) δ -120.02 (dd, J = 55.2, 12.6 Hz, 2F); HRMS (EI): m/z calcd $C_{13}H_{15}F_2N$ $[M]^+$ 223.1173, found 223.1172.

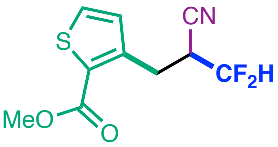

Using general procedure D. **2-((4-acetylbenzo[d][1,3]dioxol-5-yl)methyl)-3,3-difluoropropanenitrile (78):** 0.25 mmol scale, 21 mg, 34%, colorless oil, 10% EA in Hexane, R_f = 0.10.

^{1}H NMR (400 MHz, $CDCl_3$) δ 7.53 (d, J = 5.0 Hz, 1H), 7.11 (d, J = 5.0 Hz, 1H), 5.95 (td, J = 55.0, 2.8 Hz, 1H), 3.89 (s, 3H), 3.72 – 3.50 (m, 2H), 3.32 – 3.22 (m, 1H); ^{13}C NMR (101 MHz, $CDCl_3$) δ 162.8, 143.0, 131.7, 131.4, 128.5, 115.6 (t, J = 4.9 Hz), 112.9 (t, J = 247.9 Hz), 52.4, 37.7 (t, J = 22.2 Hz), 26.4 (dd, J = 4.8, 3.0 Hz); ^{19}F NMR (376 MHz, $CDCl_3$) δ -118.49 (ddd, J = 283.2, 54.9, 9.6 Hz, 1F), -121.90 (ddd, J = 282.6, 54.4, 17.4 Hz, 1F); HRMS (EI): m/z calcd $C_{10}H_9F_2NO_2S$ $[M]^+$ 245.0322, found 245.0324.

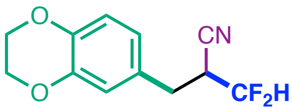

Using general procedure D. **2-((2,3-dihydrobenzo[b][1,4]dioxin-6-yl)methyl)-3,3-difluoropropanenitrile (79):** 0.25 mmol scale,45 mg, 75%, colorless oil, 15% EA in Hexane, R_f = 0.21.

^{1}H NMR (400 MHz, $CDCl_3$) δ 6.85 (d, J = 8.2 Hz, 1H), 6.79 – 6.71 (m, 2H), 5.82 (td, J = 54.9, 3.8 Hz, 1H), 4.25 (s, 4H), 3.22 (tdd, J = 15.0, 6.3, 3.8 Hz, 1H), 2.98 (h, J = 5.8, 5.4 Hz, 2H); ^{13}C NMR (101 MHz, $CDCl_3$) δ 144.0, 143.5, 127.6, 122.0, 118.0, 117.9, 115.6 (t, J = 5.5 Hz), 112.7 (t, J = 247.4 Hz), 64.5, 64.4, 39.3 (t, J = 22.8 Hz), 31.8 (t, J = 3.6 Hz); ^{19}F NMR (376 MHz, $CDCl_3$) δ -119.91 (ddd, J = 283.6, 55.0, 10.2 Hz, 1F), -121.64 (ddd, J = 283.5, 55.1, 15.0 Hz, 1F); HRMS (EI): m/z calcd $C_{12}H_{11}F_2NO_2$ $[M]^+$ 239.0758, found 239.0761.

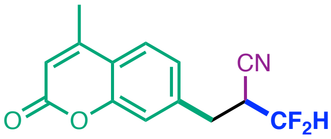

Using general procedure D. **3,3-difluoro-2-((4-methyl-2-oxo-2H-chromen-7-yl)methyl)propanenitrile (80):** 0.25 mmol scale, 21 mg, 32%, white solid, 20% EA in Hexane, R_f = 0.12.

^{1}H NMR (400 MHz, $CDCl_3$) δ 7.65 (d, J = 8.4 Hz, 1H), 7.28 (d, J = 5.7 Hz, 2H), 6.33 (s, 1H), 5.96 (td, J = 54.8, 3.3 Hz, 1H), 3.44 – 3.30 (m, 1H), 3.29 – 3.09 (m, 2H), 2.47 (s, 3H); ^{13}C NMR (101 MHz, $CDCl_3$) δ 159.5, 152.9, 151.0, 137.9, 124.6, 124.1, 118.8, 116.6, 114.5, 114.1 (t, J = 6.0 Hz), 111.5 (t, J = 248.4 Hz), 37.9 (t, J = 23.8 Hz), 30.9 (t, J = 3.5 Hz), 17.8; ^{19}F NMR (376 MHz, $CDCl_3$) δ -118.96 – -121.34 (m, 2F); HRMS (EI): m/z calcd $C_{14}H_{11}F_2NO_2$ $[M]^+$ 263.0758, found 263.0756.

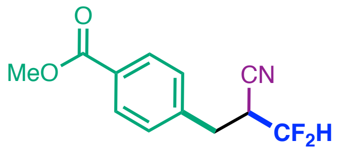

Using general procedure D. **Methyl-4-(2-cyano-3,3-difluoropropyl)benzoate (81):** 0.25 mmol scale, 29.5 mg, 43%, colorless oil, 10% EA in Hexane, R_f = 0.15.

^{1}H NMR (400 MHz, $CDCl_3$) δ 8.04 (d, J = 8.3 Hz, 2H), 7.36 (d, J = 8.2 Hz, 2H), 5.88 (td, J = 54.8, 3.7 Hz, 1H), 3.92 (s, 3H), 3.36 – 3.24 (m, 1H), 3.12 (qd, J = 13.9, 7.5 Hz, 2H); ^{13}C NMR (101 MHz, $CDCl_3$) δ 166.7, 139.7, 130.5, 130.1, 129.2, 115.2 (t, J = 5.6 Hz), 112.50 (t, J = 247.9 Hz), 52.4, 38.9 (t, J = 23.6 Hz), 32.1 (t, J = 3.5 Hz); ^{19}F NMR (376 MHz, $CDCl_3$) δ -119.80 (ddd, J = 284.1, 54.8, 11.3 Hz, 1F), -120.90 (ddd, J = 284.1, 54.9, 13.5 Hz, 1F); HRMS (EI): m/z calcd $C_{13}H_{16}F_2O_4$ $[M]^+$ 239.0758, found 239.0759.

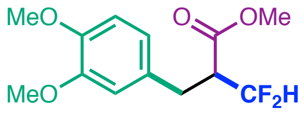

Using general procedure D. **Methyl-2-(3,4-dimethoxybenzyl)-3,3-difluoropropanoate (82):** 0.25 mmol scale, 29.5 mg, 43%, colorless oil, 10% EA in Hexane, R_f = 0.10.

^{1}H NMR (400 MHz, $CDCl_3$) δ 6.78 (d, J = 8.1 Hz, 1H), 6.72 – 6.67 (m, 2H), 5.95 (td, J = 55.7, 5.6 Hz, 1H), 3.86 (s, 6H), 3.65 (s, 3H), 3.12 (ddt, J = 17.7, 11.7, 6.6 Hz, 1H), 3.00 (d, J = 7.2 Hz, 2H); ^{13}C NMR (101 MHz, $CDCl_3$) δ 170.0 (dd, J = 9.0, 3.7 Hz), 149.0, 148.1, 129.4, 121.0, 115.7 (t, J = 244.1 Hz), 112.0, 111.3, 56.0 (t, J = 4.0 Hz), 52.4, 52.3 (t, J = 21.8 Hz), 36.1, 32.0 (dd, J = 5.7, 3.5 Hz); ^{19}F NMR (376 MHz, $CDCl_3$) δ -119.07 (ddd, J = 284.6, 55.2, 11.2 Hz, 1F), -123.38 (ddd, J = 284.7, 56.1, 12.4 Hz, 1F); HRMS (EI): m/z calcd $C_{13}H_{16}F_2O_4$ $[M]^+$ 274.1017, found 274.1019.

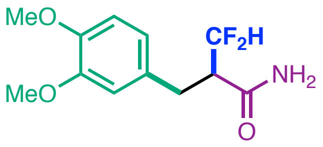

Using general procedure D. **2-(3,4-dimethoxybenzyl)-3,3-difluoropropanamide (83):** 0.2 mmol scale, 41.5 mg, 80%, white solid, 50% EA in Hexane, R_f = 0.12.

^{1}H NMR (400 MHz, DMSO-d_6) δ 7.50 (s, 1H), 7.11 (s, 1H), 6.86 – 6.78 (m, 2H), 6.69 (dd, J = 8.1, 1.8 Hz, 1H), 6.05 (td, J = 56.2, 6.0 Hz, 1H), 3.72 (s, 3H), 3.70 (s, 3H), 3.00 (qd, J = 11.3, 6.0 Hz, 1H), 3.00 – 2.81 (m, 1H), 2.74 (dd, J = 13.5, 3.9 Hz, 1H); ^{13}C NMR (101 MHz, DMSO-d_6) δ 169.9, 148.5, 147.4, 130.3, 120.8, 117.4 (t, J = 241.8 Hz), 112.6, 111.6, 55.5, 55.4, 51.5 (t, J = 19.6 Hz), 31.4 (dd, J = 6.3, 2.7 Hz); ^{19}F NMR (376 MHz, DMSO-d_6) δ -117.58 (ddd, J = 278.5, 56.0, 12.0 Hz, 1F), -122.10 (ddd, J = 278.1, 56.3, 11.8 Hz, 1F); HRMS (ESI): m/z calcd $C_{12}H_{15}F_2NNaO_3$ $[M+Na]^+$ 282.0912, found 282.0915.

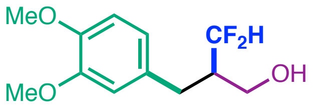

Using general procedure D. **2-(3,4-dimethoxybenzyl)-3,3-difluoropropan-1-ol (84):** 0.2 mmol scale, 42 mg, 85%, white solid, 40% EA in Hexane, R_f = 0.18.

^{1}H NMR (400 MHz, $CDCl_3$) δ 6.80 (d, J = 8.0 Hz, 1H), 6.77 – 6.64 (m, 2H), 5.91 (td, J = 56.4, 3.7 Hz, 1H), 3.87 (s, 3H), 3.86 (s, 3H), 3.77 – 3.64 (m, 2H), 2.85 (dd, J = 14.0, 5.7 Hz, 1H), 2.67 (dd, J = 14.0, 9.4 Hz, 1H), 2.24 (tdp, J = 15.2, 9.8, 5.8, 5.1 Hz, 1H); ^{13}C NMR (101 MHz, $CDCl_3$) δ 149.2, 147.8, 130.9, 121.2, 117.8 (t, J = 241.6 Hz), 112.2, 111.4, 59.8 (t, J = 5.1 Hz), 56.1, 56.0, 46.9 (t, J = 18.4 Hz), 30.4 (t, J = 4.8 Hz); ^{19}F NMR (376 MHz, $CDCl_3$) δ -124.66

(ddd, J = 282.4, 56.3, 14.9 Hz, 1F), -125.75 (ddd, J = 282.6, 56.5, 15.2 Hz, 1F); HRMS (EI): m/z calcd $C_{12}H_{16}F_2O_3$ $[M]^+$ 246.1068, found 246.1076.

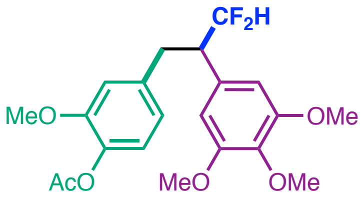

Using general procedure D. **4-(3,3-difluoro-2-(3,4,5-trimethoxyphenyl)propyl)-2-methoxyphenyl acetate (85):** 0.25 mmol scale, 46 mg, 45%, colorless oil, 15% EA in Hexane, R_f = 0.12.

^{1}H NMR (400 MHz, $CDCl_3$) δ 6.91 – 6.69 (m, 3H), 6.36 (s, 2H), 5.90 (td, J = 56.5, 3.1 Hz, 1H), 3.83 (s, 3H), 3.80 (s, 6H), 3.77 (s, 3H), 3.19 – 3.08 (m, 2H), 2.98 – 2.80 (m, 1H), 2.28 (s, 3H); ^{13}C NMR (101 MHz, $CDCl_3$) δ 169.1, 153.3, 149.75, 139.6, 137.4, 132.0 (dd, J = 4.7, 2.4 Hz), 130.9, 127.5, 123.4, 117.2 (t, J = 244.4 Hz), 112.3, 106.0, 60.9, 56.2, 56.0, 52.0 (t, J = 19.7 Hz), 34.6 (dd, J = 5.4, 3.1 Hz), 20.8; ^{19}F NMR (376 MHz, $CDCl_3$) δ -120.02 (ddd, J = 278.0, 56.2, 15.6 Hz, 1F), -122.99 (ddd, J = 278.0, 56.4, 15.0 Hz, 1F); HRMS (EI): m/z calcd $C_{12}H_{16}F_2O_3$ $[M]^+$ 410.1541, found 410.1548.

X. Crystallographic data for radical-relayed difluoromethylation product 27

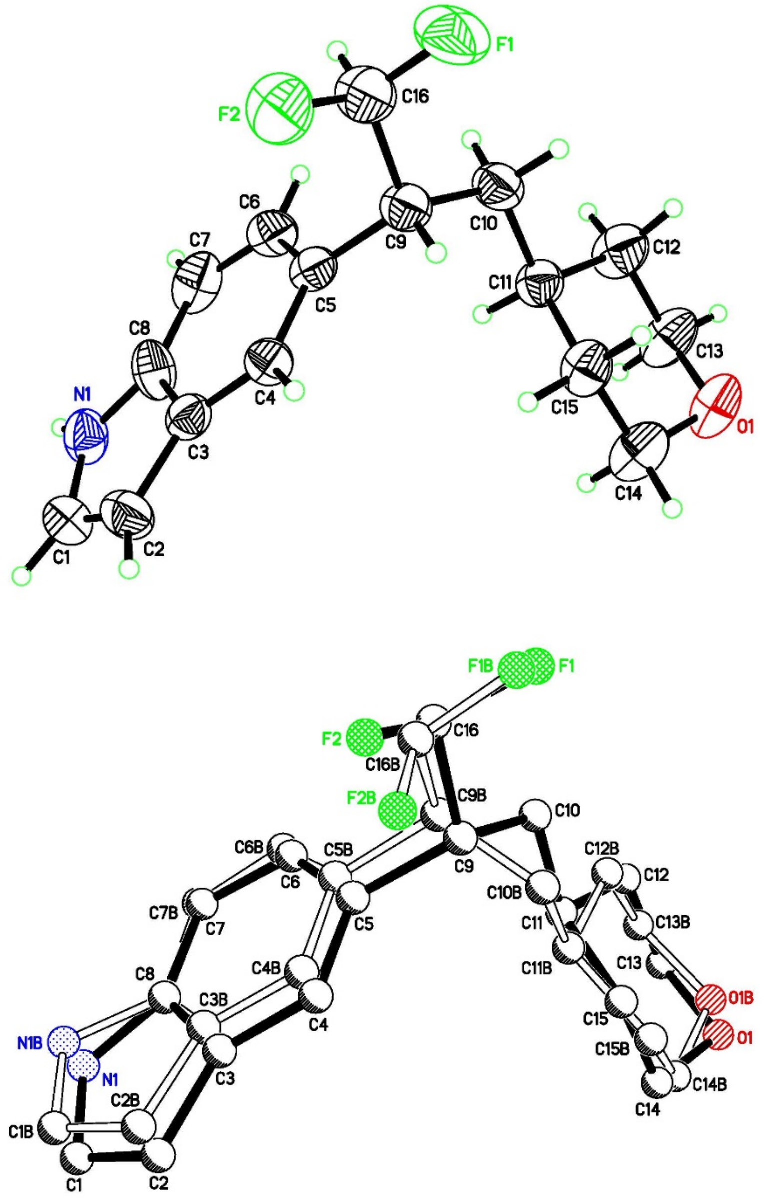

Supplementary Fig. 15. ORTEP drawing of compound 27 (CCDC-2033193). The major component and also the relationship of the minor component: (top) Major component (75% occupancy) of 5-(1,1-difluoro-3-(tetrahydro-2H-pyran-4-yl)propan-2-yl)-1H-indole (27) drawn with 50% anisotropic displacement ellipsoids. (bottom) Relationship of the major (black bonds) and minor (white bonds) components. Further details are provided in CCDC-2033193.

Supplementary Table 4. Crystal data and structure refinement for compound 27.

Empirical formula	$C_{16}H_{19}NOF_2$	
Formula weight	279.32	
Temperature	150(2) K	
Wavelength	1.54178 Å	
Crystal system	Orthorhombic	
Space group	Pbca	
Unit cell dimensions	a = 17.2539(6) Å	α = 90°
	b = 9.2536(3) Å	β = 90°
	c = 18.1714(7) Å	γ = 90°
Volume	2901.26(18) $Å^3$	
Z	8	
Density (calculated)	1.279 Mg/m^3	
Absorption coefficient	0.806 mm^{-1}	
F(000)	1184	
Crystal size	0.178 x 0.068 x 0.020 mm^3	
θ range for data collection	4.867 to 72.538°	
Index ranges	$-21 \leq h \leq 21$, $-9 \leq k \leq 11$, $-22 \leq l \leq 22$	
Reflections collected	40792	
Independent reflections	2872 [R(int) = 0.0571]	
Completeness to θ = 67.679°	100.0 %	
Absorption correction	Semi-empirical from equivalents	
Max. and min. transmission	0.865 and 0.835	
Refinement method	Full-matrix least-squares on F^2	
Data / restraints / parameters	2872 / 706 / 362	
Goodness-of-fit on F^2	1.205	
Final R indices [I>2σ(I)]	R1 = 0.0603, wR2 = 0.1372	
R indices (all data)	R1 = 0.0635, wR2 = 0.1387	
Largest diff. peak and hole	0.154 and -0.202 $eÅ^{-3}$	

Atomic coordinates [x10^4] and equivalent isotropic displacement parameters [Å^{2}x10^3] for compound 27. U(eq) is defined as one third of the trace of the orthogonalized U_{ij} tensor.

	x	y	z	U(eq)
F(1)	6124(4)	5926(9)	5609(6)	71(1)
F(2)	5744(2)	4111(3)	6271(2)	69(1)
O(1)	2989(3)	10057(6)	5064(3)	55(1)
N(1)	3027(3)	4097(7)	8527(3)	47(1)
C(1)	2597(3)	3070(6)	8173(3)	51(1)
C(2)	2815(3)	2981(7)	7458(4)	47(1)
C(3)	3416(4)	4026(8)	7342(3)	36(1)
C(4)	3869(3)	4466(6)	6738(3)	34(1)
C(5)	4403(4)	5565(7)	6808(3)	34(1)
C(6)	4515(5)	6192(9)	7506(4)	40(1)
C(7)	4103(6)	5761(10)	8113(4)	51(2)
C(8)	3569(6)	4652(9)	8028(4)	46(2)
C(9)	4874(3)	6069(5)	6151(3)	40(1)
C(10)	4862(2)	7716(3)	6032(2)	46(1)
C(11)	4055(2)	8375(5)	5964(2)	40(1)
C(12)	4121(2)	10016(5)	5852(2)	55(1)
C(13)	3351(3)	10703(4)	5679(3)	58(1)
C(14)	2831(4)	8567(6)	5214(4)	57(2)
C(15)	3582(3)	7739(4)	5335(3)	45(1)
C(16)	5712(4)	5593(6)	6229(5)	53(1)
F(1B)	6114(16)	5640(30)	5670(19)	102(8)
F(2B)	5348(7)	3824(9)	5954(5)	86(3)
O(1B)	3209(11)	10317(18)	5189(10)	63(4)
N(1B)	3205(11)	3990(20)	8646(10)	80(6)
C(1B)	2837(9)	2890(20)	8276(11)	65(5)
C(2B)	3026(11)	2860(20)	7559(11)	65(6)
C(3B)	3566(13)	4020(30)	7465(11)	49(6)
C(4B)	3993(14)	4590(30)	6886(11)	64(6)
C(5B)	4502(13)	5700(30)	6990(9)	42(5)
C(6B)	4513(19)	6430(30)	7658(12)	44(5)
C(7B)	4063(15)	5960(30)	8223(12)	37(4)
C(8B)	3526(13)	4850(30)	8103(11)	36(4)
C(9B)	4998(10)	6180(20)	6353(8)	57(5)
C(10B)	4549(5)	6509(10)	5655(5)	51(3)
C(11B)	3921(6)	7691(13)	5736(7)	42(2)
C(12B)	4278(7)	9188(17)	5813(7)	63(3)
C(13B)	3700(10)	10368(15)	5807(8)	70(4)
C(14B)	2817(10)	8990(20)	5165(16)	71(6)
C(15B)	3351(9)	7705(18)	5096(10)	67(4)
C(16B)	5643(11)	5109(19)	6214(13)	68(6)
H(1)	2973	4363	8989	56
H(1A)	2202	2502	8395	61
H(2A)	2608	2343	7099	56
H(4A)	3806	3999	6277	41
H(6A)	4889	6938	7558	48

H(7A)	4181	6209	8577	61
H(9A)	4656	5601	5700	48
H(10A)	5132	8183	6450	55
H(10B)	5157	7942	5580	55
H(11A)	3769	8196	6434	47
H(12A)	4336	10460	6304	65
H(12B)	4486	10212	5444	65
H(13A)	3006	10612	6113	70
H(13B)	3429	11745	5581	70
H(14A)	2545	8139	4795	69
H(14B)	2501	8490	5657	69
H(15A)	3460	6717	5447	54
H(15B)	3894	7762	4878	54
H(16A)	5955	6042	6673	64
H(2)	3231	4111	9125	96
H(1B)	2490	2224	8501	78
H(2B)	2842	2206	7194	78
H(4B)	3930	4192	6407	77
H(6B)	4833	7259	7719	53
H(7B)	4114	6376	8698	44
H(9B)	5251	7100	6508	68
H(10C)	4297	5609	5483	61
H(10D)	4920	6815	5271	61
H(11B)	3622	7485	6196	51
H(12C)	4574	9231	6279	76
H(12D)	4648	9342	5404	76
H(13C)	3382	10308	6259	84
H(13D)	3975	11308	5813	84
H(14C)	2455	8996	4741	85
H(14D)	2504	8884	5618	85
H(15C)	3638	7759	4625	81
H(15D)	3043	6802	5097	81
H(16B)	5947	4942	6675	82

Supplementary Table 5. Bond lengths [Å] and angles [°] for compound 27.

F(1)-C(16)	1.367(7)	F(2)-C(16)	1.375(5)
O(1)-C(13)	1.413(5)	O(1)-C(14)	1.432(7)
N(1)-C(1)	1.367(6)	N(1)-C(8)	1.399(6)
C(1)-C(2)	1.355(6)	C(2)-C(3)	1.433(6)
C(3)-C(8)	1.399(7)	C(3)-C(4)	1.408(5)
C(4)-C(5)	1.378(6)	C(5)-C(6)	1.408(7)
C(5)-C(9)	1.518(5)	C(6)-C(7)	1.371(7)
C(7)-C(8)	1.388(7)	C(9)-C(16)	1.518(7)
C(9)-C(10)	1.539(5)	C(10)-C(11)	1.525(4)
C(11)-C(15)	1.522(5)	C(11)-C(12)	1.536(5)
C(12)-C(13)	1.506(5)	C(14)-C(15)	1.522(7)
F(1B)-C(16B)	1.371(16)	F(2B)-C(16B)	1.378(16)
O(1B)-C(14B)	1.403(15)	O(1B)-C(13B)	1.408(14)
N(1B)-C(1B)	1.374(15)	N(1B)-C(8B)	1.388(15)
C(1B)-C(2B)	1.343(15)	C(2B)-C(3B)	1.436(15)
C(3B)-C(4B)	1.386(15)	C(3B)-C(8B)	1.393(16)
C(4B)-C(5B)	1.367(15)	C(5B)-C(6B)	1.390(15)
C(5B)-C(9B)	1.504(14)	C(6B)-C(7B)	1.361(15)
C(7B)-C(8B)	1.397(14)	C(9B)-C(16B)	1.510(15)
C(9B)-C(10B)	1.518(14)	C(10B)-C(11B)	1.546(11)
C(11B)-C(12B)	1.522(13)	C(11B)-C(15B)	1.525(12)
C(12B)-C(13B)	1.479(14)	C(14B)-C(15B)	1.510(16)
C(13)-O(1)-C(14)	109.9(5)	C(1)-N(1)-C(8)	108.3(4)
C(2)-C(1)-N(1)	110.0(4)	C(1)-C(2)-C(3)	107.5(5)
C(8)-C(3)-C(4)	118.1(5)	C(8)-C(3)-C(2)	106.5(4)
C(4)-C(3)-C(2)	135.3(5)	C(5)-C(4)-C(3)	120.9(5)
C(4)-C(5)-C(6)	118.6(4)	C(4)-C(5)-C(9)	120.9(5)
C(6)-C(5)-C(9)	120.6(5)	C(7)-C(6)-C(5)	122.2(5)
C(6)-C(7)-C(8)	118.1(6)	C(7)-C(8)-N(1)	130.1(6)
C(7)-C(8)-C(3)	121.9(5)	N(1)-C(8)-C(3)	107.4(5)
C(16)-C(9)-C(5)	110.3(5)	C(16)-C(9)-C(10)	108.3(4)
C(5)-C(9)-C(10)	114.0(4)	C(11)-C(10)-C(9)	114.8(3)
C(15)-C(11)-C(10)	113.3(4)	C(15)-C(11)-C(12)	108.8(3)
C(10)-C(11)-C(12)	109.8(4)	C(13)-C(12)-C(11)	112.3(3)
O(1)-C(13)-C(12)	112.1(4)	O(1)-C(14)-C(15)	110.5(5)
C(11)-C(15)-C(14)	111.7(3)	F(1)-C(16)-F(2)	104.4(6)
F(1)-C(16)-C(9)	110.6(6)	F(2)-C(16)-C(9)	109.5(5)
C(14B)-O(1B)-C(13B)	110.2(15)	C(1B)-N(1B)-C(8B)	105.4(13)
C(2B)-C(1B)-N(1B)	112.2(14)	C(1B)-C(2B)-C(3B)	104.9(14)
C(4B)-C(3B)-C(8B)	116.7(15)	C(4B)-C(3B)-C(2B)	135.9(16)
C(8B)-C(3B)-C(2B)	106.5(13)	C(5B)-C(4B)-C(3B)	121.4(15)
C(4B)-C(5B)-C(6B)	119.8(15)	C(4B)-C(5B)-C(9B)	118.7(15)
C(6B)-C(5B)-C(9B)	121.3(15)	C(7B)-C(6B)-C(5B)	119.6(17)
C(6B)-C(7B)-C(8B)	119.8(17)	N(1B)-C(8B)-C(3B)	106.9(14)
N(1B)-C(8B)-C(7B)	125.3(16)	C(3B)-C(8B)-C(7B)	120.1(16)
C(5B)-C(9B)-C(16B)	110.9(15)	C(5B)-C(9B)-C(10B)	114.3(13)
C(16B)-C(9B)-C(10B)	111.7(14)	C(9B)-C(10B)-C(11B)	114.9(10)
C(12B)-C(11B)-C(15B)	108.9(10)	C(12B)-C(11B)-C(10B)	111.7(10)
C(15B)-C(11B)-C(10B)	112.7(12)	C(13B)-C(12B)-C(11B)	113.5(10)
O(1B)-C(13B)-C(12B)	112.7(12)	O(1B)-C(14B)-C(15B)	113.4(15)
C(14B)-C(15B)-C(11B)	109.7(13)	F(1B)-C(16B)-F(2B)	106.3(18)
F(1B)-C(16B)-C(9B)	108.9(17)	F(2B)-C(16B)-C(9B)	110.4(14)

Supplementary Table 6. Anisotropic displacement parameters [$Å^2 x10^3$] for compound 27. The anisotropic displacement factor exponent takes the form: $-2\pi^2[h^2a^{*2}U_{11}+...+2hka^*b^*U_{12}]$

	U_{11}	U_{22}	U_{33}	U_{23}	U_{13}	U_{12}
F(1)	43(2)	73(2)	98(3)	10(2)	25(2)	7(1)
F(2)	61(2)	45(1)	102(2)	12(1)	13(1)	21(1)
O(1)	76(3)	46(2)	44(2)	-5(2)	-18(2)	15(2)
N(1)	61(2)	44(2)	35(2)	5(2)	-2(2)	-3(2)
C(1)	47(2)	47(2)	59(3)	10(2)	-4(2)	-10(2)
C(2)	48(3)	44(2)	49(2)	1(2)	-4(2)	-14(2)
C(3)	39(2)	32(2)	37(2)	-1(2)	-9(2)	0(2)
C(4)	38(2)	28(2)	36(2)	-5(2)	-4(2)	-2(1)
C(5)	34(2)	26(2)	43(3)	-3(2)	-8(2)	2(2)
C(6)	42(2)	26(3)	53(4)	-8(2)	-8(2)	-3(2)
C(7)	74(4)	39(4)	40(2)	-10(2)	-15(2)	9(2)
C(8)	65(4)	26(2)	46(3)	1(2)	6(2)	2(2)
C(9)	35(2)	34(2)	51(3)	2(2)	1(2)	2(2)
C(10)	38(2)	39(2)	61(2)	8(1)	2(1)	-2(1)
C(11)	39(2)	36(2)	44(2)	0(2)	-2(1)	2(1)
C(12)	57(2)	35(2)	72(2)	-2(2)	-14(2)	6(2)
C(13)	69(3)	44(2)	62(3)	-11(2)	-21(2)	18(2)
C(14)	65(3)	47(3)	59(3)	-8(2)	-21(2)	10(2)
C(15)	53(3)	35(2)	46(3)	-2(2)	-4(2)	6(2)
C(16)	46(2)	40(2)	74(3)	3(2)	6(2)	5(2)
F(1B)	82(10)	128(18)	95(11)	-8(12)	33(8)	22(10)
F(2B)	100(7)	73(5)	86(6)	-4(4)	15(5)	47(5)
O(1B)	87(10)	43(6)	58(9)	-4(5)	-21(6)	6(6)
N(1B)	130(14)	60(9)	50(7)	9(6)	-21(8)	-22(9)
C(1B)	74(12)	66(9)	54(7)	19(6)	-15(8)	-16(8)
C(2B)	77(12)	61(9)	57(8)	-14(7)	-15(8)	-13(8)
C(3B)	63(11)	37(7)	47(8)	5(6)	-19(7)	-12(7)
C(4B)	79(12)	65(9)	49(9)	-28(7)	-14(8)	6(8)
C(5B)	44(7)	38(7)	42(9)	-15(7)	-10(7)	-2(5)
C(6B)	68(8)	21(8)	42(9)	-9(6)	-11(7)	-2(6)
C(7B)	44(7)	14(5)	52(9)	-3(6)	2(6)	-9(5)
C(8B)	42(7)	26(7)	41(7)	-9(5)	-17(6)	10(6)
C(9B)	49(8)	59(8)	61(9)	-1(7)	-5(6)	1(6)
C(10B)	51(5)	49(5)	52(5)	5(4)	10(4)	11(4)
C(11B)	48(5)	44(6)	35(5)	1(5)	-6(4)	0(5)
C(12B)	71(7)	40(6)	79(7)	-17(6)	-23(6)	-2(7)
C(13B)	74(9)	52(8)	84(8)	-15(6)	-32(8)	15(7)
C(14B)	39(7)	72(13)	102(11)	16(10)	-5(7)	-16(8)
C(15B)	56(8)	79(9)	66(9)	1(7)	-24(6)	-9(7)
C(16B)	57(9)	74(14)	73(9)	-6(11)	3(7)	28(10)

Supplementary Table 7. Torsion angles [°] for compound 27.

C(8)-N(1)-C(1)-C(2)	-2.6(7)	N(1)-C(1)-C(2)-C(3)	-0.7(7)
C(1)-C(2)-C(3)-C(8)	3.7(8)	C(1)-C(2)-C(3)-C(4)	-179.6(8)
C(8)-C(3)-C(4)-C(5)	-5.2(11)	C(2)-C(3)-C(4)-C(5)	178.3(8)
C(3)-C(4)-C(5)-C(6)	3.2(11)	C(3)-C(4)-C(5)-C(9)	-178.1(6)
C(4)-C(5)-C(6)-C(7)	-1.1(13)	C(9)-C(5)-C(6)-C(7)	-179.7(9)
C(5)-C(6)-C(7)-C(8)	1.1(15)	C(6)-C(7)-C(8)-N(1)	-173.4(10)
C(6)-C(7)-C(8)-C(3)	-3.3(16)	C(1)-N(1)-C(8)-C(7)	176.1(10)
C(1)-N(1)-C(8)-C(3)	4.9(9)	C(4)-C(3)-C(8)-C(7)	5.3(14)
C(2)-C(3)-C(8)-C(7)	-177.3(9)	C(4)-C(3)-C(8)-N(1)	177.4(7)
C(2)-C(3)-C(8)-N(1)	-5.2(9)	C(4)-C(5)-C(9)-C(16)	-108.3(7)
C(6)-C(5)-C(9)-C(16)	70.3(8)	C(4)-C(5)-C(9)-C(10)	129.6(6)
C(6)-C(5)-C(9)-C(10)	-51.8(8)	C(16)-C(9)-C(10)-C(11)	-178.4(4)
C(5)-C(9)-C(10)-C(11)	-55.2(5)	C(9)-C(10)-C(11)-C(15)	-58.4(4)
C(9)-C(10)-C(11)-C(12)	179.7(3)	C(15)-C(11)-C(12)-C(13)	47.8(5)
C(10)-C(11)-C(12)-C(13)	172.3(4)	C(14)-O(1)-C(13)-C(12)	62.2(6)
C(11)-C(12)-C(13)-O(1)	-55.3(5)	C(13)-O(1)-C(14)-C(15)	-63.2(7)
C(10)-C(11)-C(15)-C(14)	-171.7(4)	C(12)-C(11)-C(15)-C(14)	-49.3(5)
O(1)-C(14)-C(15)-C(11)	57.9(6)	C(5)-C(9)-C(16)-F(1)	174.4(6)
C(10)-C(9)-C(16)-F(1)	-60.2(7)	C(5)-C(9)-C(16)-F(2)	59.8(7)
C(10)-C(9)-C(16)-F(2)	-174.7(5)	C(8B)-N(1B)-C(1B)-C(2B)	11.6(13)
N(1B)-C(1B)-C(2B)-C(3B)	0.7(13)	C(1B)-C(2B)-C(3B)-C(4B)	179(3)
C(1B)-C(2B)-C(3B)-C(8B)	-13(2)	C(8B)-C(3B)-C(4B)-C(5B)	16(4)
C(2B)-C(3B)-C(4B)-C(5B)	-177(3)	C(3B)-C(4B)-C(5B)-C(6B)	-10(4)
C(3B)-C(4B)-C(5B)-C(9B)	175(2)	C(4B)-C(5B)-C(6B)-C(7B)	6(5)
C(9B)-C(5B)-C(6B)-C(7B)	-179(3)	C(5B)-C(6B)-C(7B)-C(8B)	-8(5)
C(1B)-N(1B)-C(8B)-C(3B)	-19(2)	C(1B)-N(1B)-C(8B)-C(7B)	-168(2)
C(4B)-C(3B)-C(8B)-N(1B)	-169(2)	C(2B)-C(3B)-C(8B)-N(1B)	20(3)
C(4B)-C(3B)-C(8B)-C(7B)	-18(4)	C(2B)-C(3B)-C(8B)-C(7B)	171(2)
C(6B)-C(7B)-C(8B)-N(1B)	160(3)	C(6B)-C(7B)-C(8B)-C(3B)	14(4)
C(4B)-C(5B)-C(9B)-C(16B)	-75(3)	C(6B)-C(5B)-C(9B)-C(16B)	110(3)
C(4B)-C(5B)-C(9B)-C(10B)	52(3)	C(6B)-C(5B)-C(9B)-C(10B)	-123(3)
C(5B)-C(9B)-C(10B)-C(11B)	58.1(18)	C(16B)-C(9B)-C(10B)-C(11B)	-175.0(13)
C(9B)-C(10B)-C(11B)-C(12B)	72.5(14)	C(9B)-C(10B)-C(11B)-C(15B)	-164.6(13)
C(15B)-C(11B)-C(12B)-C(13B)	48.4(16)	C(10B)-C(11B)-C(12B)-C(13B)	173.5(11)
C(14B)-O(1B)-C(13B)-C(12B)	58(2)	C(11B)-C(12B)-C(13B)-O(1B)	-53.6(19)
C(13B)-O(1B)-C(14B)-C(15B)	-61(3)	O(1B)-C(14B)-C(15B)-C(11B)	58(2)
C(12B)-C(11B)-C(15B)-C(14B)	-48.8(17)	C(10B)-C(11B)-C(15B)-C(14B)	-173.3(13)
C(5B)-C(9B)-C(16B)-F(1B)	-175(2)	C(10B)-C(9B)-C(16B)-F(1B)	56(3)
C(5B)-C(9B)-C(16B)-F(2B)	68(2)	C(10B)-C(9B)-C(16B)-F(2B)	-60(2)

XI. Crystallographic data for radical-relayed difluoromethylation product 43

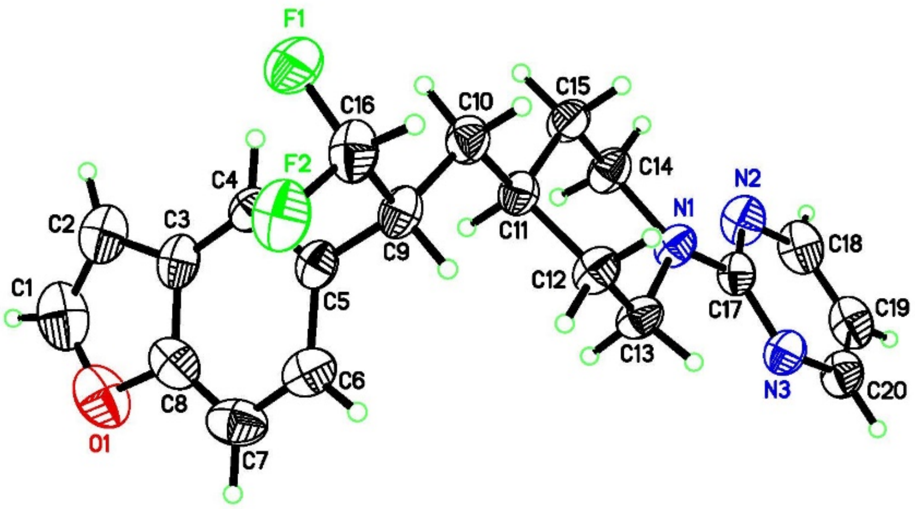

Supplementary Fig. 16. ORTEP drawing of compound 43 (CCDC-2033194). drawn with 50% anisotropic displacement ellipsoids. Further details are provided in CCDC-2033194.

Supplementary Table 8. Crystal data and structure refinement for compound 43.

Empirical formula	$C_{20}H_{21}ON_3F_2$	
Formula weight	357.40	
Temperature	150(2) K	
Wavelength	1.54178 Å	
Crystal system	Monoclinic	
Space group	$P2_1/n$	
Unit cell dimensions	a = 9.1478(2) Å	α = 90°
	b = 9.2119(2) Å	β = 95.6177(9)°
	c = 20.7098(5) Å	γ = 90°
Volume	1736.80(7) $Å^3$	
Z	4	
Density (calculated)	1.367 Mg/m^3	
Absorption coefficient	0.836 mm^{-1}	
F(000)	752	
Crystal size	0.102 x 0.098 x 0.066 mm^3	
θ range for data collection	4.290 to 74.580°	
Index ranges	$-11 \leq h \leq 11$, $-11 \leq k \leq 11$, $-25 \leq l \leq 25$	
Reflections collected	33654	
Independent reflections	3565 [R_{int} = 0.0196]	
Completeness to θ = 67.679°	100.0 %	
Absorption correction	Semi-empirical from equivalents	
Max. and min. transmission	0.865 and 0.815	
Refinement method	Full-matrix least-squares on F^2	
Data / restraints / parameters	3565 / 0 / 235	
Goodness-of-fit on F^2	1.047	
Final R indices [I>2σ(I)]	R1 = 0.0458, wR2 = 0.1276	
R indices (all data)	R1 = 0.0473, wR2 = 0.1288	
Largest diff. peak and hole	0.401 and -0.181 $eÅ^{-3}$	

Supplementary Table 9. Atomic coordinates [x10^4] and equivalent isotropic displacement parameters [Å^{2}x10^3] for compound 43. U(eq) is defined as one third of the trace of the orthogonalized U_{ij} tensor.

	x	y	z	U(eq)
F(1)	10293(1)	-1464(1)	3490(1)	52(1)
F(2)	10034(1)	-581(1)	2515(1)	54(1)
O(1)	3494(1)	-1277(2)	2524(1)	61(1)
N(1)	7605(1)	4974(1)	4960(1)	35(1)
N(2)	6771(2)	6035(2)	5872(1)	45(1)
N(3)	6968(1)	7403(1)	4897(1)	38(1)
C(1)	3448(2)	-2408(3)	2939(1)	62(1)
C(2)	4654(2)	-2533(2)	3356(1)	50(1)
C(3)	5598(2)	-1368(2)	3198(1)	38(1)
C(4)	7014(2)	-887(2)	3435(1)	34(1)
C(5)	7598(2)	299(2)	3138(1)	33(1)
C(6)	6766(2)	1004(2)	2624(1)	42(1)
C(7)	5374(2)	553(2)	2391(1)	51(1)
C(8)	4842(2)	-635(2)	2687(1)	45(1)
C(9)	9139(2)	850(2)	3344(1)	37(1)
C(10)	9414(2)	1181(2)	4072(1)	38(1)
C(11)	8374(2)	2321(2)	4307(1)	33(1)
C(12)	8574(2)	3813(2)	4012(1)	38(1)
C(13)	7518(2)	4920(2)	4248(1)	38(1)
C(14)	7505(2)	3562(2)	5281(1)	40(1)
C(15)	8582(2)	2483(2)	5042(1)	38(1)
C(16)	10301(2)	-192(2)	3148(1)	45(1)
C(17)	7088(2)	6174(2)	5250(1)	33(1)
C(18)	6310(2)	7234(2)	6146(1)	50(1)
C(19)	6138(2)	8546(2)	5829(1)	48(1)
C(20)	6493(2)	8566(2)	5195(1)	44(1)
H(1A)	2635	-3051	2935	75
H(2A)	4845	-3242	3686	60
H(4A)	7554	-1364	3788	40
H(6A)	7177	1823	2430	51
H(7A)	4818	1041	2045	61
H(9A)	9263	1782	3108	44
H(10A)	9309	272	4318	46
H(10B)	10438	1524	4169	46
H(11A)	7342	1996	4181	40
H(12A)	9595	4145	4128	46
H(12B)	8413	3742	3534	46
H(13A)	7748	5891	4081	46
H(13B)	6502	4669	4075	46
H(14A)	6494	3179	5194	48
H(14B)	7715	3687	5756	48
H(15A)	8445	1527	5246	46
H(15B)	9597	2812	5175	46
H(16A)	11292	272	3222	54
H(18A)	6086	7183	6584	59
H(19A)	5797	9386	6033	58
H(20A)	6392	9453	4961	53

Supplementary Table 10. Bond lengths [Å] and angles [°] for cpmpound 43.

F(1)-C(16)	1.370(2)	F(2)-C(16)	1.3582(19)
O(1)-C(1)	1.354(3)	O(1)-C(8)	1.379(2)
N(1)-C(17)	1.3646(18)	N(1)-C(14)	1.4675(18)
N(1)-C(13)	1.4694(17)	N(2)-C(18)	1.329(2)
N(2)-C(17)	1.3539(19)	N(3)-C(20)	1.3302(19)
N(3)-C(17)	1.3464(19)	C(1)-C(2)	1.337(3)
C(2)-C(3)	1.434(2)	C(3)-C(8)	1.383(2)
C(3)-C(4)	1.412(2)	C(4)-C(5)	1.386(2)
C(5)-C(6)	1.405(2)	C(5)-C(9)	1.520(2)
C(6)-C(7)	1.381(2)	C(7)-C(8)	1.367(3)
C(9)-C(16)	1.518(2)	C(9)-C(10)	1.534(2)
C(10)-C(11)	1.5278(19)	C(11)-C(12)	1.522(2)
C(11)-C(15)	1.523(2)	C(12)-C(13)	1.5187(19)
C(14)-C(15)	1.516(2)	C(18)-C(19)	1.377(3)
C(19)-C(20)	1.383(2)		
C(1)-O(1)-C(8)	104.93(14)	C(17)-N(1)-C(14)	118.65(11)
C(17)-N(1)-C(13)	118.74(12)	C(14)-N(1)-C(13)	115.16(11)
C(18)-N(2)-C(17)	115.70(14)	C(20)-N(3)-C(17)	116.01(13)
C(2)-C(1)-O(1)	113.62(17)	C(1)-C(2)-C(3)	105.40(18)
C(8)-C(3)-C(4)	119.07(15)	C(8)-C(3)-C(2)	105.82(15)
C(4)-C(3)-C(2)	135.10(16)	C(5)-C(4)-C(3)	118.29(14)
C(4)-C(5)-C(6)	119.77(14)	C(4)-C(5)-C(9)	121.71(13)
C(6)-C(5)-C(9)	118.51(13)	C(7)-C(6)-C(5)	122.60(16)
C(8)-C(7)-C(6)	116.14(15)	C(7)-C(8)-O(1)	125.67(16)
C(7)-C(8)-C(3)	124.11(15)	O(1)-C(8)-C(3)	110.22(16)
C(16)-C(9)-C(5)	111.61(12)	C(16)-C(9)-C(10)	109.71(13)
C(5)-C(9)-C(10)	113.66(12)	C(11)-C(10)-C(9)	113.67(12)
C(12)-C(11)-C(15)	107.70(11)	C(12)-C(11)-C(10)	112.96(12)
C(15)-C(11)-C(10)	111.37(12)	C(13)-C(12)-C(11)	111.84(12)
N(1)-C(13)-C(12)	111.82(12)	N(1)-C(14)-C(15)	111.18(12)
C(14)-C(15)-C(11)	111.68(12)	F(2)-C(16)-F(1)	105.27(12)
F(2)-C(16)-C(9)	111.18(14)	F(1)-C(16)-C(9)	111.29(13)
N(3)-C(17)-N(2)	125.63(13)	N(3)-C(17)-N(1)	117.14(12)
N(2)-C(17)-N(1)	117.21(13)	N(2)-C(18)-C(19)	123.52(15)
C(18)-C(19)-C(20)	115.99(14)	N(3)-C(20)-C(19)	123.16(16)

Supplementary Table 11. Anisotropic displacement parameters [$Å^2 x 10^3$] for compound 43. The anisotropic displacement factor exponent takes the form: $-2\pi^2[h^2a^{*2}U_{11}+...+2hka^*b^*U_{12}]$

	U_{11}	U_{22}	U_{33}	U_{23}	U_{13}	U_{12}
F(1)	51(1)	44(1)	61(1)	-5(1)	9(1)	16(1)
F(2)	58(1)	52(1)	56(1)	-13(1)	27(1)	1(1)
O(1)	32(1)	84(1)	67(1)	-18(1)	6(1)	0(1)
N(1)	46(1)	28(1)	31(1)	-1(1)	11(1)	3(1)
N(2)	55(1)	44(1)	38(1)	-7(1)	17(1)	-1(1)
N(3)	40(1)	32(1)	43(1)	-4(1)	9(1)	5(1)
C(1)	37(1)	73(1)	80(1)	-23(1)	18(1)	-7(1)
C(2)	41(1)	47(1)	66(1)	-7(1)	20(1)	-1(1)
C(3)	38(1)	35(1)	46(1)	-11(1)	19(1)	3(1)
C(4)	36(1)	30(1)	36(1)	-2(1)	9(1)	6(1)
C(5)	37(1)	29(1)	35(1)	-6(1)	12(1)	4(1)
C(6)	49(1)	42(1)	39(1)	3(1)	14(1)	9(1)
C(7)	48(1)	64(1)	40(1)	2(1)	6(1)	18(1)
C(8)	33(1)	59(1)	42(1)	-14(1)	5(1)	10(1)
C(9)	38(1)	29(1)	44(1)	-4(1)	13(1)	0(1)
C(10)	37(1)	33(1)	45(1)	-6(1)	4(1)	4(1)
C(11)	35(1)	29(1)	37(1)	-3(1)	6(1)	2(1)
C(12)	49(1)	33(1)	35(1)	-1(1)	14(1)	5(1)
C(13)	53(1)	31(1)	32(1)	1(1)	10(1)	8(1)
C(14)	53(1)	32(1)	35(1)	2(1)	13(1)	0(1)
C(15)	50(1)	29(1)	37(1)	2(1)	5(1)	3(1)
C(16)	38(1)	41(1)	59(1)	-13(1)	14(1)	-1(1)
C(17)	33(1)	33(1)	35(1)	-6(1)	8(1)	-1(1)
C(18)	50(1)	58(1)	43(1)	-16(1)	16(1)	-1(1)
C(19)	40(1)	49(1)	57(1)	-23(1)	9(1)	8(1)
C(20)	41(1)	35(1)	56(1)	-9(1)	5(1)	7(1)

Supplementary Table 12. Torsion angles [°] for compound 43.

C(8)-O(1)-C(1)-C(2)	0.4(2)	O(1)-C(1)-C(2)-C(3)	-0.3(2)
C(1)-C(2)-C(3)-C(8)	0.04(18)	C(1)-C(2)-C(3)-C(4)	-179.05(16)
C(8)-C(3)-C(4)-C(5)	-0.67(19)	C(2)-C(3)-C(4)-C(5)	178.32(15)
C(3)-C(4)-C(5)-C(6)	1.29(19)	C(3)-C(4)-C(5)-C(9)	-177.49(12)
C(4)-C(5)-C(6)-C(7)	-0.8(2)	C(9)-C(5)-C(6)-C(7)	178.01(14)
C(5)-C(6)-C(7)-C(8)	-0.3(2)	C(6)-C(7)-C(8)-O(1)	-178.94(14)
C(6)-C(7)-C(8)-C(3)	1.0(2)	C(1)-O(1)-C(8)-C(7)	179.63(16)
C(1)-O(1)-C(8)-C(3)	-0.32(17)	C(4)-C(3)-C(8)-C(7)	-0.5(2)
C(2)-C(3)-C(8)-C(7)	-179.78(15)	C(4)-C(3)-C(8)-O(1)	179.44(12)
C(2)-C(3)-C(8)-O(1)	0.18(17)	C(4)-C(5)-C(9)-C(16)	70.58(17)
C(6)-C(5)-C(9)-C(16)	-108.22(15)	C(4)-C(5)-C(9)-C(10)	-54.15(17)
C(6)-C(5)-C(9)-C(10)	127.06(14)	C(16)-C(9)-C(10)-C(11)	175.22(12)
C(5)-C(9)-C(10)-C(11)	-59.05(17)	C(9)-C(10)-C(11)-C(12)	-65.01(17)
C(9)-C(10)-C(11)-C(15)	173.63(12)	C(15)-C(11)-C(12)-C(13)	-57.14(16)
C(10)-C(11)-C(12)-C(13)	179.45(12)	C(17)-N(1)-C(13)-C(12)	160.24(13)
C(14)-N(1)-C(13)-C(12)	-50.25(18)	C(11)-C(12)-C(13)-N(1)	53.44(17)
C(17)-N(1)-C(14)-C(15)	-159.38(13)	C(13)-N(1)-C(14)-C(15)	51.08(18)
N(1)-C(14)-C(15)-C(11)	-55.38(17)	C(12)-C(11)-C(15)-C(14)	58.26(16)
C(10)-C(11)-C(15)-C(14)	-177.37(12)	C(5)-C(9)-C(16)-F(2)	49.14(17)
C(10)-C(9)-C(16)-F(2)	176.03(12)	C(5)-C(9)-C(16)-F(1)	-67.87(17)
C(10)-C(9)-C(16)-F(1)	59.03(17)	C(20)-N(3)-C(17)-N(2)	-0.1(2)
C(20)-N(3)-C(17)-N(1)	-178.55(13)	C(18)-N(2)-C(17)-N(3)	-0.3(2)
C(18)-N(2)-C(17)-N(1)	178.17(14)	C(14)-N(1)-C(17)-N(3)	-169.19(13)
C(13)-N(1)-C(17)-N(3)	-20.7(2)	C(14)-N(1)-C(17)-N(2)	12.2(2)
C(13)-N(1)-C(17)-N(2)	160.66(14)	C(17)-N(2)-C(18)-C(19)	0.7(3)
N(2)-C(18)-C(19)-C(20)	-0.7(3)	C(17)-N(3)-C(20)-C(19)	0.1(2)
C(18)-C(19)-C(20)-N(3)	0.2(2)		

XII. Computational details

The geometry optimizations and zero-point vibrational energy (ZPVE) were carried out using the B3LYP functional with the LACVP** basis set (LanL2DZ for Cu and 6-31G** for the others). Then single point energy calculations using a larger basis set (LACV3P++**, LanL2TZ for Cu, and 6-311++G** for the others) were performed on the B3LYP/LACVP** optimized structures to obtain more accurate electronic energies. All calculations were performed under the influence of solvent, which was simulated using the Poisson-Boltzmann self-consistent polarizable continuum method implemented in Jaguar to represent DMSO (dielectric constant = 47.24 and effective radius = 2.41 Å). All energies discussed in this work are Gibbs free energies, calculated as **Supplementary Equation 1**.

$$G = E_{elect} + ZPVE + \sum_{v} \frac{hv}{e^{hv/k_B T} - 1} + \frac{n}{2} k_B T - T \times (S_{trans} + S_{rot} + S_{vib})$$

where T = 298 K and *n* = 8.

Supplementary Table 13. Energy components for forming the Gibbs free energy for each stationary points along the reaction pathway.

	E_{elec}	G_{solv}	ZPE	4KT	H_{vib}	S_{vib}	S_{trans}	S_{rot}	G(298.15)
[Cu^{II}.Bpy.CHF$_2$.CHF$_2$] **(Cu-2)**	-1168.598744	-11.758	126.839	2.370	9.822	65.470	43.195	33.773	-1168.463598
Benzylic.Rad **(Rad)**	-310.315114	-2.925	89.747	2.370	2.884	16.911	39.865	28.029	-310.208675
[Cu^{III}.Bpy.CHF$_2$.CHF$_2$.Rad]-RB **(TS-2)**	-1478.904157	-13.506	217.303	2.370	15.139	101.435	44.039	35.495	-1478.637468
[Cu^{III}.Bpy.CHF$_2$.CHF$_2$.Rad] **(Cu-2A)**	-1478.923036	-12.189	220.006	2.370	14.649	96.455	44.039	35.393	-1478.648307
[Cu^{III}.Bpy.CHF$_2$.CHF$_2$.Rad]-RE **(TSre-2)**	-1478.892748	-12.365	219.190	2.370	14.531	95.886	44.039	35.423	-1478.619531
[Cu^{I}.Bpy.CHF$_2$]	-930.168151	-13.849	113.369	2.370	7.395	48.634	42.679	33.072	-930.053094
Benzylic-CHF$_2$	-548.830498	-4.200	107.294	2.370	4.436	27.028	41.045	30.256	-548.702081
[Cu^{II}.Tpy.CHF$_2$]$^{+1}$ **(Cu-1)**	-1177.133091	-44.102	158.288	2.370	9.251	62.144	43.427	34.473	-1176.999145
[Cu^{III}.Tpy.CHF$_2$.Rad]$^{+1}$ **(Cu-1A)**	-1487.458265	-39.939	249.914	2.370	15.331	100.853	44.215	35.751	-1487.181354
[Cu^{III}.Tpy.CHF$_2$.Rad]$^{+1}$-RE **(TSre-1)**	-1487.449876	-39.841	250.034	2.370	14.792	96.183	44.215	35.739	-1487.171251
[Cu^{I}.Tpy]$^{+1}$	-938.694688	-39.939	144.291	2.370	7.397	45.354	42.953	33.588	-938.570745
CH-Rad	-235.278660	-0.403	97.738	2.370	2.015	10.418	39.166	26.311	-235.152619
[Cu^{III}.Bpy.CHF$_2$.CHF$_2$.CH] **(Cu-2B)**	-1403.904045	-10.966	227.906	2.370	13.788	91.113	43.881	35.081	-1403.613387
[Cu^{III}.Bpy.CHF$_2$.CHF$_2$.Cy]-RE **(TSre-3)**	-1403.875594	-12.571	227.185	2.370	13.776	91.840	43.881	35.121	-1403.589028
CH-CHF$_2$	-473.815538	-2.556	115.607	2.370	3.501	20.148	40.592	29.157	-473.668737

Coordinates for optimized structures

[Cu^{II}.Bpy.CHF_2.CHF_2] **(Cu-2)**

Cu	-0.00032476	-1.02482827	-0.00297272
N	1.29787130	0.63172073	-0.28047562
C	0.73777943	1.84572965	-0.08166212
C	2.62616346	0.52841892	-0.43186382
C	1.52241074	3.00346897	-0.02539015
C	3.46516628	1.63878815	-0.40868544
H	3.00312685	-0.47916970	-0.56828575
C	2.89974363	2.89667844	-0.19634530
H	1.06930140	3.96932428	0.16669478
H	4.53402204	1.51477884	-0.54495174
H	3.52417240	3.78407181	-0.15455336
N	-1.29819227	0.63156548	0.27660149
C	-2.62620350	0.52835827	0.43004046
C	-0.73777355	1.84576686	0.08006723
C	-3.46477800	1.63911233	0.41061390
C	-1.52205576	3.00388532	0.02718291
C	-2.89919911	2.89717634	0.19983335
H	-4.53342318	1.51527771	0.54862894
H	-1.06882441	3.97000003	-0.16330795
H	-3.52335203	3.78487857	0.16076742
H	-3.00318737	-0.47936884	0.56546674
C	-1.36465545	-2.38345750	-0.60244034
H	-1.01496950	-3.36018692	-0.96490323
C	1.36498923	-2.37970066	0.60453259
H	1.01593614	-3.35554638	0.96997659
F	-2.15594719	-1.83195897	-1.60124829
F	-2.22843230	-2.63701694	0.46569222
F	2.15234846	-1.82286183	1.60353239
F	2.23232778	-2.63599146	-0.45998906

Benzylic.Rad **(Rad)**

C	-0.95249460	-1.42522042	-2.16925984
H	-1.87953144	-1.50949728	-1.60777710
C	-0.67915634	-0.18155450	-2.78894791
C	-1.59638700	0.90218380	-2.66400747
C	-1.34914485	2.12761534	-3.26125444
H	-2.50479484	0.75301320	-2.08535459
C	0.73716281	1.28130109	-4.14791238
C	-0.18069546	2.32994371	-4.00914685

H	-2.06664722	2.93613506	-3.14905268
H	1.64471673	1.43175353	-4.72671068
H	0.01104862	3.29118269	-4.47683579
C	-0.05972642	-2.62487733	-2.23631770
H	0.94025810	-2.41889210	-1.82793086
H	-0.47885944	-3.46167257	-1.67191727
H	0.09399307	-2.96819943	-3.26982200
C	0.50031028	0.05011077	-3.55390978
H	1.22387492	-0.75038037	-3.67347241

[Cu^{III}.Bpy.CHF$_2$.CHF$_2$.Rad]-RB **(TS-2)**

C	0.29914207	2.33797158	1.01118457
C	-1.80409769	1.82906864	0.16002111
C	-2.20865543	3.16343748	0.29044035
C	-1.30915528	4.10071823	0.78969452
C	-0.02744483	3.68459511	1.14980267
H	1.27063445	1.95063602	1.29797990
H	-3.21630991	3.46333778	0.02638747
H	-1.60907667	5.13825883	0.90363998
H	0.70527520	4.38299470	1.54004012
C	-2.68938284	0.76384922	-0.37401735
C	-3.89879002	1.02778595	-1.03060352
C	-4.66962465	-0.03962626	-1.48260671
H	-4.22816637	2.04652274	-1.19853487
C	-2.99175772	-1.52594455	-0.63773997
C	-4.21284107	-1.34204144	-1.28201892
H	-5.61140997	0.14464957	-1.99090151
H	-2.56748452	-2.50944588	-0.46851303
H	-4.78335983	-2.20083983	-1.61926463
N	-0.56648471	1.43017036	0.53119319
N	-2.25319483	-0.50081060	-0.19044572
Cu	-0.28600387	-0.69595083	0.76396001
C	0.06640483	-2.69557901	0.82875251
F	1.37522953	-2.99987538	0.49919442
F	-0.72702467	-3.46075466	-0.04084240
H	-0.10621717	-3.09274968	1.83627524
C	0.82418176	-0.44370079	2.48543885
H	0.40226227	0.31610601	3.16562897
F	2.07995480	0.02668604	2.10570975
F	1.03686238	-1.57574751	3.23314334
C	0.89861708	-0.53001478	-2.09655289
C	2.10448292	0.10814204	-1.68984178

C	2.20896455	1.52827535	-1.74731962
C	3.37813675	2.18278938	-1.39058876
H	1.35231847	2.09953967	-2.09753268
C	4.40912471	0.05794543	-0.87219586
C	4.49060244	1.45389728	-0.94990937
H	3.43152212	3.26684328	-1.45824128
H	5.26274385	-0.51283607	-0.51628854
H	5.40544217	1.96672529	-0.66670688
C	3.24727079	-0.60829396	-1.23388875
H	3.19495707	-1.68678377	-1.13445621
H	0.08484923	0.12761110	-2.39752571
C	0.75420618	-1.98230122	-2.42622573
H	1.10613110	-2.18294817	-3.45138723
H	1.31539498	-2.62616495	-1.74815832
H	-0.29061261	-2.29824153	-2.37162080

[Cu^{III}.Bpy.CHF_2.CHF_2.Rad] **(Cu-2A)**

C	0.46612487	2.17120929	1.24905186
C	-1.58984452	1.77266459	0.23654053
C	-1.89355716	3.13958062	0.27815447
C	-0.97392442	4.03212398	0.81910684
C	0.23243147	3.54189632	1.31325857
H	1.38321648	1.73932605	1.63113329
H	-2.84636829	3.50034587	-0.09039288
H	-1.20196213	5.09321921	0.85869812
H	0.97965076	4.19928643	1.74365358
C	-2.53352911	0.77052935	-0.33255999
C	-3.64077962	1.13138035	-1.11471940
C	-4.47062522	0.12902305	-1.60913493
H	-3.84417259	2.16847517	-1.35555654
C	-3.04804083	-1.47440004	-0.54482620
C	-4.17628439	-1.20241120	-1.31902327
H	-5.33042869	0.38660064	-2.22090223
H	-2.75378989	-2.49184780	-0.30253226
H	-4.79621930	-2.01180921	-1.69033492
N	-0.41785672	1.30811841	0.72706165
N	-2.25221790	-0.51569124	-0.06375319
Cu	0.03172622	-0.74565437	0.79078553
C	0.48844482	-2.62615785	1.07712478
F	1.73535626	-3.02393200	0.67189917
F	-0.43074856	-3.38235274	0.38097818
H	0.38634386	-2.86529436	2.13640931

C	-0.56925967	-0.61951256	2.75281838
H	-1.53398975	-0.09694185	2.83102389
F	0.37326607	0.15328522	3.42109528
F	-0.69841952	-1.78377977	3.48060736
C	0.72617078	-0.69334429	-1.16978568
C	1.99455325	0.08052947	-1.11187676
C	2.03195510	1.42605849	-1.53304549
C	3.20721924	2.17476135	-1.48572841
H	1.12212217	1.88134504	-1.91922585
C	4.37230008	0.26779509	-0.58921129
C	4.38685604	1.60054918	-1.01039345
H	3.20174293	3.20728390	-1.82778876
H	5.28366044	-0.19155479	-0.21443845
H	5.30453567	2.18094990	-0.97034100
C	3.19958055	-0.48164280	-0.64101504
H	3.19768204	-1.50783499	-0.29052809
H	-0.06833395	-0.05535439	-1.56912573
C	0.76393314	-1.99201256	-1.96576654
H	0.98804947	-1.77856443	-3.02217027
H	1.51955550	-2.68802096	-1.60056485
H	-0.20010166	-2.50628676	-1.92715746

[Cu^{III}.Bpy.CHF_2.CHF_2.Rad]-RE **(TSre-2)**

C	0.64685900	2.27827164	0.75967672
C	-1.53862036	1.87381450	0.09686064
C	-1.89182043	3.20191912	0.37001350
C	-0.92334154	4.08169706	0.84363324
C	0.37458035	3.61388073	1.04214570
H	1.63757415	1.86453027	0.90910778
H	-2.91151159	3.54311835	0.23518822
H	-1.18259601	5.11319147	1.06342459
H	1.16013265	4.26193708	1.41626842
C	-2.52459367	0.86686316	-0.38320077
C	-3.81485856	1.20942739	-0.81392082
C	-4.68414041	0.20377759	-1.22429076
H	-4.13493711	2.24440888	-0.84137567
C	-2.95258021	-1.37995690	-0.76512568
C	-4.25006514	-1.12153100	-1.20001971
H	-5.68600805	0.45311730	-1.56121382
H	-2.55598809	-2.39000783	-0.72407809
H	-4.89542469	-1.93602607	-1.51190773
N	-0.27790607	1.42908452	0.29065043

N	-2.11266767	-0.41627695	-0.36913125
Cu	-0.06845701	-0.73209268	0.63416850
C	0.39565411	-2.77436302	0.61862501
F	1.54222377	-3.52003782	0.53231853
F	-0.61573117	-3.48326081	0.01427566
H	0.17322292	-2.72213841	1.68870390
C	-0.30866819	-0.48780123	2.63950012
H	-1.23418692	-0.02677074	3.02401043
F	0.73173674	0.31876735	3.12932976
F	-0.20146333	-1.70294149	3.34634797
C	0.97311721	-1.55287740	-1.12769981
C	2.19037809	-0.70045160	-0.92129760
C	2.30543806	0.51459031	-1.62155097
C	3.45316386	1.30089397	-1.52357144
H	1.48376852	0.84035289	-2.25458898
C	4.40804889	-0.29929690	0.00344732
C	4.50999244	0.89925042	-0.70505553
H	3.51956518	2.22915826	-2.08545049
H	5.22186007	-0.61938342	0.64869985
H	5.40186881	1.51397395	-0.61885212
C	3.26889744	-1.09573362	-0.10876151
H	3.20663927	-2.02544096	0.44379561
H	0.16608457	-0.96991702	-1.57956114
C	1.21236702	-2.77006238	-2.01860669
H	1.47097104	-2.40247758	-3.02106655
H	2.03749016	-3.39052745	-1.66944150
H	0.31892940	-3.39085805	-2.10739657

[Cu^{I}.Bpy.CHF_2]

C	0.20462922	2.46745362	1.10759490
C	-1.70542398	1.85354065	-0.05682523
C	-2.01726907	3.19100618	-0.33094090
C	-1.15434444	4.18386218	0.13140172
C	-0.02382251	3.82298350	0.86349268
H	1.04228018	2.10746960	1.69842202
H	-2.91676976	3.46082322	-0.87287620
H	-1.37254187	5.22875016	-0.06882467
H	0.66295918	4.57118479	1.24524964
C	-2.55520037	0.70662287	-0.48390723
C	-3.58627795	0.83573760	-1.42213678
C	-4.34501786	-0.27952169	-1.76526921
H	-3.78313093	1.79207934	-1.89189997

C	-3.01365350	-1.55926522	-0.24626280
C	-4.05661918	-1.50449843	-1.16704689
H	-5.14608058	-0.19266316	-2.49322894
H	-2.74884969	-2.48744471	0.24993364
H	-4.62102950	-2.39988154	-1.40374401
N	-0.61041565	1.51639893	0.64448837
N	-2.27924830	-0.48834726	0.09169295
Cu	-0.68550640	-0.63975123	1.37161499
C	0.71810075	-1.04159850	2.65102262
H	0.56353250	-1.61370286	3.57760346
F	1.22899986	0.20962941	3.10406709
F	1.80163681	-1.65960700	2.01476961

Benzylic-CHF_2

C	0.84058220	-0.39103320	2.68899417
H	0.63533182	0.49204510	3.30510719
F	2.13023476	-0.28864233	2.22475231
F	0.79796484	-1.49682355	3.49879168
C	-0.14550596	-0.56346520	1.53713078
H	-1.09858687	-0.80279965	2.02480811
C	0.25726487	-1.75532721	0.64861452
H	0.36515635	-2.66102313	1.25143829
H	-0.50535792	-1.93068146	-0.11437478
H	1.20752729	-1.56333215	0.14422321
C	-0.33906943	0.72567684	0.75214235
C	-1.62041327	1.27407123	0.61396720
C	0.73776701	1.38171400	0.13668820
C	-1.82775665	2.44195453	-0.12104561
H	-2.46684596	0.77831957	1.08396439
C	0.53286626	2.55018442	-0.59635925
H	1.74044473	0.98105307	0.24036733
C	-0.74969731	3.08443923	-0.72911441
H	-2.83062693	2.84909597	-0.21586808
H	1.37928726	3.04635364	-1.06332008
H	-0.90674589	3.99480084	-1.30075099

$[Cu^{II}.Tpy.CHF_2]^{+1}$ **(Cu-1)**

N	1.01871134	1.70097657	-0.15182217
C	0.23650081	2.78072192	-0.01887130
C	0.72525066	4.05081909	-0.34146151
C	2.03781094	4.16018721	-0.79779431
C	2.83563311	3.02387050	-0.92183899

C	2.28477901	1.78372187	-0.58227817
N	2.27350413	-0.60305571	-0.26922603
C	2.84493347	-1.81422604	-0.29722851
C	4.16656301	-2.00988479	-0.69784921
C	4.91599862	-0.90455118	-1.08656455
C	4.32307775	0.35752777	-1.06150506
C	2.99695736	0.48035651	-0.64689549
H	0.10880011	4.93553411	-0.24358834
H	2.44086493	5.13428693	-1.05625757
H	3.85612704	3.11233868	-1.27276671
H	2.21644262	-2.64130965	0.00671694
H	4.58564321	-3.00989510	-0.70223943
H	5.94732196	-1.01637594	-1.40573080
H	4.89144579	1.22974421	-1.36176230
C	-1.13089333	2.48952184	0.48659630
C	-2.08592497	3.48069603	0.71279507
N	-1.40248787	1.18195282	0.72275791
C	-3.34467282	3.11863046	1.19126790
H	-1.85755915	4.52237754	0.52141244
C	-2.61491095	0.83682795	1.17613277
C	-3.61595762	1.77501628	1.42741986
H	-4.09769937	3.87845306	1.37452213
H	-2.77971164	-0.22186677	1.33113966
H	-4.58151958	1.44722152	1.79612865
Cu	0.25836767	-0.13795555	0.33131575
C	-0.47882961	-1.90943451	0.90286435
H	-0.38962144	-2.10563242	1.98191670
F	0.14198391	-2.93063356	0.22854831
F	-1.81009481	-1.96309722	0.56564701

$[Cu^{III}.Tpy.CHF_2.Rad]^{+1}$ **(Cu-1A)**

N	1.41675028	1.19536412	-0.27286063
C	0.75648993	2.35367317	-0.45446176
C	1.44443809	3.55295727	-0.68103199
C	2.83395898	3.53797021	-0.71904937
C	3.50886533	2.33991955	-0.50715008
C	2.76342721	1.17793466	-0.27565038
N	2.57494210	-1.10588129	0.45775123
C	3.07853802	-2.29042808	0.82603477
C	4.44143106	-2.57549120	0.77595964
C	5.30542073	-1.58408617	0.31480037
C	4.78543480	-0.34636054	-0.05874227

C	3.40563805	-0.13193440	0.03577536
H	0.90551933	4.48566356	-0.79191794
H	3.38765859	4.45665415	-0.88575843
H	4.59087172	2.32329580	-0.49585289
H	2.35742062	-3.02047820	1.18072753
H	4.80812452	-3.54676476	1.08959915
H	6.37339281	-1.76739770	0.25025525
H	5.44849479	0.42999938	-0.42016325
C	-0.73063051	2.31403277	-0.36034774
C	-1.53564677	3.30583941	-0.93641848
N	-1.25251396	1.27730934	0.31873519
C	-2.91804840	3.22629502	-0.78188483
H	-1.09931588	4.11313772	-1.51372595
C	-2.58116165	1.20866301	0.46210406
C	-3.45638235	2.16180512	-0.06160167
H	-3.56207282	3.98168492	-1.22194643
H	-2.95330067	0.35301373	1.01890372
H	-4.52652514	2.06401859	0.08807779
Cu	0.41752591	-0.71750555	0.09767123
C	-0.50131951	-1.52571577	1.65670443
H	-0.25857939	-0.73236600	2.38117454
F	0.08189763	-2.69008818	2.05163980
F	-1.84664879	-1.71311734	1.64487953
C	-0.77249895	-2.00352642	-0.97267583
H	-1.77196324	-1.67518719	-0.69666217
C	-0.25975729	-1.36715517	-2.19237096
C	-0.98631090	-0.30533409	-2.78656026
C	-0.55336288	0.28229734	-3.96779561
H	-1.91033433	0.02522443	-2.32133017
C	1.36496941	-1.19565827	-4.00424031
C	0.62530954	-0.15820708	-4.57905233
H	-1.13752702	1.07643098	-4.42309266
H	2.27399582	-1.54452959	-4.48490799
H	0.96089157	0.29935304	-5.50507475
C	-0.52696958	-3.47268607	-0.73275893
H	0.53253373	-3.74102677	-0.75272960
H	-0.94379758	-3.79412125	0.22012890
H	-1.02223298	-4.04695205	-1.52800965
C	0.93182634	-1.79595606	-2.82704336
H	1.50507577	-2.61335672	-2.40192501

$[Cu^{III}.Tpy.CHF_2.Rad]^{+1}$-RE **(TSre-1)**

N	1.30823543	1.14871069	-0.37055410
C	0.62891550	2.29461610	-0.55430948
C	1.29516997	3.48972783	-0.85732408
C	2.68024546	3.47786984	-0.97449644
C	3.37423740	2.29174206	-0.75689879
C	2.65247743	1.13518321	-0.44056860
N	2.49655174	-1.15637260	0.29830650
C	3.02140990	-2.32812261	0.67974151
C	4.39008771	-2.58388068	0.65520230
C	5.24000794	-1.57376457	0.21003706
C	4.69834713	-0.34905387	-0.17318524
C	3.31314678	-0.16252879	-0.11056377
H	0.74684065	4.41681181	-0.97034634
H	3.21837226	4.39099764	-1.20900312
H	4.45492824	2.28167210	-0.81531728
H	2.31254018	-3.07429433	1.02449798
H	4.77164893	-3.54668043	0.97679233
H	6.31314020	-1.73119859	0.16707423
H	5.35277537	0.44392280	-0.51364187
C	-0.85048345	2.24751320	-0.37301158
C	-1.68973430	3.24373125	-0.88968548
N	-1.33032178	1.20556114	0.33018148
C	-3.05915205	3.16770480	-0.64527687
H	-1.29127954	4.05467920	-1.48852218
C	-2.64626747	1.14462086	0.56472865
C	-3.55168579	2.10315783	0.10649415
H	-3.72789318	3.92734390	-1.03857225
H	-2.98622205	0.29035374	1.14447987
H	-4.60929779	2.00899468	0.32925147
Cu	0.39763469	-0.76759922	-0.03515787
C	-0.62723474	-1.67037754	1.53413641
H	-0.28108331	-0.78504398	2.08745986
F	0.04987731	-2.76198902	1.97271462
F	-1.94412677	-1.83177185	1.82566466
C	-1.09873075	-2.16847071	-0.60069574
H	-2.07230675	-1.72717570	-0.40944384
C	-0.52210908	-1.67378332	-1.88670280
C	-1.16333947	-0.61793945	-2.57729242
C	-0.69048233	-0.18152179	-3.81039002
H	-2.05439819	-0.16998185	-2.14801567
C	1.09531449	-1.80955860	-3.70855296
C	0.44154489	-0.77284587	-4.37808323

H	-1.21098310	0.61278695	-4.33715995
H	1.96846253	-2.27778769	-4.15304630
H	0.80608306	-0.43413722	-5.34333639
C	-1.06277260	-3.66465479	-0.37348068
H	-0.04720218	-4.06152871	-0.30374230
H	-1.60218633	-3.94695179	0.52939839
H	-1.54968240	-4.15046123	-1.22789494
C	0.62274143	-2.25965445	-2.47792851
H	1.12831155	-3.08011028	-1.97906448

$[Cu^{I}.Tpy]^{+1}$

N	1.10515079	1.81027978	-0.08945768
C	0.27287152	2.84419013	-0.03876653
C	0.69929640	4.09550110	-0.50087772
C	1.99619893	4.18899989	-1.01664252
C	2.82997897	3.06827115	-1.08912307
C	2.32755887	1.85331067	-0.60721192
N	2.20709664	-0.56383739	-0.36379072
C	2.76442033	-1.78461230	-0.35688060
C	4.11913915	-2.00322356	-0.60161159
C	4.93036146	-0.90519343	-0.87023576
C	4.36140004	0.36791819	-0.88328427
C	2.99944080	0.51672384	-0.62525515
H	0.05381220	4.96641819	-0.48818322
H	2.35377214	5.14373033	-1.38950224
H	3.81820309	3.15113094	-1.52724965
H	2.09838000	-2.61510068	-0.14693319
H	4.51729014	-3.01147009	-0.57872224
H	5.99122807	-1.03202725	-1.06136868
H	4.97691547	1.23941381	-1.07542579
C	-1.07371343	2.48237097	0.50406927
C	-2.00791833	3.44406540	0.88604851
N	-1.34430119	1.14868686	0.61976879
C	-3.23984455	3.04176980	1.40097112
H	-1.76960427	4.49810123	0.79942201
C	-2.53467521	0.77250053	1.11360973
C	-3.51036053	1.68189854	1.51857069
H	-3.97119936	3.78203796	1.70945976
H	-2.70845840	-0.29605417	1.18511950
H	-4.45351403	1.32289780	1.91498891
Cu	0.26530239	-0.06550035	0.15500146

CH-Rad

C	-0.75919541	1.23608173	0.00000000
C	-0.03079065	0.76453412	1.26783732
C	0.11844328	-0.77205158	1.29178676
C	0.65693139	-1.30490877	0.00000000
C	0.11844328	-0.77205158	-1.29178676
C	-0.03079065	0.76453412	-1.26783732
H	0.74790221	-1.08341329	2.13429066
H	0.96723598	1.22194882	1.29824706
H	-0.56209305	1.10310825	2.16538076
H	-1.78496643	0.83864718	0.00000000
H	-0.84913693	2.32913532	0.00000000
H	1.21936157	-2.23529266	0.00000000
H	0.74790221	-1.08341329	-2.13429066
H	-0.88051908	-1.20862372	-1.48932845
H	0.96723598	1.22194882	-1.29824706
H	-0.56209305	1.10310825	-2.16538076
H	-0.88051908	-1.20862372	1.48932845

[Cu^{III}.Bpy.CHF_2.CHF_2.CH] **(Cu-2B)**

C	-2.84560906	0.13667646	-0.11921318
C	-2.55701940	-2.16012935	-0.31748616
C	-4.22529274	-0.00360202	-0.31881801
C	-3.91941200	-2.37311351	-0.50778705
H	-1.85023213	-2.98127705	-0.34753172
C	-4.76903196	-1.26897459	-0.50581371
H	-4.86633782	0.86929122	-0.34682980
H	-4.29615894	-3.37801266	-0.66293940
H	-5.83772425	-1.38652516	-0.65944116
C	-0.22974048	2.65413198	0.15653333
C	-2.21144555	1.46815603	0.07932883
C	-0.89203723	3.86221077	0.37647039
H	0.85372087	2.60205407	0.09538155
C	-2.95575435	2.63405743	0.31970469
C	-2.28292410	3.84342080	0.46796367
H	-0.33131850	4.78513970	0.48266469
H	-4.03551644	2.60433019	0.40787989
H	-2.83910492	4.75714356	0.65747634
N	-2.03275102	-0.94348869	-0.11240094
N	-0.87033756	1.49079286	0.01569347
Cu	0.04396790	-0.74141076	0.11137112
C	0.18943554	-0.99403709	-1.88638755

H	1.16893219	-0.85103158	-2.35351855
C	1.97860718	-0.83106776	0.28311124
H	2.32380947	-1.21265120	1.24898898
F	-0.20248011	-2.30864137	-2.13786513
F	-0.71605901	-0.19092070	-2.55332214
F	2.60825080	-1.56651233	-0.68450857
F	2.42087881	0.47055234	0.15026943
C	-0.13182345	-0.65989323	2.16017284
C	-0.05804283	-2.06812117	2.74646452
C	0.75758963	0.32623076	2.91300967
H	-1.16827577	-0.30227462	2.21796953
C	-0.35738561	-2.07641016	4.26303503
H	0.94621051	-2.49253417	2.58988697
H	-0.75954164	-2.74008698	2.23312239
C	0.45069193	0.32344319	4.42773262
H	1.82109293	0.07808985	2.78452489
H	0.63082118	1.33971560	2.51512387
C	0.55272970	-1.09276055	5.01381737
H	-0.24449396	-3.09082476	4.66828475
H	-1.40741595	-1.78931939	4.42026610
H	1.13310719	1.00492965	4.95332786
H	-0.56597352	0.71051487	4.59012069
H	0.30571955	-1.08377404	6.08318442
H	1.59466218	-1.43793455	4.93839078

[Cu^{III}.Bpy.CHF$_2$.CHF$_2$.Cy]-RE **(TSre-3)**

C	-2.75702775	0.23477823	0.17595408
C	-2.52741606	-2.05129088	0.49405271
C	-4.10211044	0.06834922	-0.17958635
C	-3.85505702	-2.29566862	0.16033759
H	-1.85712355	-2.86666426	0.75058451
C	-4.65629838	-1.20709696	-0.18223978
H	-4.70989925	0.91737703	-0.46728245
H	-4.24276819	-3.30885925	0.16124132
H	-5.69701295	-1.34901502	-0.45785472
C	-0.07110397	2.70144964	0.36817330
C	-2.07875472	1.55966962	0.17156560
C	-0.67123745	3.93073641	0.11288031
H	0.99980684	2.61908952	0.53161198
C	-2.75775422	2.75930390	-0.08807776
C	-2.04736708	3.95400850	-0.11498892
H	-0.07600635	4.83758552	0.09023761

H	-3.82606676	2.76504659	-0.26844516
H	-2.56091316	4.88955323	-0.31624082
N	-1.99438490	-0.82347970	0.52161835
N	-0.75183604	1.55010290	0.41136863
Cu	0.15972570	-0.49775178	0.21330873
C	-0.04997984	-0.91142271	-1.73934026
H	0.80464104	-0.87598433	-2.43144274
C	2.21296316	-0.57054198	0.54462054
H	2.97611985	-0.57105087	1.32573703
F	-0.58423600	-2.20741499	-1.87352384
F	-1.02095684	-0.06461978	-2.30665332
F	2.51919128	-1.62827284	-0.26521031
F	2.42597711	0.61505183	-0.14246786
C	0.93739831	-0.82636919	2.21332557
C	1.42370585	-2.22038801	2.59140624
C	1.46096340	0.28039426	3.11691116
H	-0.15837327	-0.81197791	2.24912840
C	1.10445833	-2.51966512	4.07172302
H	2.51087264	-2.30678388	2.44907799
H	0.96508496	-2.97406633	1.94102032
C	1.15531137	-0.03702805	4.59814306
H	2.55091626	0.38793451	3.01021772
H	1.01697950	1.24167178	2.83837305
C	1.67165599	-1.42902883	4.99305319
H	1.50813778	-3.50275128	4.34437264
H	0.01494667	-2.57915674	4.20562291
H	1.60113129	0.73440021	5.23806419
H	0.06964940	0.00825018	4.76159768
H	1.41882545	-1.64444949	6.03836302
H	2.76982029	-1.43916485	4.92997223

CH-CHF_2

C	-0.47568731	-1.37311633	0.00468420
C	1.06003411	-1.38903541	0.02349796
C	1.63518102	0.03957329	0.03108649
C	1.06270441	0.86461711	1.19853446
C	-0.47355154	0.87631729	1.17857899
C	-1.04760883	-0.54893424	1.16848610
H	1.41400899	-1.91282852	0.92058913
H	1.45263270	-1.93844980	-0.83799803
H	-0.82282643	-0.94498045	-0.94668566
H	-0.85890773	-2.39955937	0.04053527

H	1.41688064	0.42851202	2.14142537
H	1.44983950	1.89135357	1.16255000
H	-0.85291566	1.43506503	2.04205394
H	-0.81973858	1.41275094	0.28357641
H	-0.80020154	-1.04426899	2.11809102
H	-2.14193971	-0.51556450	1.10969482
H	1.35981862	0.53319681	-0.91289048
C	3.15330307	0.03567910	0.06541410
H	3.58853977	1.04371055	0.04390073
F	3.65564874	-0.65905171	-1.00522321
F	3.60913146	-0.58593034	1.20231619

XIII. NMR spectra of radical-relayed difluoromethylation products

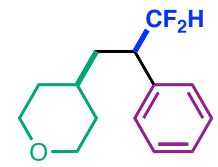

12

^1H NMR (400 MHz, $CDCl_3$)

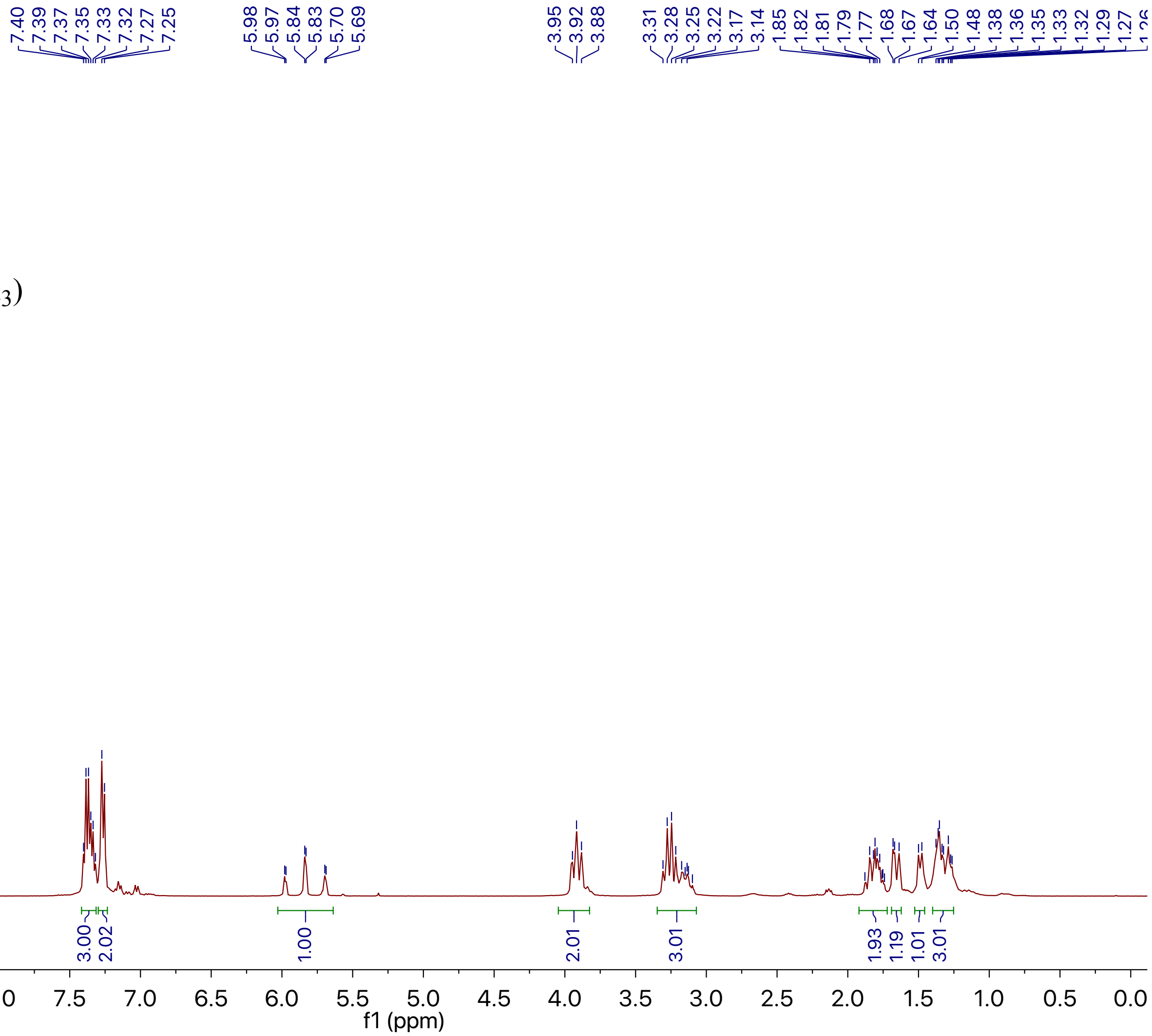

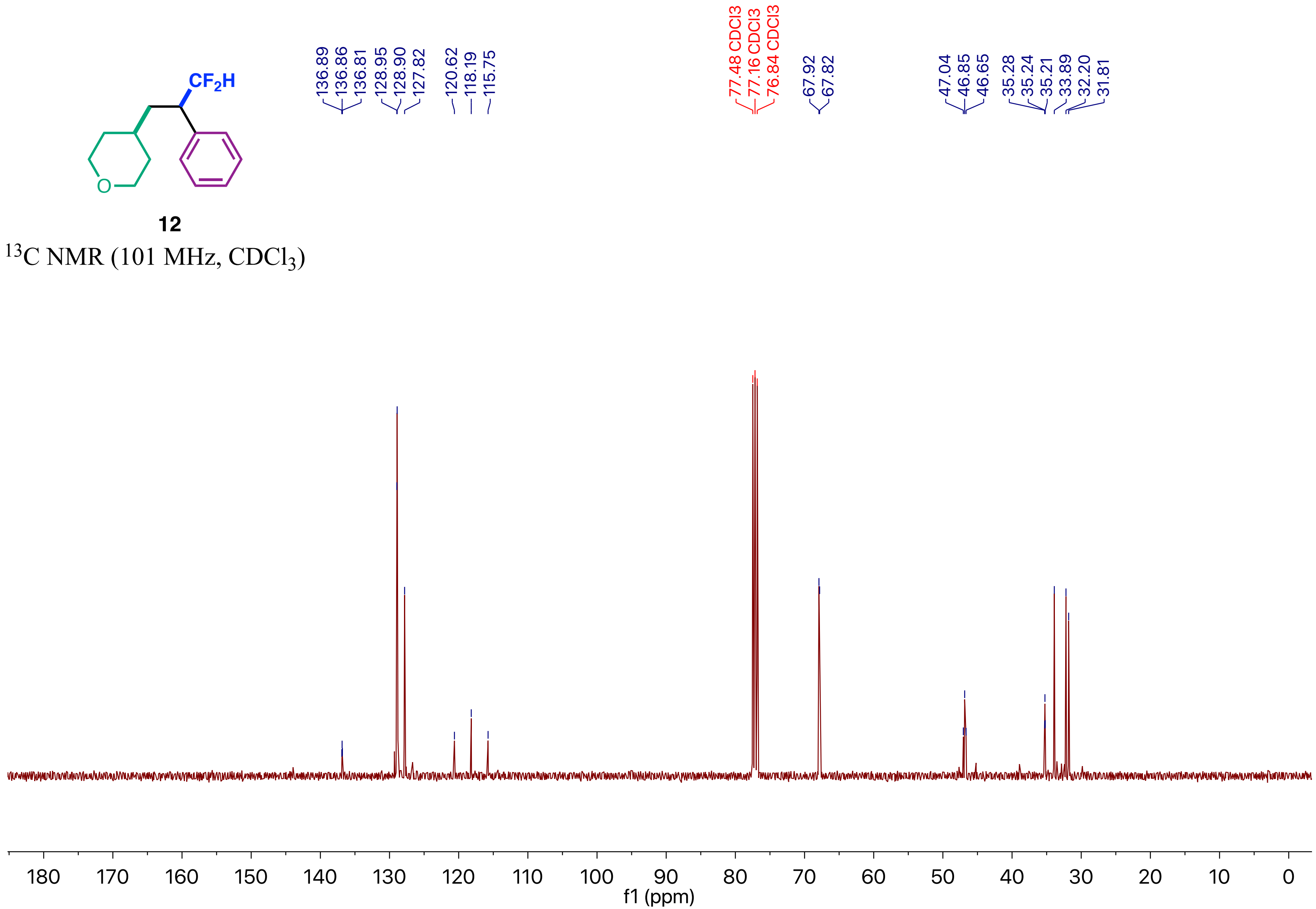
CF2H
O
12
13C NMR (101 MHz, CDCl3)
136.89
136.86
136.81
128.95
128.90
127.82
120.62
118.19
115.75
77.48 CDCl3
77.16 CDCl3
76.84 CDCl3
67.92
67.82
47.04
46.85
46.65
35.28
35.24
35.21
33.89
32.20
31.81
180 170 160 150 140 130 120 110 100 90 80 70 60 50 40 30 20 10 0
f1 (ppm)

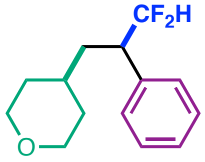

12

^{19}F NMR (376 MHz, $CDCl_3$)

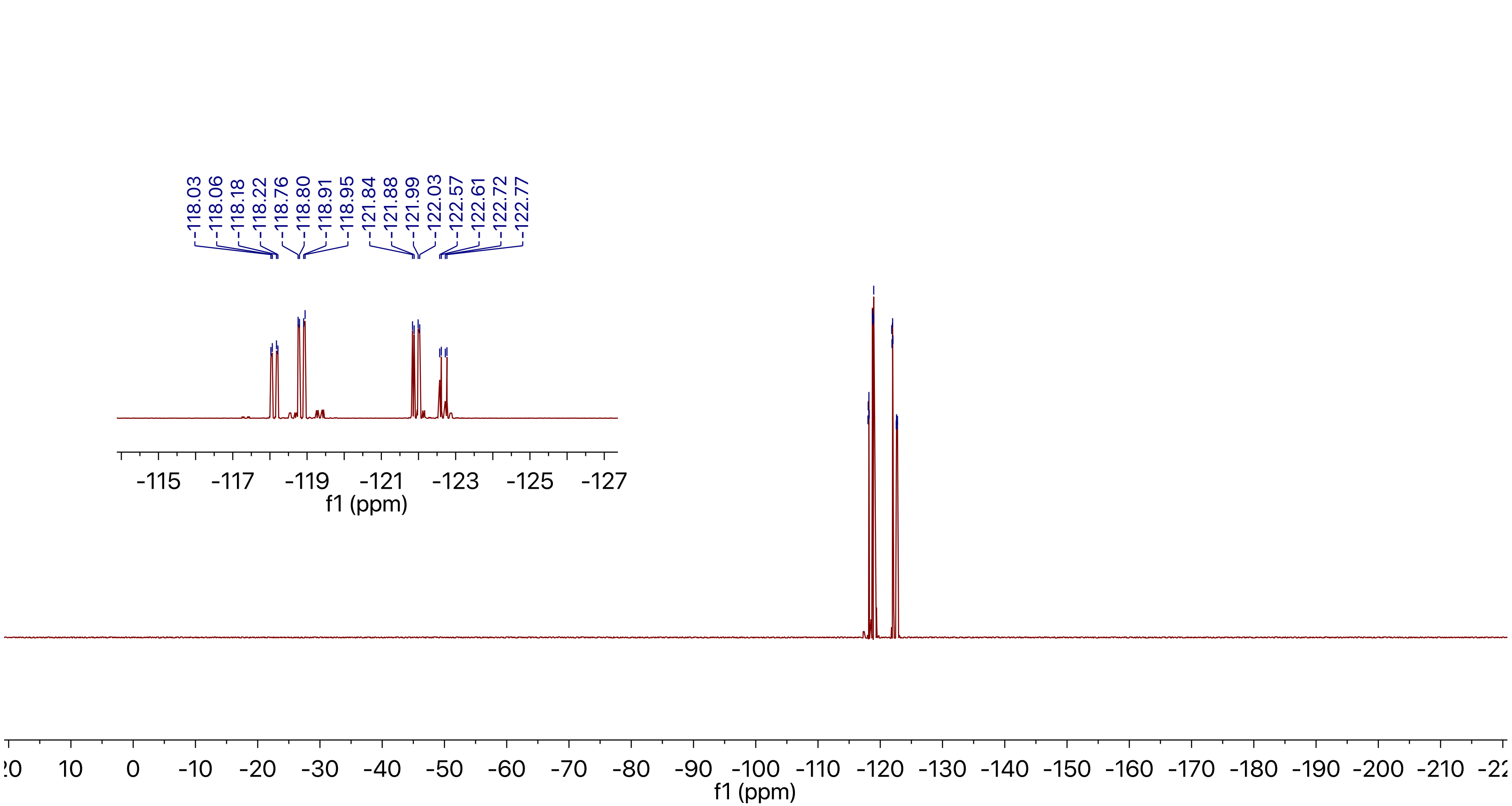

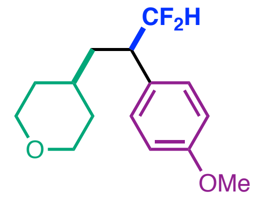

13

^{1}H NMR (400 MHz, $CDCl_3$)

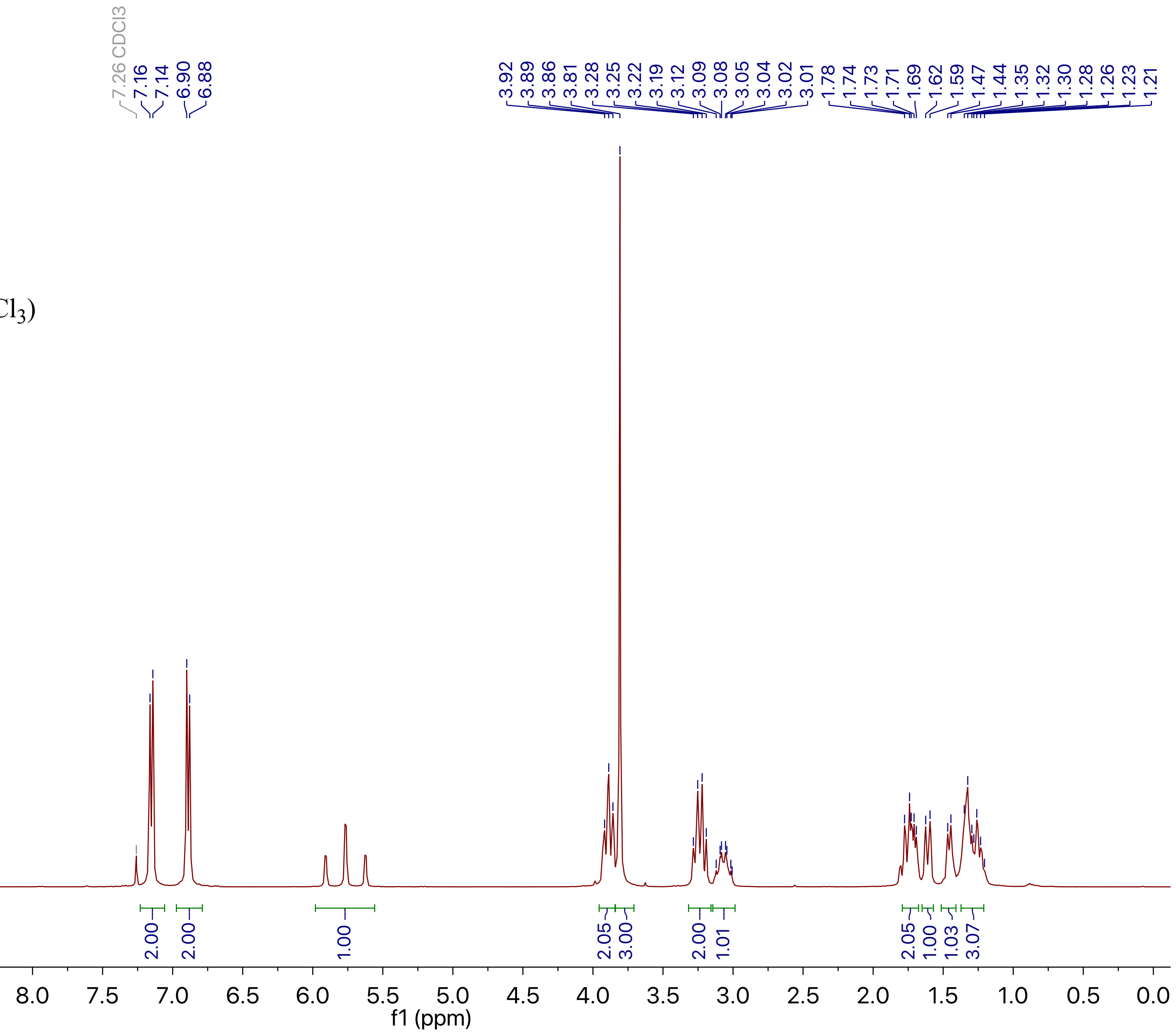

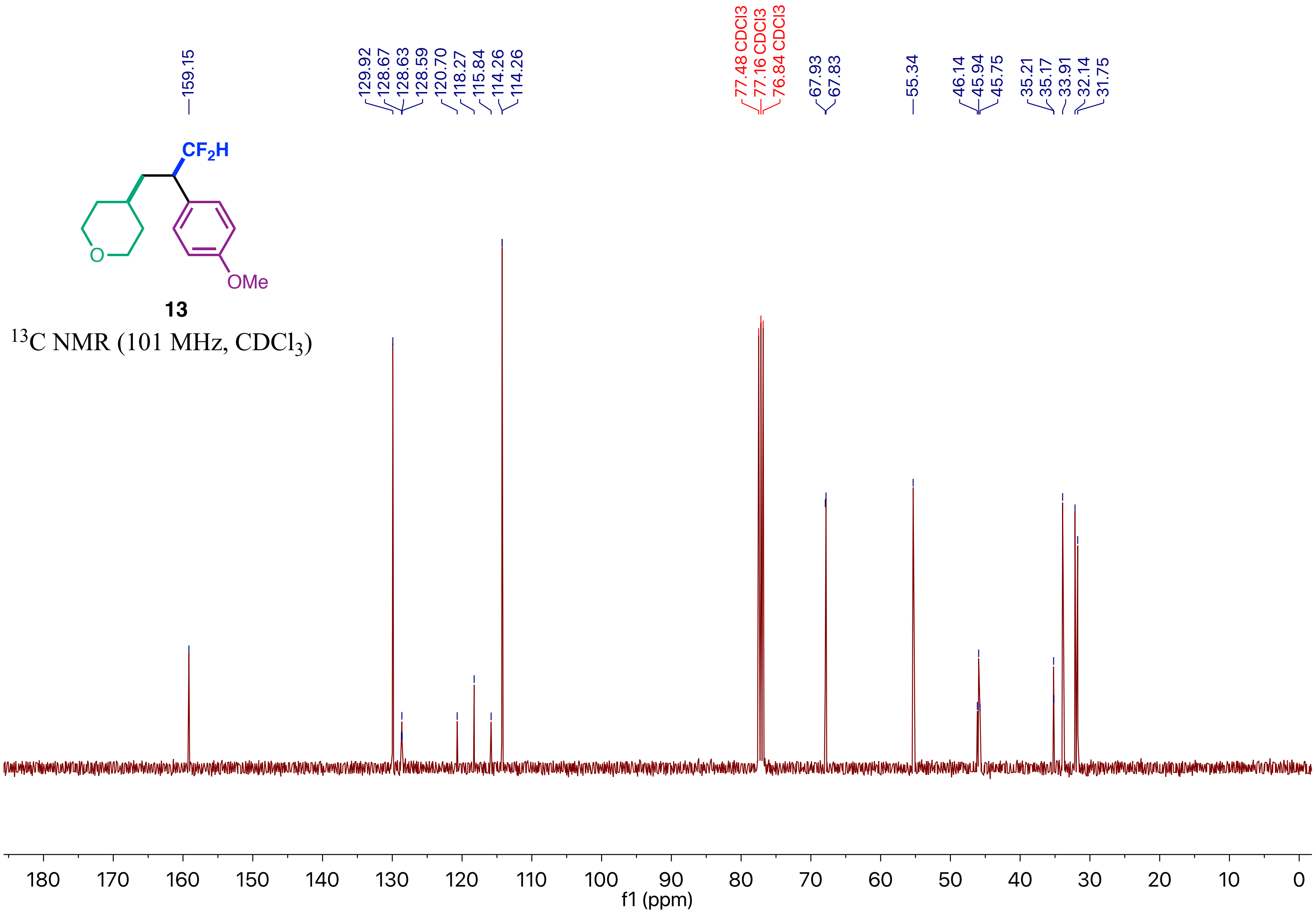

13

^{13}C NMR (101 MHz, $CDCl_3$)

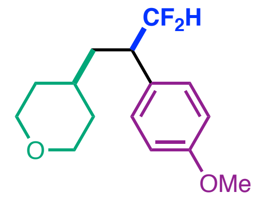

13

^{19}F NMR (376 MHz, $CDCl_3$)

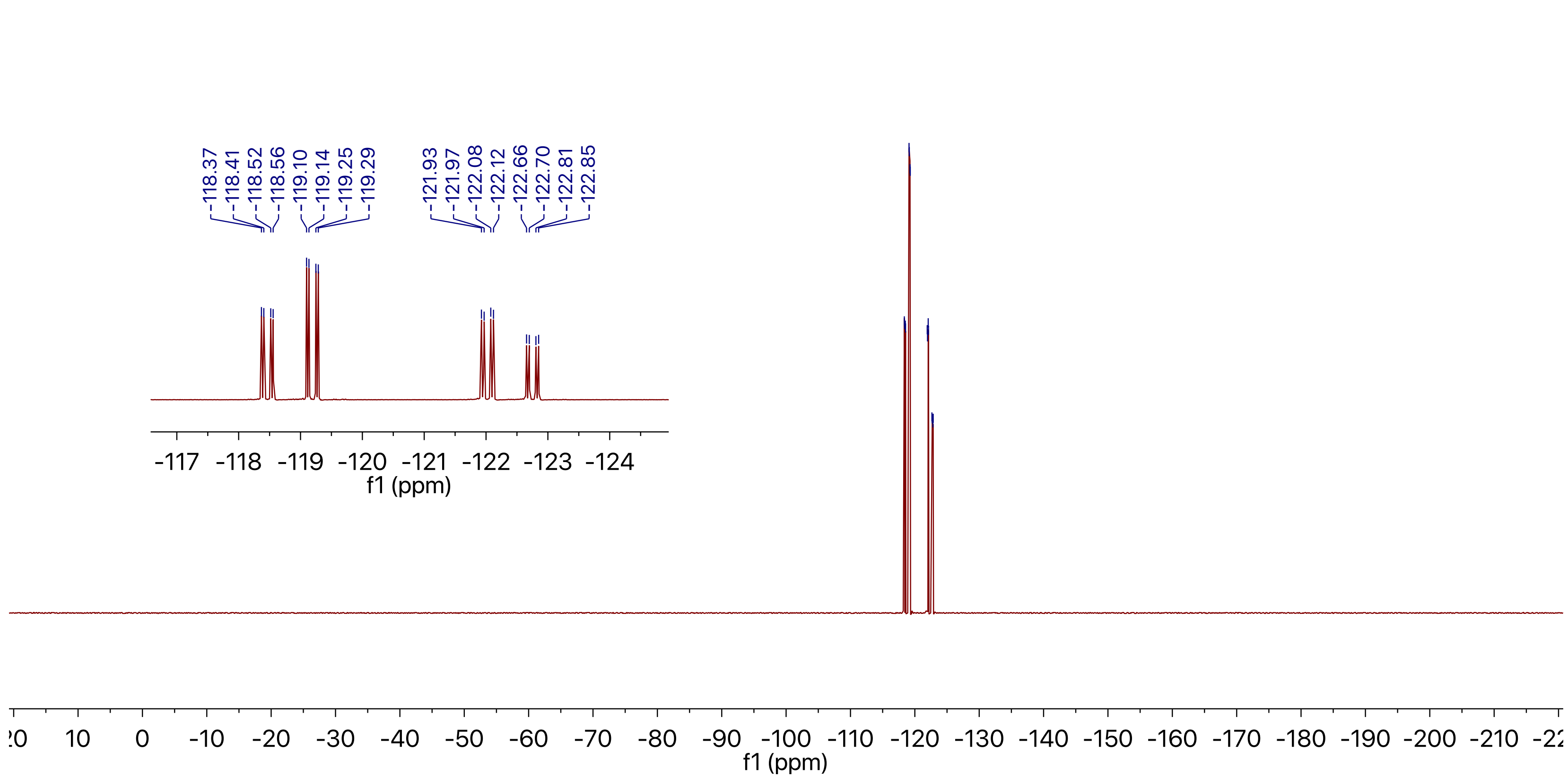

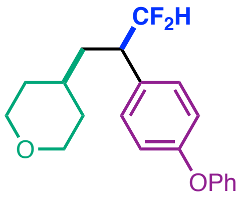

14

^{1}H NMR (400 MHz, $CDCl_3$)

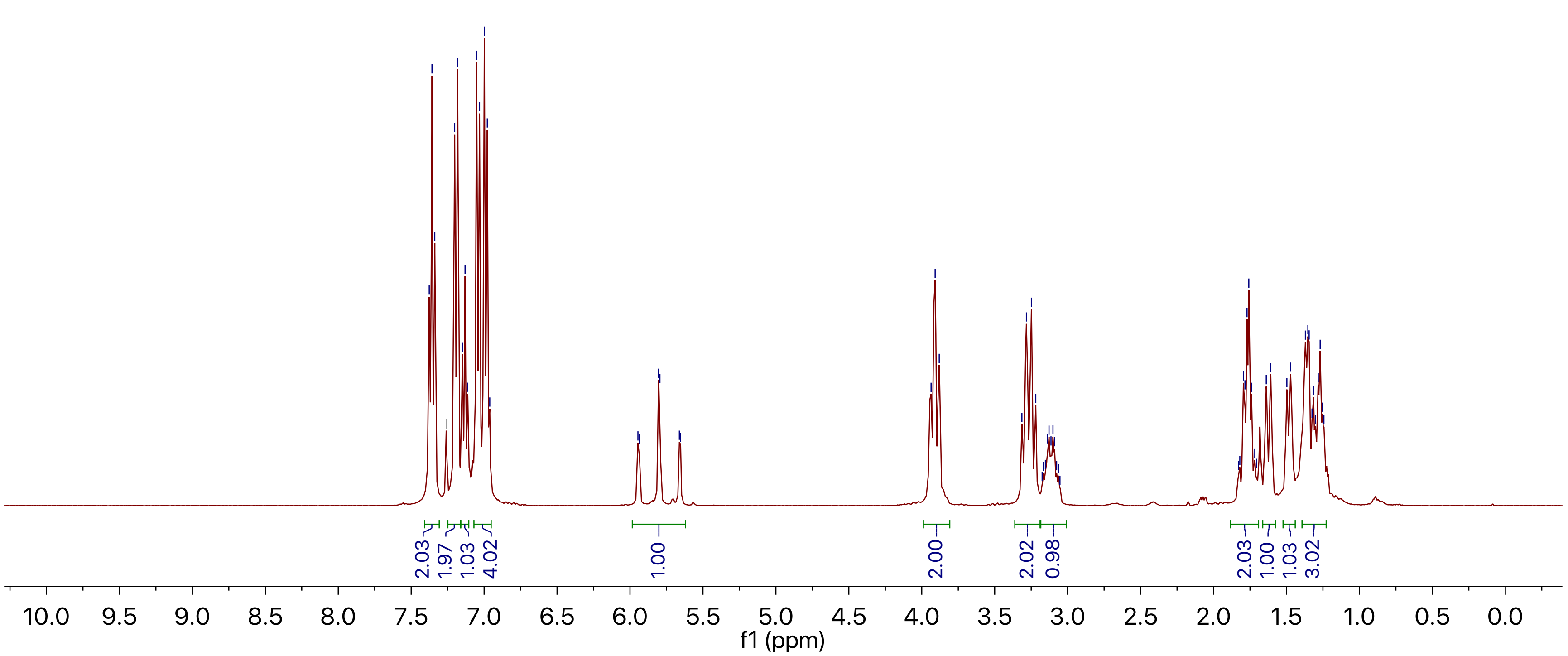

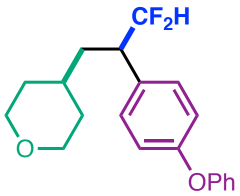

14

^{13}C NMR (101 MHz, $CDCl_3$)

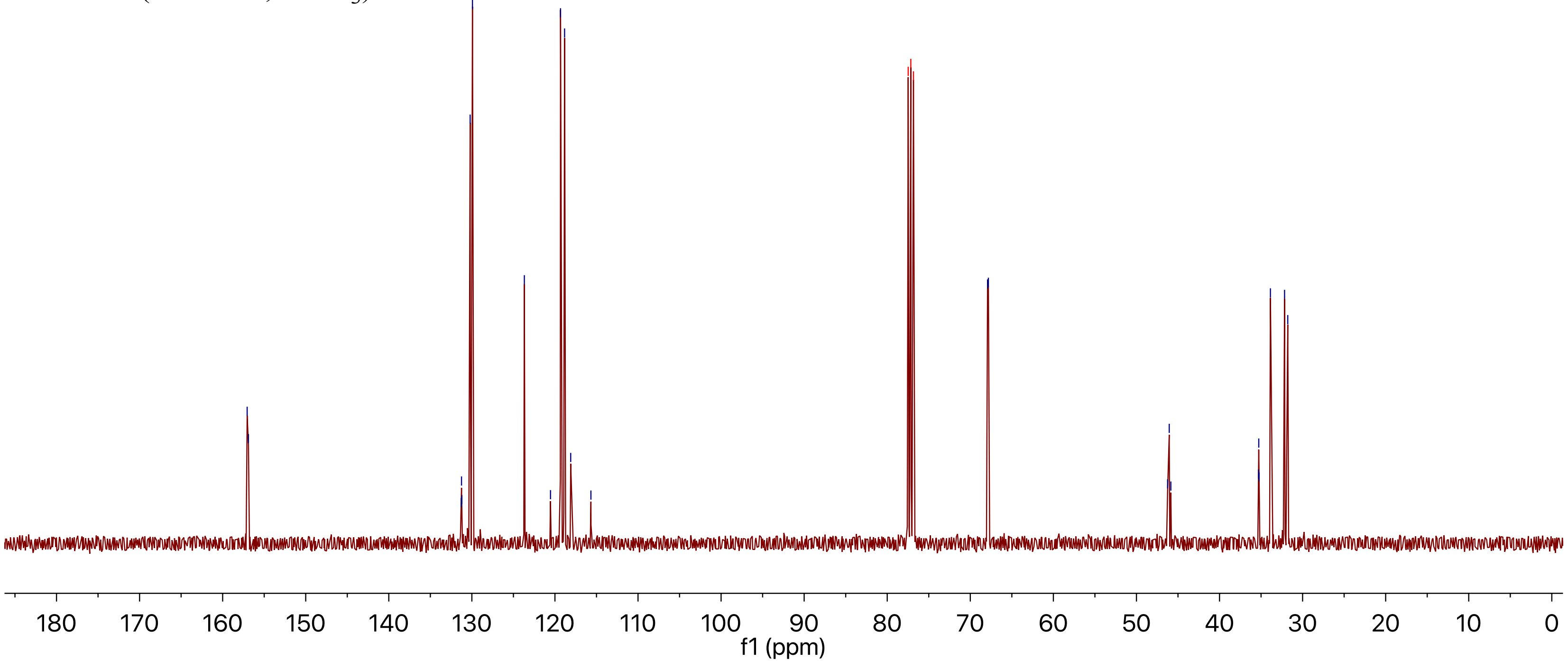

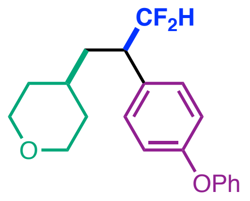

14

^{19}F NMR (376 MHz, CDCl$_3$)

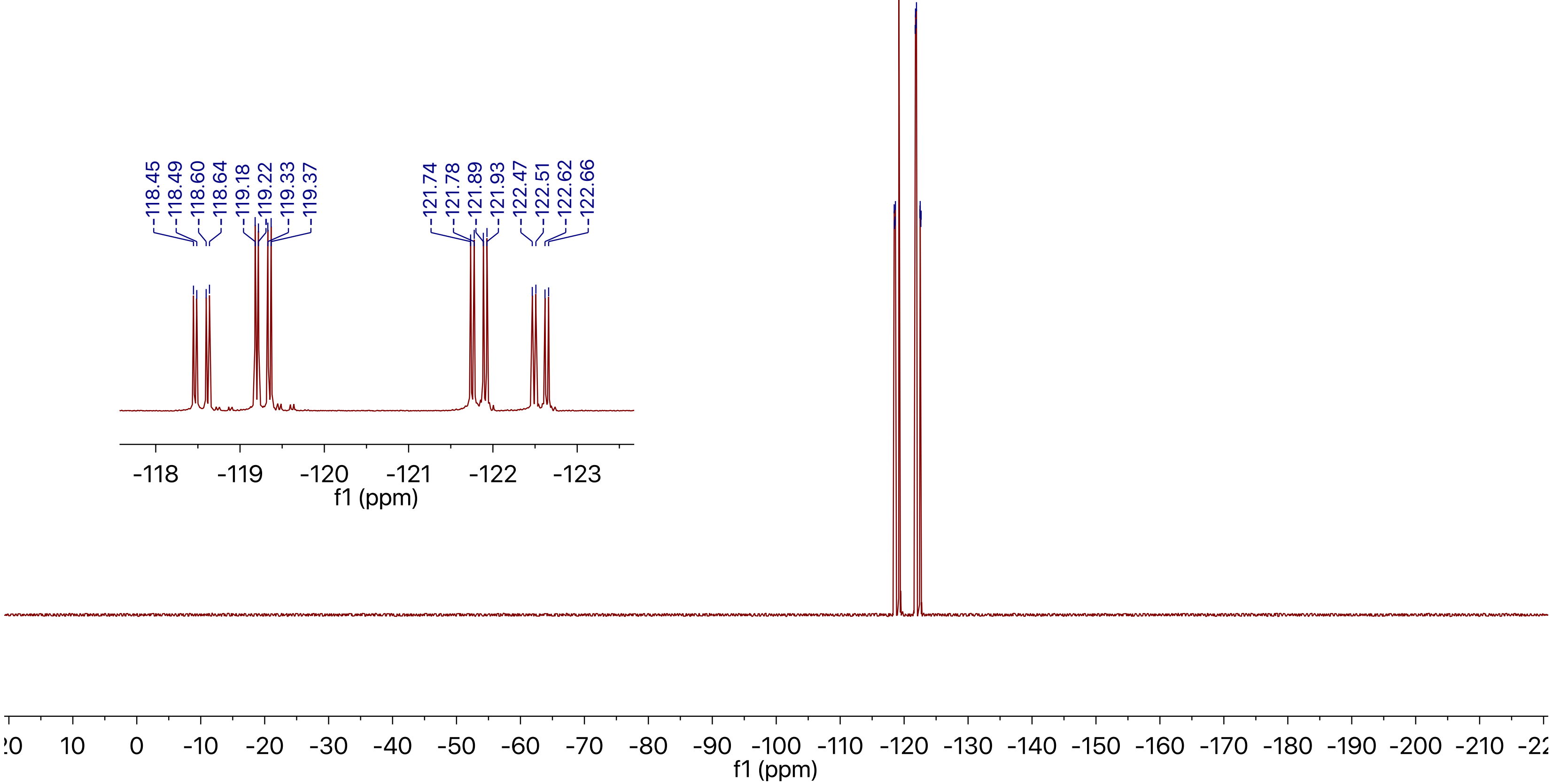

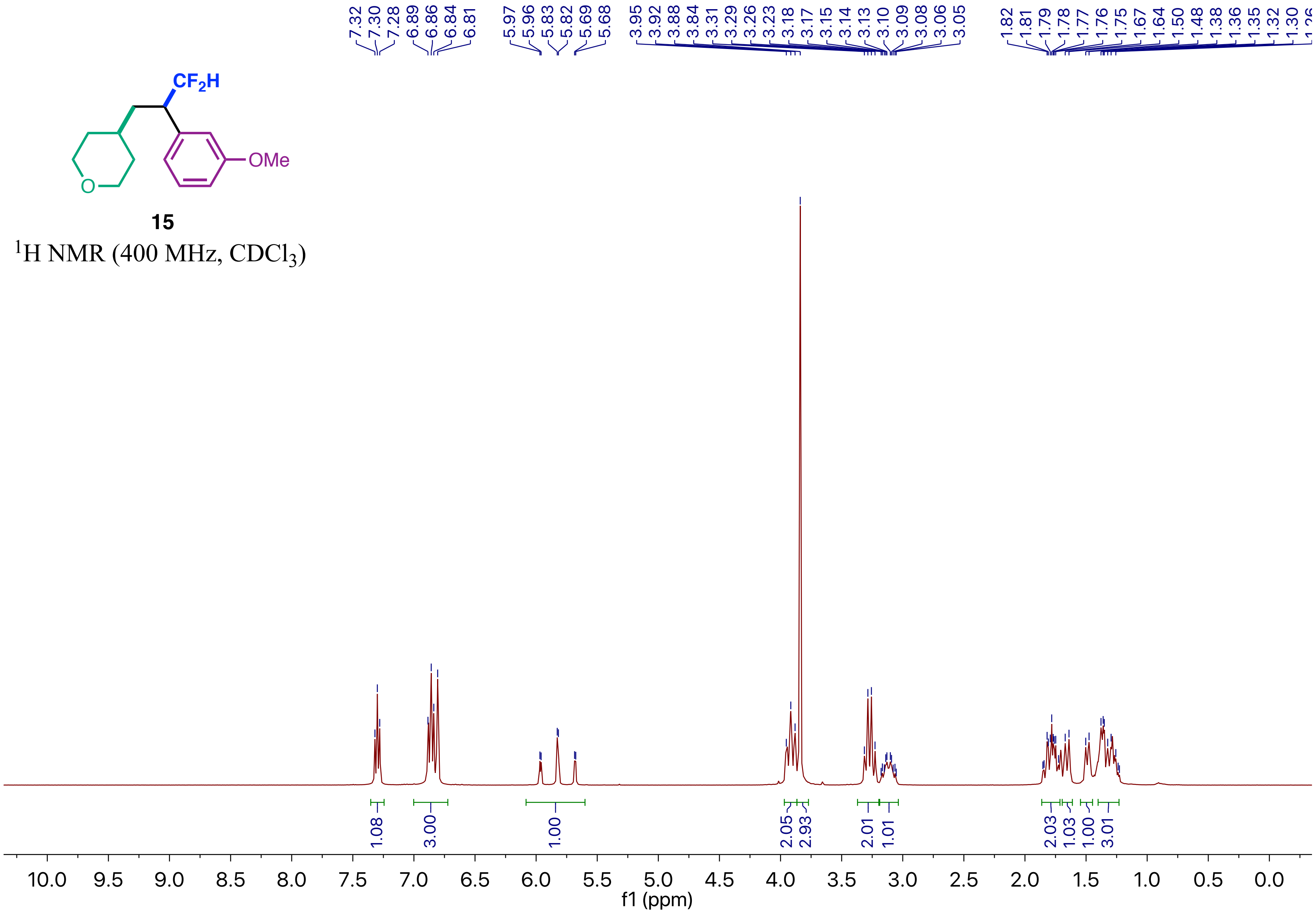

15

^{1}H NMR (400 MHz, $CDCl_3$)

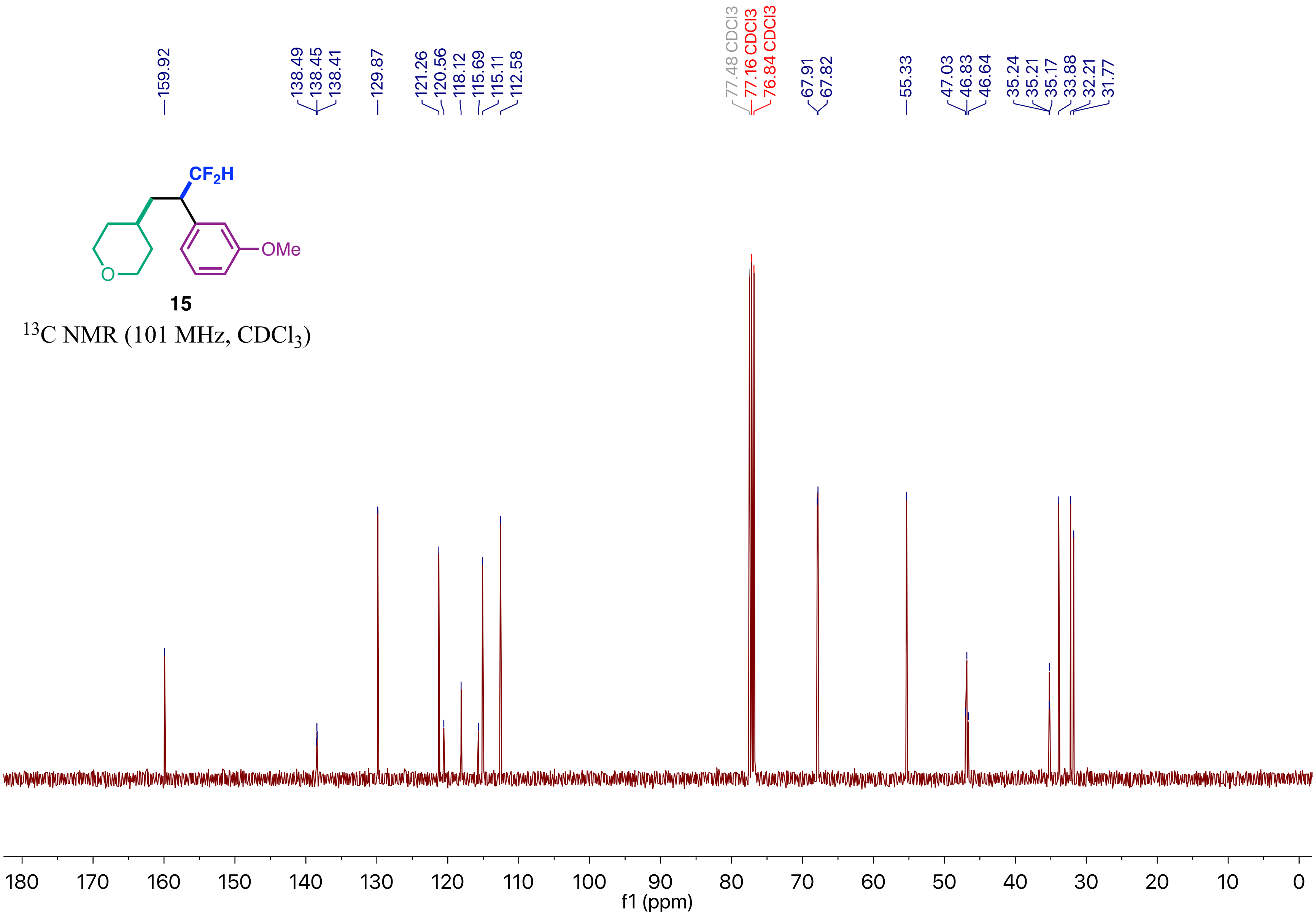

15

^{13}C NMR (101 MHz, $CDCl_3$)

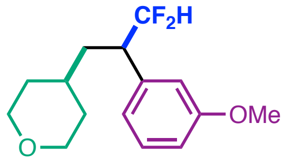

15

^{19}F NMR (376 MHz, $CDCl_3$)

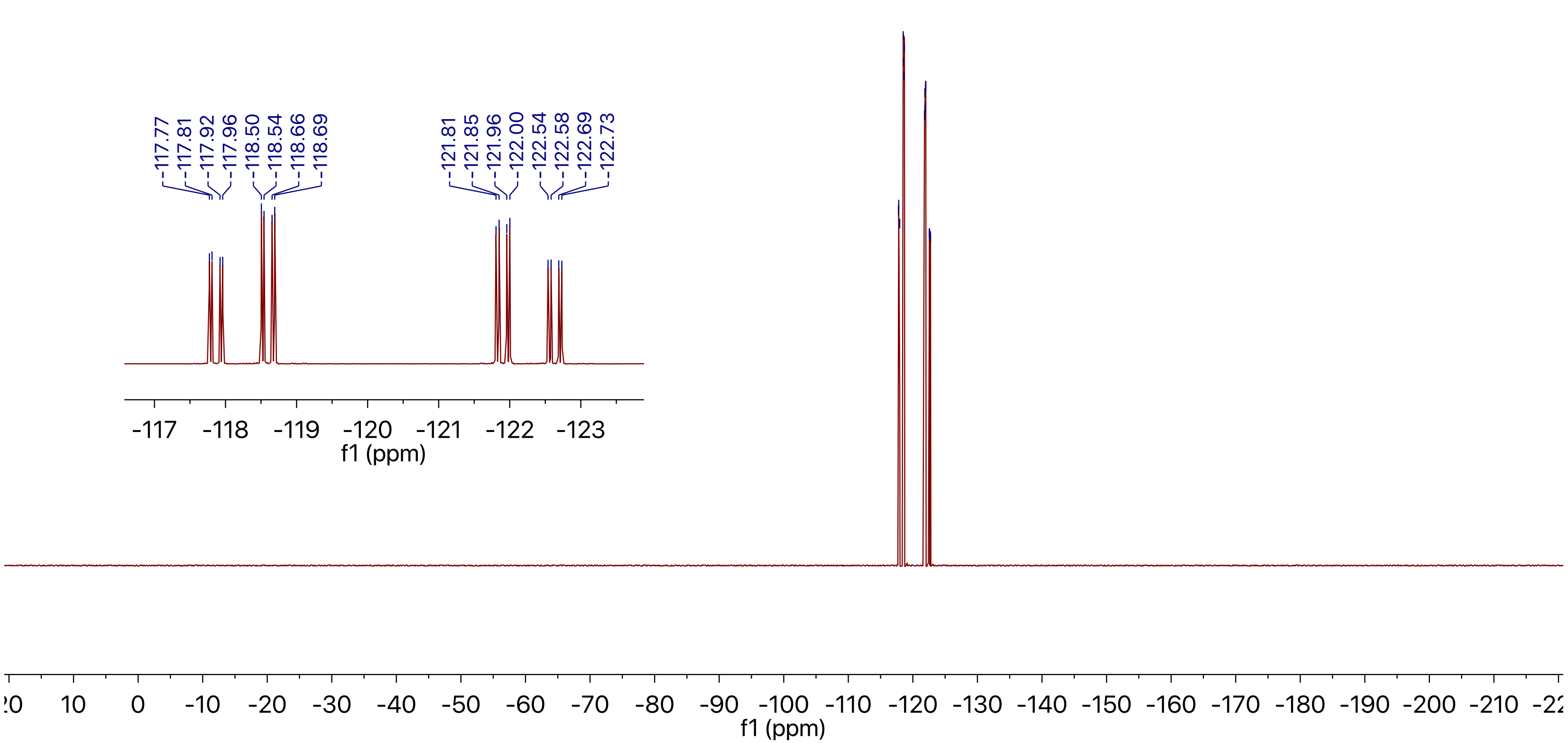

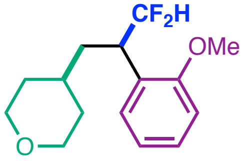

16

^{1}H NMR (400 MHz, $CDCl_3$)

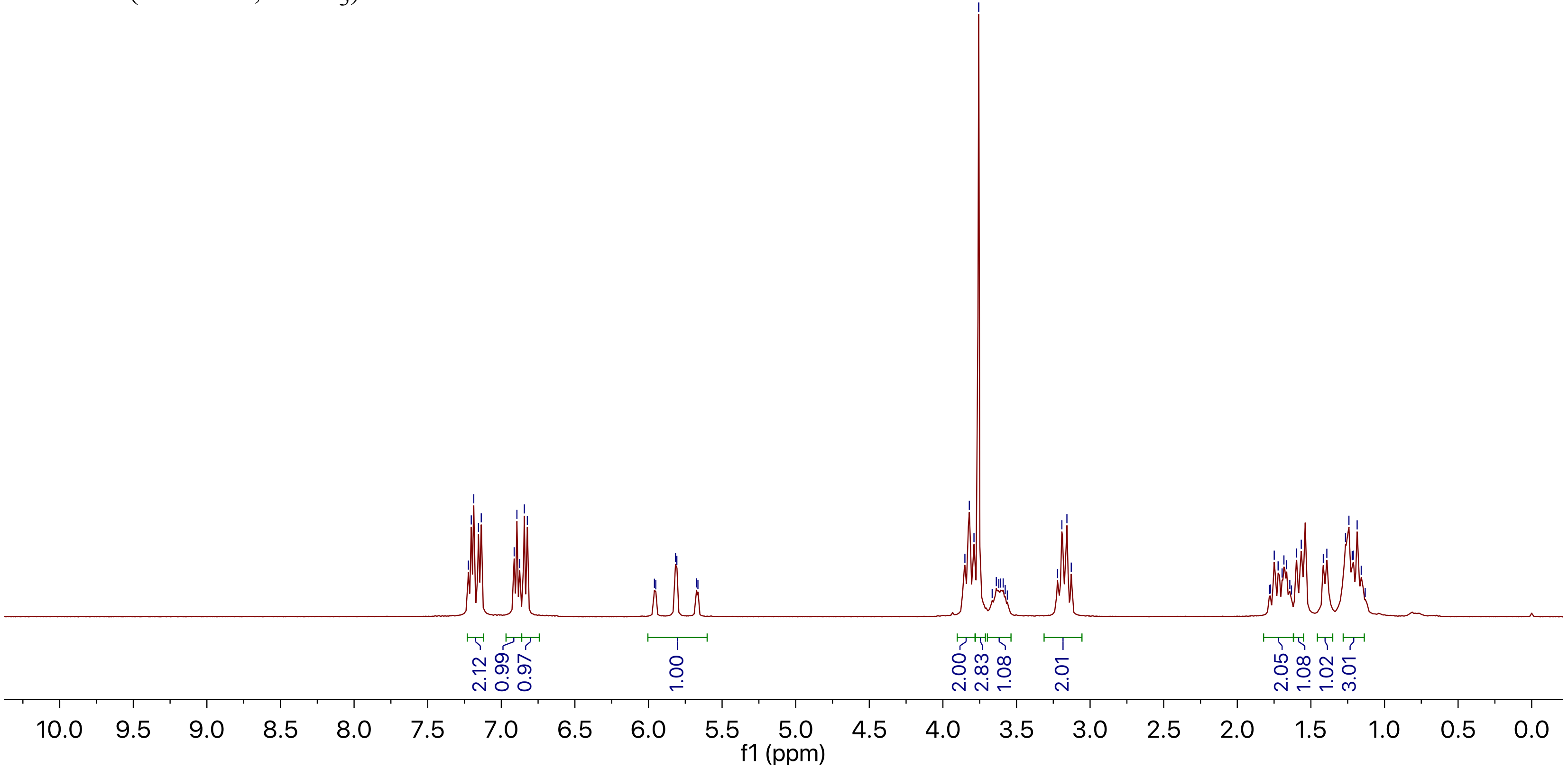

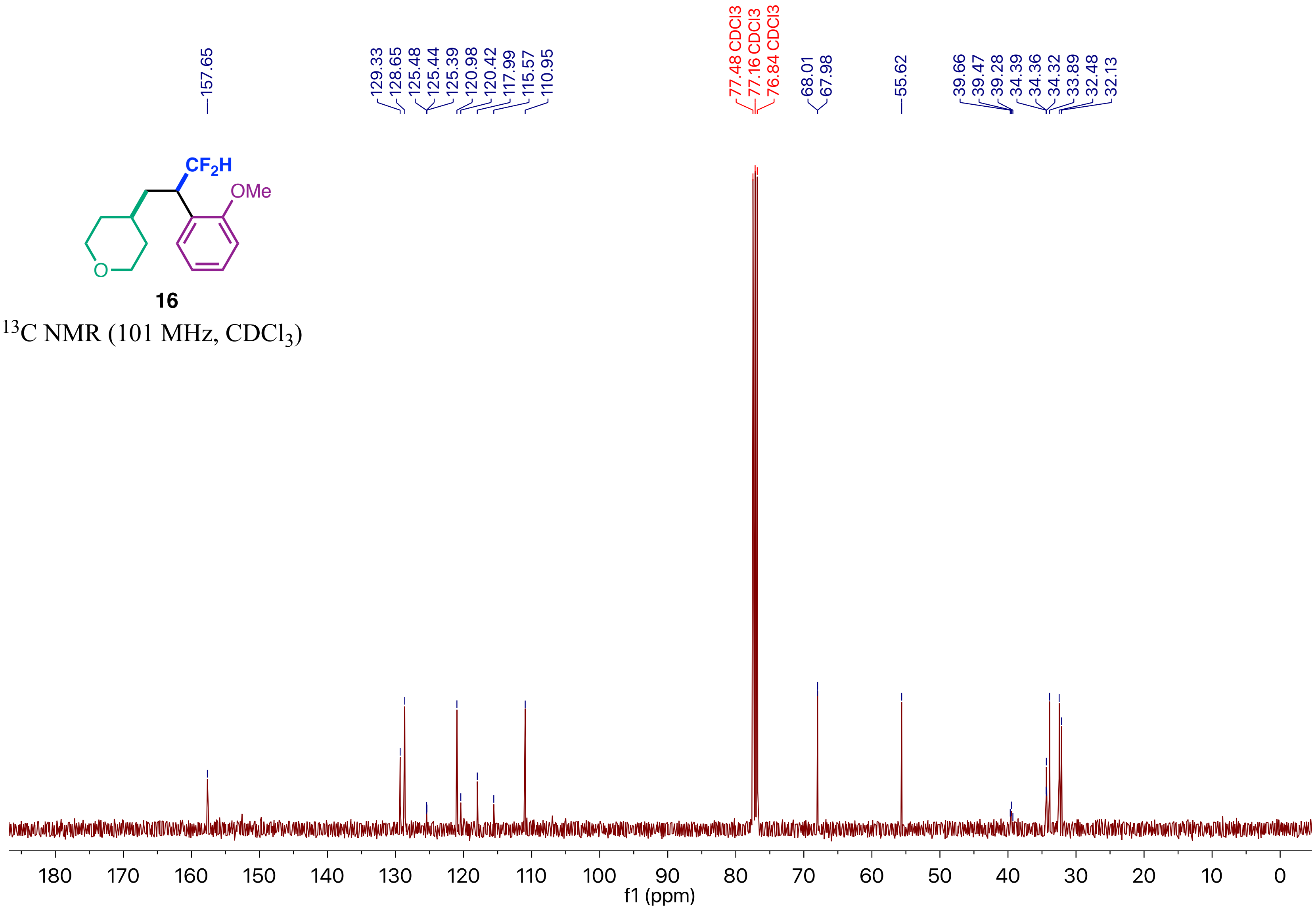

16

^{13}C NMR (101 MHz, $CDCl_3$)

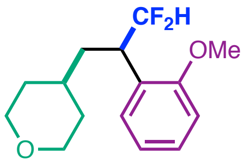

16

^{19}F NMR (376 MHz, $CDCl_3$)

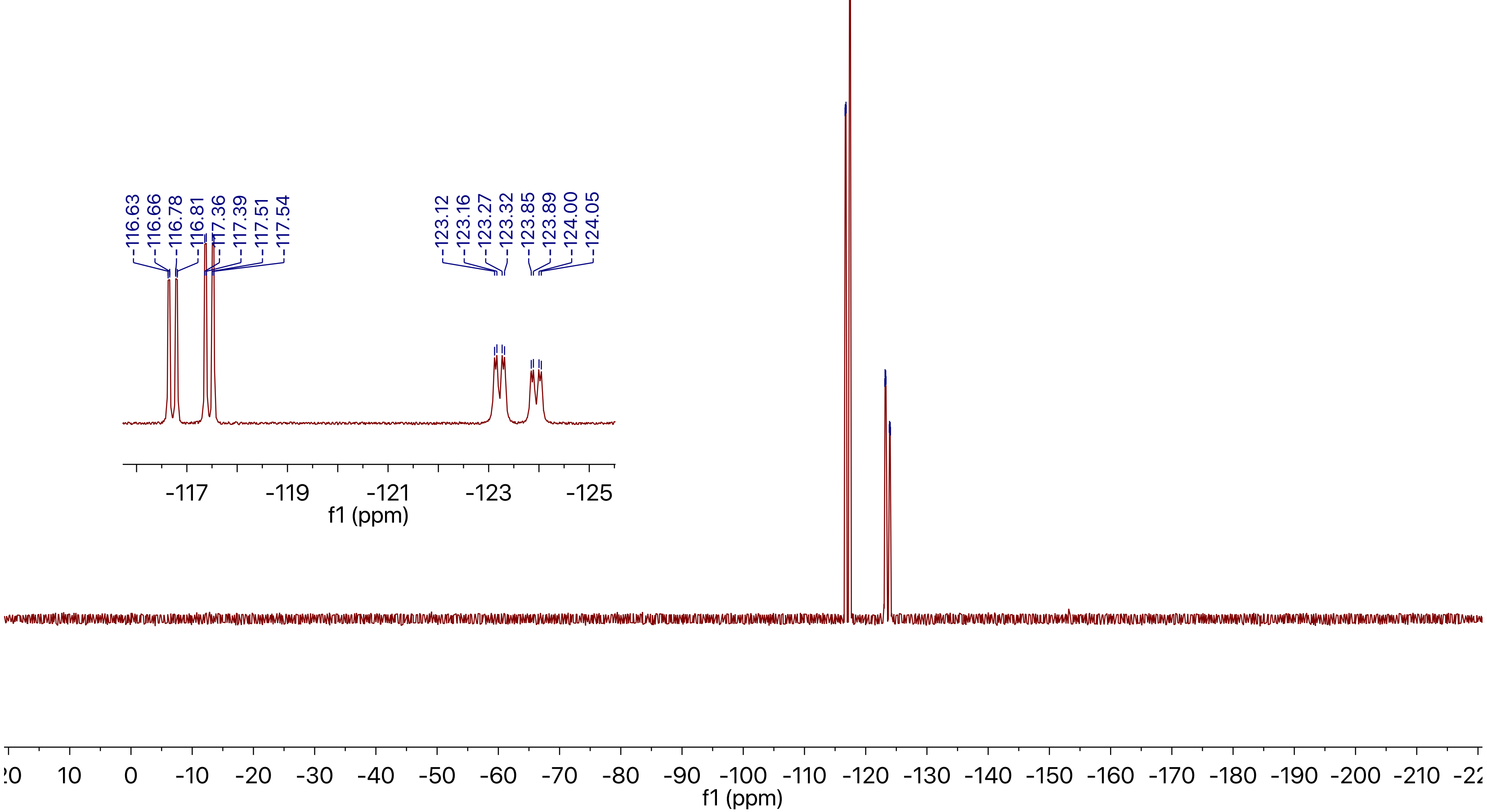

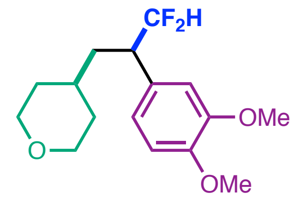

17

^{1}H NMR (400 MHz, $CDCl_3$)

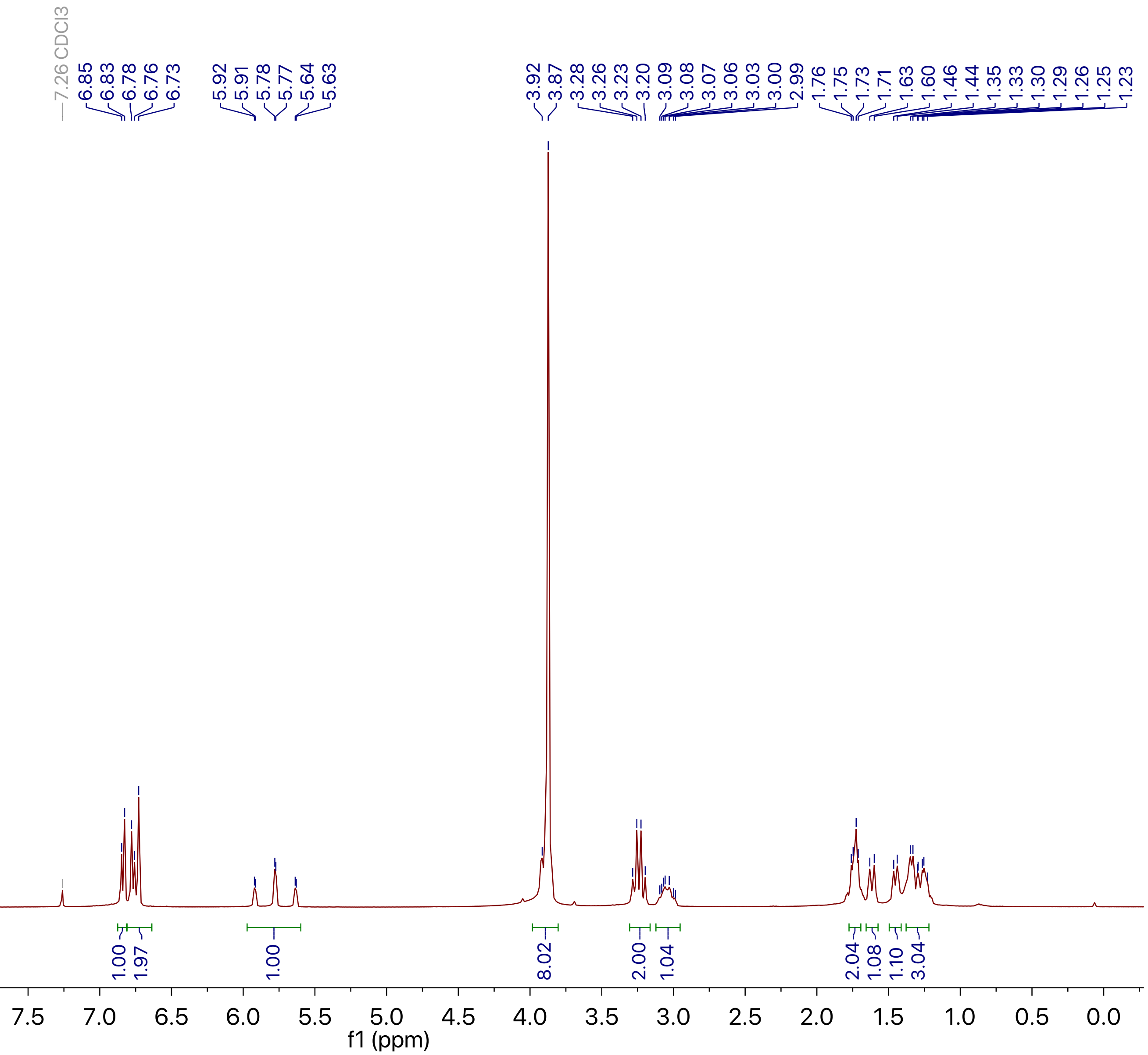

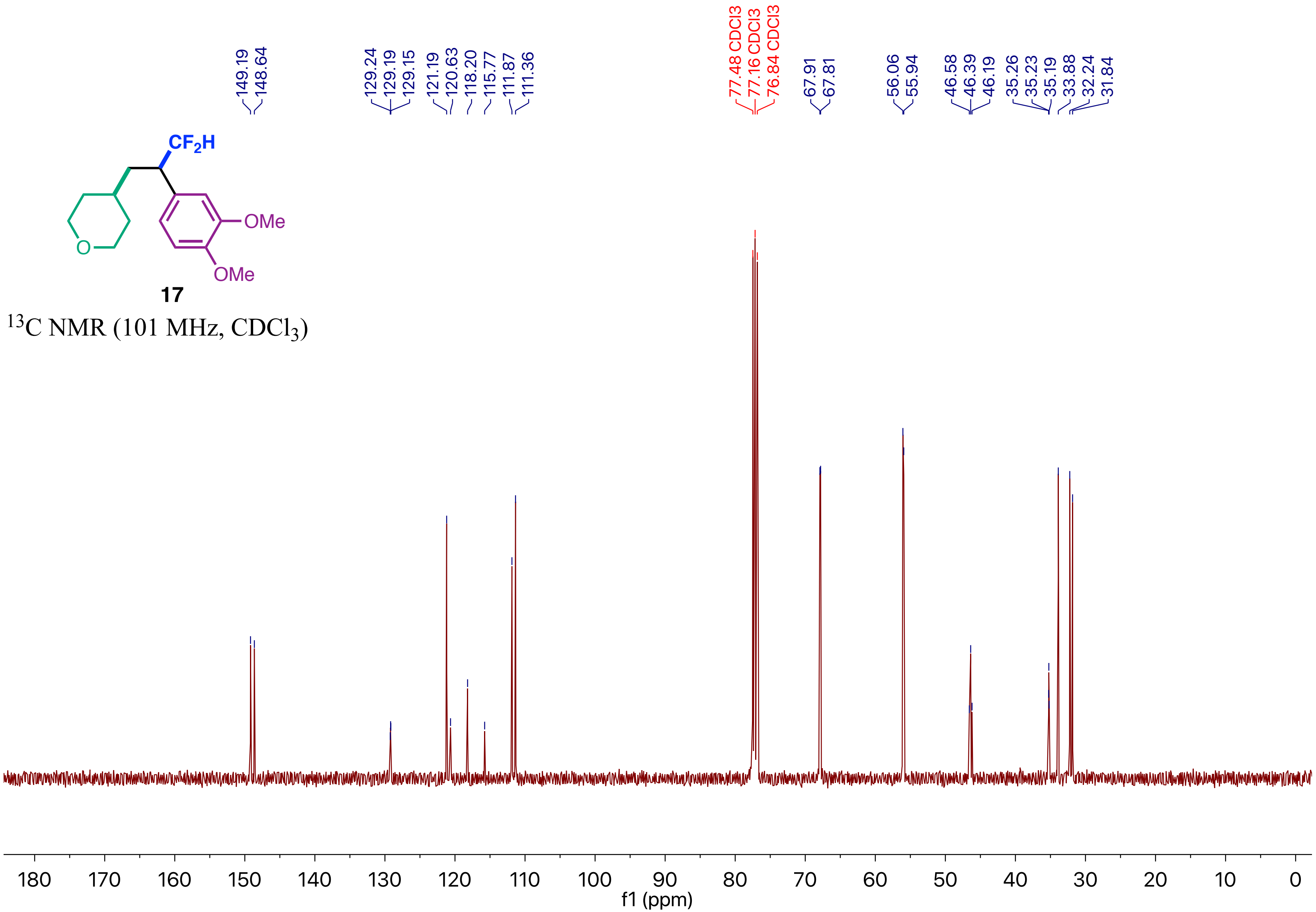

17

^{13}C NMR (101 MHz, $CDCl_3$)

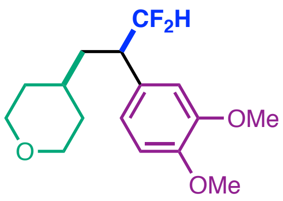

17

^{19}F NMR (376 MHz, $CDCl_3$)

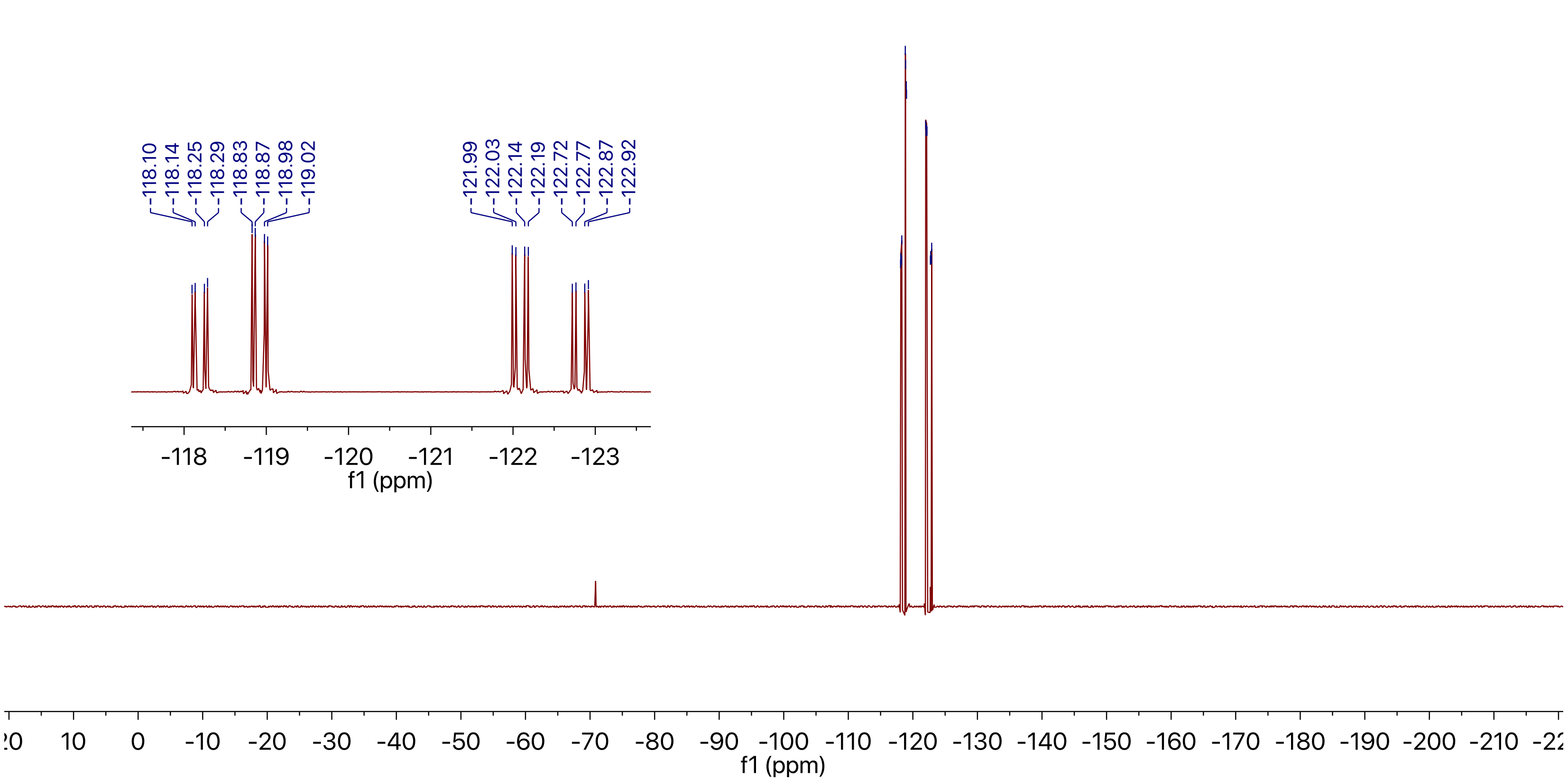

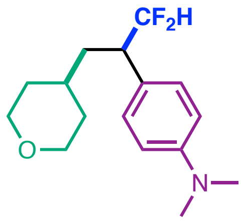

18

^{1}H NMR (400 MHz, $CDCl_3$)

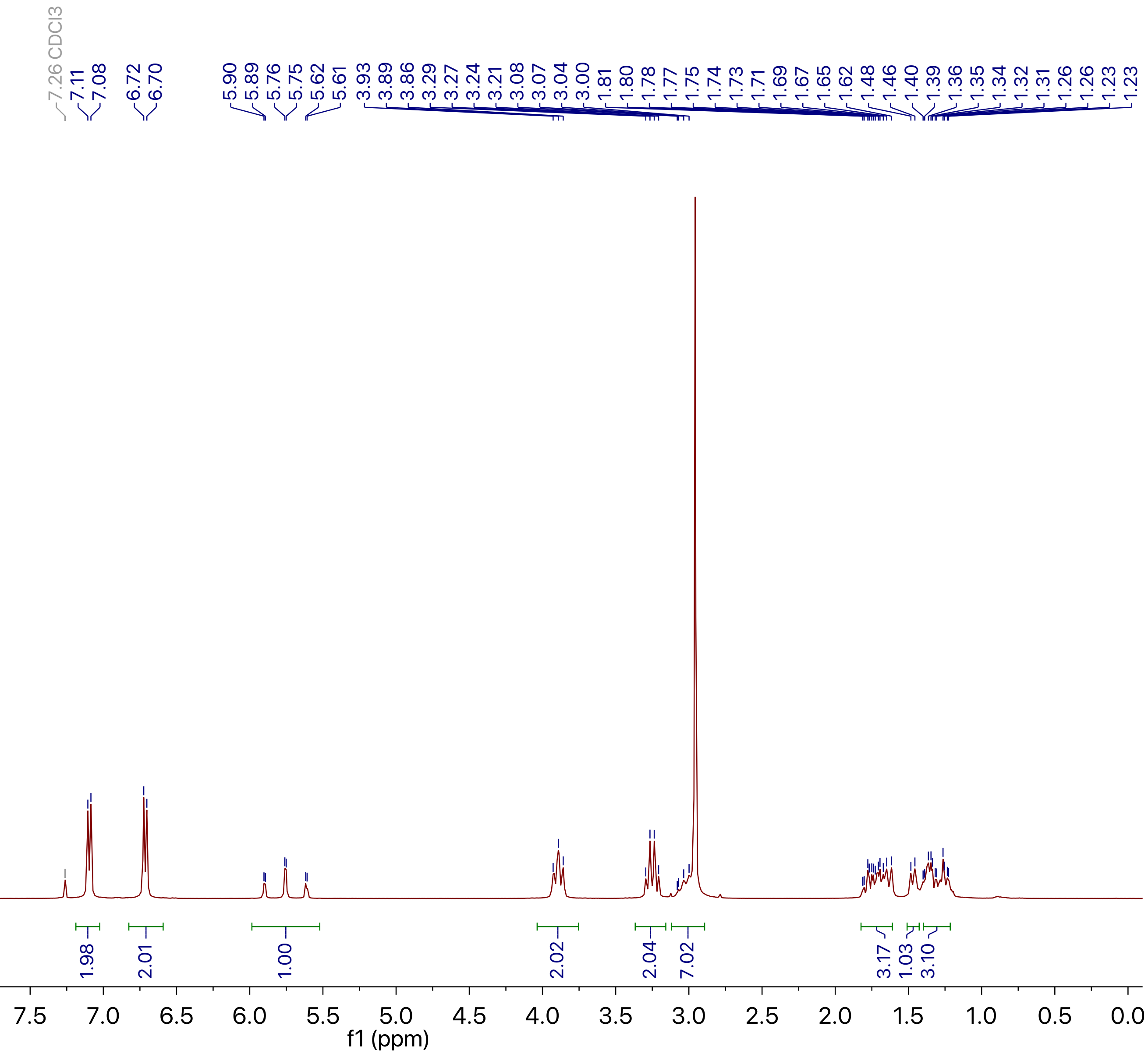

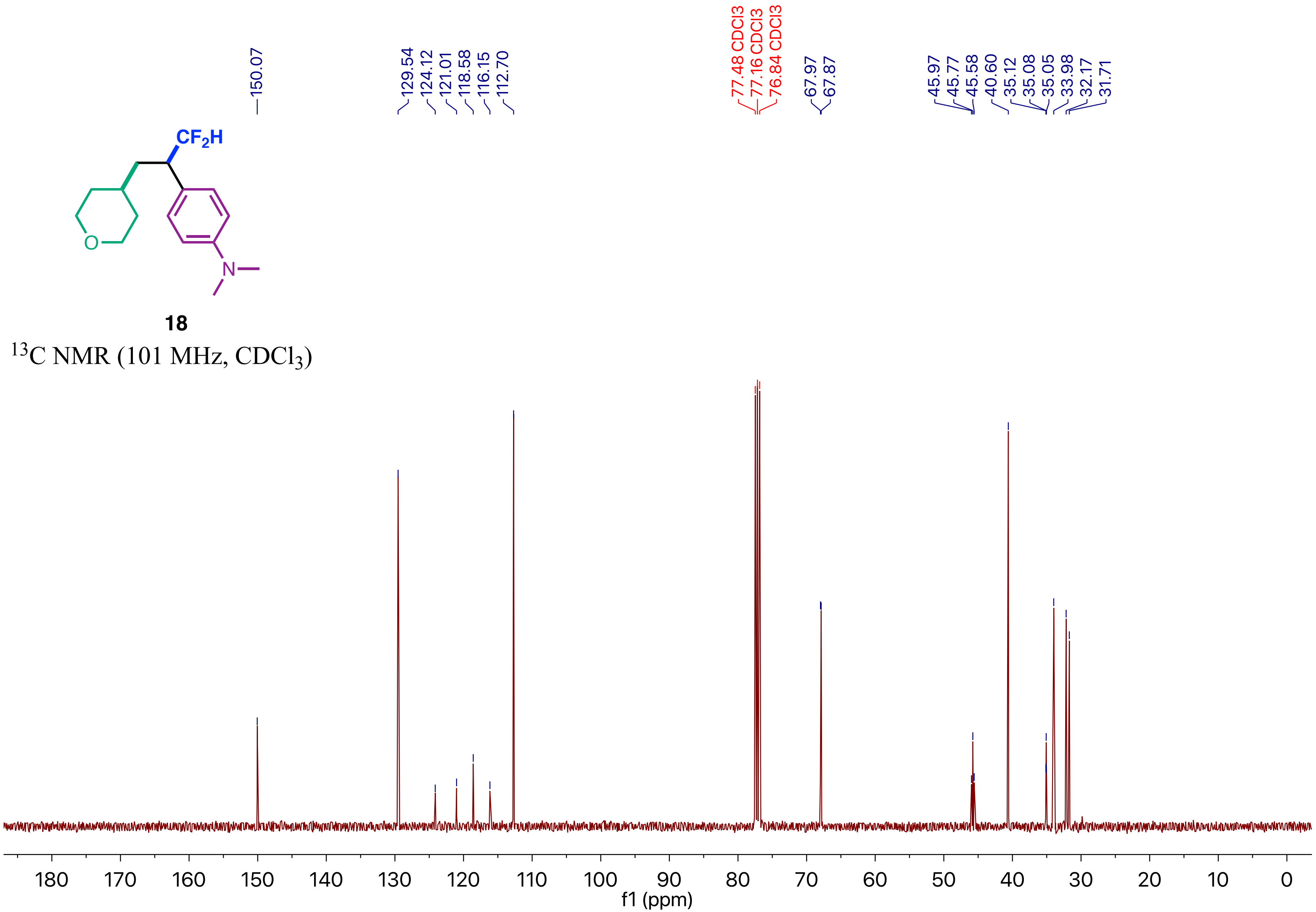

18

^{13}C NMR (101 MHz, $CDCl_3$)

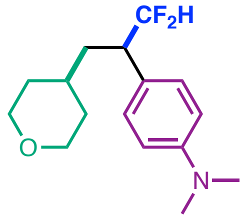

18

^{19}F NMR (376 MHz, $CDCl_3$)

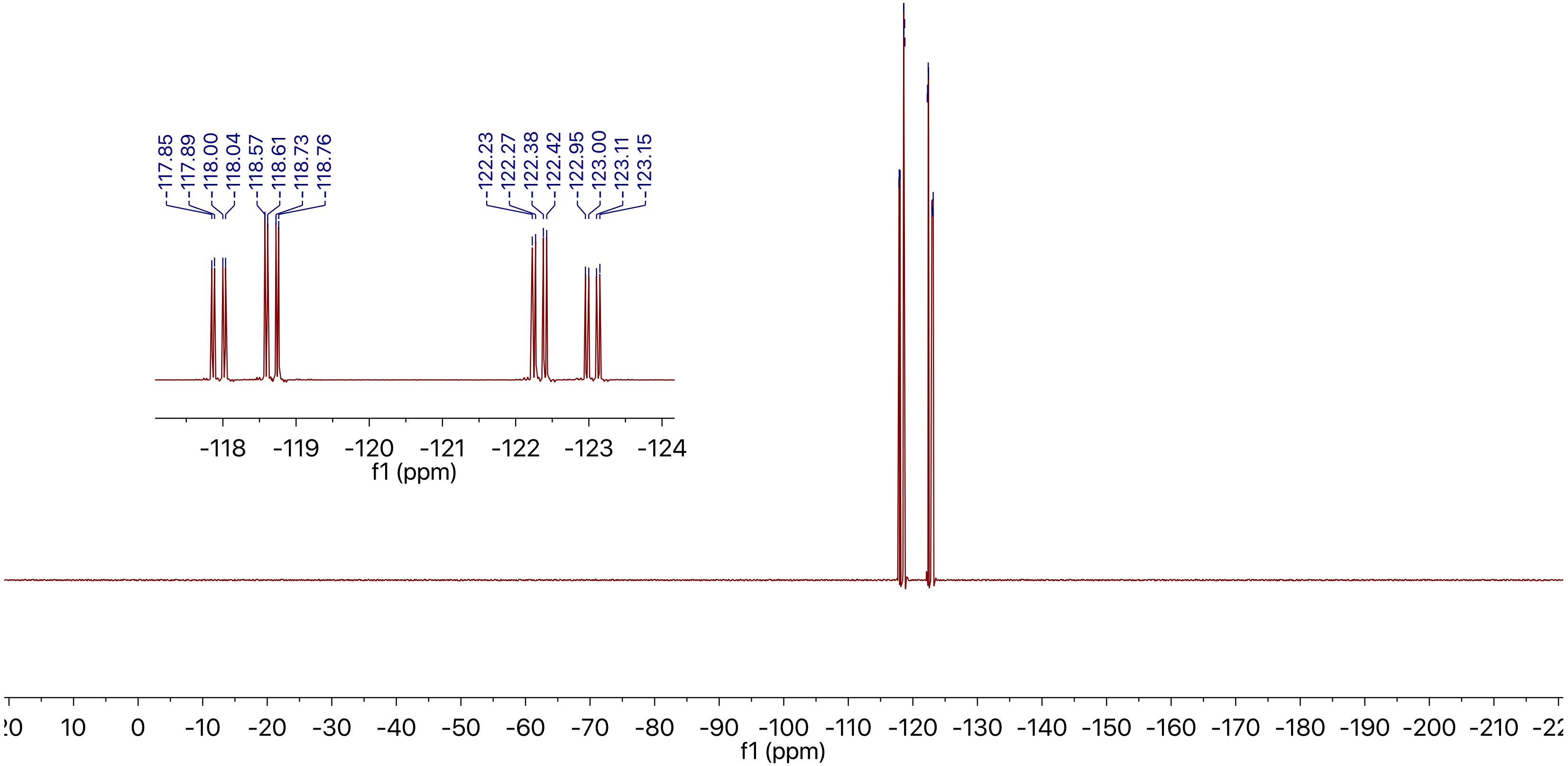

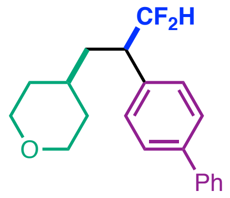

19

^1H NMR (400 MHz, $CDCl_3$)

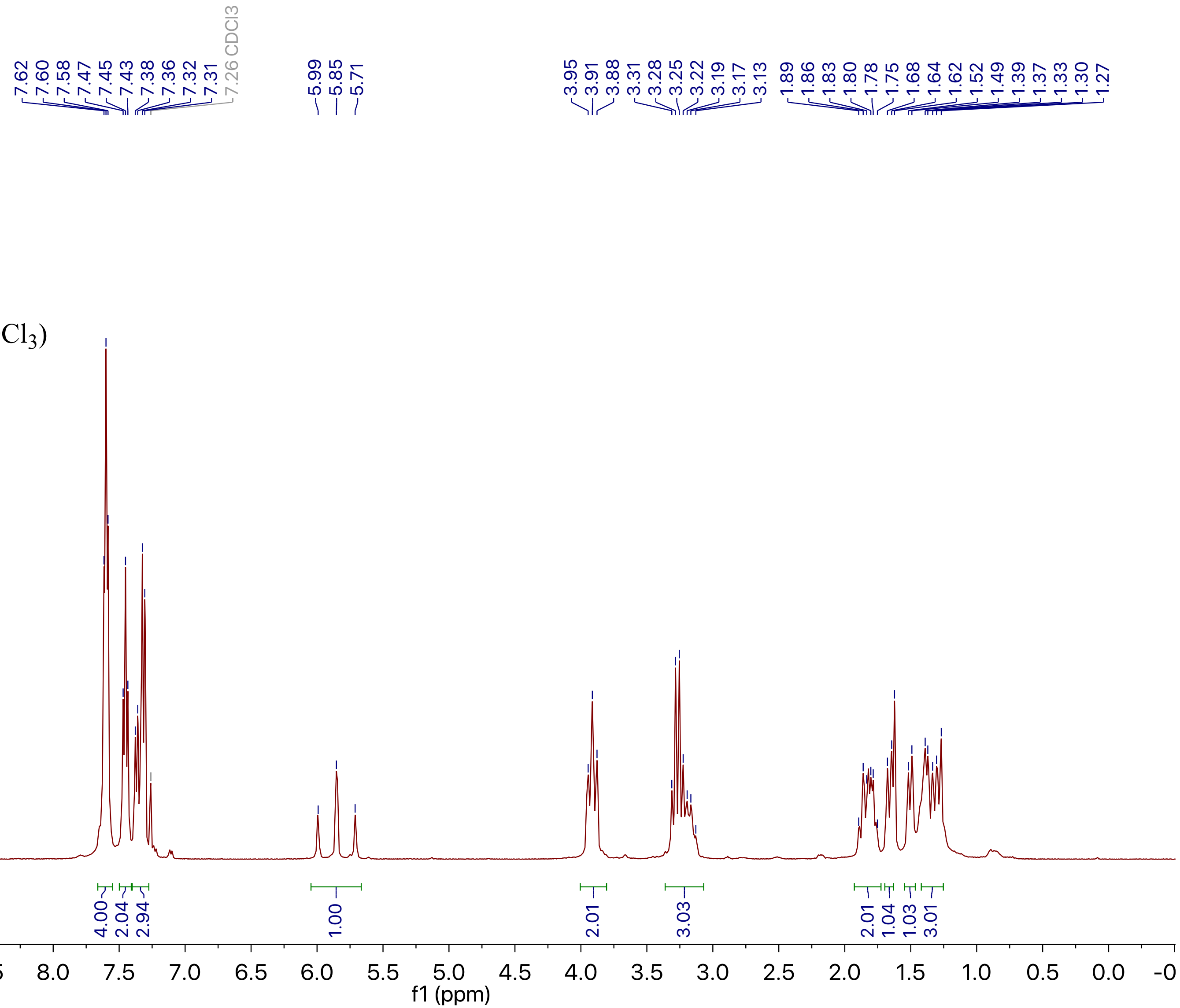

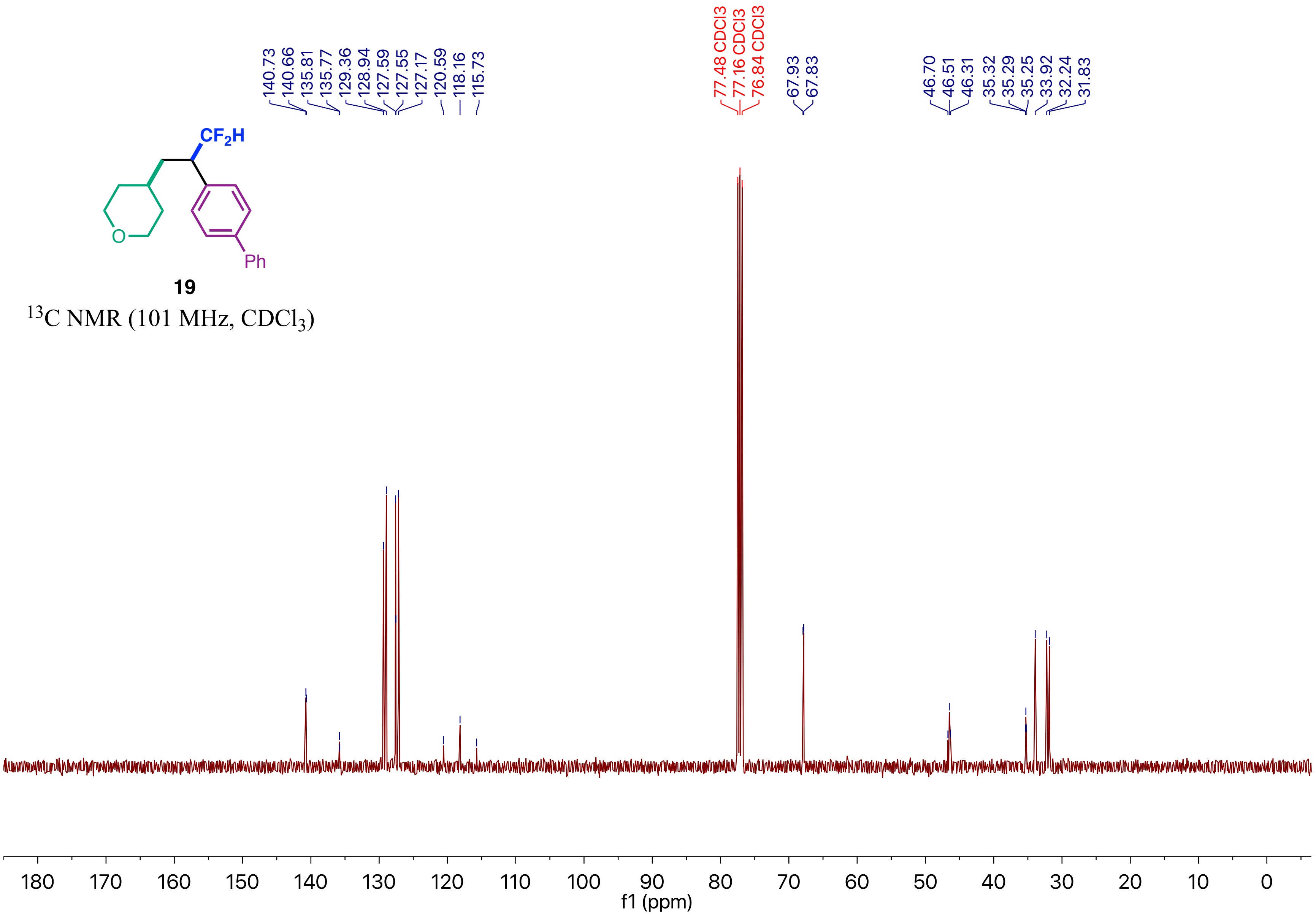

19

^{13}C NMR (101 MHz, $CDCl_3$)

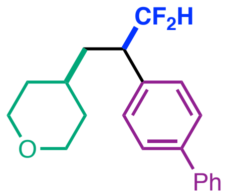

19

^{19}F NMR (376 MHz, $CDCl_3$)

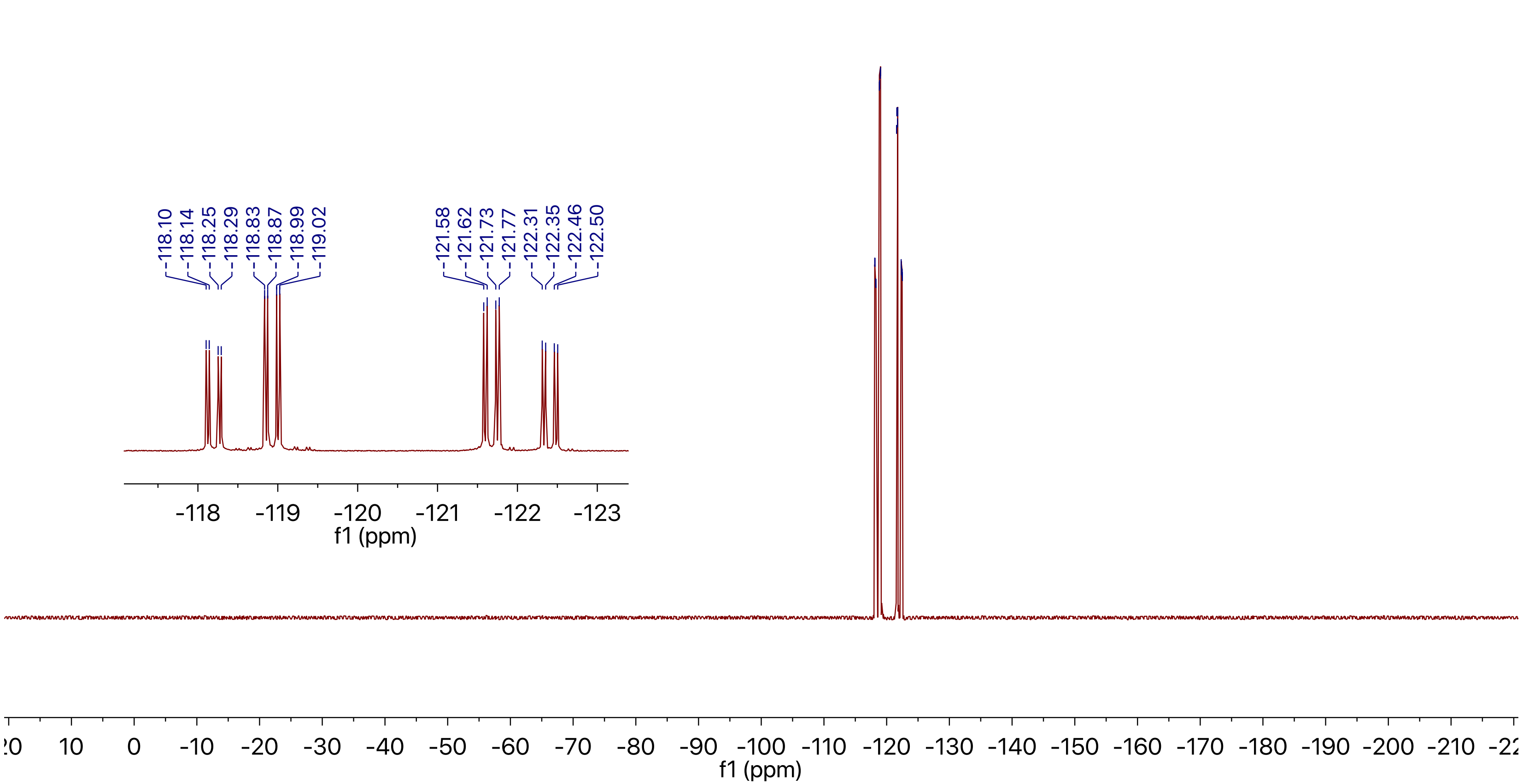

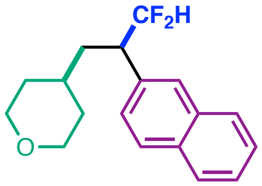

20

^{1}H NMR (400 MHz, $CDCl_3$)

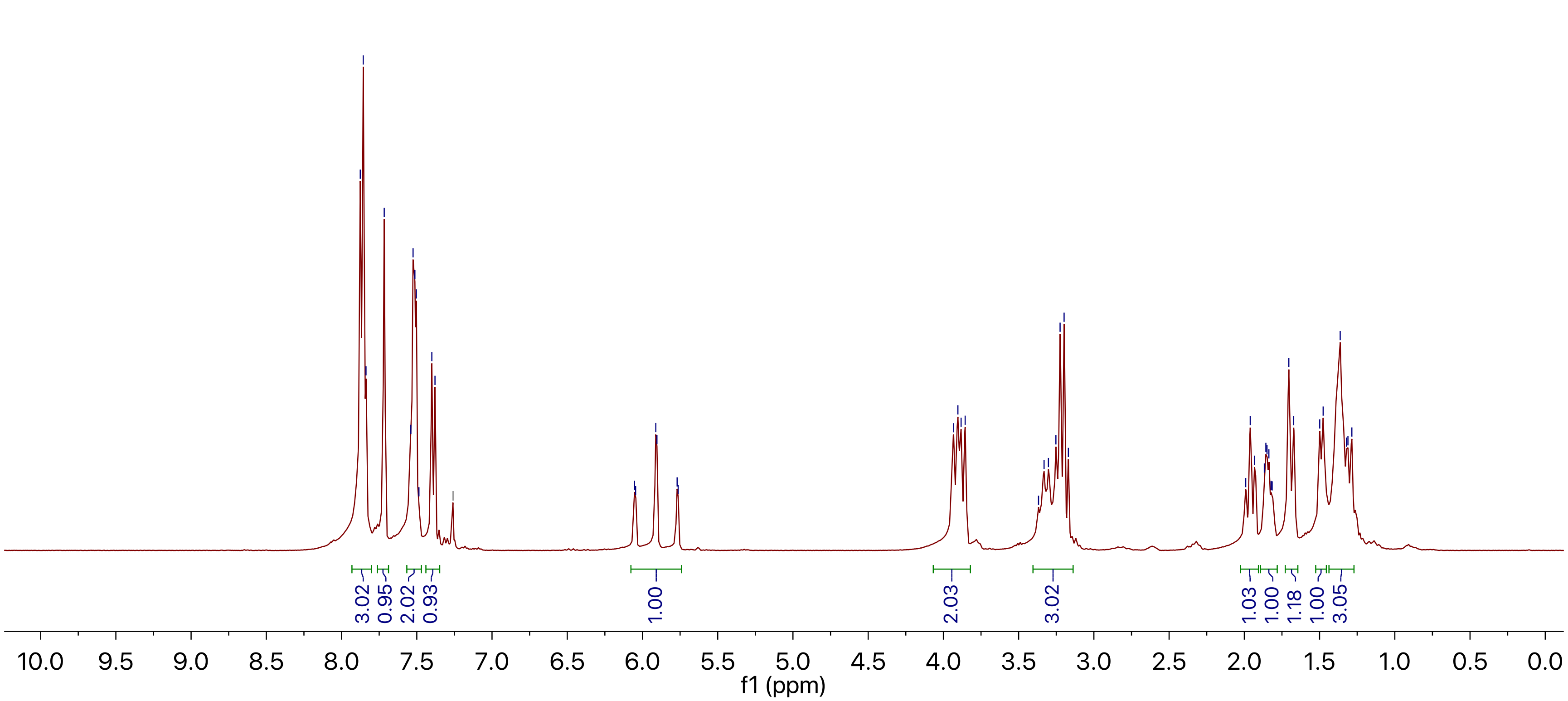

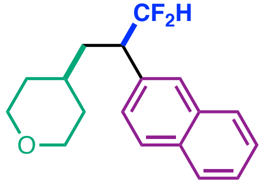

20

^{13}C NMR (101 MHz, $CDCl_3$)

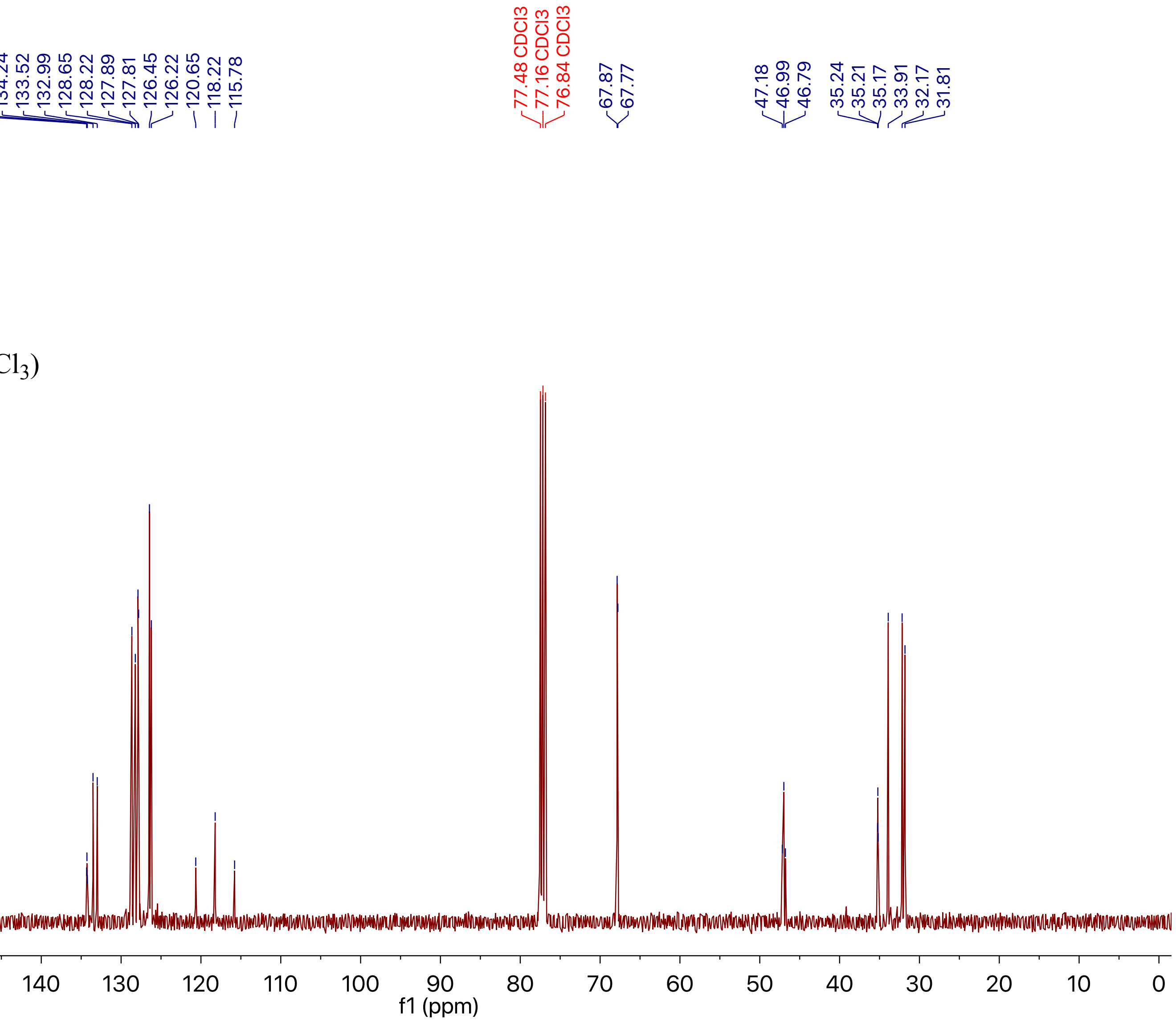

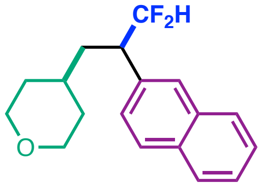

20

^{19}F NMR (376 MHz, $CDCl_3$)

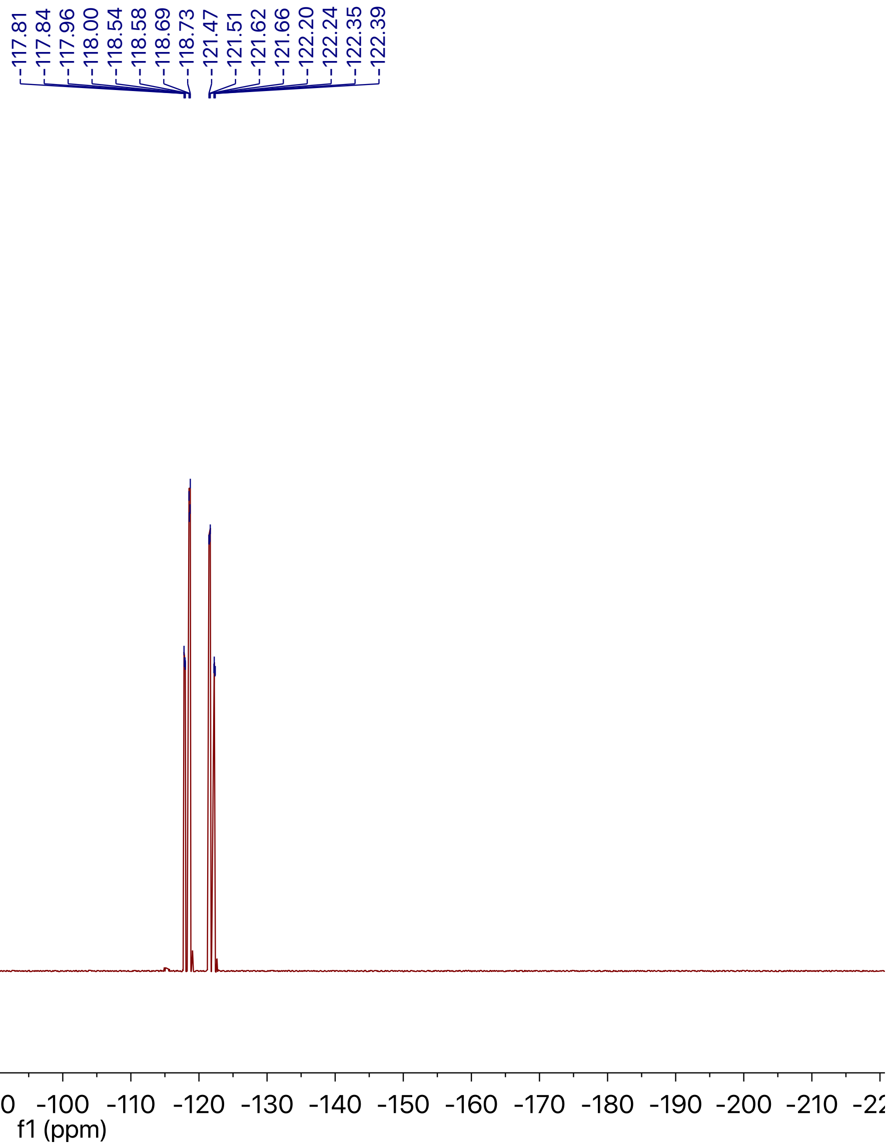

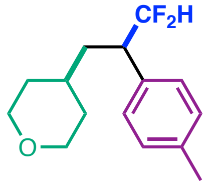

21

^{1}H NMR (400 MHz, $CDCl_3$)

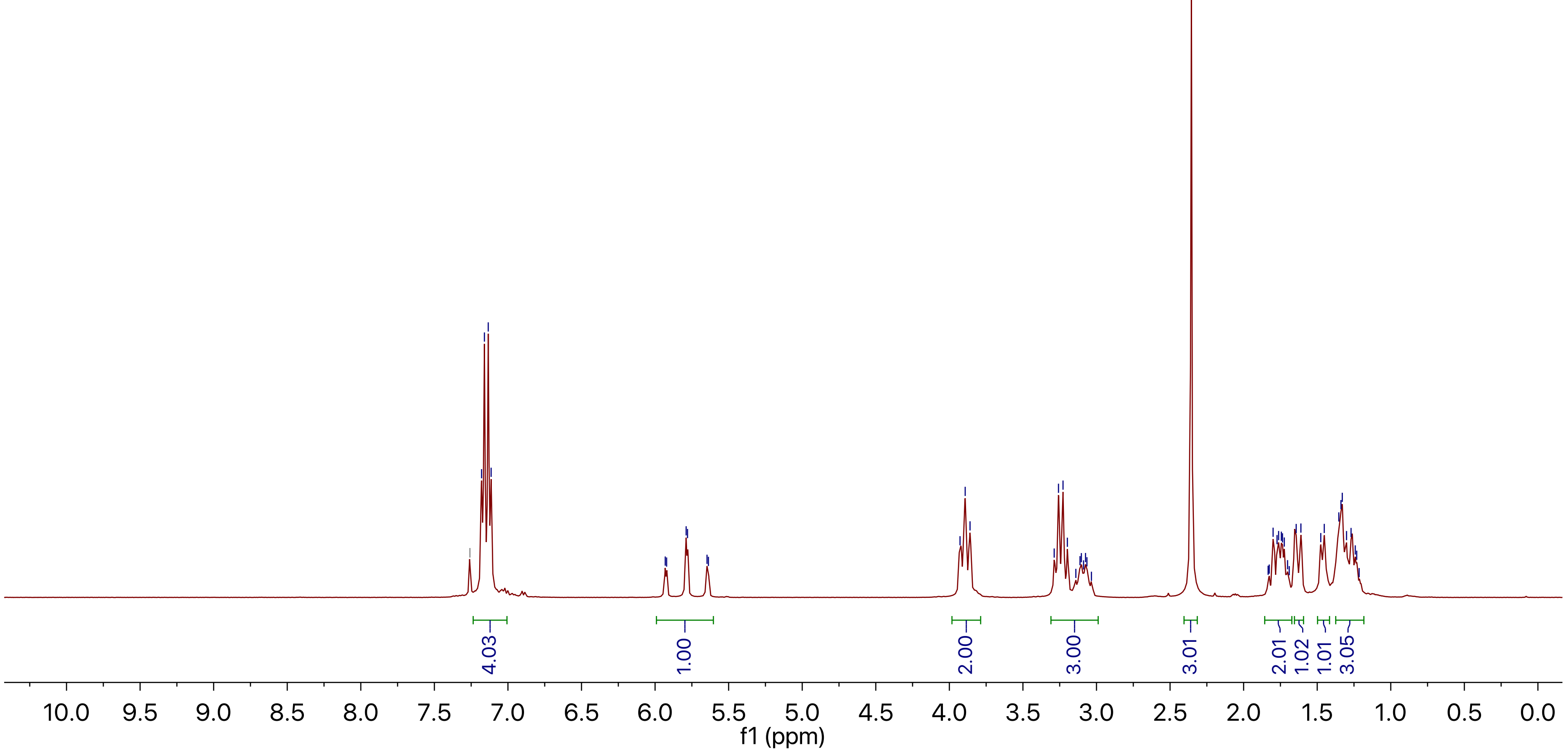

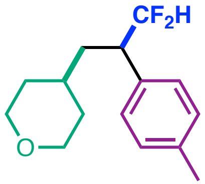

21

^{13}C NMR (101 MHz, $CDCl_3$)

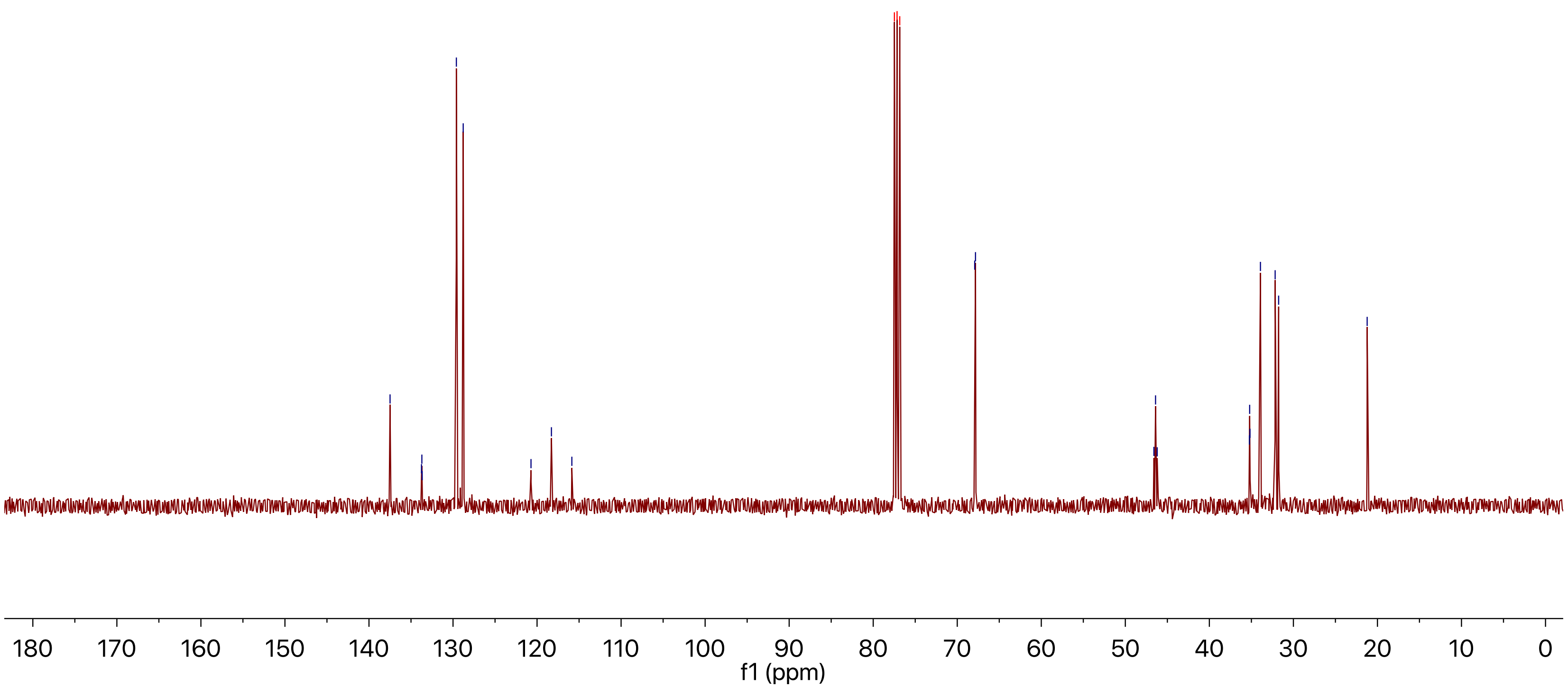

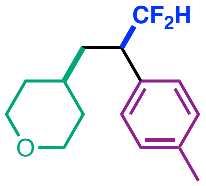

21

^{19}F NMR (376 MHz, $CDCl_3$)

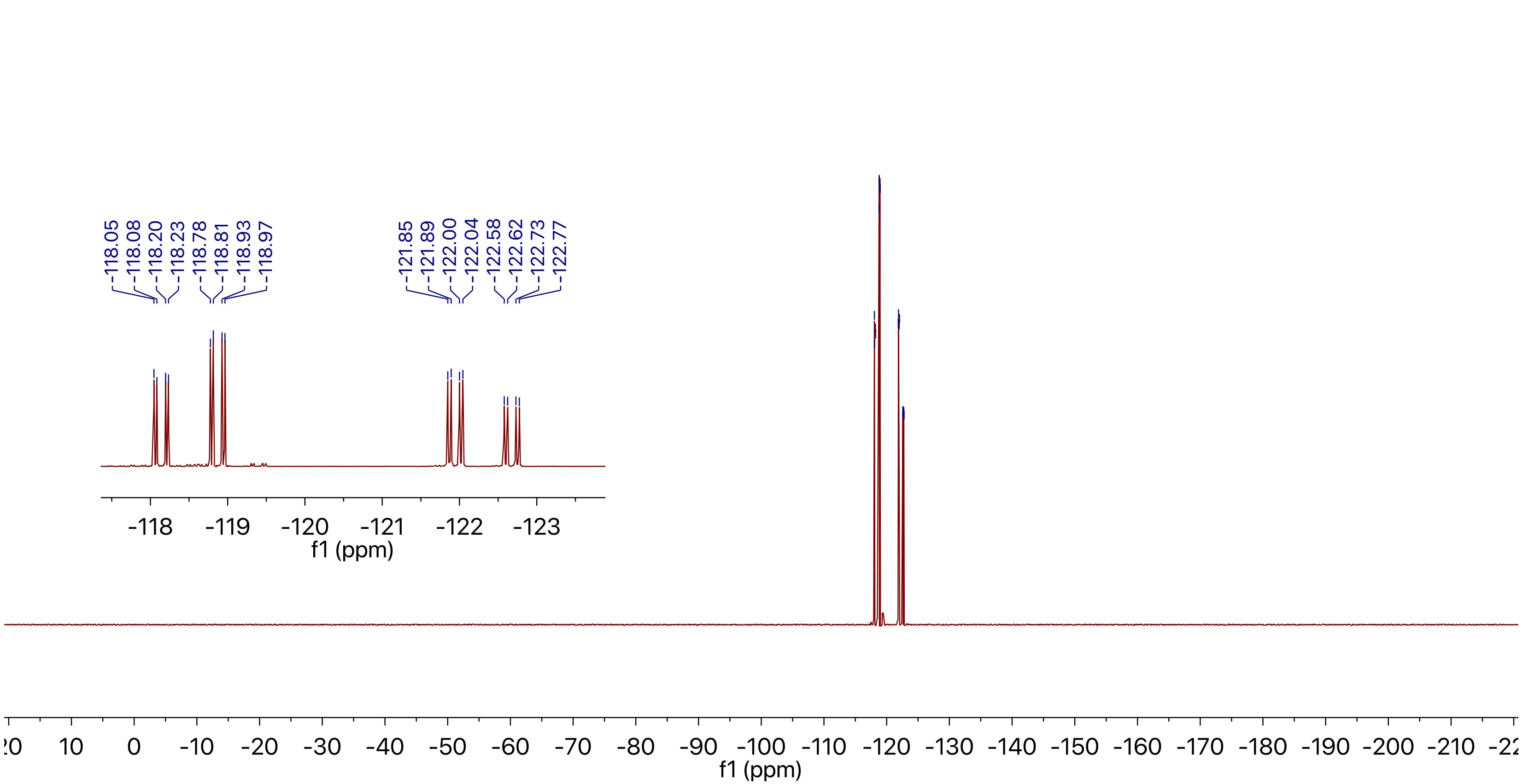

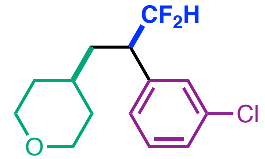

22

^{1}H NMR (400 MHz, $CDCl_3$)

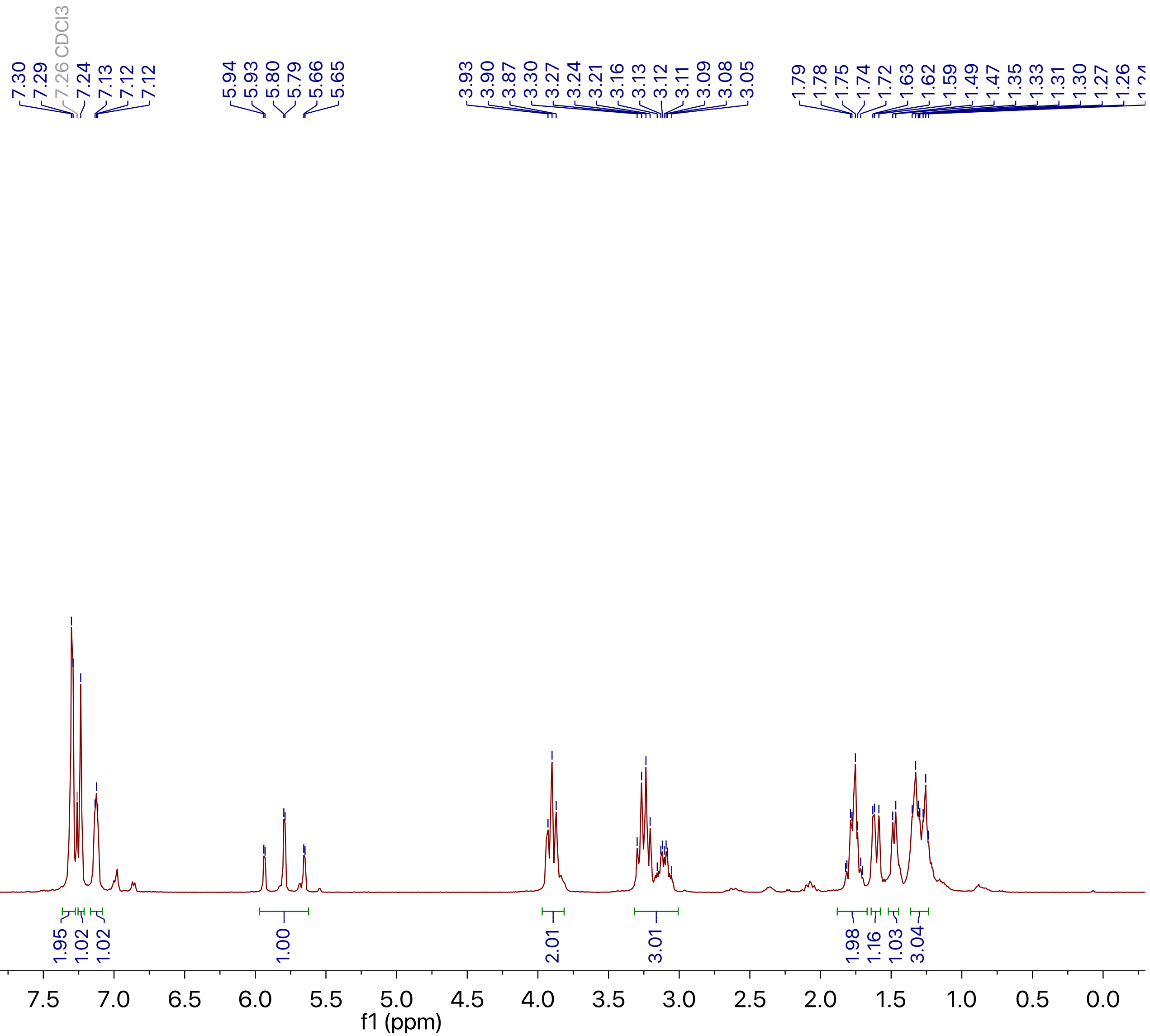

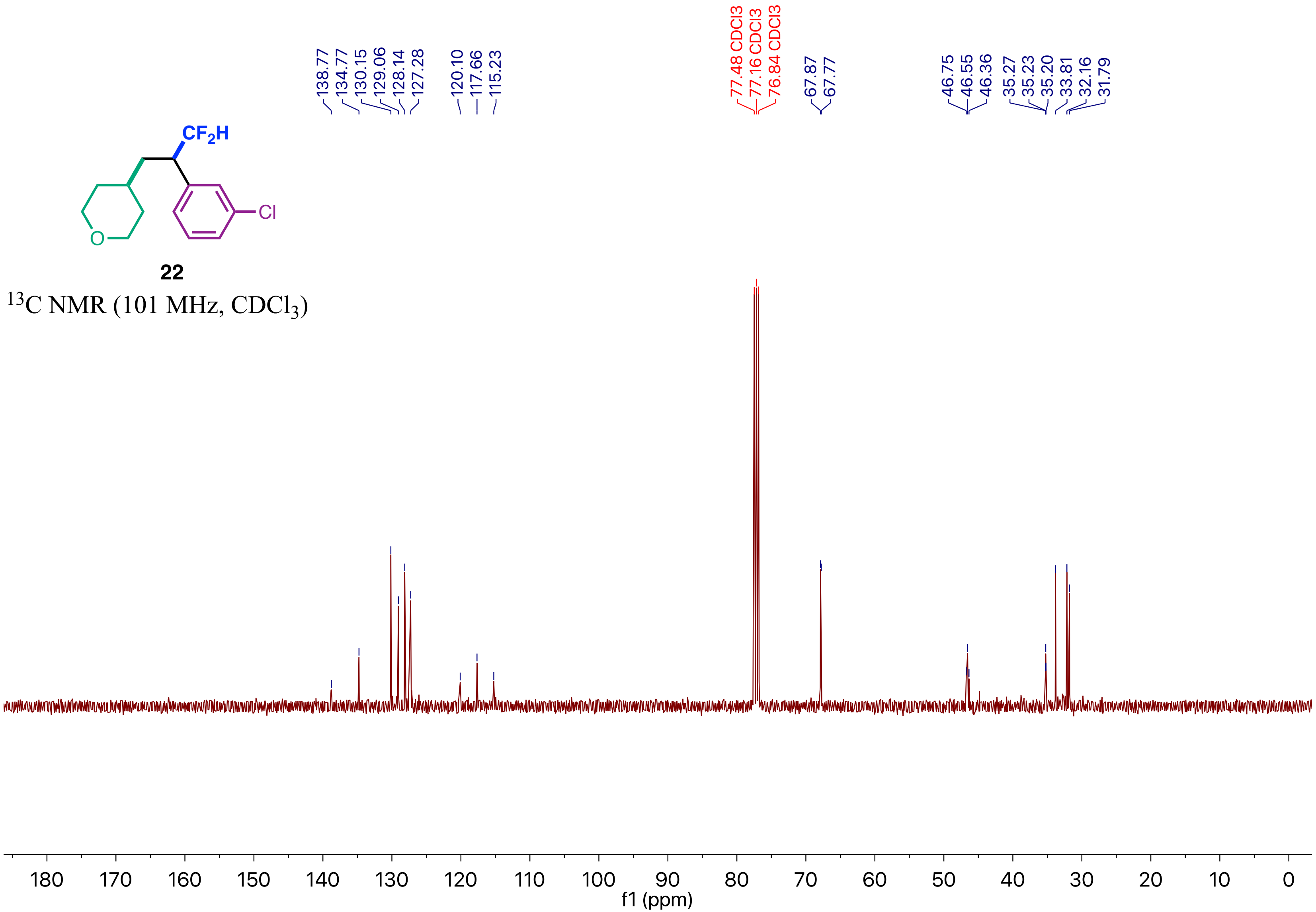

22

^{13}C NMR (101 MHz, $CDCl_3$)

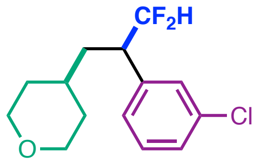

22

^{19}F NMR (376 MHz, $CDCl_3$)

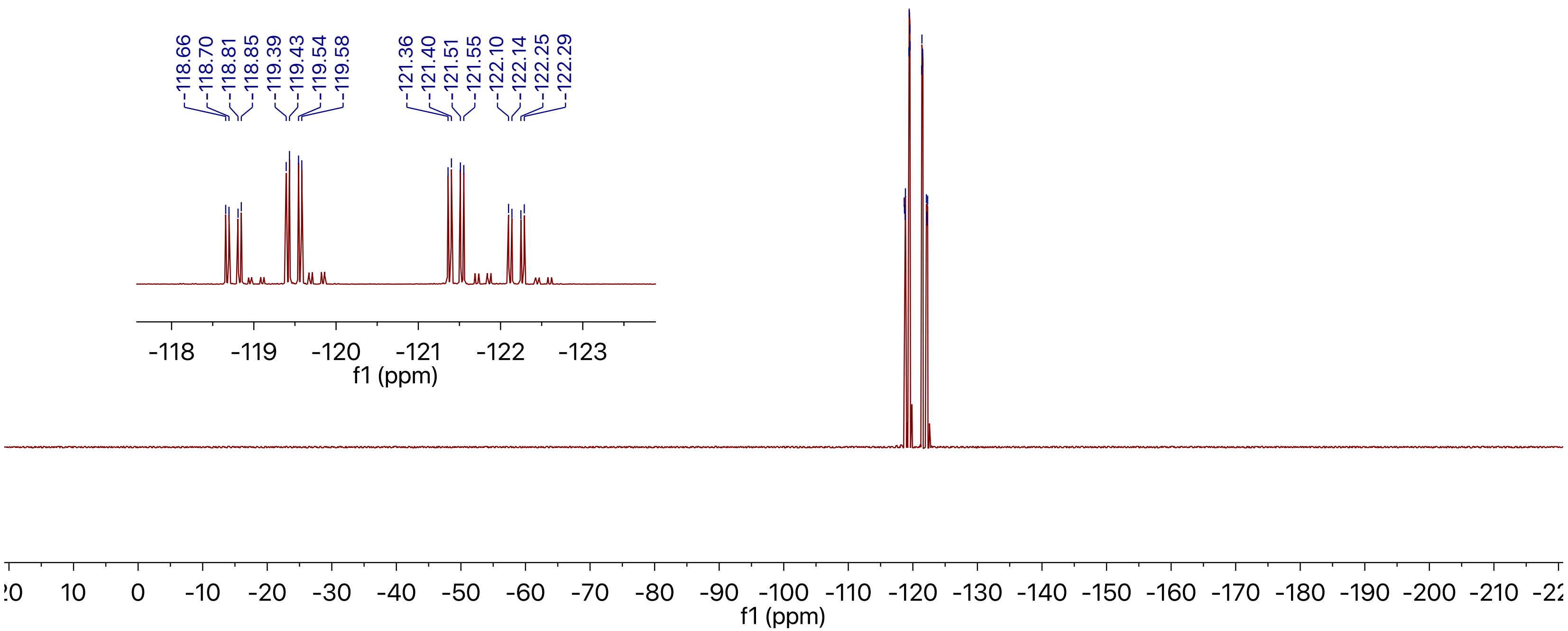

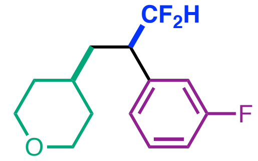

23

^{1}H NMR (400 MHz, $CDCl_3$)

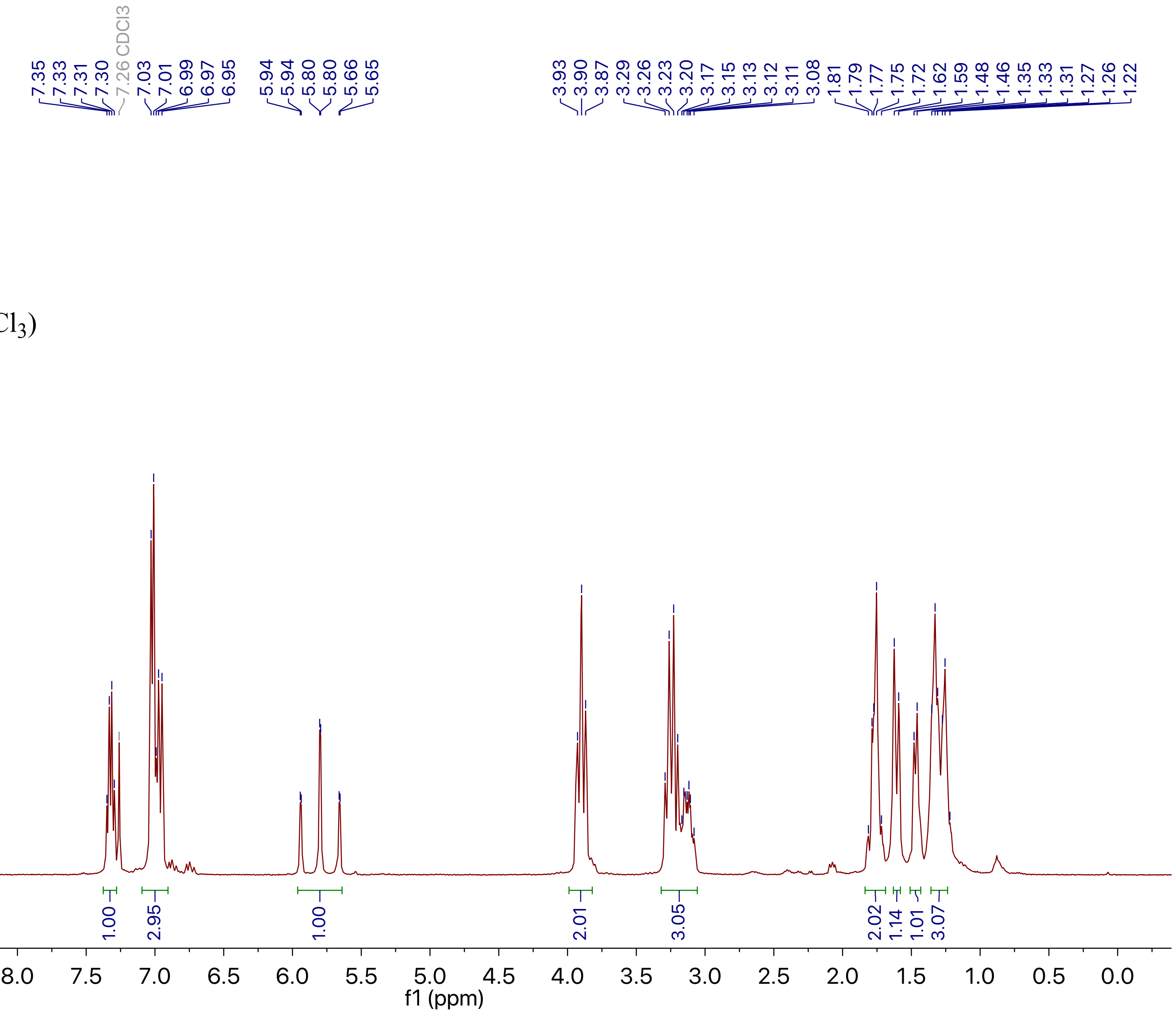

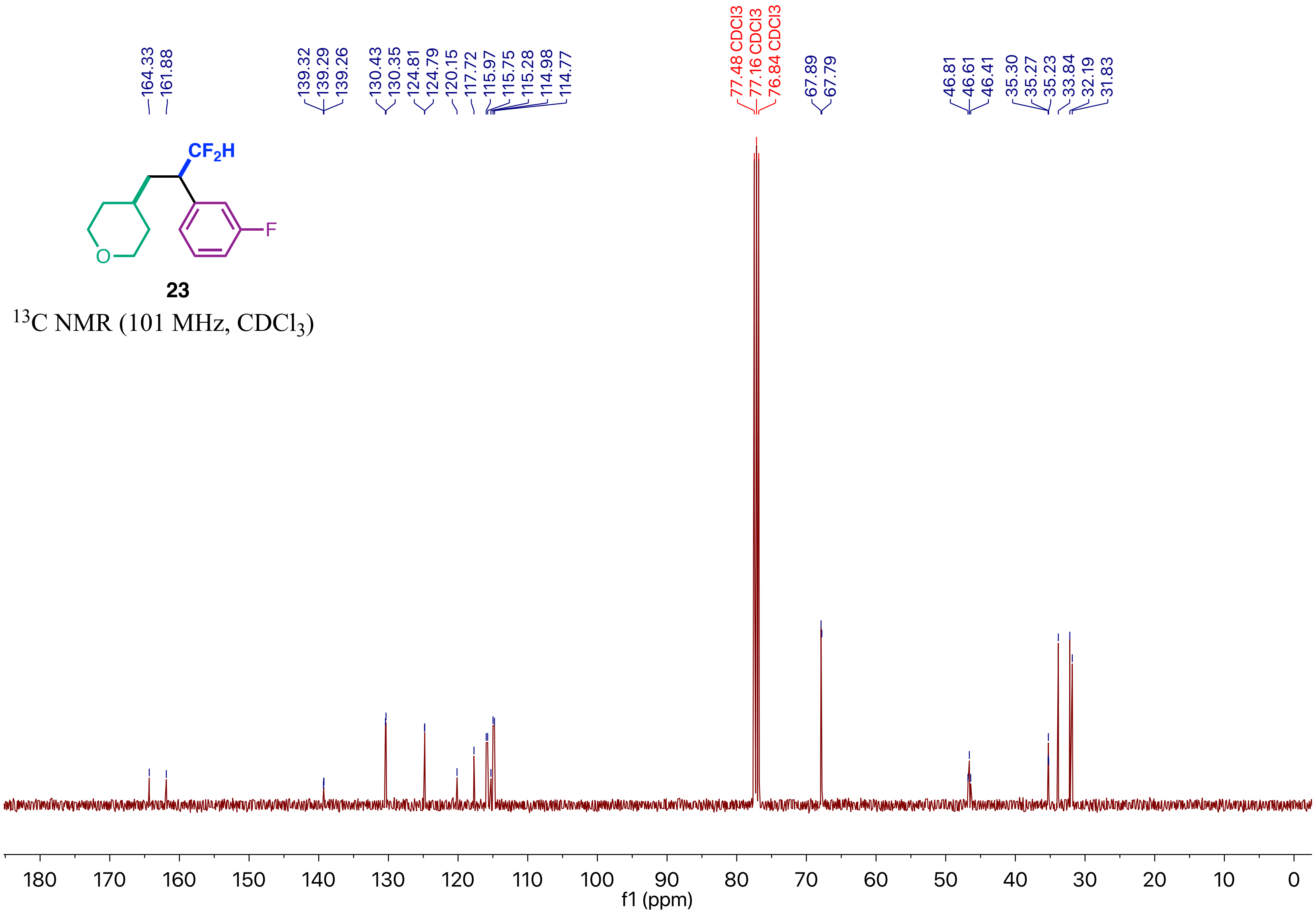

23

^{13}C NMR (101 MHz, $CDCl_3$)

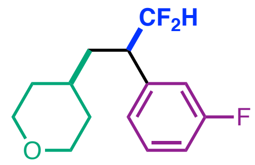

23

^{19}F NMR (376 MHz, CDCl$_3$)

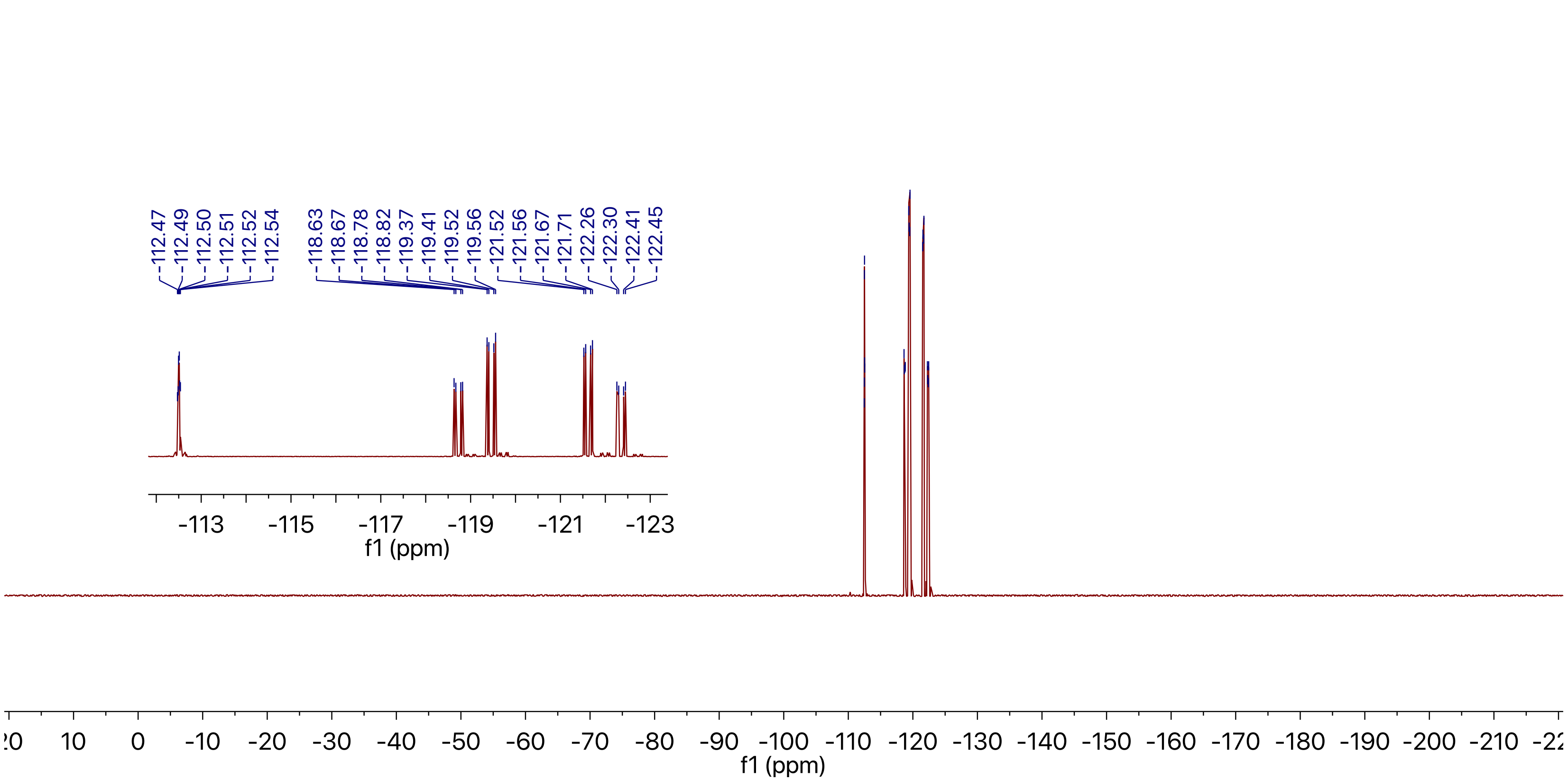

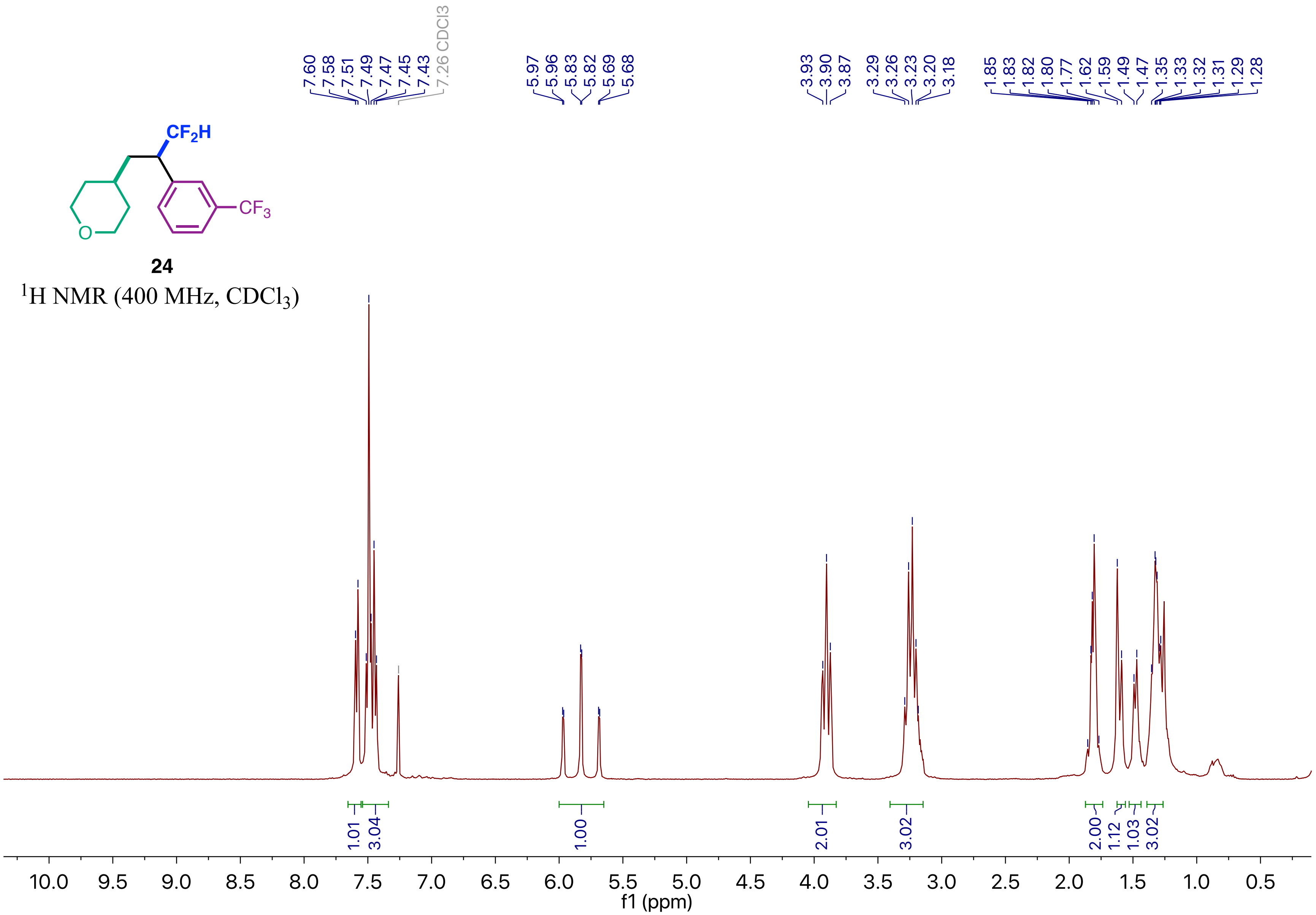

CF2H
CF3
O
24
1H NMR (400 MHz, CDCl3)
7.60 7.58 7.51 7.49 7.47 7.45 7.43 7.26 CDCl3
5.97 5.96 5.83 5.82 5.69 5.68
3.93 3.90 3.87 3.29 3.26 3.23 3.20 3.18
1.85 1.83 1.82 1.80 1.77 1.62 1.59 1.49 1.47 1.35 1.33 1.32 1.31 1.29 1.28
1.01 3.04 1.00 2.01 3.02 2.00 1.12 1.03 3.02
10.0 9.5 9.0 8.5 8.0 7.5 7.0 6.5 6.0 5.5 5.0 4.5 4.0 3.5 3.0 2.5 2.0 1.5 1.0 0.5
f1 (ppm)

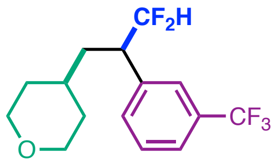

24

^{13}C NMR (101 MHz, $CDCl_3$)

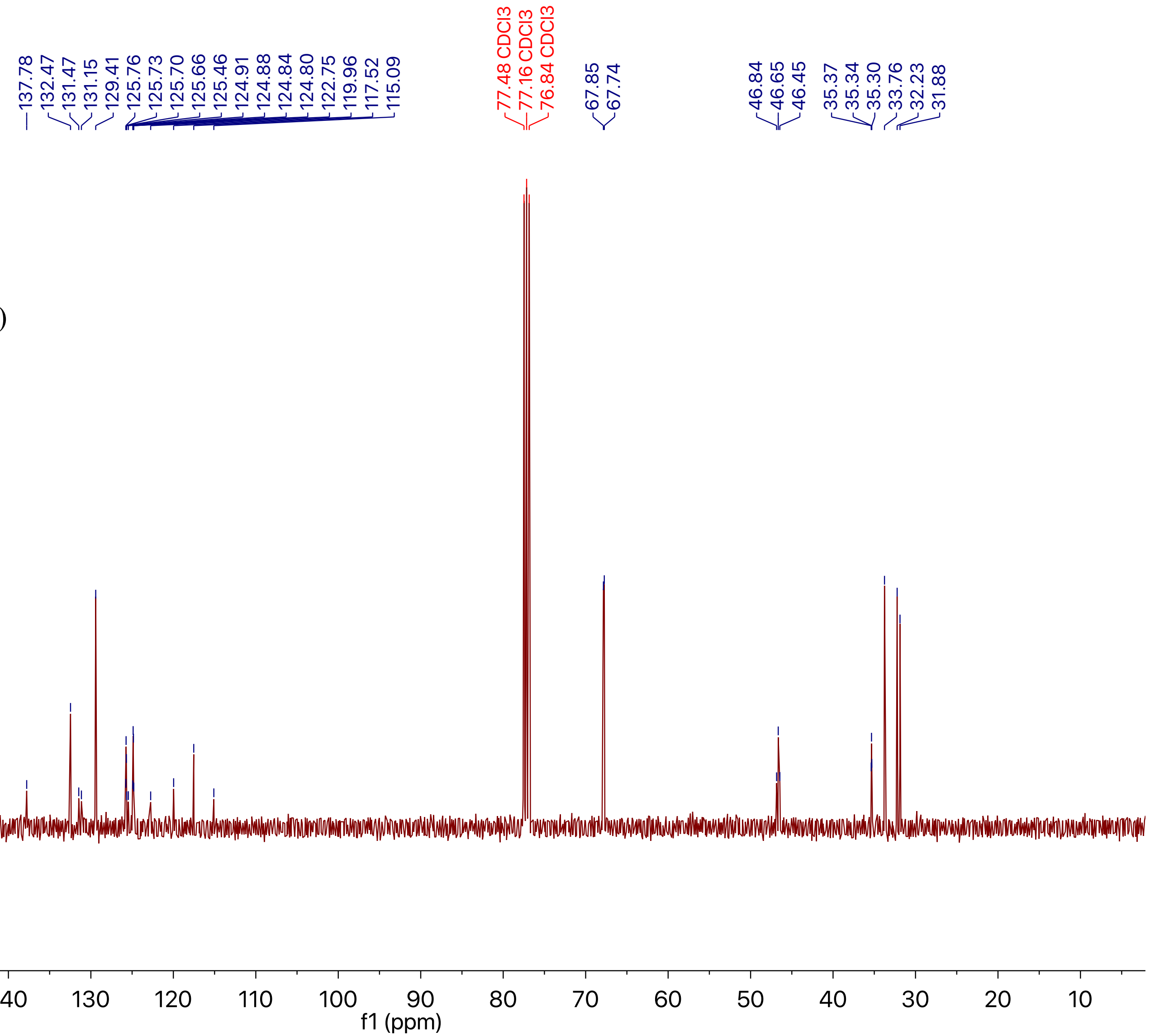

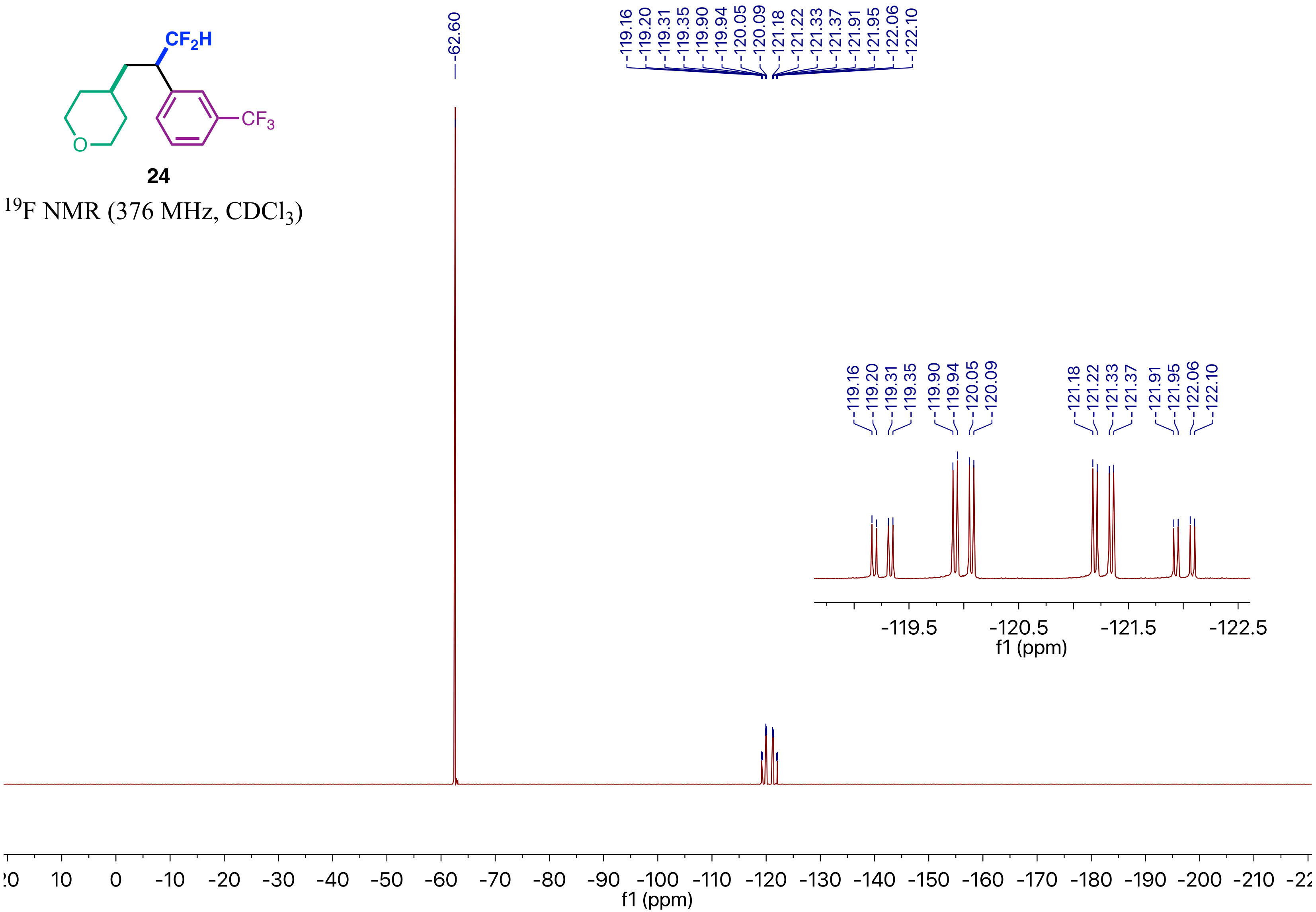
CF2H
CF3
O
24
19F NMR (376 MHz, CDCl3)
-62.60
-119.16
-119.20
-119.31
-119.35
-119.90
-119.94
-120.05
-120.09
-121.18
-121.22
-121.33
-121.37
-121.91
-121.95
-122.06
-122.10
-119.5
-120.5
-121.5
-122.5
f1 (ppm)
10
0
-10
-20
-30
-40
-50
-60
-70
-80
-90
-100
-110
-120
-130
-140
-150
-160
-170
-180
-190
-200
-210
f1 (ppm)

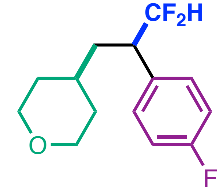

25

^{1}H NMR (400 MHz, $CDCl_3$)

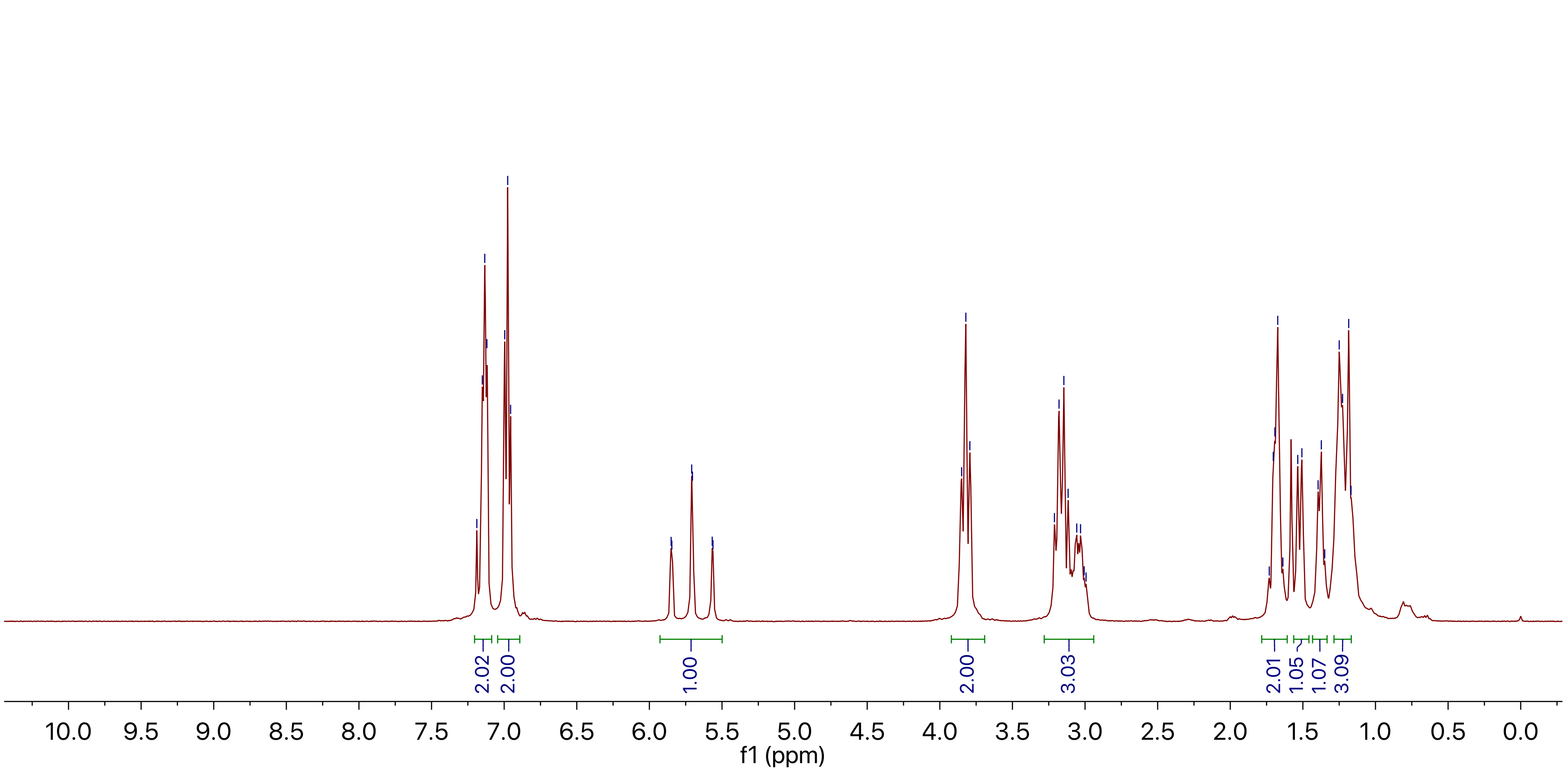

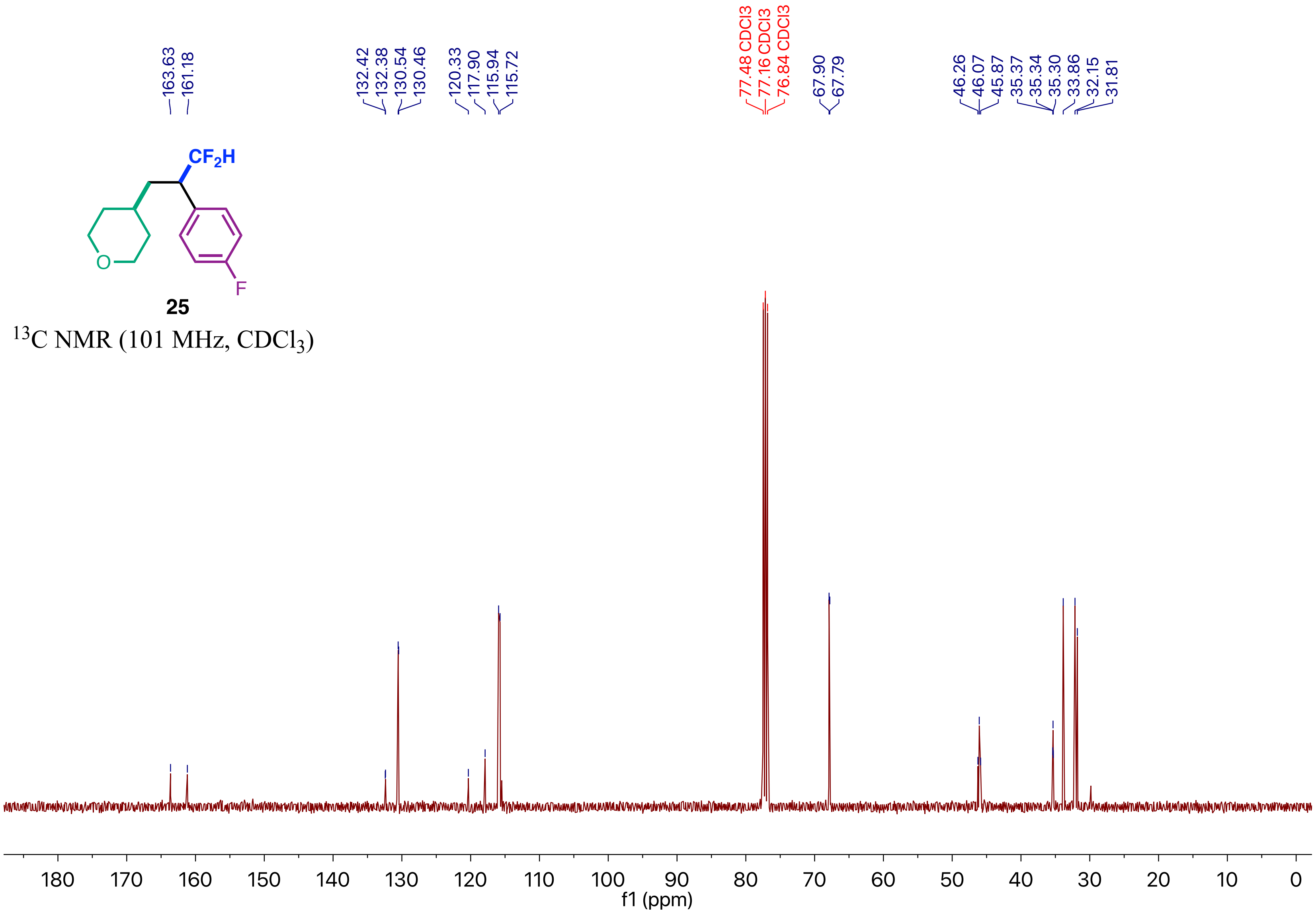

25

^{13}C NMR (101 MHz, $CDCl_3$)

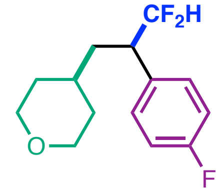

25

^{19}F NMR (376 MHz, $CDCl_3$)

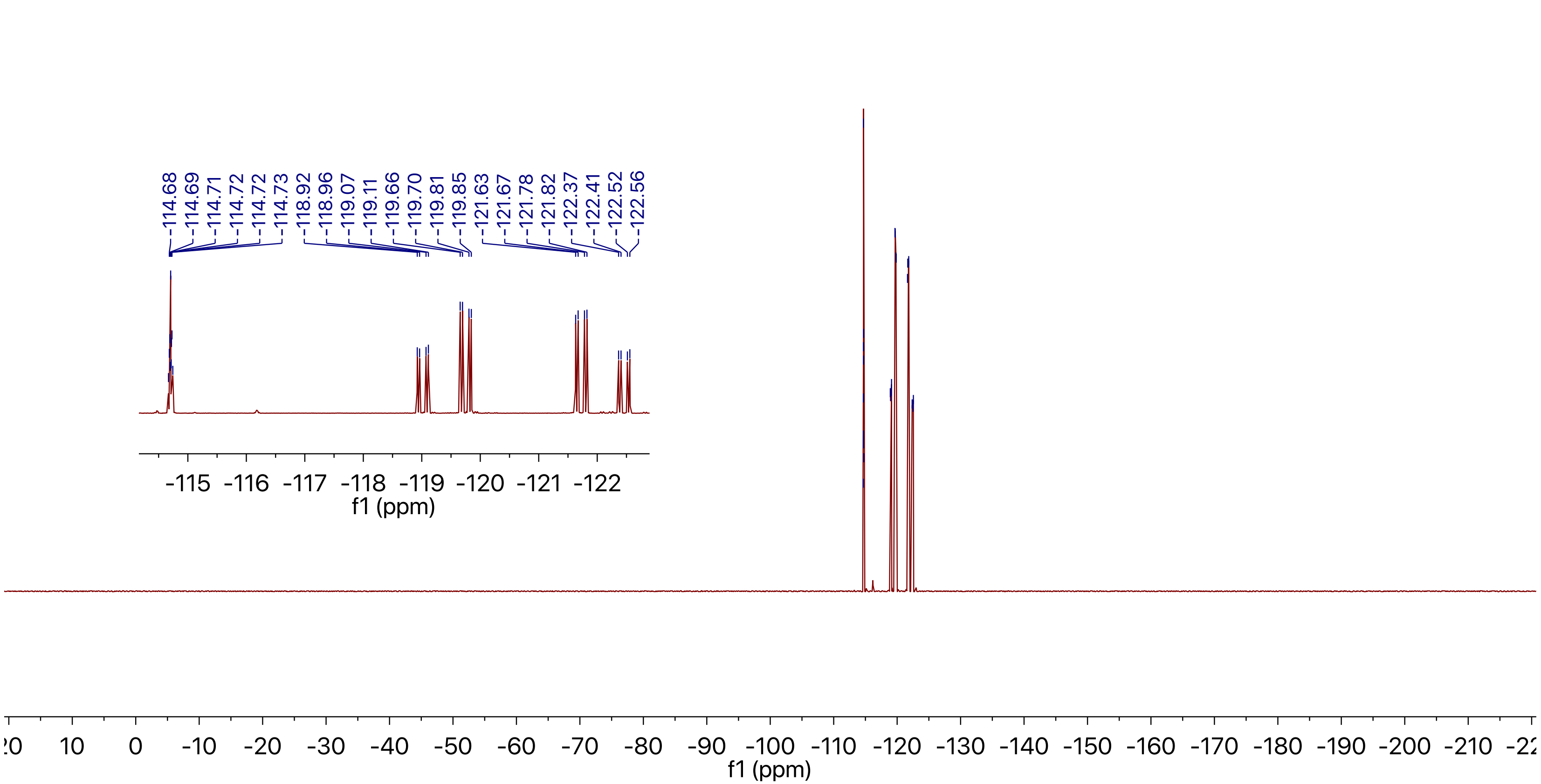

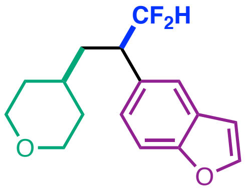

26

^{1}H NMR (400 MHz, $CDCl_3$)

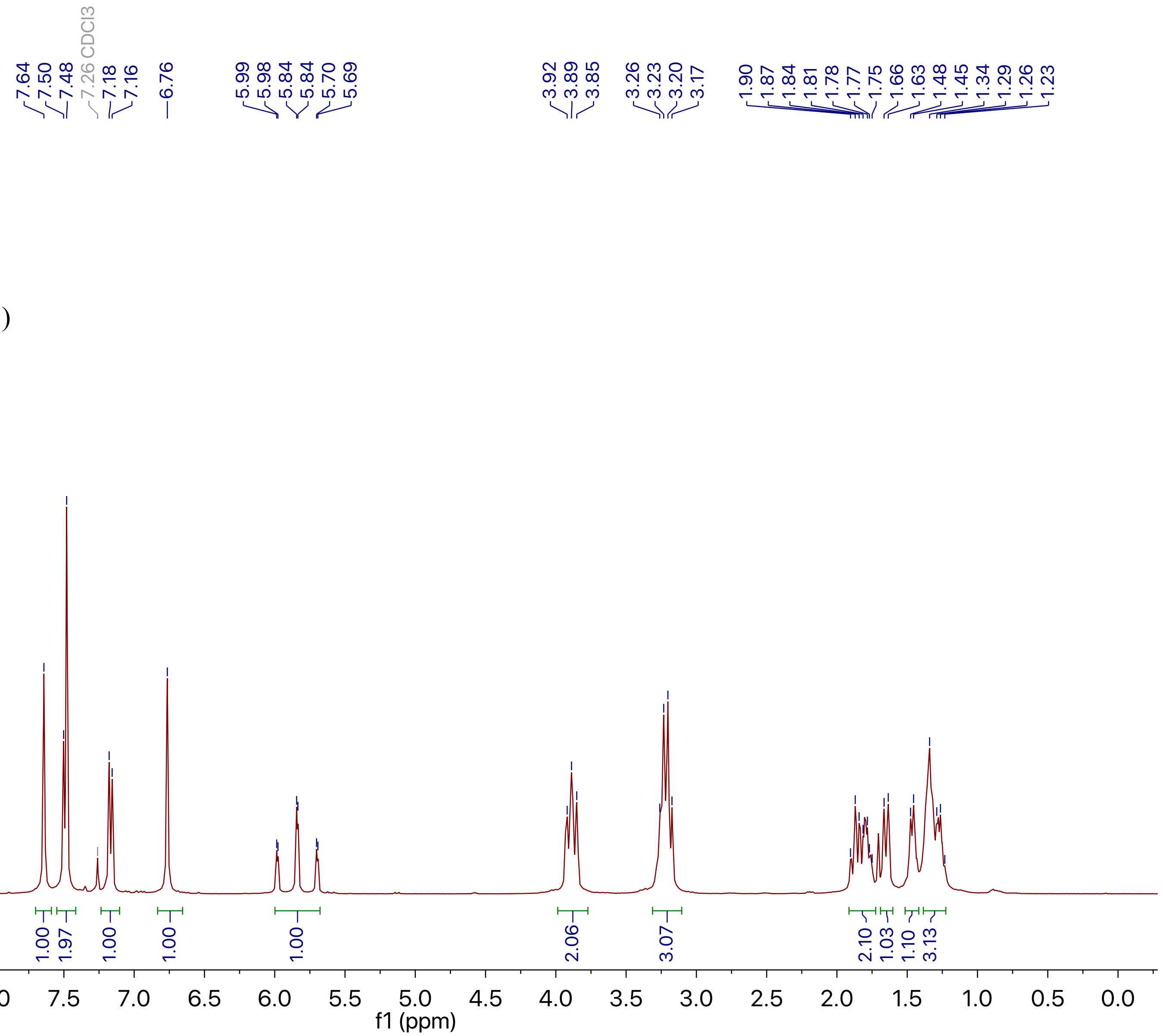

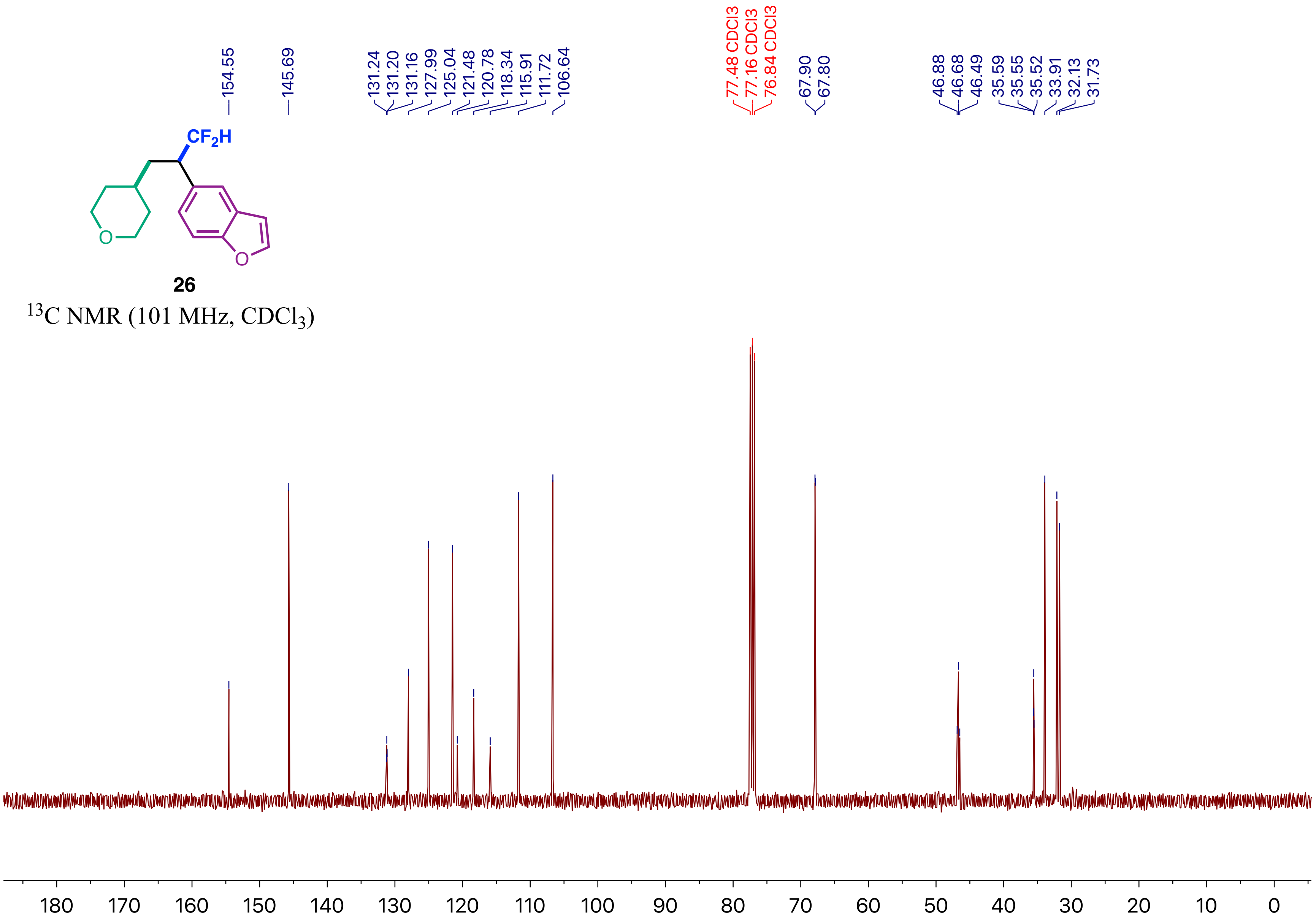

154.55
145.69
131.24
131.20
131.16
127.99
125.04
121.48
120.78
118.34
115.91
111.72
106.64
77.48 CDCl3
77.16 CDCl3
76.84 CDCl3
67.90
67.80
46.88
46.68
46.49
35.59
35.55
35.52
33.91
32.13
31.73
CF2H
O
O
26
13C NMR (101 MHz, CDCl3)
180 170 160 150 140 130 120 110 100 90 80 70 60 50 40 30 20 10 0

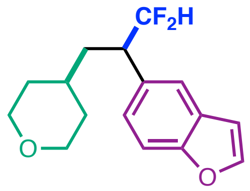

26

^{19}F NMR (376 MHz, $CDCl_3$)

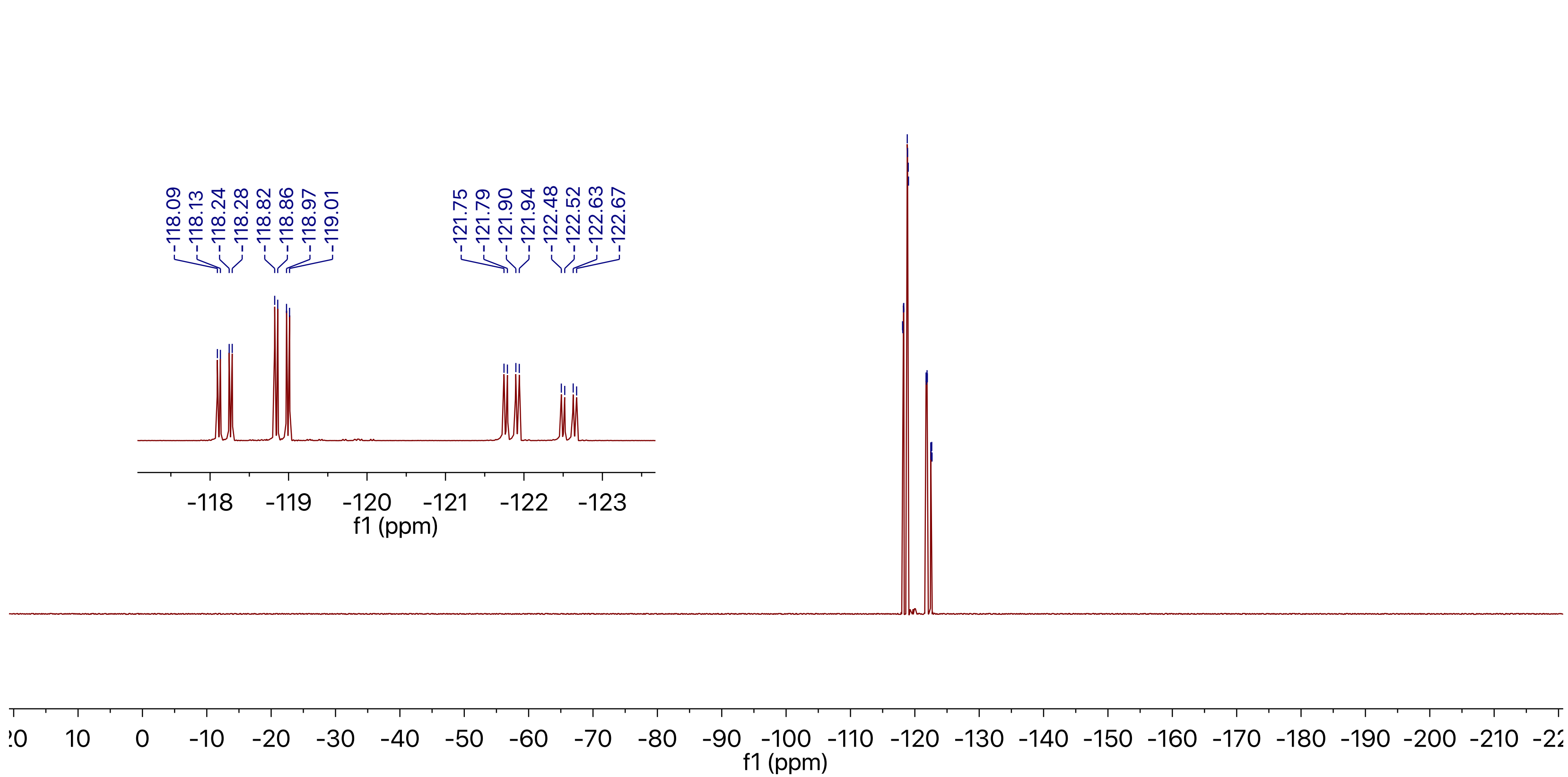

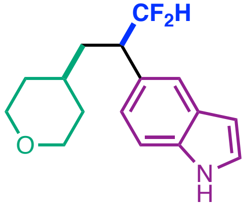

27

^{1}H NMR (400 MHz, $CDCl_3$)

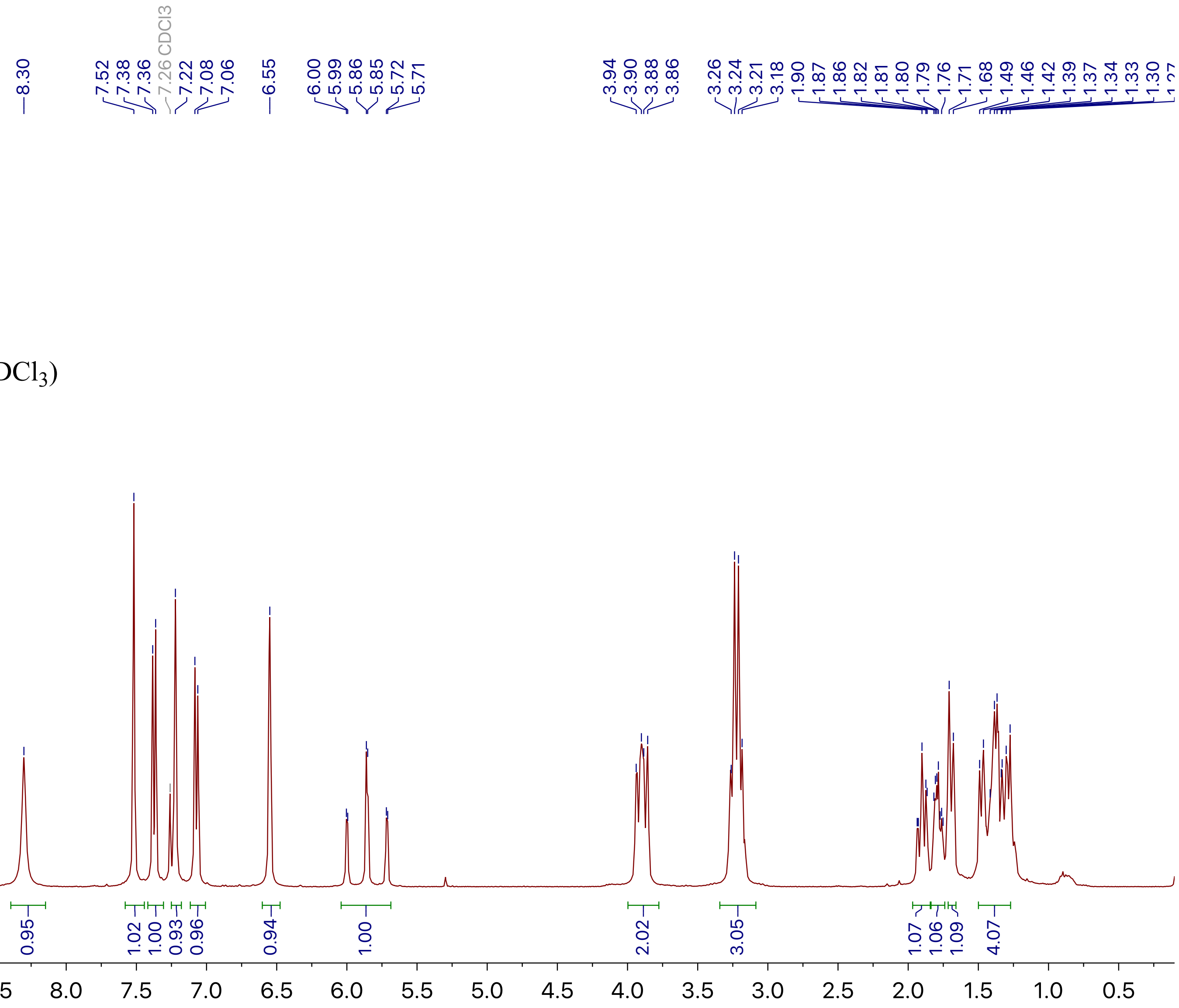

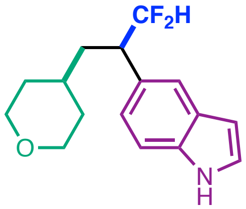

27

^{13}C NMR (101 MHz, $CDCl_3$)

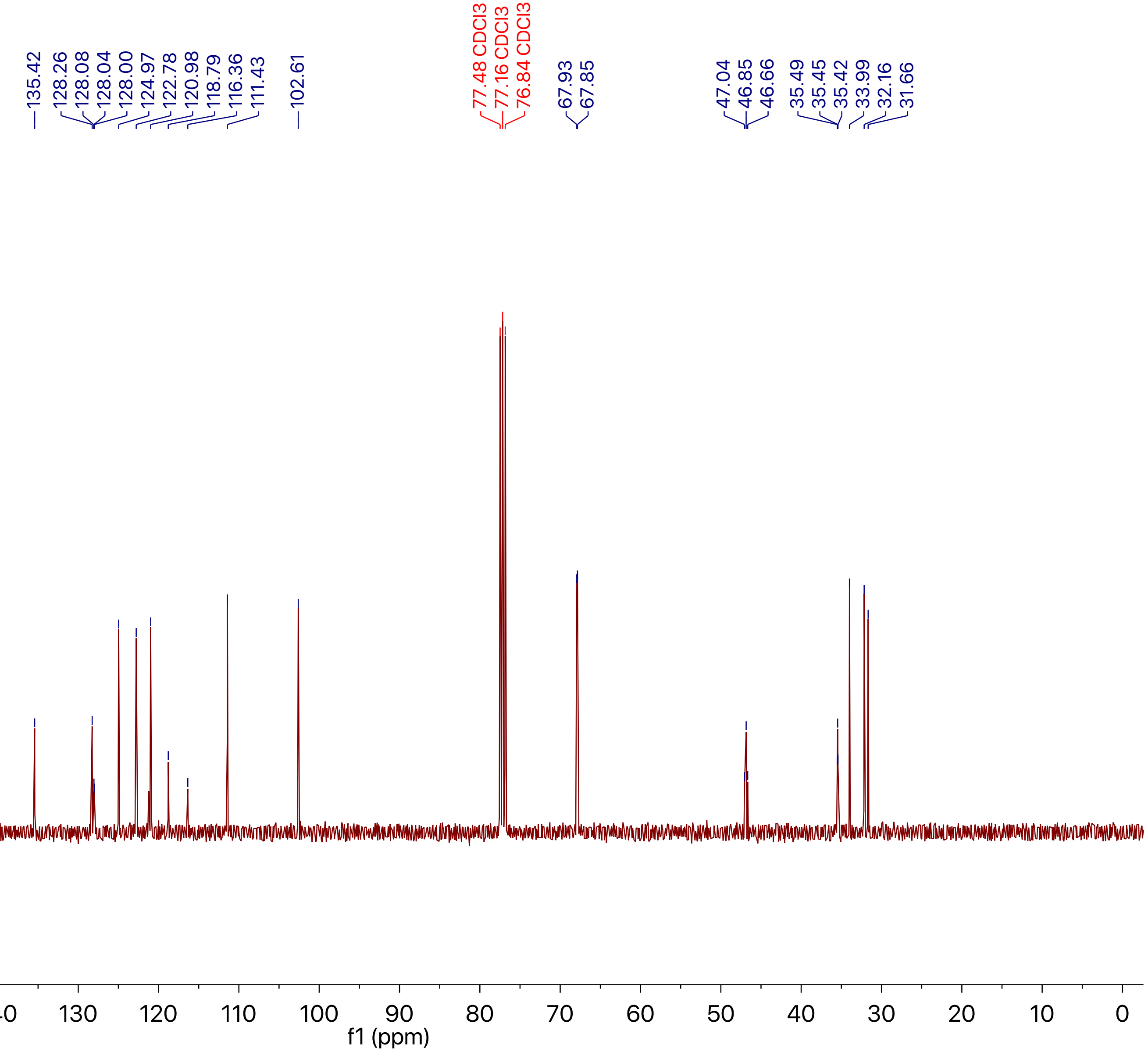

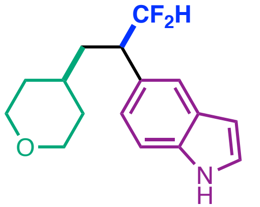

27

^{19}F NMR (376 MHz, $CDCl_3$)

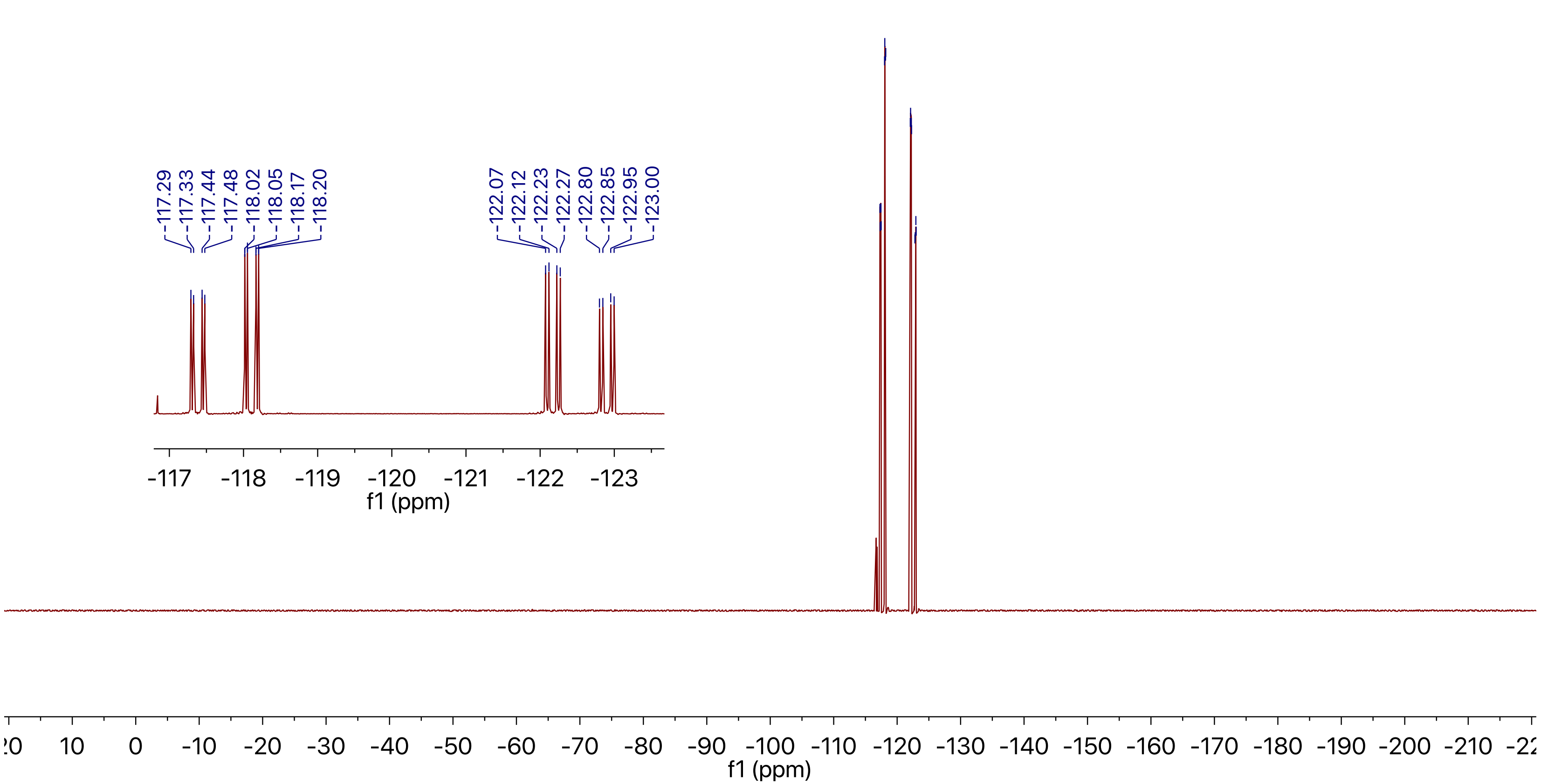

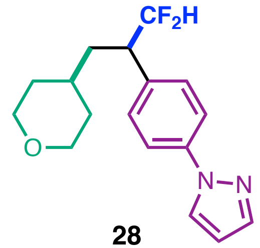

28

^{1}H NMR (400 MHz, $CDCl_3$)

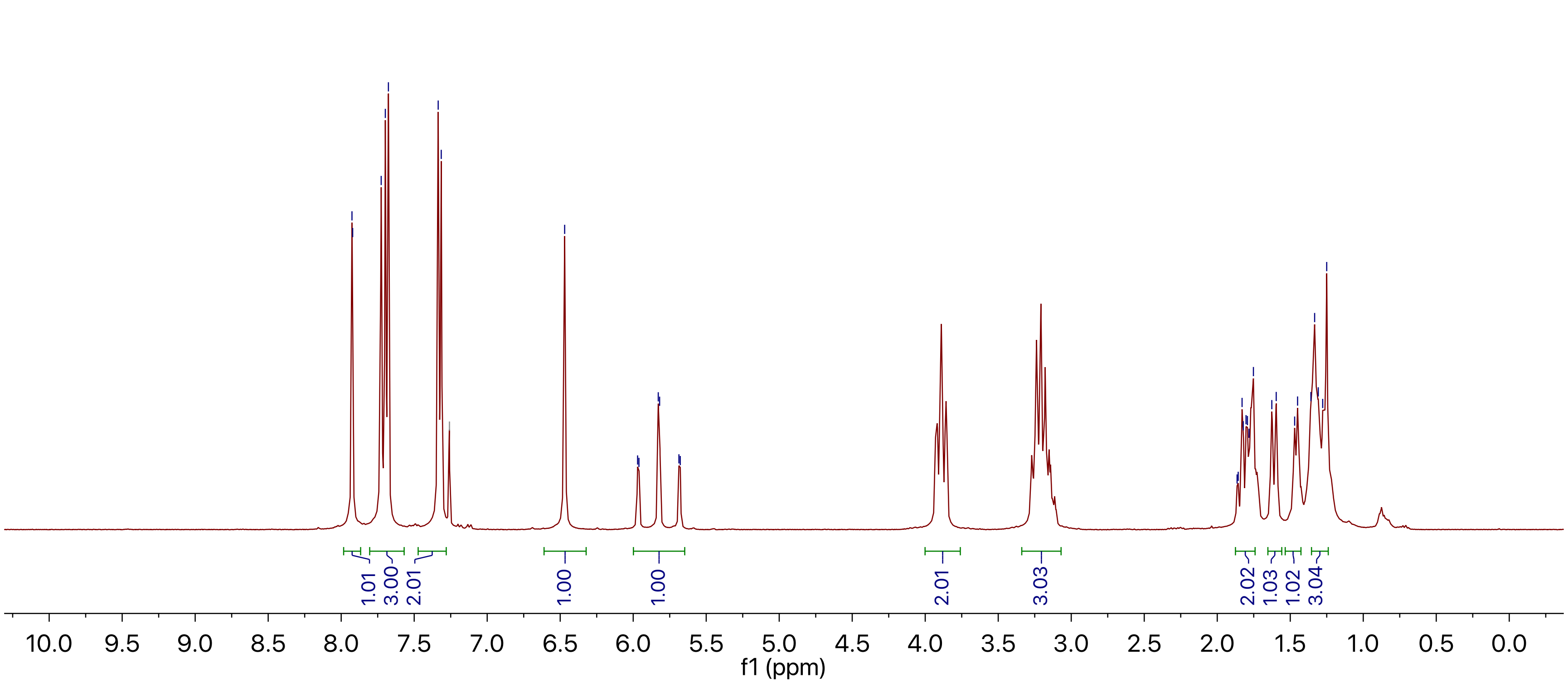

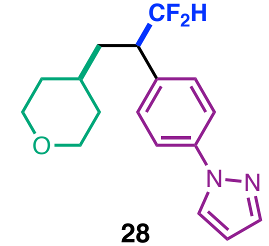

28

^{13}C NMR (101 MHz, $CDCl_3$)

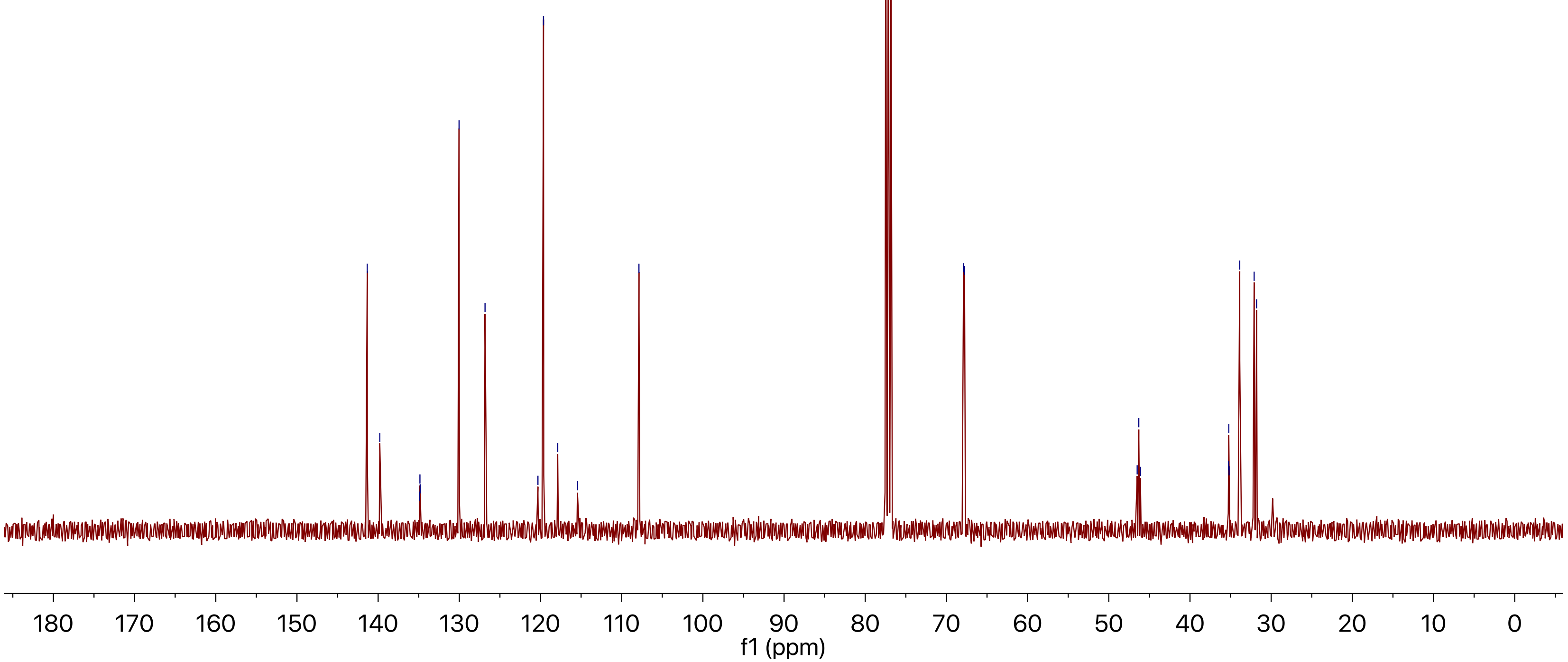

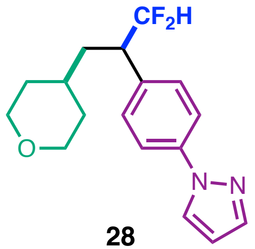

28

^{19}F NMR (376 MHz, $CDCl_3$)

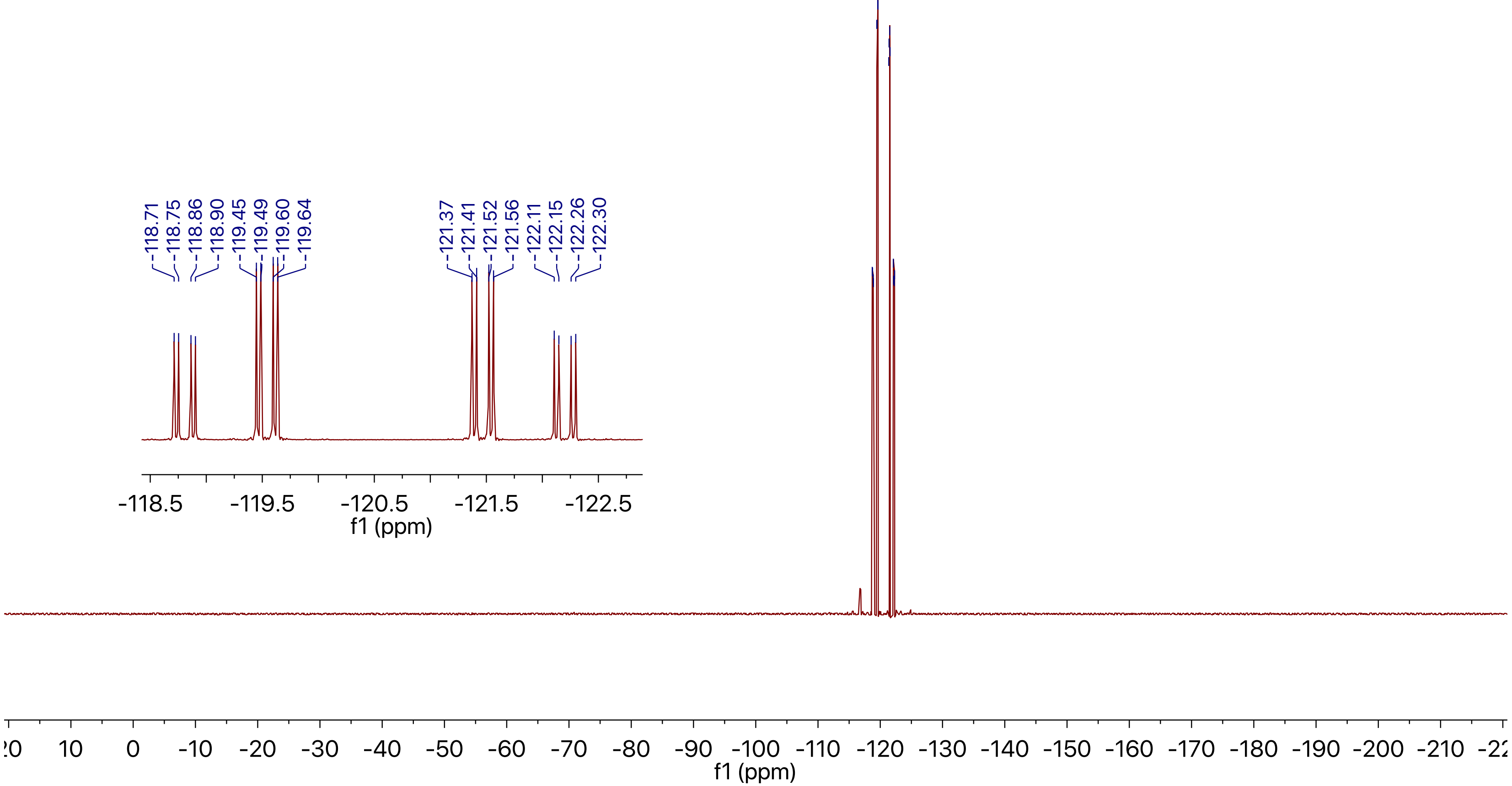

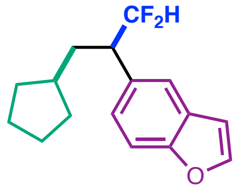

29

^{1}H NMR (400 MHz, $CDCl_3$)

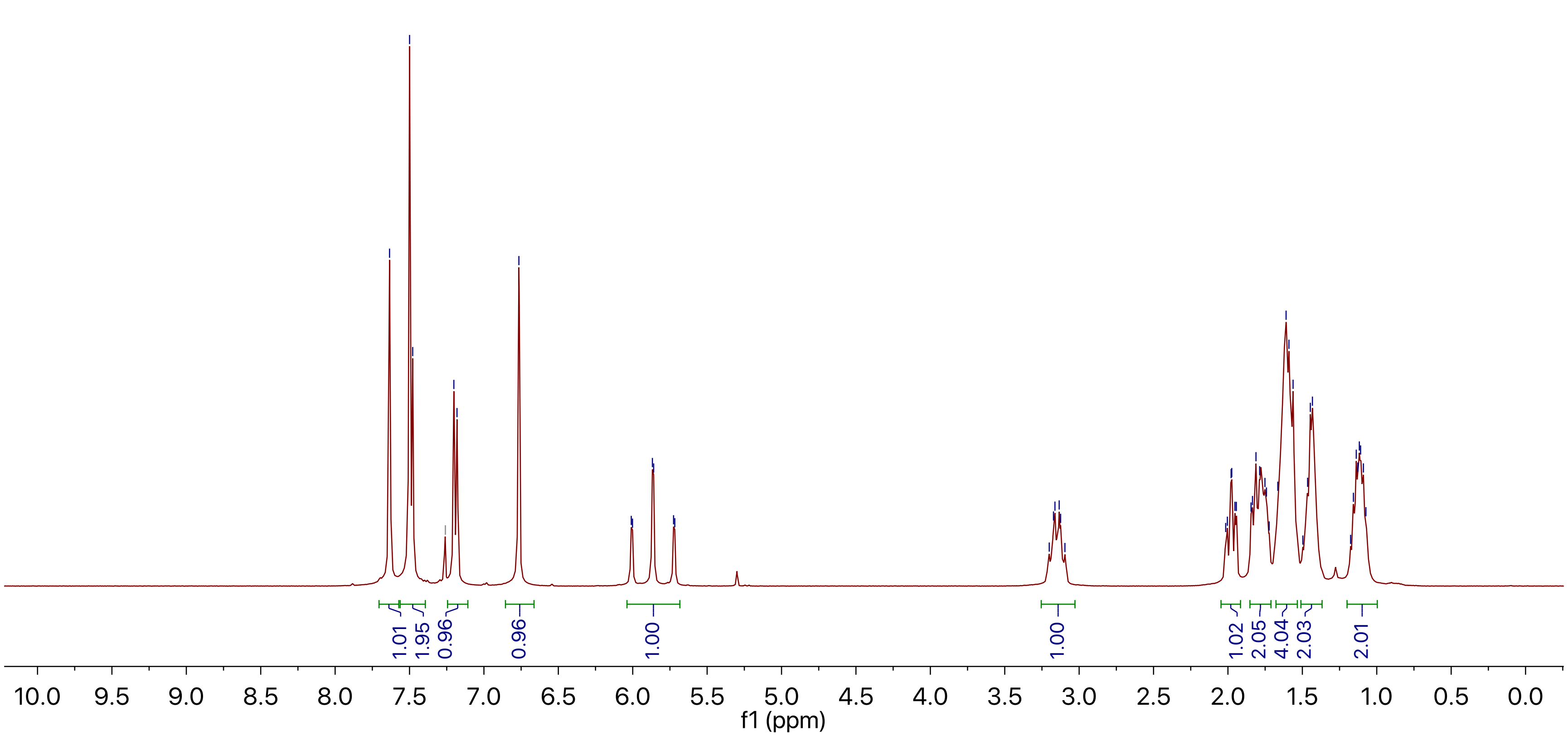

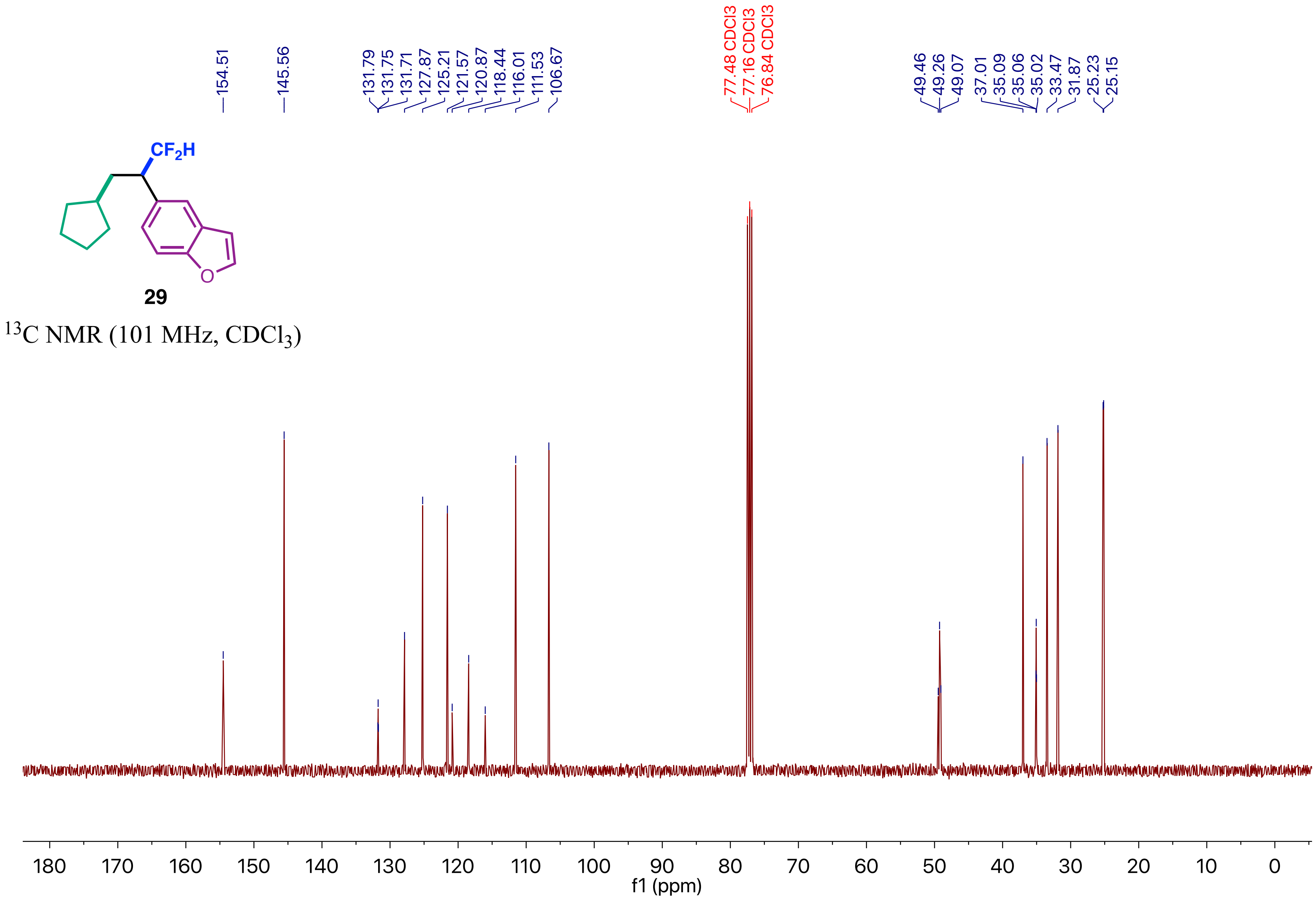
154.51
145.56
131.79
131.75
131.71
127.87
125.21
121.57
120.87
118.44
116.01
111.53
106.67
77.48 CDCl3
77.16 CDCl3
76.84 CDCl3
49.46
49.26
49.07
37.01
35.09
35.06
35.02
33.47
31.87
25.23
25.15
CF_2H
O
29
^{13}C NMR (101 MHz, $CDCl_3$)
180 170 160 150 140 130 120 110 100 90 80 70 60 50 40 30 20 10 0
f1 (ppm)

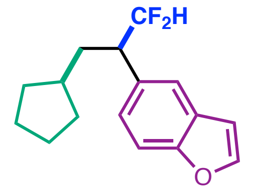

29

^{19}F NMR (376 MHz, $CDCl_3$)

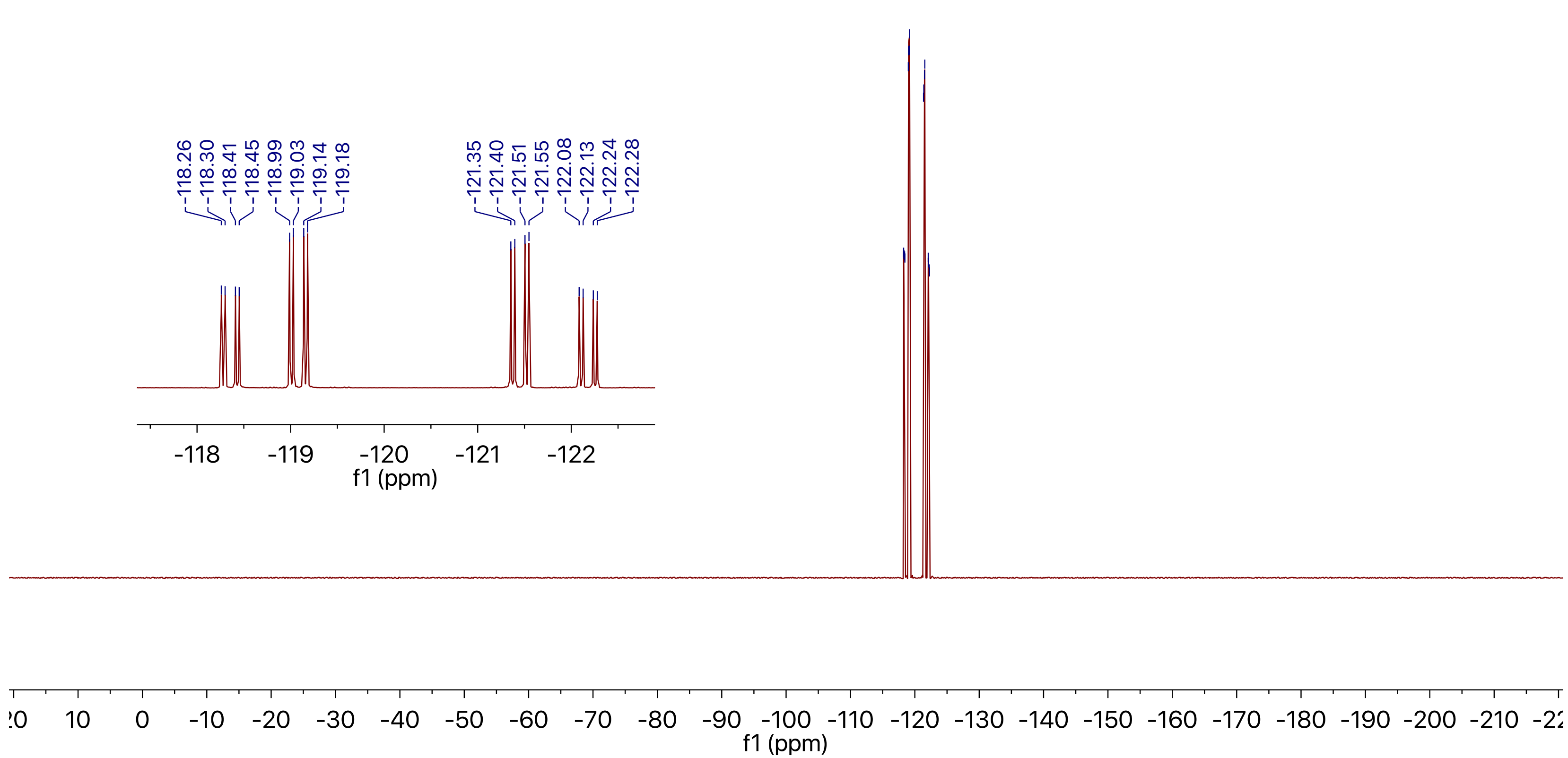

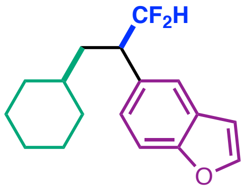

30

^{1}H NMR (400 MHz, $CDCl_3$)

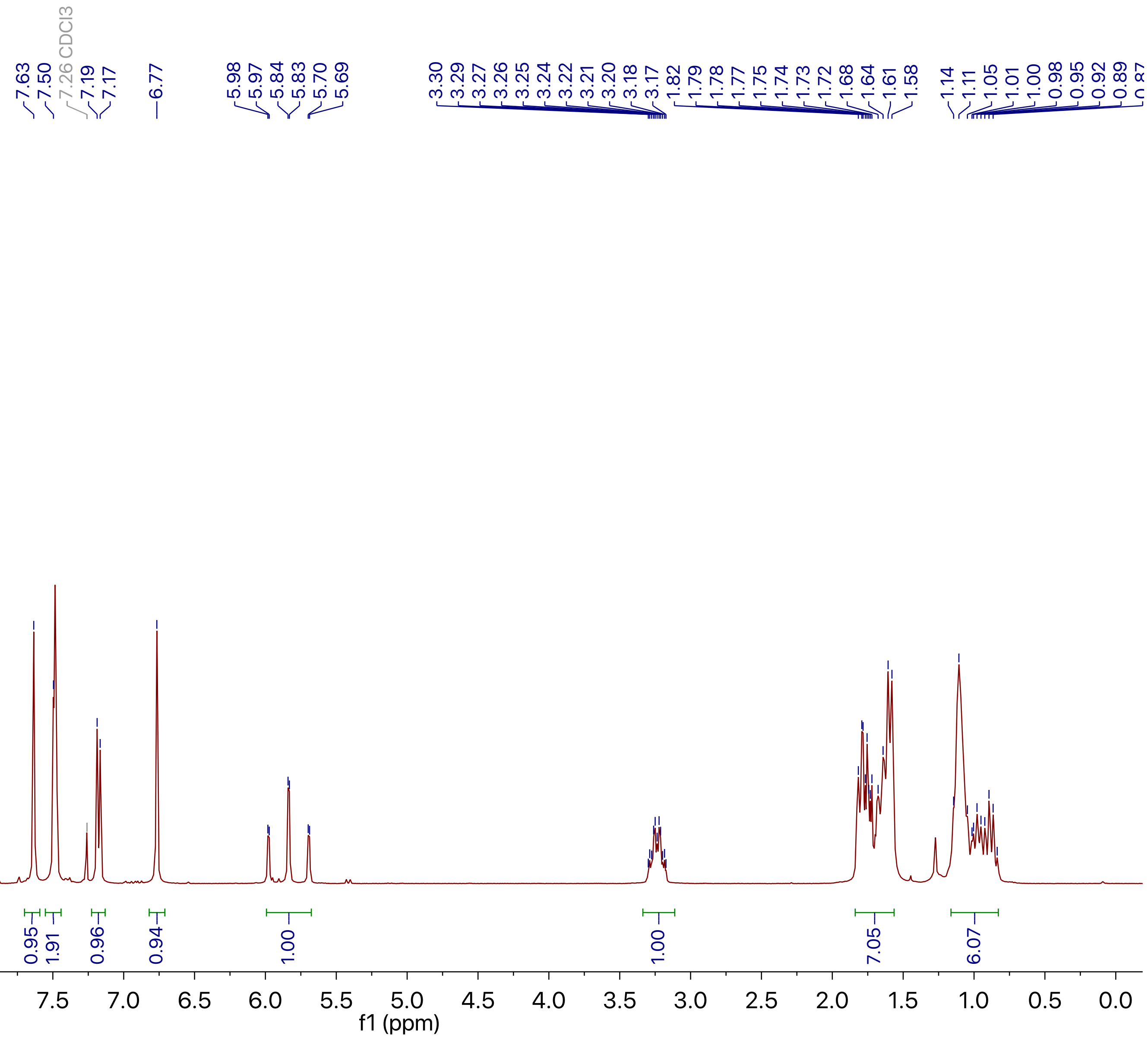

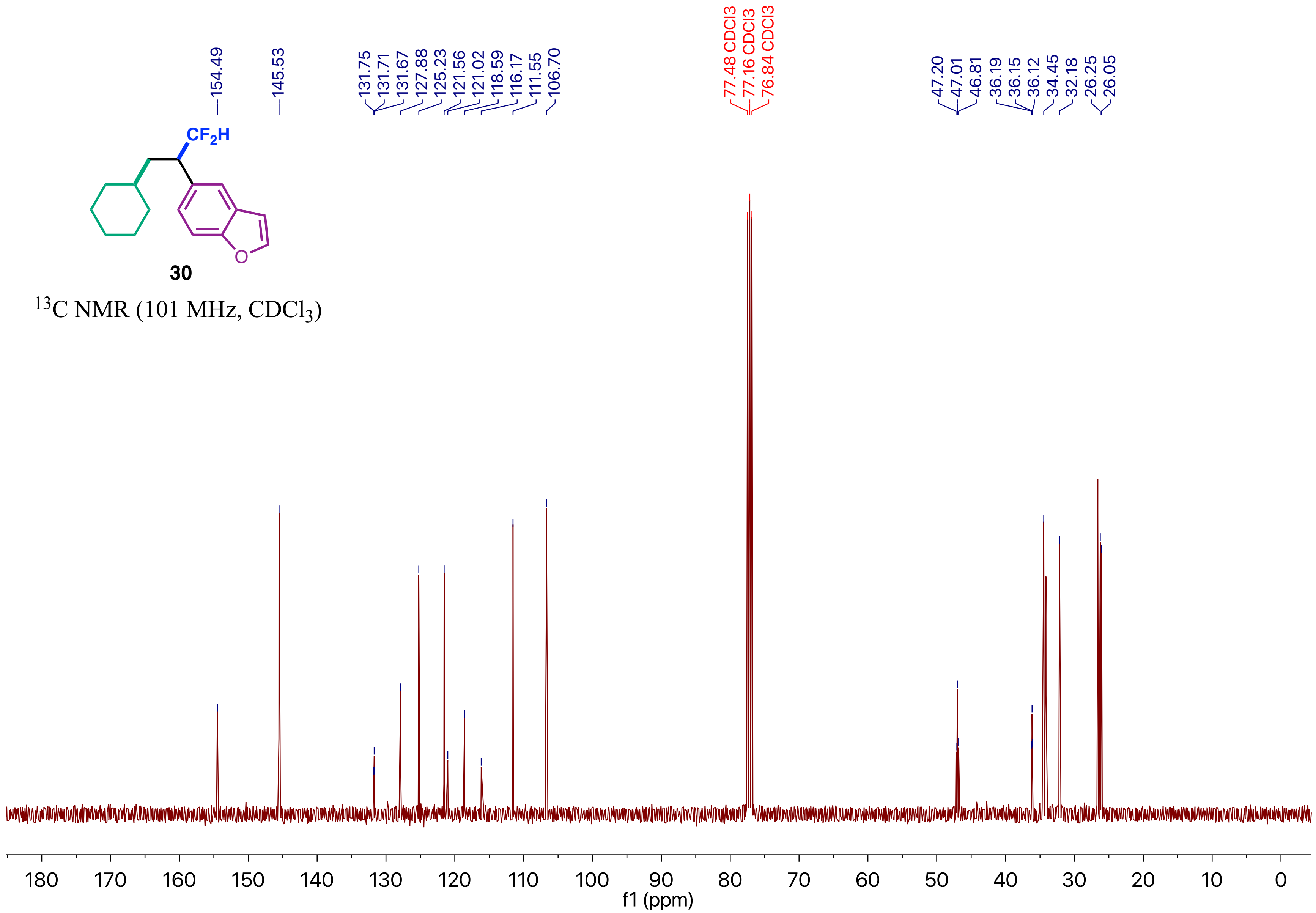

CF2H
30
13C NMR (101 MHz, CDCl3)
154.49
145.53
131.75
131.71
131.67
127.88
125.23
121.56
121.02
118.59
116.17
111.55
106.70
77.48 CDCl3
77.16 CDCl3
76.84 CDCl3
47.20
47.01
46.81
36.19
36.15
36.12
34.45
32.18
26.25
26.05
180 170 160 150 140 130 120 110 100 90 80 70 60 50 40 30 20 10 0
f1 (ppm)

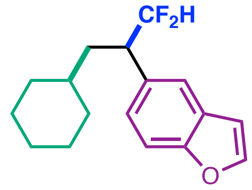

30

^{19}F NMR (376 MHz, $CDCl_3$)

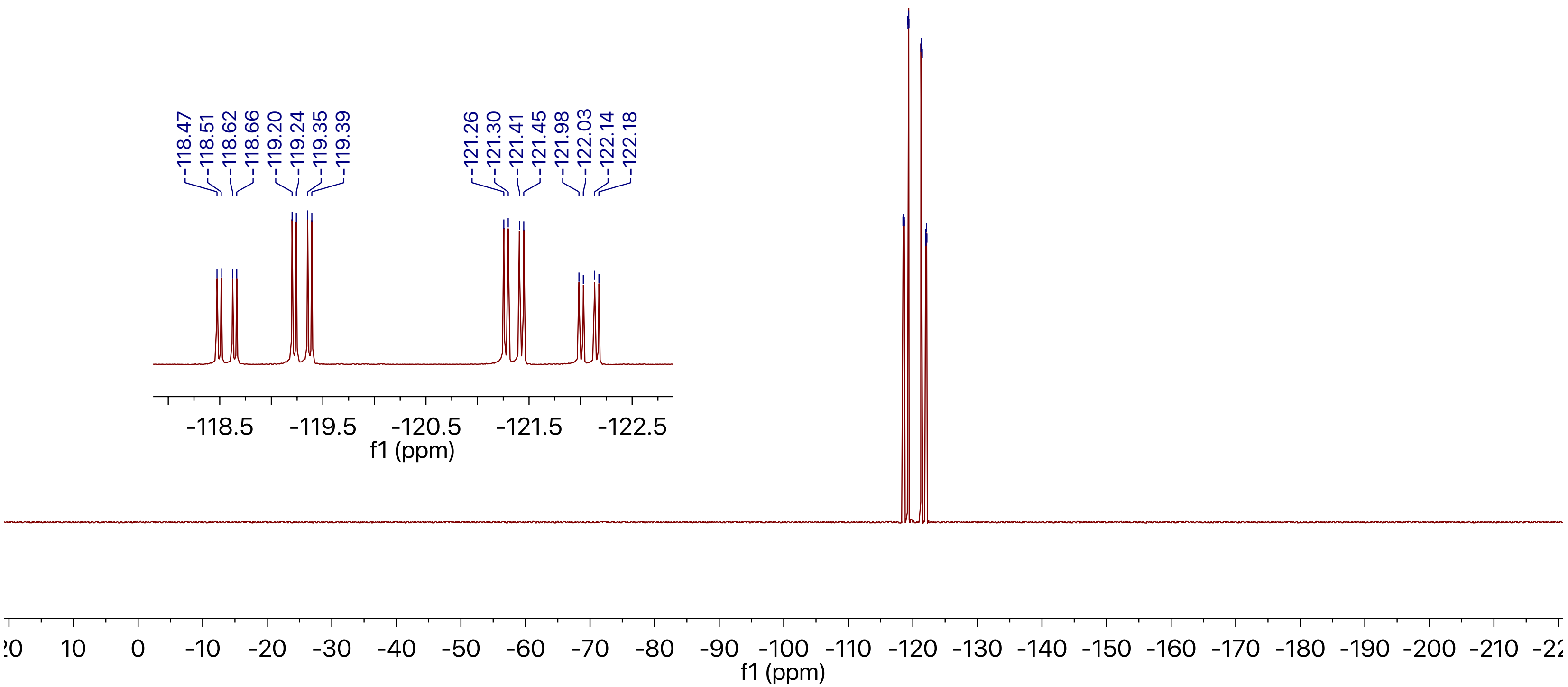

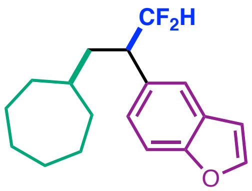

31

^{1}H NMR (400 MHz, $CDCl_3$)

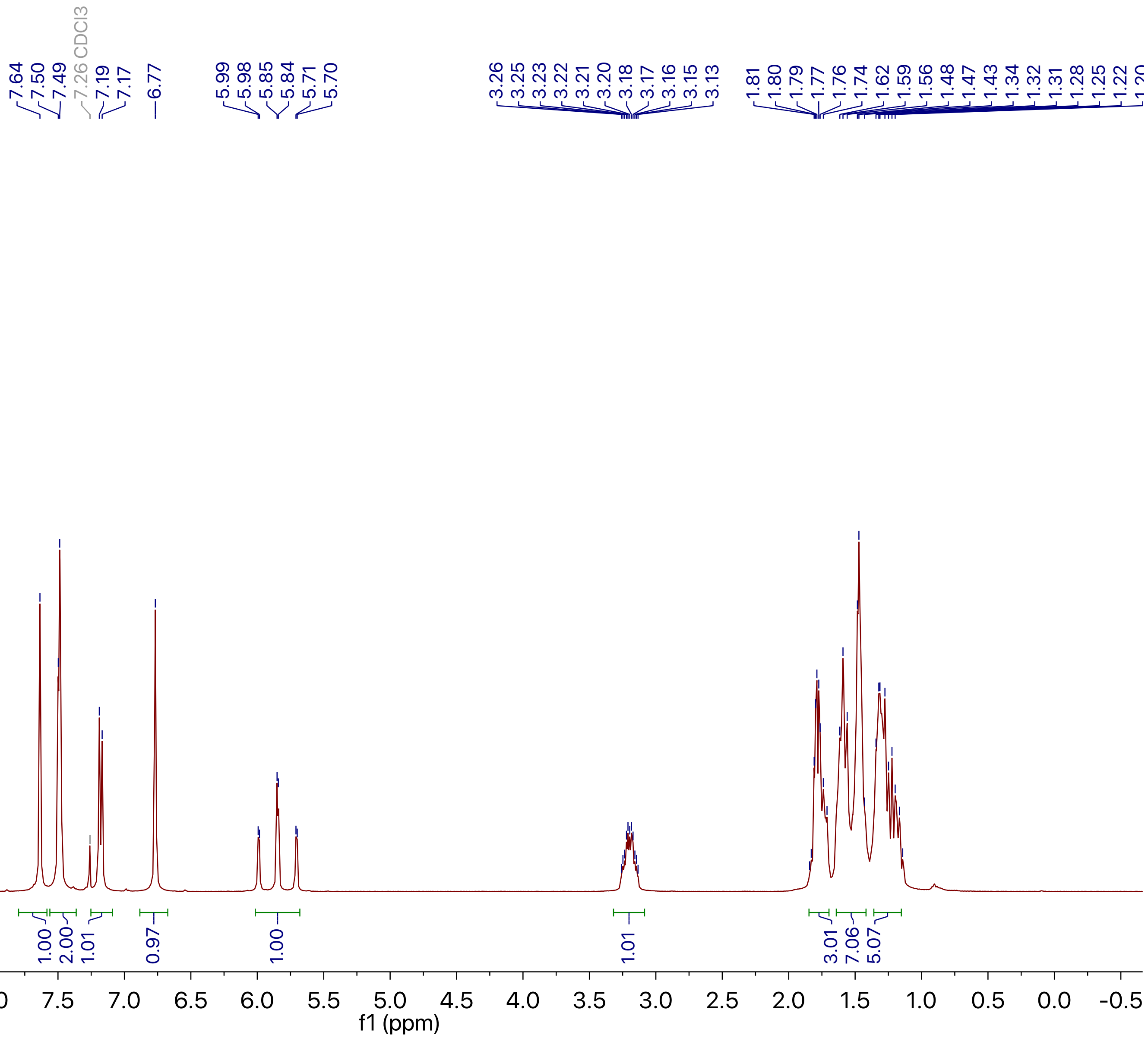

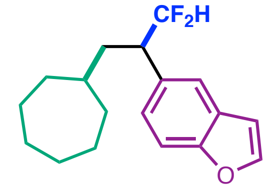

31

^{13}C NMR (101 MHz, $CDCl_3$)

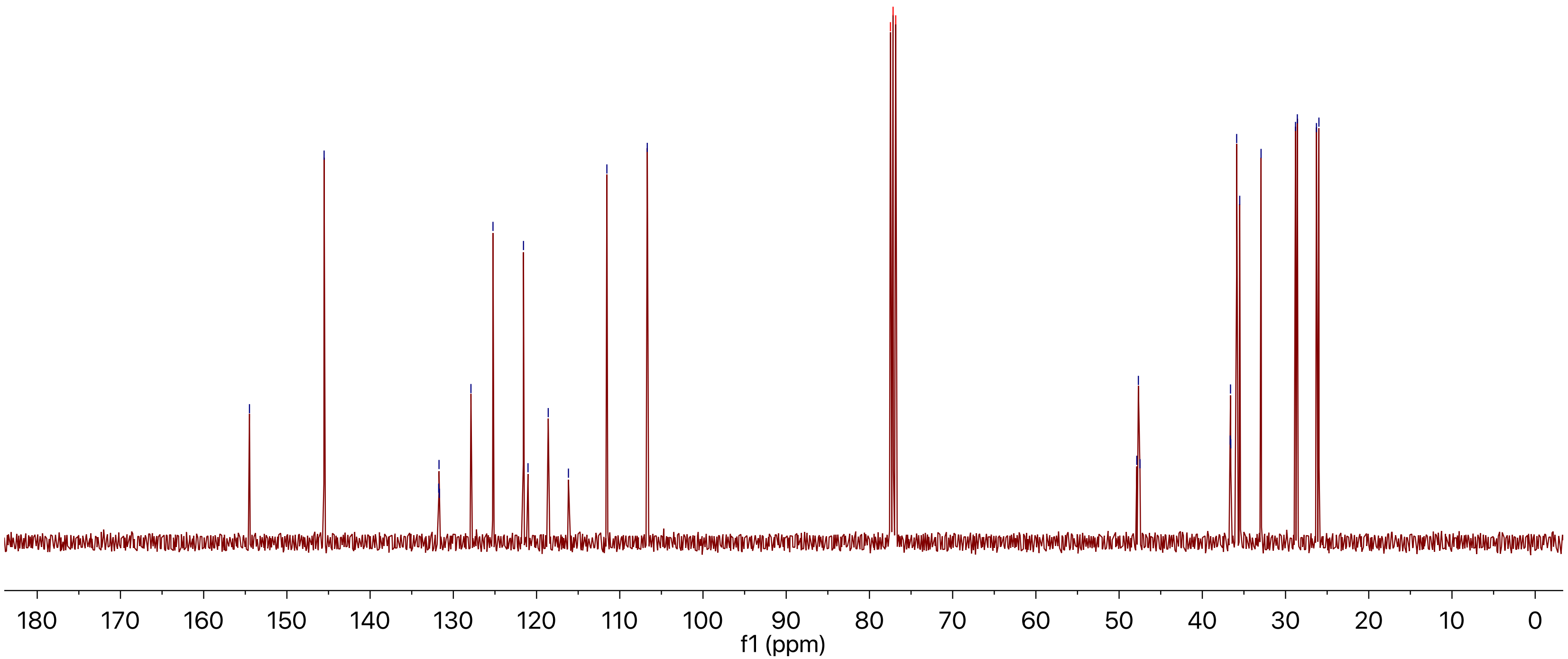

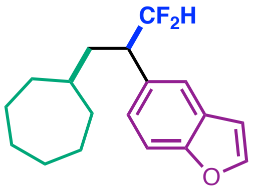

31

^{19}F NMR (376 MHz, $CDCl_3$)

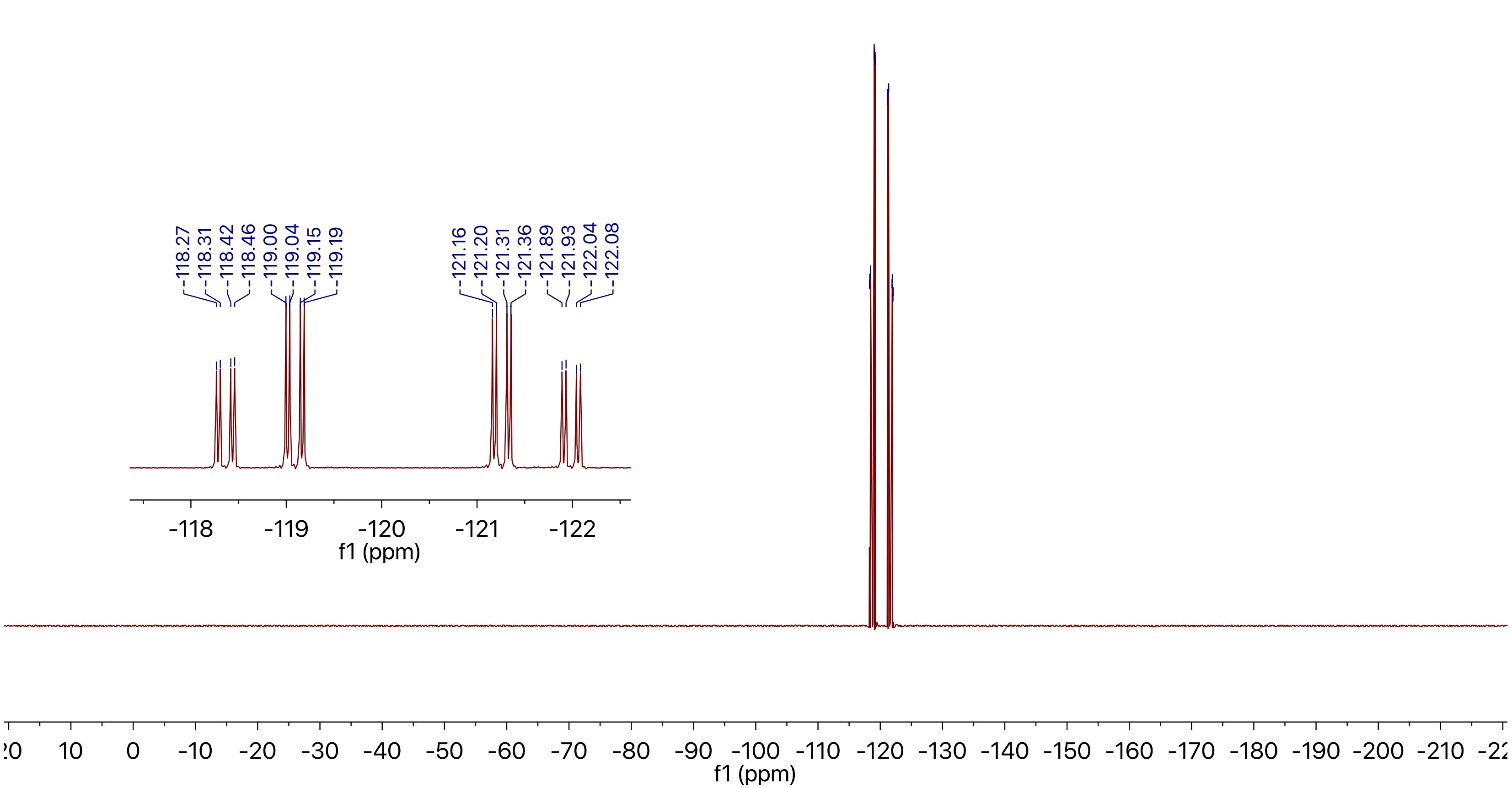

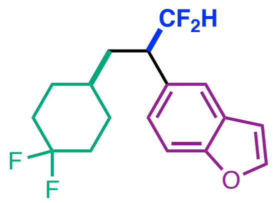

32

^{1}H NMR (400 MHz, $CDCl_3$)

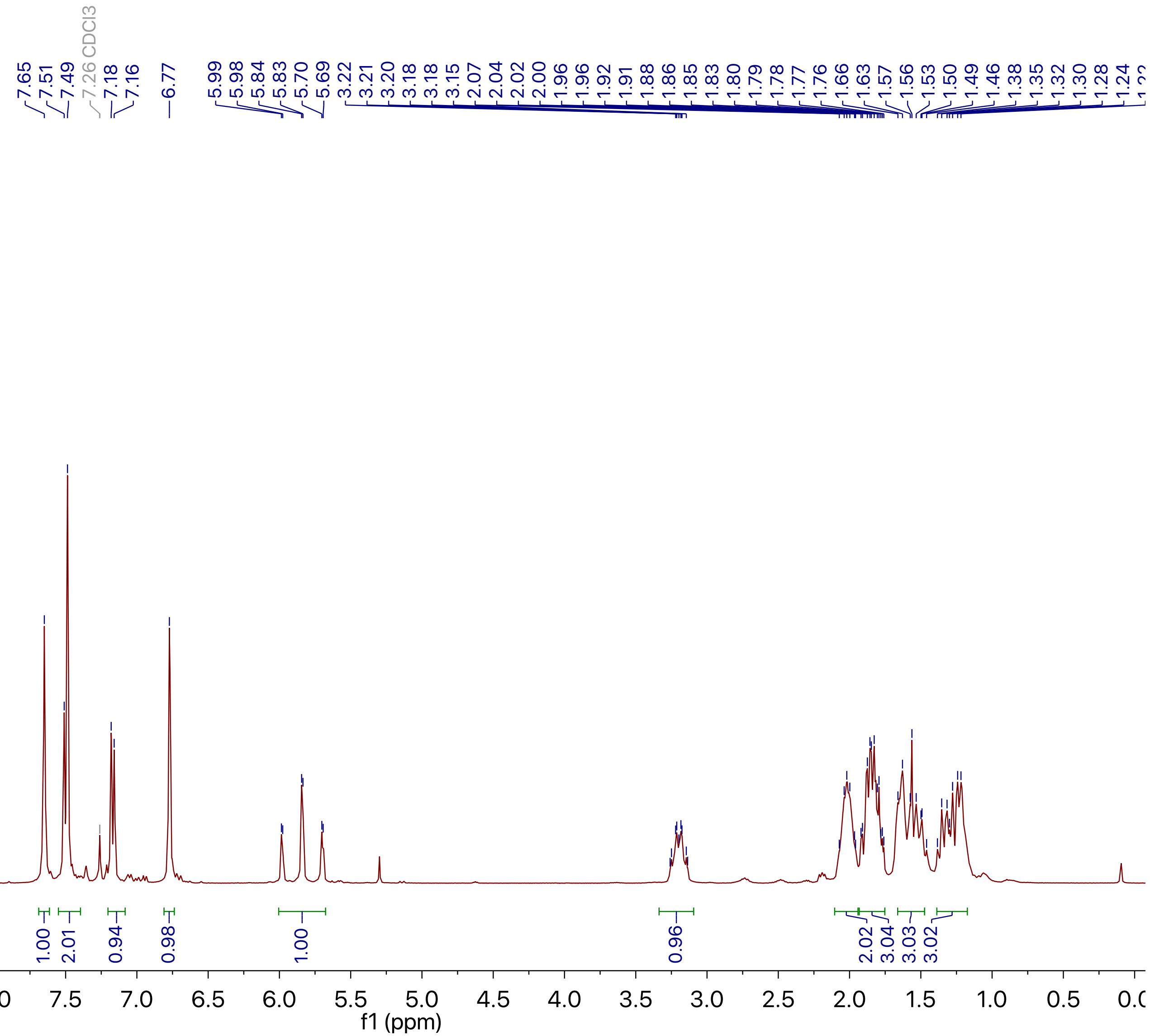

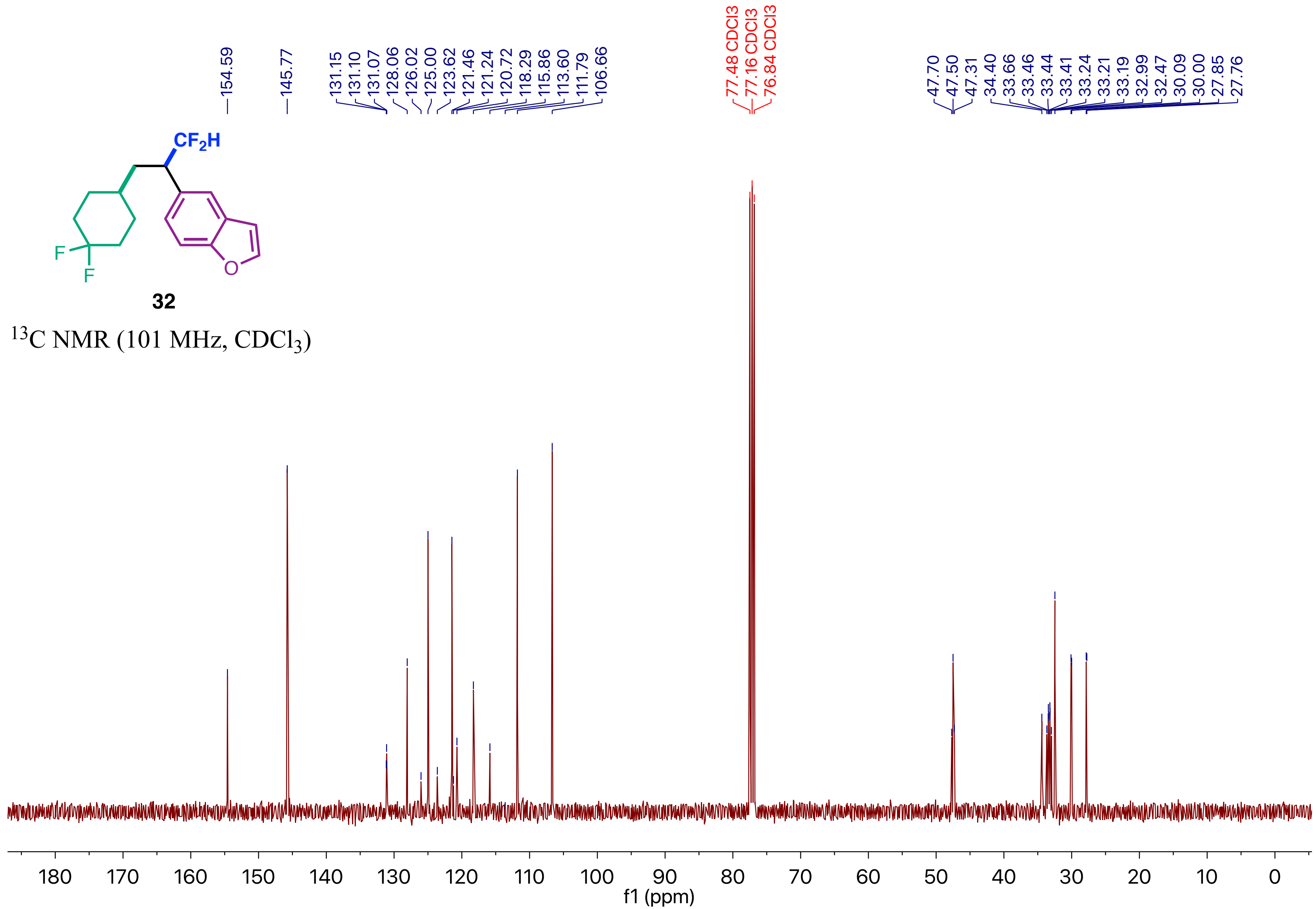
154.59
145.77
131.15
131.10
131.07
128.06
126.02
125.00
123.62
121.46
121.24
120.72
118.29
115.86
113.60
111.79
106.66
77.48 CDCl3
77.16 CDCl3
76.84 CDCl3
47.70
47.50
47.31
34.40
33.66
33.46
33.44
33.41
33.24
33.21
33.19
32.99
32.47
30.09
30.00
27.85
27.76
CF2H
F
F
O
32
13C NMR (101 MHz, CDCl3)
180
170
160
150
140
130
120
110
100
90
80
70
60
50
40
30
20
10
0
f1 (ppm)

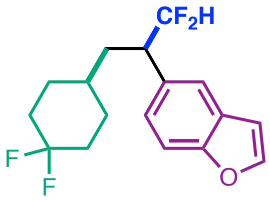

32

^{19}F NMR (376 MHz, $CDCl_3$)

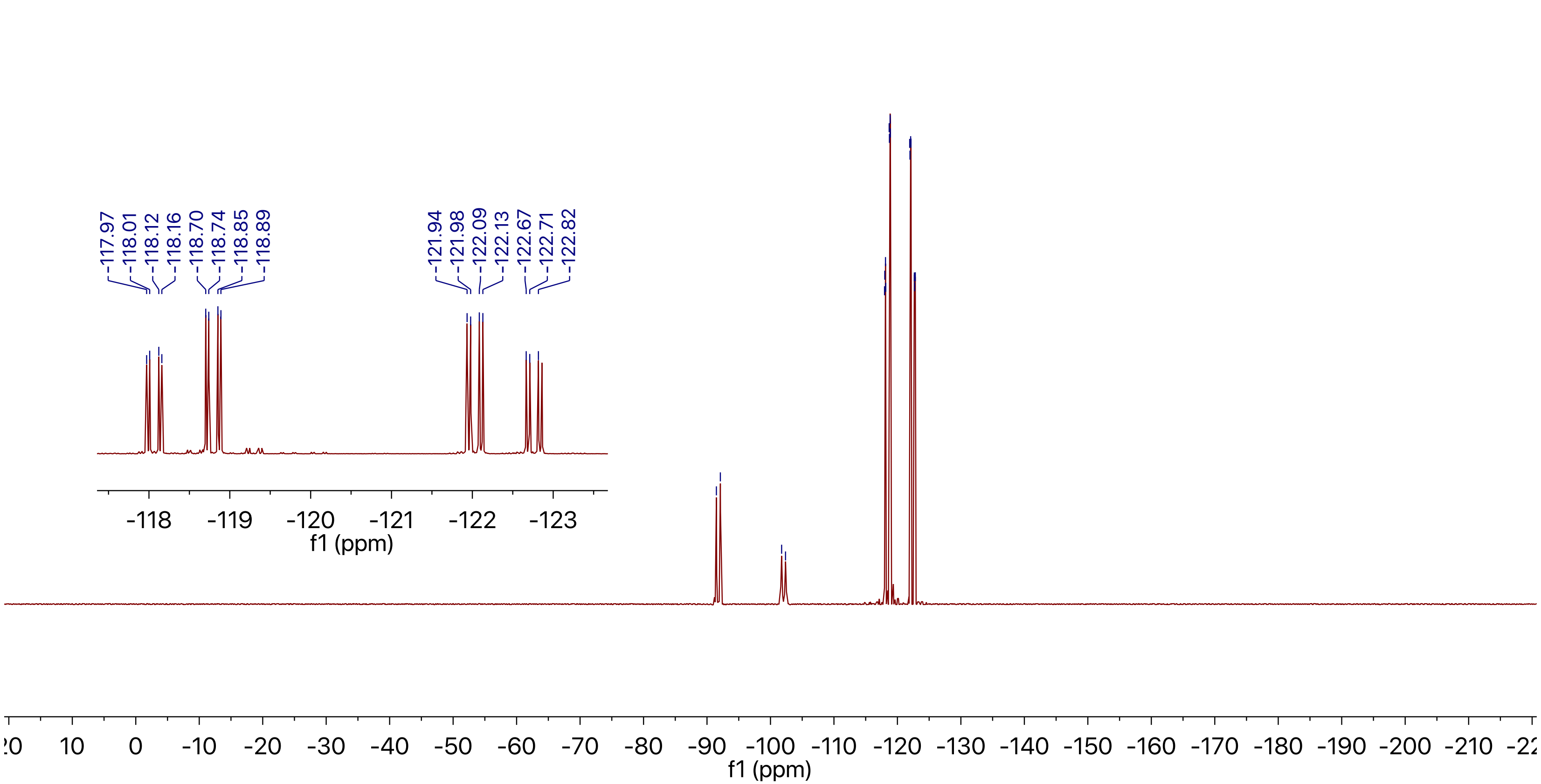

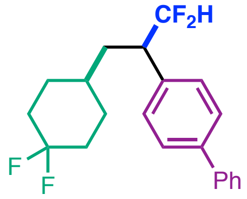

33

^{1}H NMR (400 MHz, $CDCl_3$)

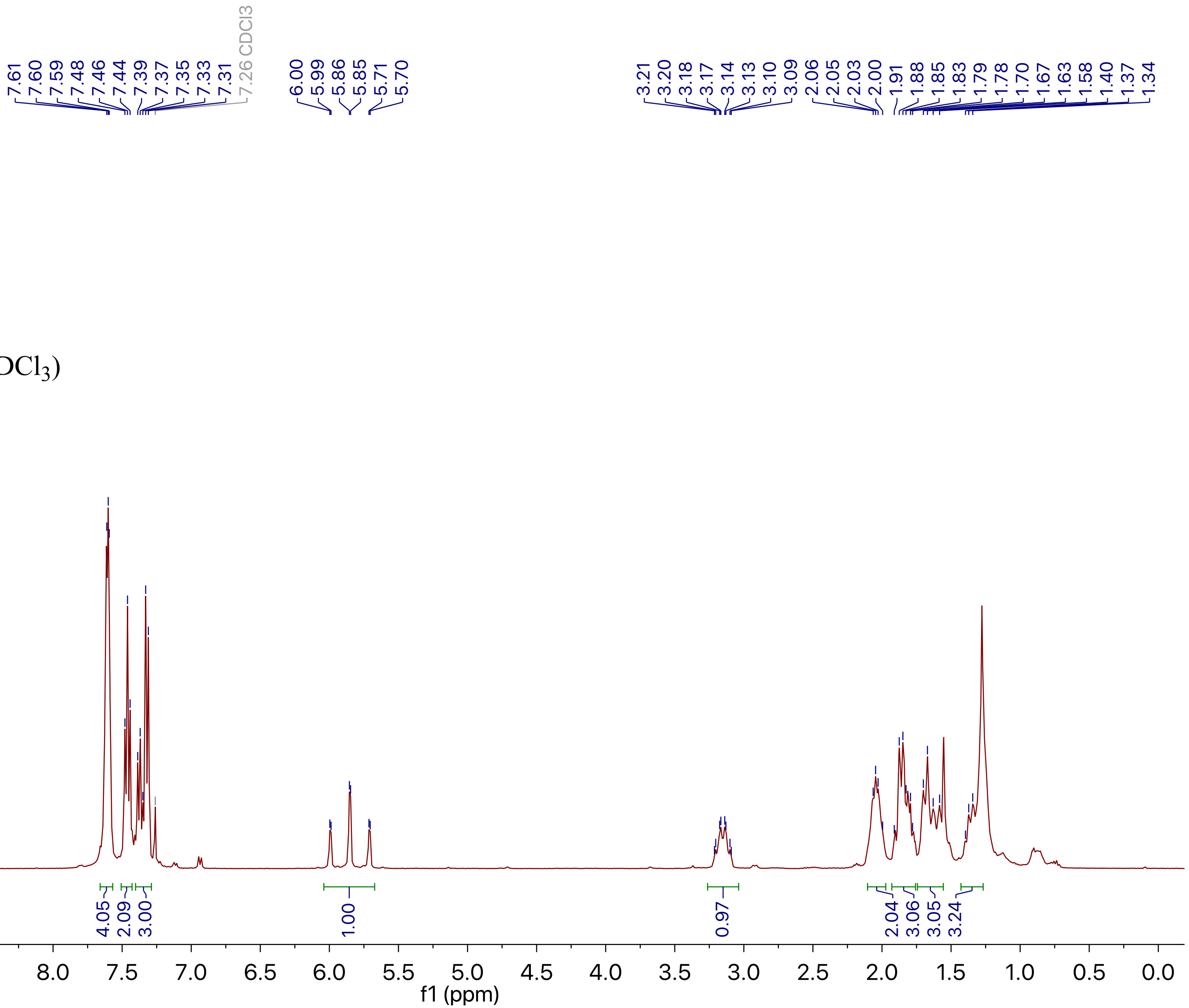

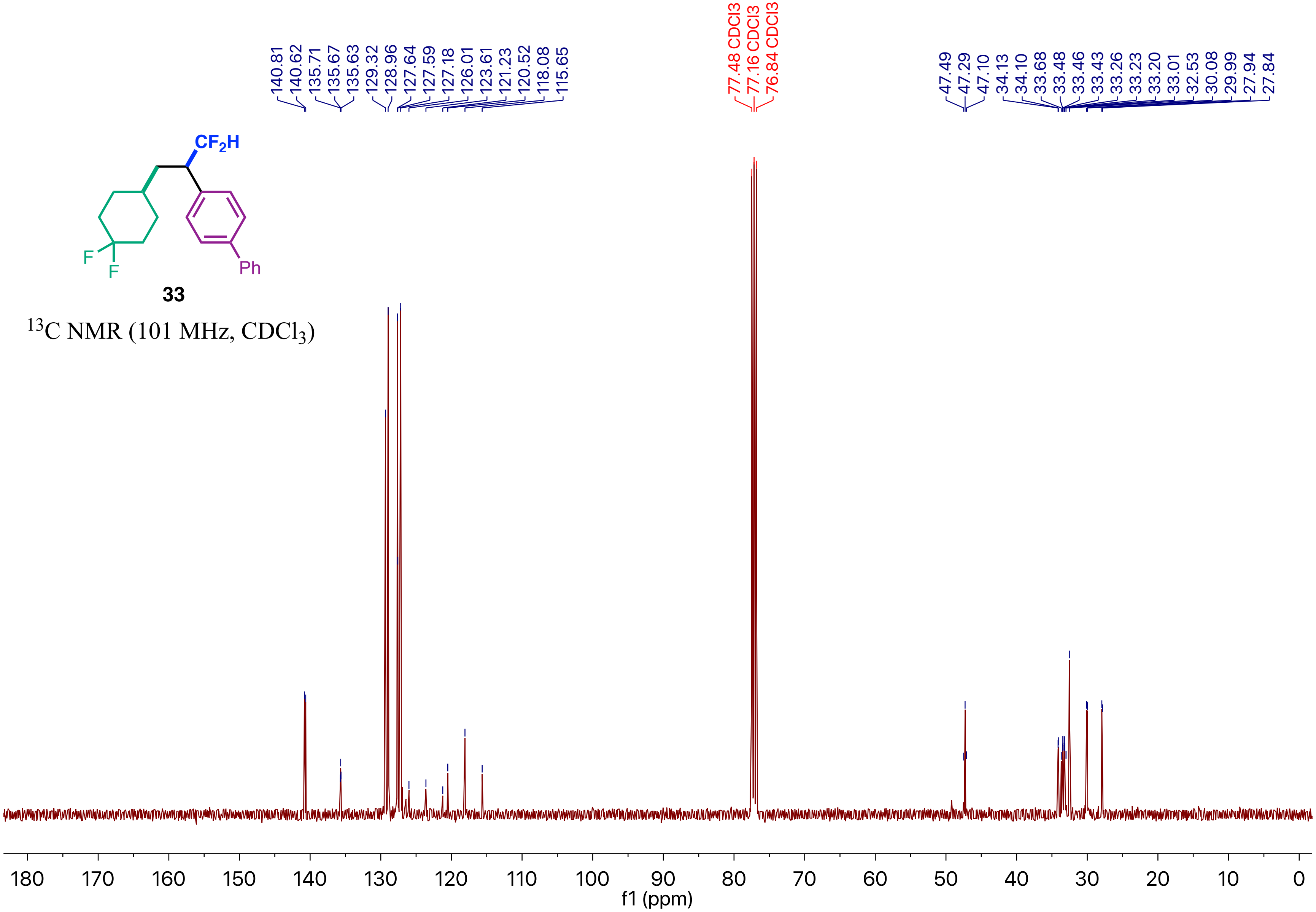

^{13}C NMR (101 MHz, $CDCl_3$)

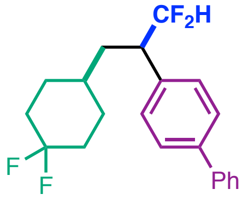

33

^{19}F NMR (376 MHz, CDCl$_3$)

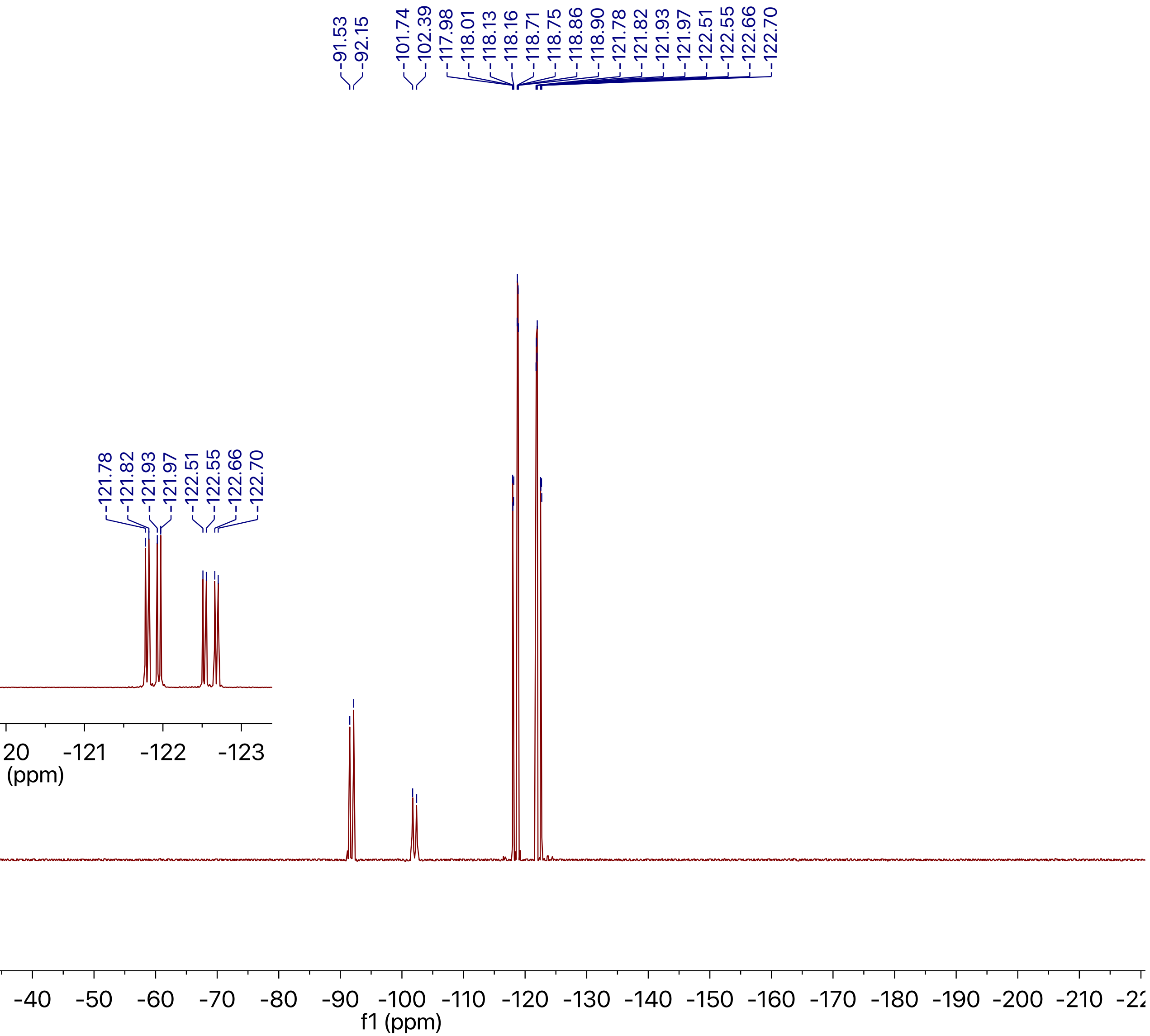

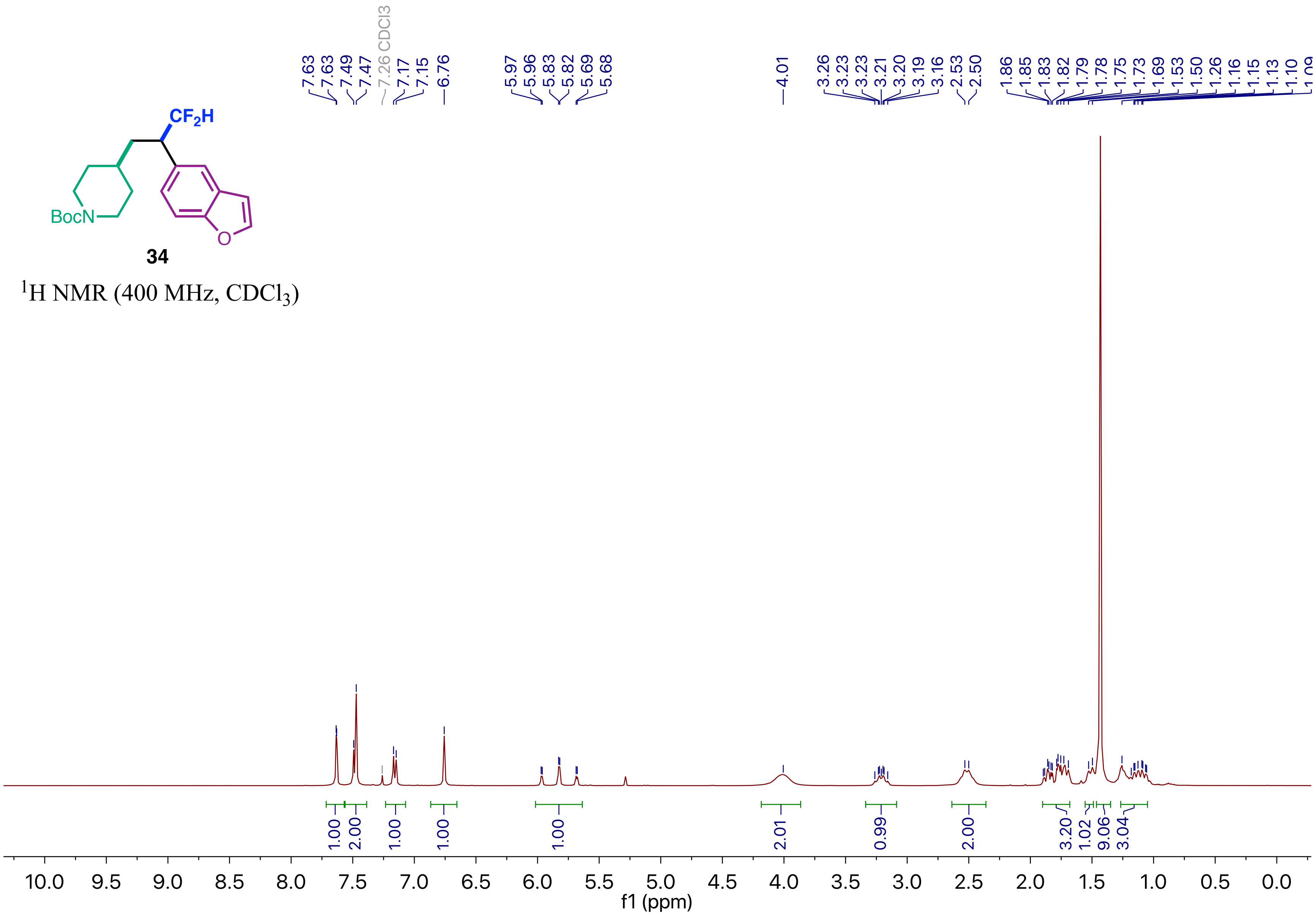
CF2H
BocN
O
34
1H NMR (400 MHz, CDCl3)
7.63
7.63
7.49
7.47
7.26 CDCl3
7.17
7.15
6.76
5.97
5.96
5.83
5.82
5.69
5.68
4.01
3.26
3.23
3.23
3.21
3.20
3.19
3.16
2.53
2.50
1.86
1.85
1.83
1.82
1.79
1.78
1.75
1.73
1.69
1.53
1.50
1.26
1.16
1.15
1.13
1.10
1.00
2.00
1.00
1.00
1.00
2.01
0.99
2.00
3.20
1.02
9.06
3.04
10.0
9.5
9.0
8.5
8.0
7.5
7.0
6.5
6.0
5.5
5.0
4.5
4.0
3.5
3.0
2.5
2.0
1.5
1.0
0.5
0.0
f1 (ppm)

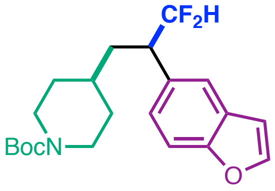

34

^{13}C NMR (101 MHz, $CDCl_3$)

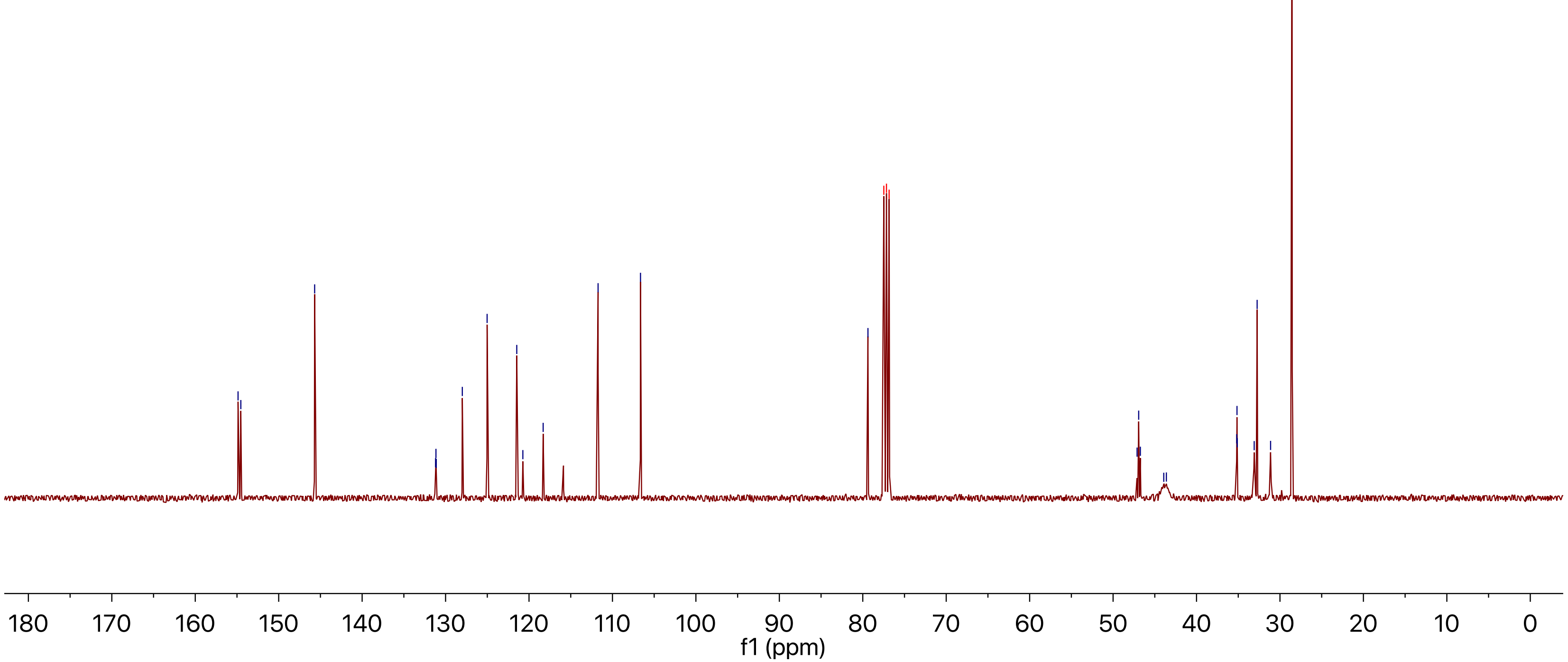

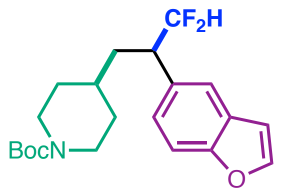

34

^{19}F NMR (376 MHz, $CDCl_3$)

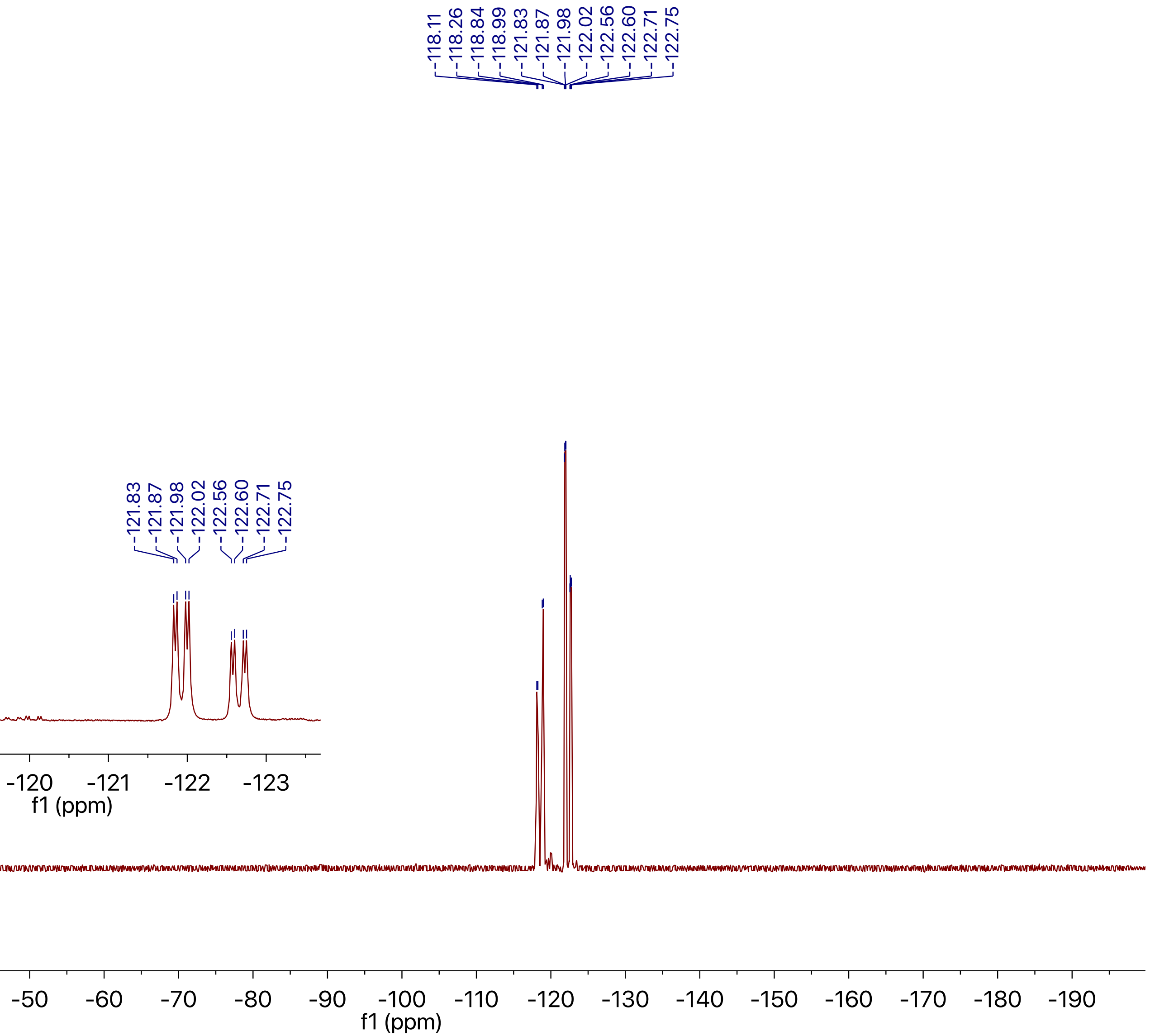

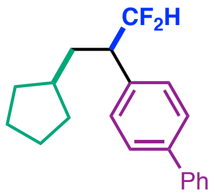

35

^1H NMR (400 MHz, $CDCl_3$)

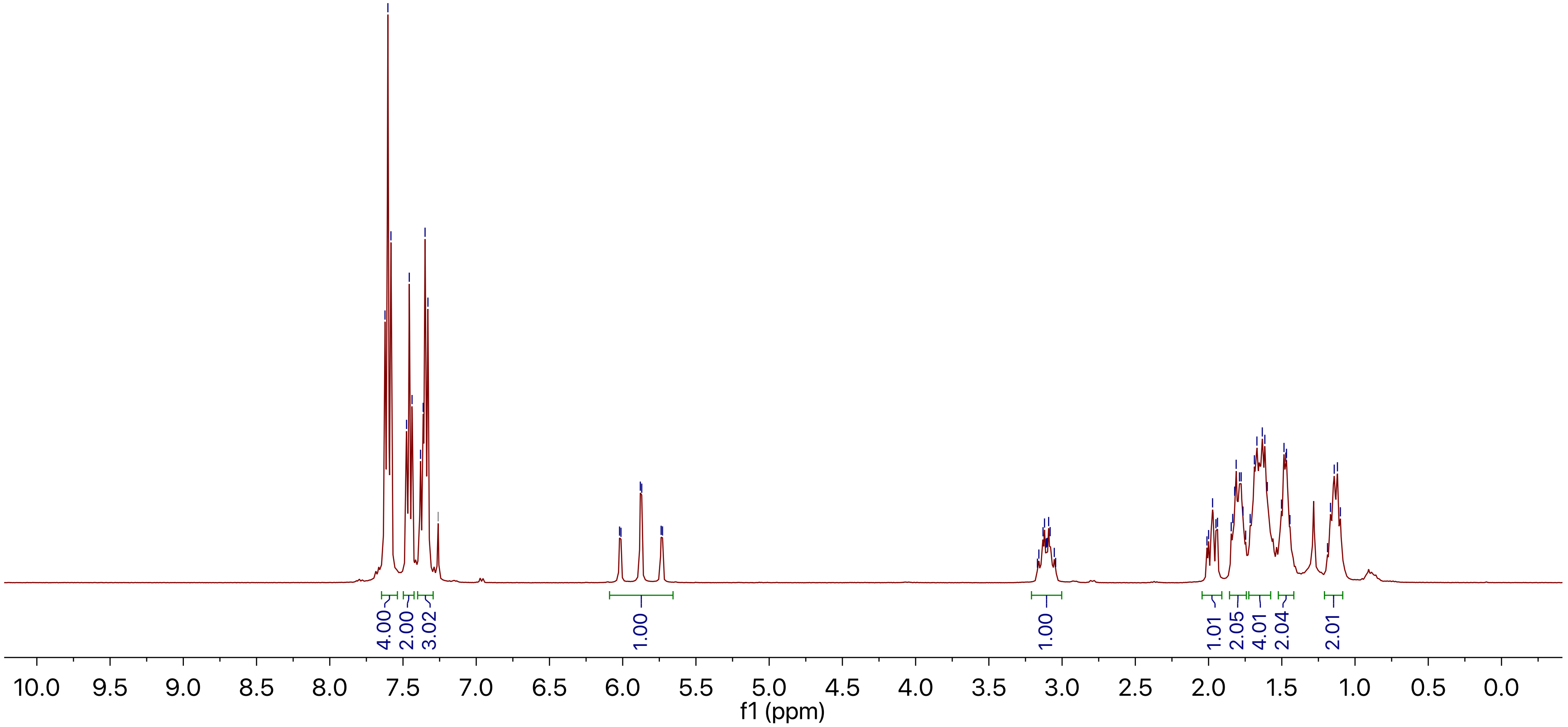

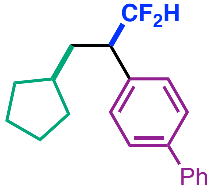

35

^{13}C NMR (101 MHz, $CDCl_3$)

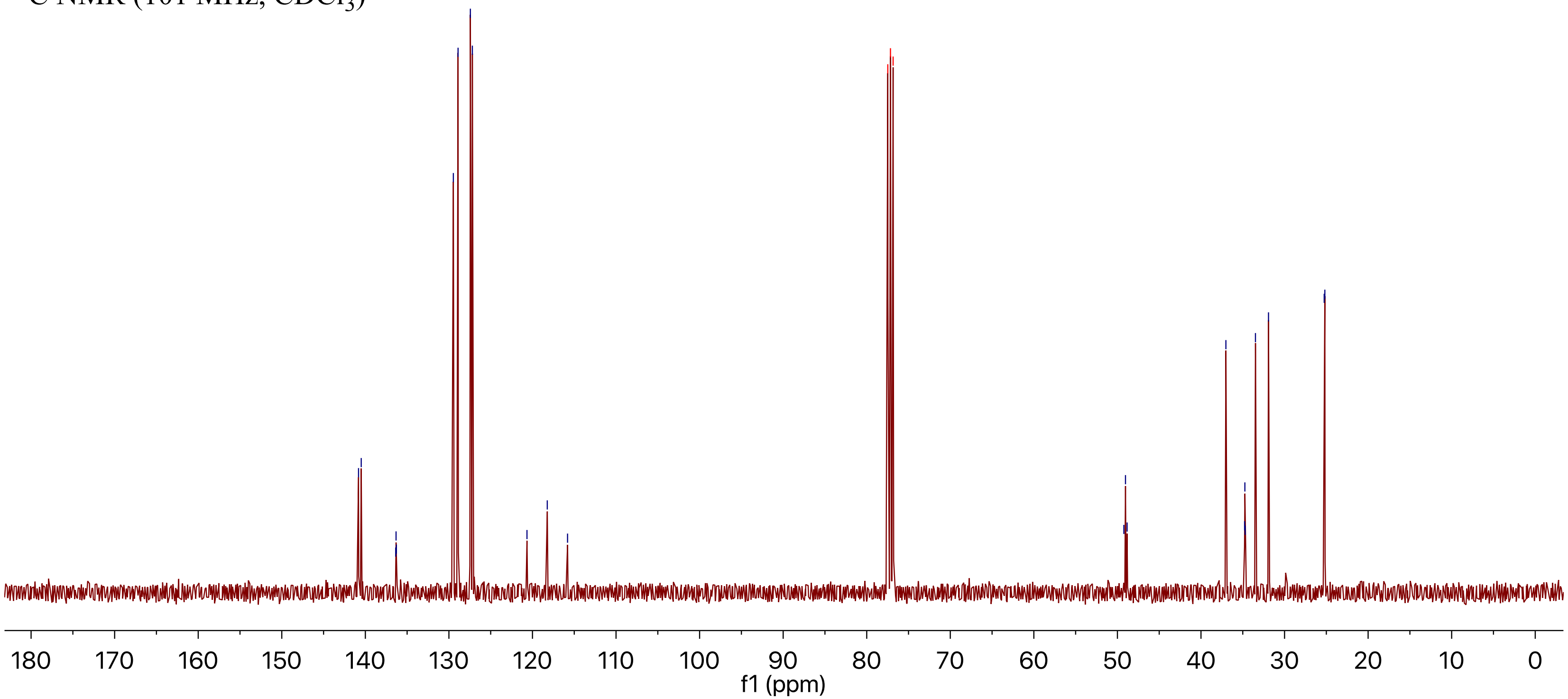

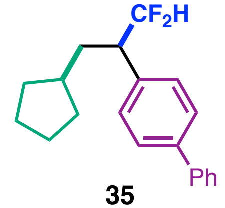

35

^{19}F NMR (376 MHz, CDCl$_3$)

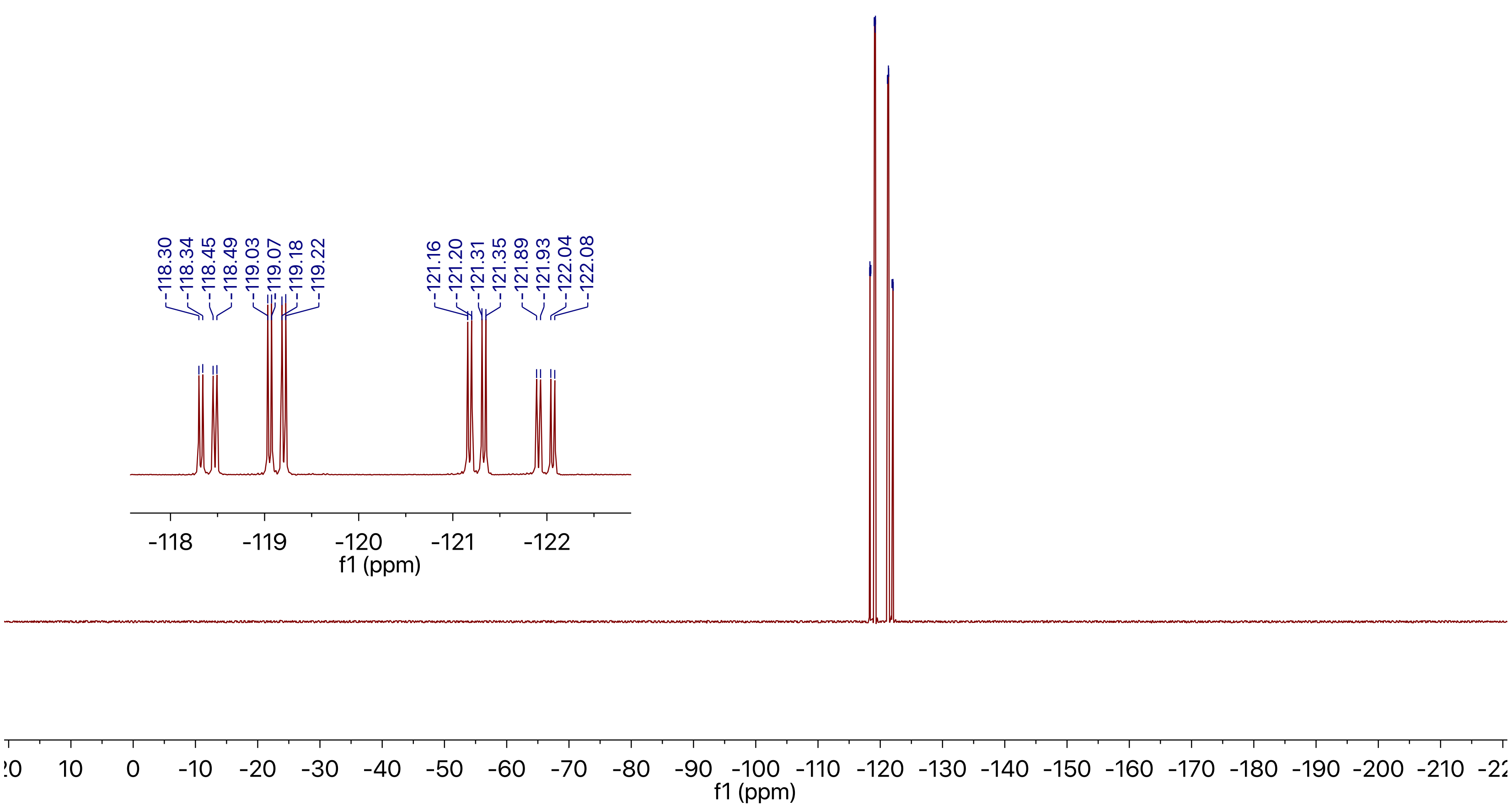

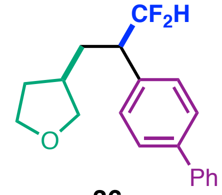

36

^{1}H NMR (400 MHz, $CDCl_3$)

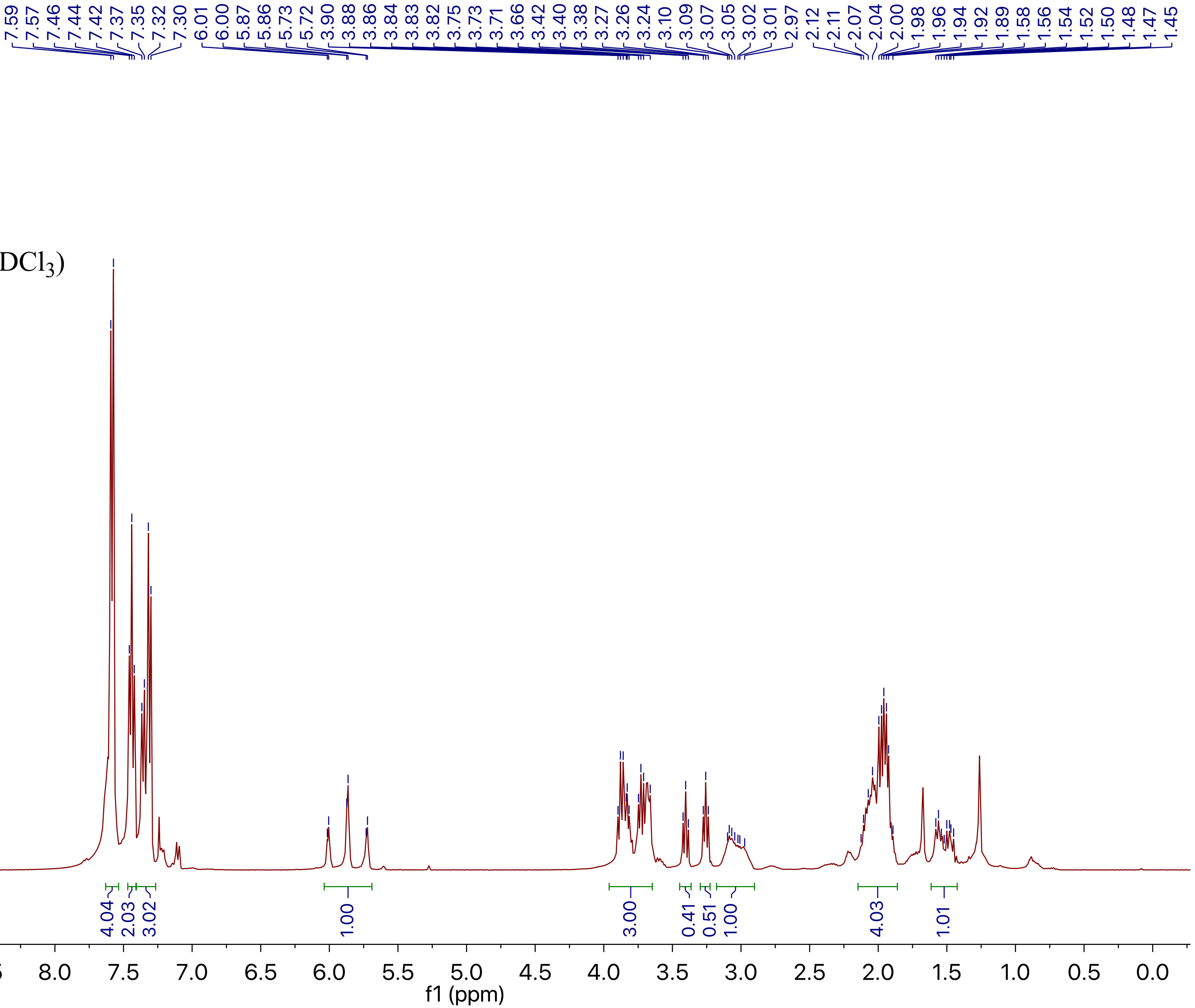

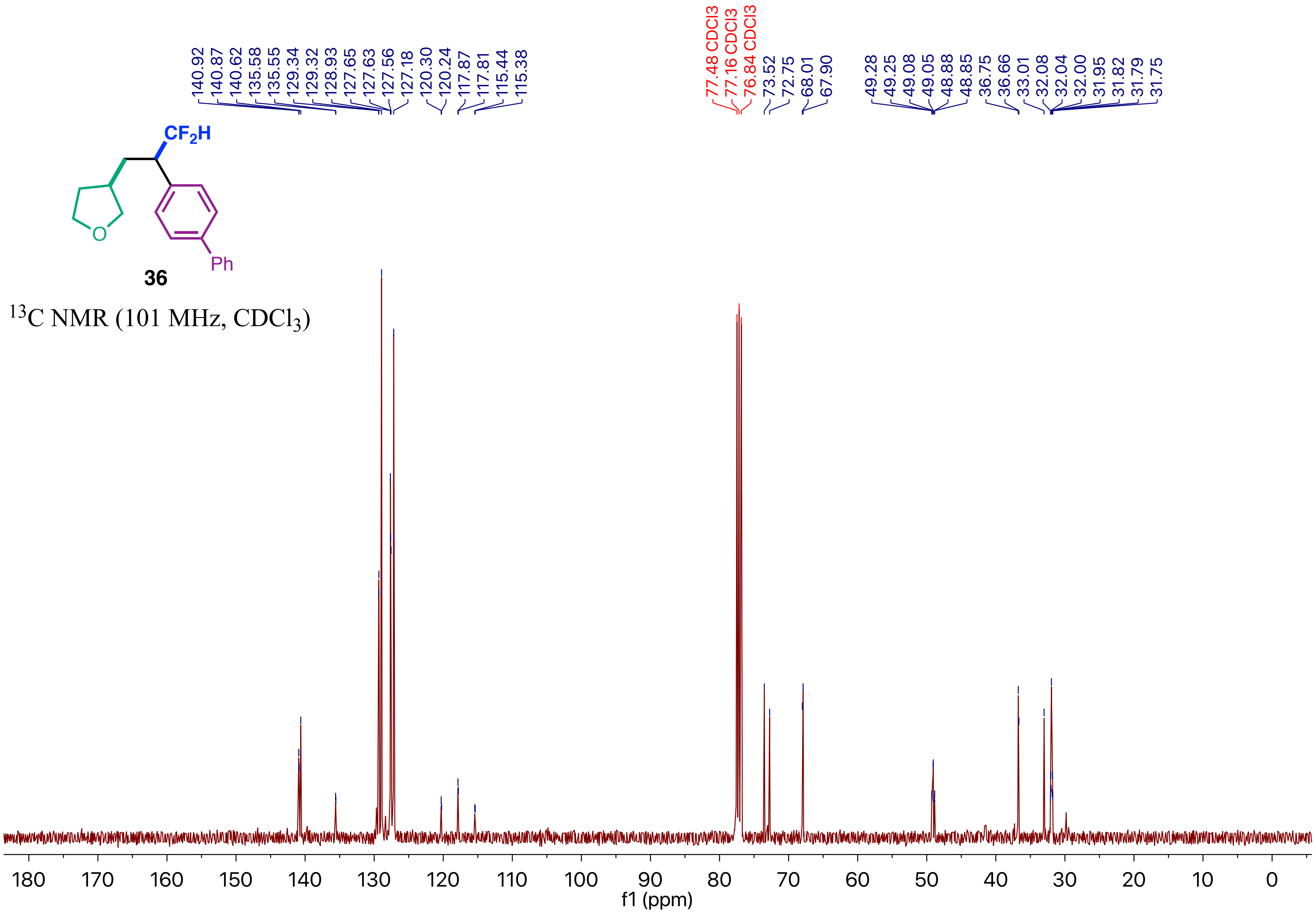

^{13}C NMR (101 MHz, $CDCl_3$)

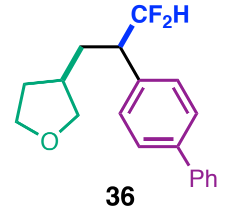

36

^{19}F NMR (376 MHz, $CDCl_3$)

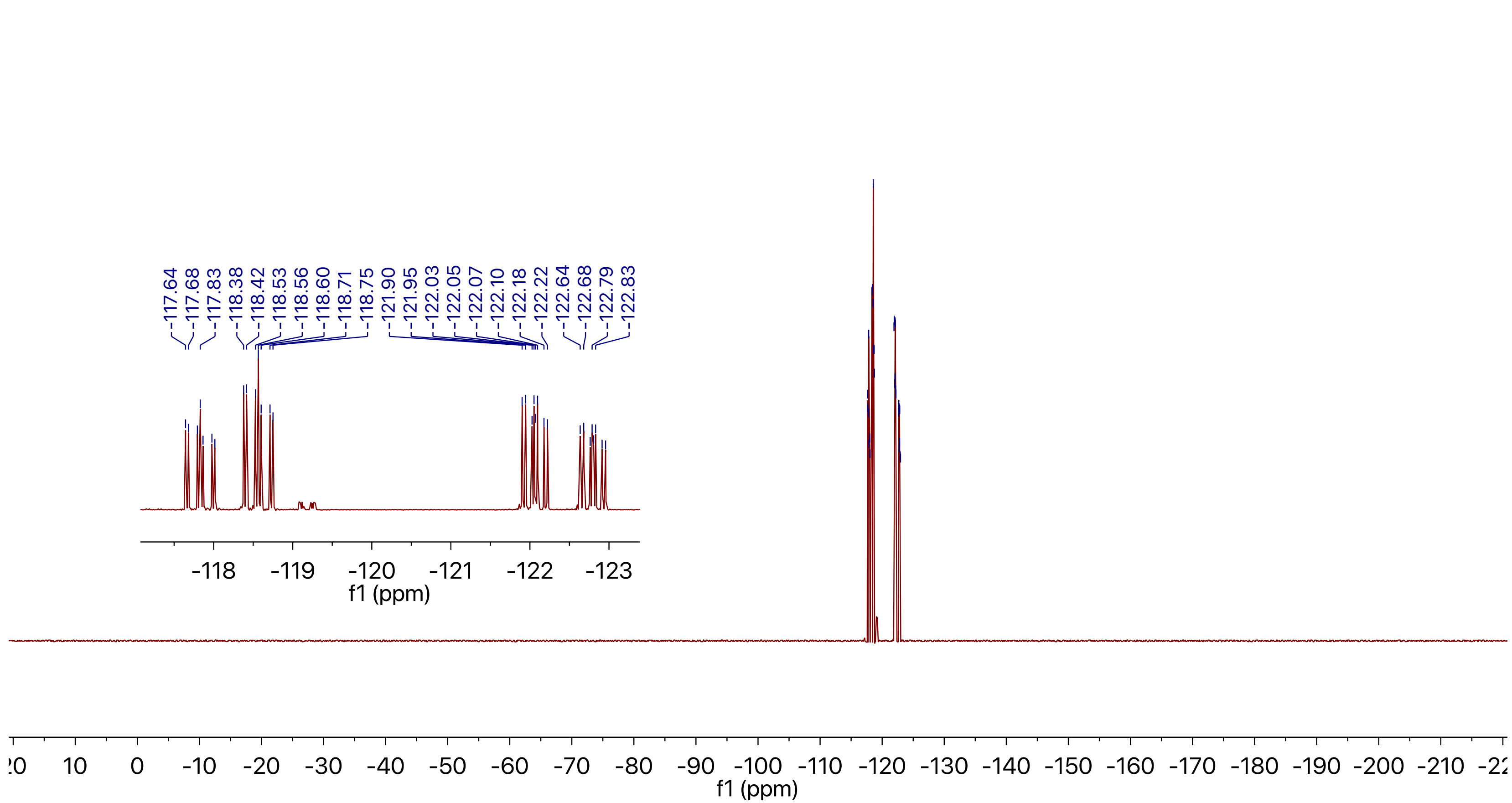

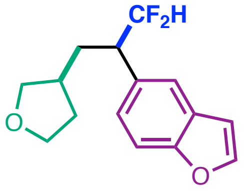

37

^{1}H NMR (400 MHz, $CDCl_3$)

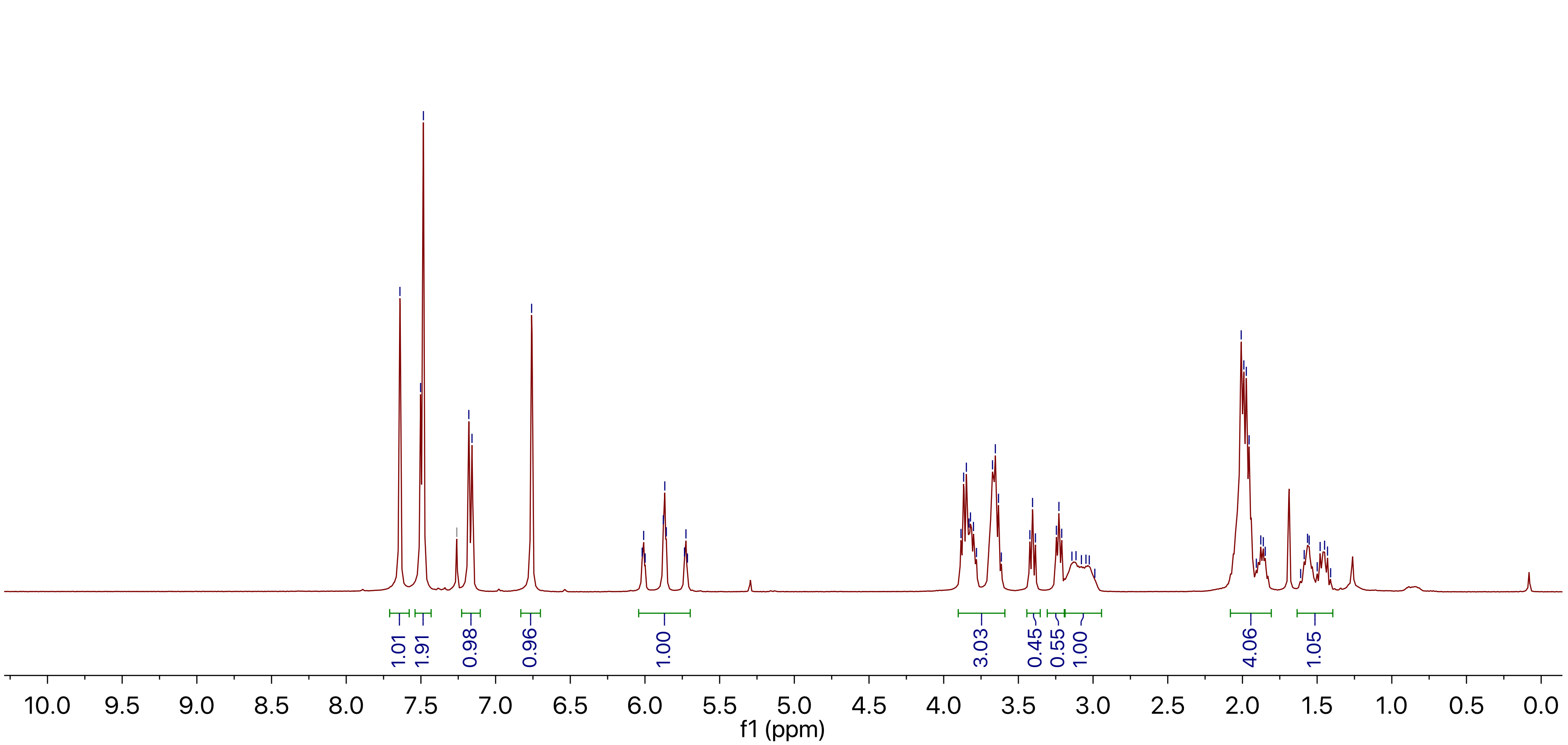

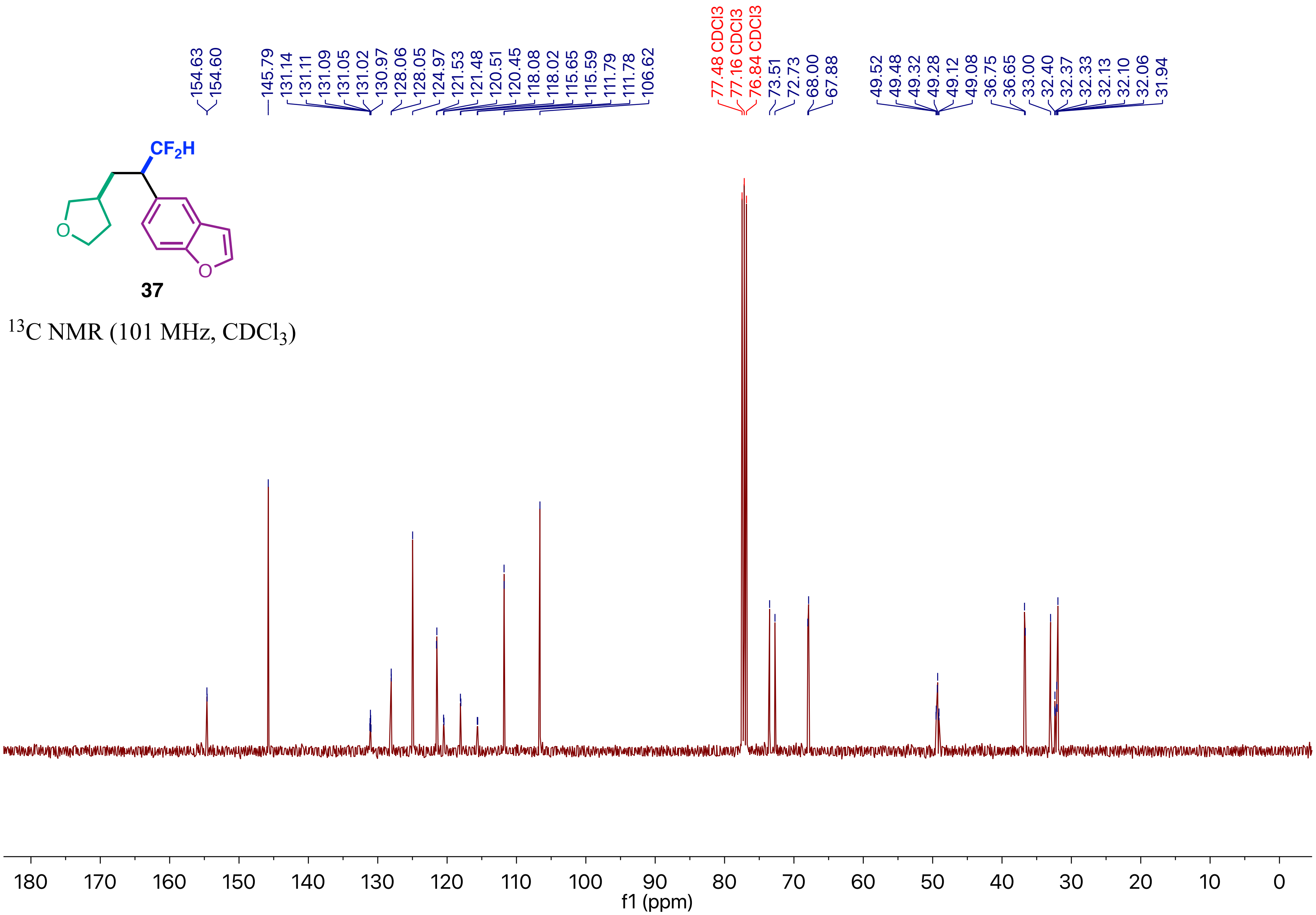
154.63
154.60
145.79
131.14
131.11
131.09
131.05
131.02
130.97
128.06
128.05
124.97
121.53
121.48
120.51
120.45
118.08
118.02
115.65
115.59
111.79
111.78
106.62
77.48 CDCl3
77.16 CDCl3
76.84 CDCl3
73.51
72.73
68.00
67.88
49.52
49.48
49.32
49.28
49.12
49.08
36.75
36.65
33.00
32.40
32.37
32.33
32.13
32.10
32.06
31.94
CF2H
O
O
37
13C NMR (101 MHz, CDCl3)
180 170 160 150 140 130 120 110 100 90 80 70 60 50 40 30 20 10 0
f1 (ppm)

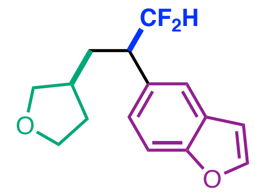

37

^{19}F NMR (376 MHz, $CDCl_3$)

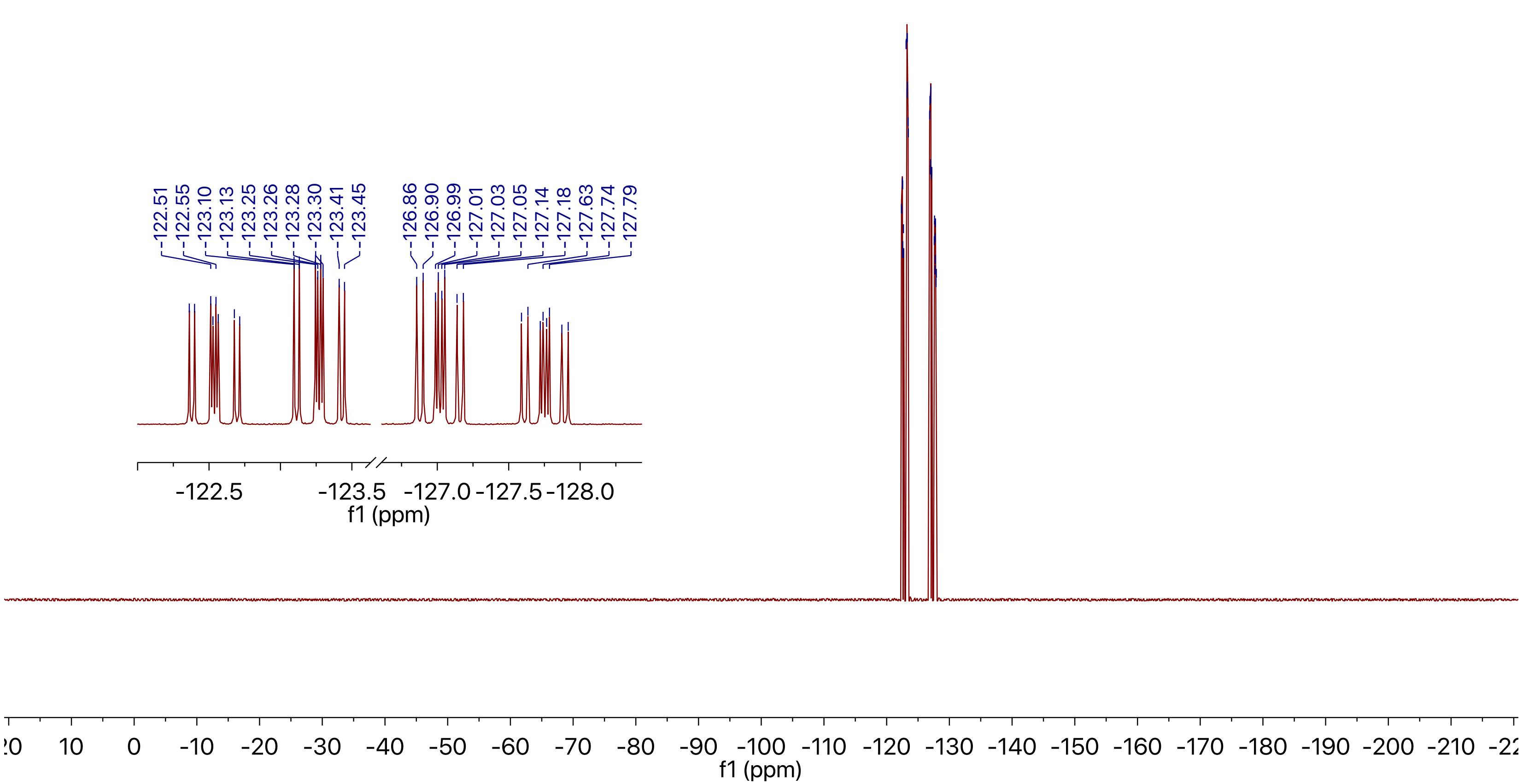

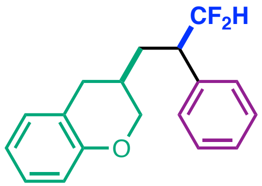

38

^1H NMR (400 MHz, $CDCl_3$)

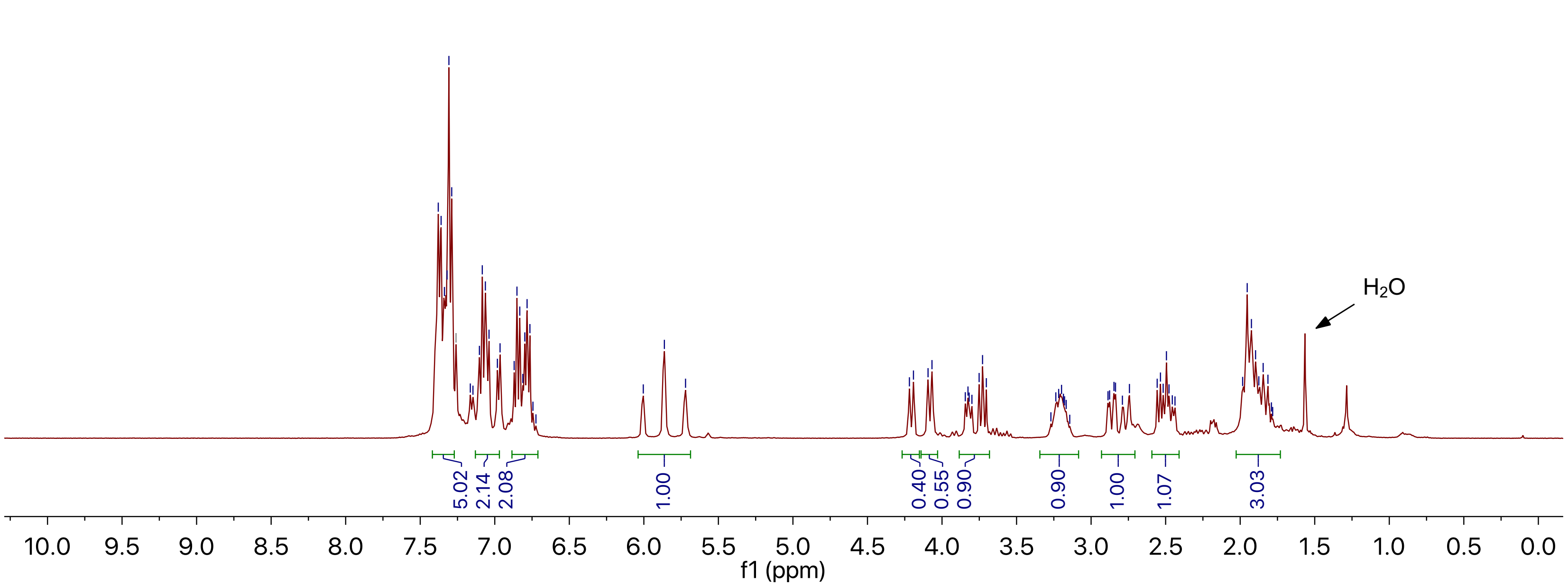

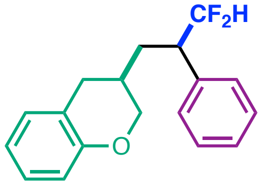

38

^{13}C NMR (101 MHz, $CDCl_3$)

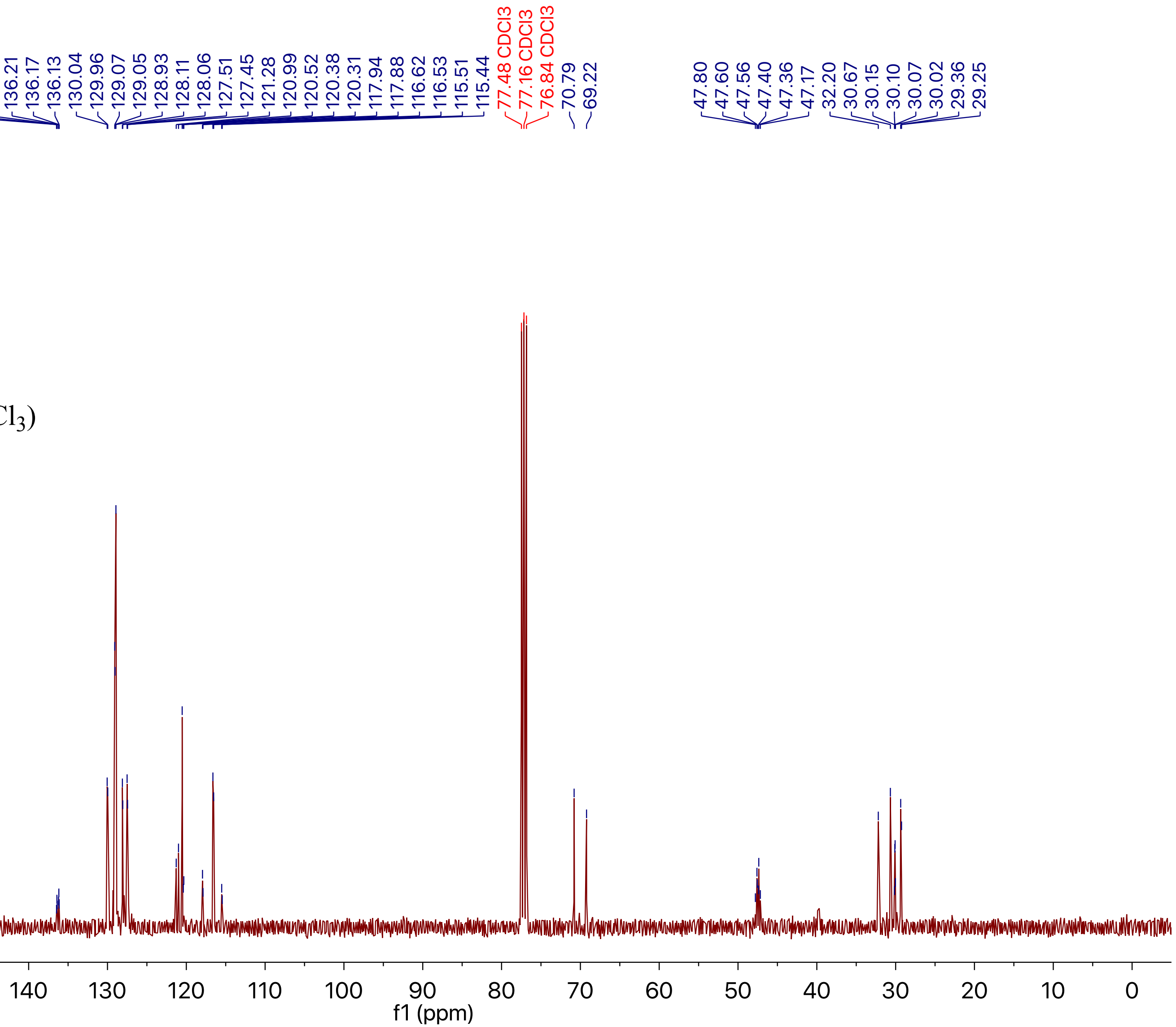

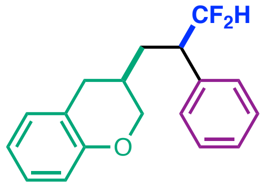

38

^{19}F NMR (376 MHz, $CDCl_3$)

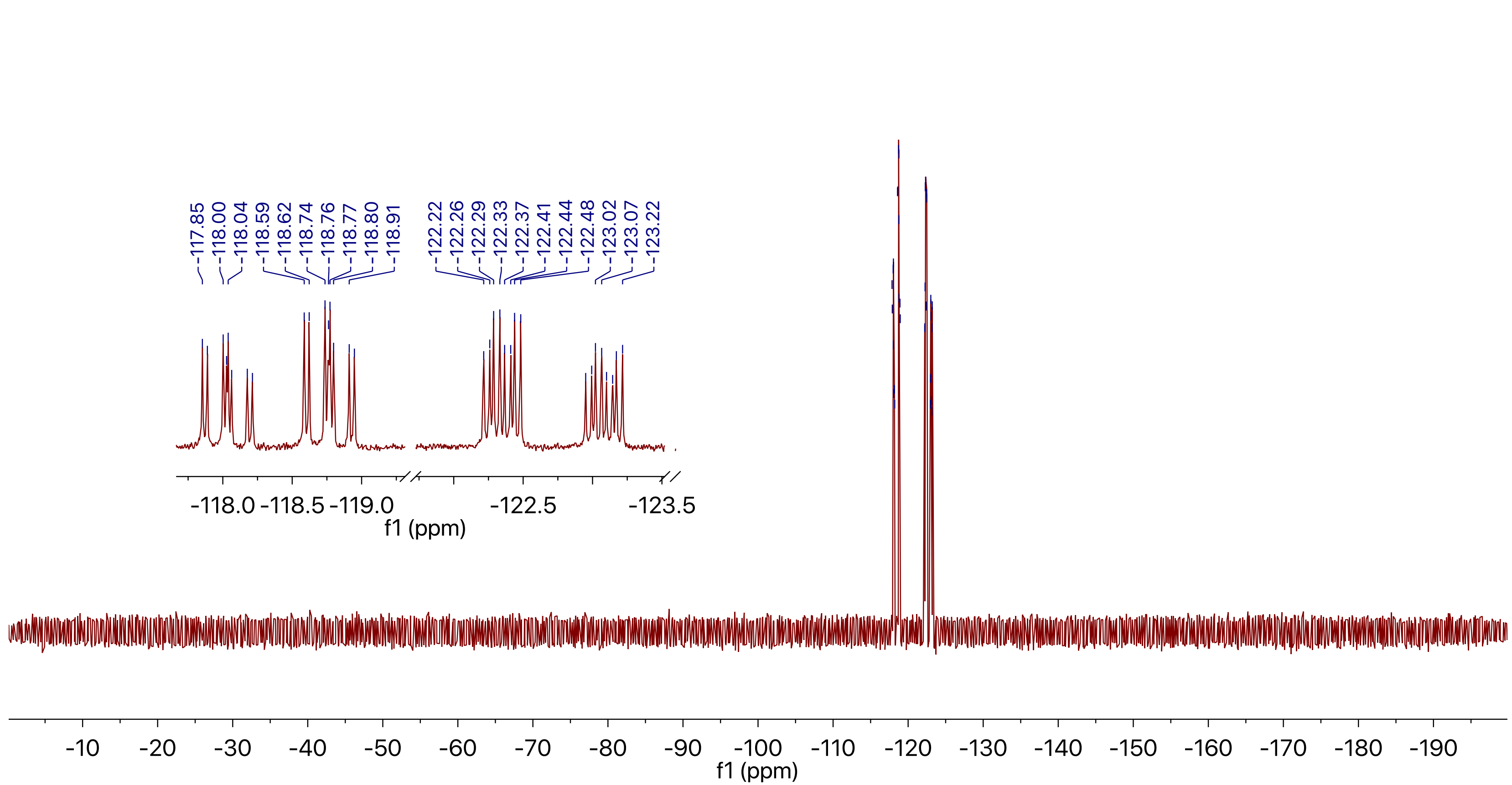

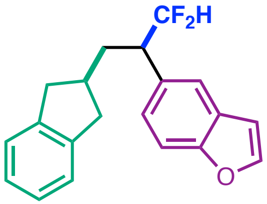

39

^{1}H NMR (400 MHz, $CDCl_3$)

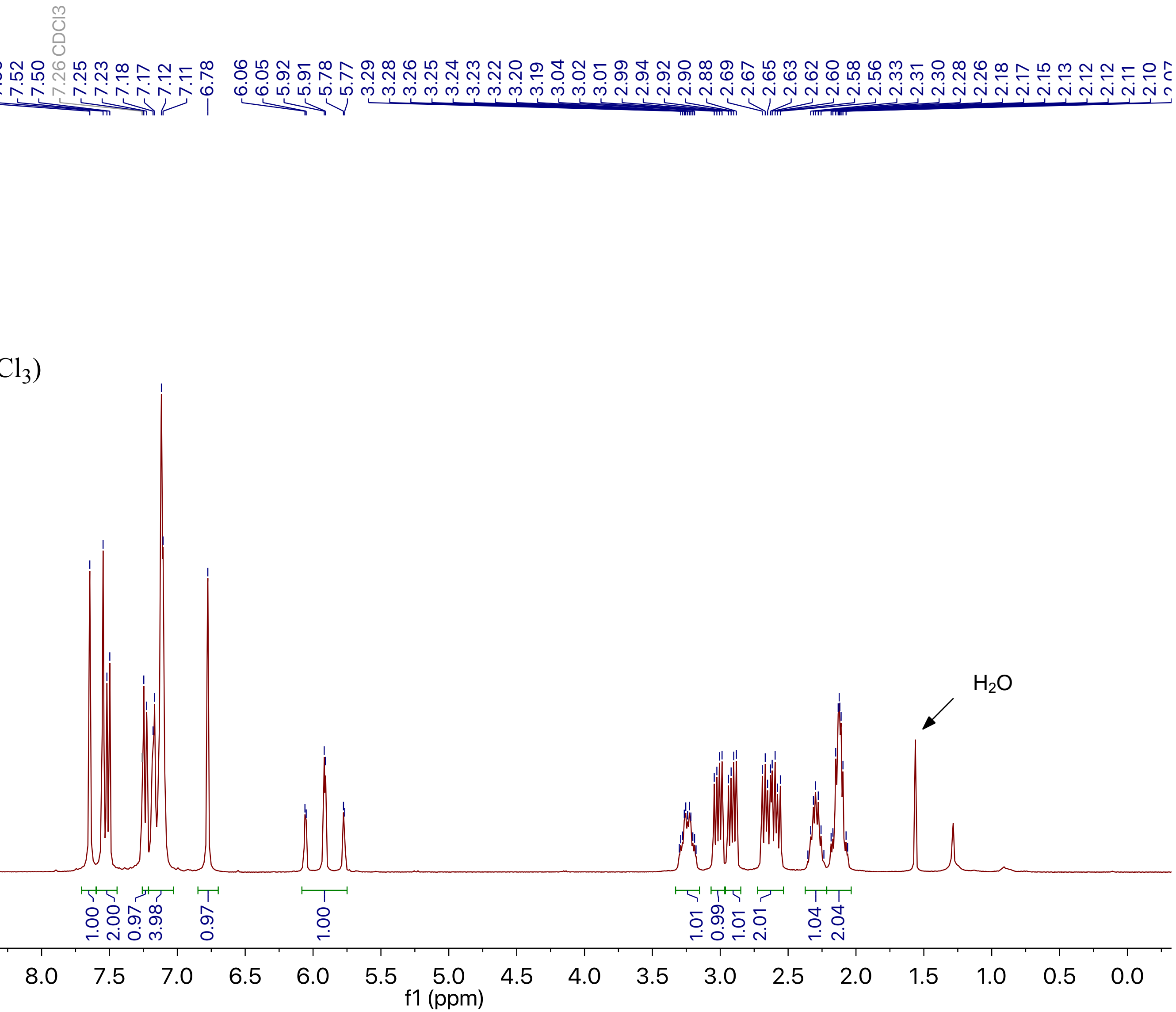

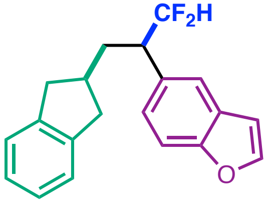

39

^{13}C NMR (101 MHz, CDCl$_3$)

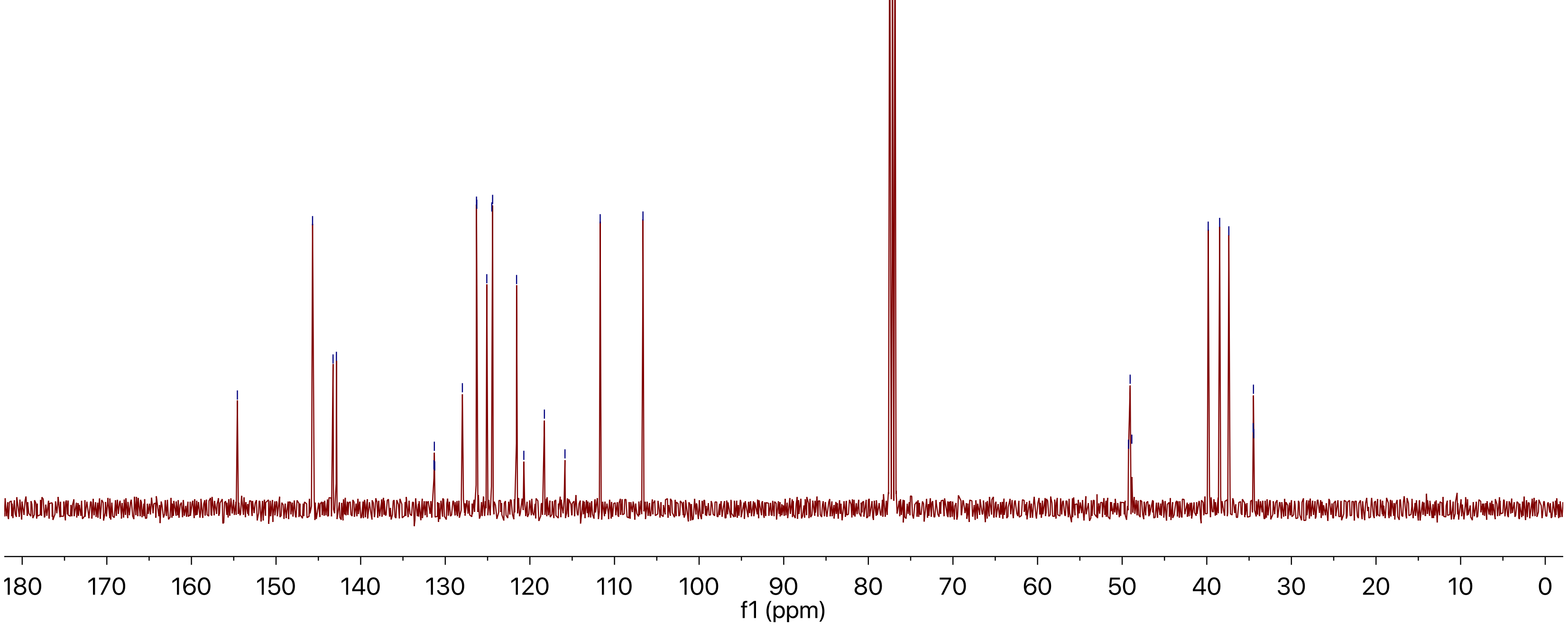

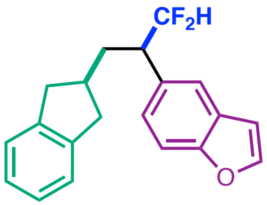

39

^{19}F NMR (376 MHz, $CDCl_3$)

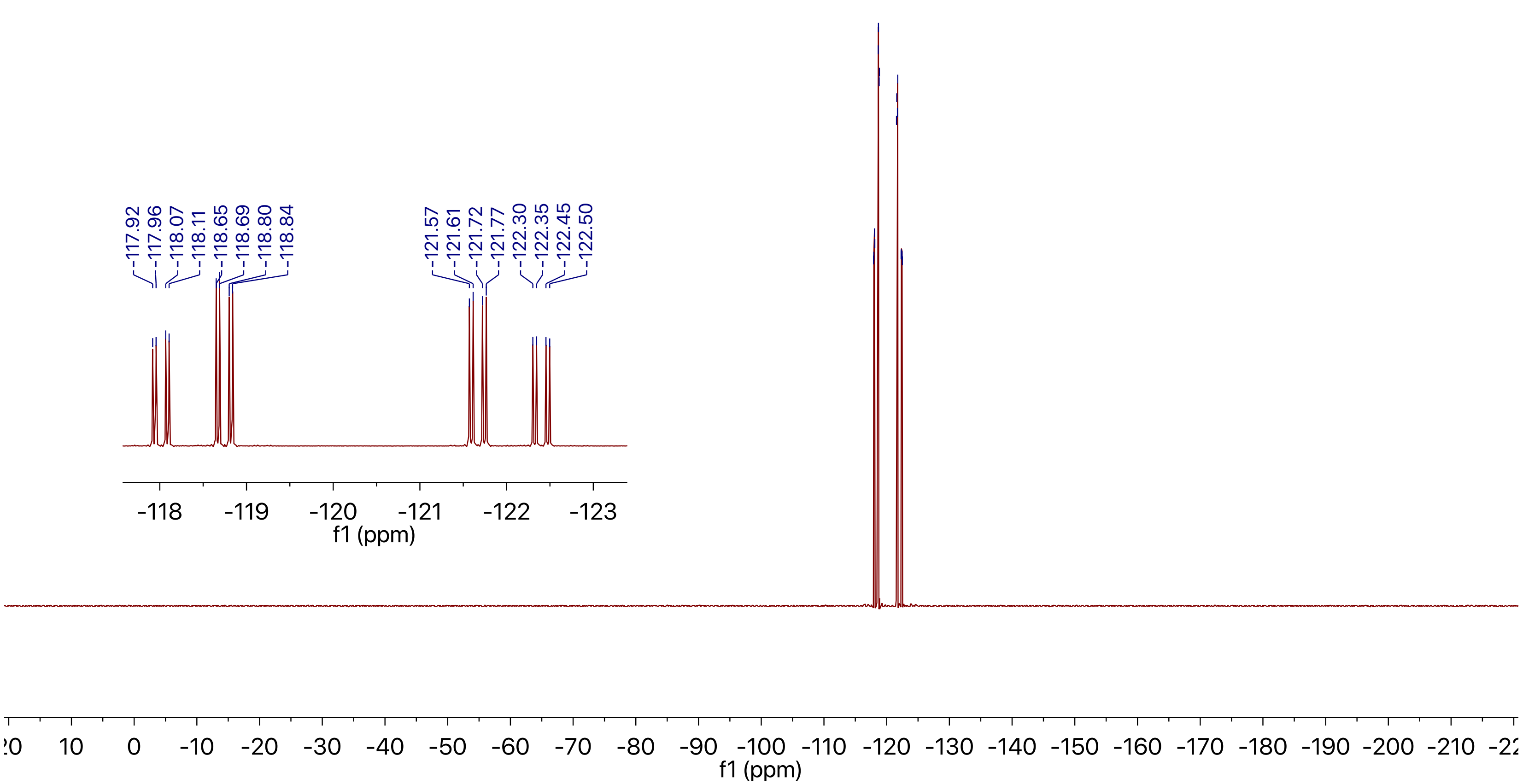

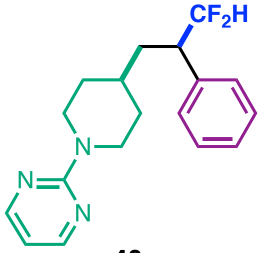

40

^1H NMR (400 MHz, $CDCl_3$)

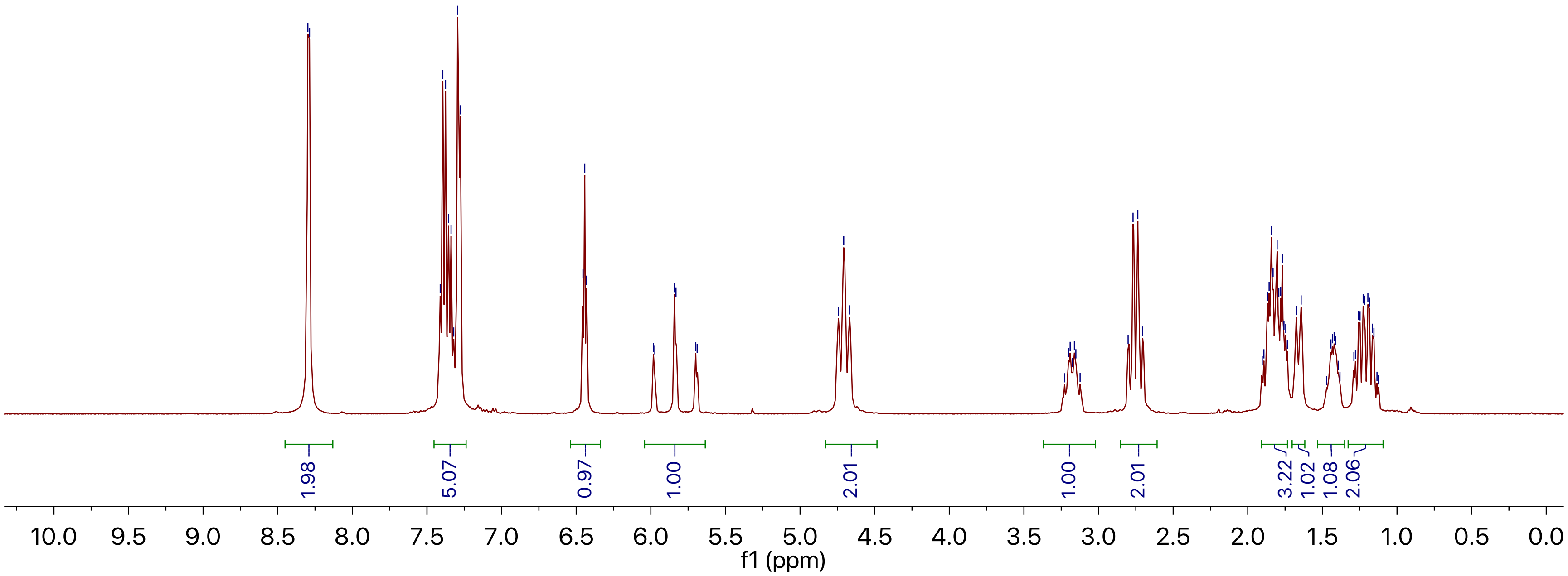

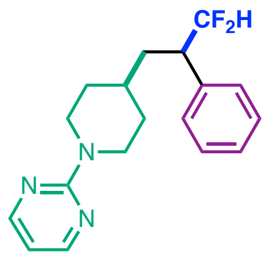

40

^{13}C NMR (101 MHz, $CDCl_3$)

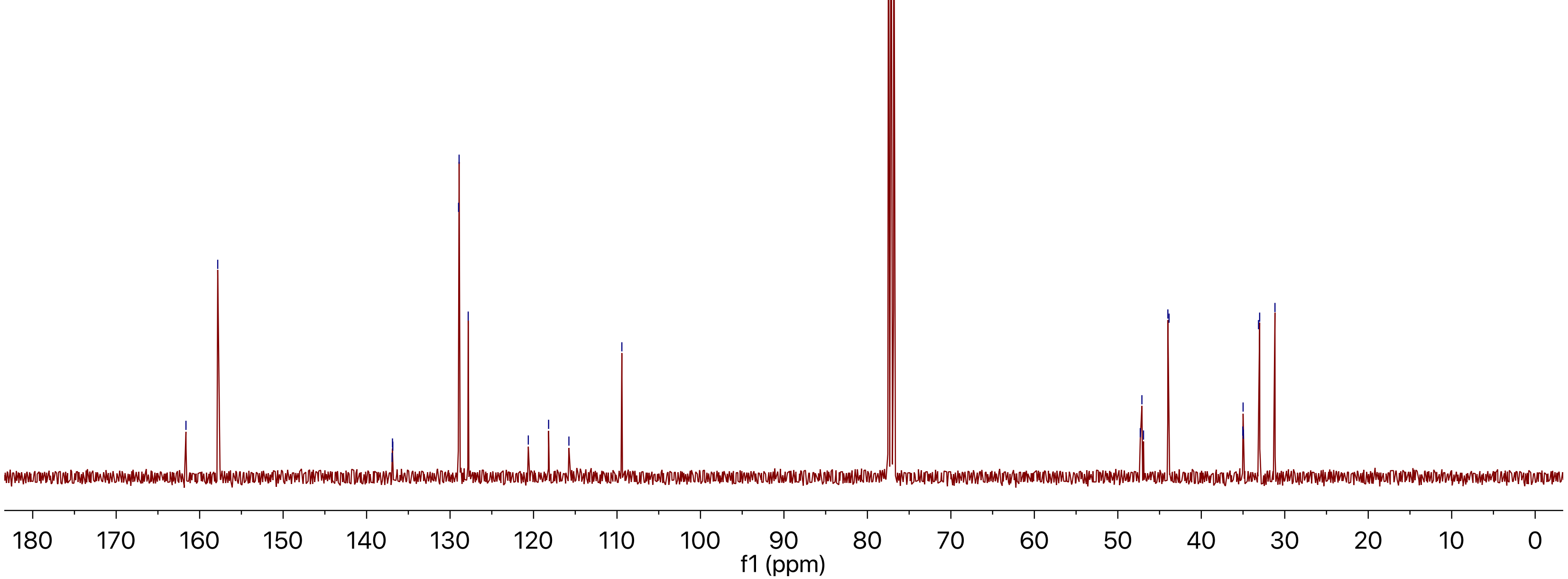

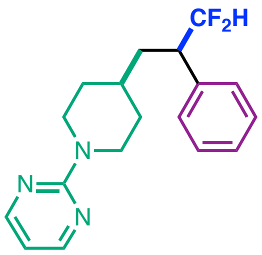

40

^{19}F NMR (376 MHz, $CDCl_3$)

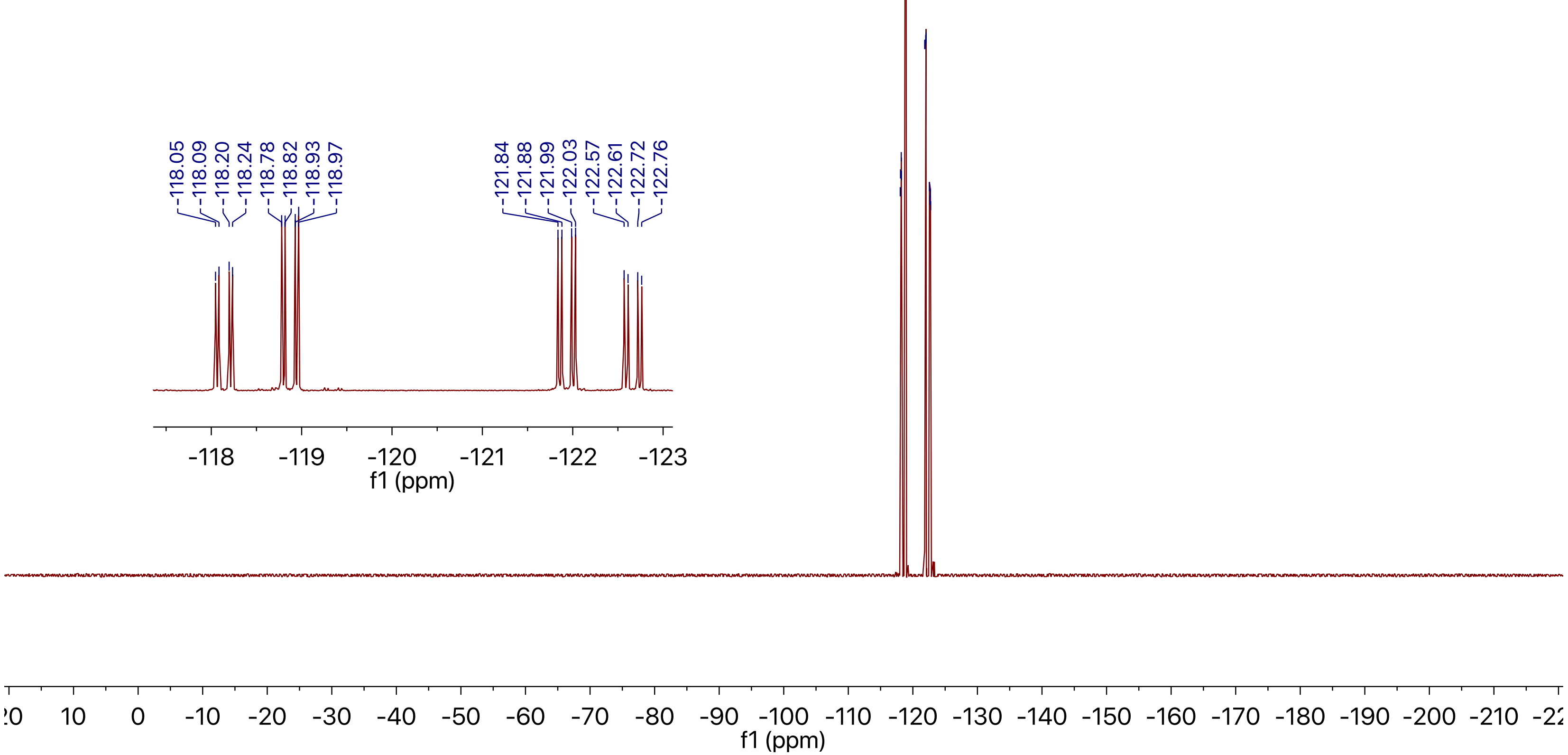

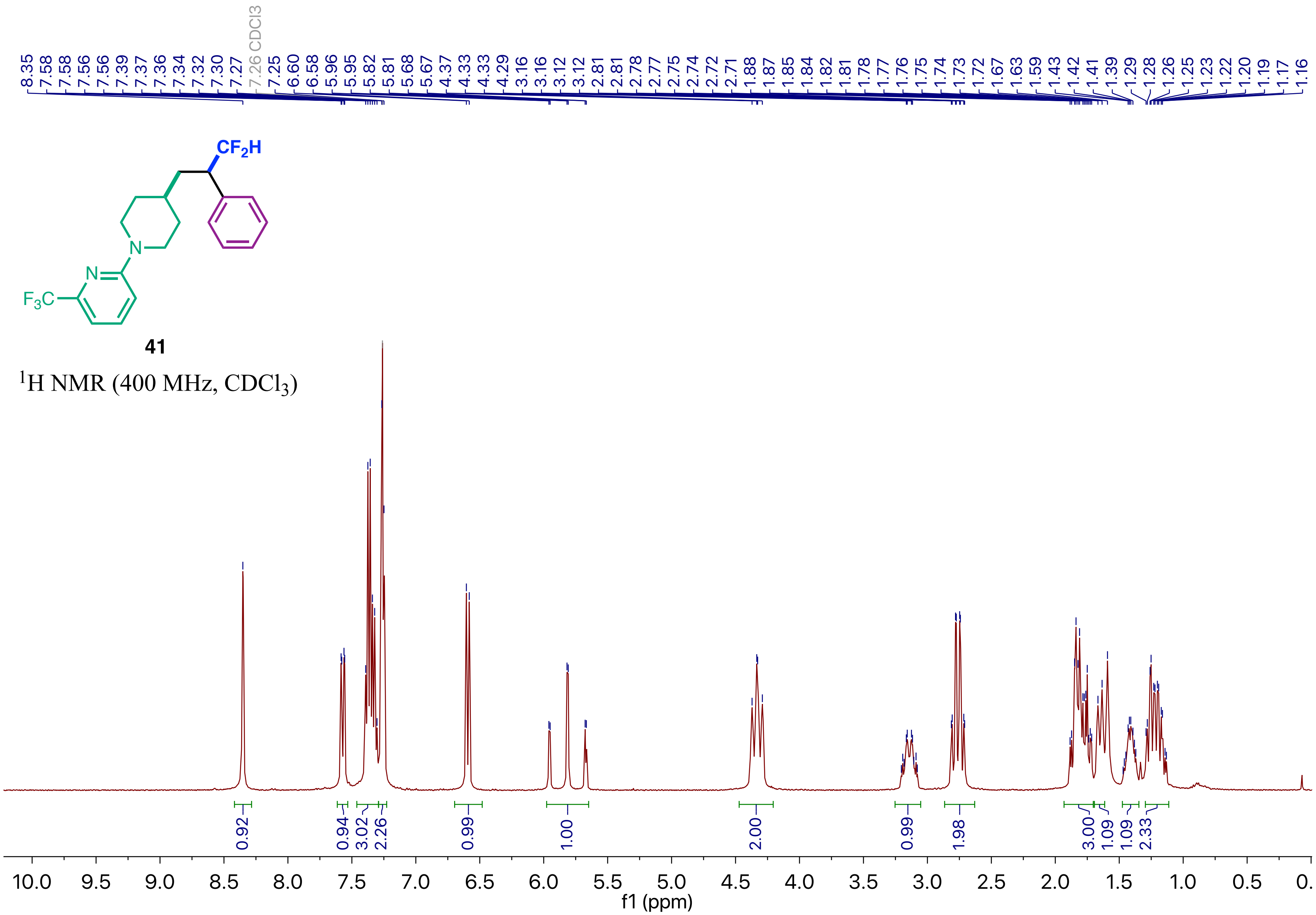

41

^{1}H NMR (400 MHz, $CDCl_3$)

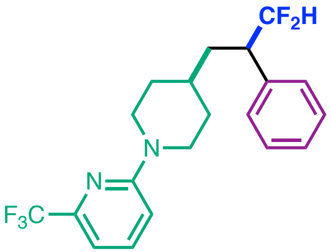

41

^{13}C NMR (101 MHz, $CDCl_3$)

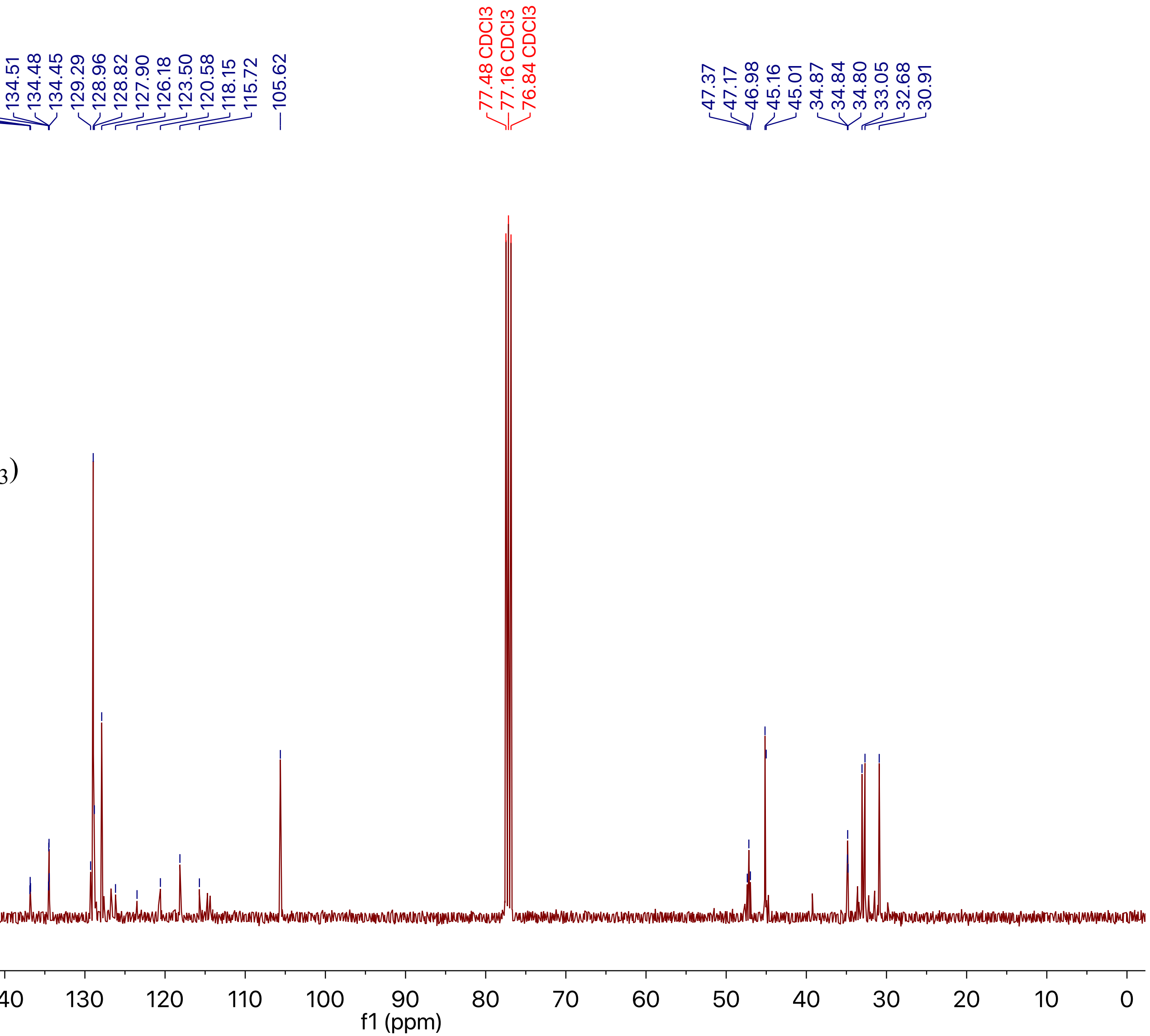

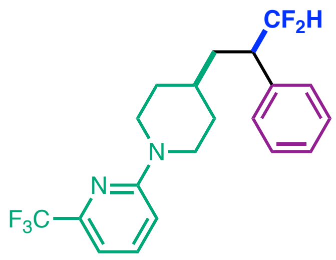

41

^{19}F NMR (376 MHz, $CDCl_3$)

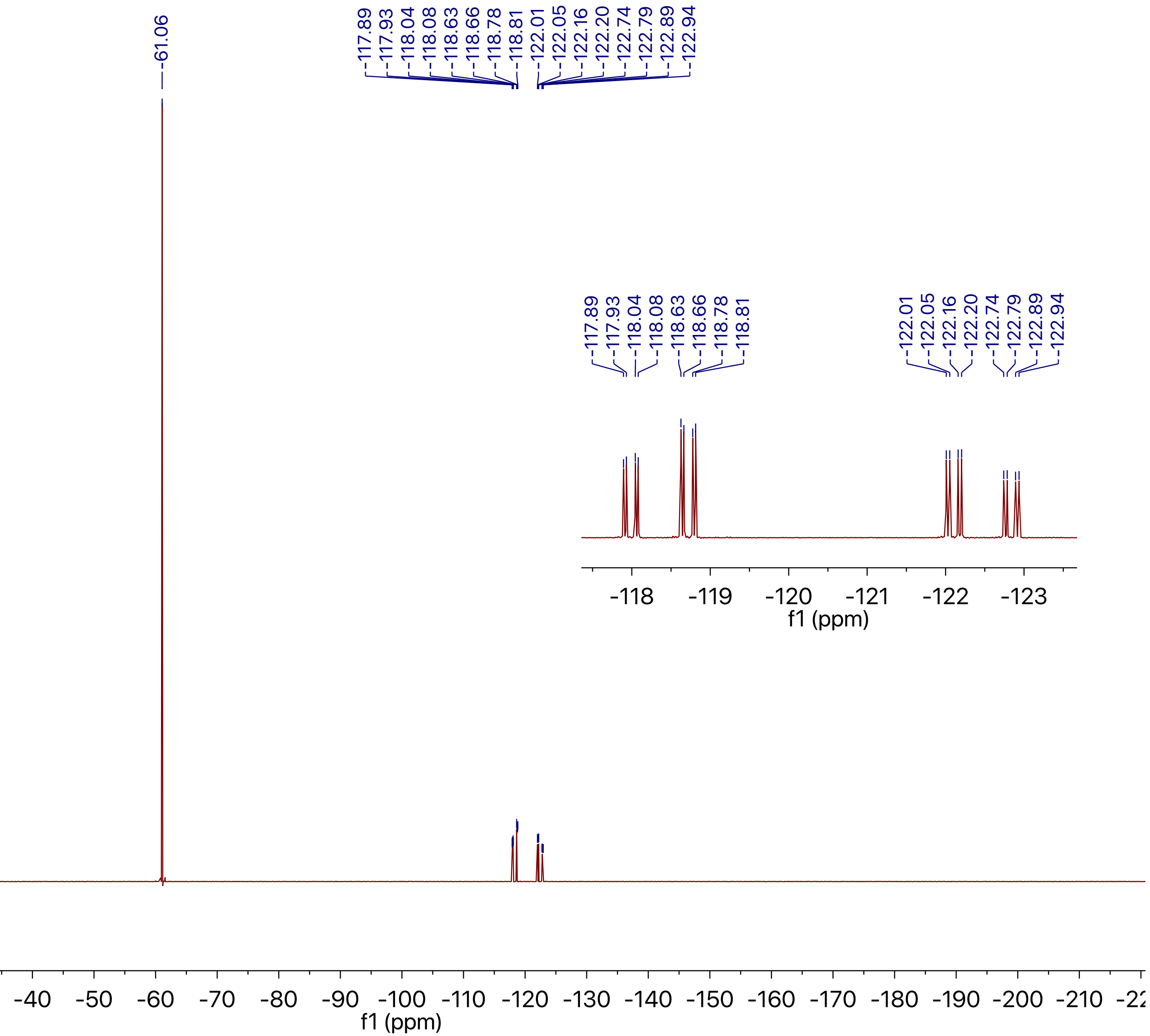

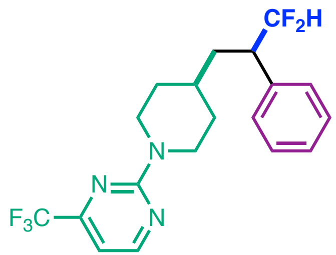

42

^{1}H NMR (400 MHz, $CDCl_3$)

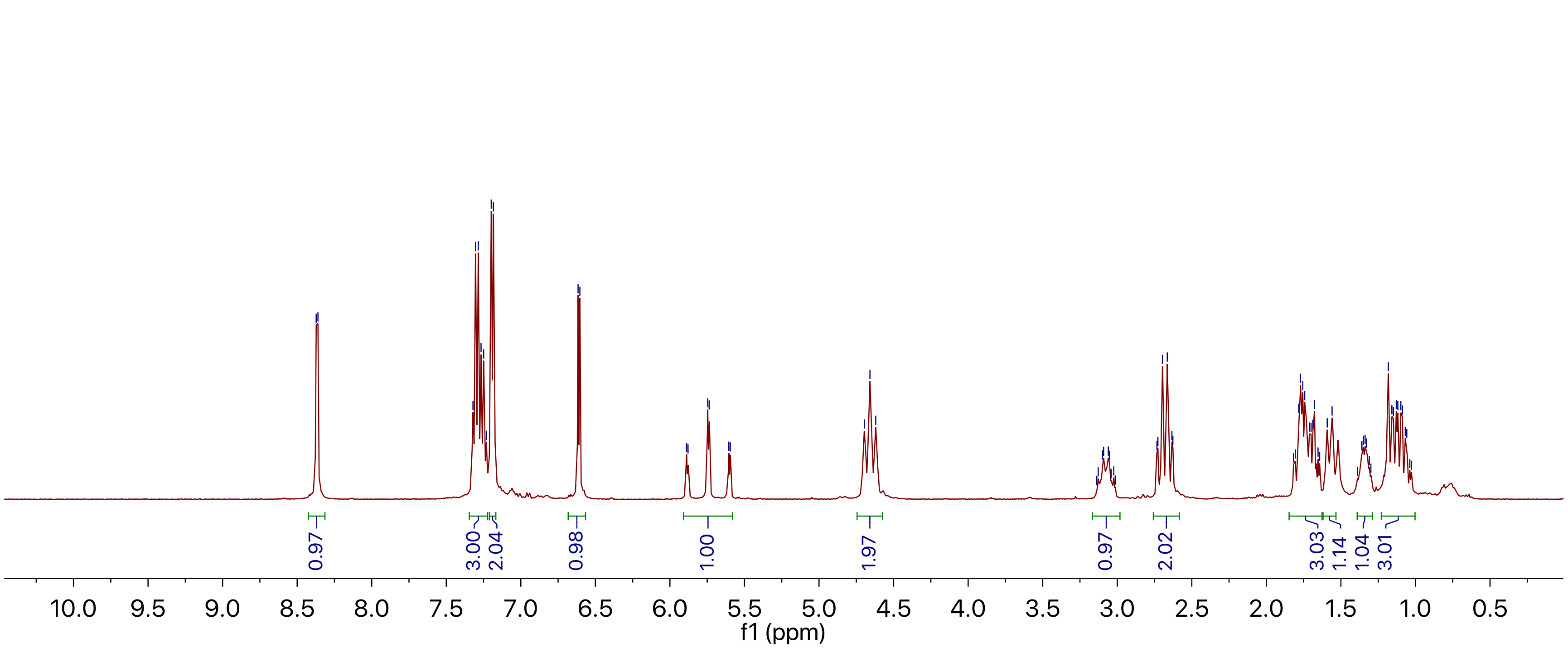

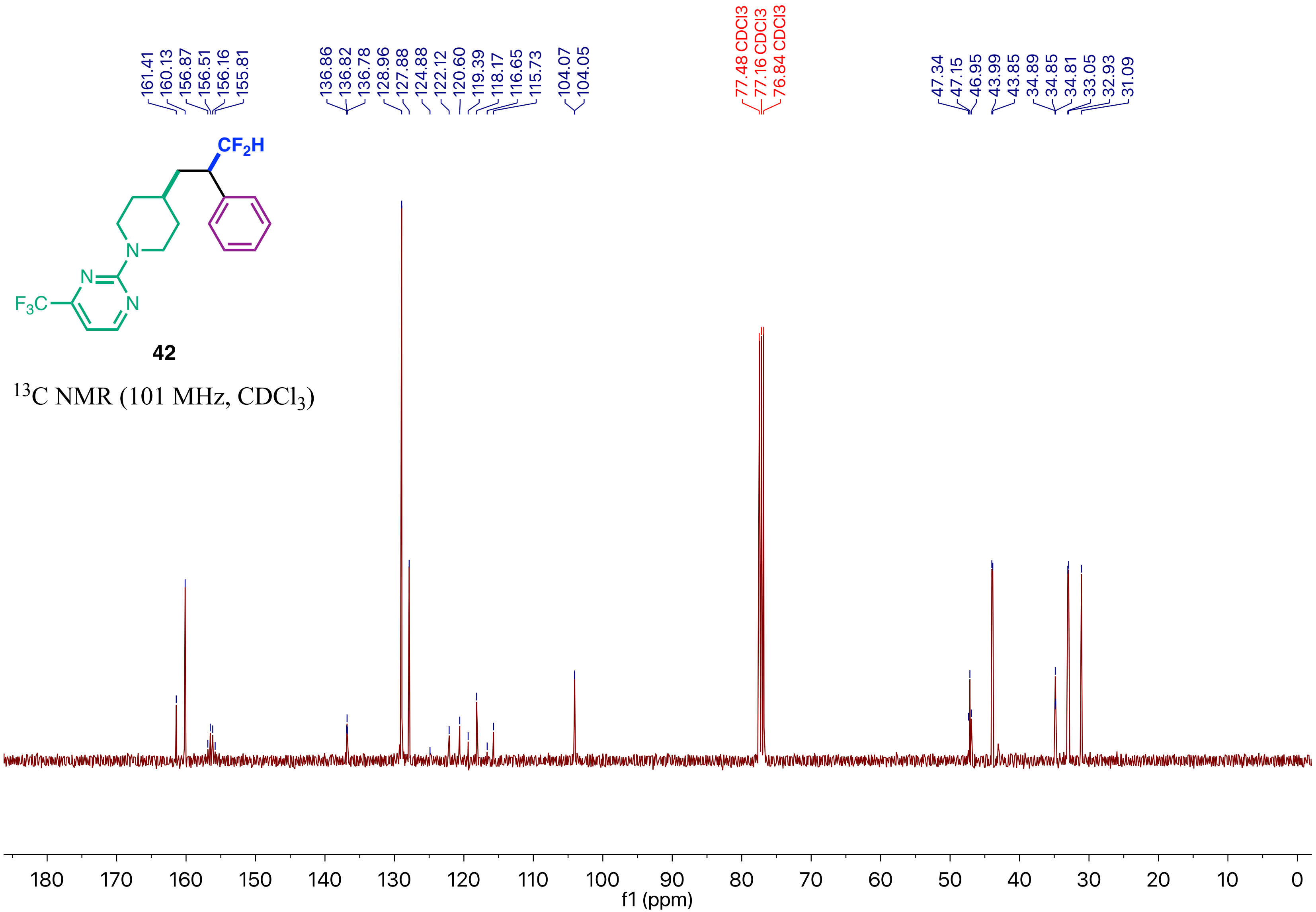

161.41
160.13
156.87
156.51
156.16
155.81
136.86
136.82
136.78
128.96
127.88
124.88
122.12
120.60
119.39
118.17
116.65
115.73
104.07
104.05
77.48 CDCl3
77.16 CDCl3
76.84 CDCl3
47.34
47.15
46.95
43.99
43.85
34.89
34.85
34.81
33.05
32.93
31.09
CF2H
N
N
N
F3C
42
13C NMR (101 MHz, CDCl3)
180
170
160
150
140
130
120
110
100
90
80
70
60
50
40
30
20
10
0
f1 (ppm)

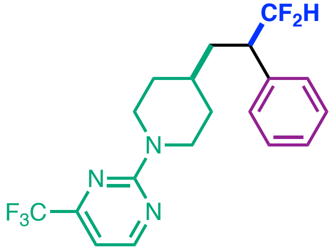

42

^{19}F NMR (376 MHz, $CDCl_3$)

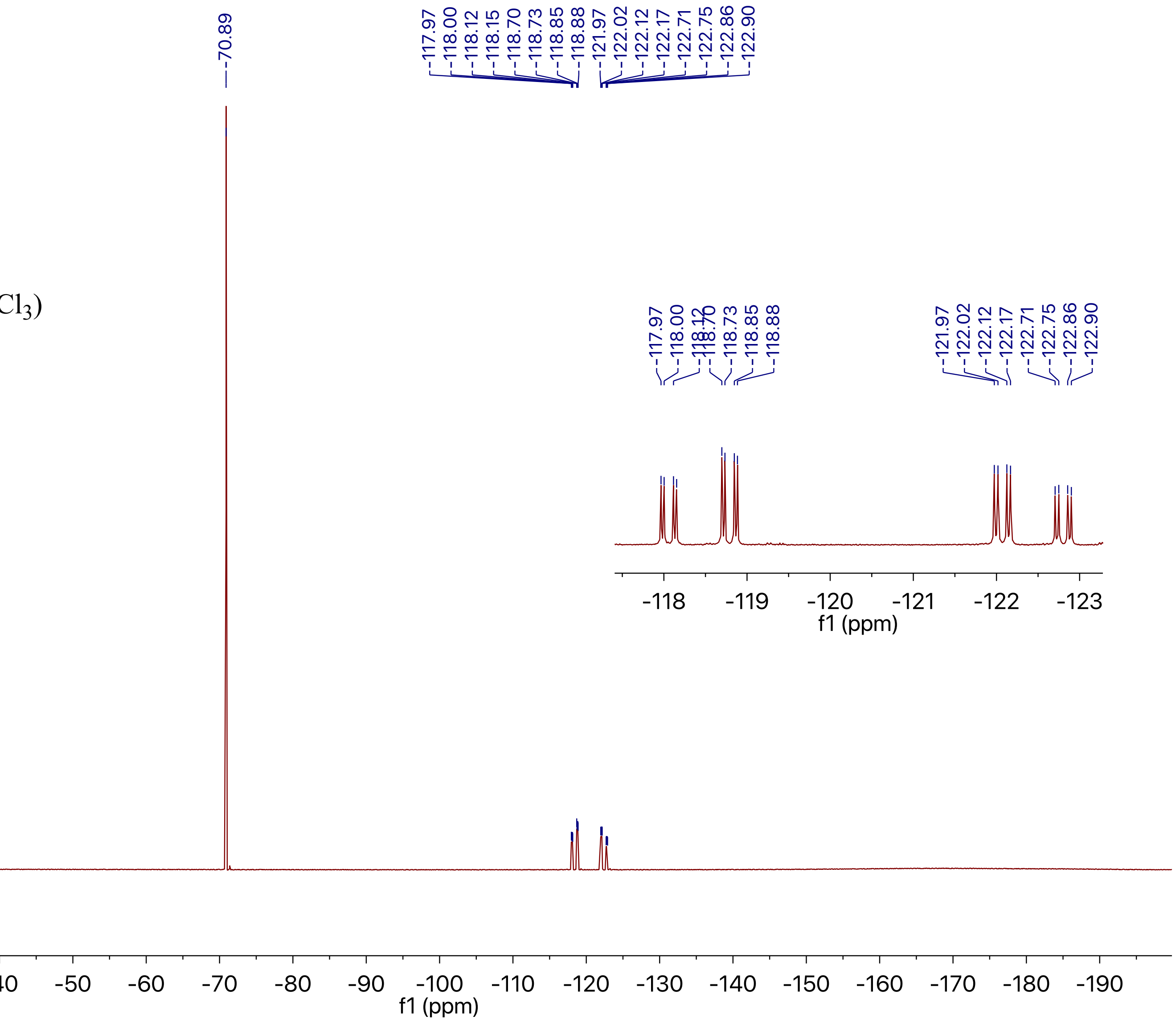

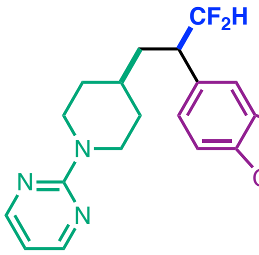

43

^{1}H NMR (400 MHz, $CDCl_3$)

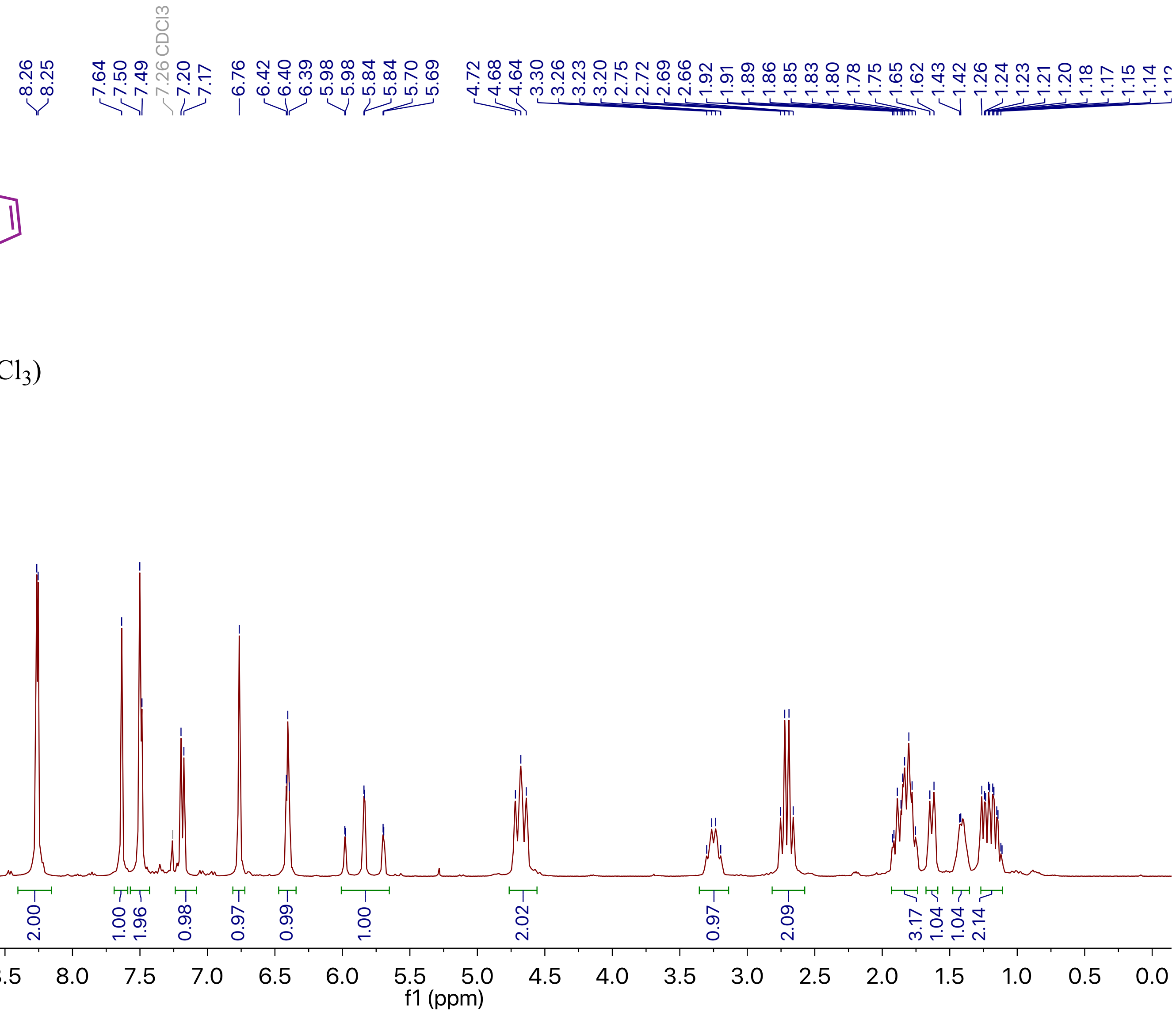

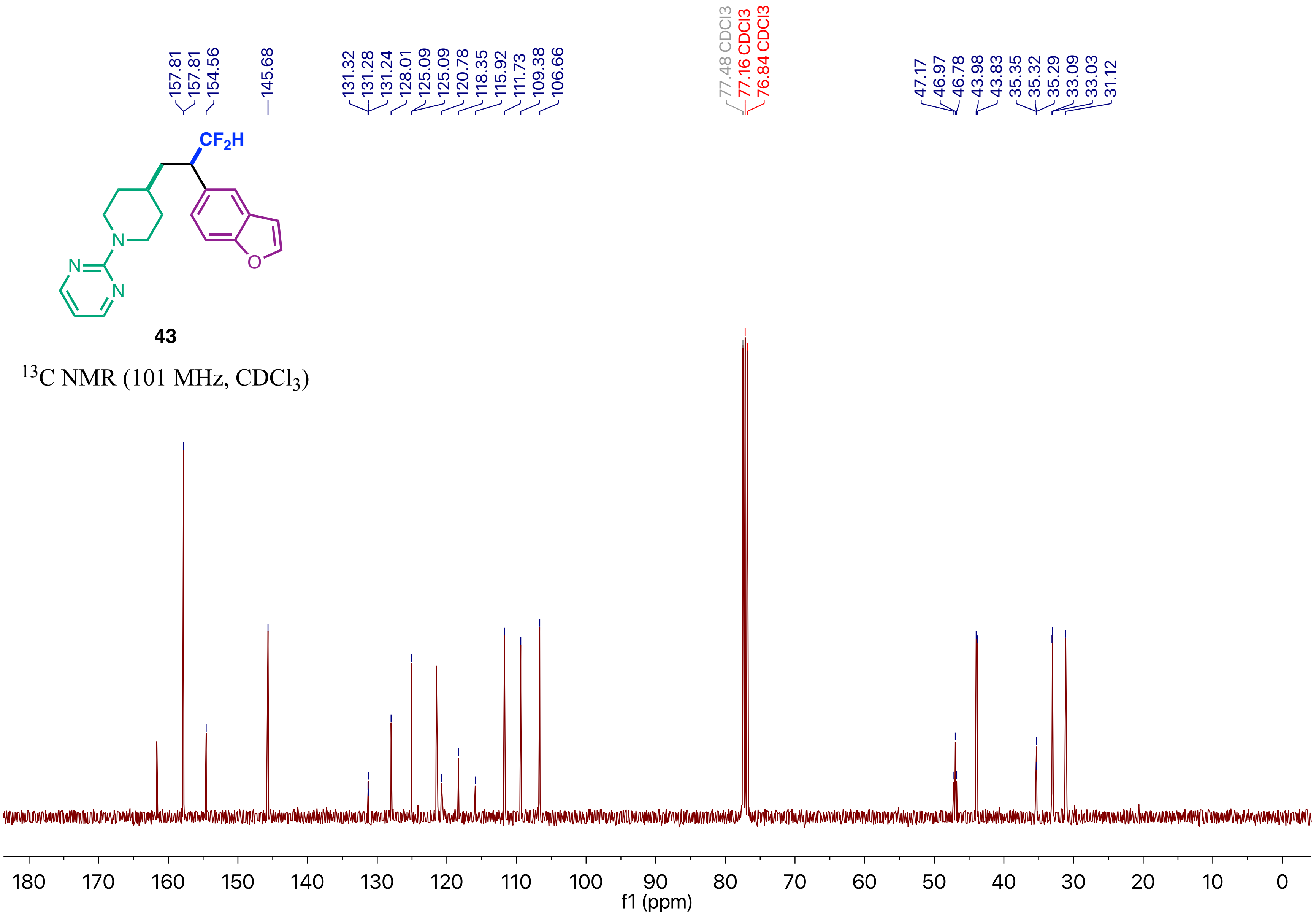
157.81
157.81
154.56
145.68
131.32
131.28
131.24
128.01
125.09
125.09
120.78
118.35
115.92
111.73
109.38
106.66
77.48 CDCl3
77.16 CDCl3
76.84 CDCl3
47.17
46.97
46.78
43.98
43.83
35.35
35.32
35.29
33.09
33.03
31.12
CF2H
N
N
N
O
43
13C NMR (101 MHz, CDCl3)
180
170
160
150
140
130
120
110
100
90
80
70
60
50
40
30
20
10
0
f1 (ppm)

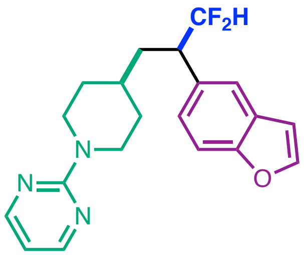

43

^{19}F NMR (376 MHz, $CDCl_3$)

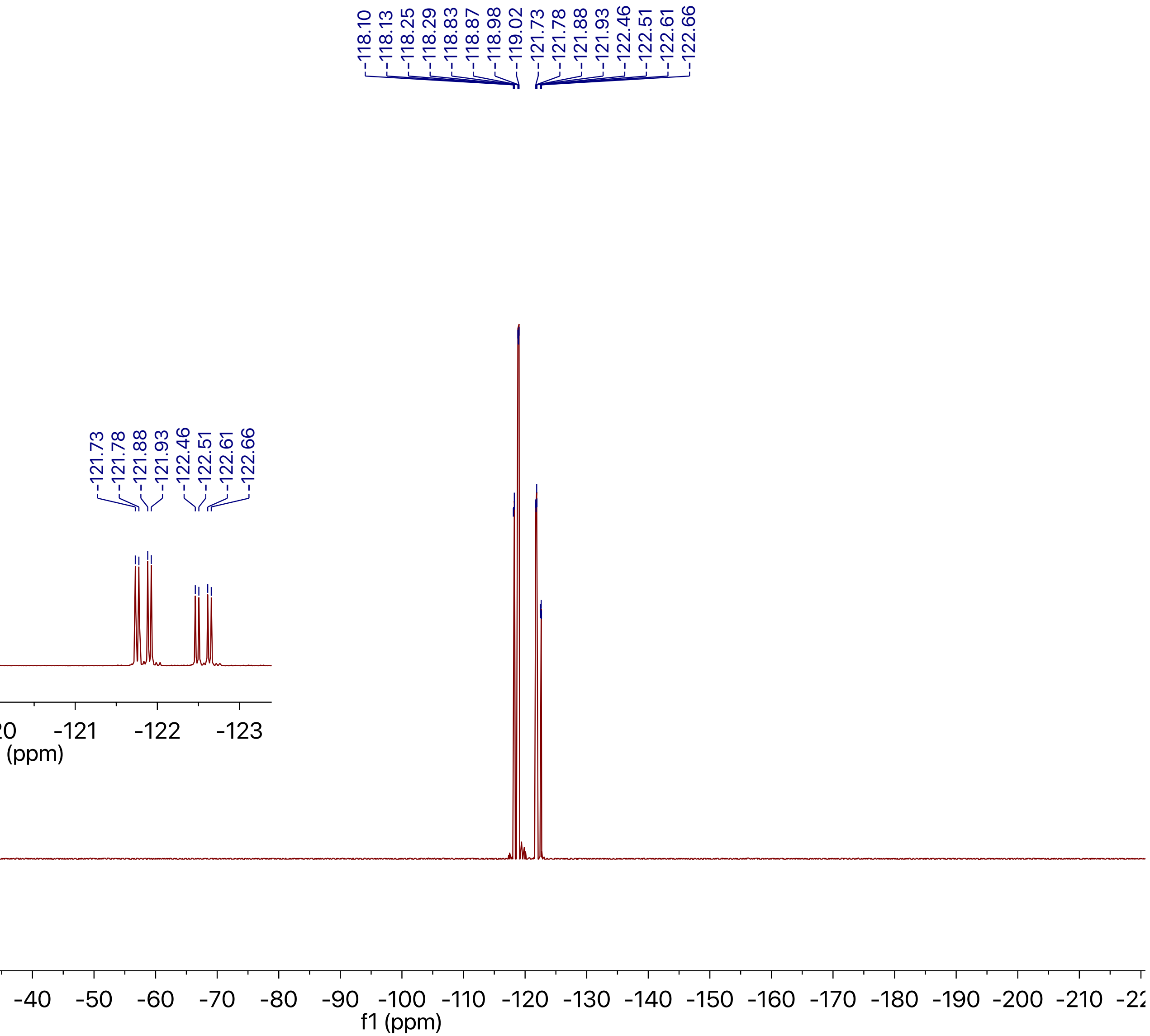

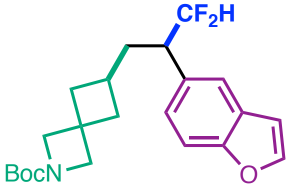

44

^{1}H NMR (400 MHz, $CDCl_3$)

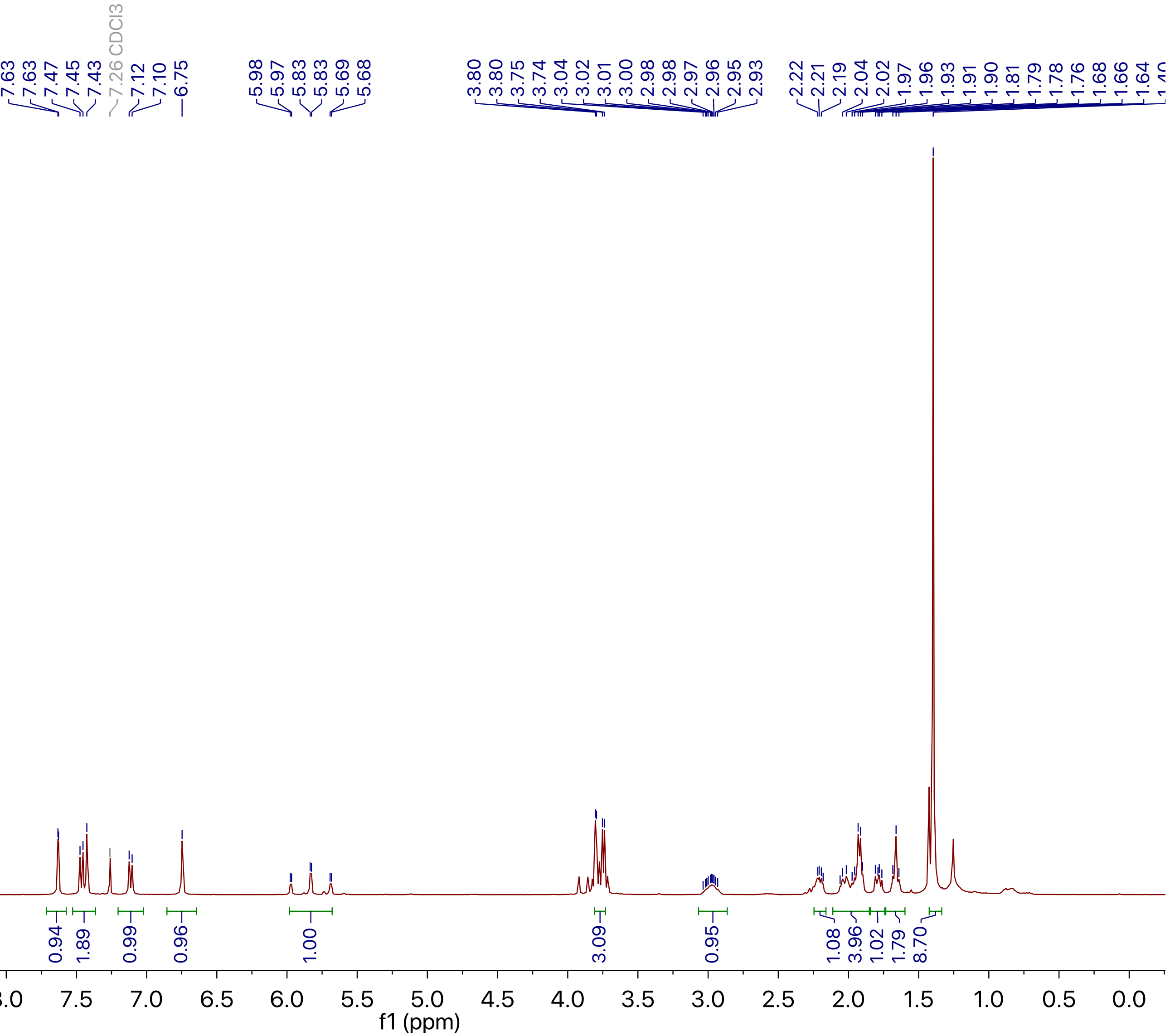

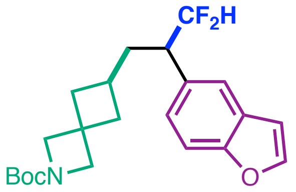

44

^{13}C NMR (101 MHz, $CDCl_3$)

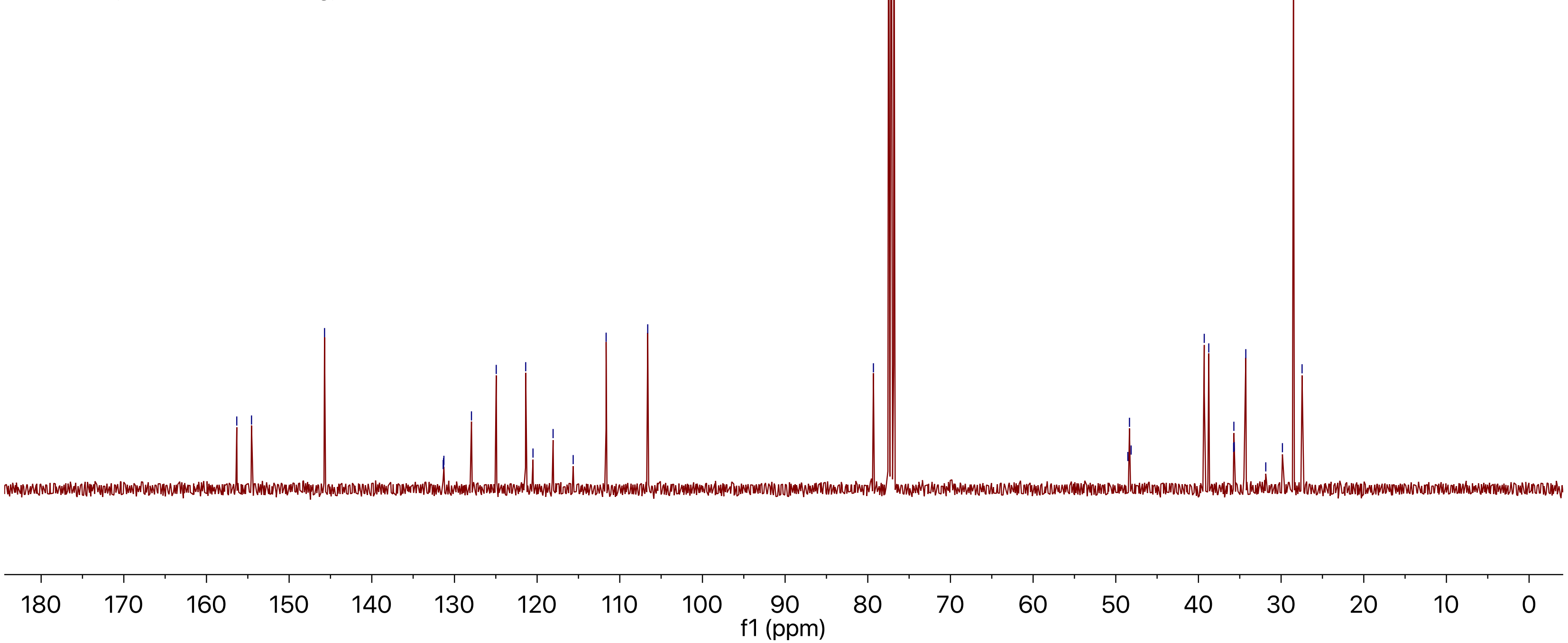

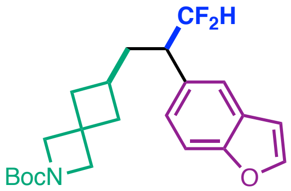

44

^{19}F NMR (376 MHz, $CDCl_3$)

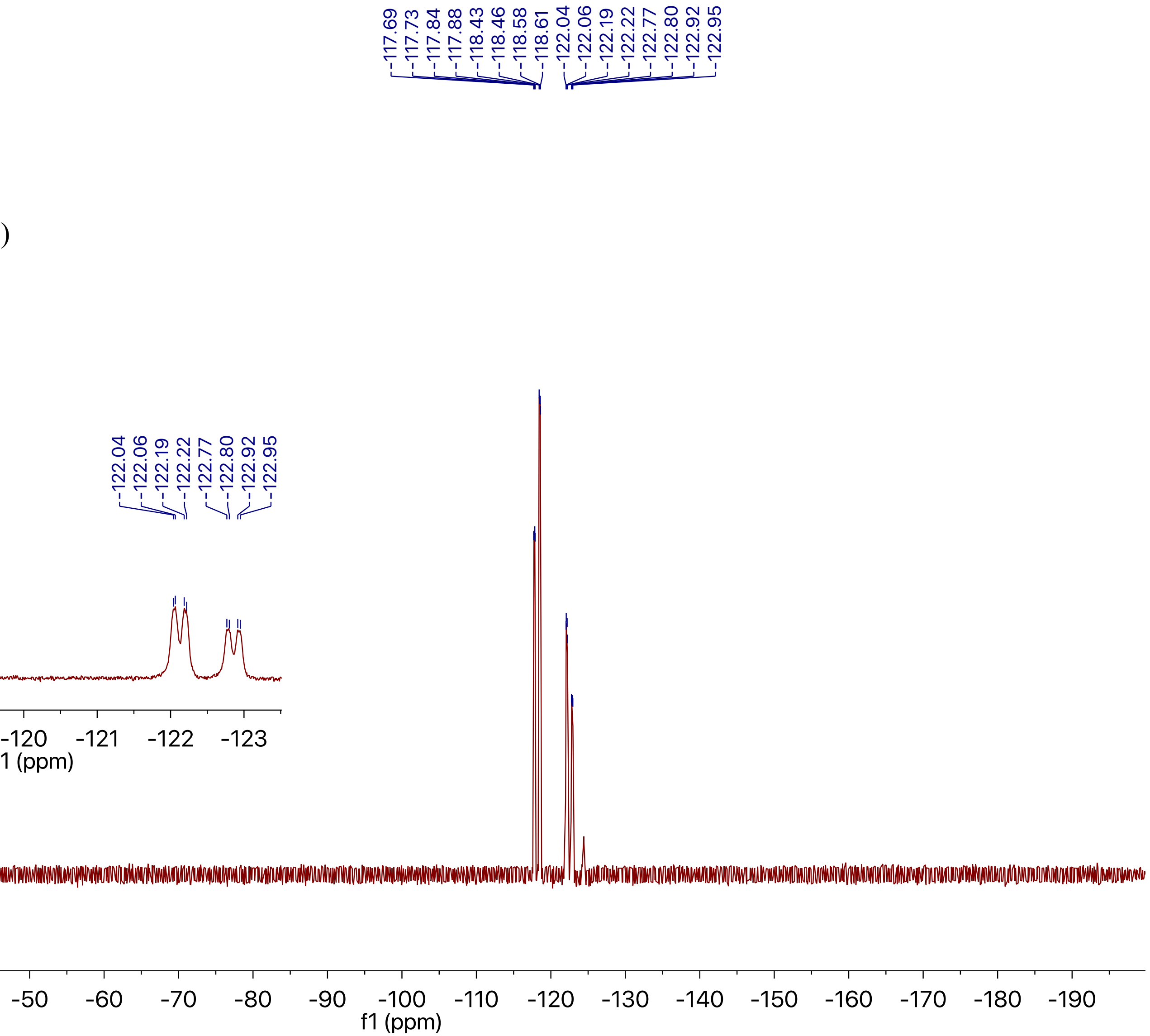

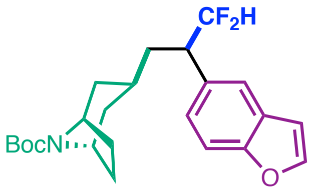

45

^{1}H NMR (400 MHz, $CDCl_3$)

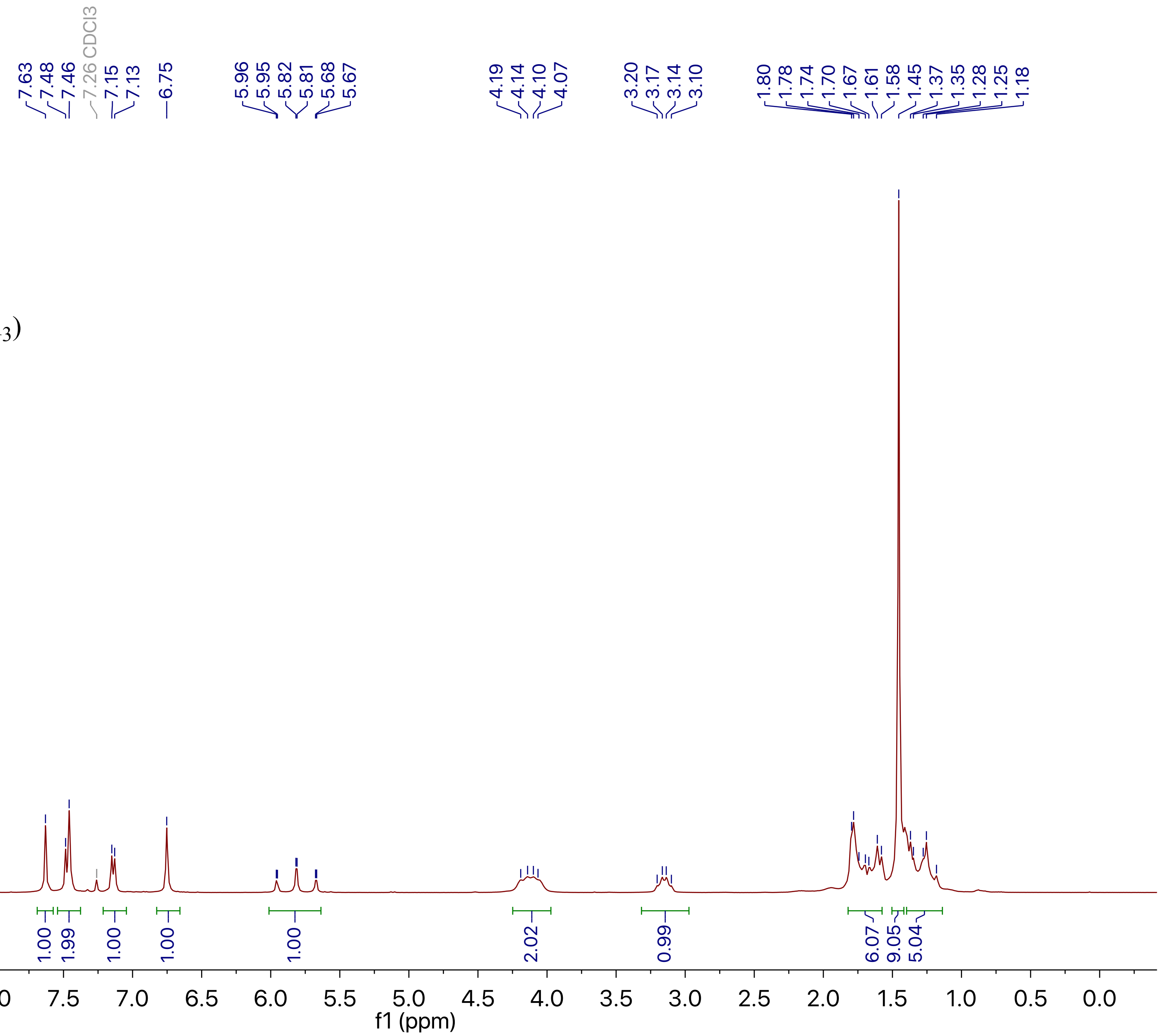

45

^{13}C NMR (101 MHz, $CDCl_3$)

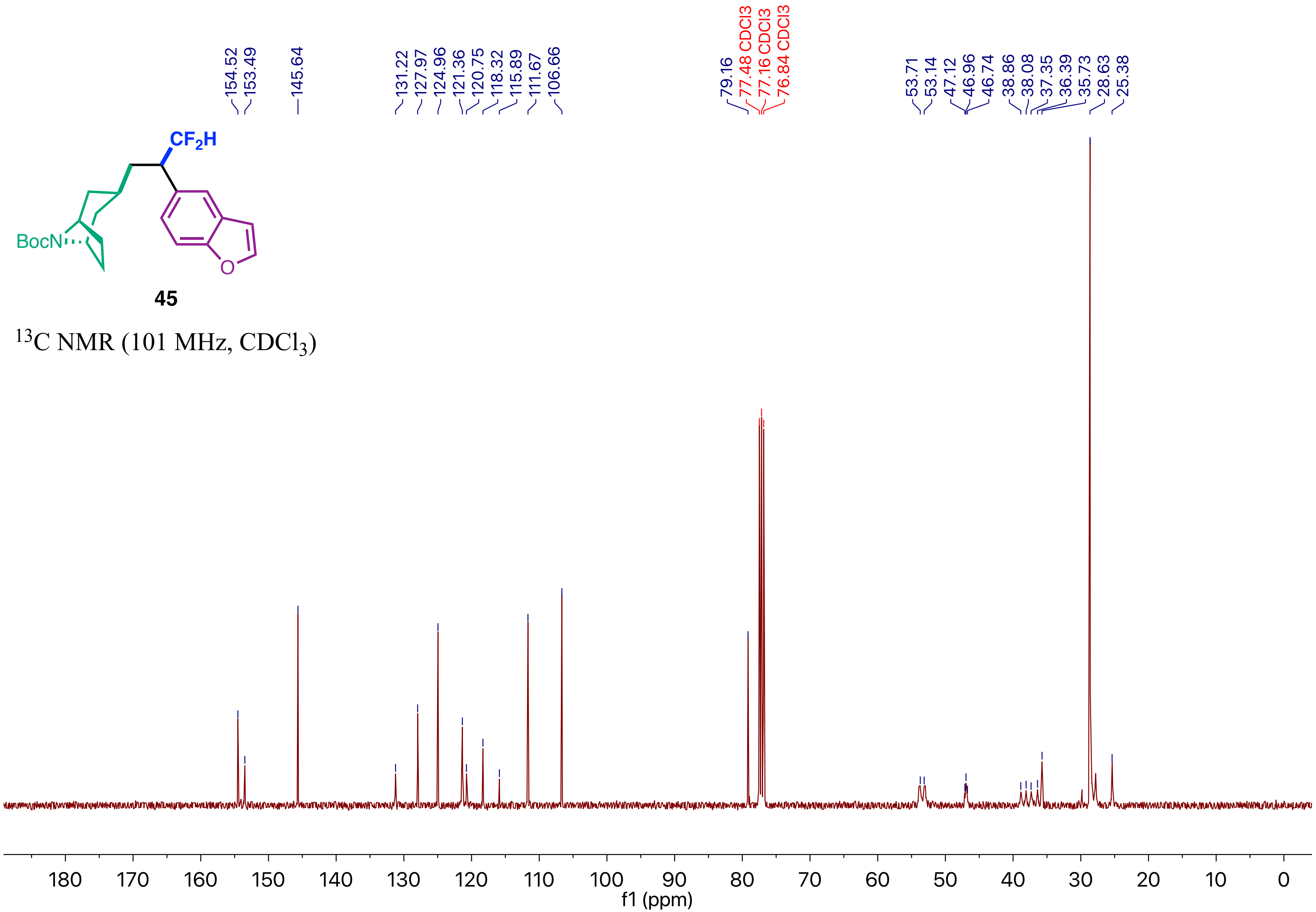

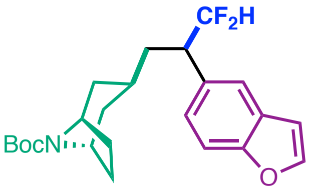

45

^{19}F NMR (376 MHz, $CDCl_3$)

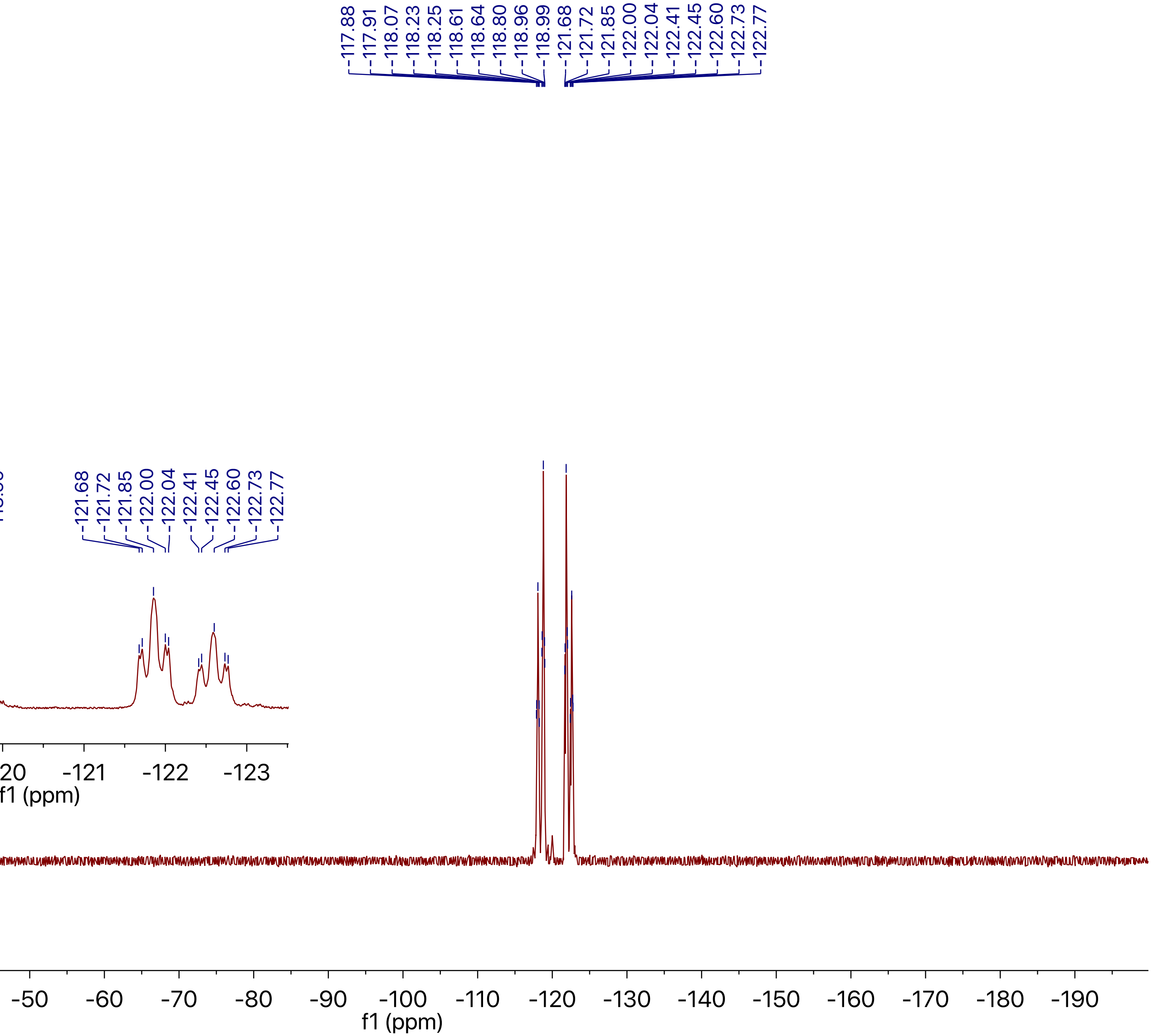

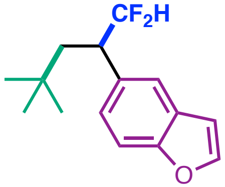

46

^{1}H NMR (400 MHz, $CDCl_3$)

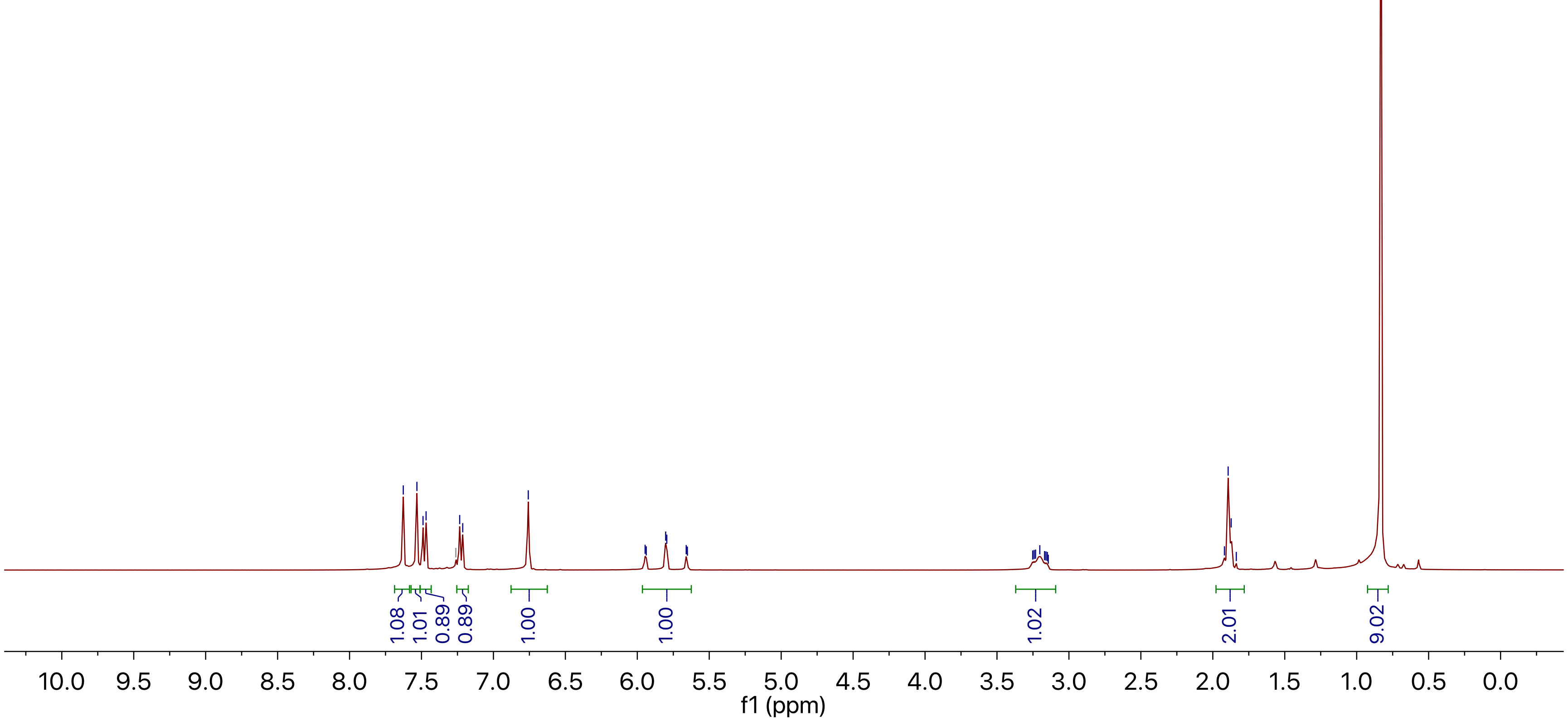

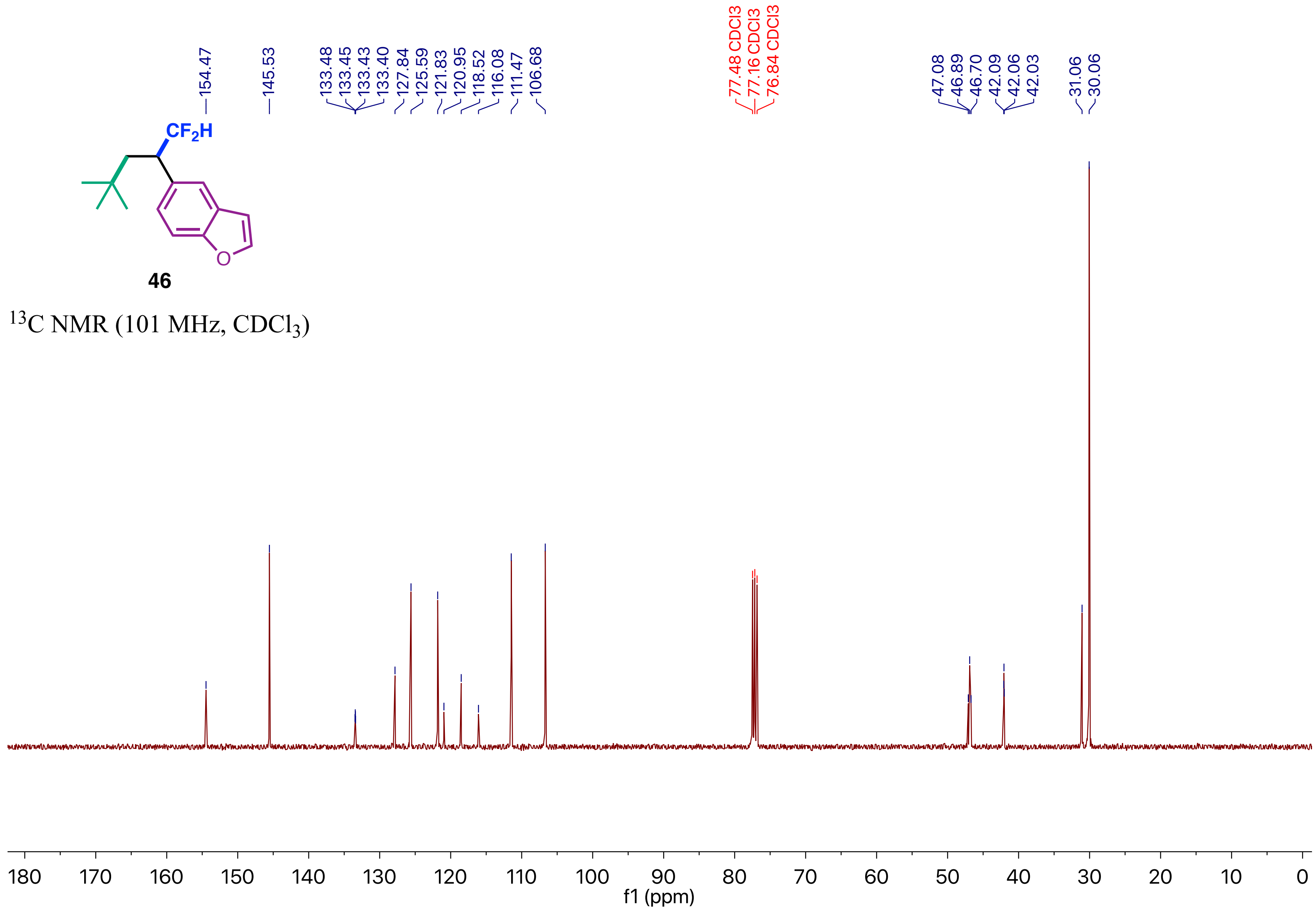

46
13C NMR (101 MHz, CDCl3)
CF2H
O
154.47
145.53
133.48
133.45
133.43
133.40
127.84
125.59
121.83
120.95
118.52
116.08
111.47
106.68
77.48 CDCl3
77.16 CDCl3
76.84 CDCl3
47.08
46.89
46.70
42.09
42.06
42.03
31.06
30.06
180 170 160 150 140 130 120 110 100 90 80 70 60 50 40 30 20 10 0
f1 (ppm)

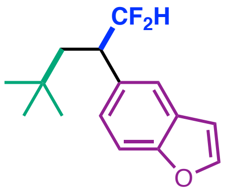

46

^{19}F NMR (376 MHz, $CDCl_3$)

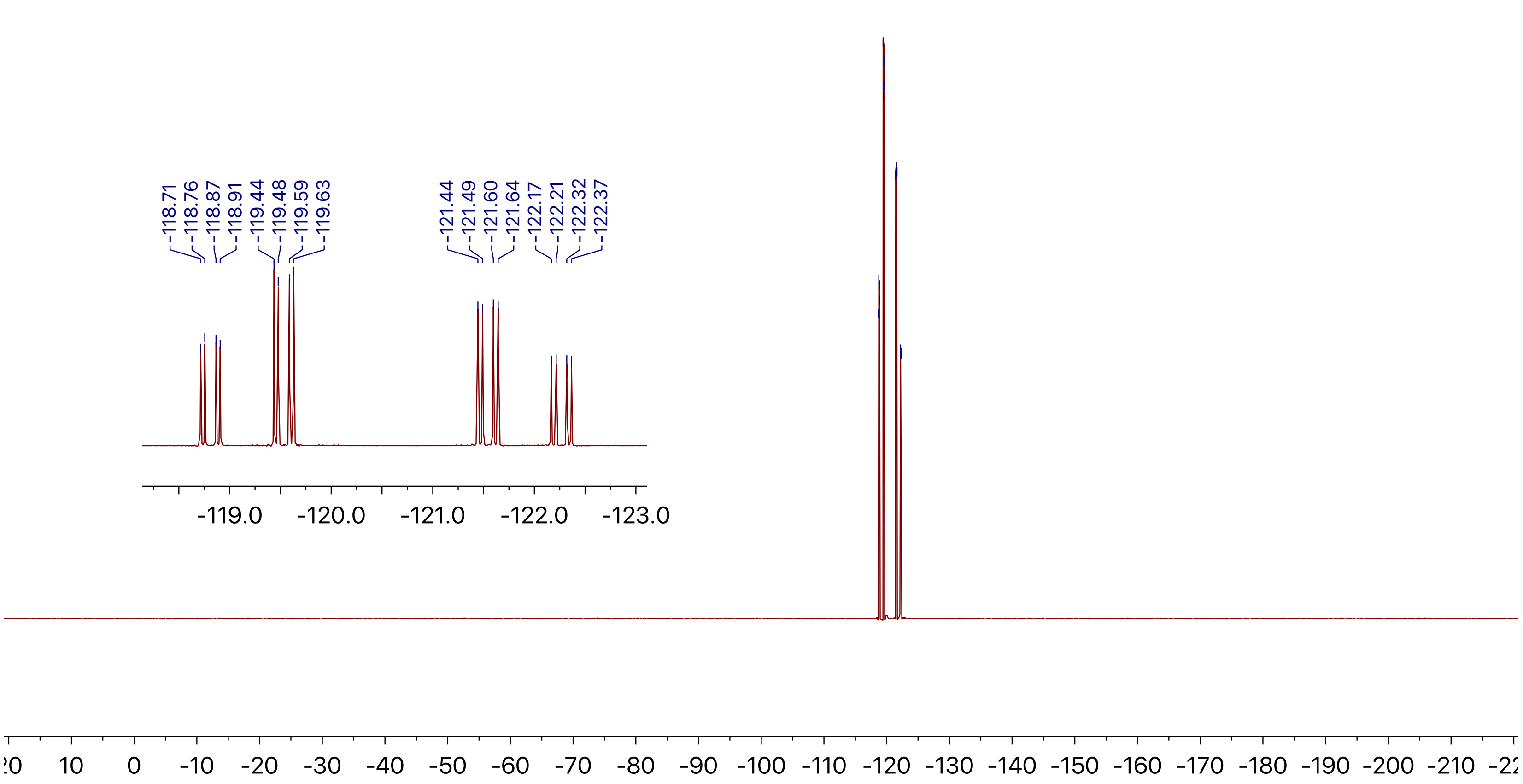

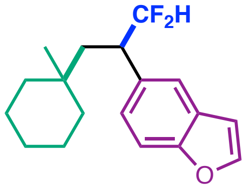

47

^1H NMR (400 MHz, $CDCl_3$)

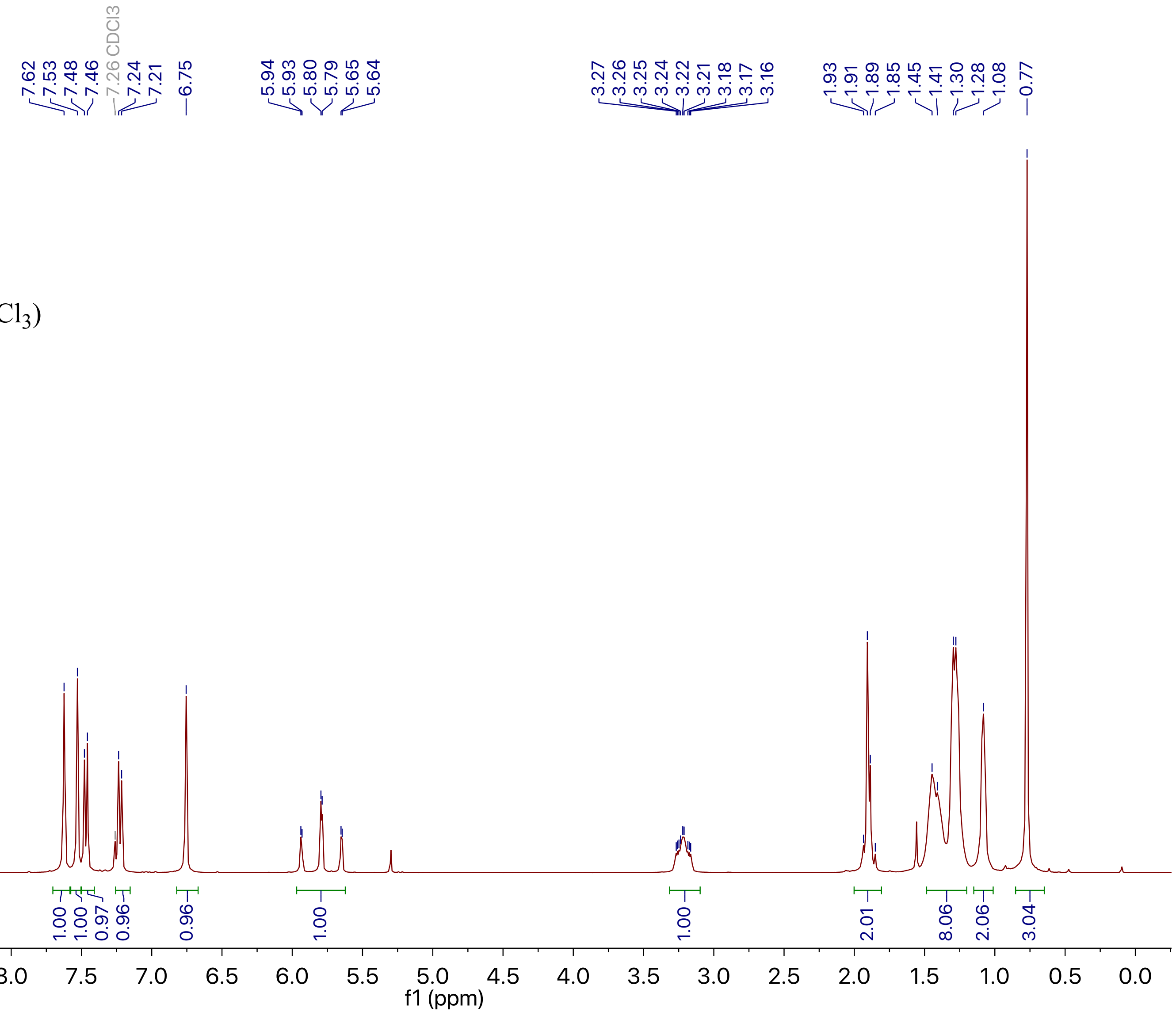

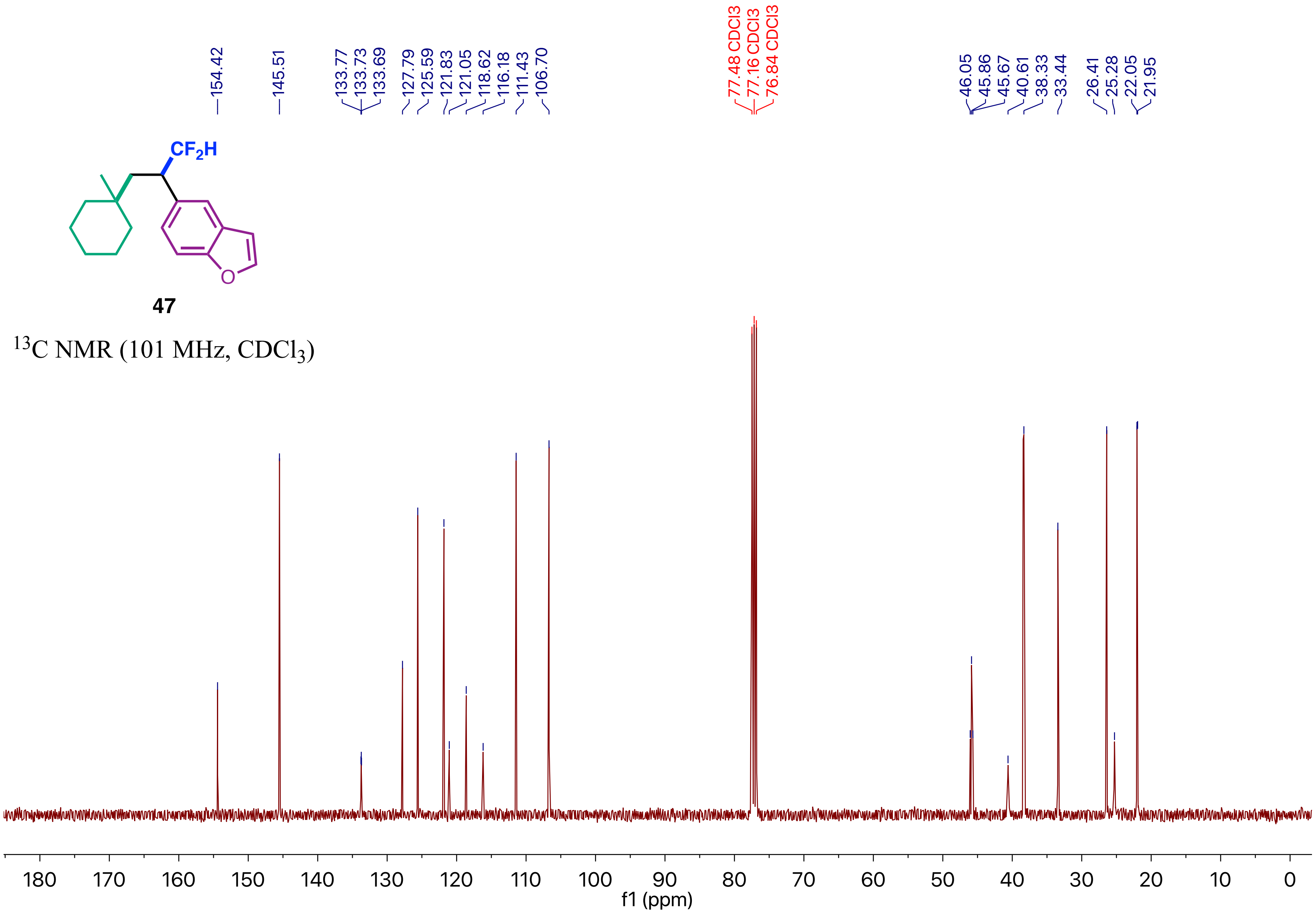
CF2H
47
13C NMR (101 MHz, CDCl3)
154.42
145.51
133.77
133.73
133.69
127.79
125.59
121.83
121.05
118.62
116.18
111.43
106.70
77.48 CDCl3
77.16 CDCl3
76.84 CDCl3
46.05
45.86
45.67
40.61
38.33
33.44
26.41
25.28
22.05
21.95
180 170 160 150 140 130 120 110 100 90 80 70 60 50 40 30 20 10 0
f1 (ppm)

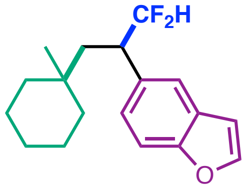

47

^{19}F NMR (376 MHz, $CDCl_3$)

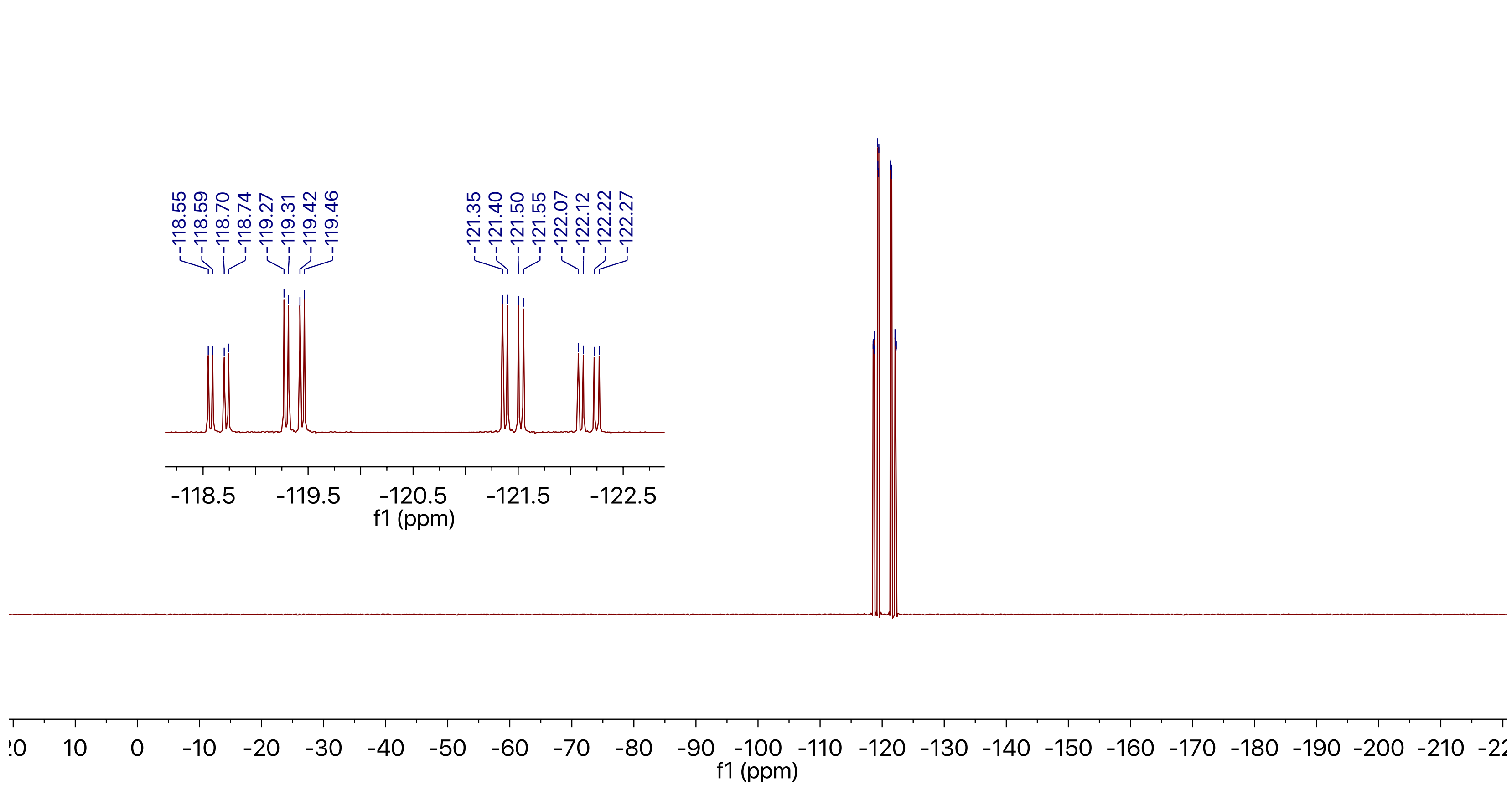

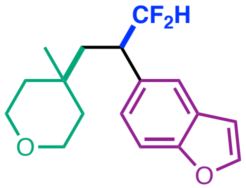

48

^1H NMR (400 MHz, $CDCl_3$)

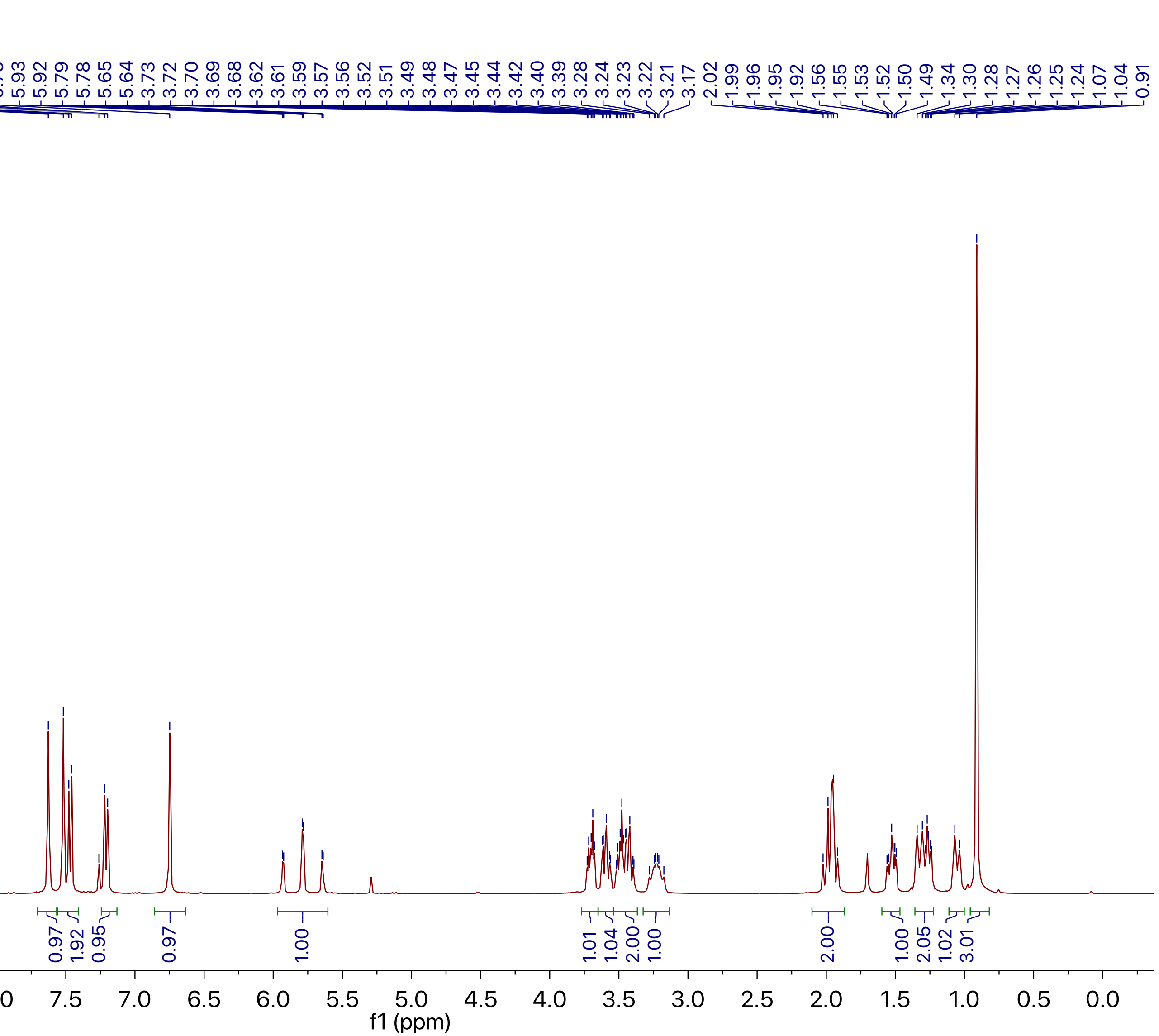

48

^{13}C NMR (101 MHz, $CDCl_3$)

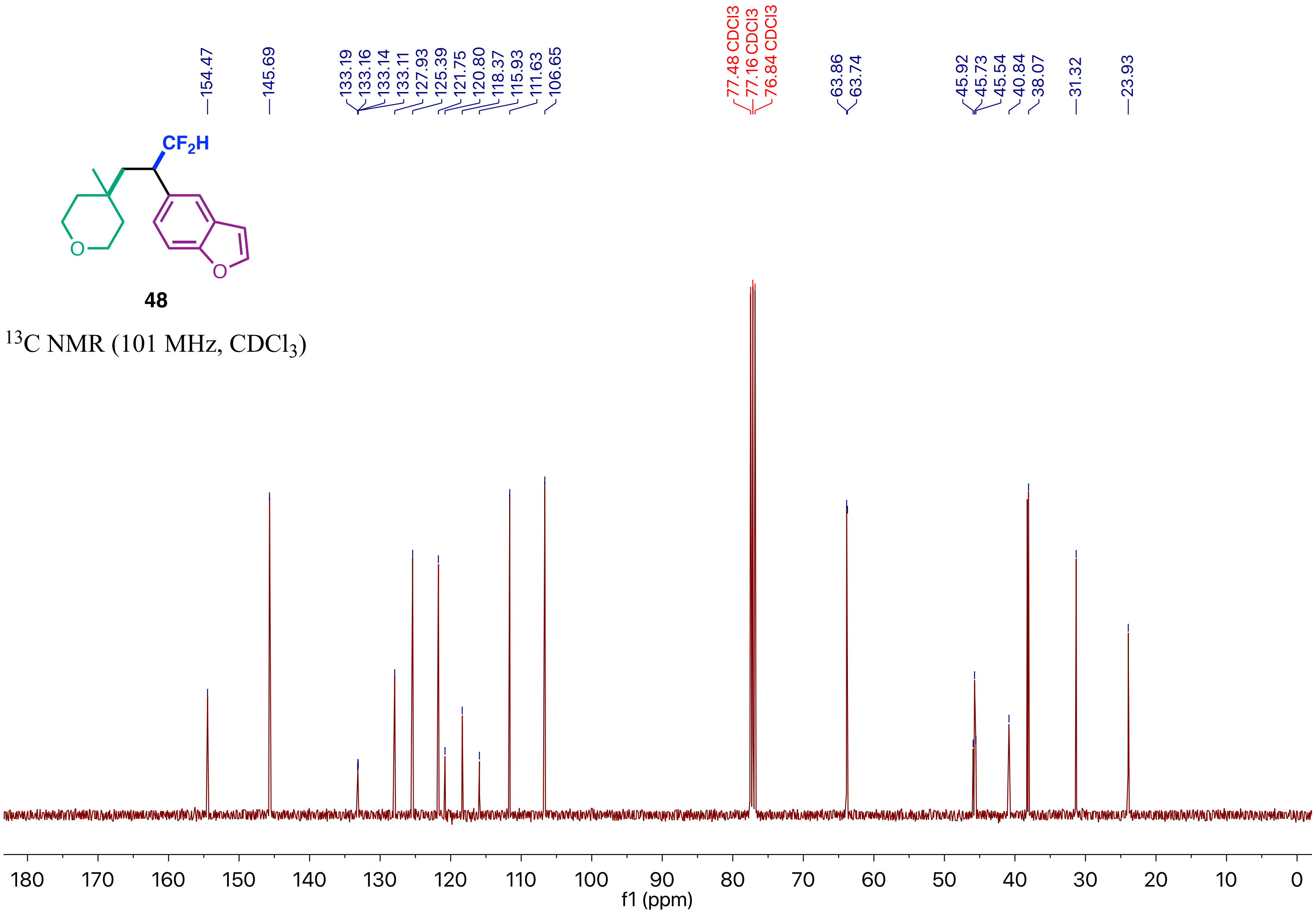

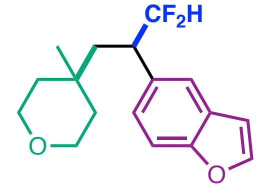

48

^{19}F NMR (376 MHz, $CDCl_3$)

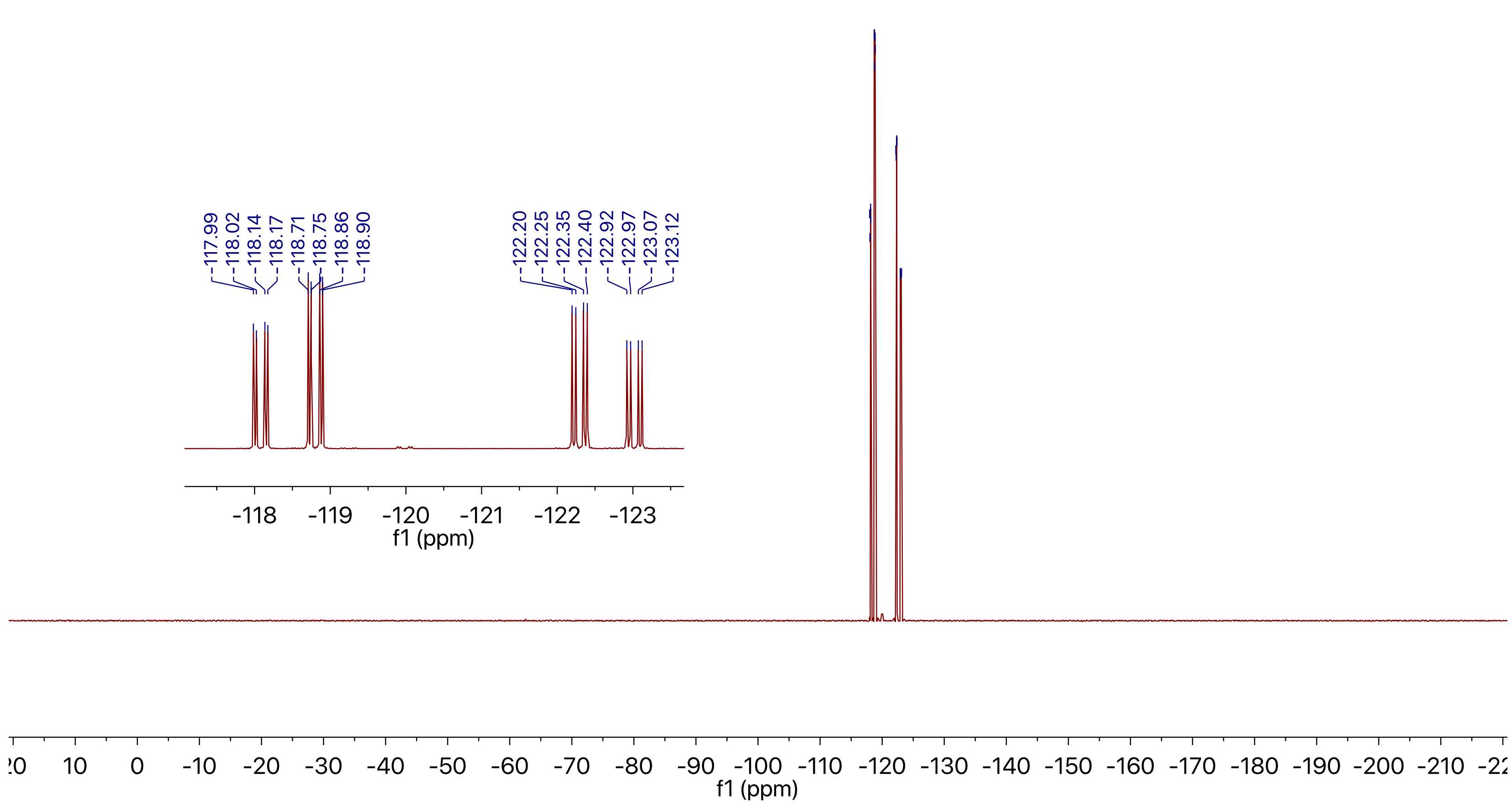

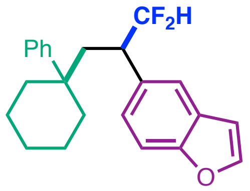

49

^{1}H NMR (400 MHz, $CDCl_3$)

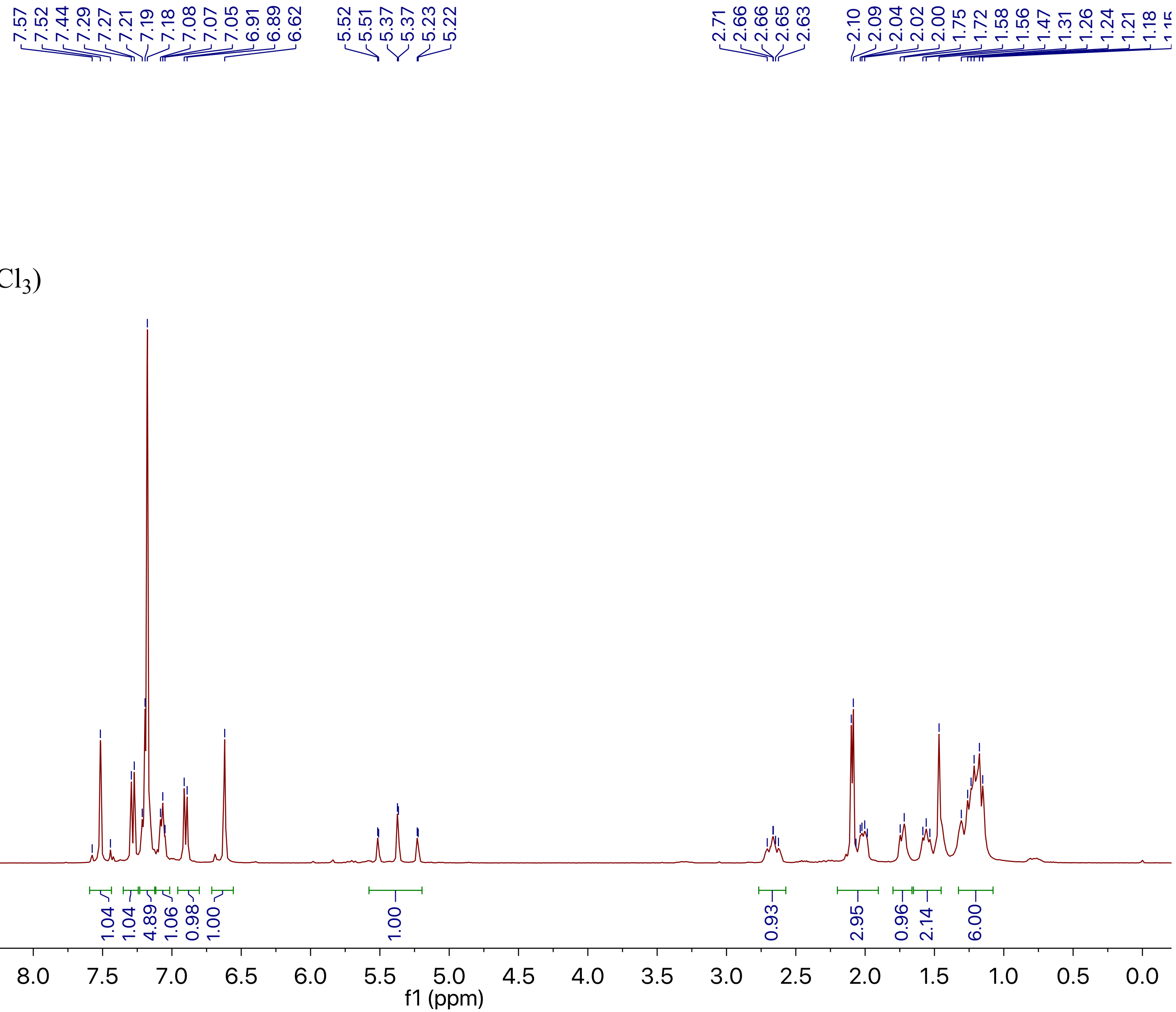

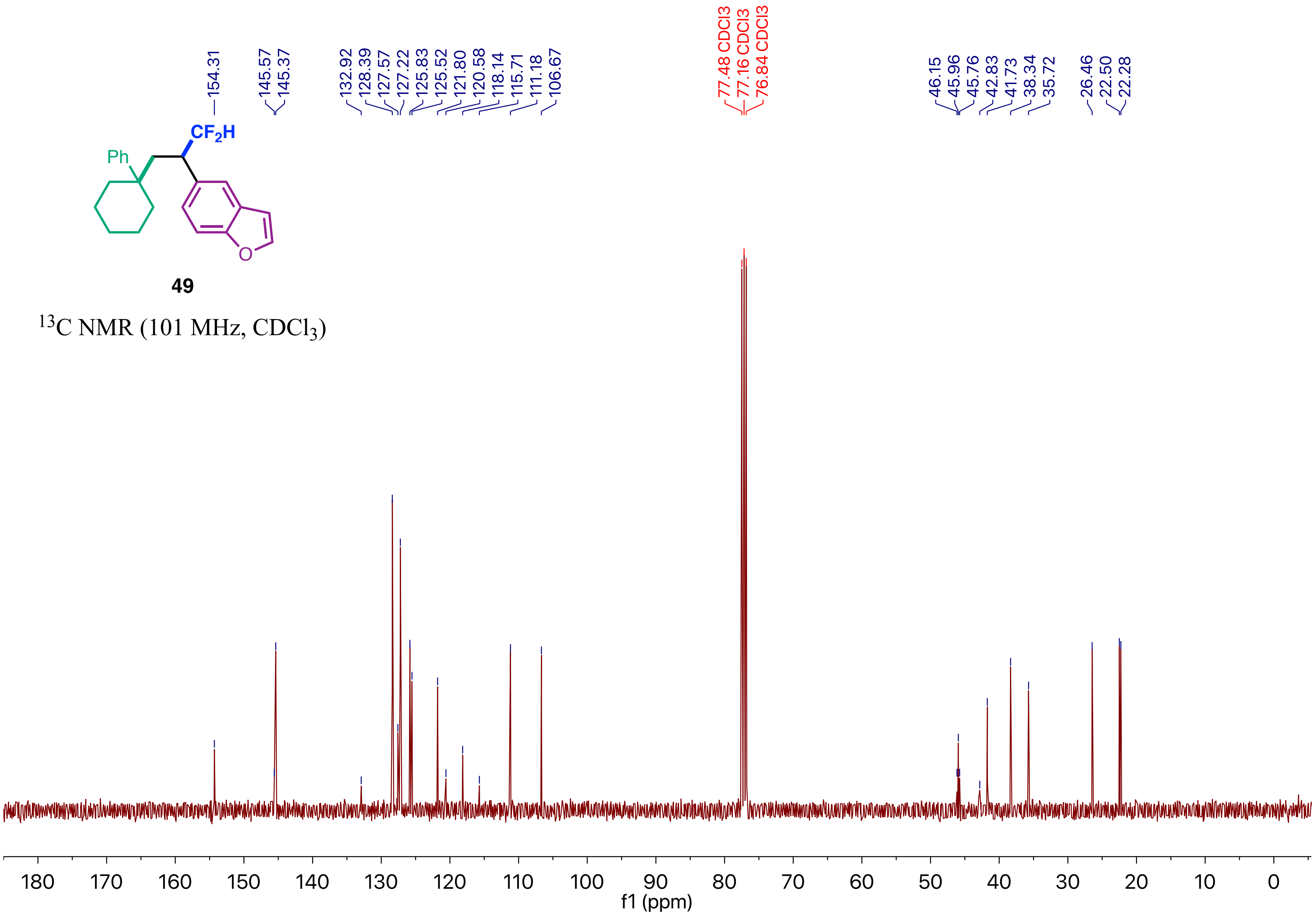
154.31
145.57
145.37
132.92
128.39
127.57
127.22
125.83
125.52
121.80
120.58
118.14
115.71
111.18
106.67
77.48 CDCl3
77.16 CDCl3
76.84 CDCl3
46.15
45.96
45.76
42.83
41.73
38.34
35.72
26.46
22.50
22.28
CF2H
Ph
O
49
13C NMR (101 MHz, CDCl3)
180 170 160 150 140 130 120 110 100 90 80 70 60 50 40 30 20 10 0
f1 (ppm)

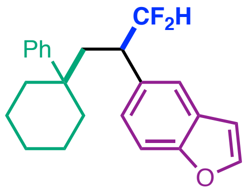

49

^{19}F NMR (376 MHz, $CDCl_3$)

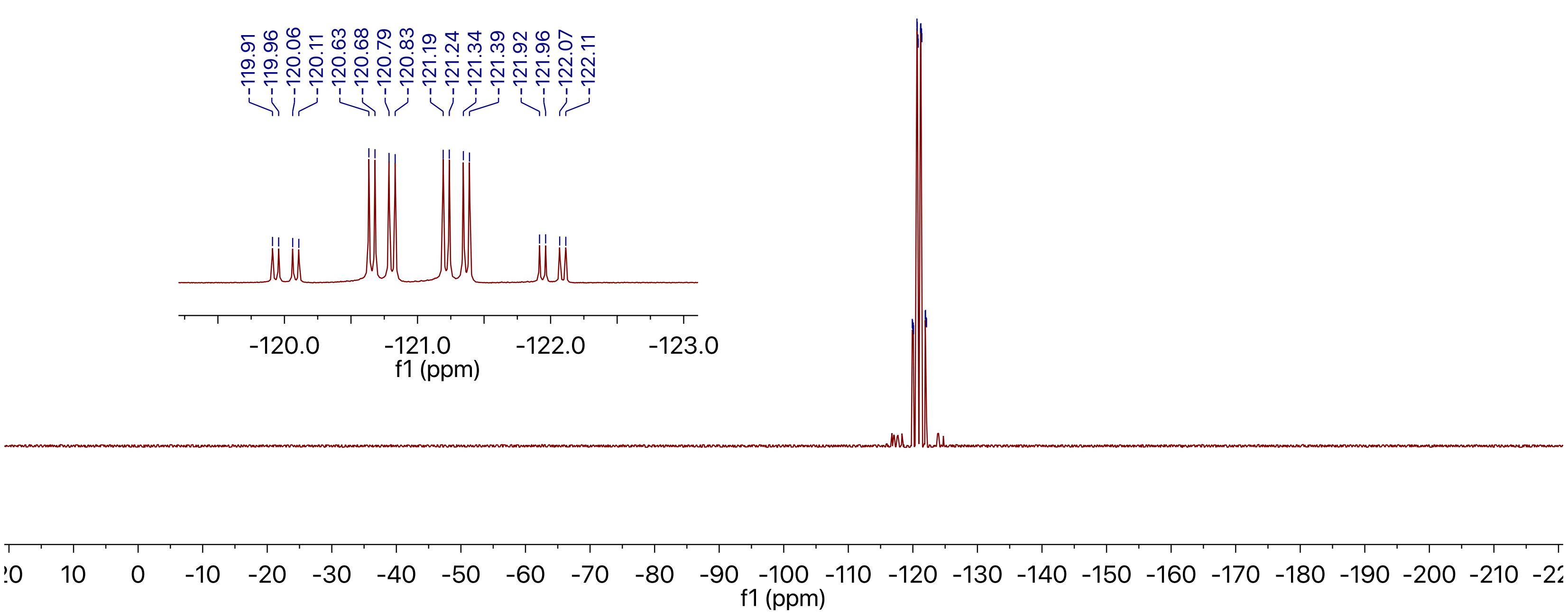

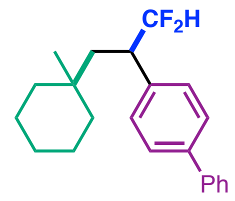

50

^{1}H NMR (400 MHz, $CDCl_3$)

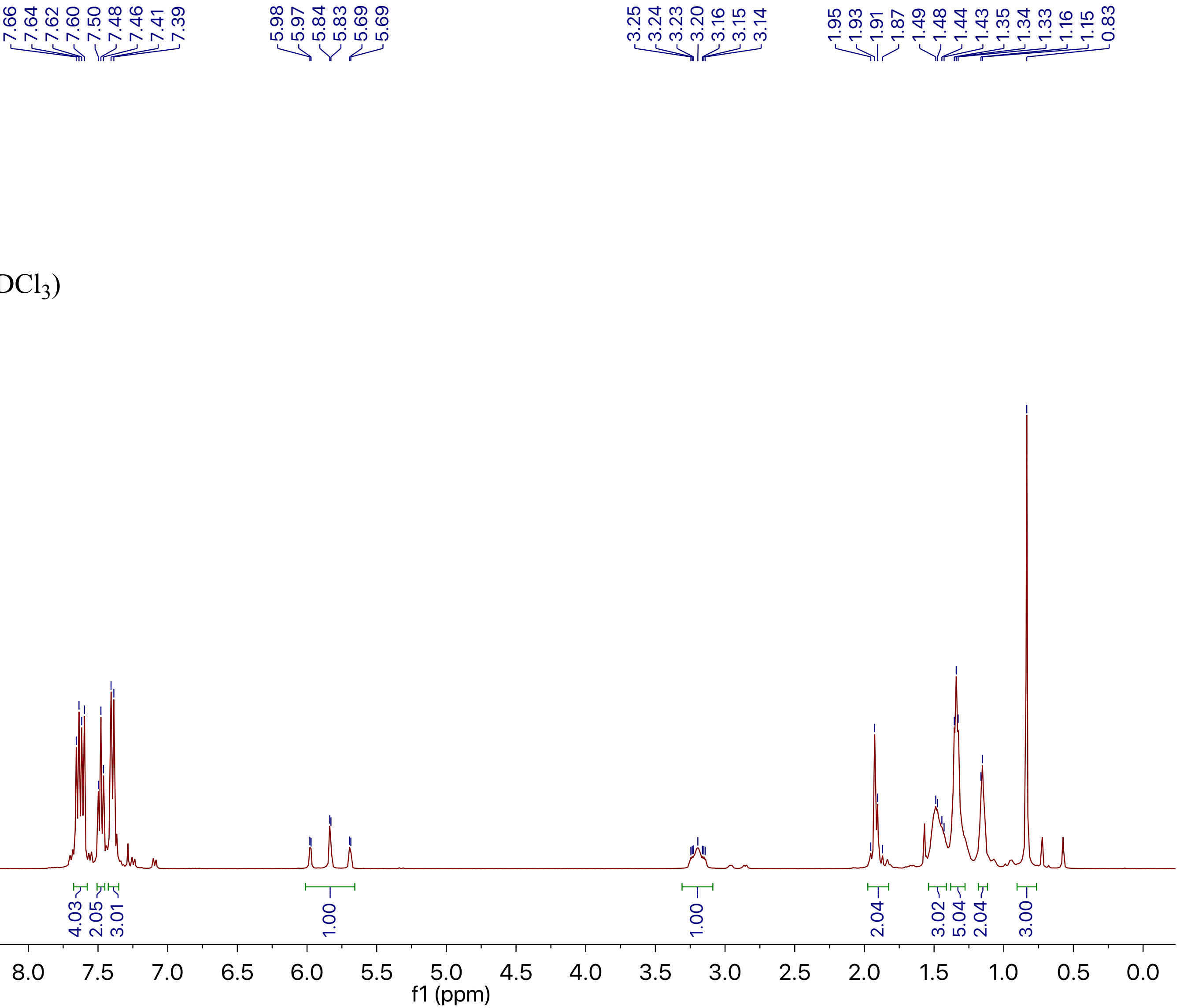

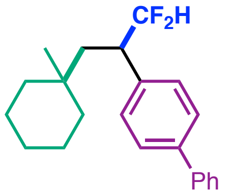

50

^{13}C NMR (101 MHz, $CDCl_3$)

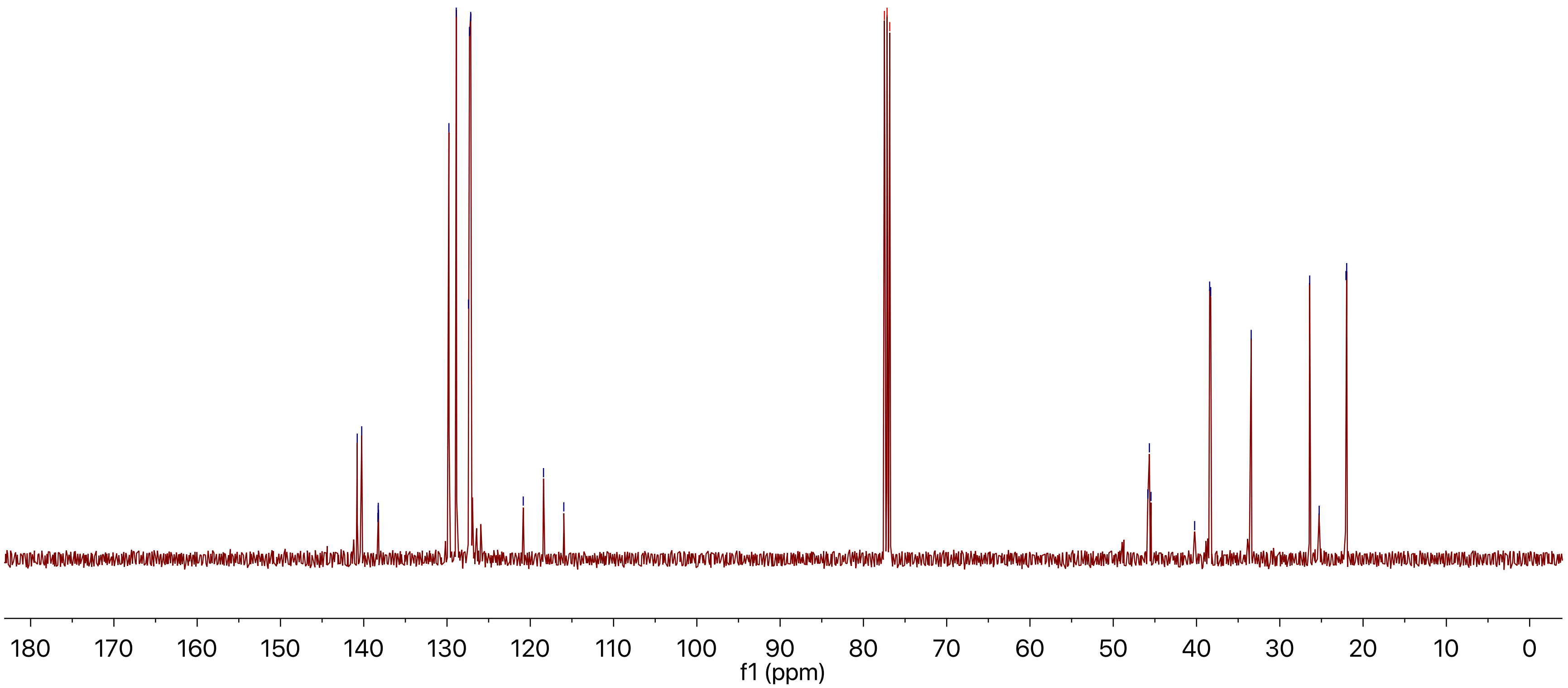

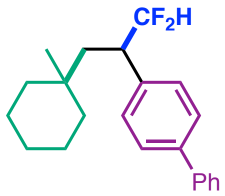

50

^{19}F NMR (376 MHz, $CDCl_3$)

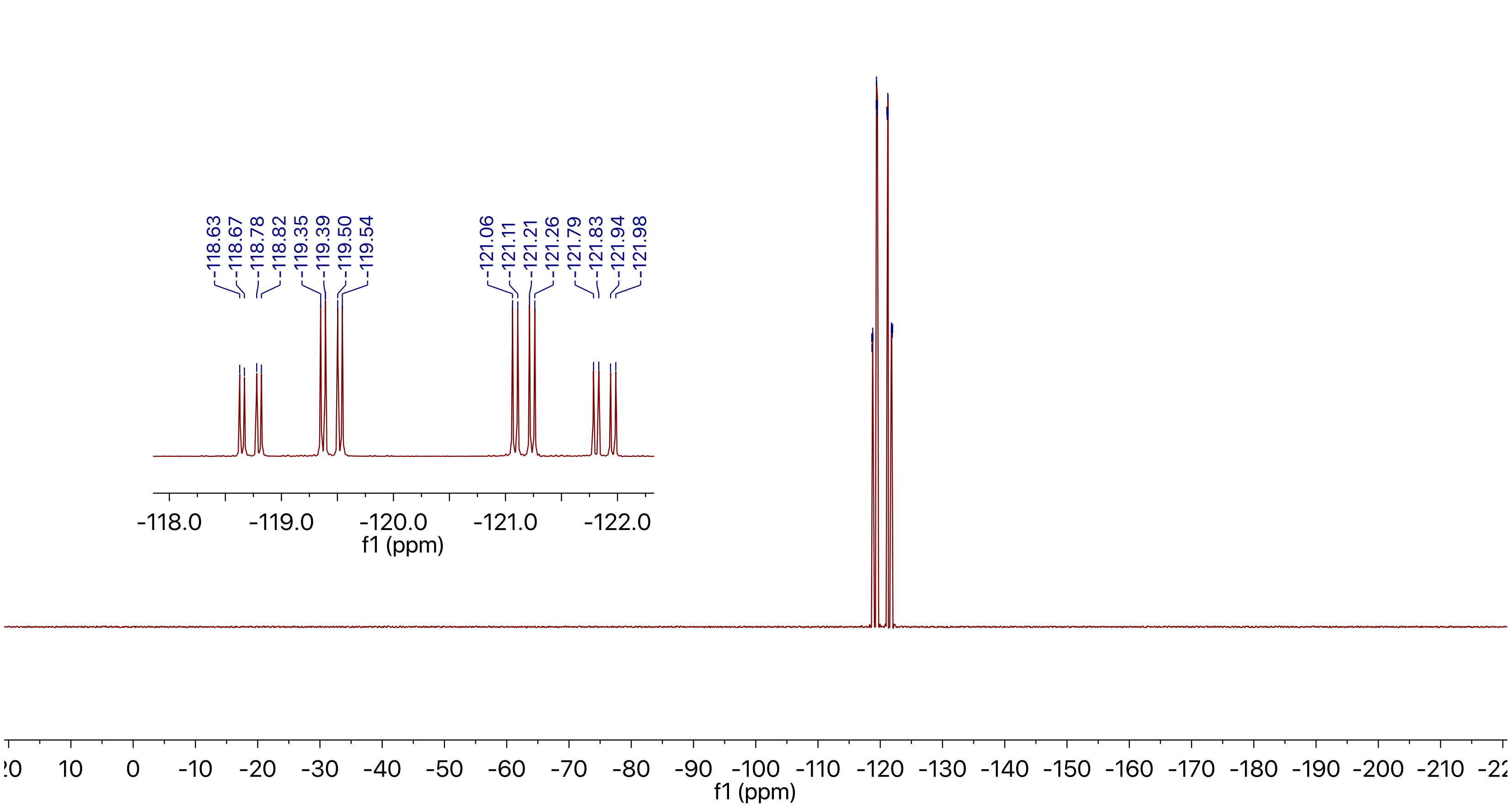

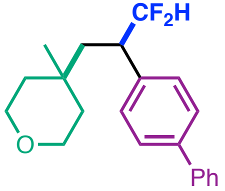

51

^{1}H NMR (400 MHz, $CDCl_3$)

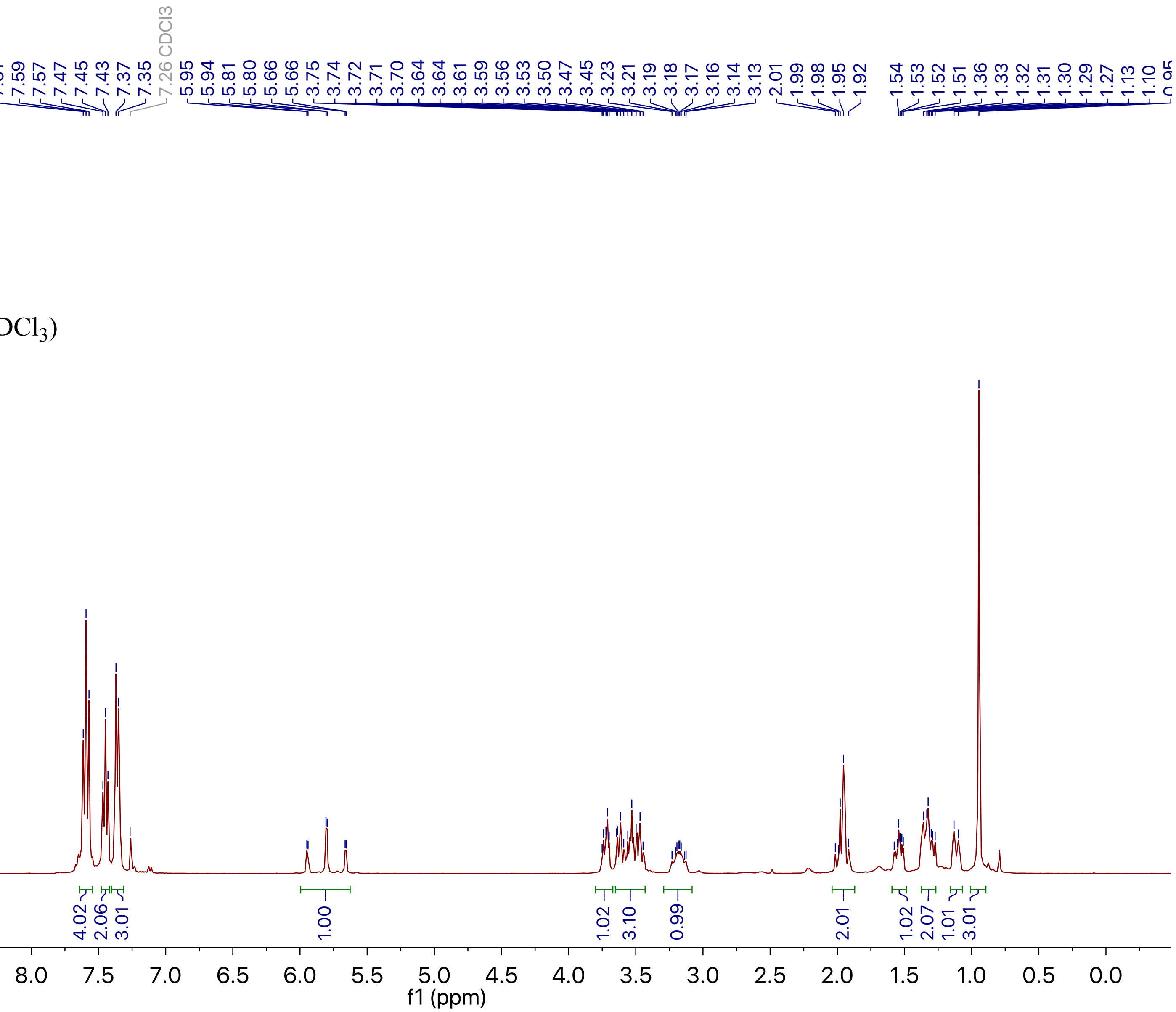

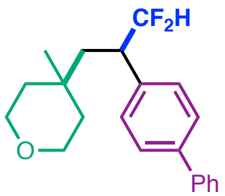

51

^{13}C NMR (101 MHz, $CDCl_3$)

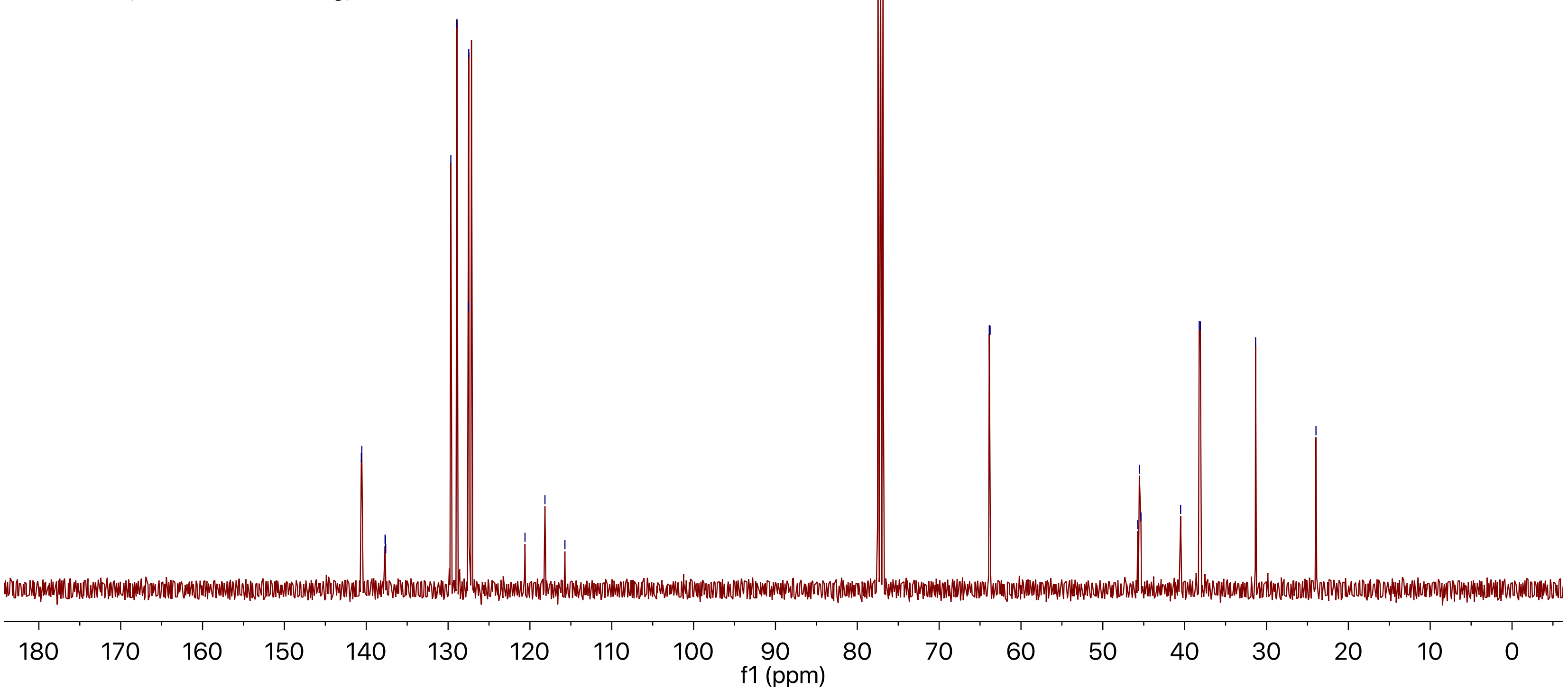

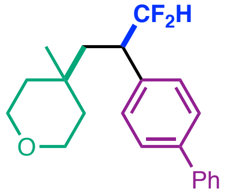

51

^{19}F NMR (376 MHz, $CDCl_3$)

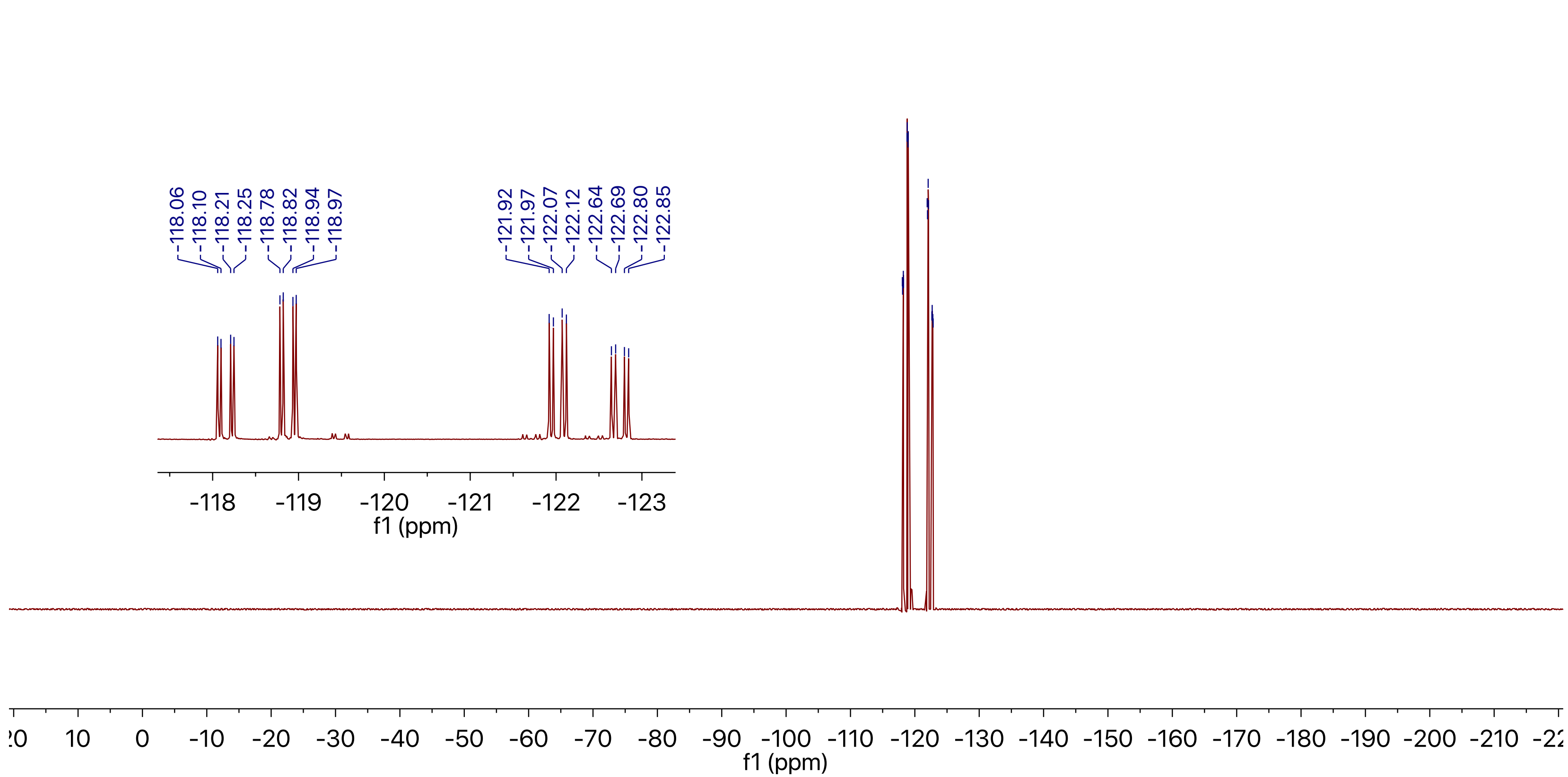

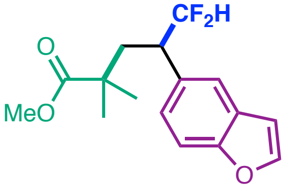

52

^{1}H NMR (400 MHz, $CDCl_3$)

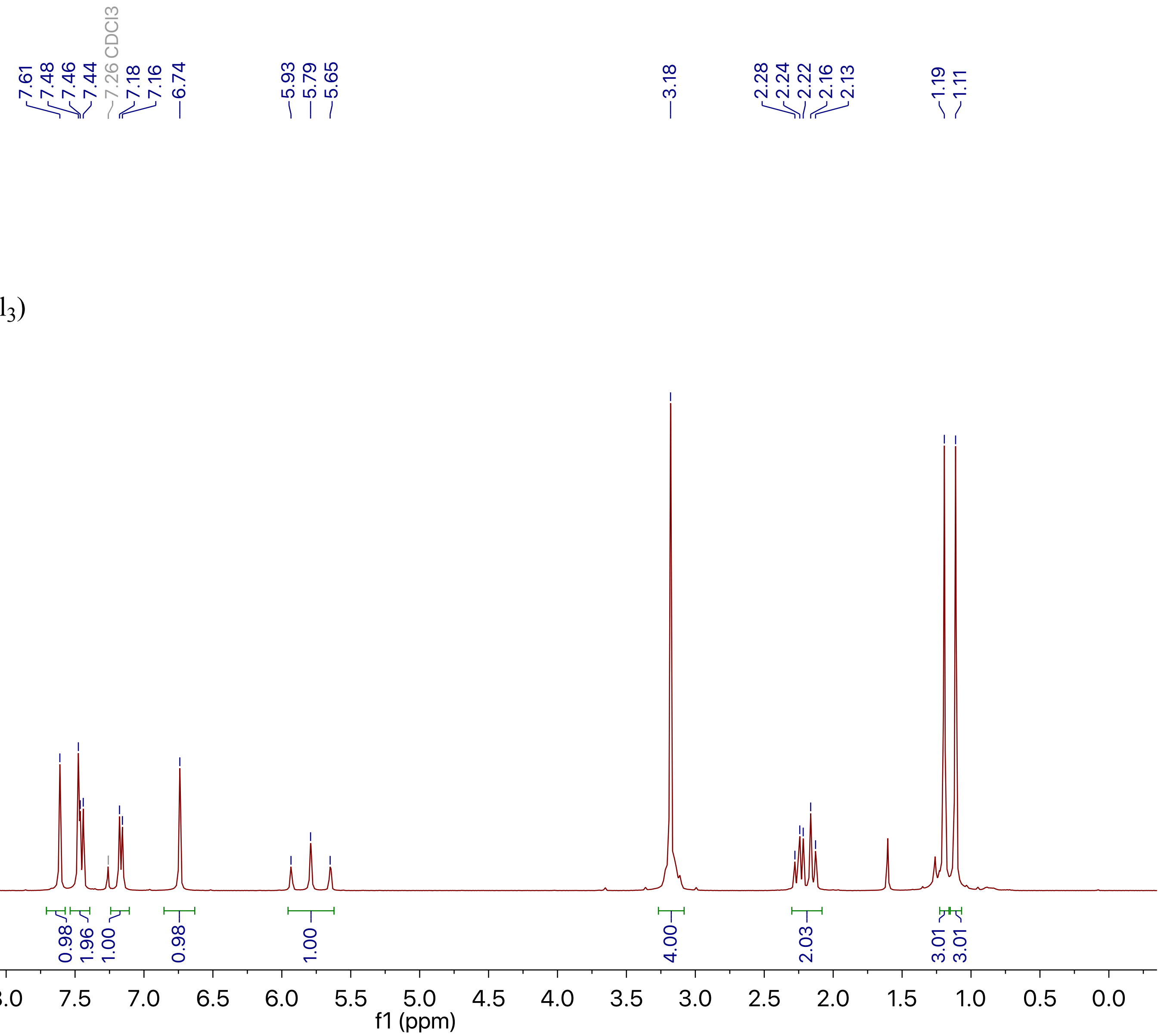

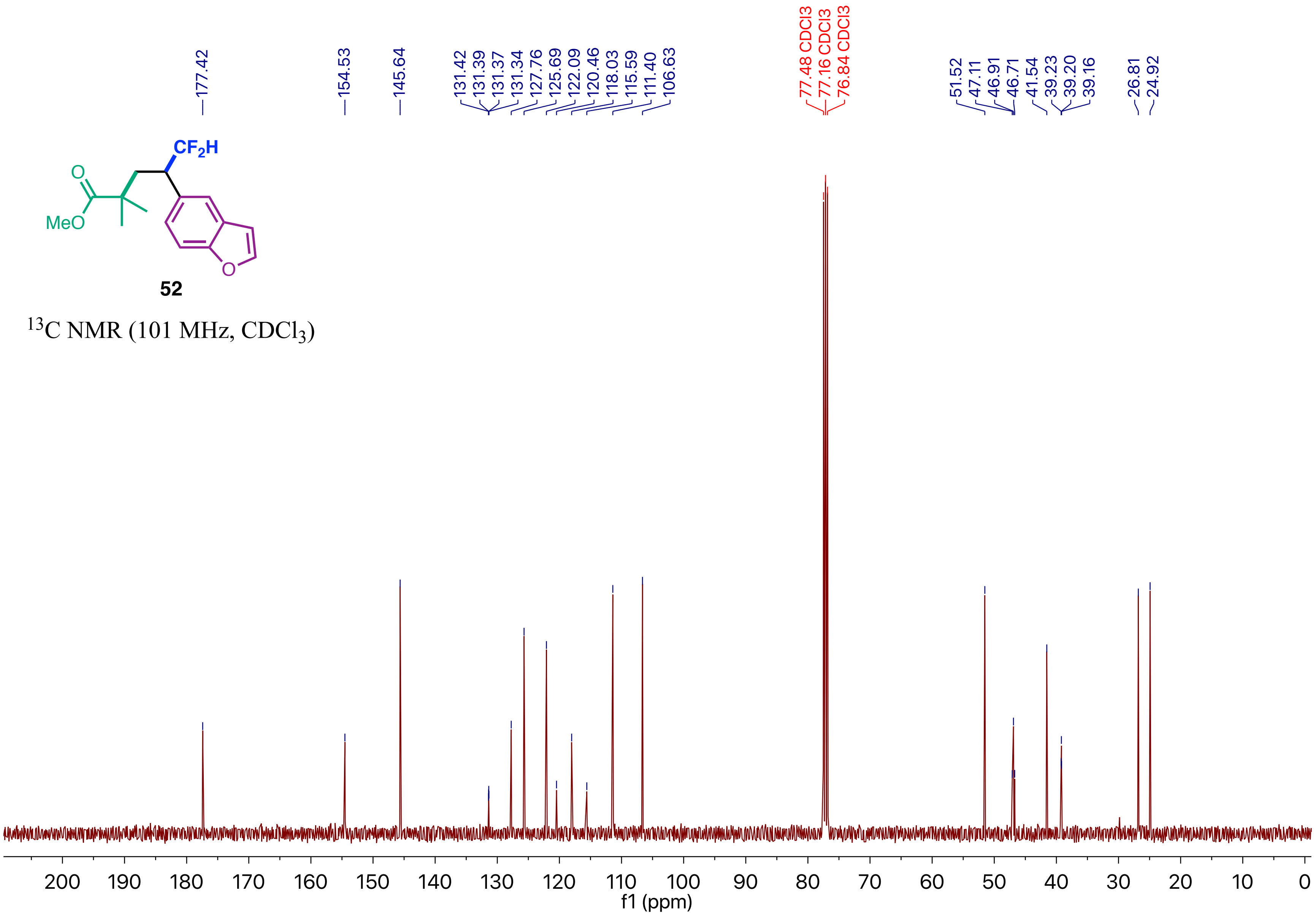

^{13}C NMR (101 MHz, $CDCl_3$)

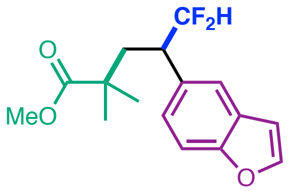

52

^{19}F NMR (376 MHz, $CDCl_3$)

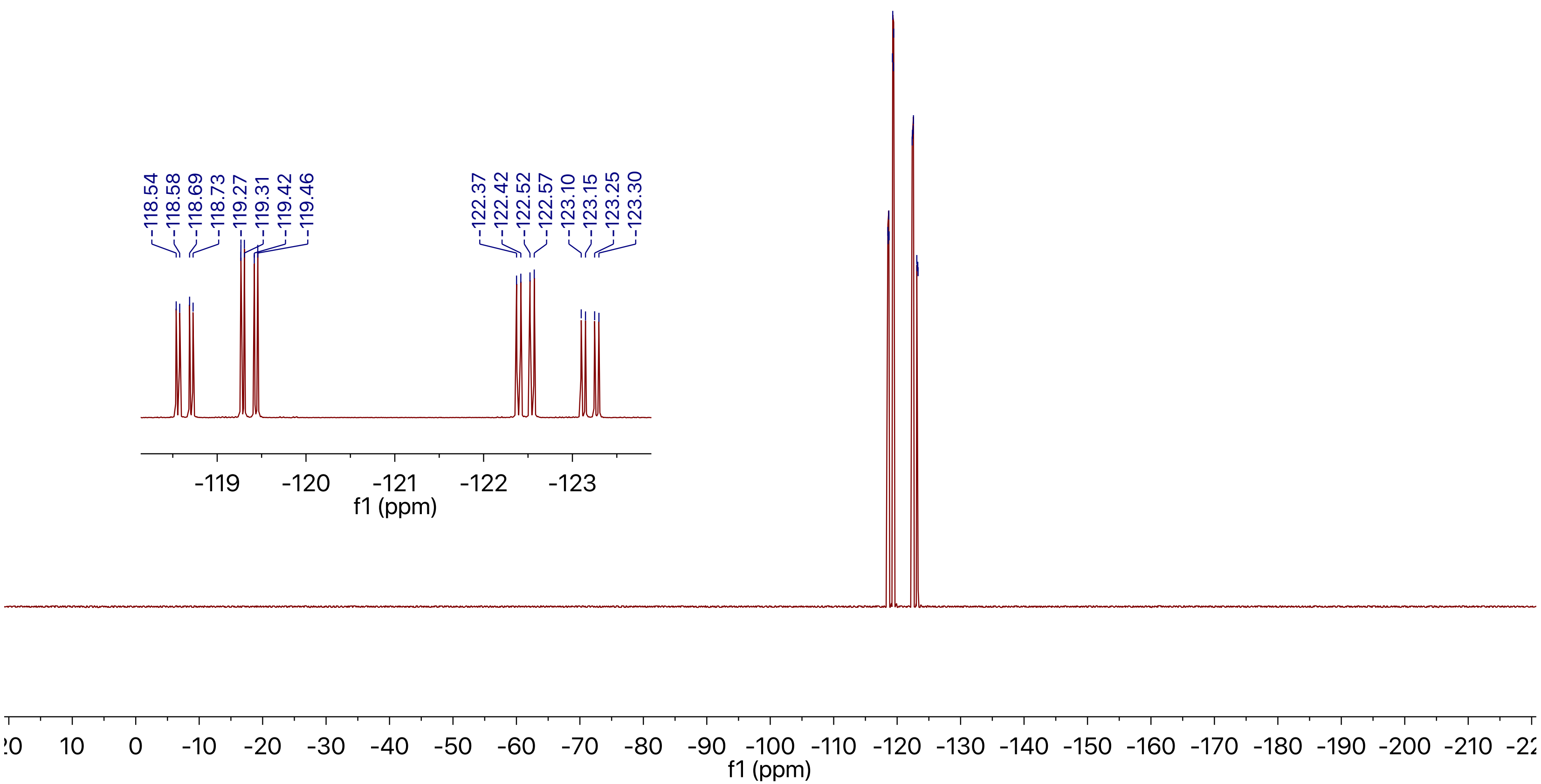

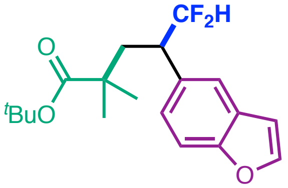

53

^{1}H NMR (400 MHz, $CDCl_3$)

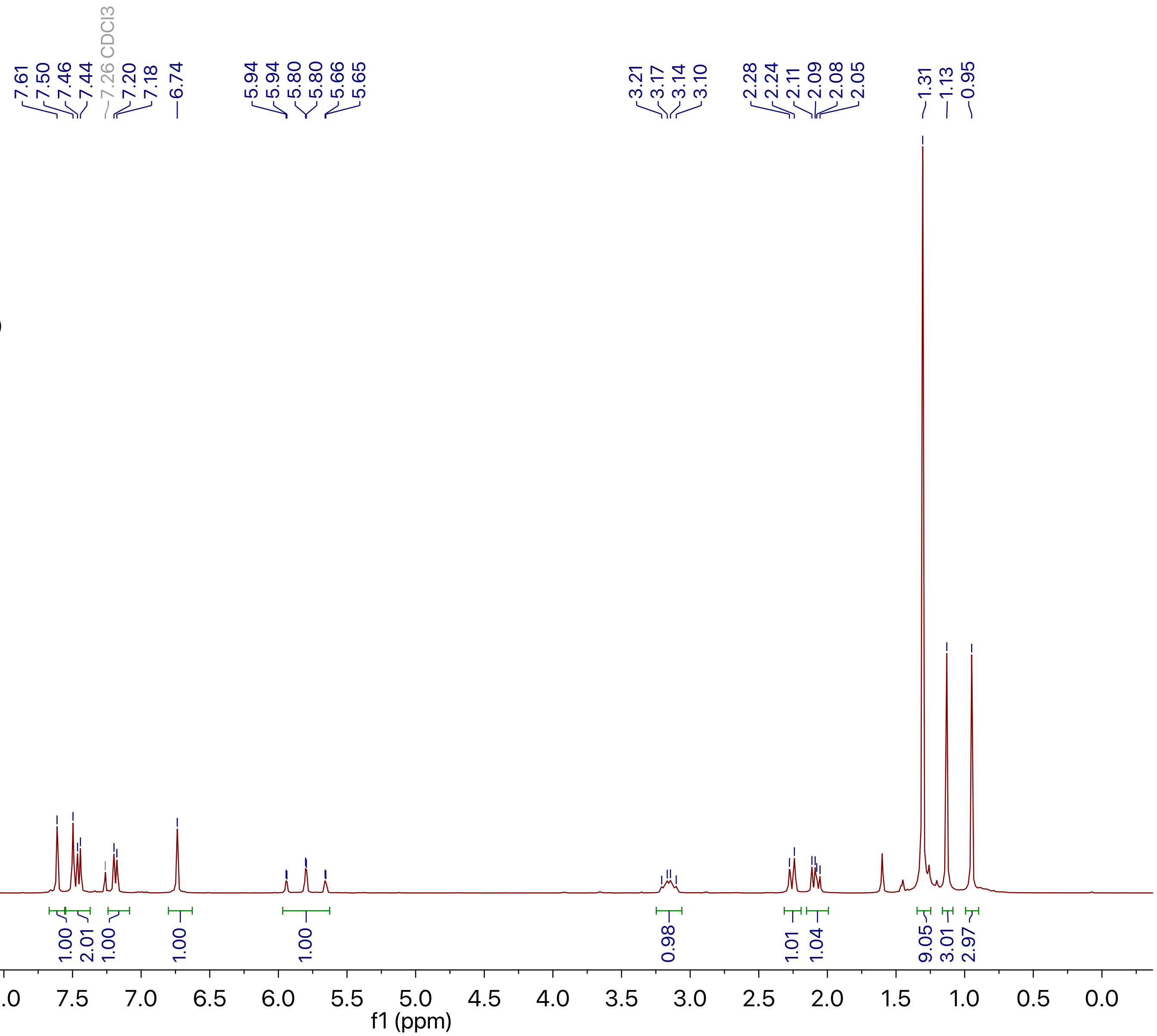

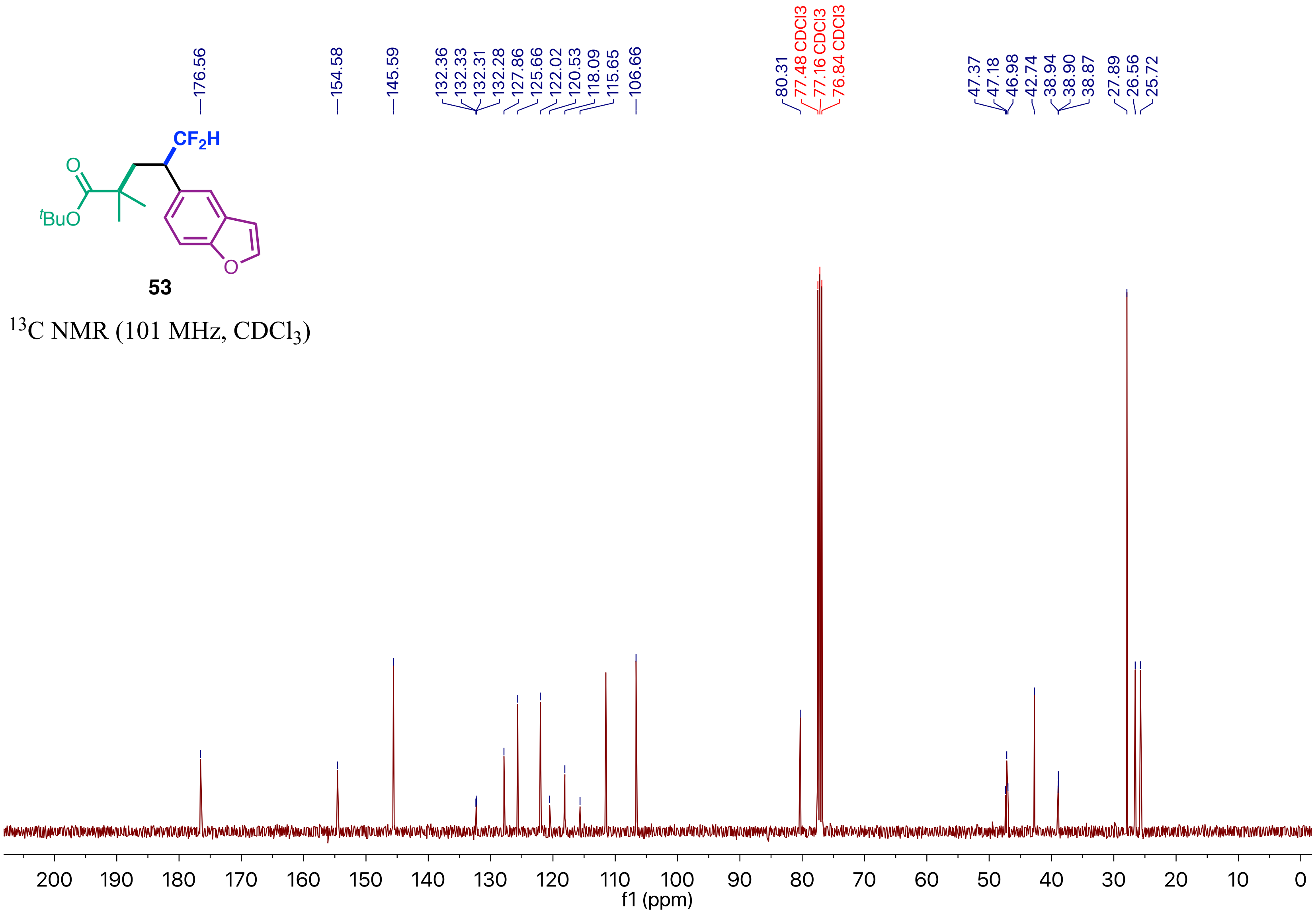

^{13}C NMR (101 MHz, $CDCl_3$)

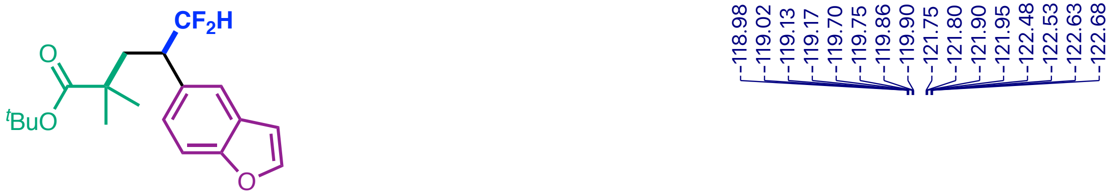

53

^{19}F NMR (376 MHz, CDCl$_3$)

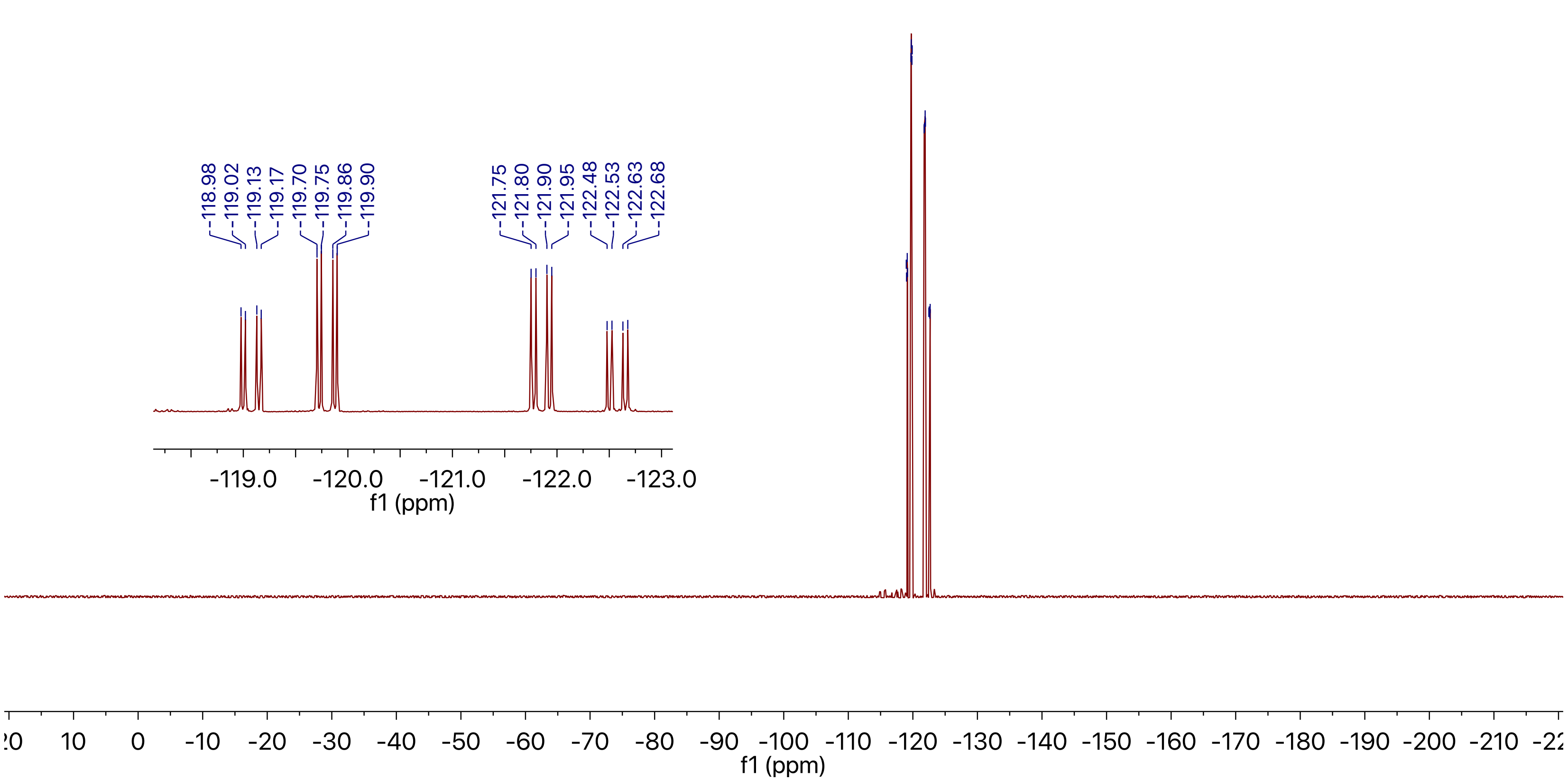

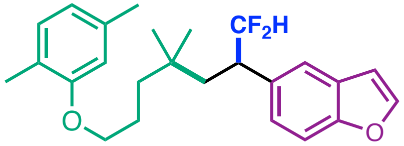

54

^1H NMR (400 MHz, $CDCl_3$)

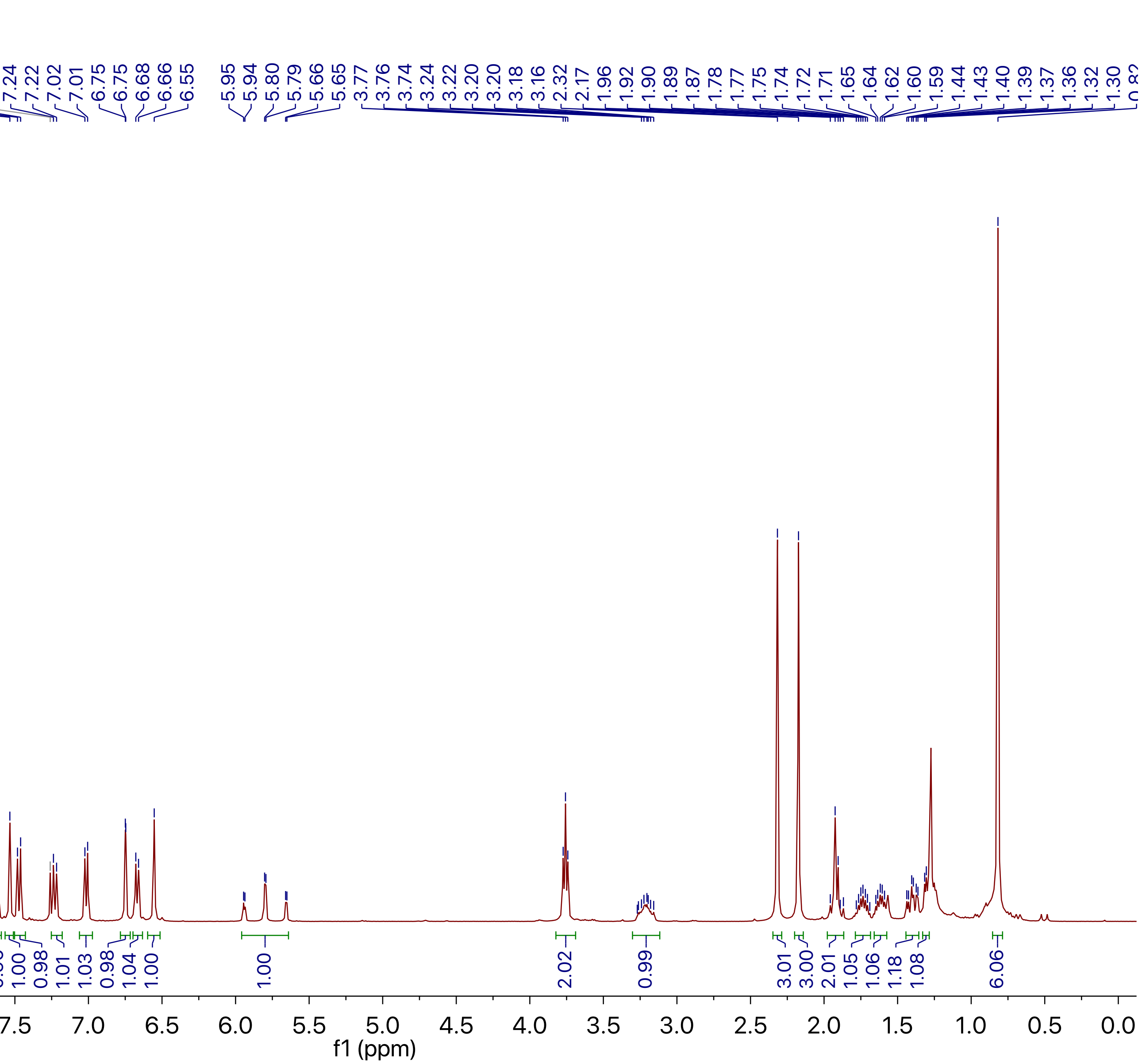

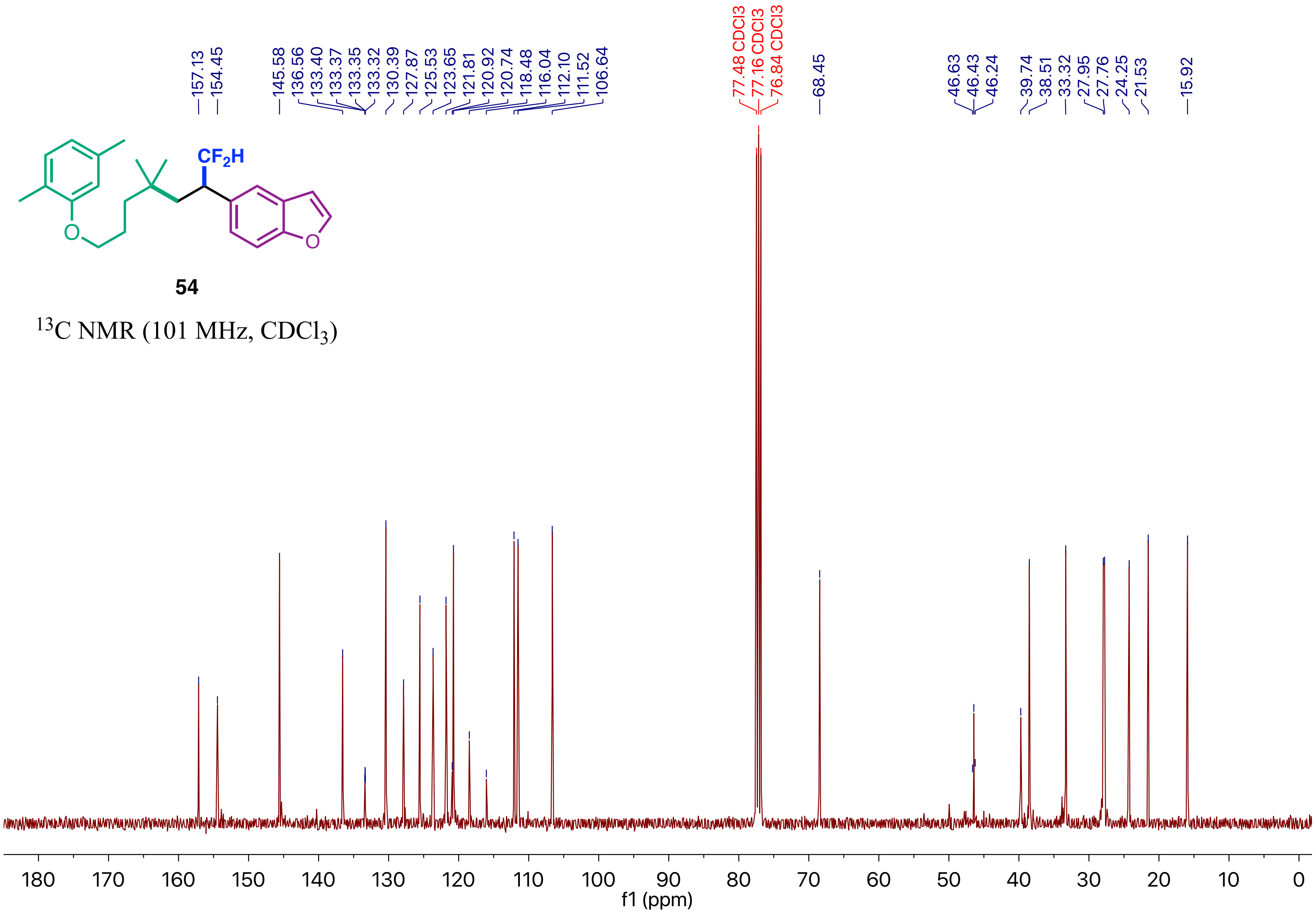

54

^{13}C NMR (101 MHz, $CDCl_3$)

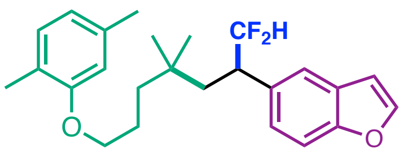

54

^{19}F NMR (376 MHz, $CDCl_3$)

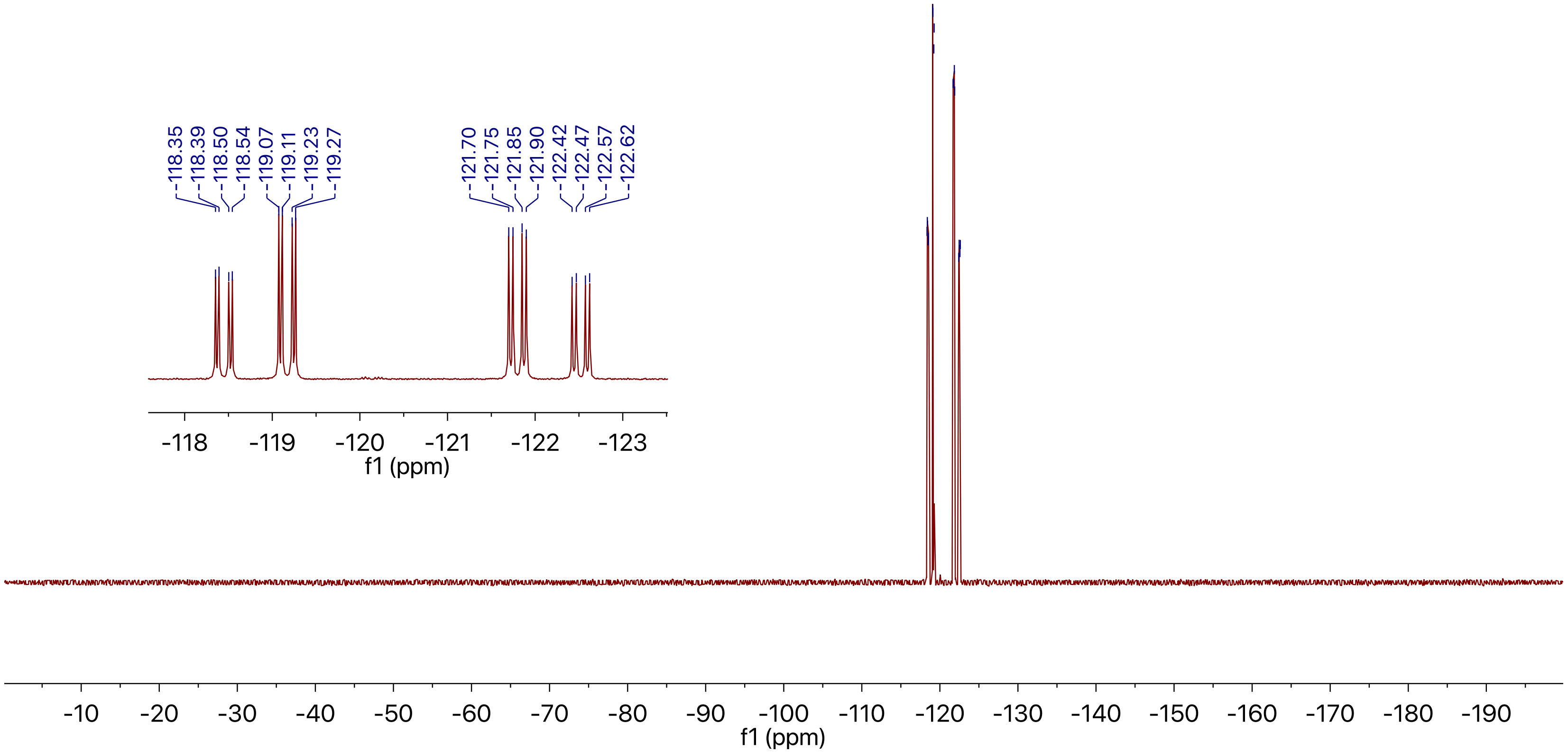

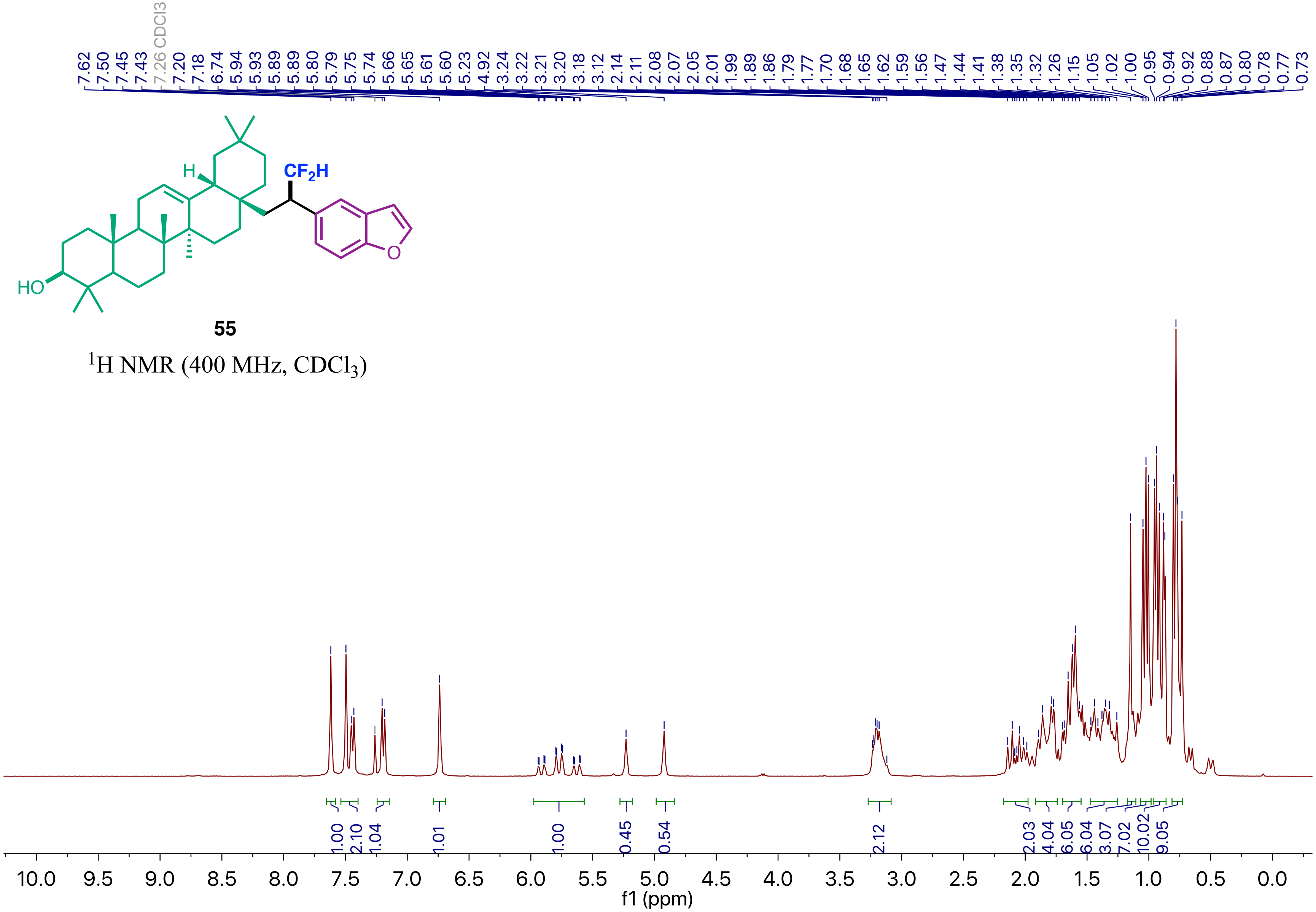

55

^{1}H NMR (400 MHz, $CDCl_3$)

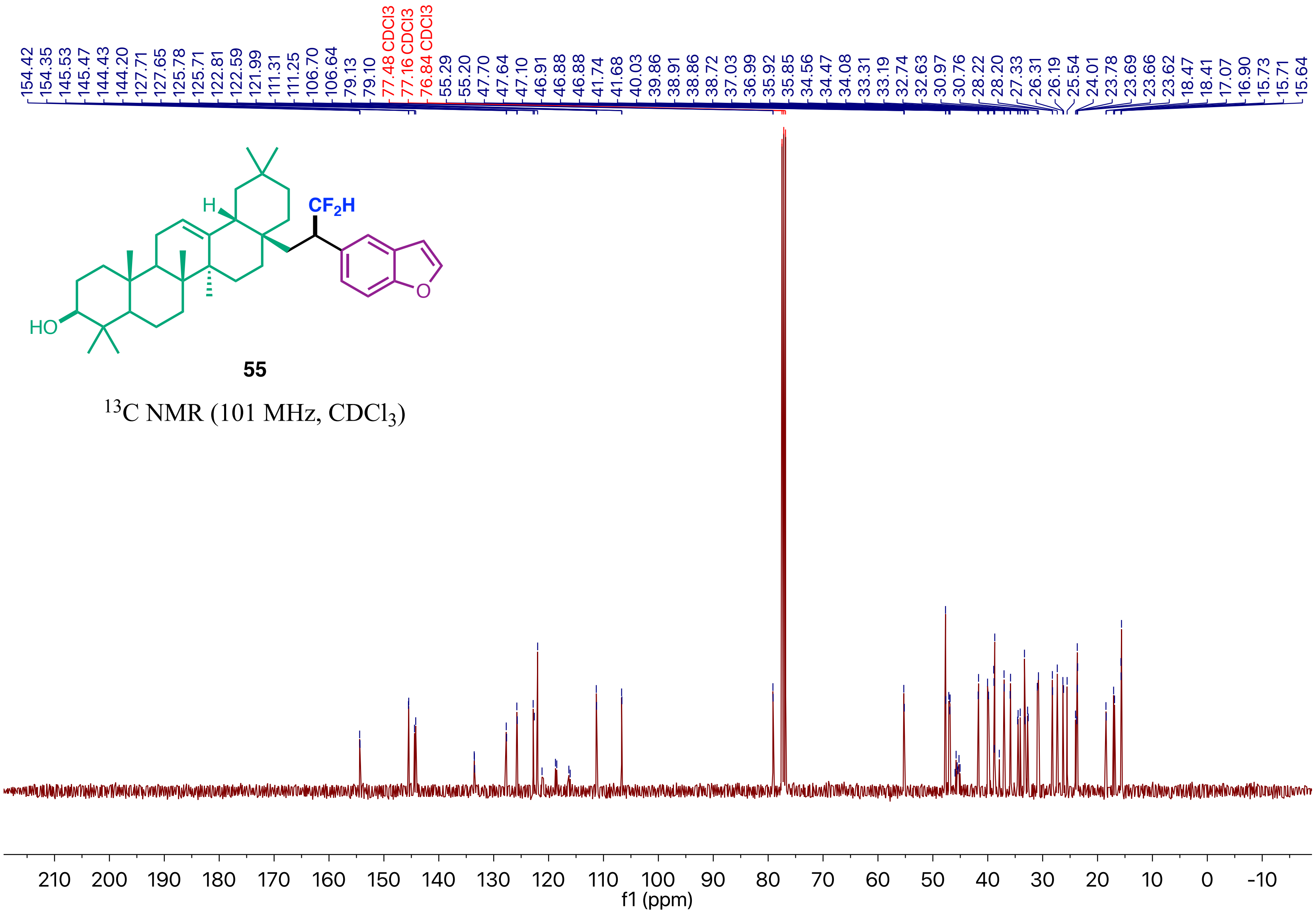
154.42
154.35
145.53
145.47
144.43
144.20
127.71
127.65
125.78
125.71
122.81
122.59
121.99
111.31
111.25
106.70
106.64
79.13
79.10
77.48 CDCl3
77.16 CDCl3
76.84 CDCl3
55.29
55.20
47.70
47.64
47.10
46.91
46.88
46.88
41.74
41.68
40.03
39.86
38.91
38.86
38.72
37.03
36.99
35.92
35.85
34.56
34.47
34.08
33.31
33.19
32.74
32.63
30.97
30.76
28.22
28.20
27.33
26.31
26.19
25.54
24.01
23.78
23.69
23.66
23.62
18.47
18.41
17.07
16.90
15.73
15.71
15.64
CF2H
H
HO
O
55
13C NMR (101 MHz, CDCl3)
210
200
190
180
170
160
150
140
130
120
110
100
90
80
70
60
50
40
30
20
10
0
-10
f1 (ppm)

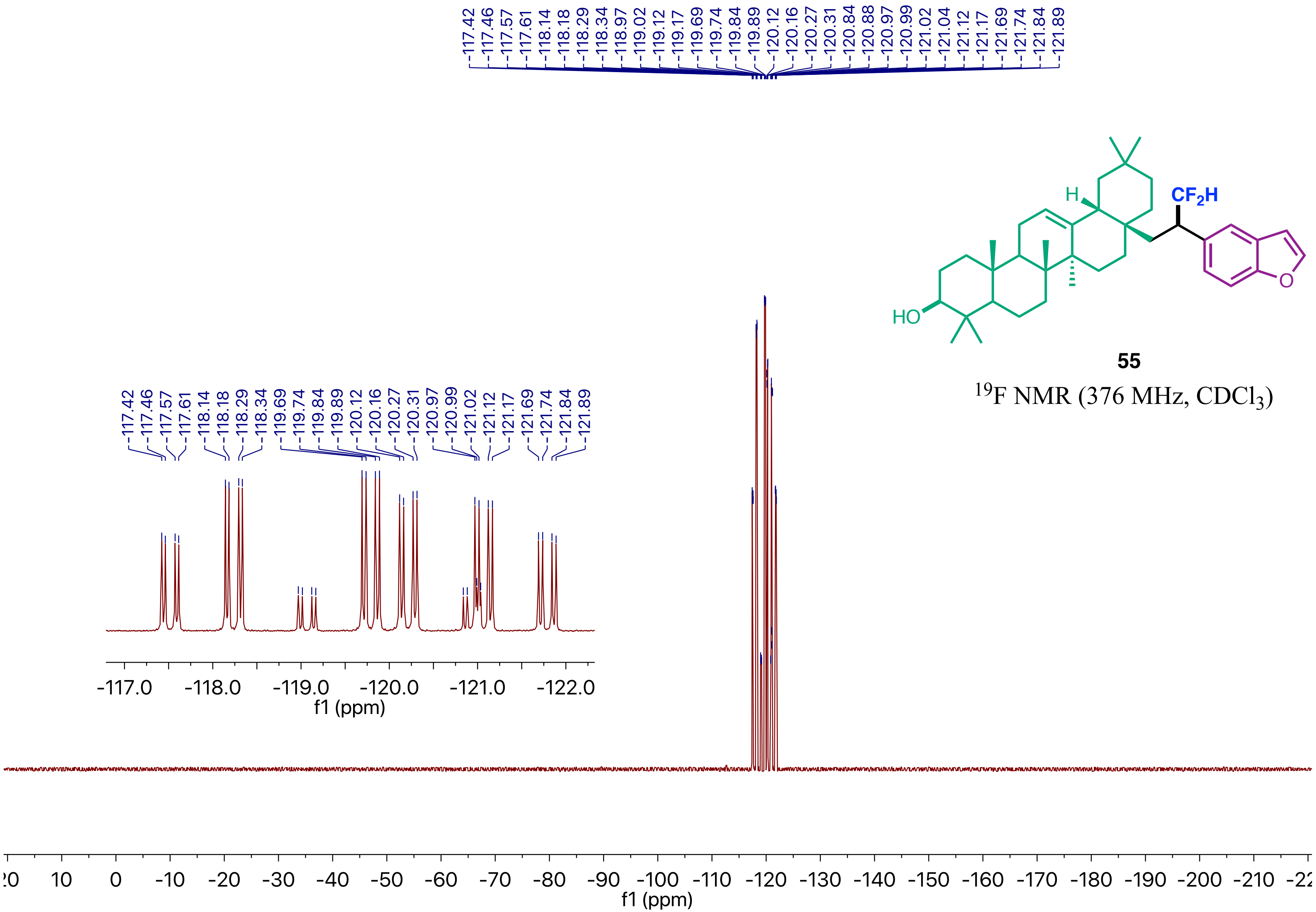

55

^{19}F NMR (376 MHz, $CDCl_3$)

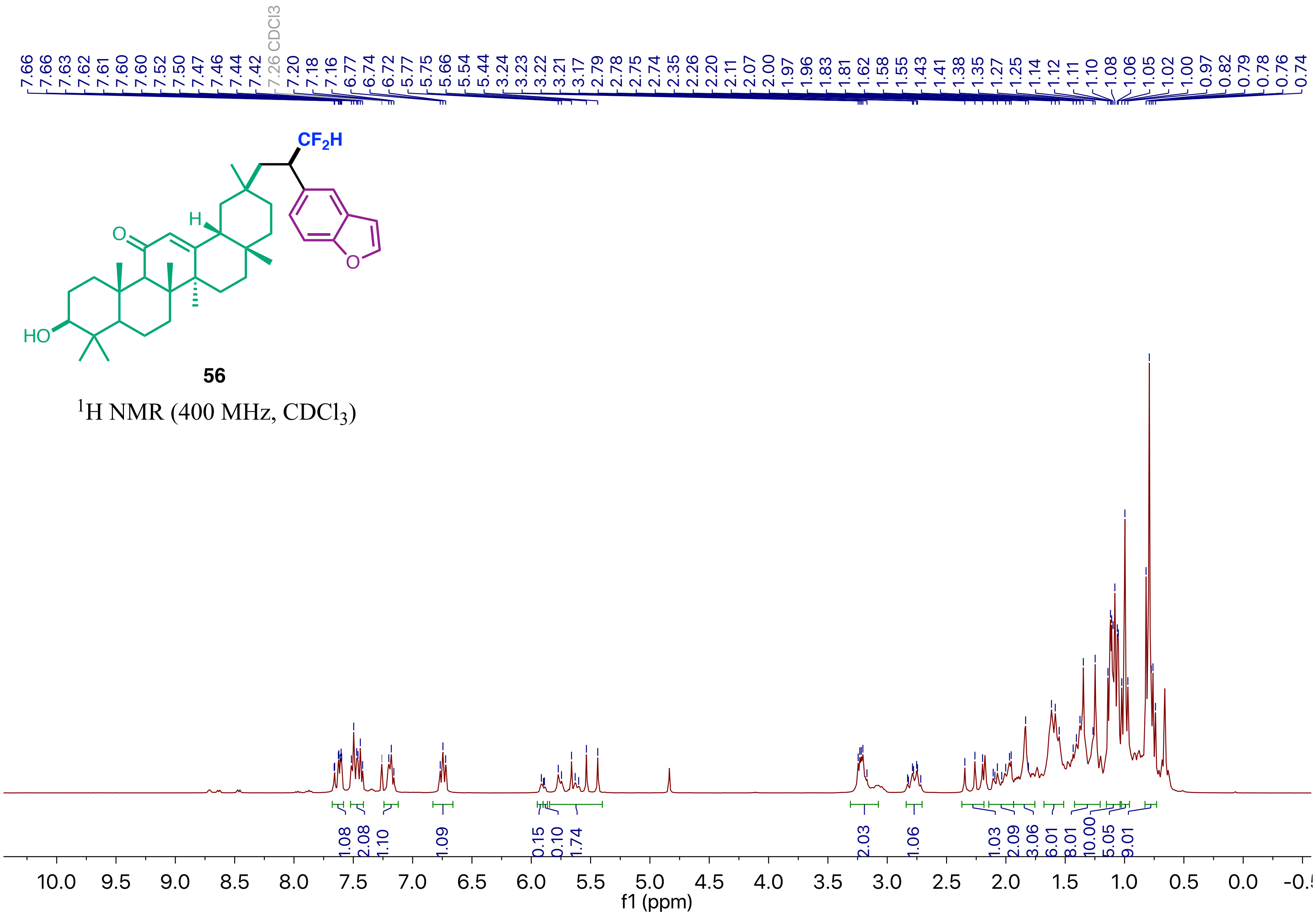
7.66
7.66
7.63
7.62
7.61
7.60
7.60
7.52
7.50
7.47
7.46
7.44
7.42
7.26 CDCl3
7.20
7.18
7.16
6.77
6.74
6.72
5.77
5.75
5.66
5.54
5.44
3.24
3.23
3.22
3.21
3.17
2.79
2.78
2.75
2.74
2.35
2.26
2.20
2.11
2.07
2.00
1.97
1.96
1.83
1.81
1.62
1.58
1.55
1.43
1.41
1.38
1.35
1.27
1.25
1.14
1.12
1.11
1.10
1.08
1.06
1.05
1.02
1.00
0.97
0.82
0.79
0.78
0.76
0.74
CF2H
H
O
HO
O
56
1H NMR (400 MHz, CDCl3)
1.08
2.08
1.10
1.09
0.15
0.10
1.74
2.03
1.06
1.03
2.09
3.06
6.01
8.01
10.00
5.05
9.01
10.0
9.5
9.0
8.5
8.0
7.5
7.0
6.5
6.0
5.5
5.0
4.5
4.0
3.5
3.0
2.5
2.0
1.5
1.0
0.5
0.0
-0.5
f1 (ppm)

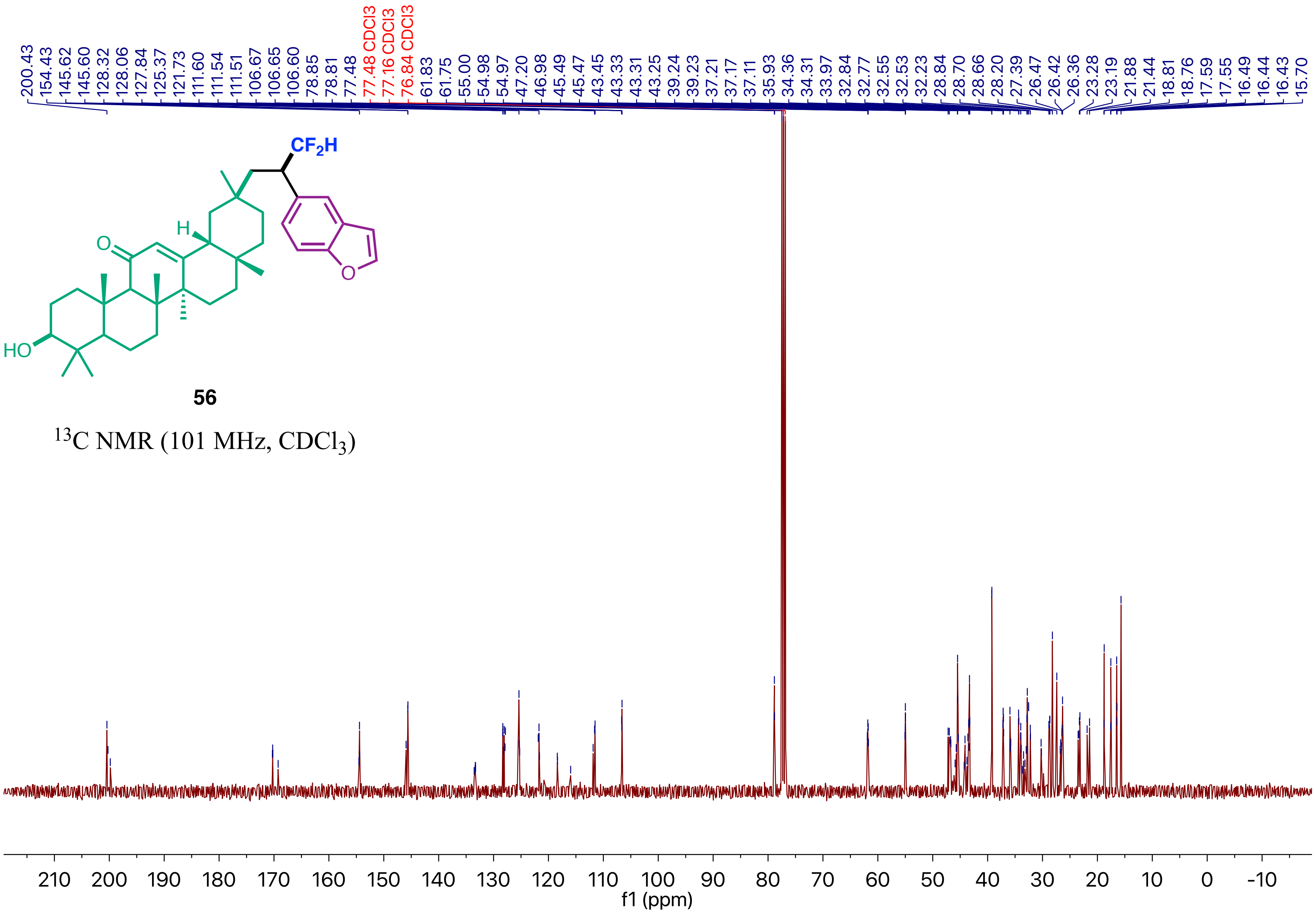

56

^{13}C NMR (101 MHz, $CDCl_3$)

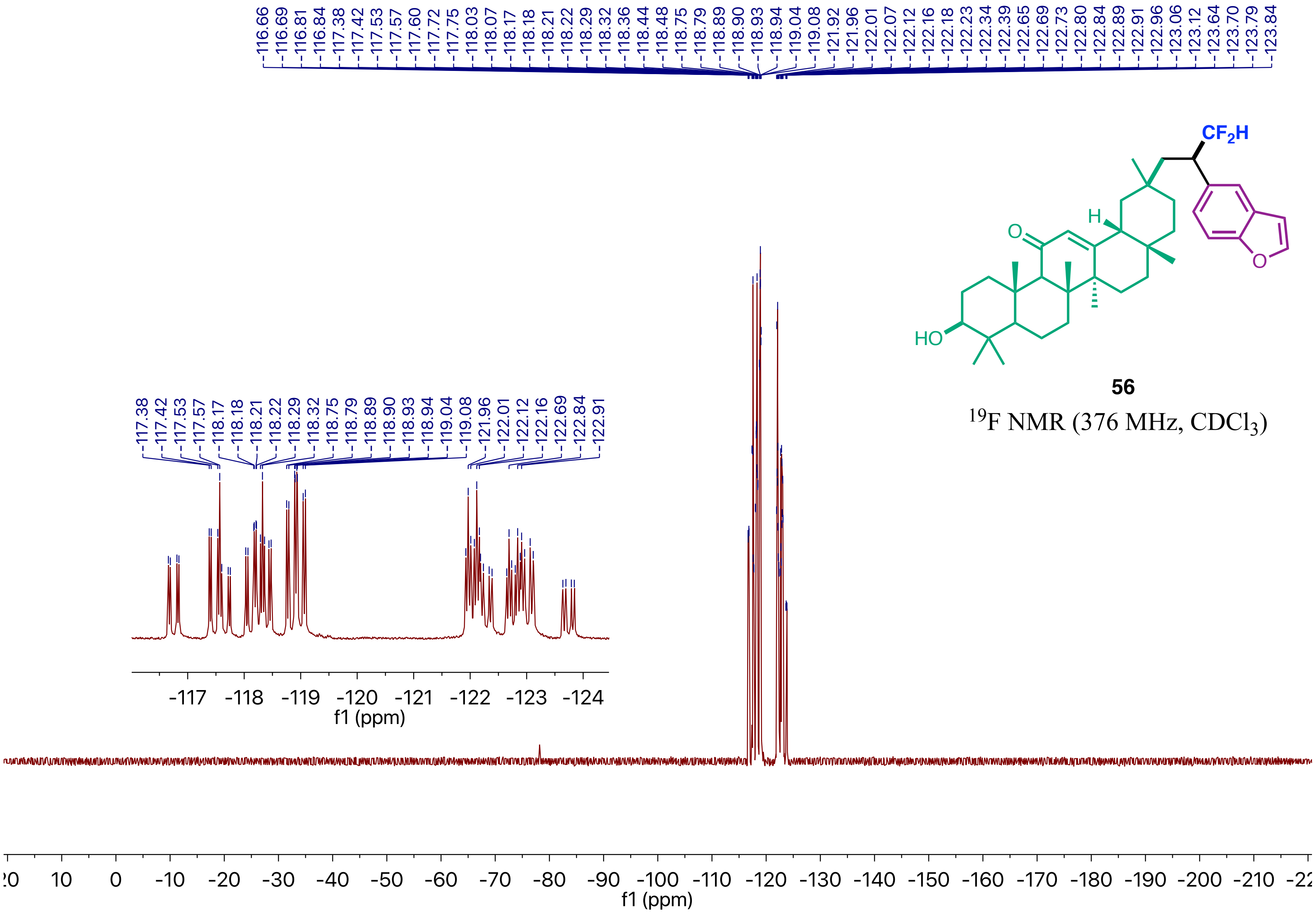

56

^{19}F NMR (376 MHz, $CDCl_3$)

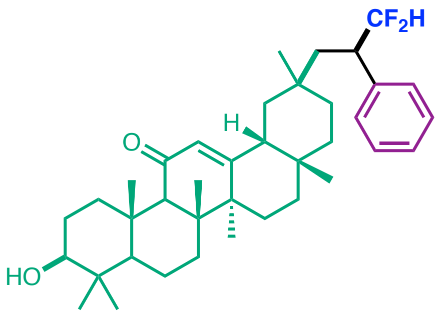

57

^{1}H NMR (400 MHz, $CDCl_3$)

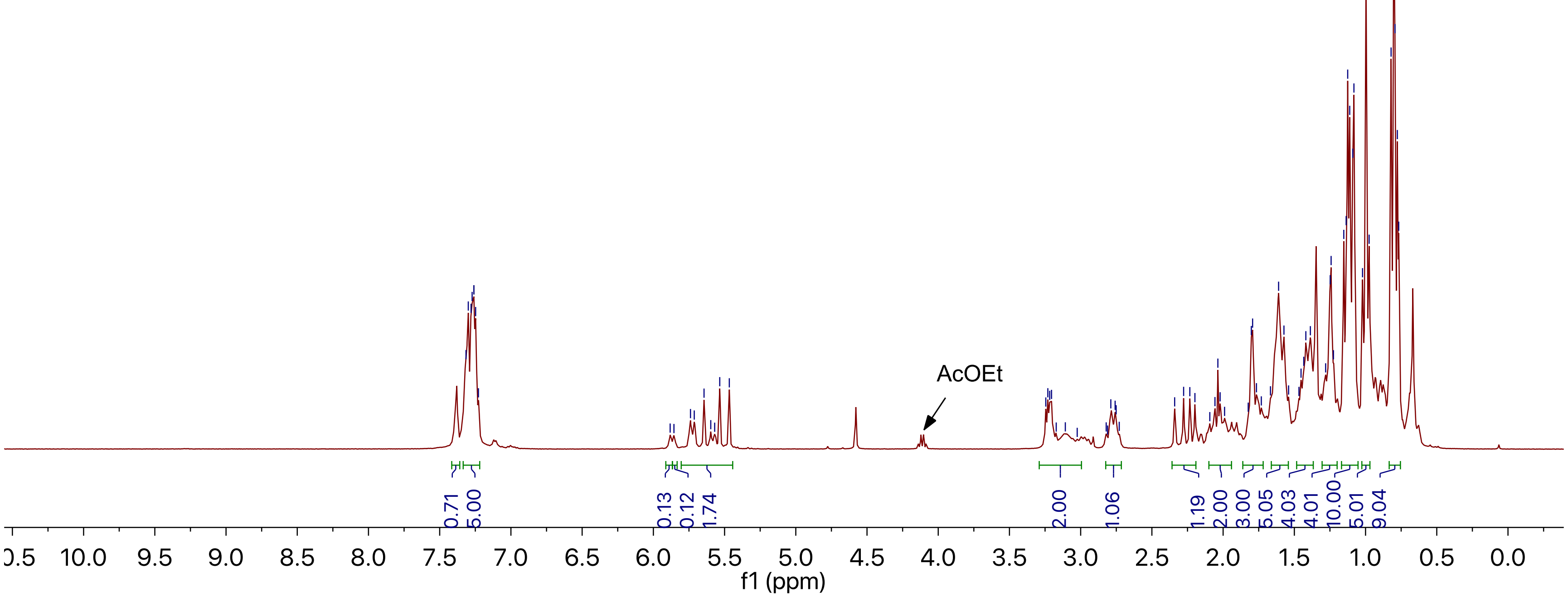

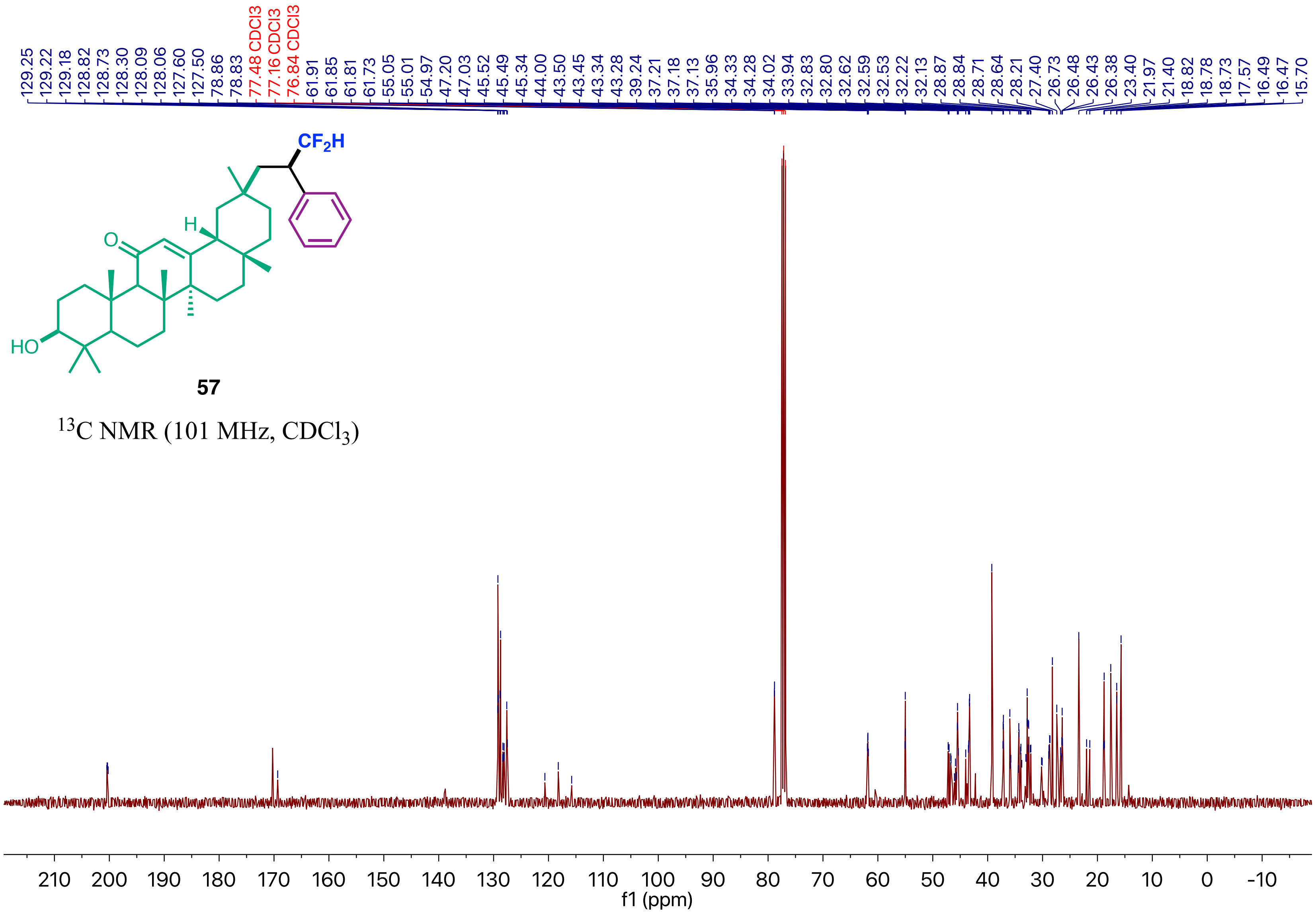
129.25
129.22
129.18
128.82
128.73
128.30
128.09
128.06
127.60
127.50
78.86
78.83
77.48 CDCl3
77.16 CDCl3
76.84 CDCl3
61.91
61.85
61.81
61.73
55.05
55.01
54.97
47.20
47.03
45.52
45.49
45.34
44.00
43.50
43.45
43.34
43.28
39.24
37.21
37.18
37.13
35.96
34.33
34.28
34.02
33.94
32.83
32.80
32.62
32.59
32.53
32.22
32.13
28.87
28.84
28.71
28.64
28.21
27.40
26.73
26.48
26.43
26.38
23.40
21.97
21.40
18.82
18.78
18.73
17.57
16.49
16.47
15.70
CF2H
O
H
HO
57
13C NMR (101 MHz, CDCl3)
210
200
190
180
170
160
150
140
130
120
110
100
90
80
70
60
50
40
30
20
10
0
-10
f1 (ppm)

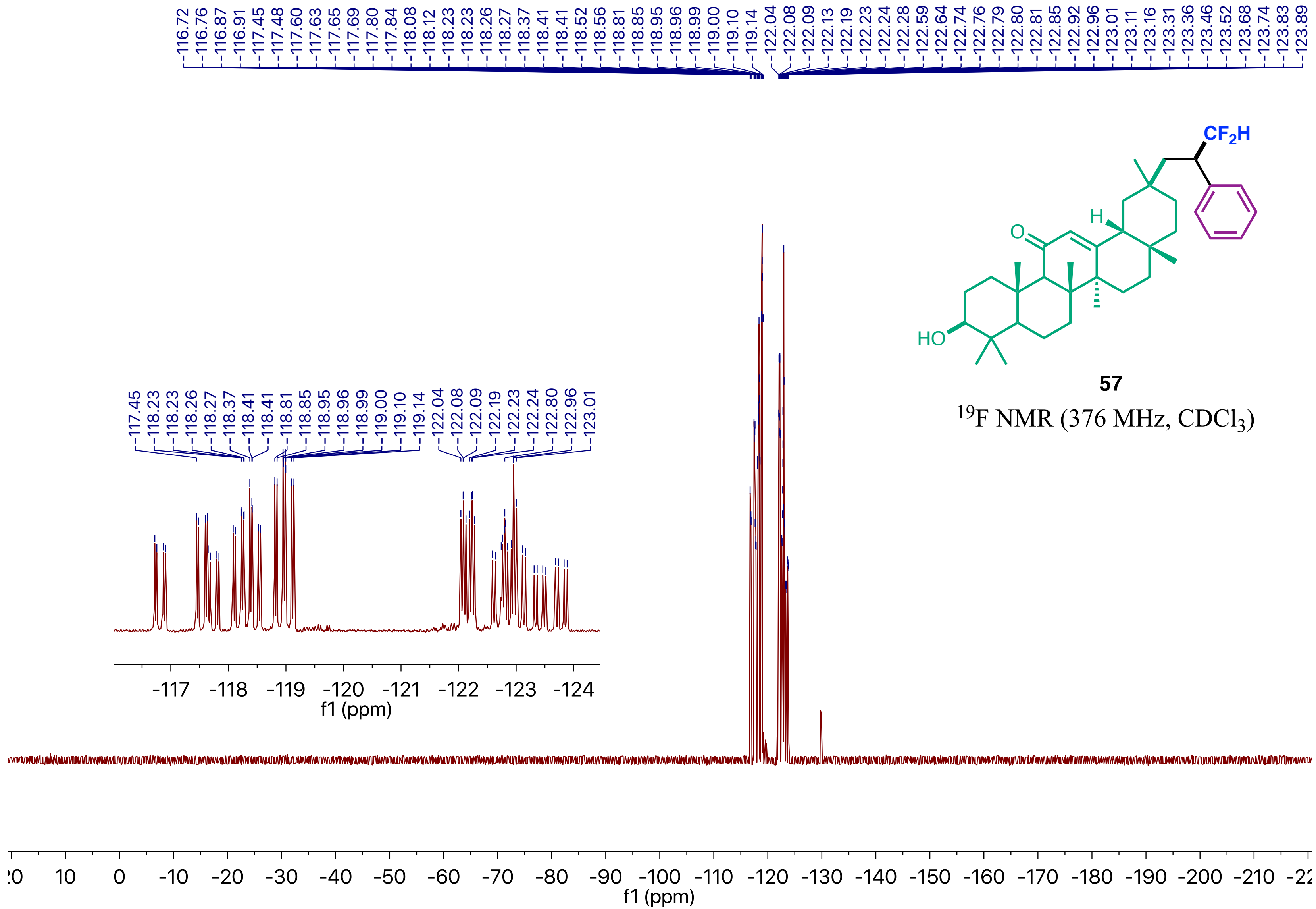

57

^{19}F NMR (376 MHz, CDCl$_3$)

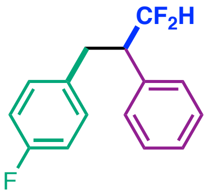

58

^{1}H NMR (400 MHz, $CDCl_3$)

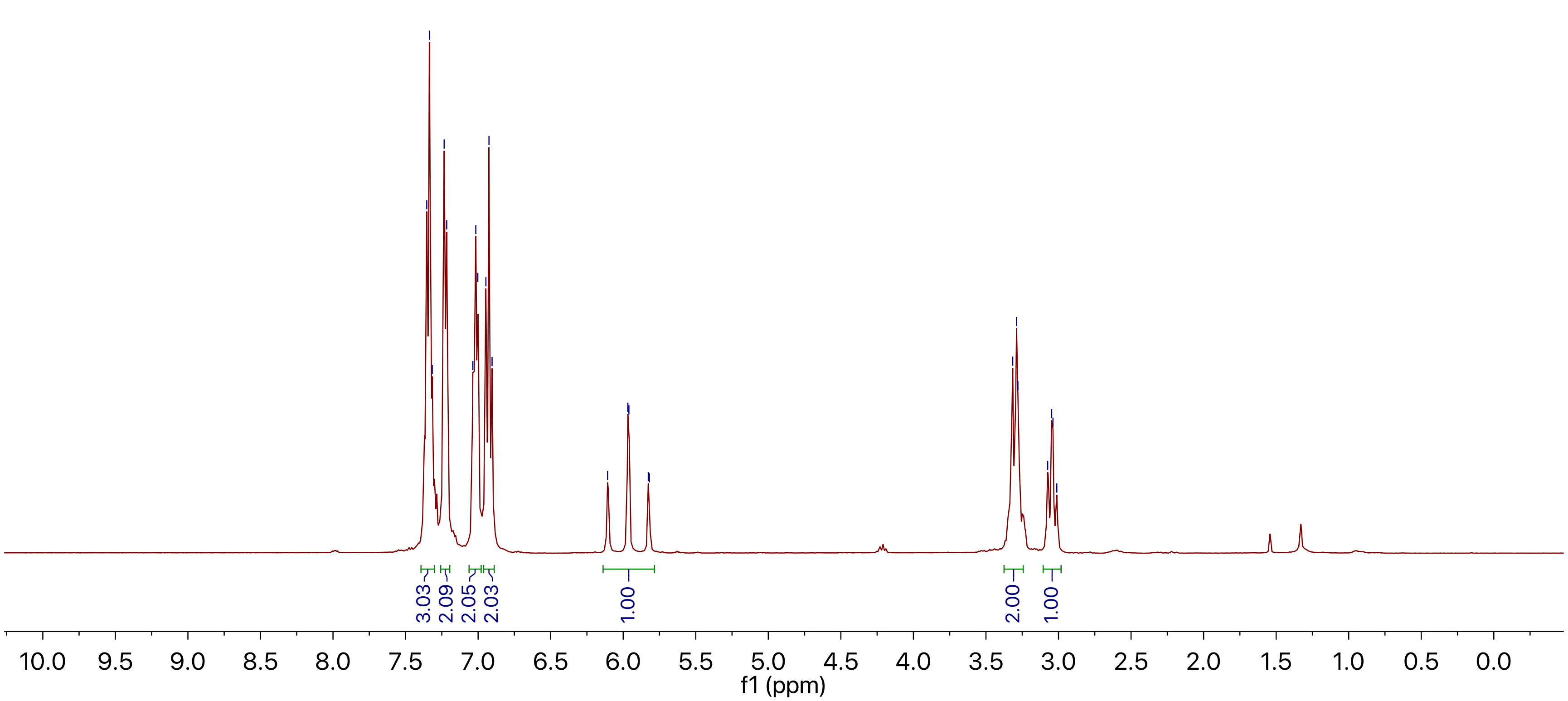

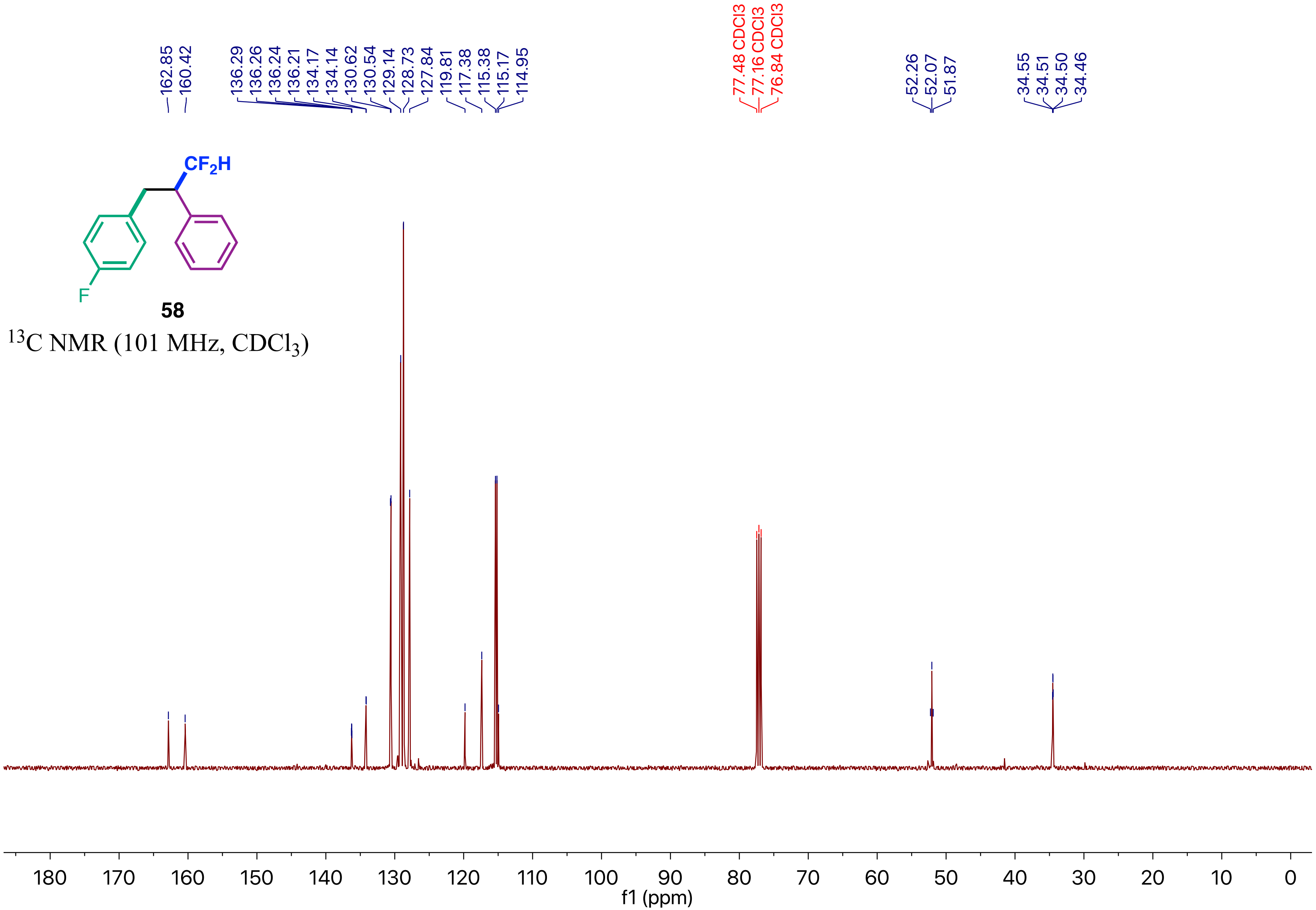

58
13C NMR (101 MHz, CDCl3)
162.85
160.42
136.29
136.26
136.24
136.21
134.17
134.14
130.62
130.54
129.14
128.73
127.84
119.81
117.38
115.38
115.17
114.95
77.48 CDCl3
77.16 CDCl3
76.84 CDCl3
52.26
52.07
51.87
34.55
34.51
34.50
34.46
180 170 160 150 140 130 120 110 100 90 80 70 60 50 40 30 20 10 0
f1 (ppm)

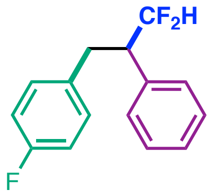

58

^{19}F NMR (376 MHz, $CDCl_3$)

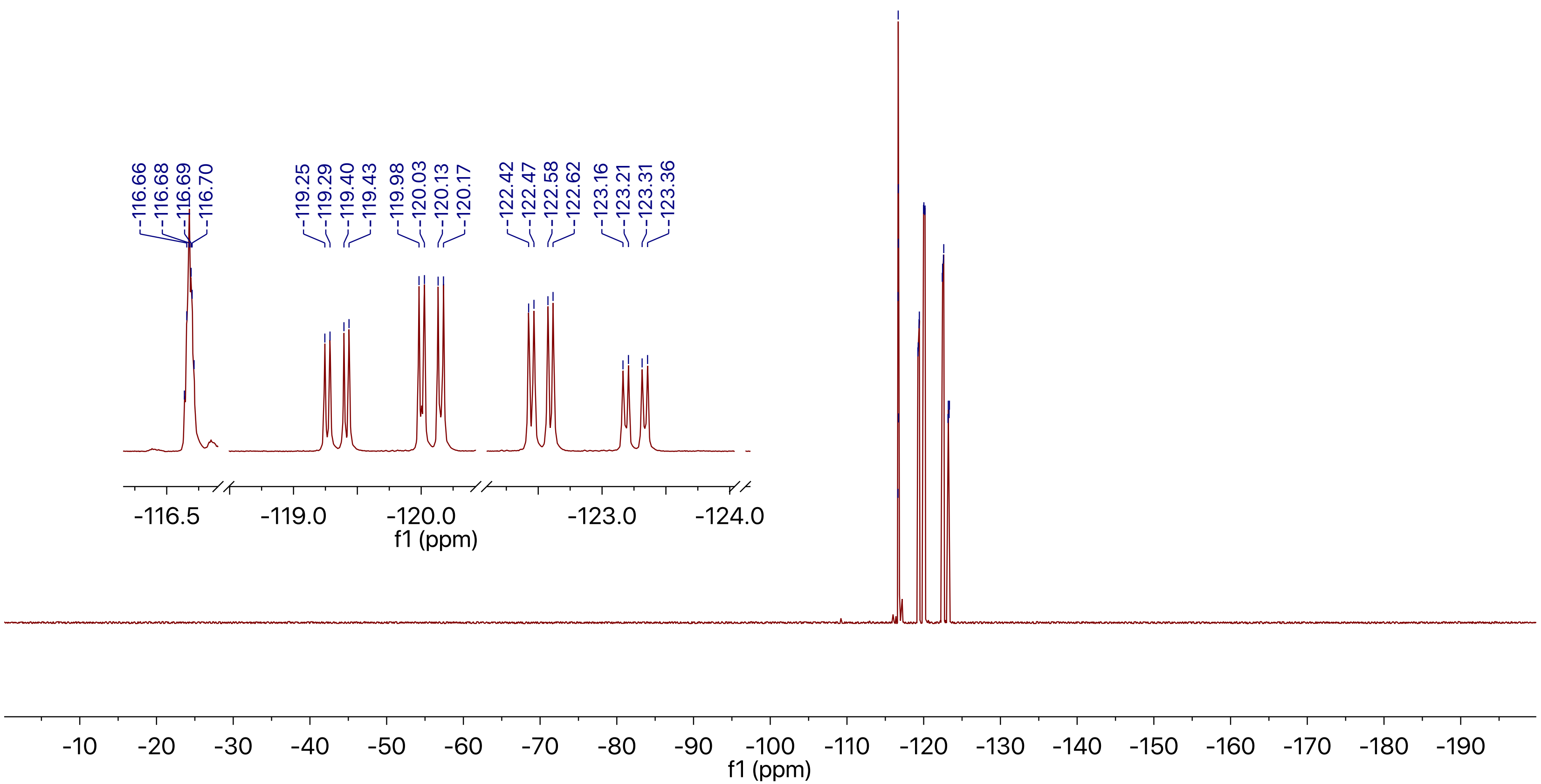

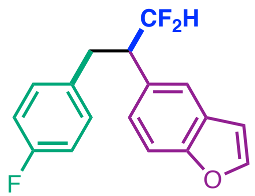

59

^{1}H NMR (400 MHz, $CDCl_3$)

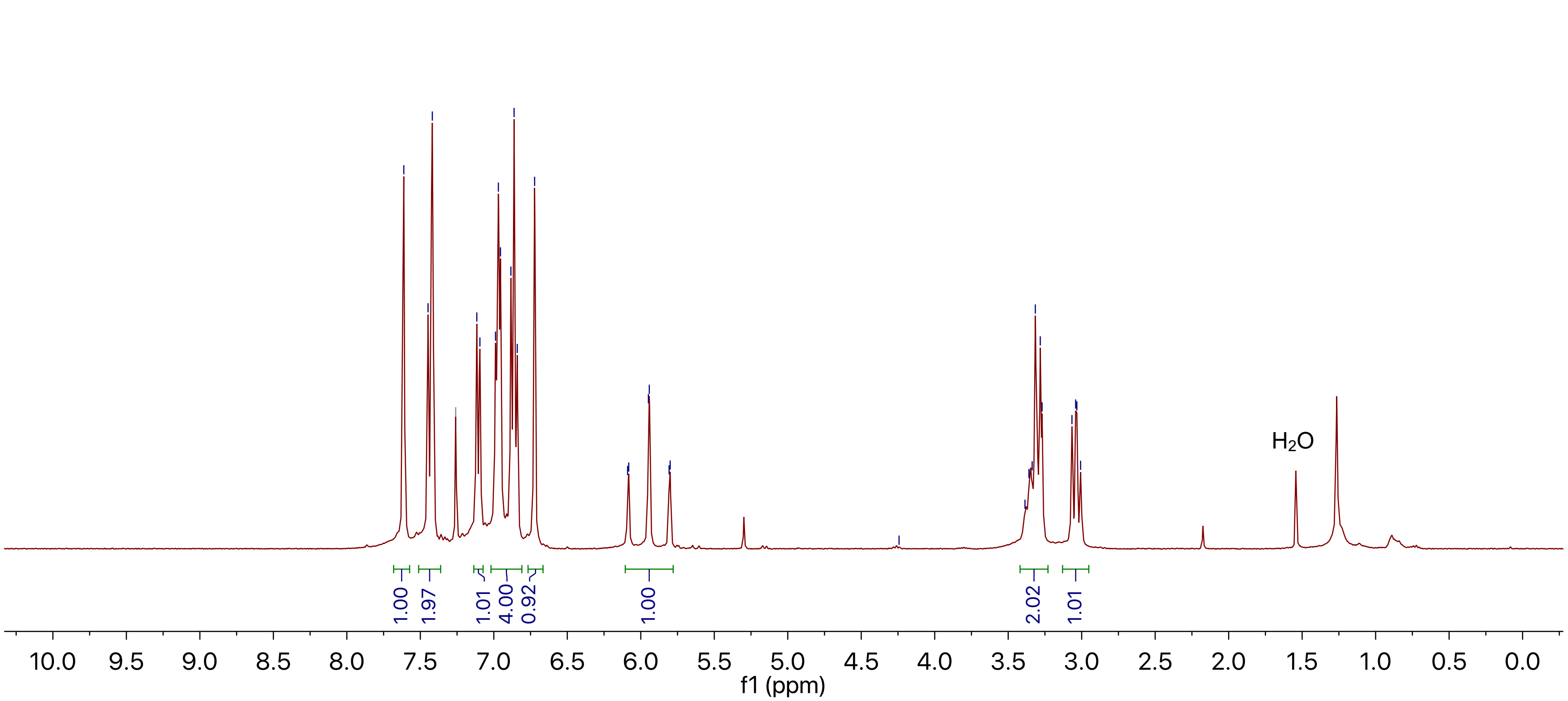

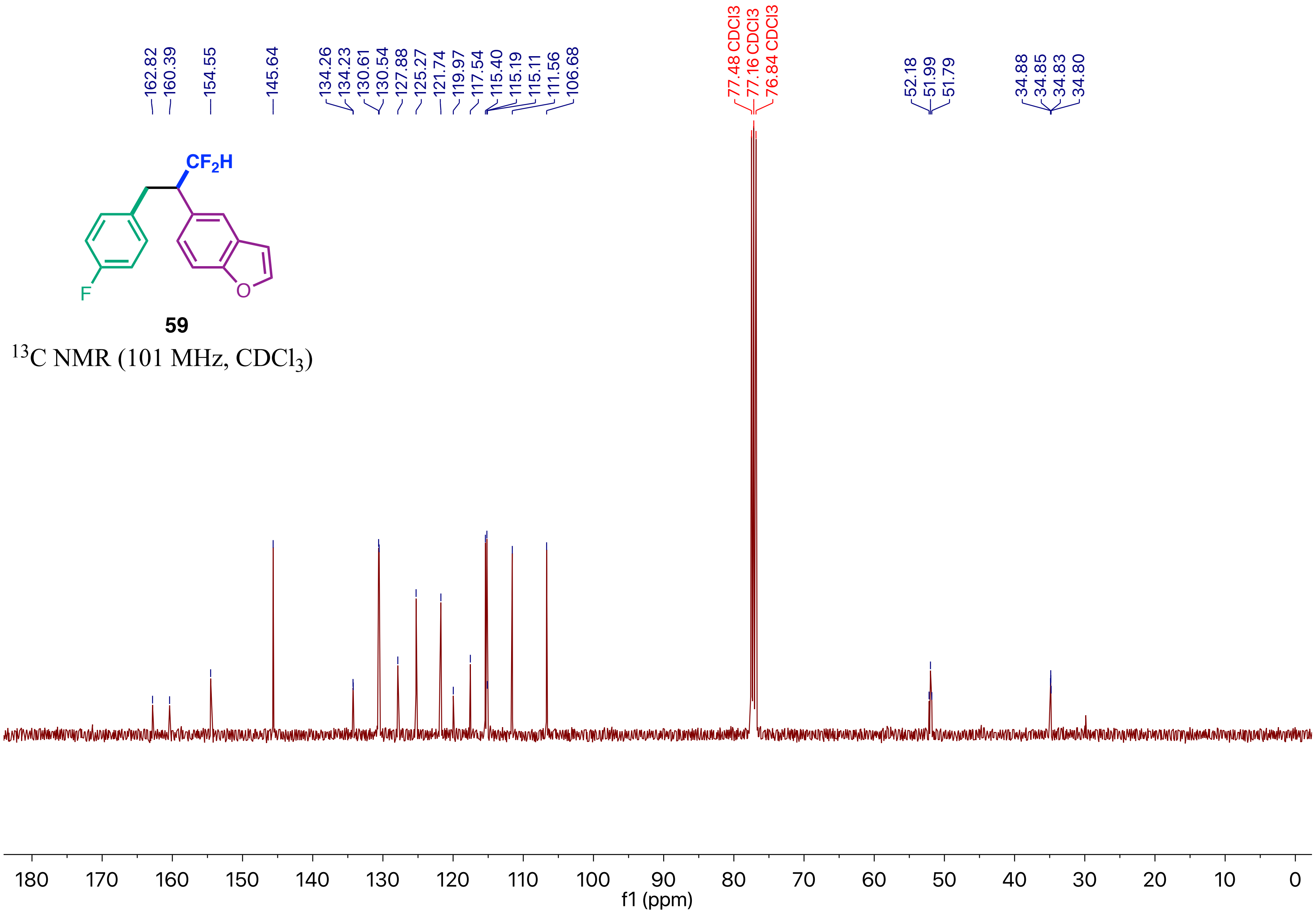
CF2H
F
O
59
13C NMR (101 MHz, CDCl3)
162.82
160.39
154.55
145.64
134.26
134.23
130.61
130.54
127.88
125.27
121.74
119.97
117.54
115.40
115.19
115.11
111.56
106.68
77.48 CDCl3
77.16 CDCl3
76.84 CDCl3
52.18
51.99
51.79
34.88
34.85
34.83
34.80
180 170 160 150 140 130 120 110 100 90 80 70 60 50 40 30 20 10 0
f1 (ppm)

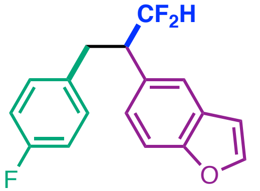

59

^{19}F NMR (376 MHz, $CDCl_3$)

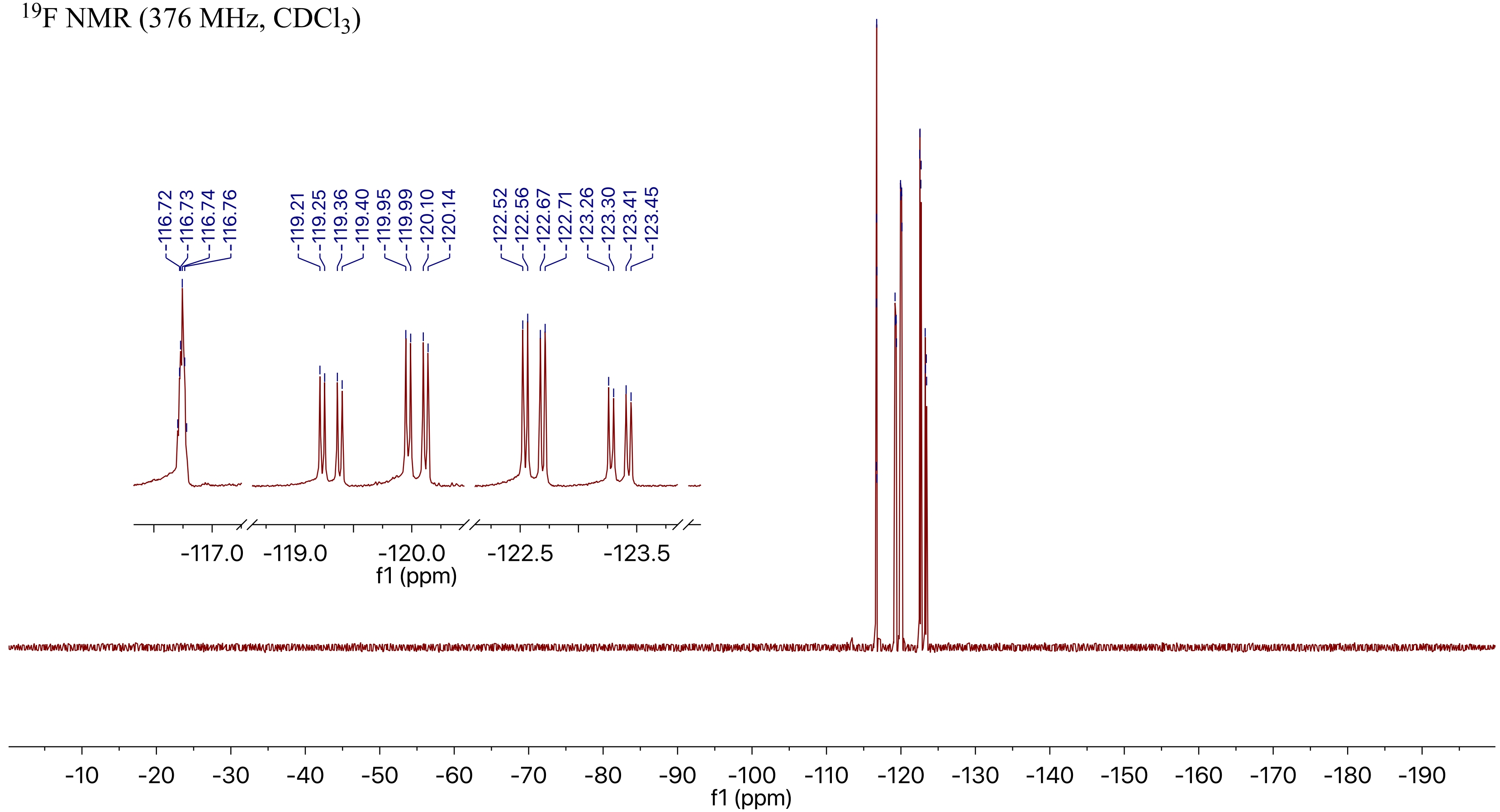

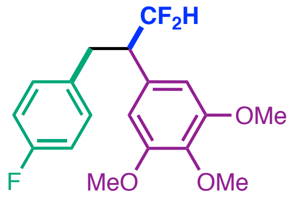

60

^{1}H NMR (400 MHz, $CDCl_3$)

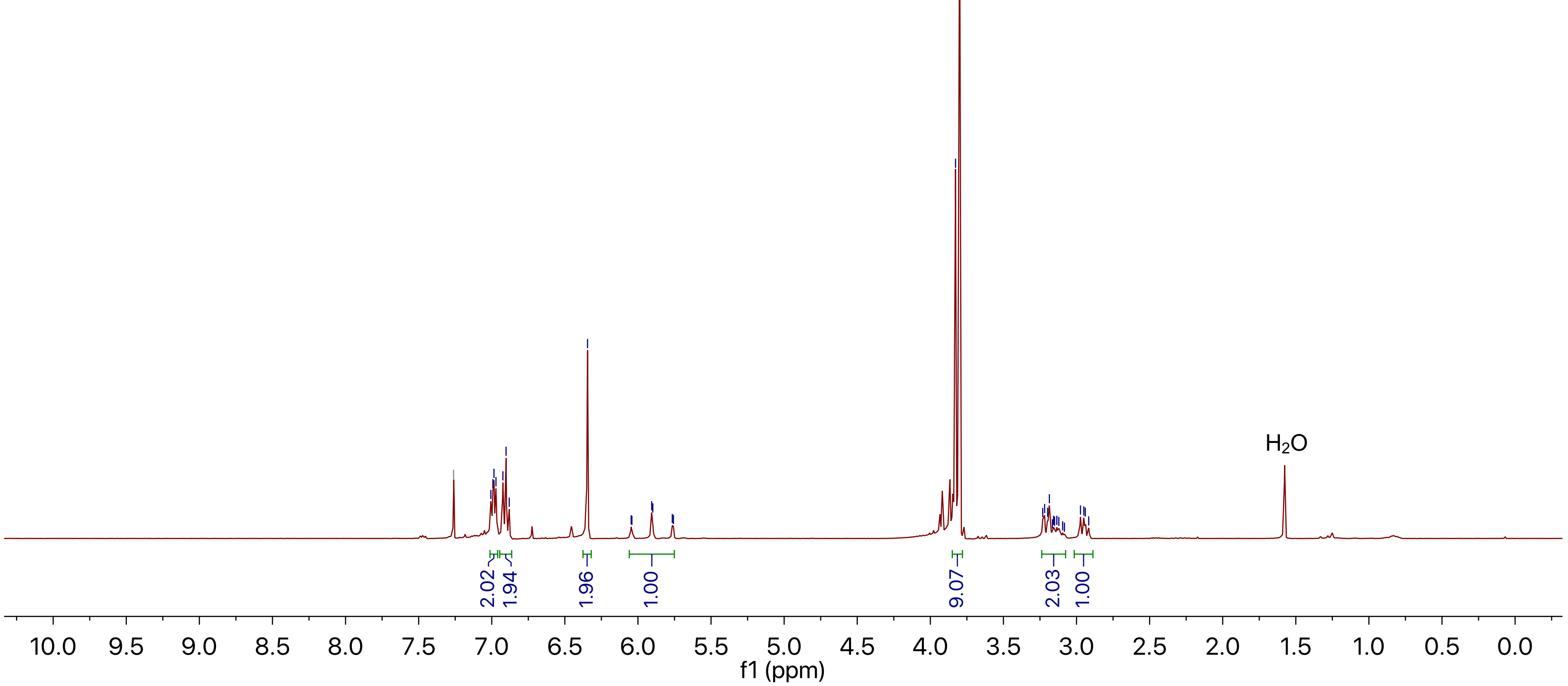

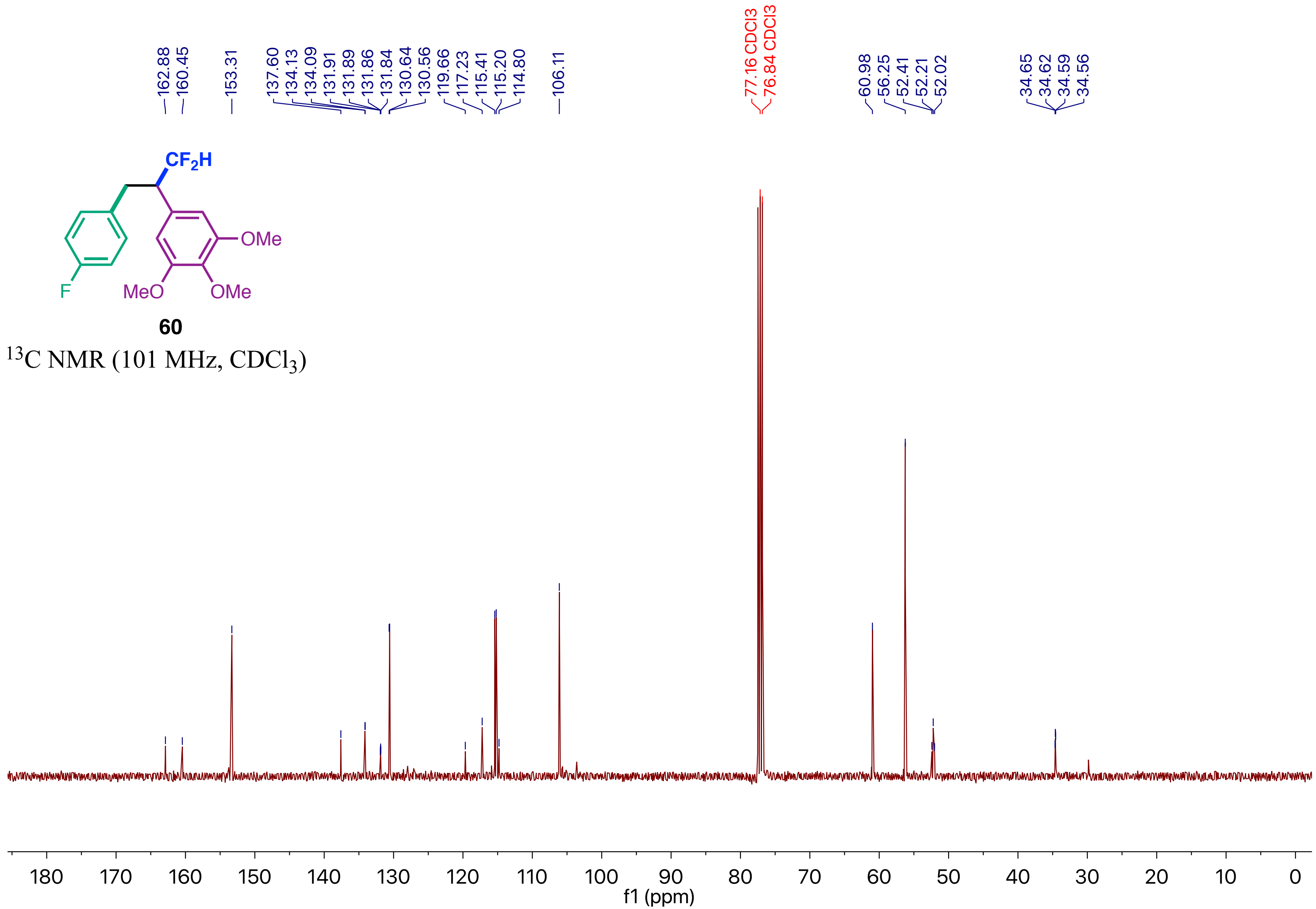

60

^{13}C NMR (101 MHz, $CDCl_3$)

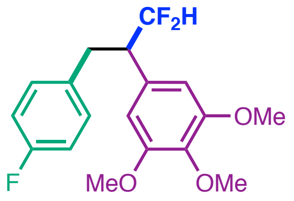

60

^{19}F NMR (376 MHz, $CDCl_3$)

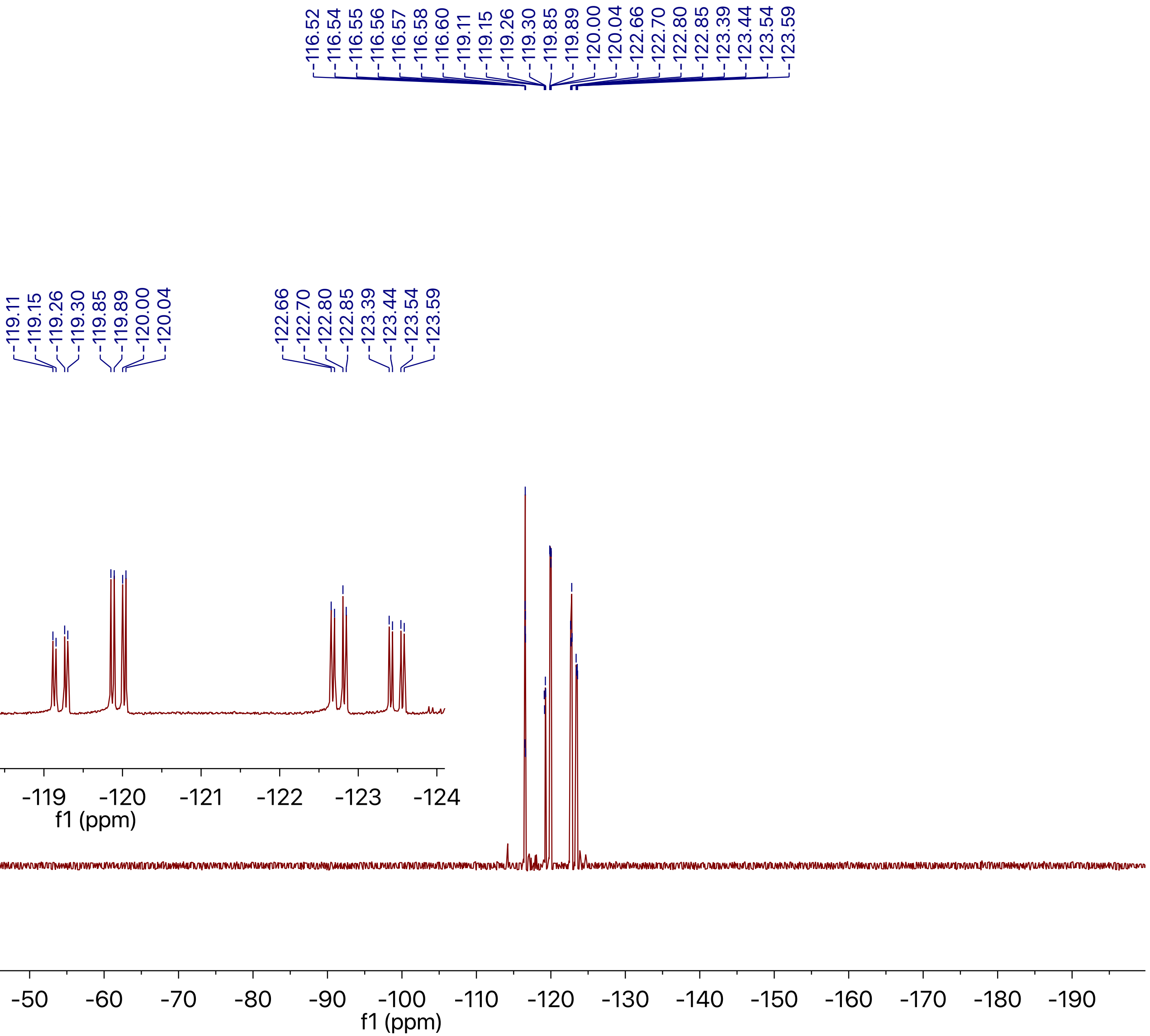

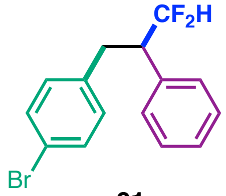

61

^{1}H NMR (400 MHz, $CDCl_3$)

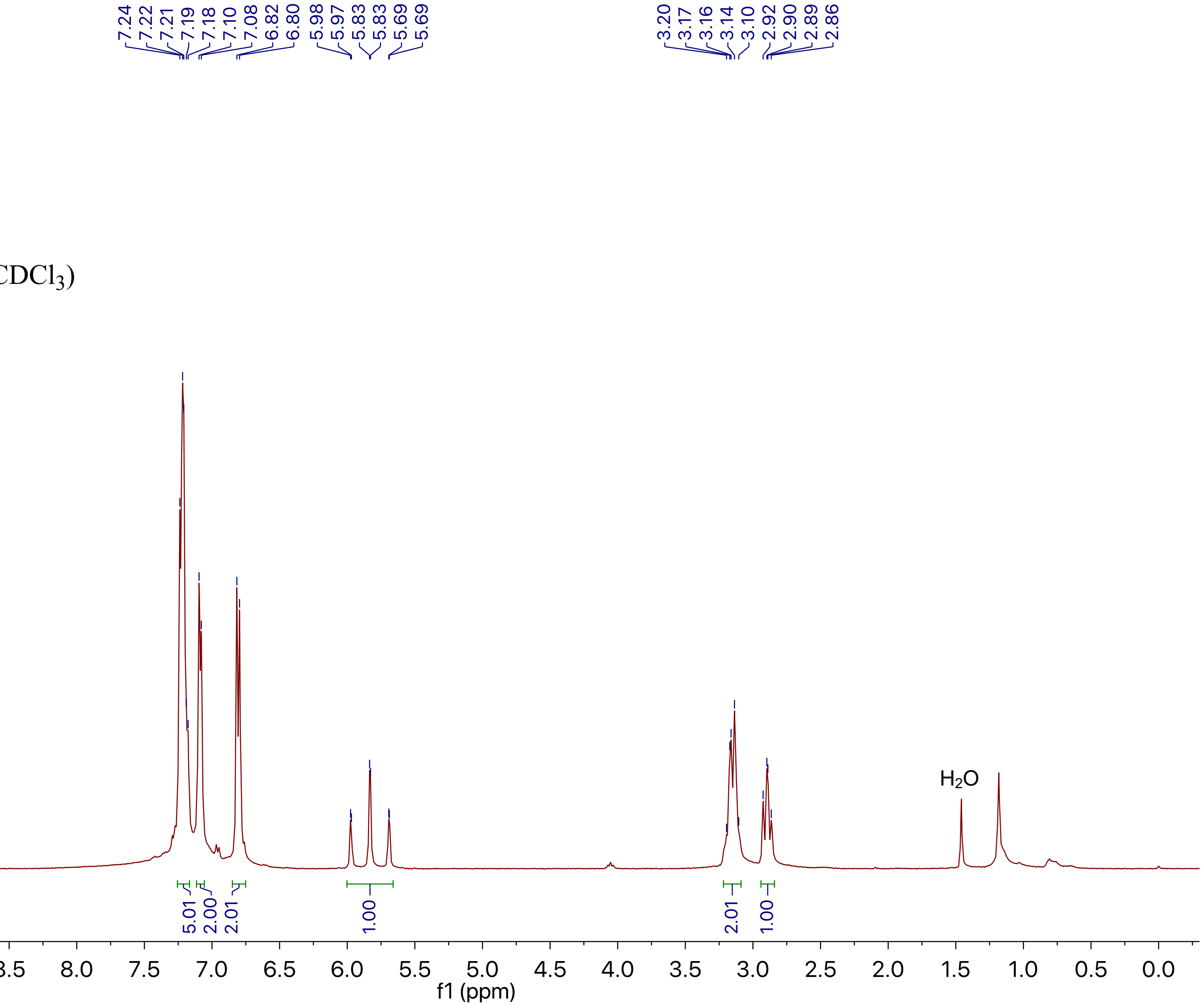

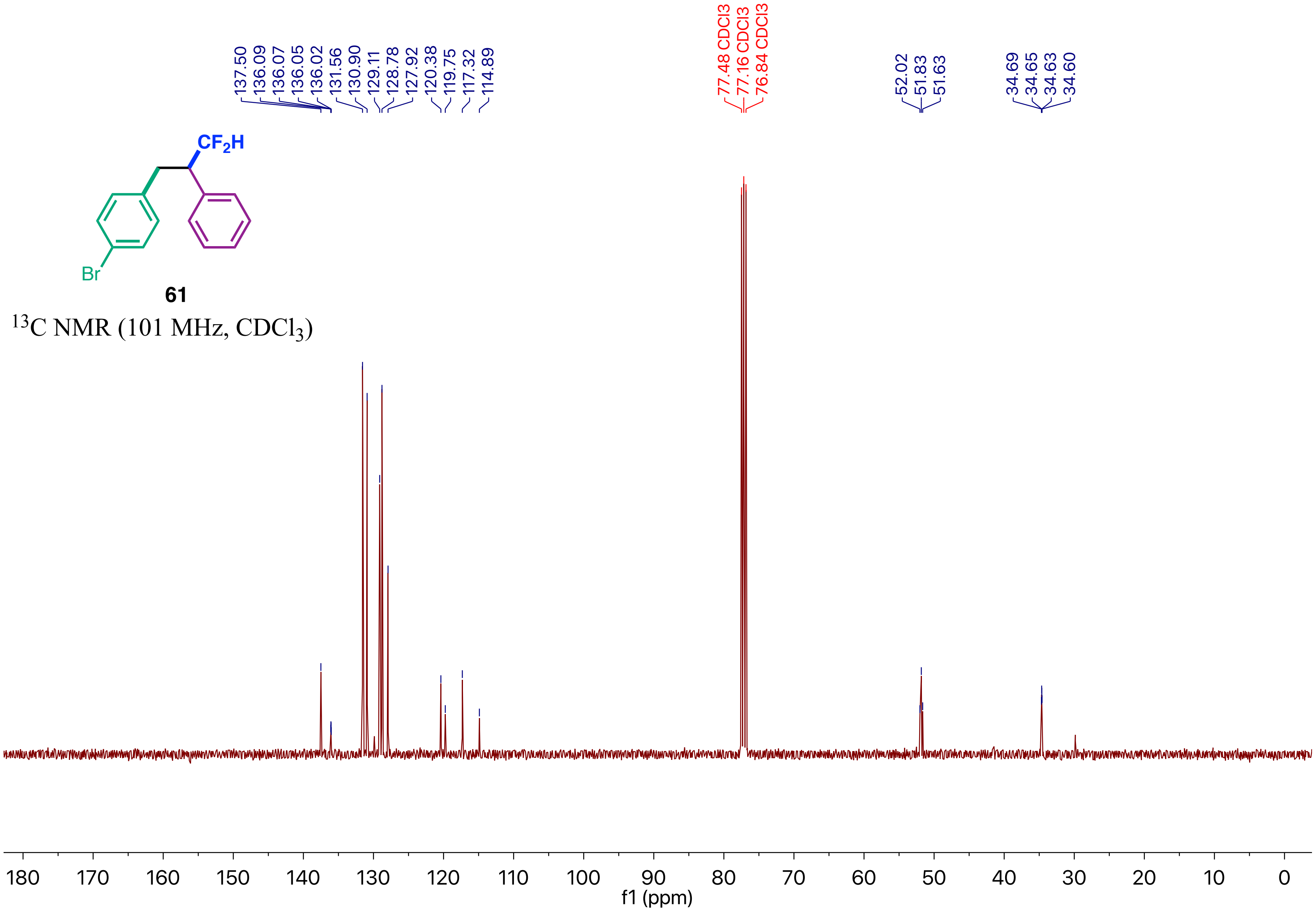

61

^{13}C NMR (101 MHz, $CDCl_3$)

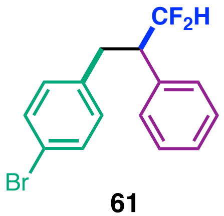

61

^{19}F NMR (376 MHz, $CDCl_3$)

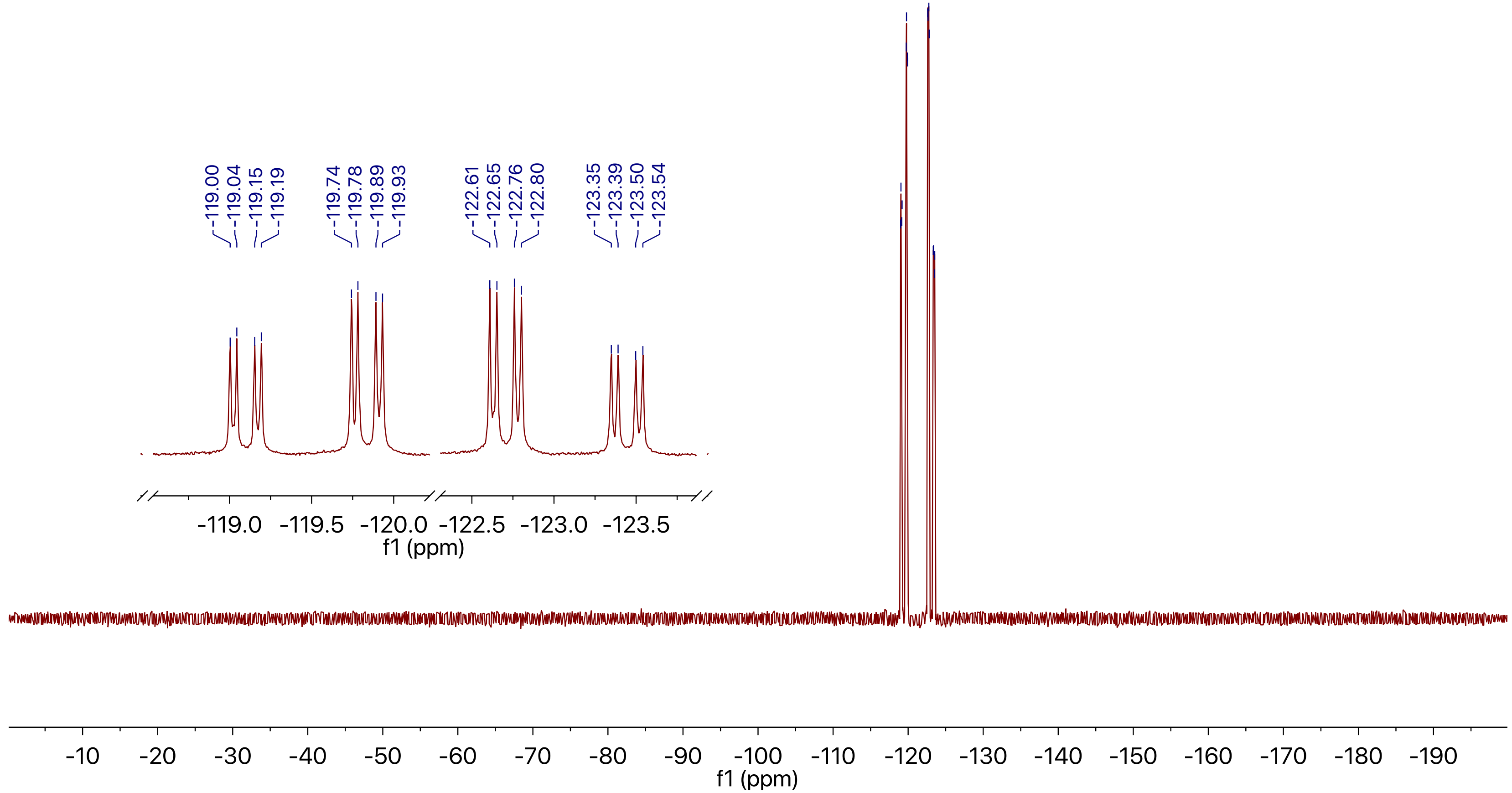

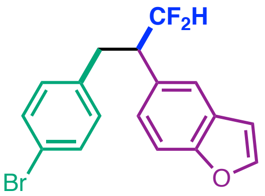

62

^{1}H NMR (400 MHz, $CDCl_3$)

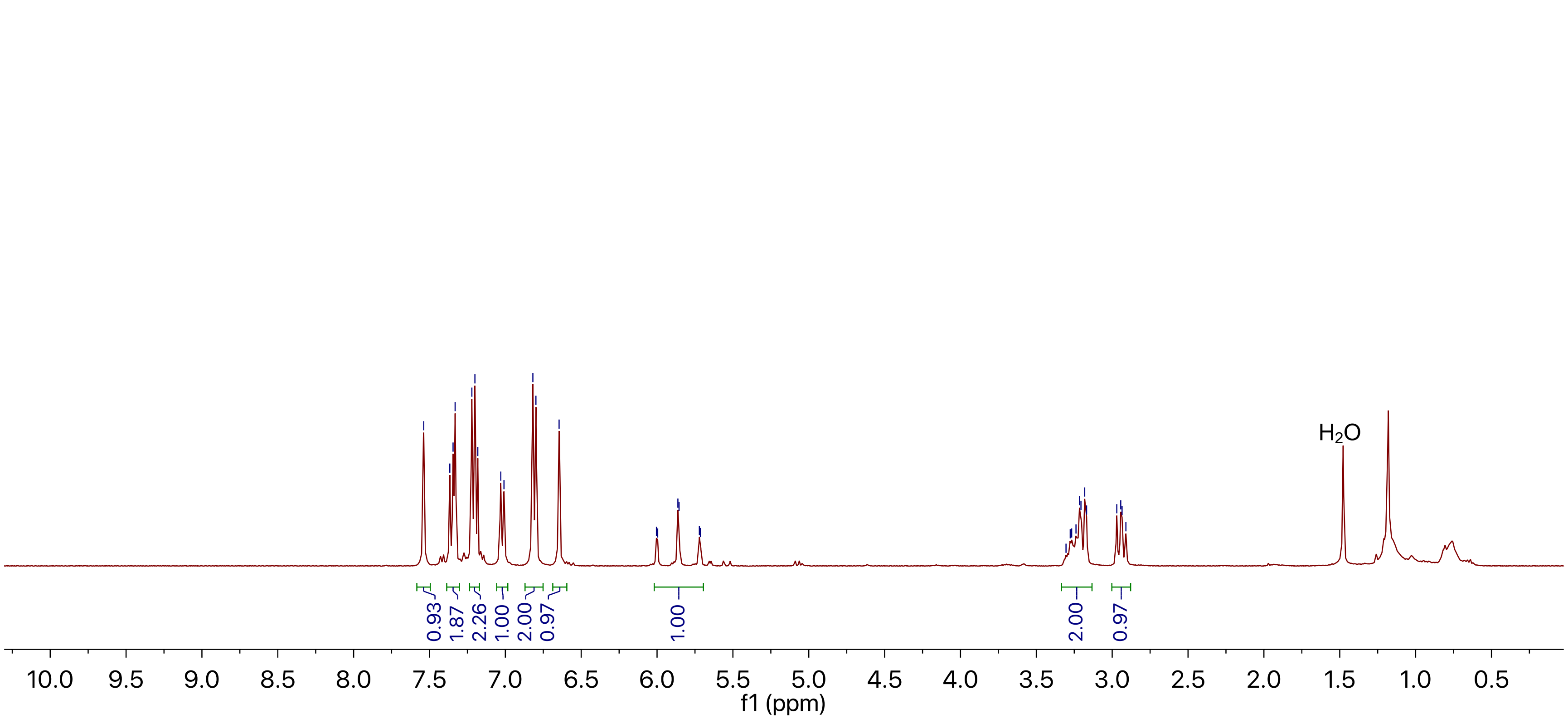

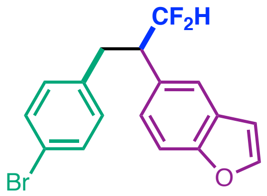

62

^{13}C NMR (101 MHz, $CDCl_3$)

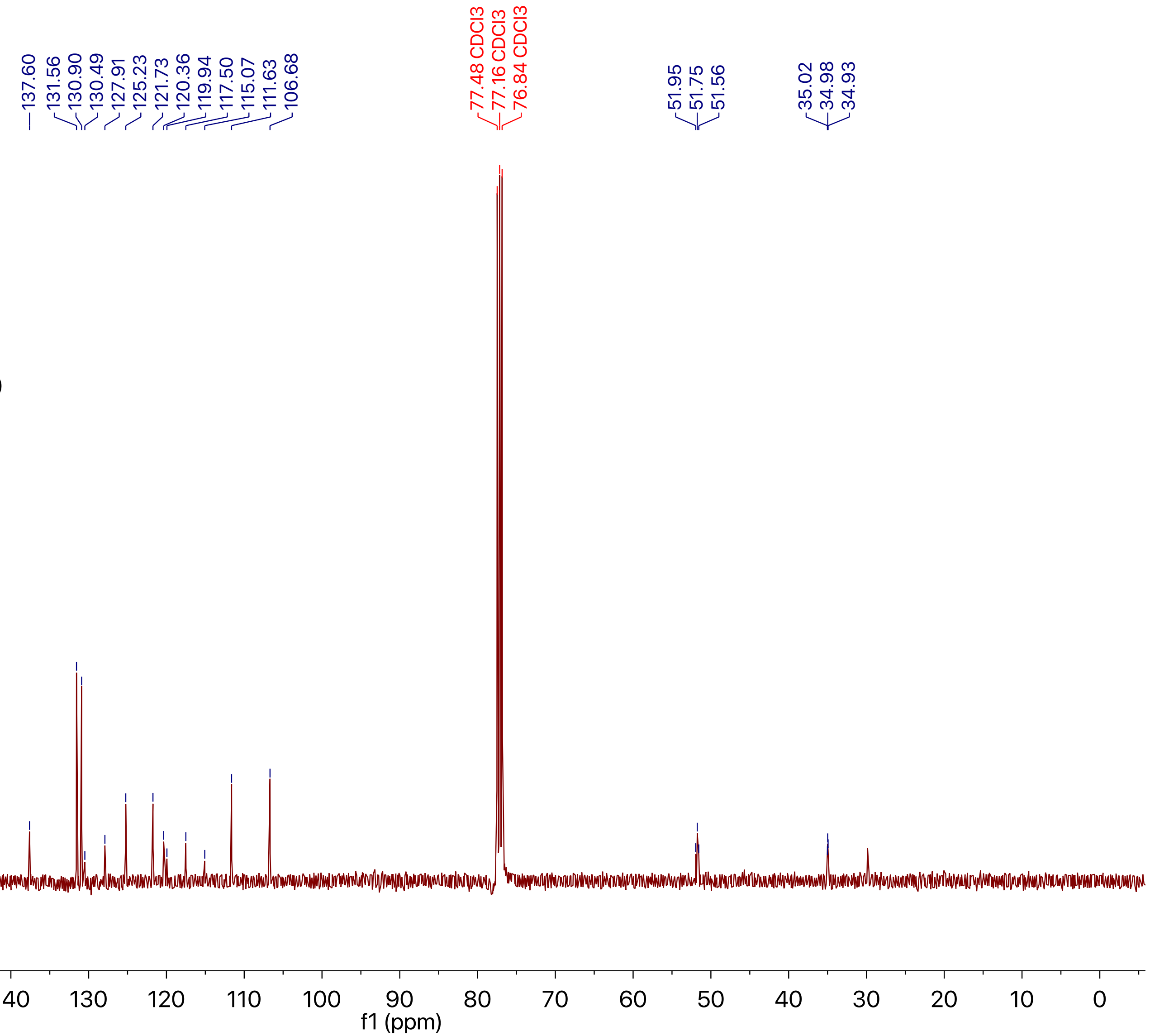

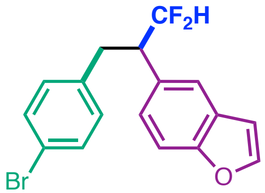

62

^{19}F NMR (376 MHz, $CDCl_3$)

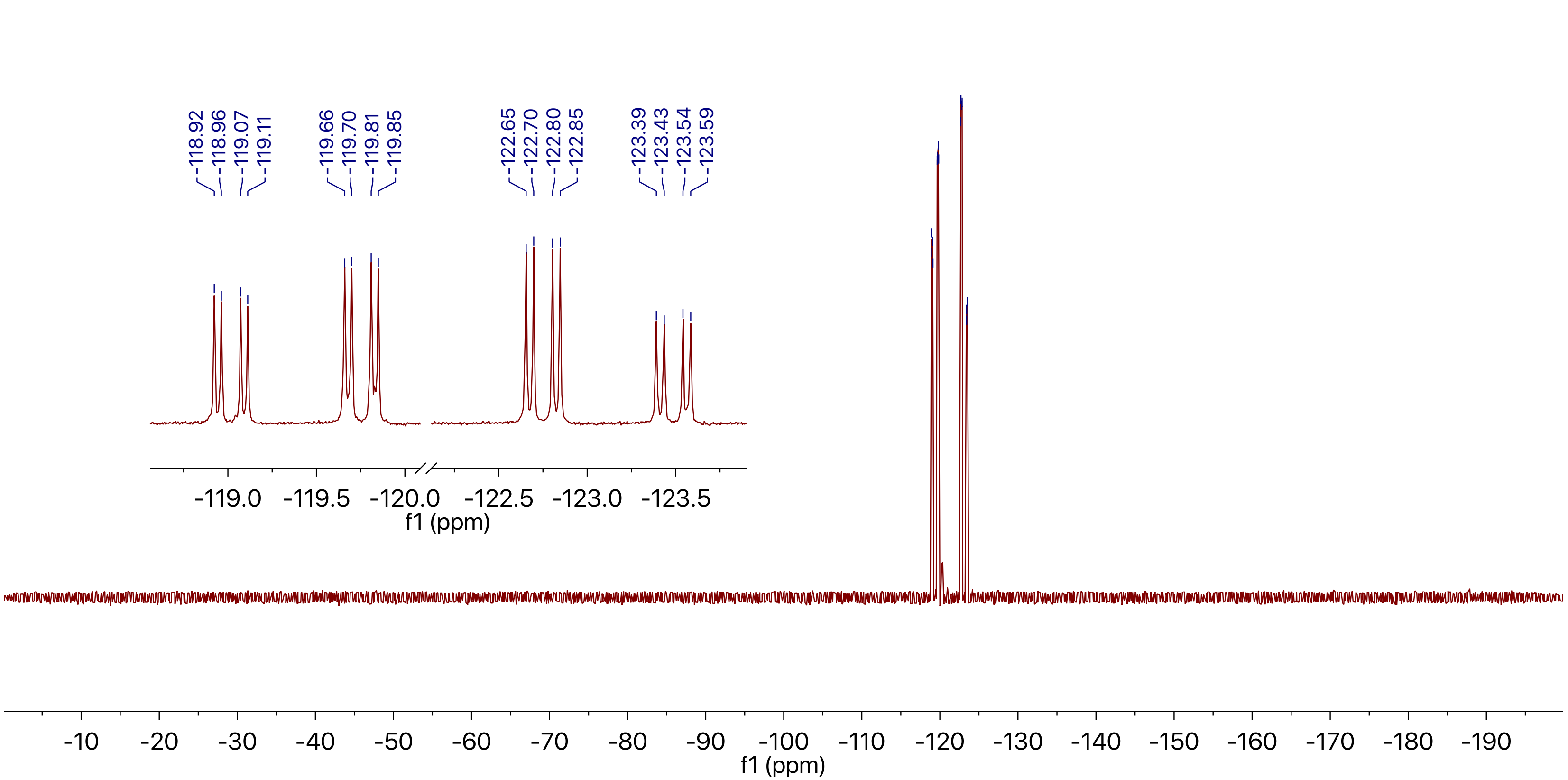

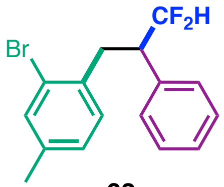

63

^1H NMR (400 MHz, $CDCl_3$)

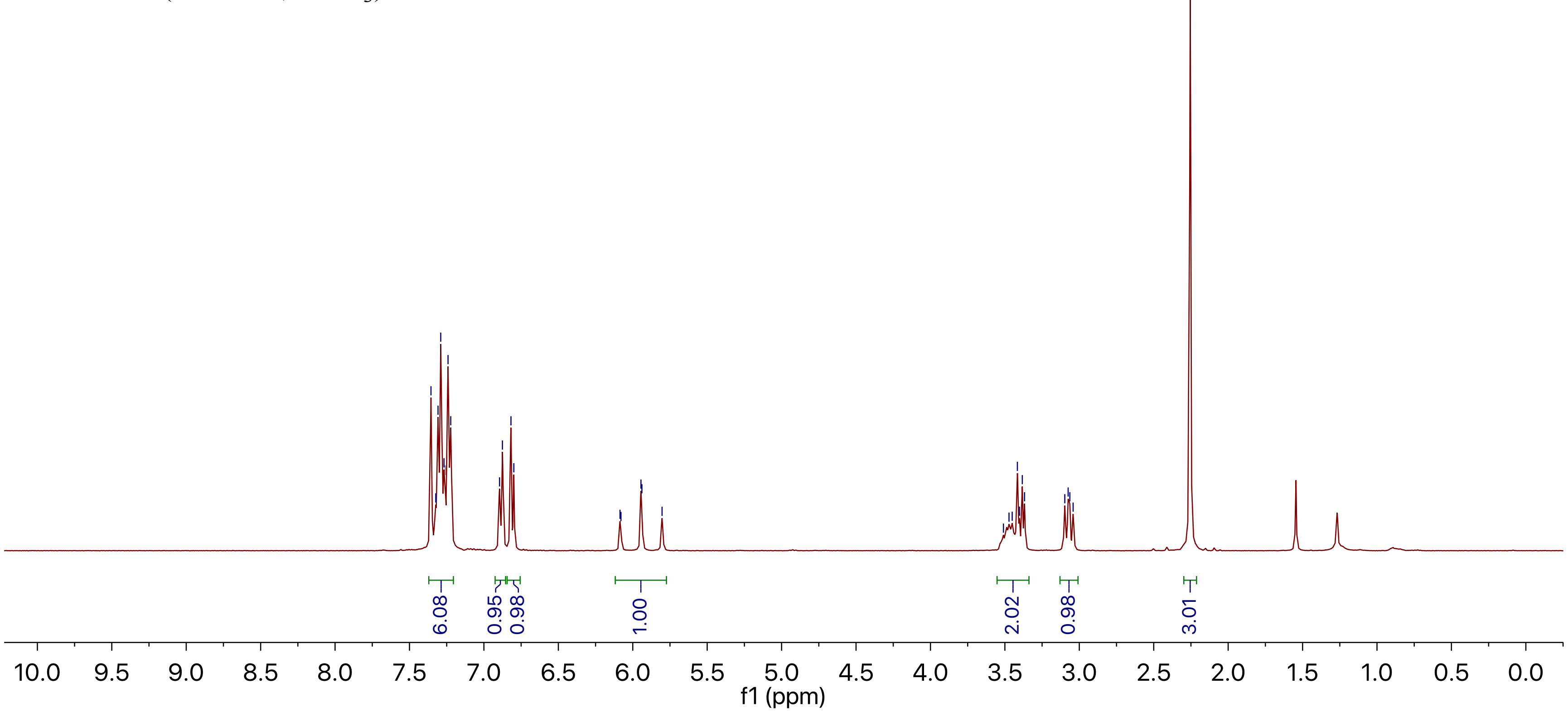

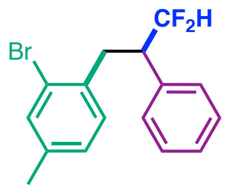

63

^{13}C NMR (101 MHz, $CDCl_3$)

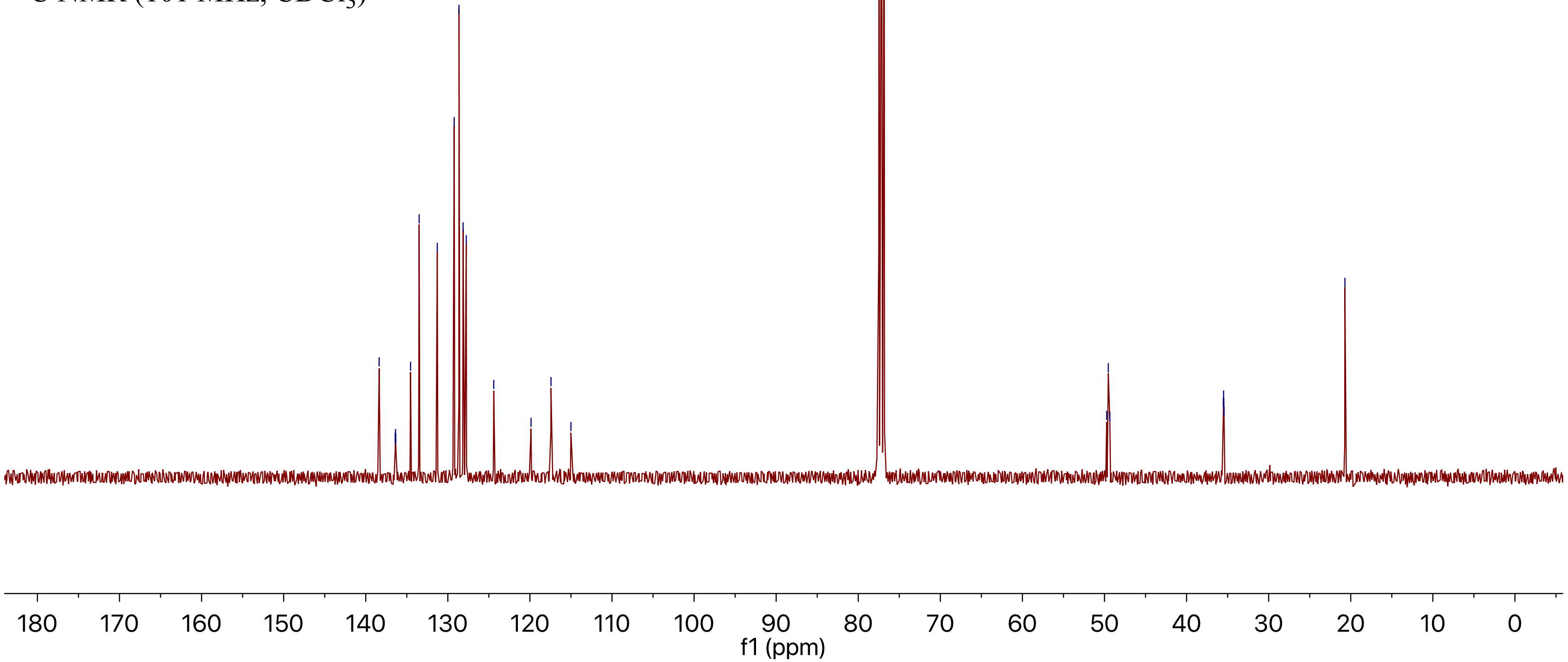

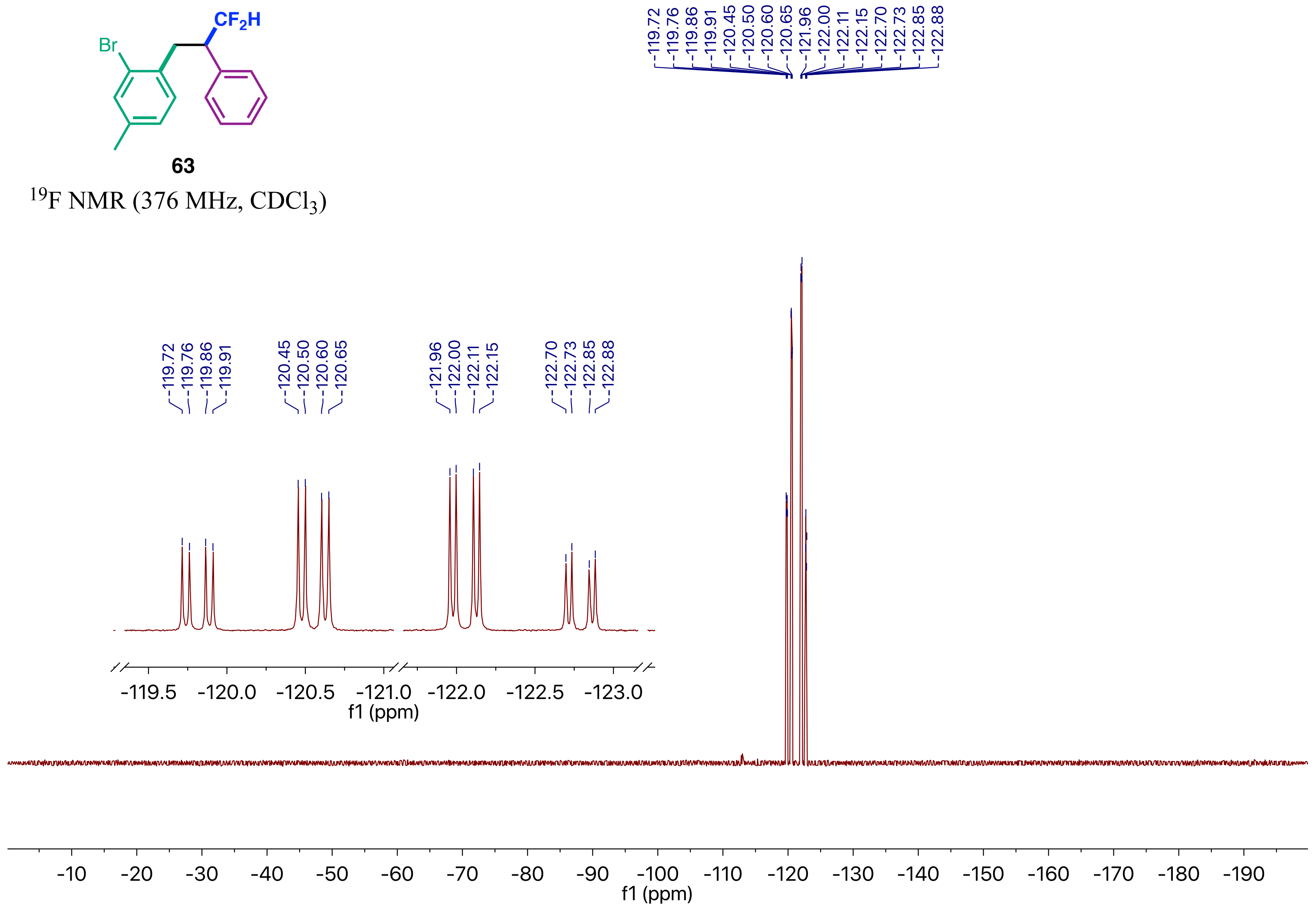
Br
CF2H
63
19F NMR (376 MHz, CDCl3)
-119.72
-119.76
-119.86
-119.91
-120.45
-120.50
-120.60
-120.65
-121.96
-122.00
-122.11
-122.15
-122.70
-122.73
-122.85
-122.88
-119.5 -120.0 -120.5 -121.0 -122.0 -122.5 -123.0
f1 (ppm)
-10 -20 -30 -40 -50 -60 -70 -80 -90 -100 -110 -120 -130 -140 -150 -160 -170 -180 -190
f1 (ppm)

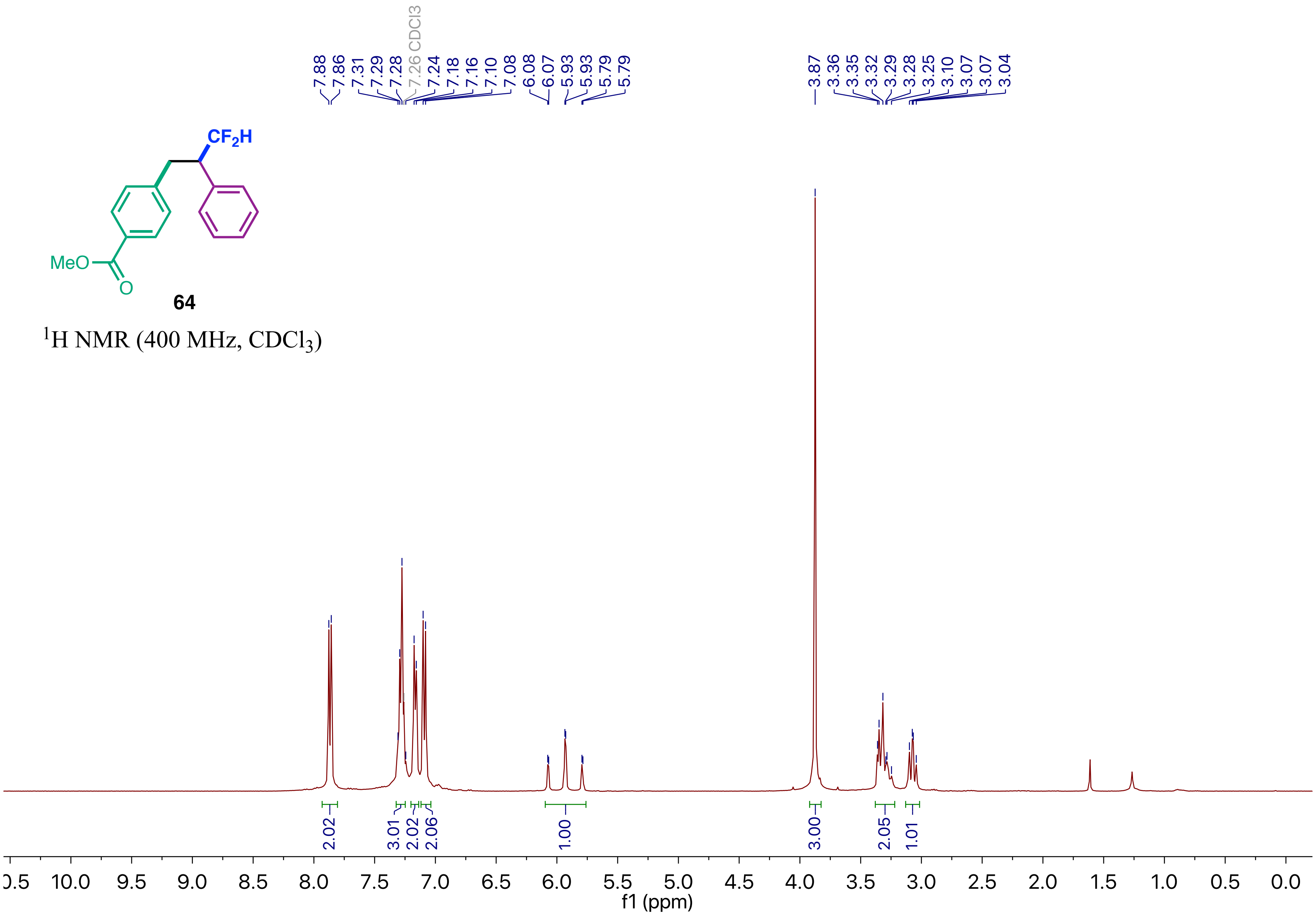

CF2H
MeO
O
64
1H NMR (400 MHz, CDCl3)
7.88
7.86
7.31
7.29
7.28
7.26 CDCl3
7.24
7.18
7.16
7.10
7.08
6.08
6.07
5.93
5.93
5.79
5.79
3.87
3.36
3.35
3.32
3.29
3.28
3.25
3.10
3.07
3.07
3.04
2.02
3.01
2.02
2.06
1.00
3.00
2.05
1.01
f1 (ppm)

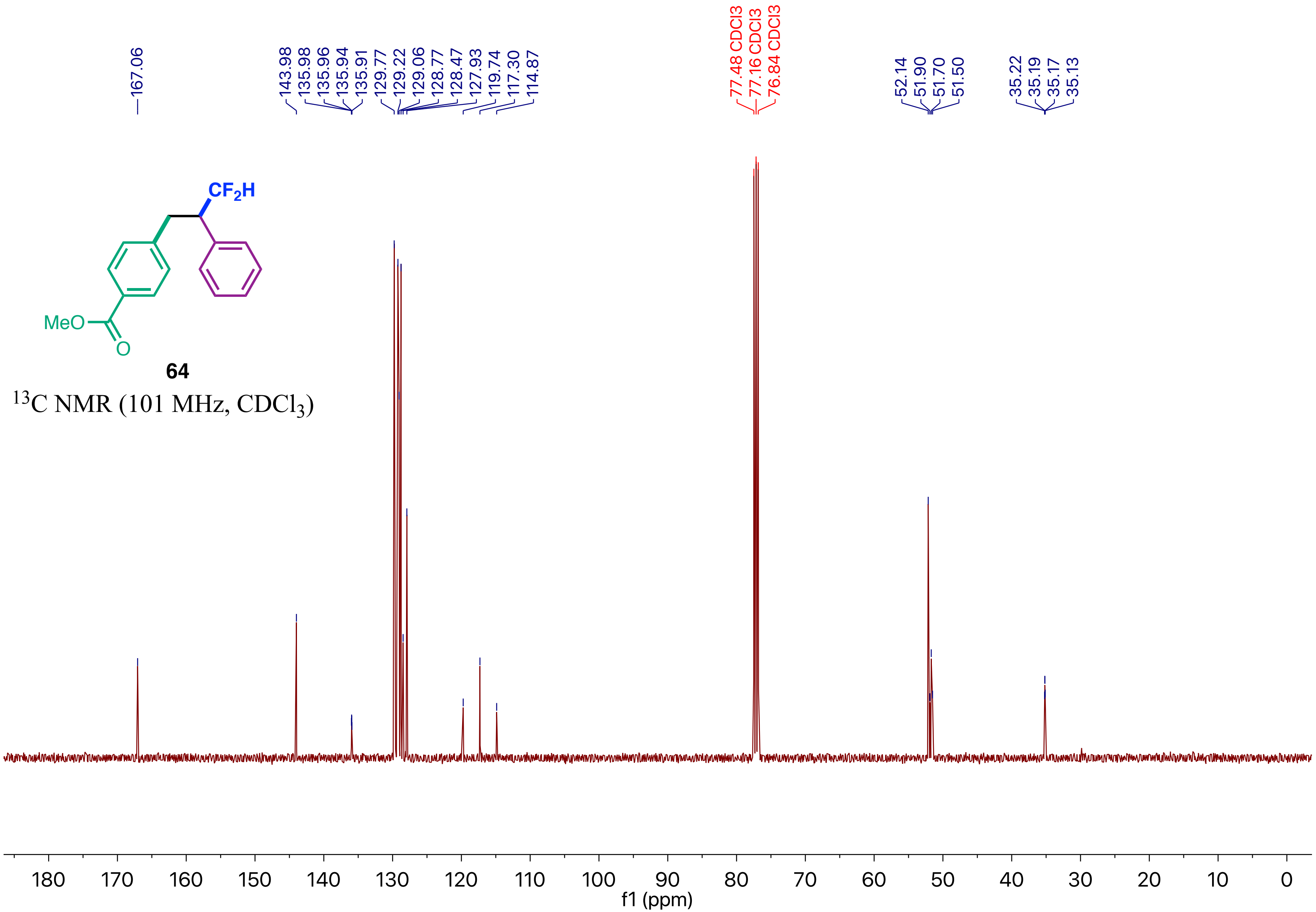

64

^{13}C NMR (101 MHz, $CDCl_3$)

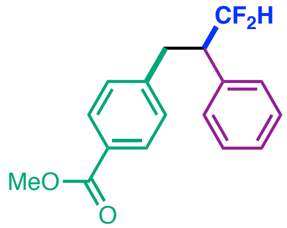

64

^{19}F NMR (376 MHz, $CDCl_3$)

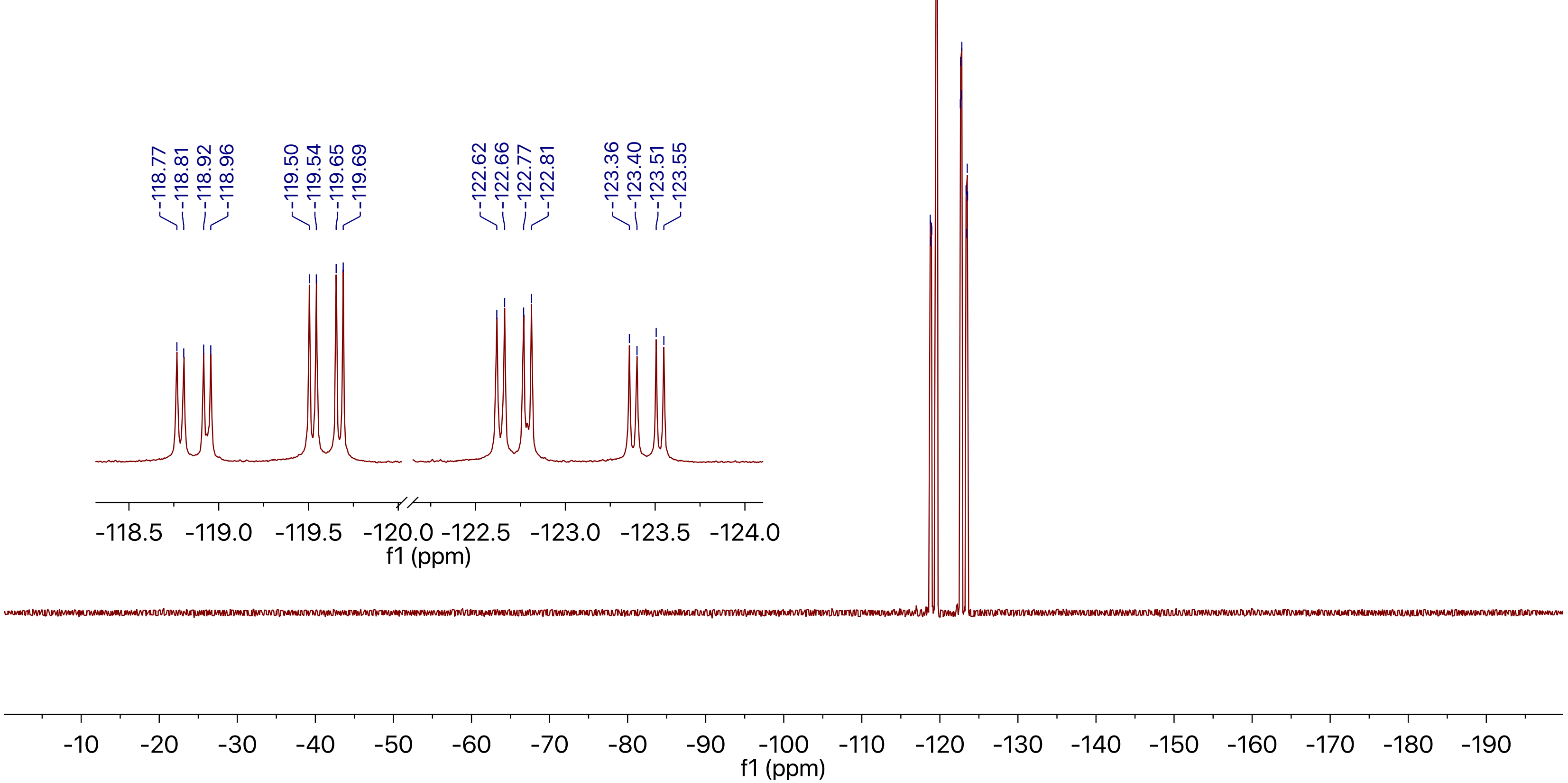

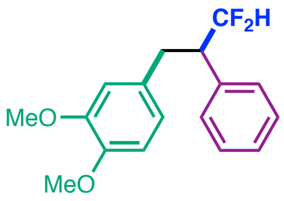

65

^{1}H NMR (400 MHz, $CDCl_3$)

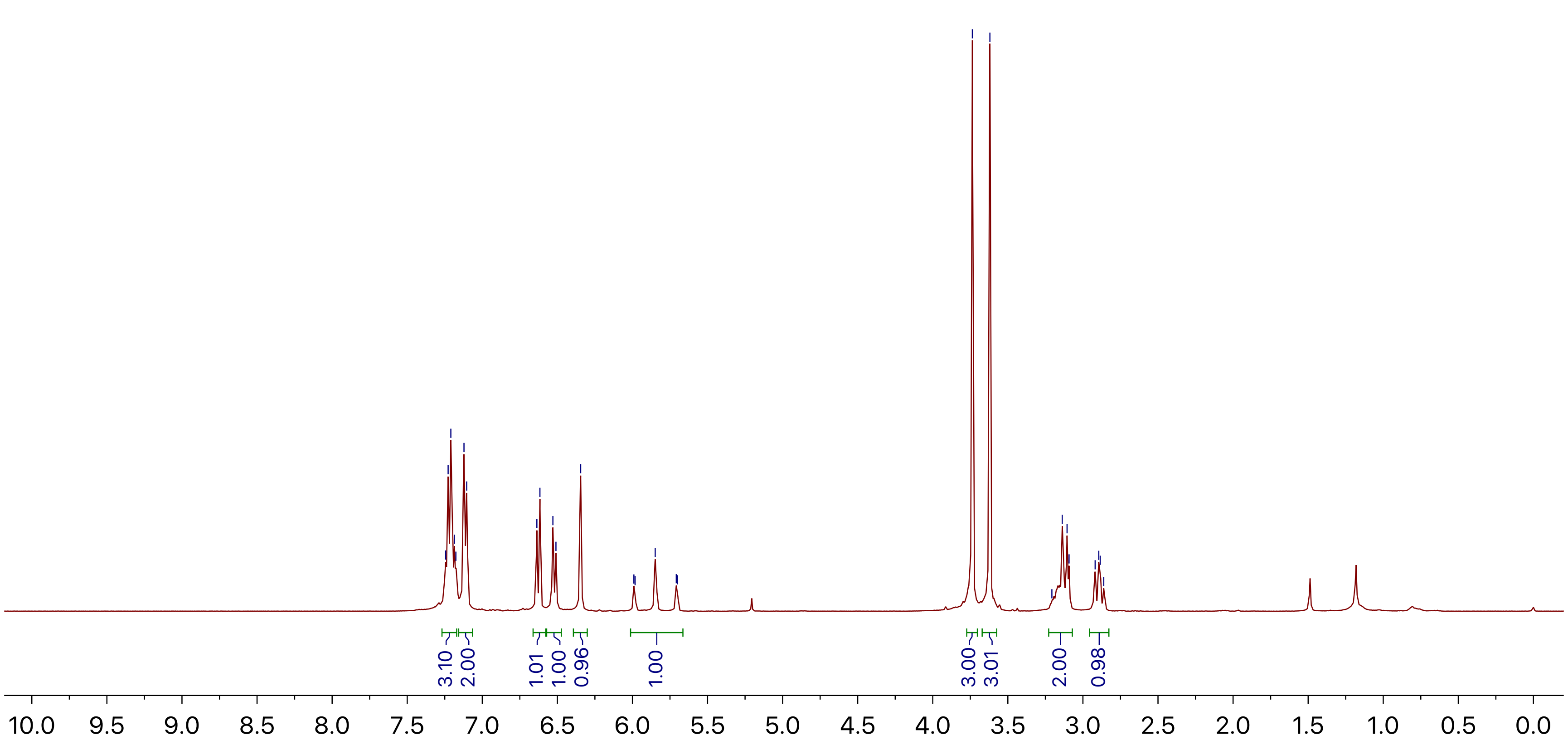

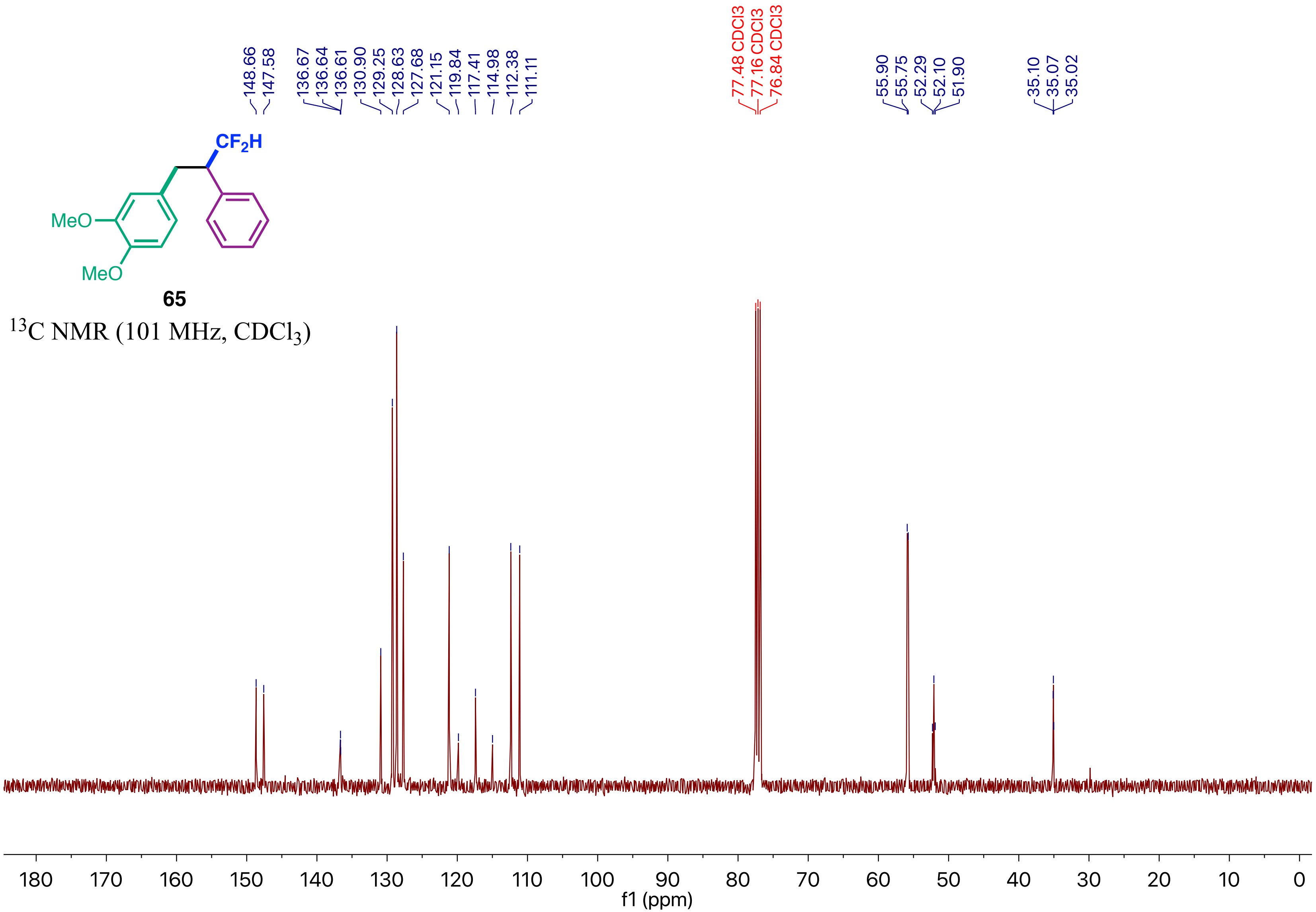
148.66
147.58
136.67
136.64
136.61
130.90
129.25
128.63
127.68
121.15
119.84
117.41
114.98
112.38
111.11
77.48 CDCl3
77.16 CDCl3
76.84 CDCl3
55.90
55.75
52.29
52.10
51.90
35.10
35.07
35.02
CF2H
MeO
MeO
65
13C NMR (101 MHz, CDCl3)
180 170 160 150 140 130 120 110 100 90 80 70 60 50 40 30 20 10 0
f1 (ppm)

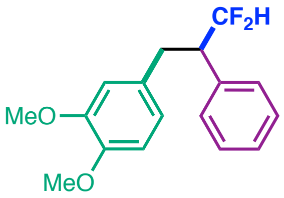

65

^{19}F NMR (376 MHz, $CDCl_3$)

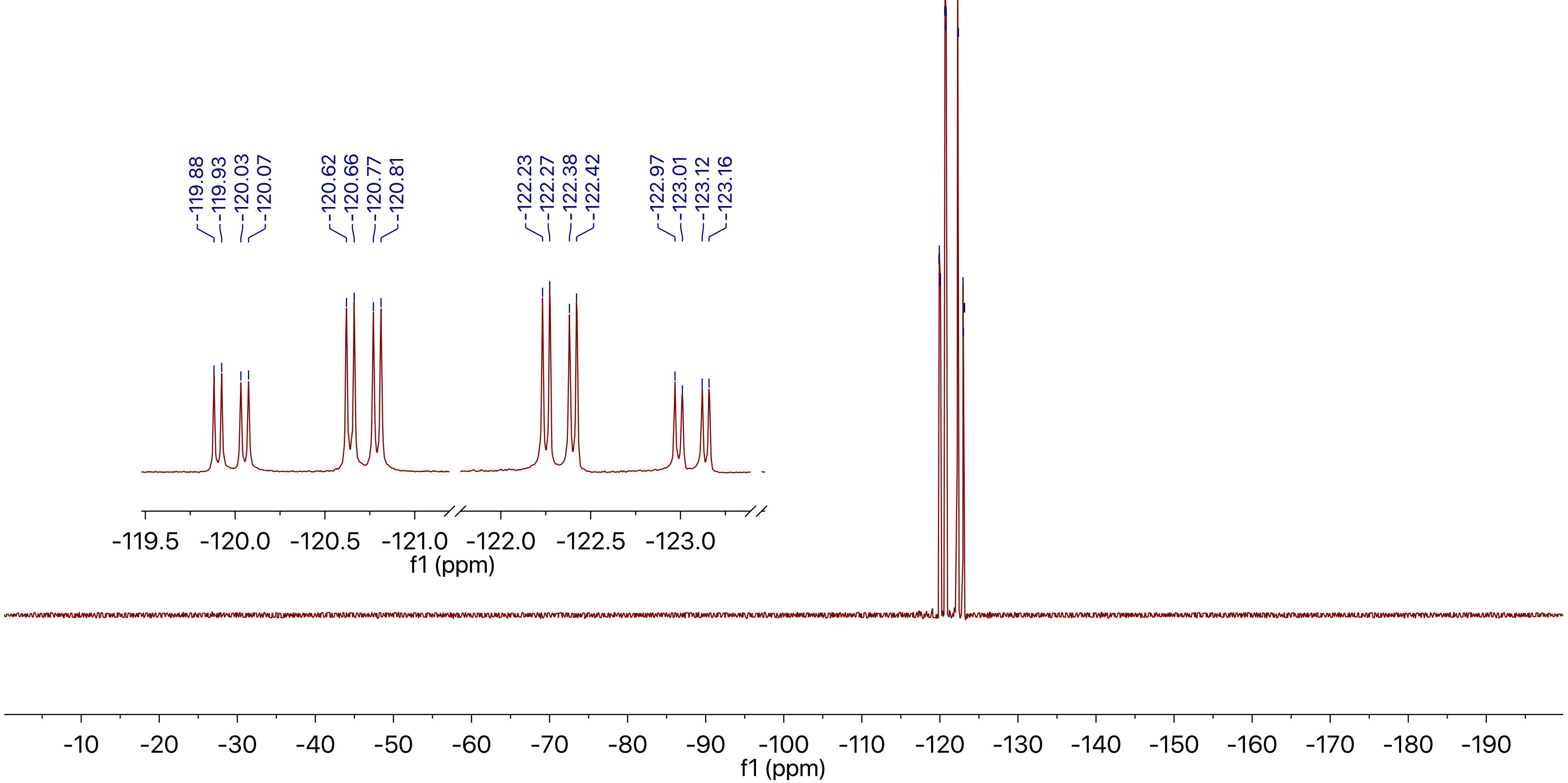

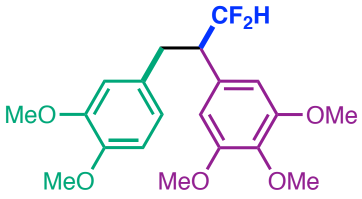

66

^1H NMR (400 MHz, $CDCl_3$)

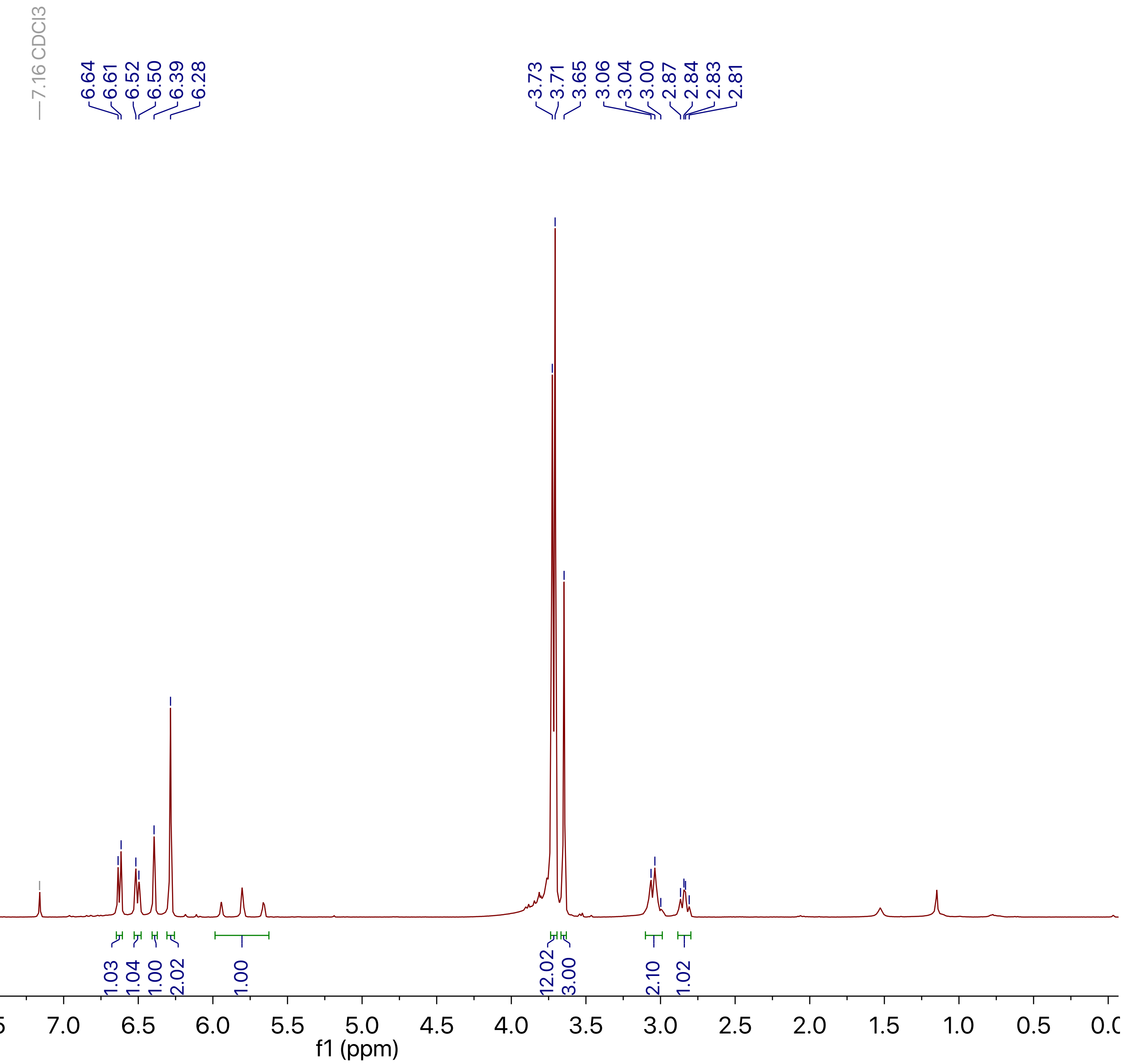

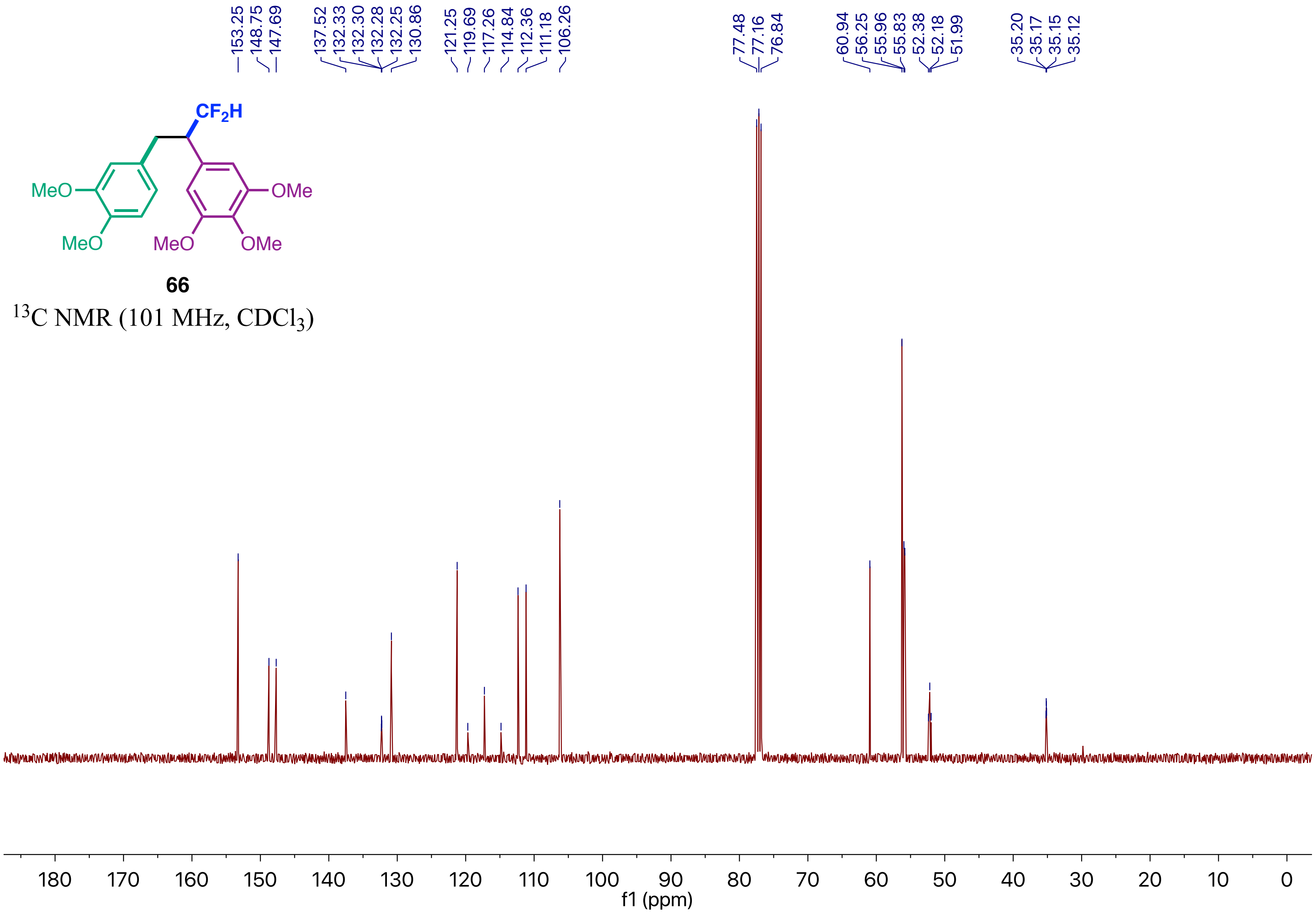
153.25
148.75
147.69
137.52
132.33
132.30
132.28
132.25
130.86
121.25
119.69
117.26
114.84
112.36
111.18
106.26
77.48
77.16
76.84
60.94
56.25
55.96
55.83
52.38
52.18
51.99
35.20
35.17
35.15
35.12
CF2H
MeO
MeO
MeO
OMe
OMe
66
13C NMR (101 MHz, CDCl3)
180 170 160 150 140 130 120 110 100 90 80 70 60 50 40 30 20 10 0
f1 (ppm)

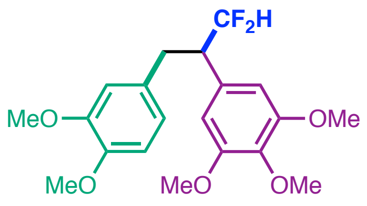

66

^{19}F NMR (376 MHz, $CDCl_3$)

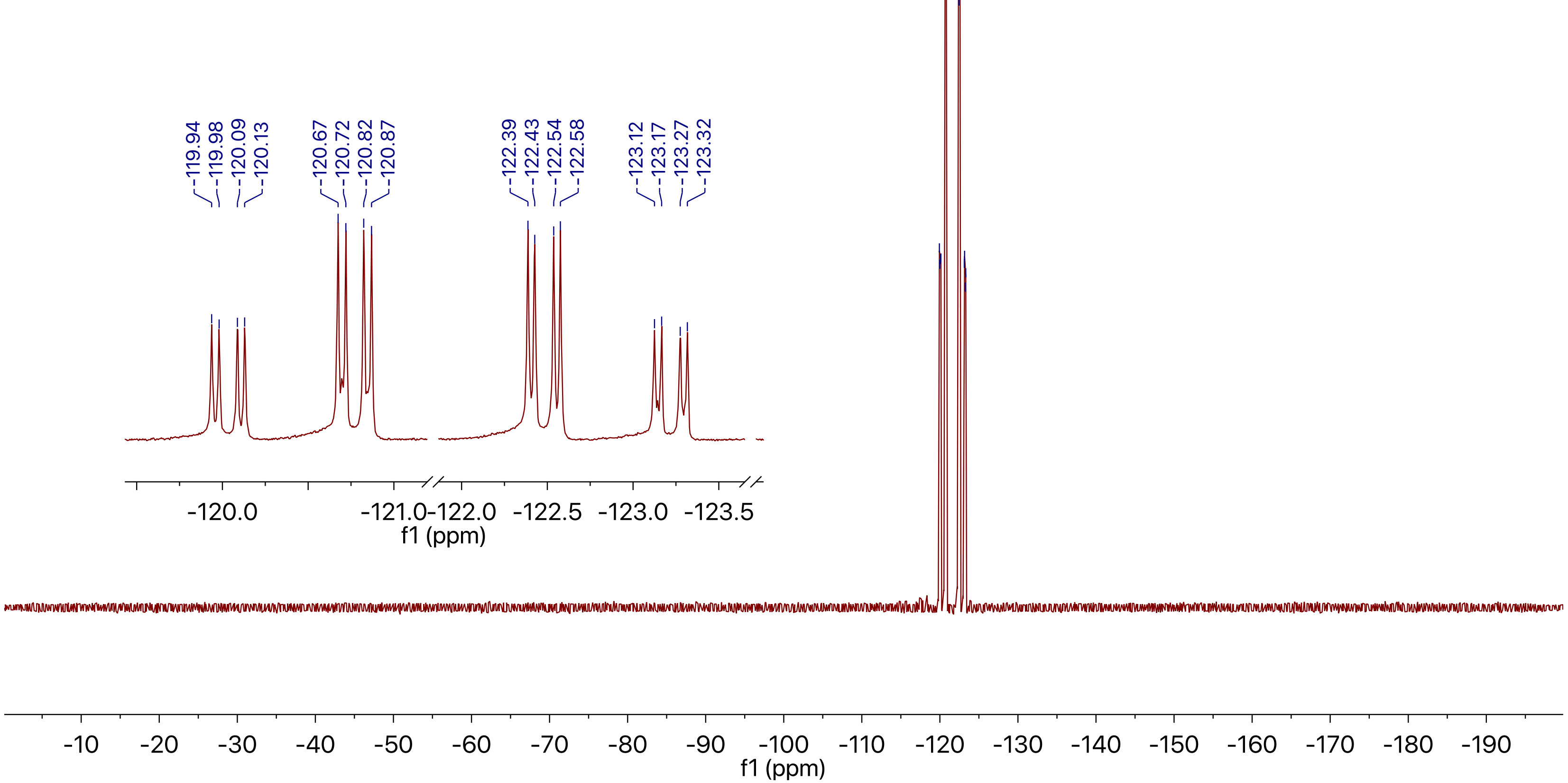

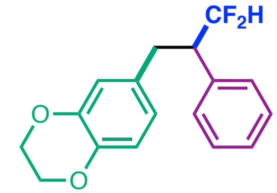

67

^{1}H NMR (400 MHz, $CDCl_3$)

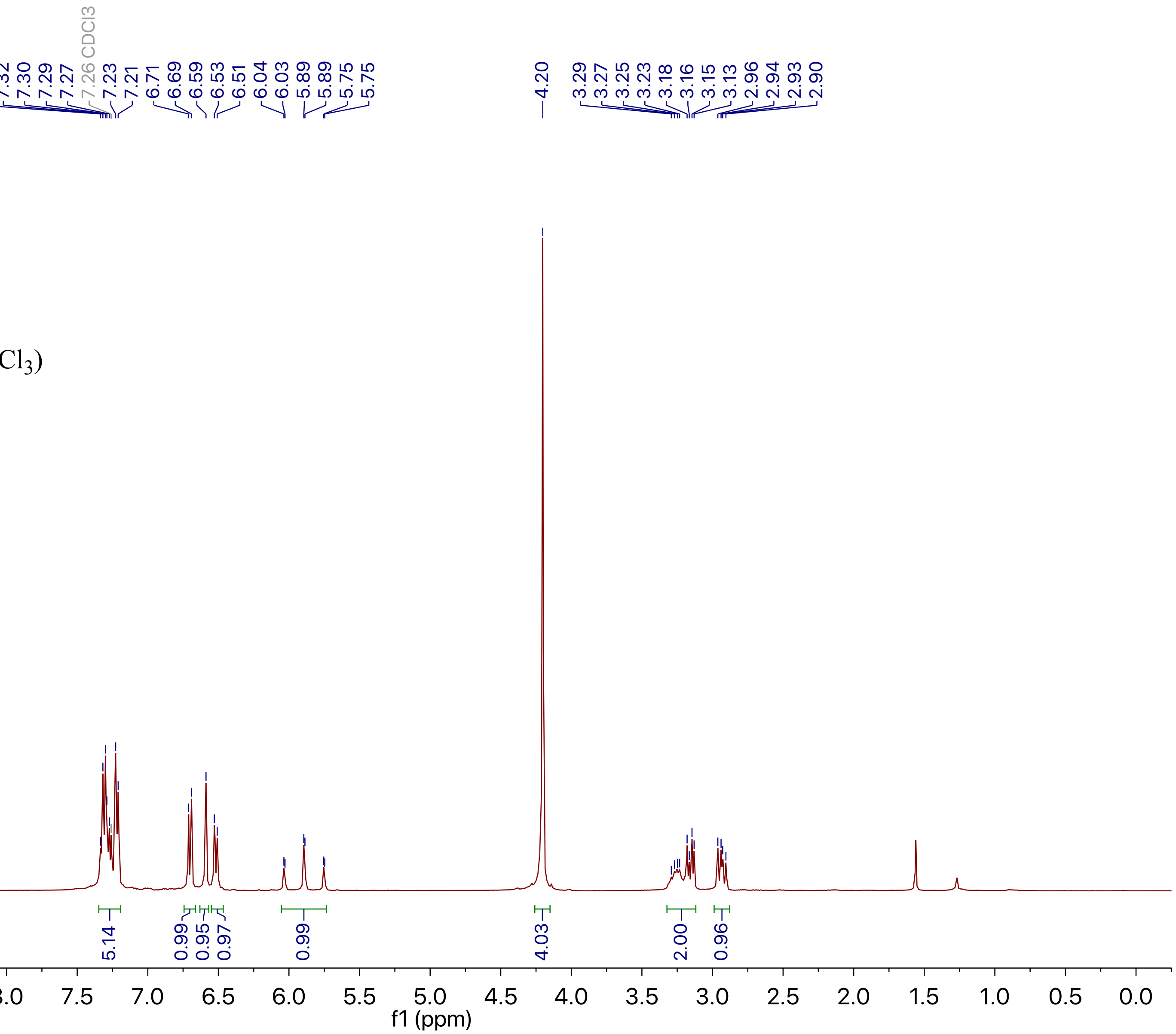

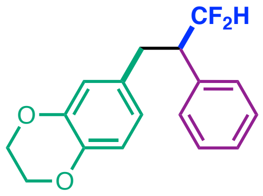

67

^{13}C NMR (101 MHz, $CDCl_3$)

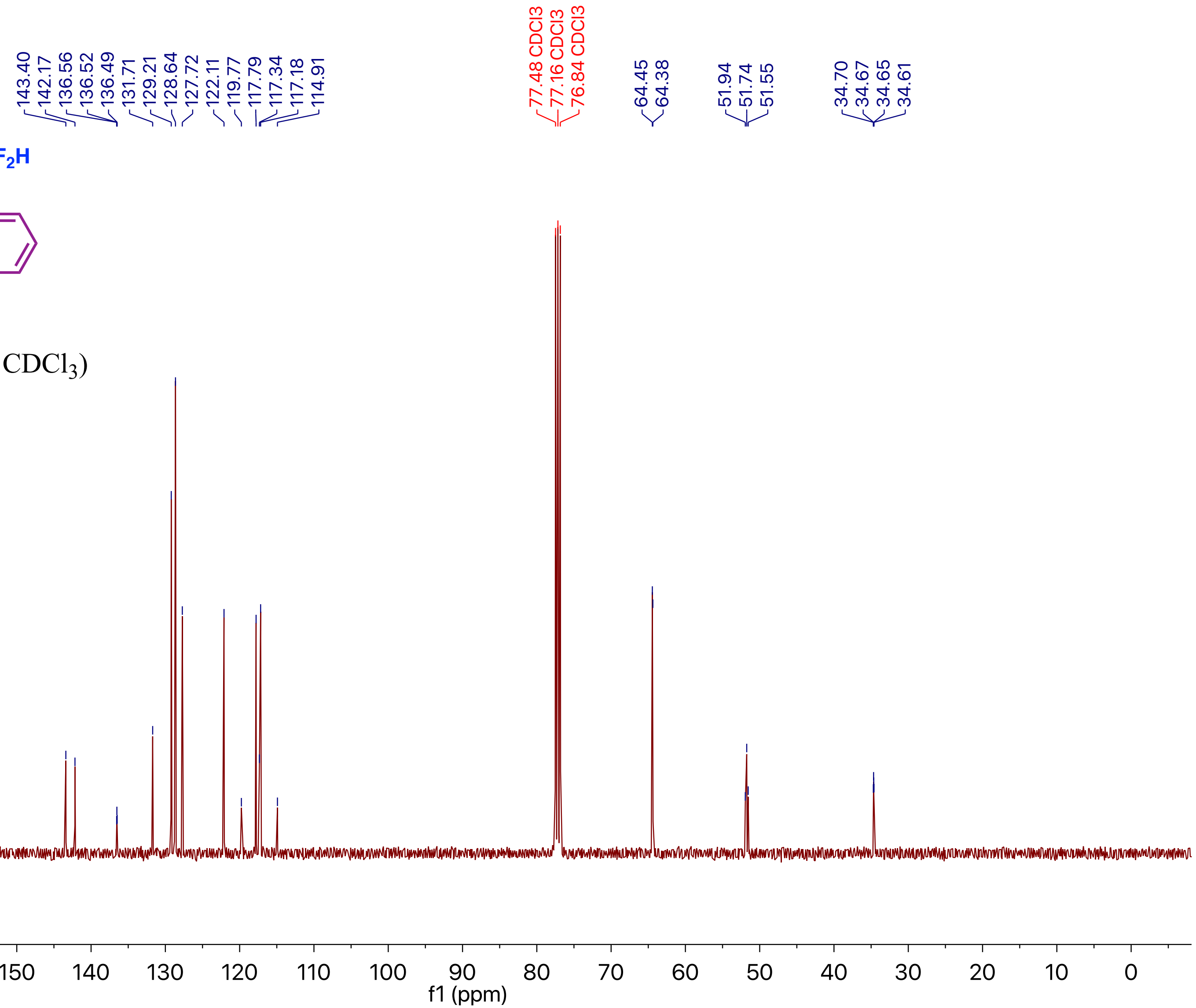

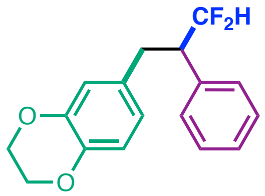

67

^{19}F NMR (376 MHz, $CDCl_3$)

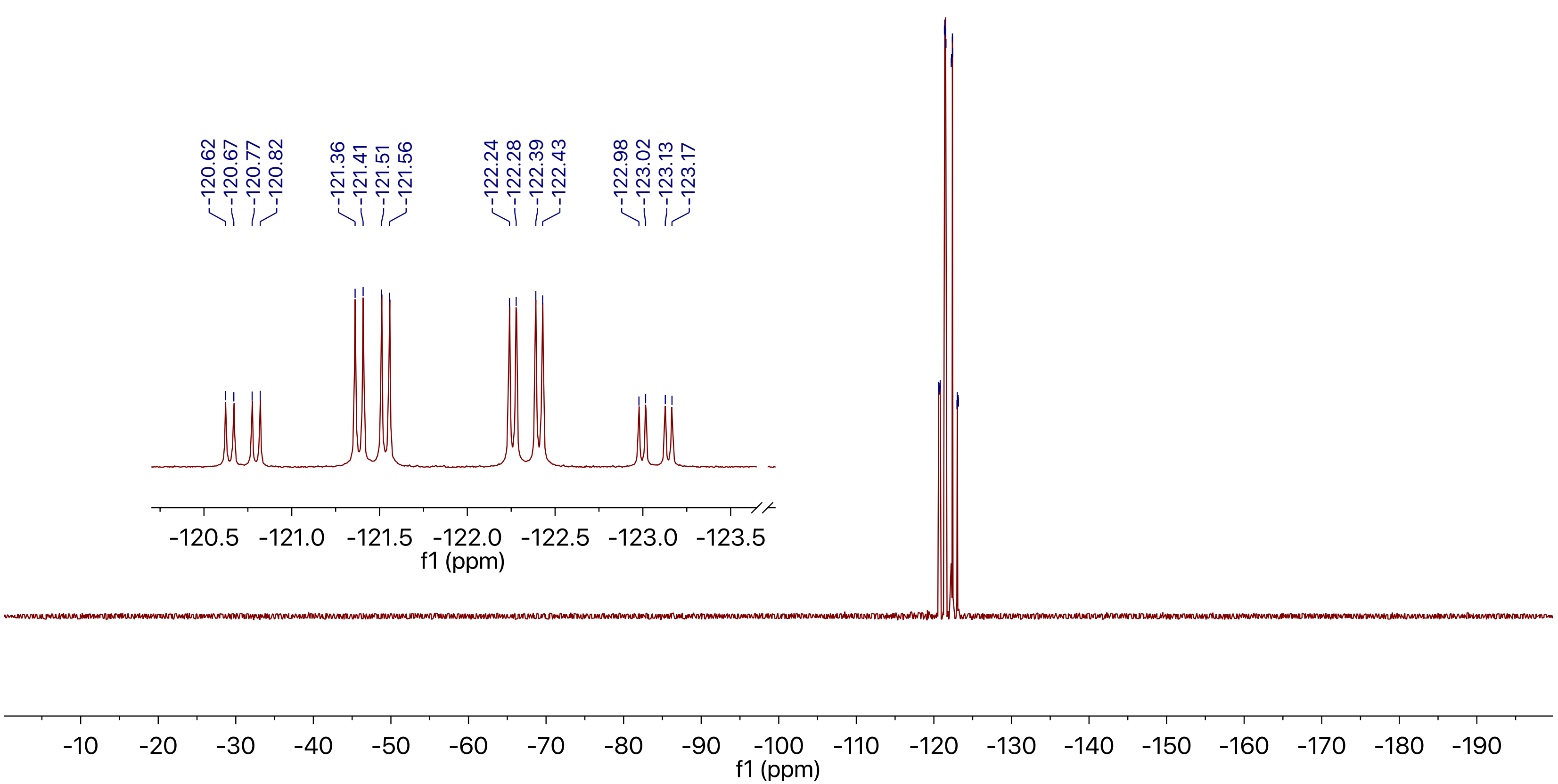

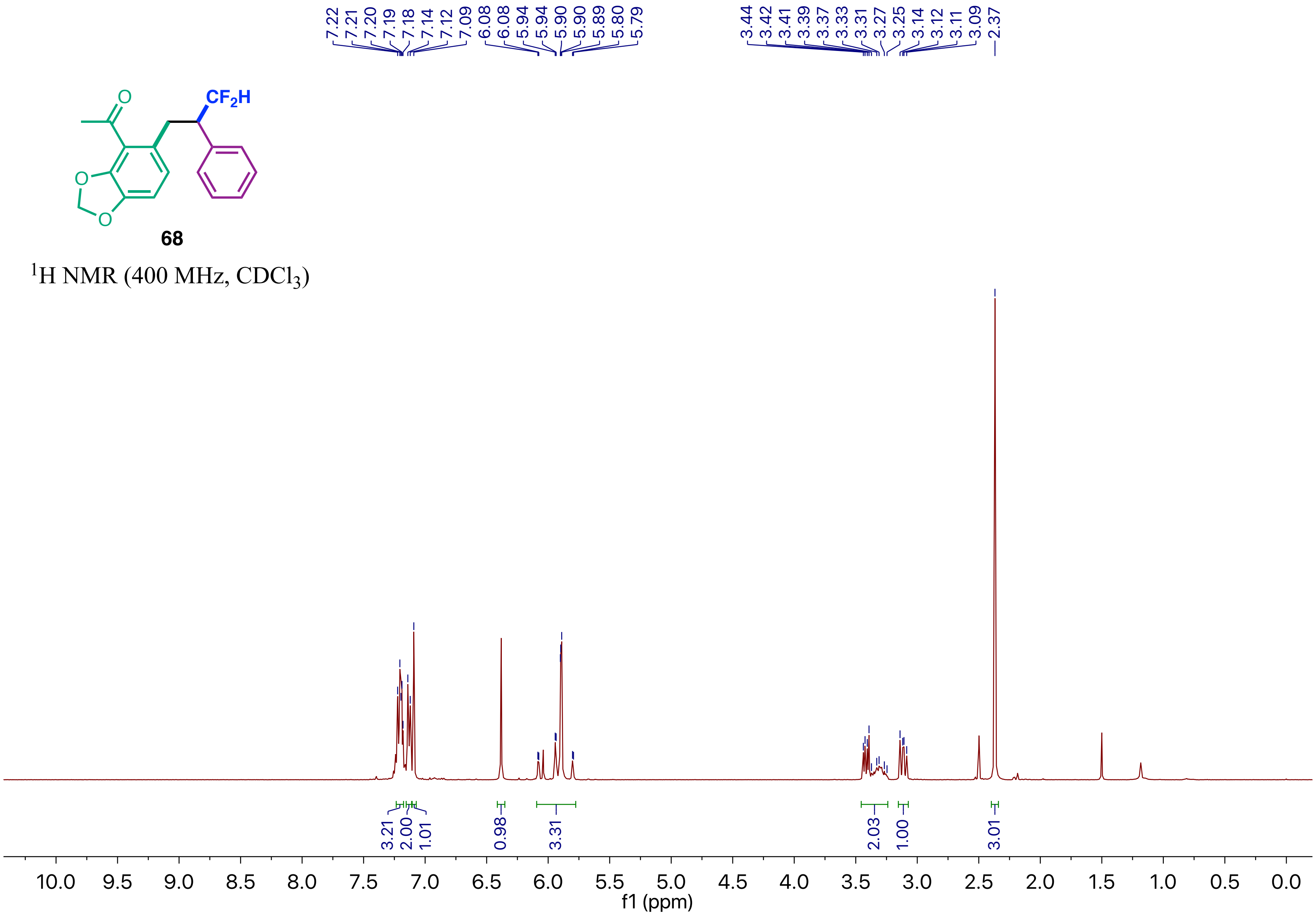
7.22
7.21
7.20
7.19
7.18
7.14
7.12
7.09
6.08
6.08
5.94
5.94
5.90
5.90
5.89
5.80
5.79
3.44
3.42
3.41
3.39
3.37
3.33
3.31
3.27
3.25
3.14
3.12
3.11
3.09
2.37
CF2H
68
1H NMR (400 MHz, CDCl3)
3.21
2.00
1.01
0.98
3.31
2.03
1.00
3.01
10.0 9.5 9.0 8.5 8.0 7.5 7.0 6.5 6.0 5.5 5.0 4.5 4.0 3.5 3.0 2.5 2.0 1.5 1.0 0.5 0.0
f1 (ppm)

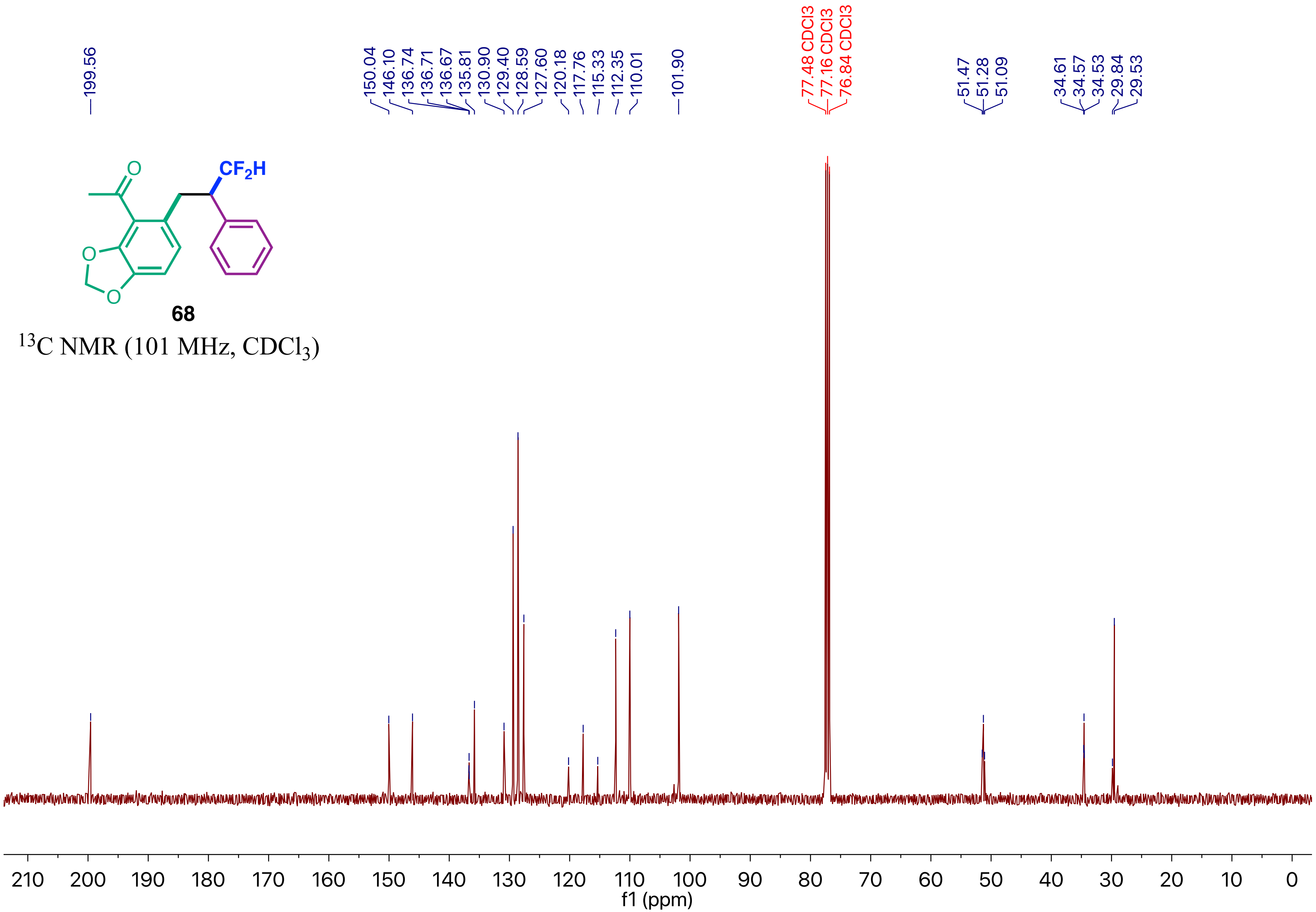

^{13}C NMR (101 MHz, $CDCl_3$)

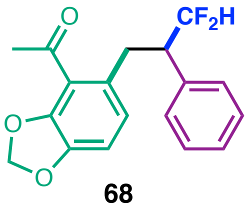

68

^{19}F NMR (376 MHz, $CDCl_3$)

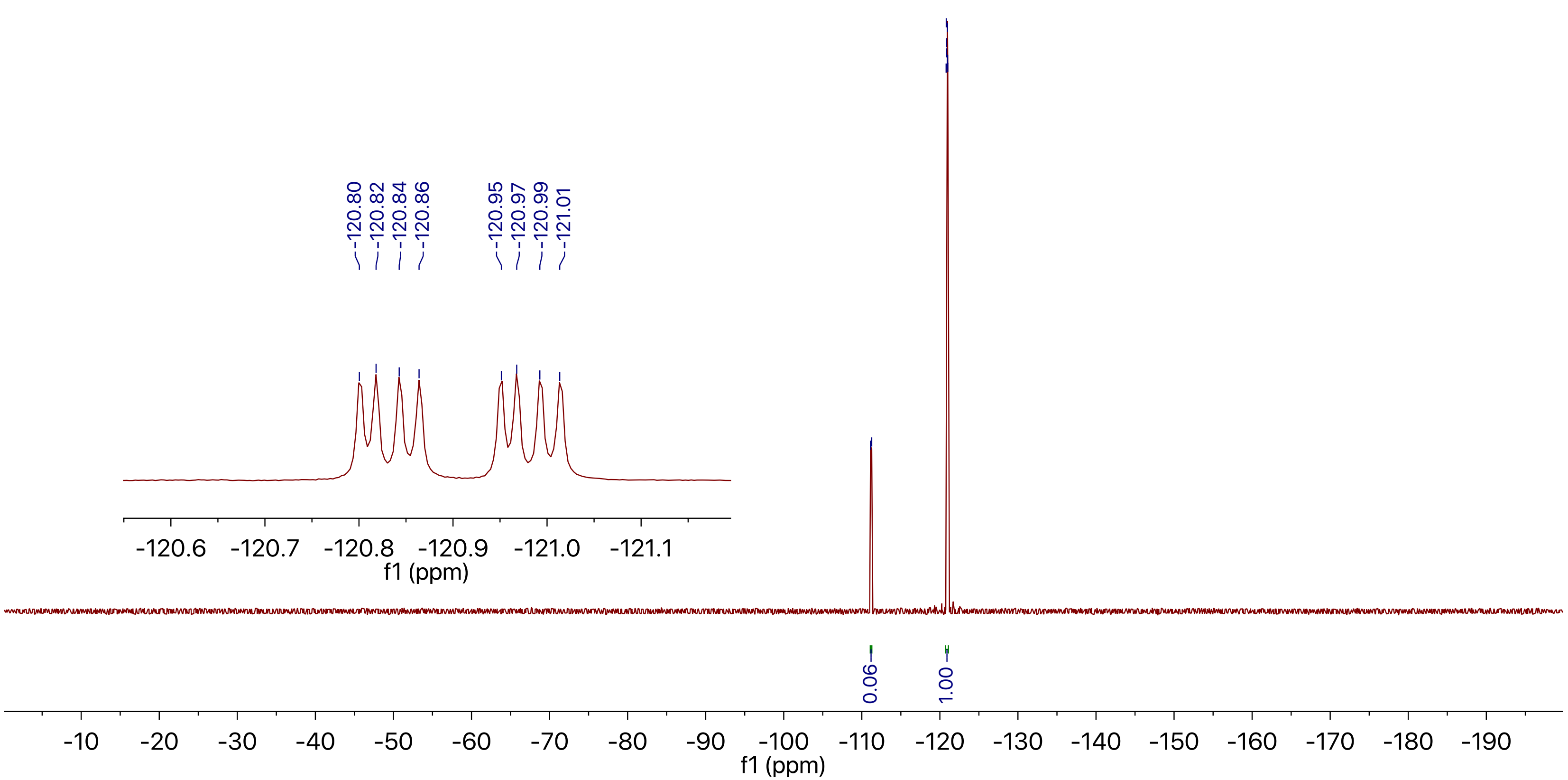

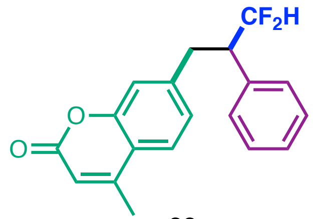

69

^{1}H NMR (400 MHz, $CDCl_3$)

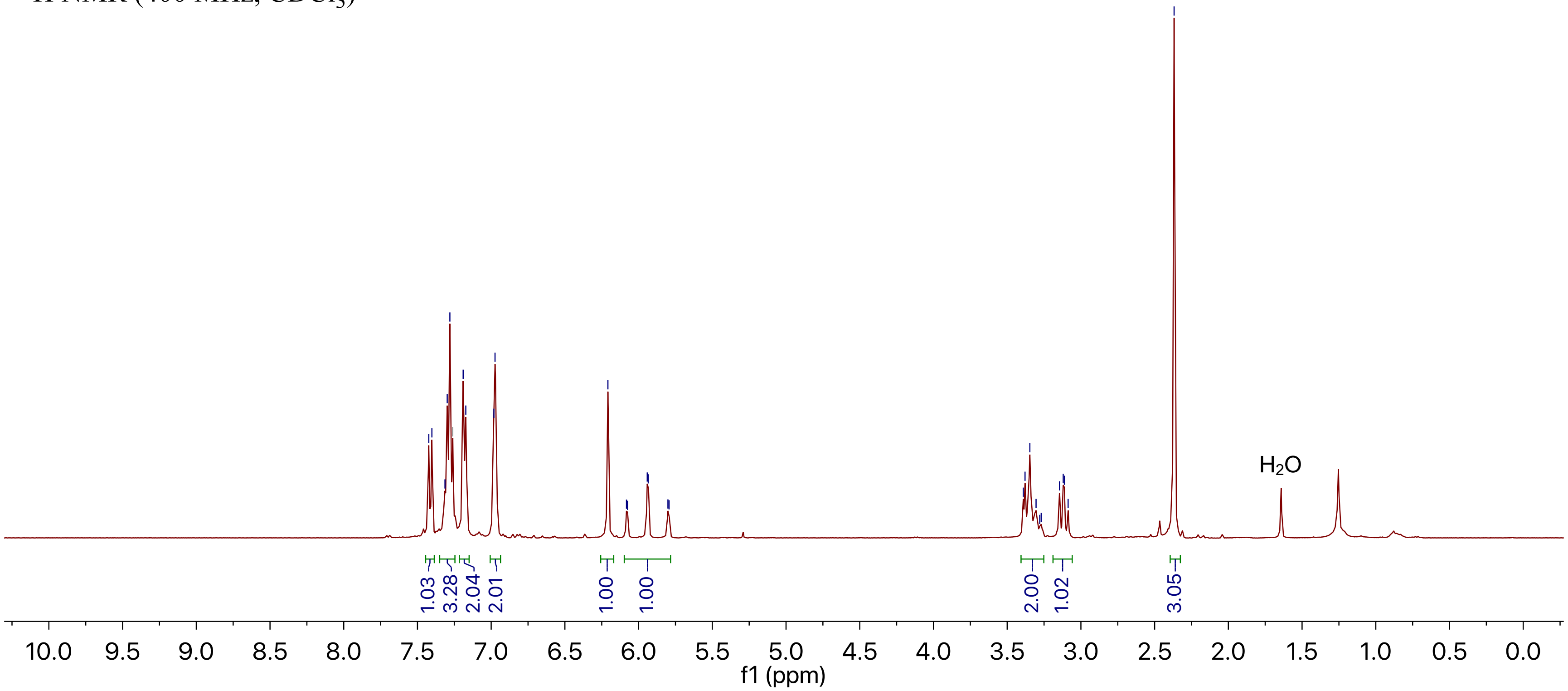

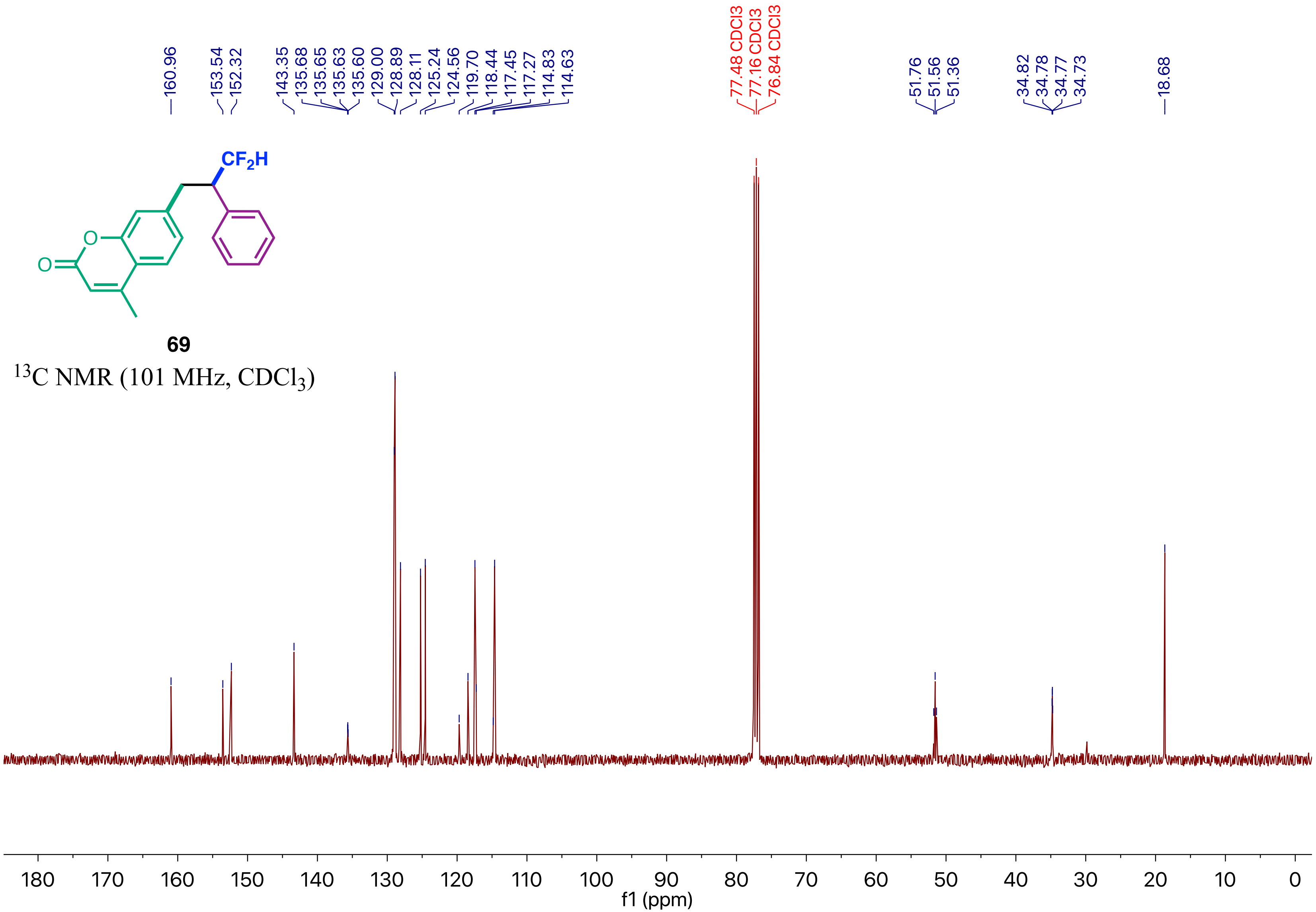

160.96
153.54
152.32
143.35
135.68
135.65
135.63
135.60
129.00
128.89
128.11
125.24
124.56
119.70
118.44
117.45
117.27
114.83
114.63
77.48 CDCl3
77.16 CDCl3
76.84 CDCl3
51.76
51.56
51.36
34.82
34.78
34.77
34.73
18.68
CF2H
O
O
69
13C NMR (101 MHz, CDCl3)
f1 (ppm)

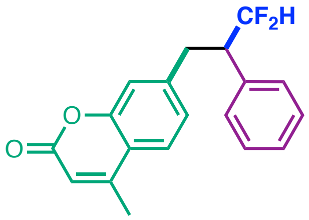

69

^{19}F NMR (376 MHz, $CDCl_3$)

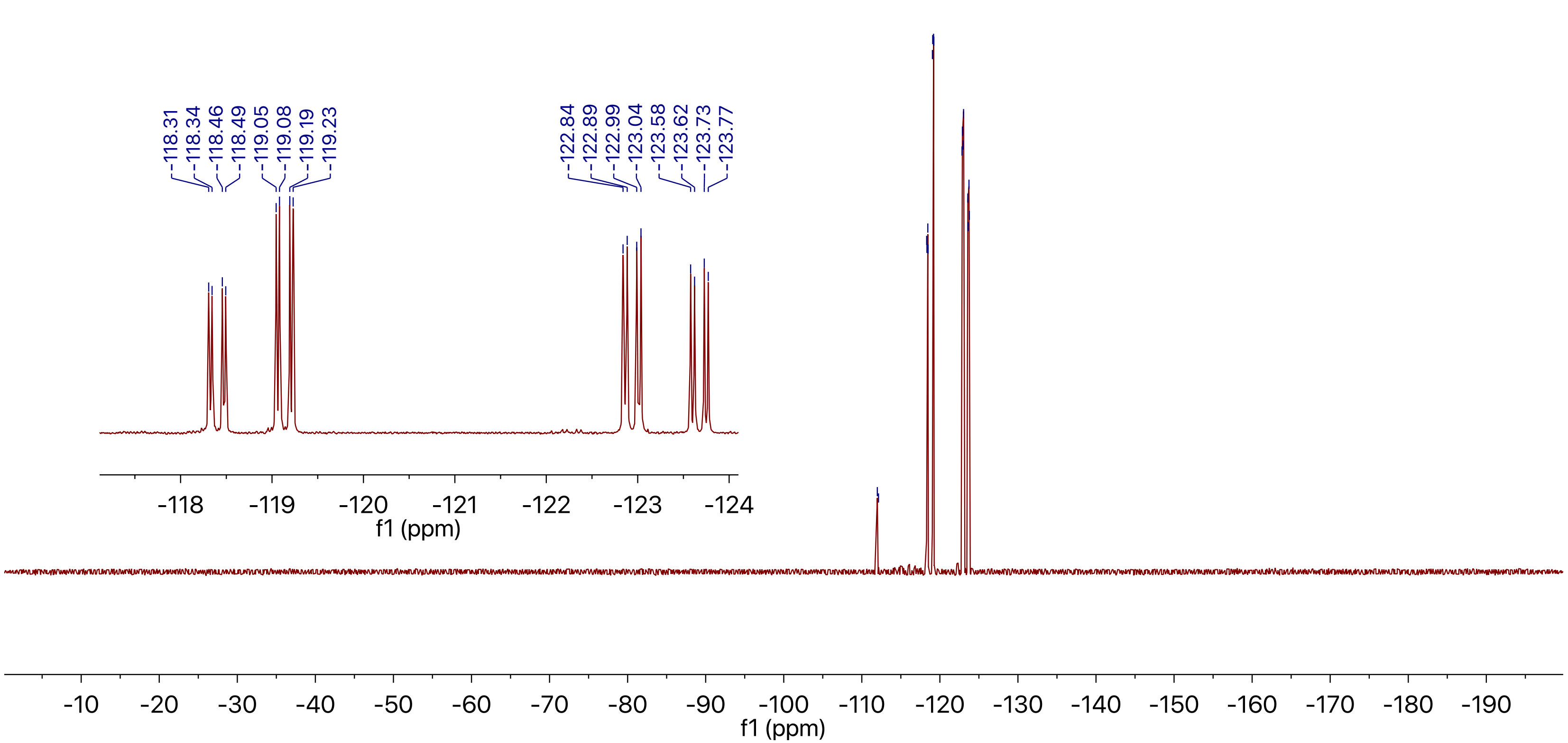

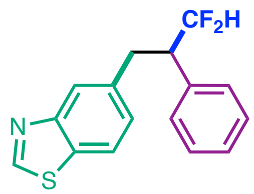

70

^{1}H NMR (400 MHz, $CDCl_3$)

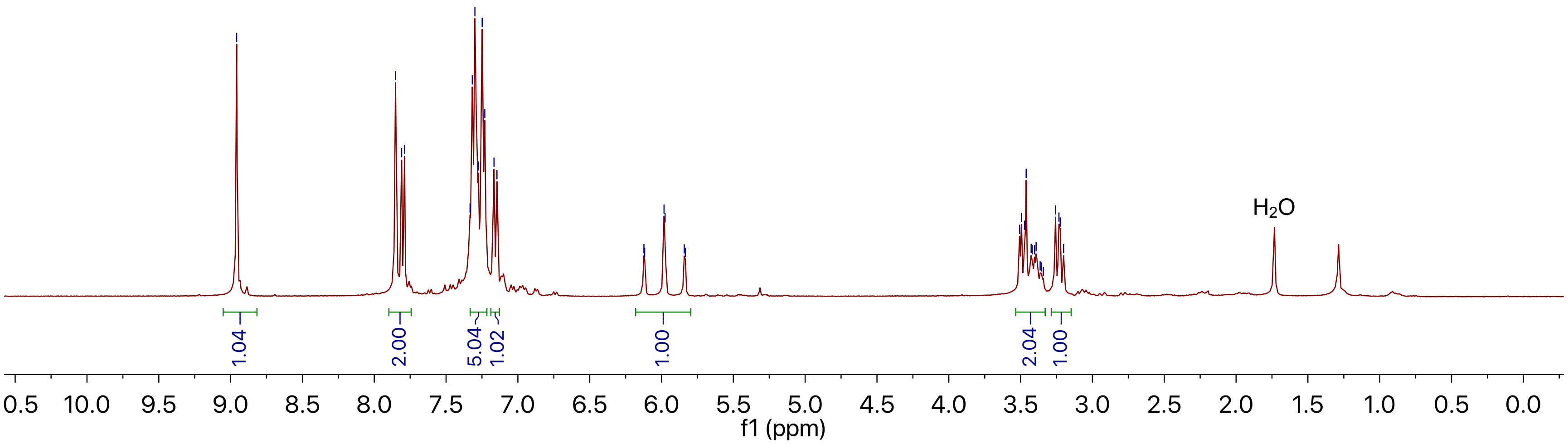

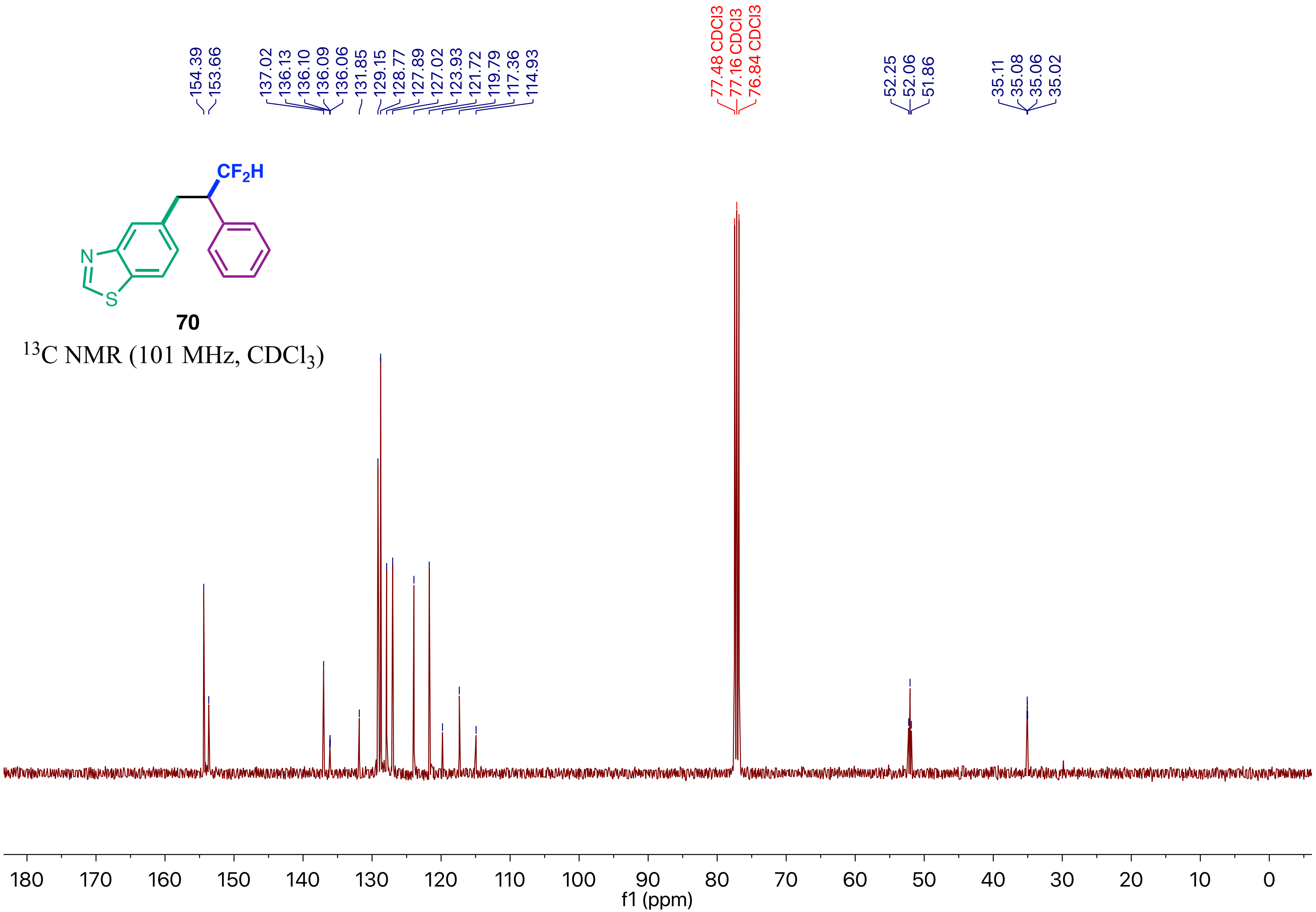

70

^{13}C NMR (101 MHz, $CDCl_3$)

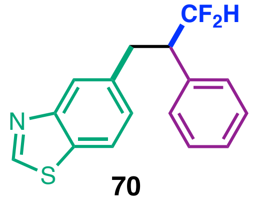

70

^{19}F NMR (376 MHz, $CDCl_3$)

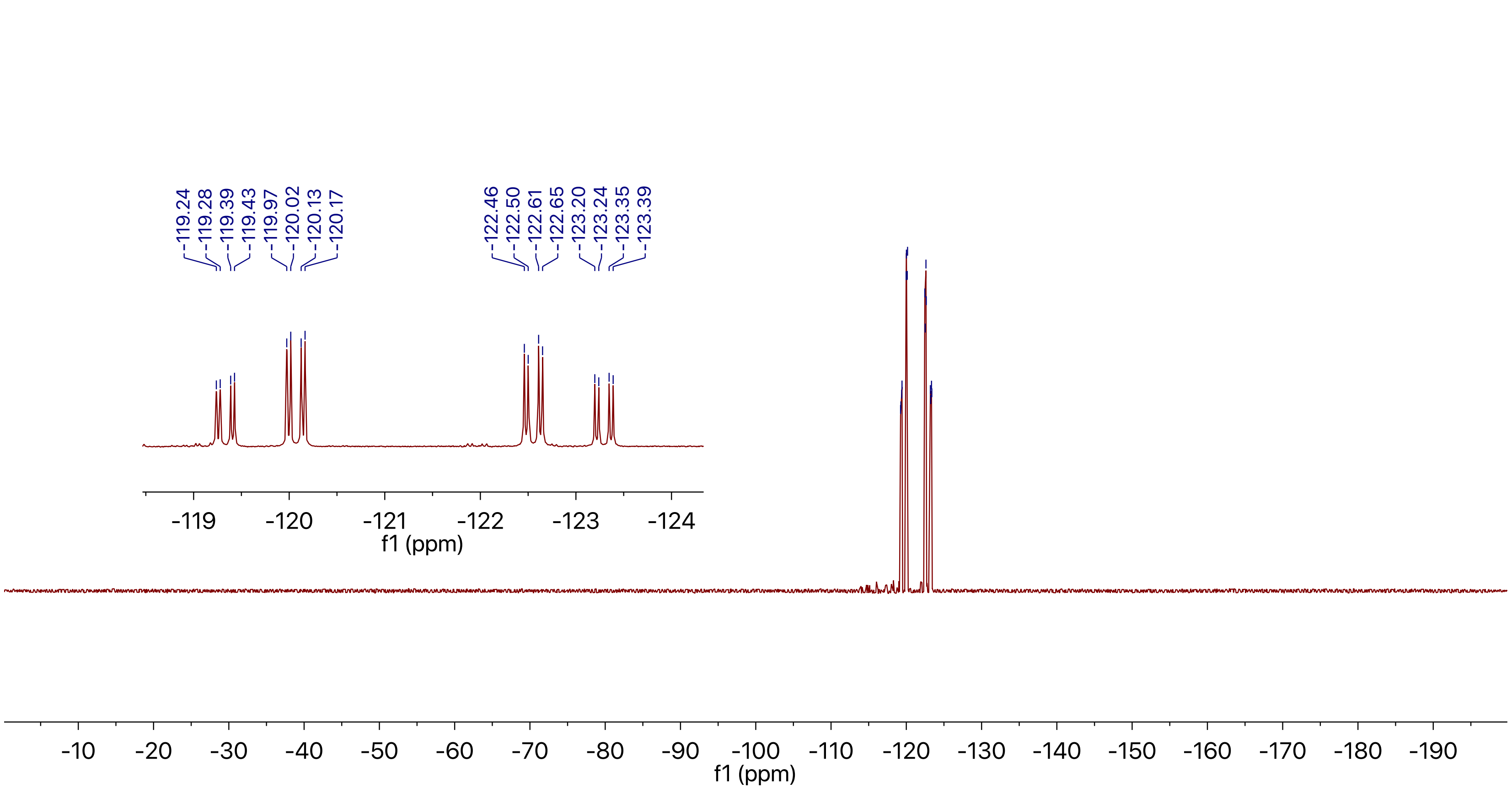

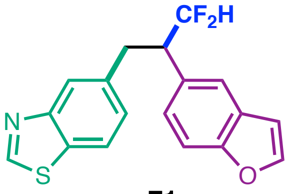

71

^1H NMR (400 MHz, $CDCl_3$)

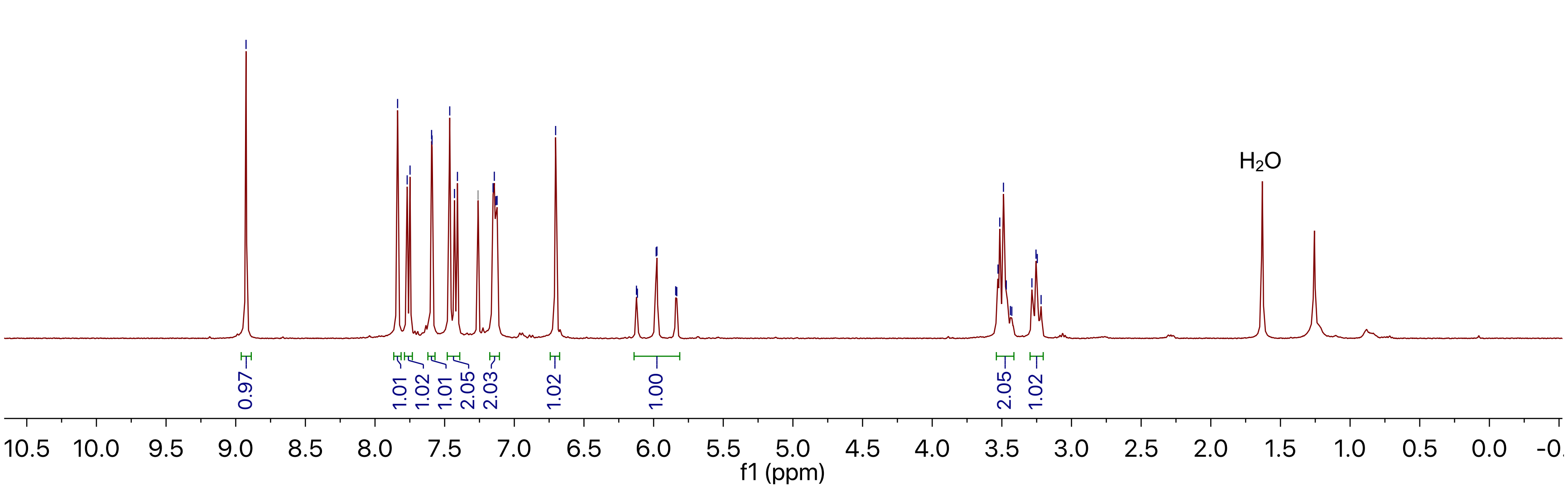

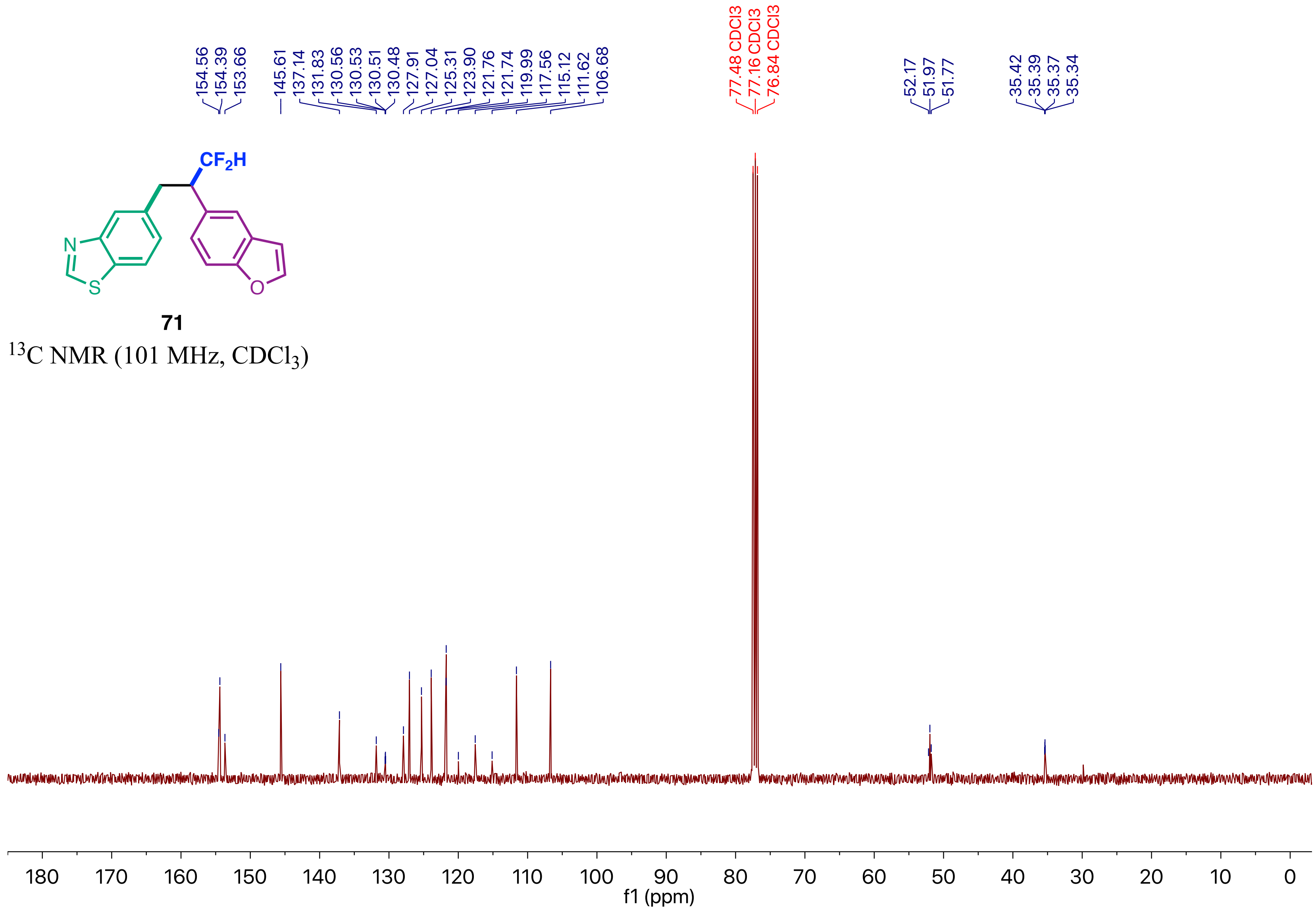

71

^{13}C NMR (101 MHz, $CDCl_3$)

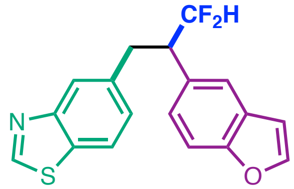

71

^{19}F NMR (376 MHz, $CDCl_3$)

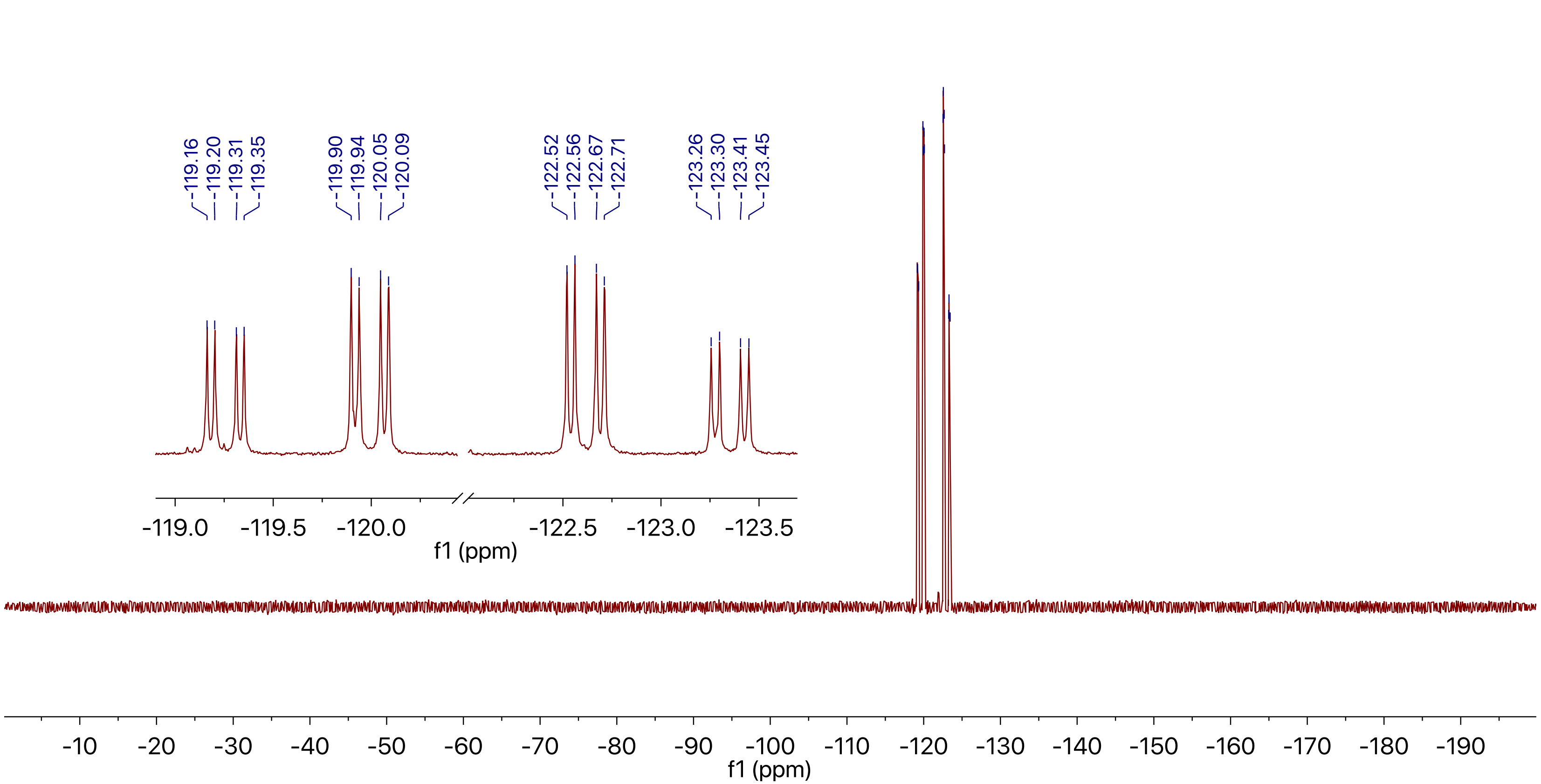

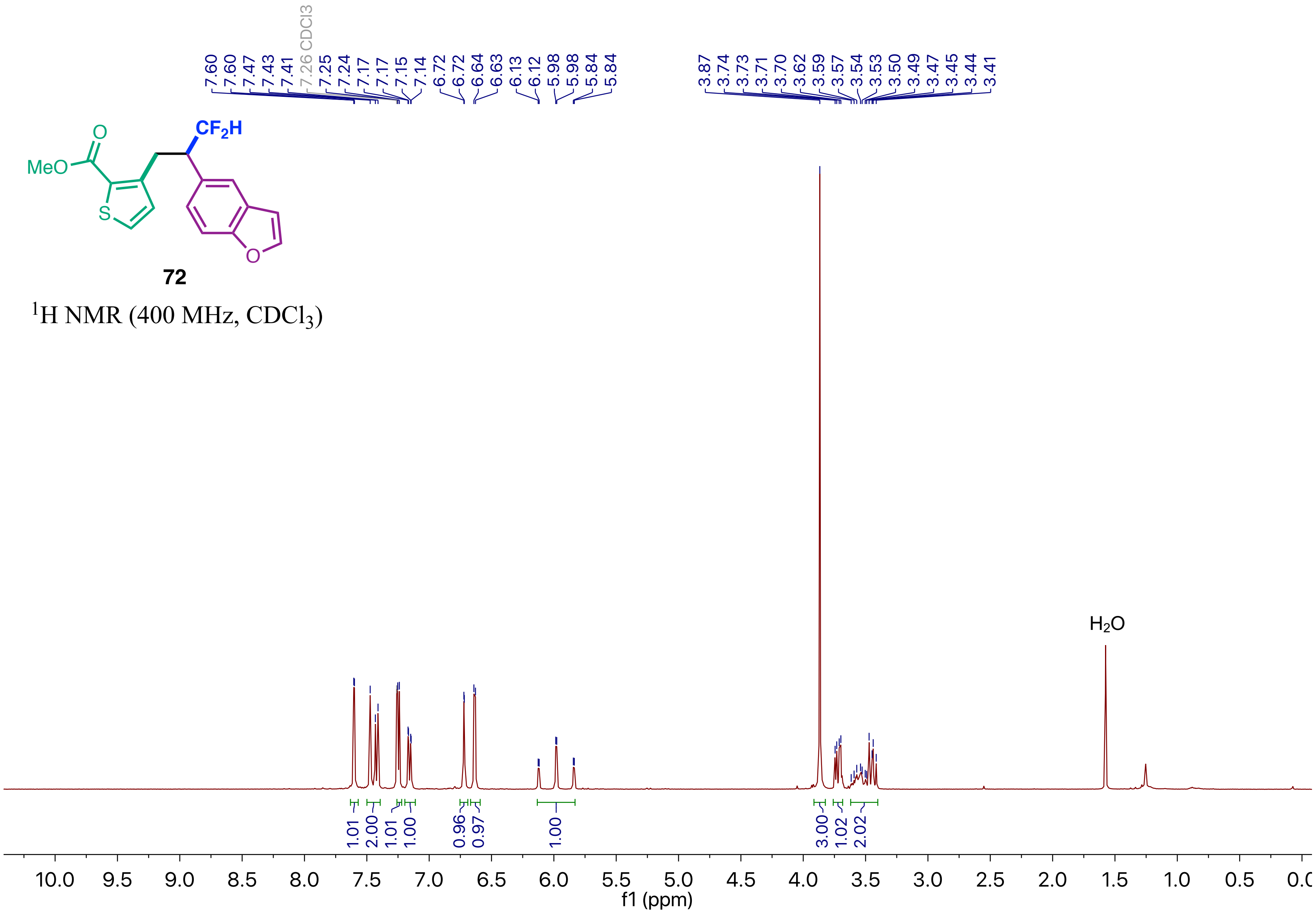

72

^{1}H NMR (400 MHz, $CDCl_3$)

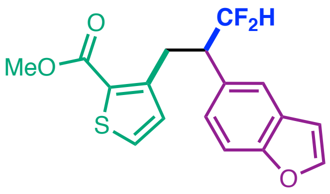

72

^{13}C NMR (101 MHz, $CDCl_3$)

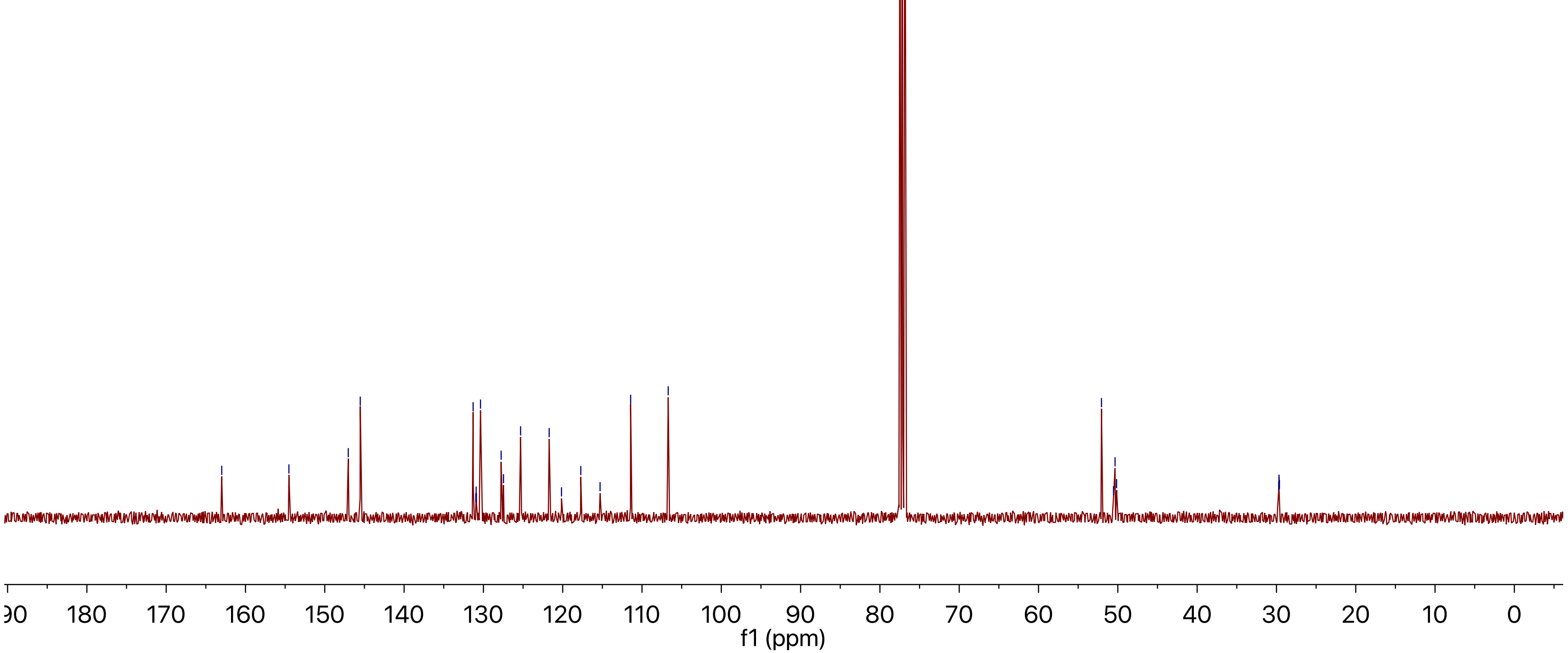

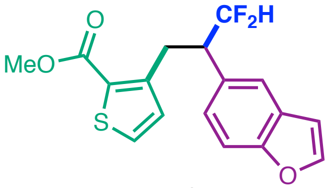

72

^{19}F NMR (376 MHz, $CDCl_3$)

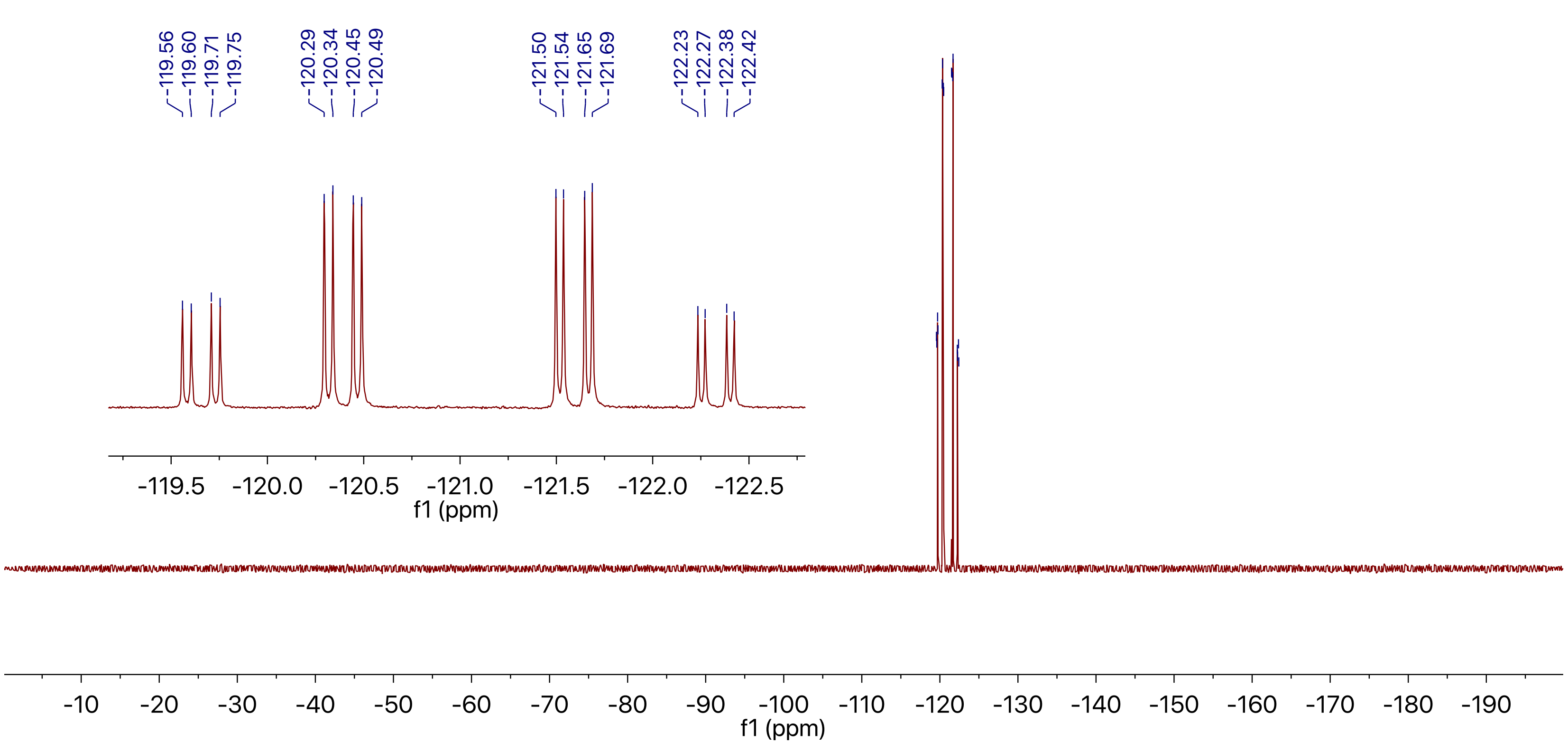

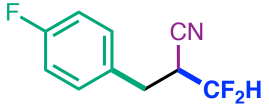

73

^1H NMR (400 MHz, $CDCl_3$)

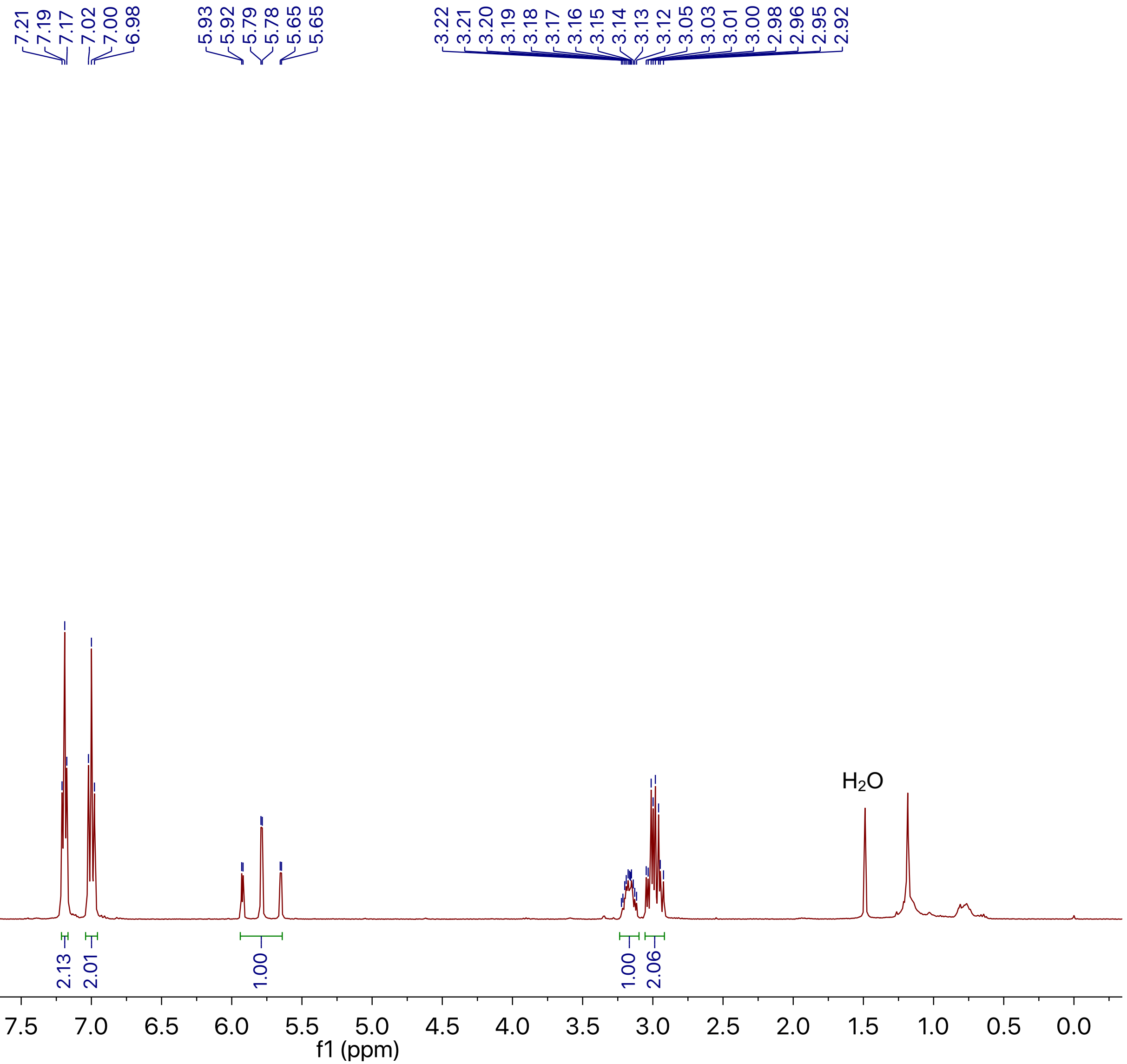

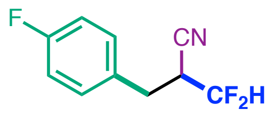

73

^{13}C NMR (101 MHz, $CDCl_3$)

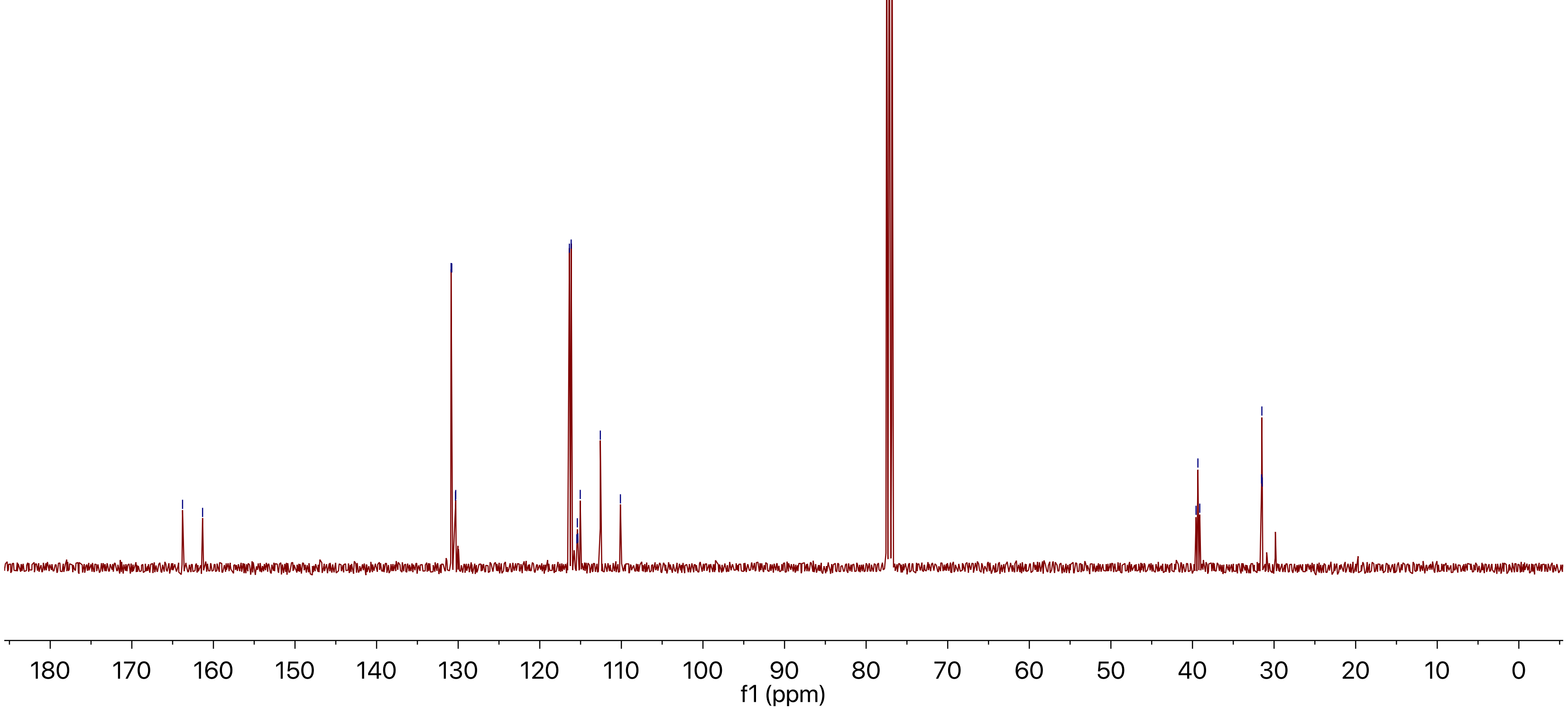

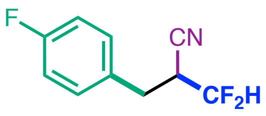

73

^{19}F NMR (376 MHz, CDCl$_3$)

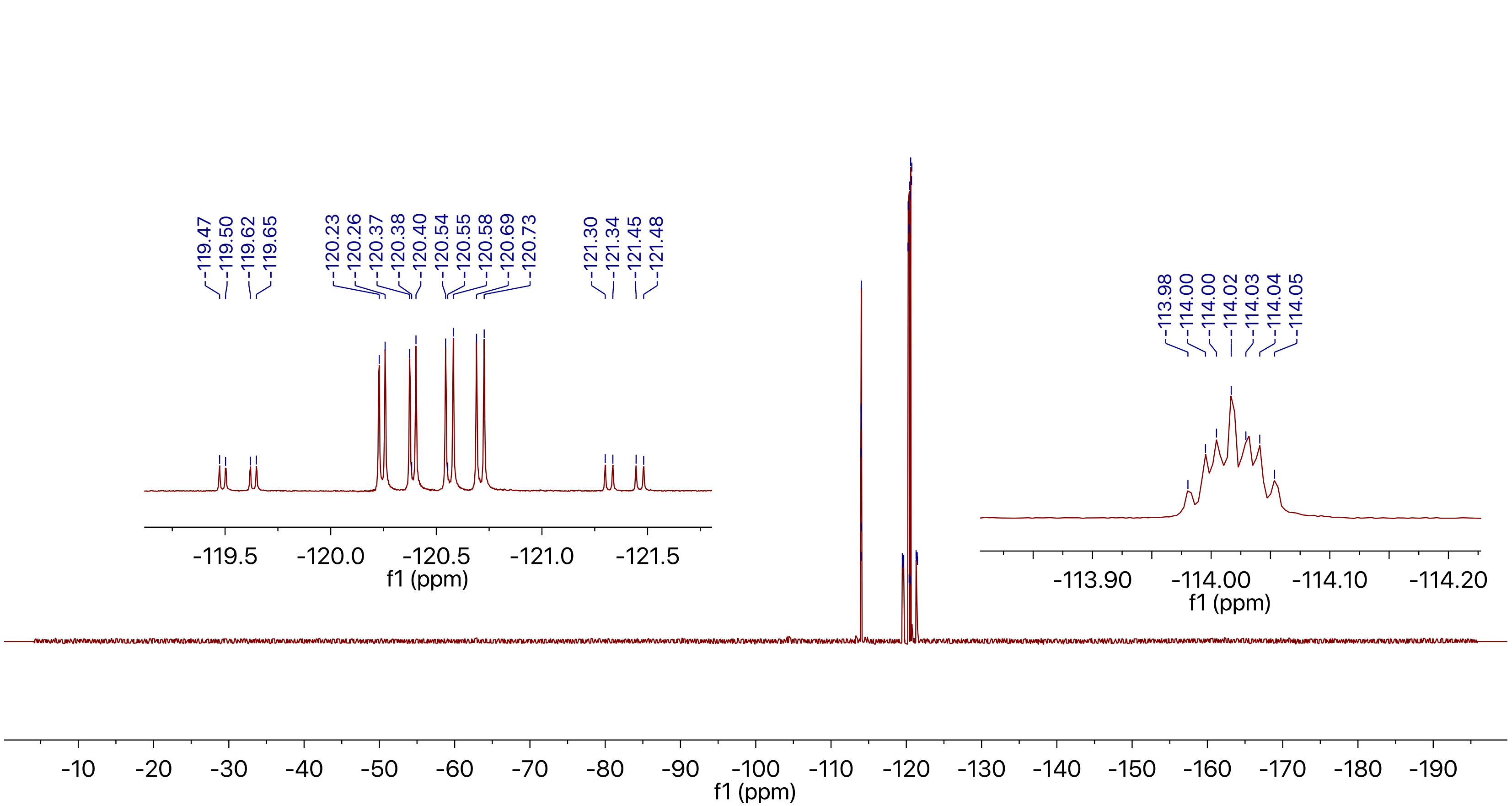

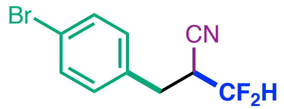

74

^{1}H NMR (400 MHz, $CDCl_3$)

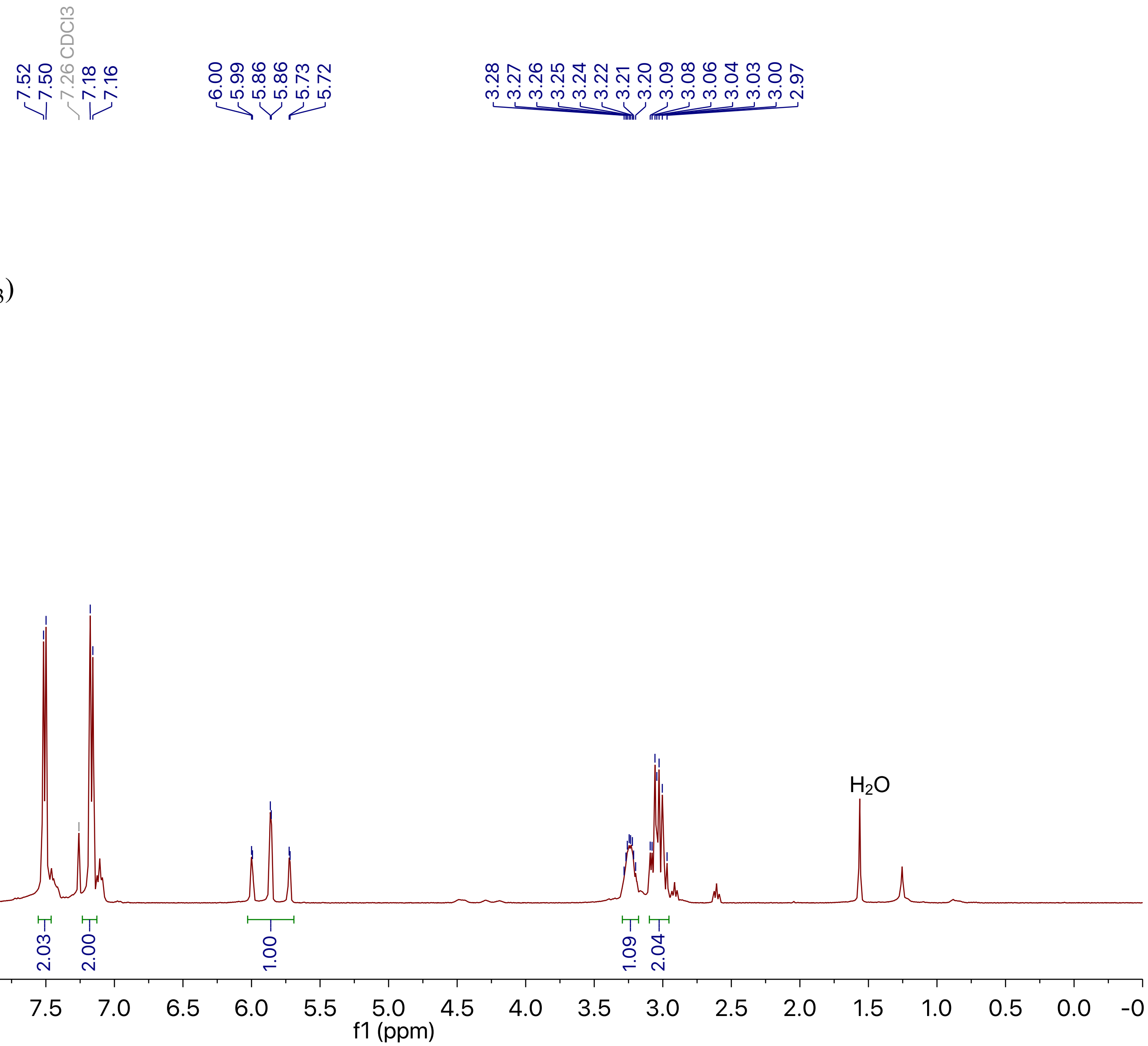

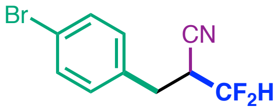

74

^{13}C NMR (101 MHz, $CDCl_3$)

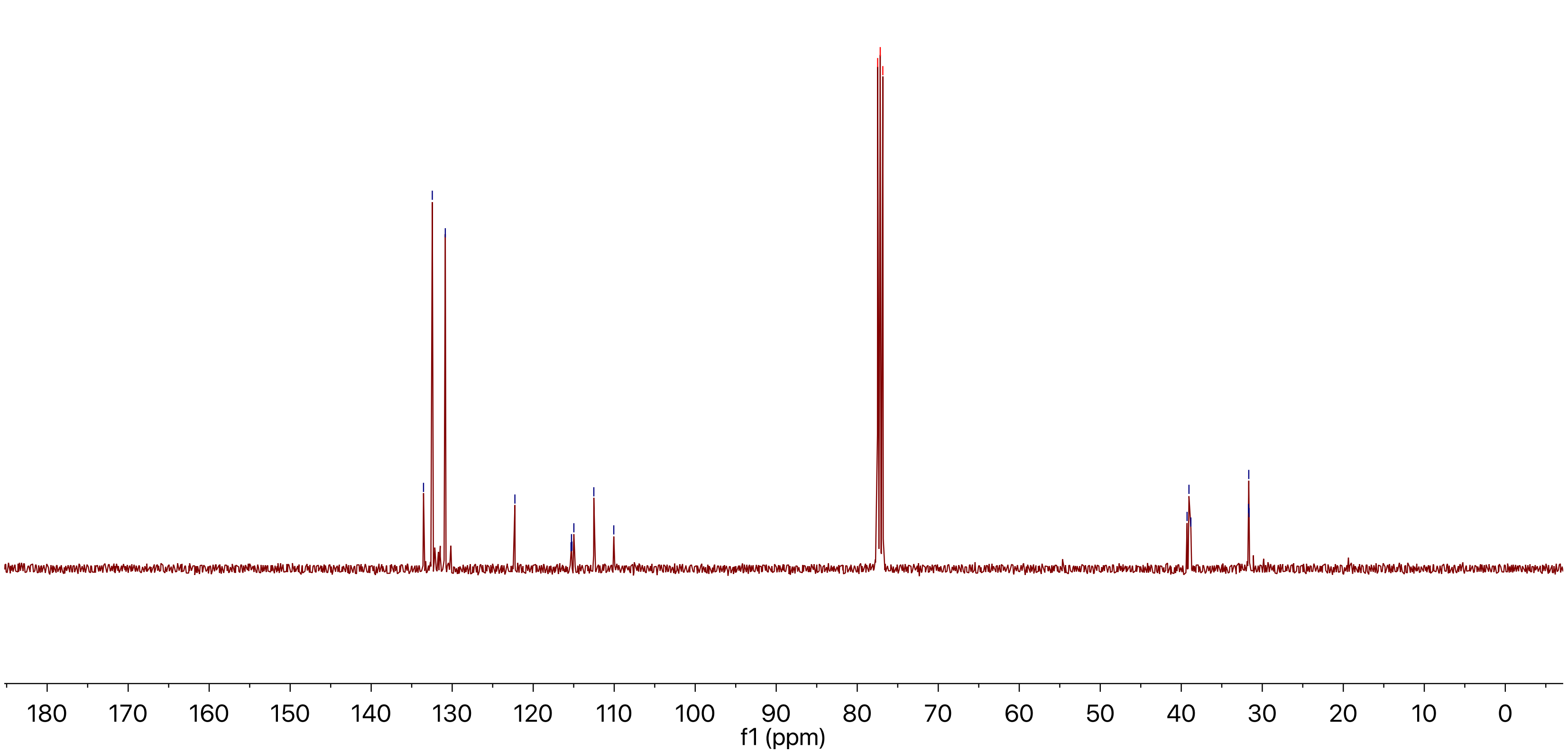

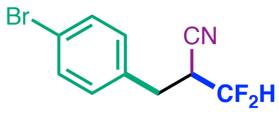

74

^{19}F NMR (376 MHz, $CDCl_3$)

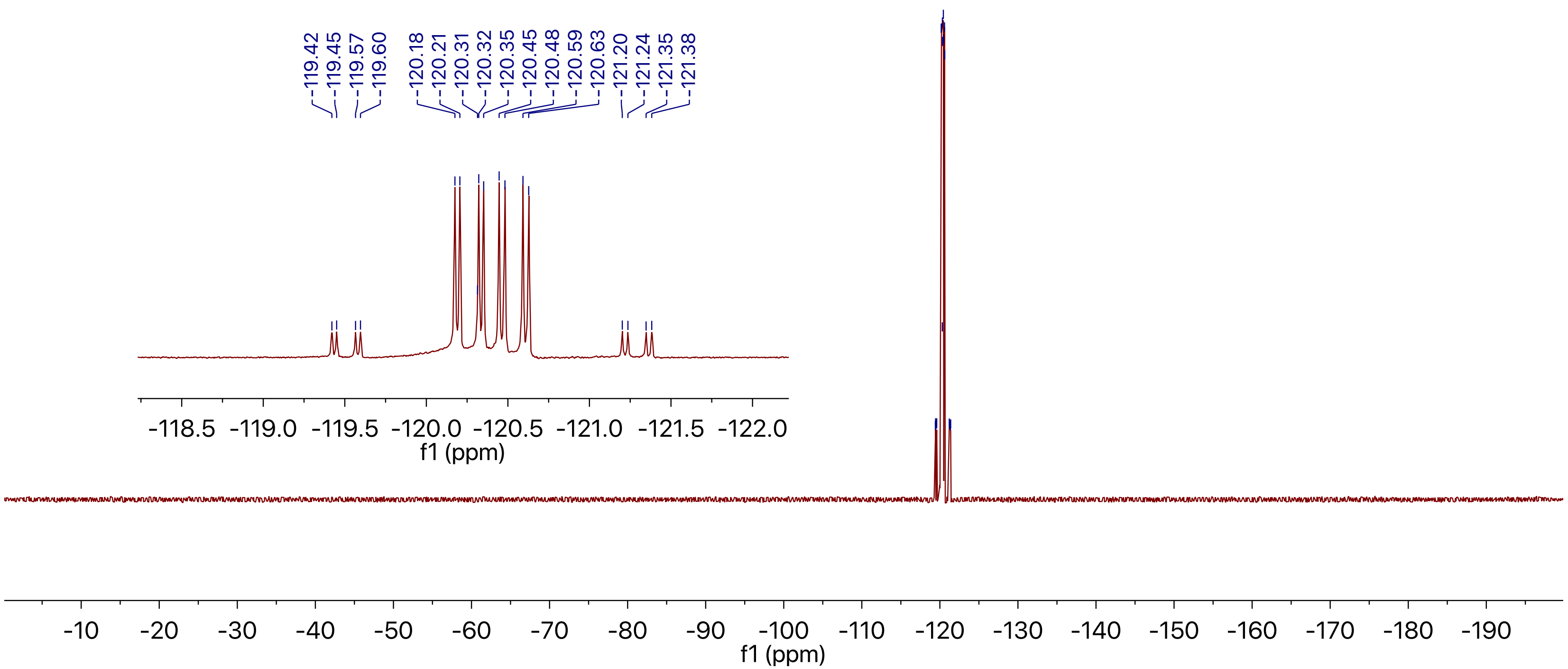

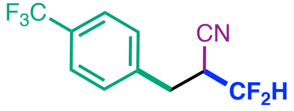

75

^{19}F NMR (376 MHz, $CDCl_3$)

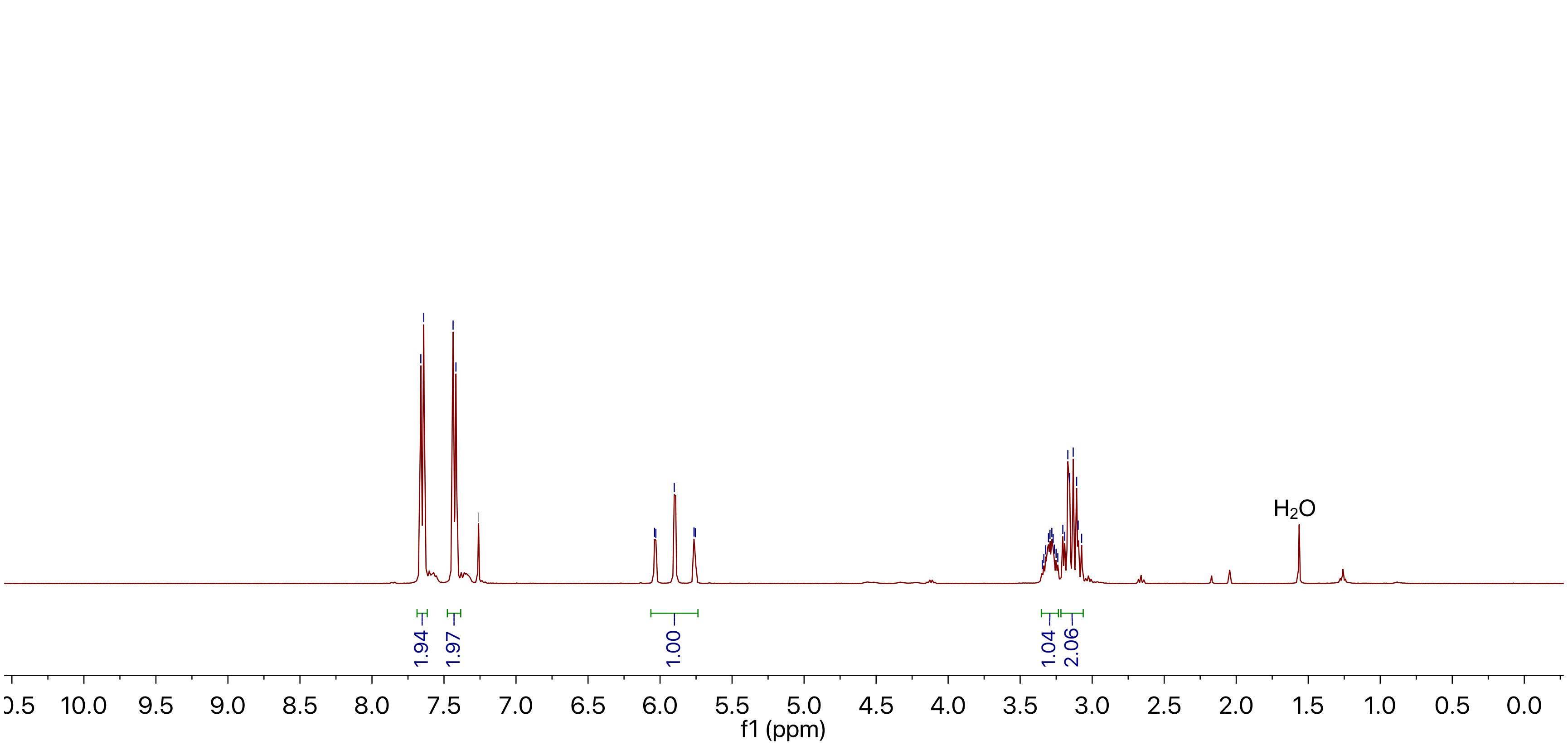

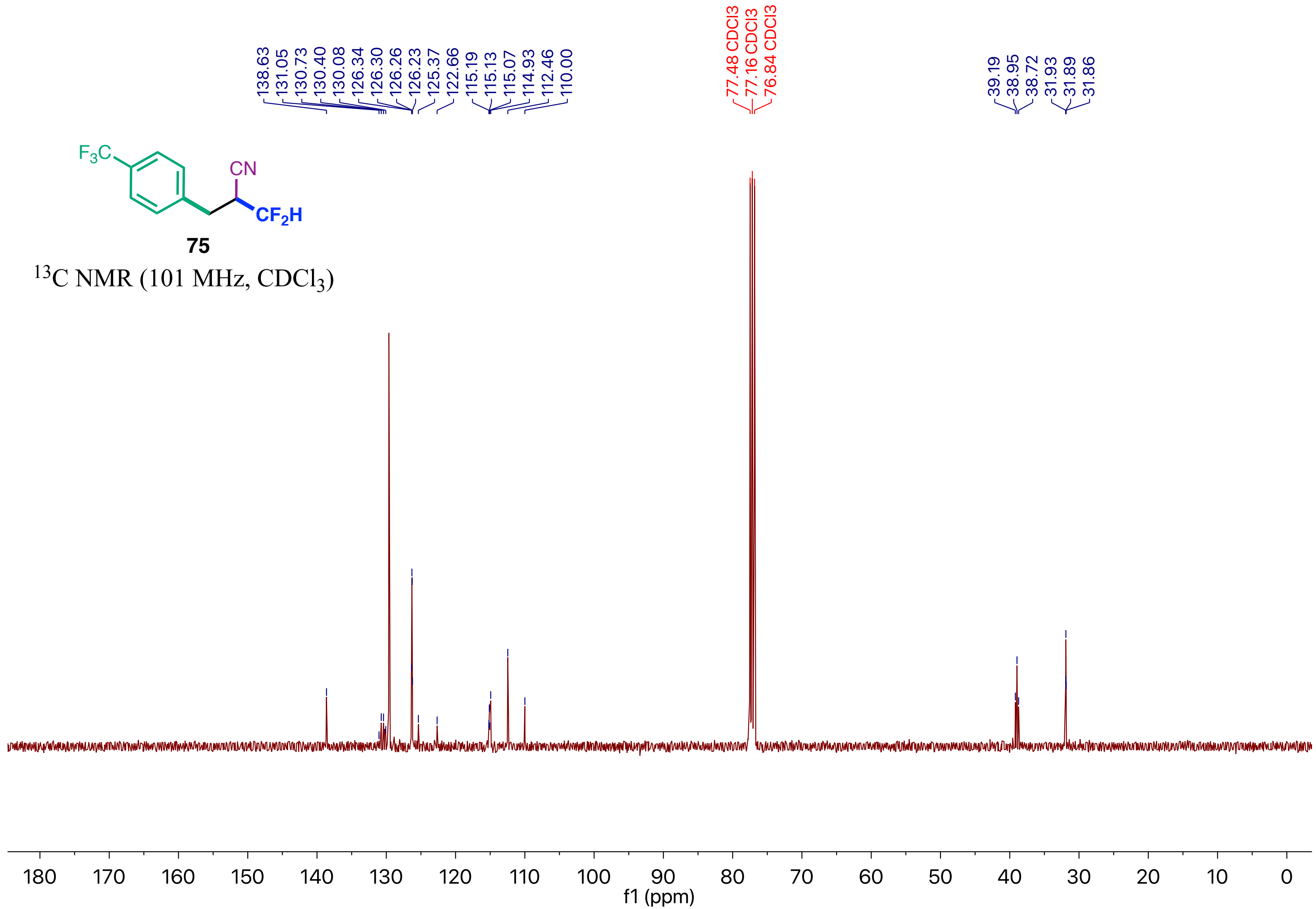

75

^{13}C NMR (101 MHz, $CDCl_3$)

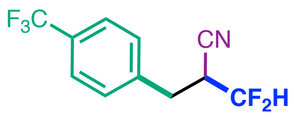

75

^{19}F NMR (376 MHz, $CDCl_3$)

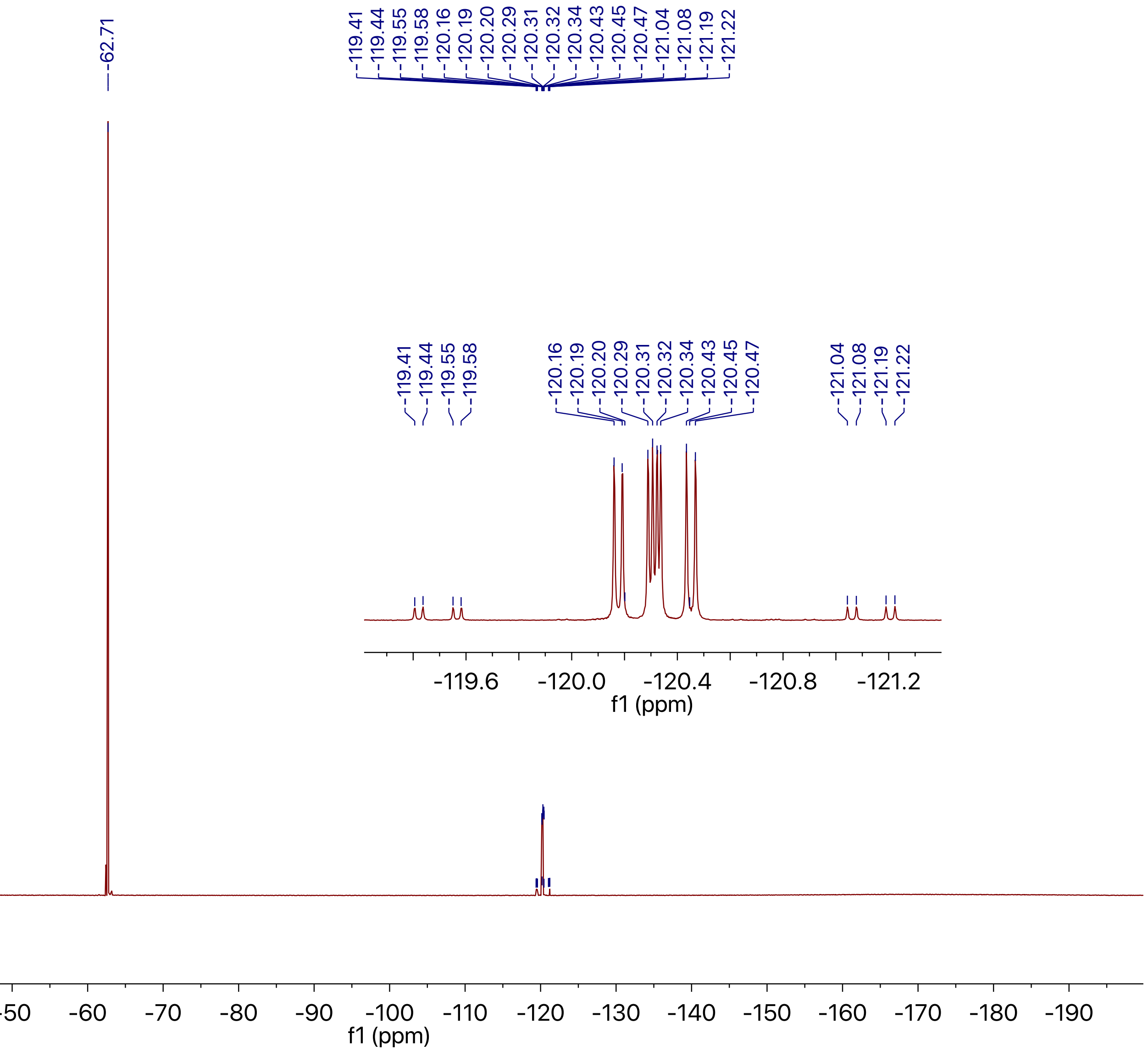

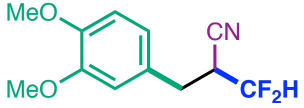

76

^{1}H NMR (400 MHz, $CDCl_3$)

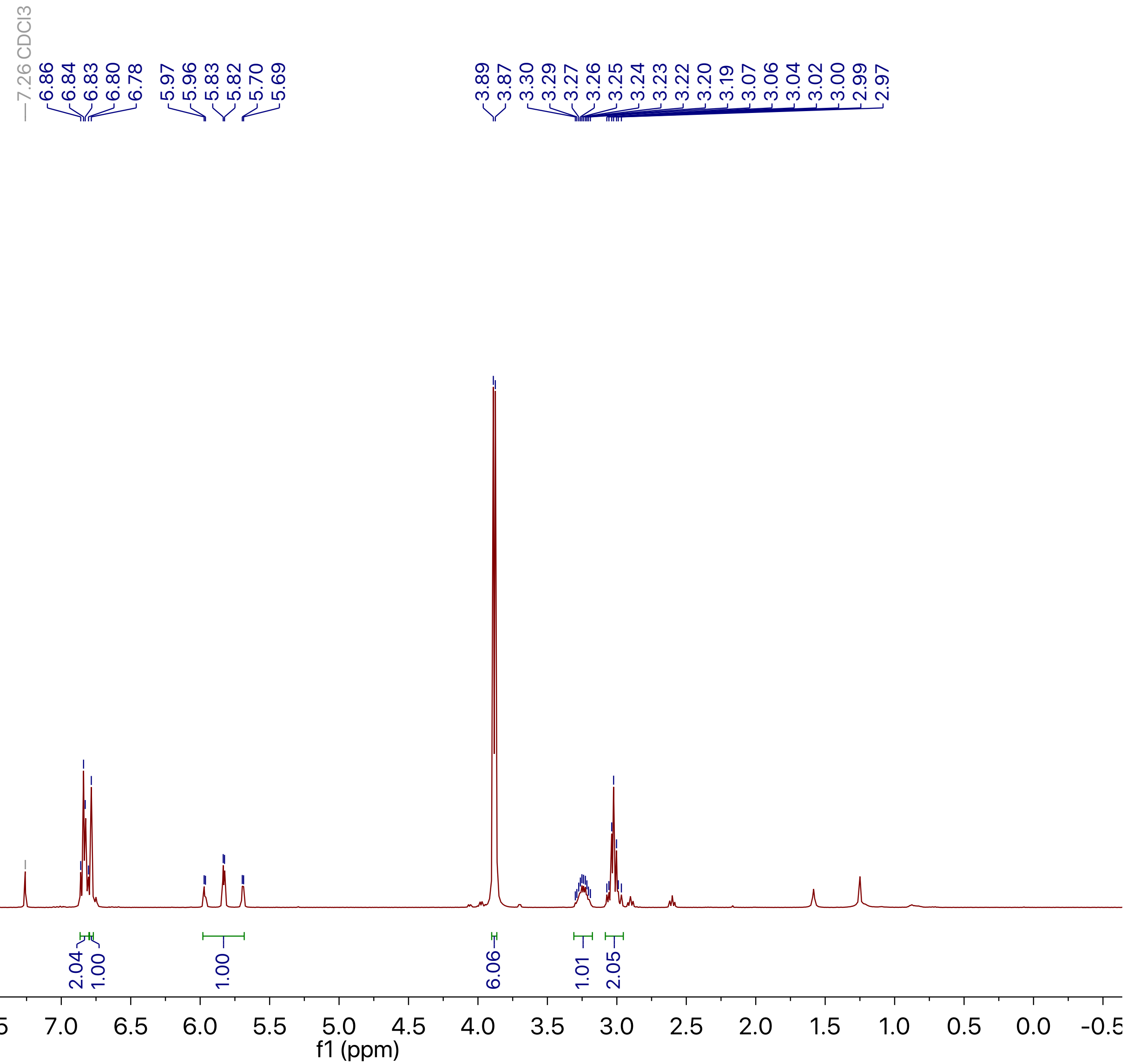

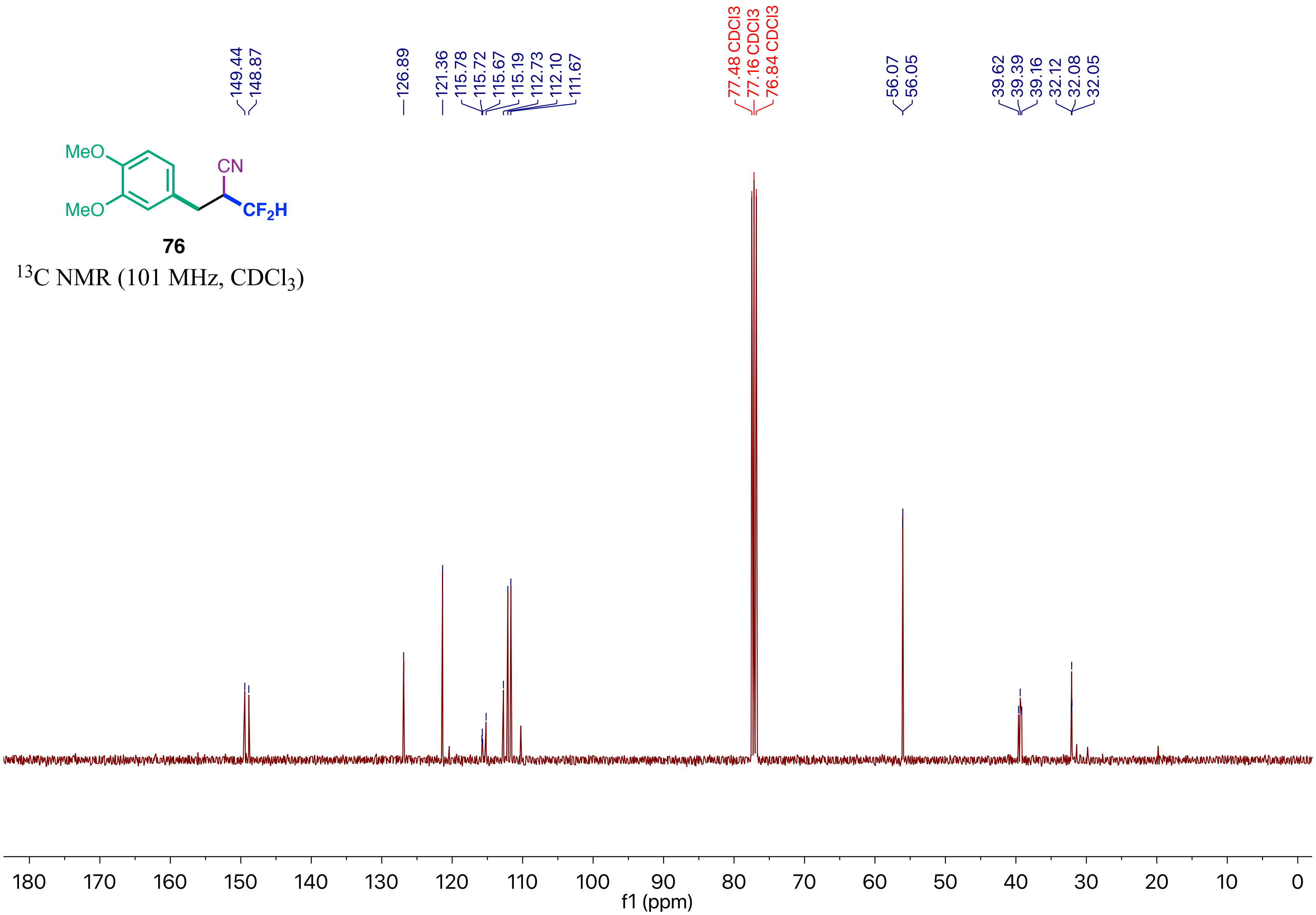

149.44
148.87
126.89
121.36
115.78
115.72
115.67
115.19
112.73
112.10
111.67
77.48 CDCl3
77.16 CDCl3
76.84 CDCl3
56.07
56.05
39.62
39.39
39.16
32.12
32.08
32.05
MeO
MeO
CN
CF_2H
76
^{13}C NMR (101 MHz, $CDCl_3$)
180 170 160 150 140 130 120 110 100 90 80 70 60 50 40 30 20 10 0
f1 (ppm)

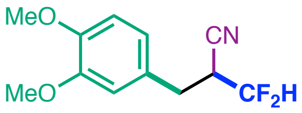

76

^{19}F NMR (376 MHz, $CDCl_3$)

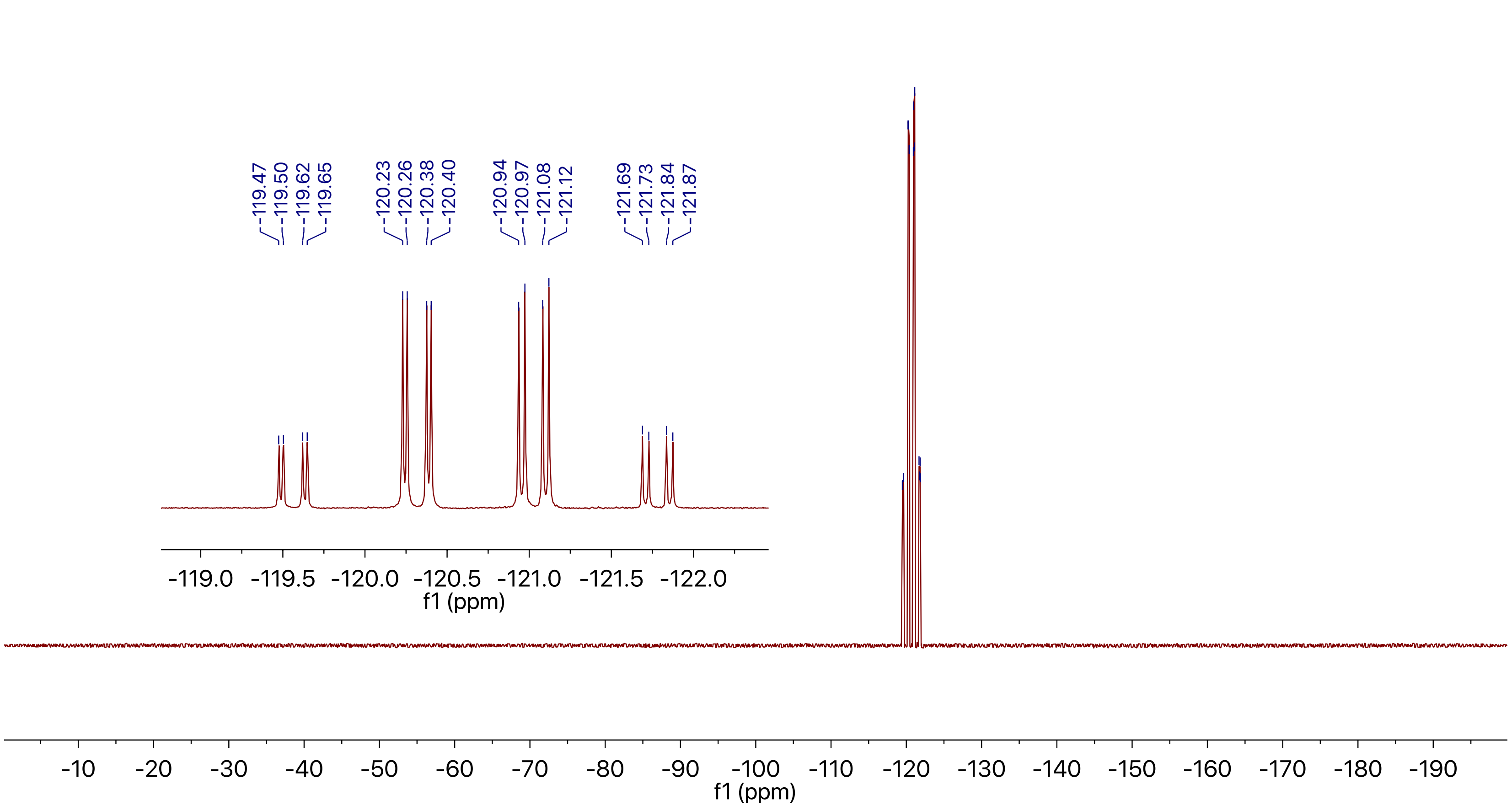

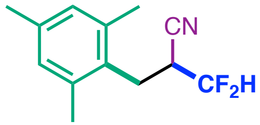

77

^{1}H NMR (400 MHz, $CDCl_3$)

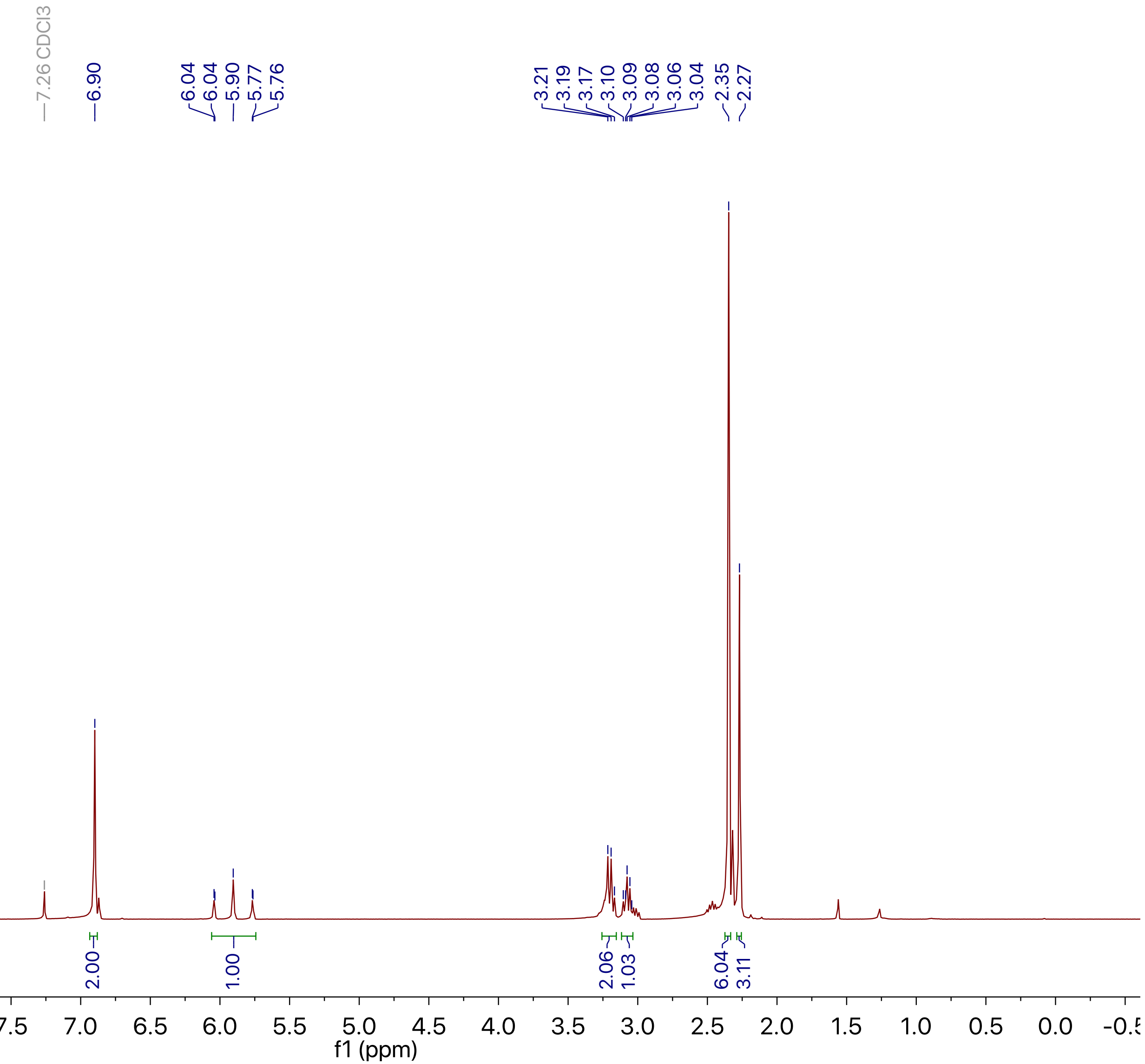

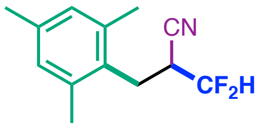

77

^{13}C NMR (101 MHz, $CDCl_3$)

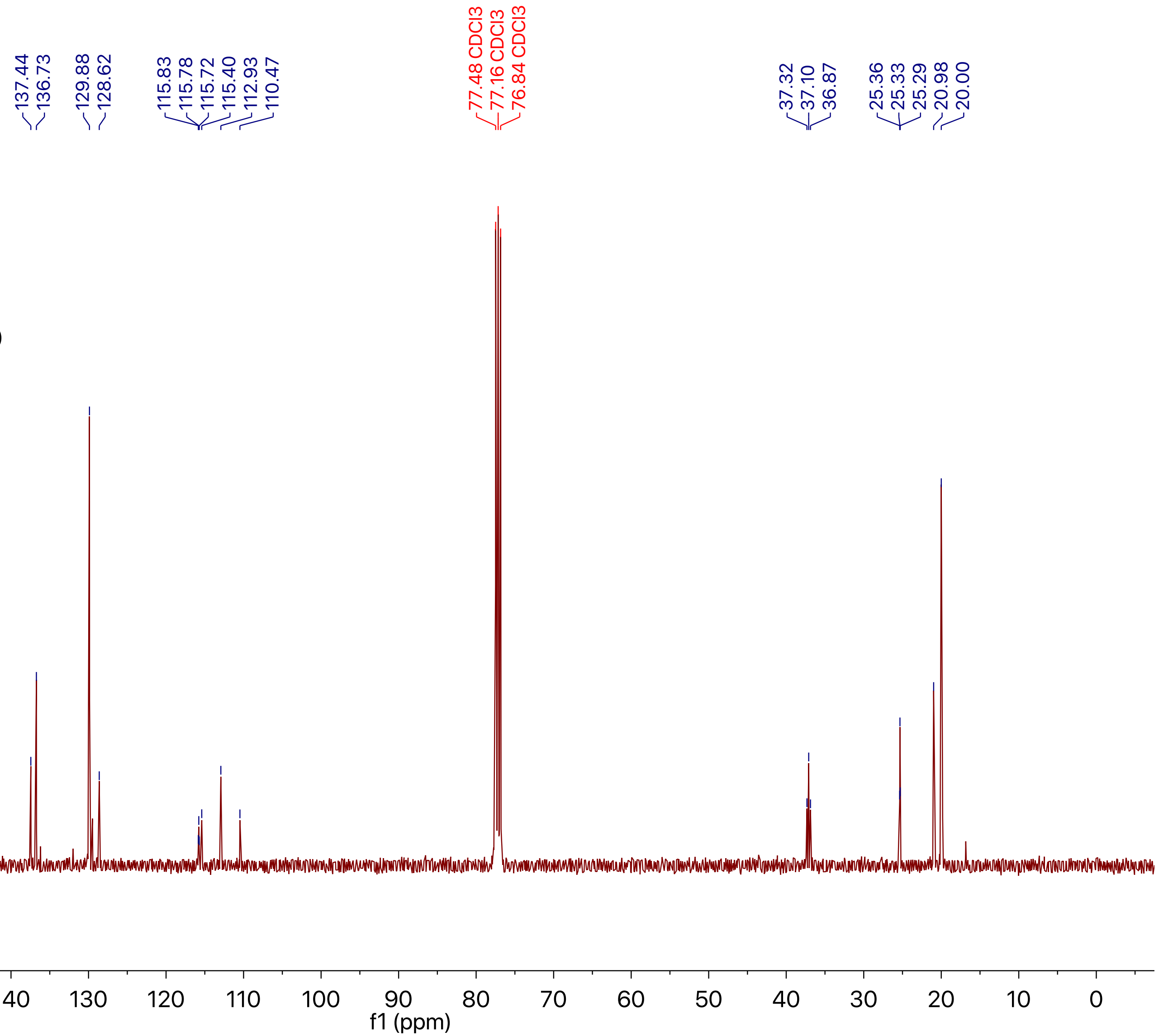

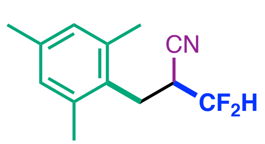

77

^{19}F NMR (376 MHz, $CDCl_3$)

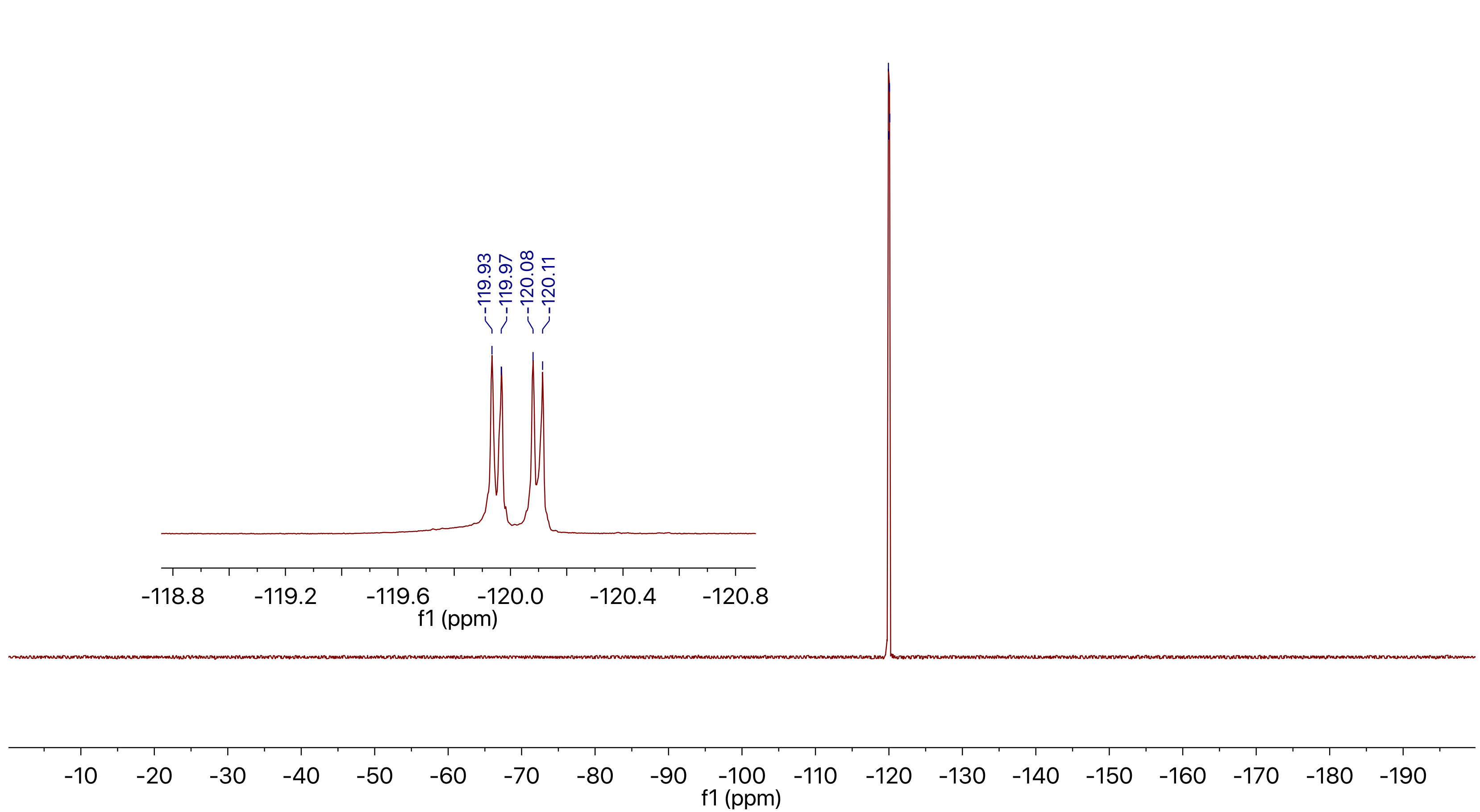

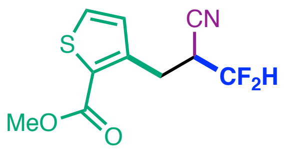

78

^1H NMR (400 MHz, $CDCl_3$)

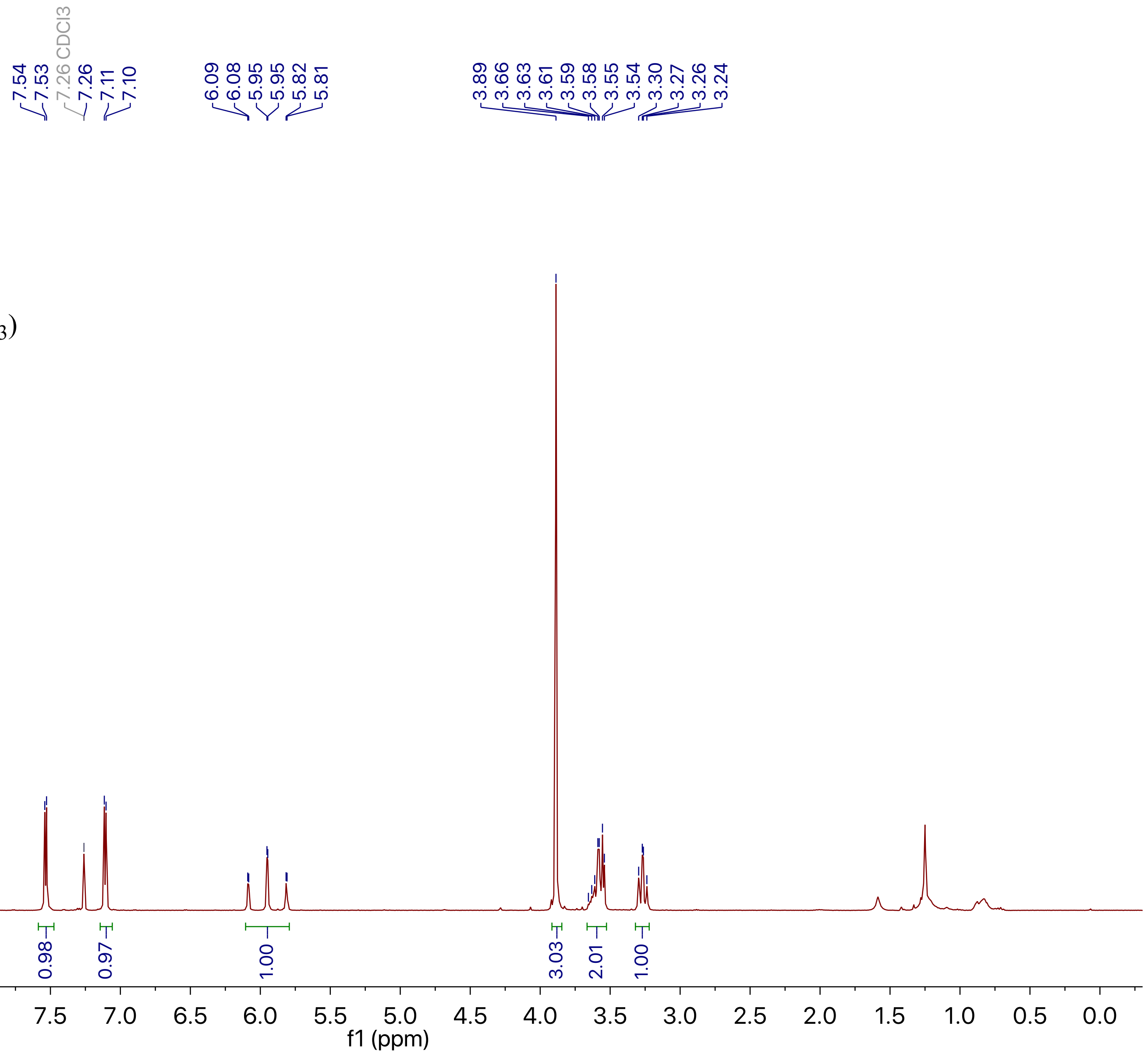

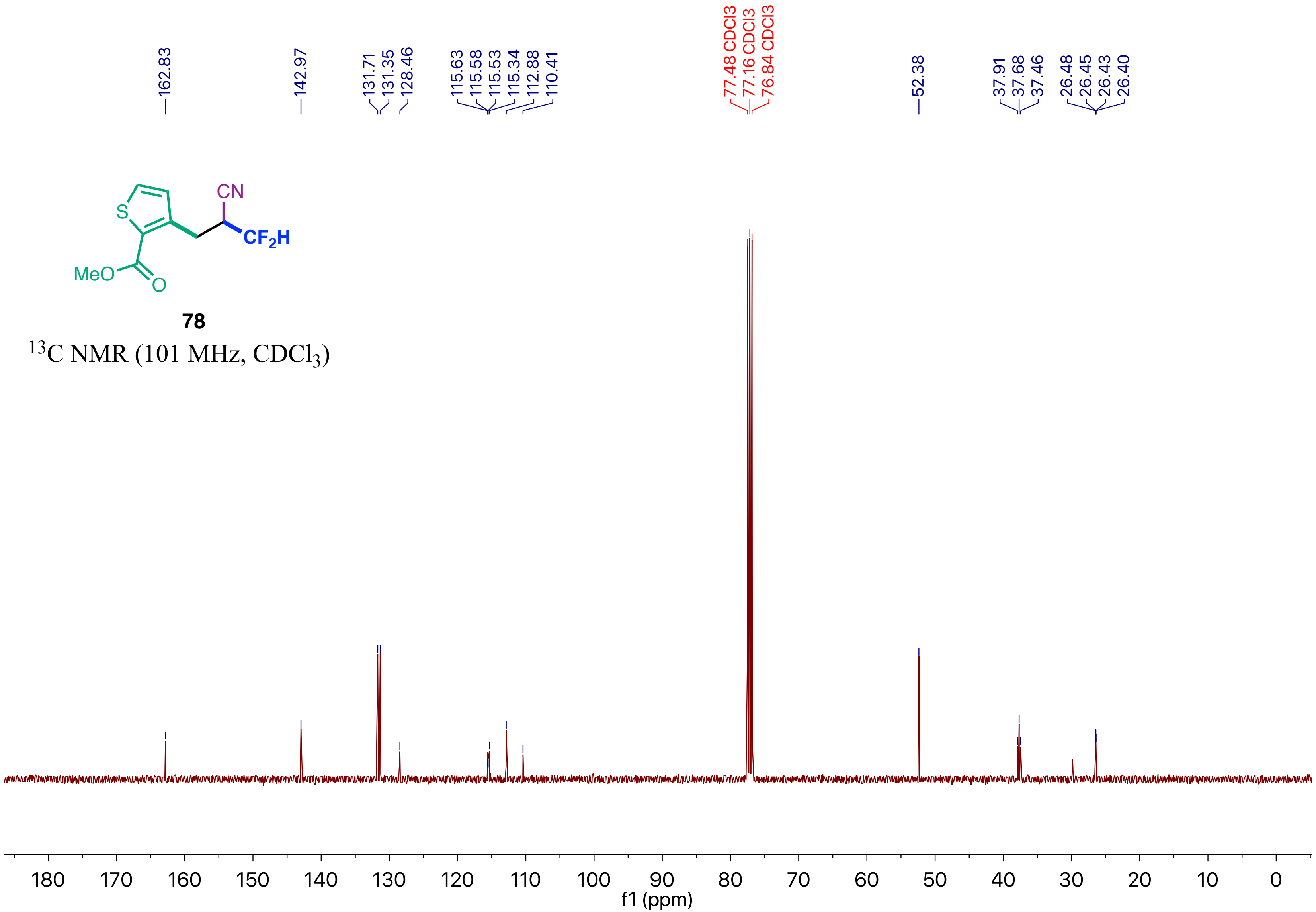

78

^{13}C NMR (101 MHz, $CDCl_3$)

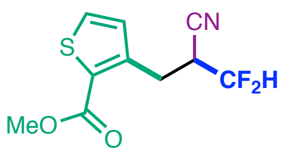

78

^{19}F NMR (376 MHz, $CDCl_3$)

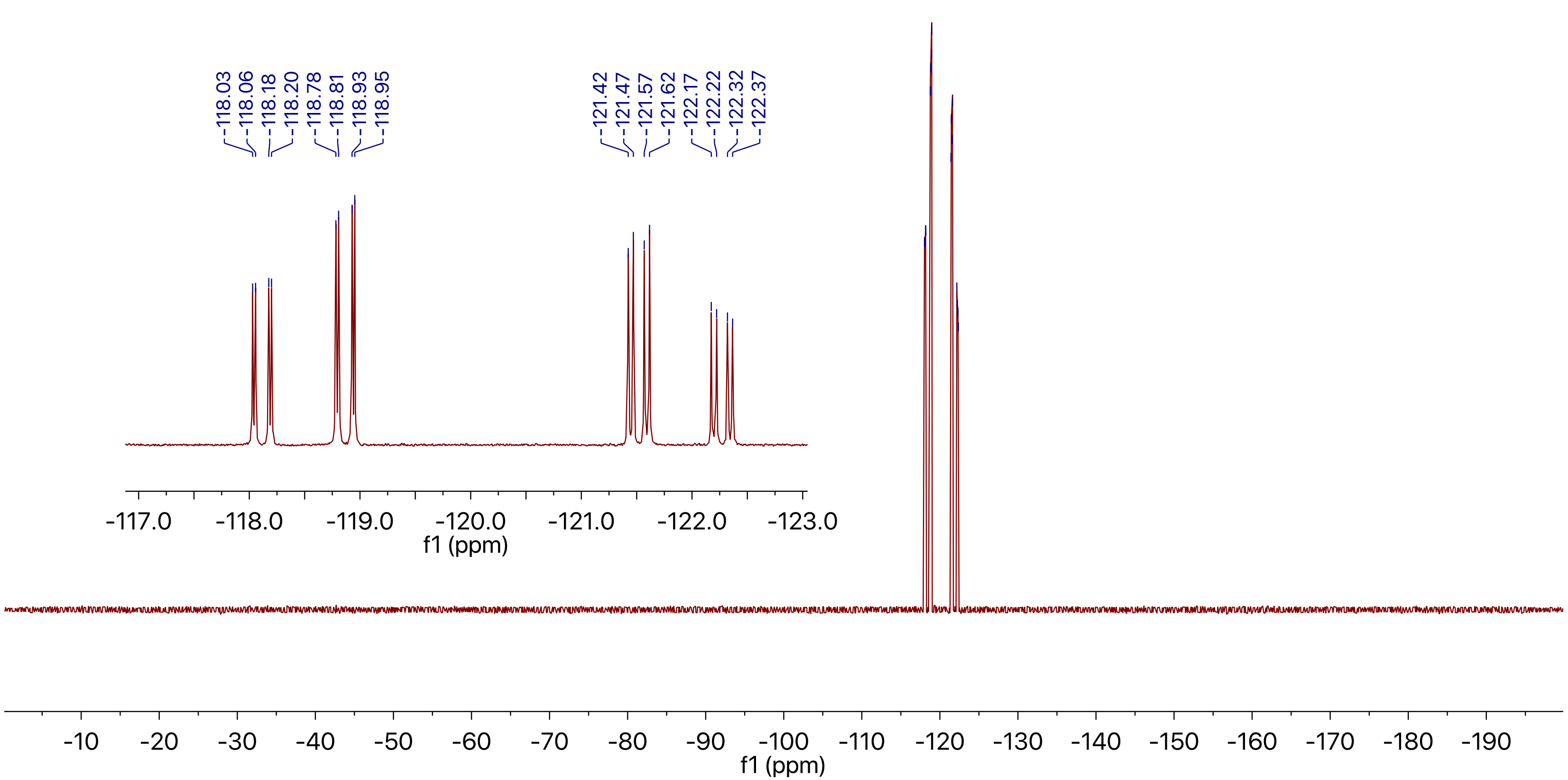

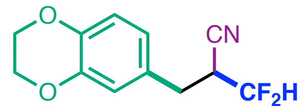

79

^{1}H NMR (400 MHz, $CDCl_3$)

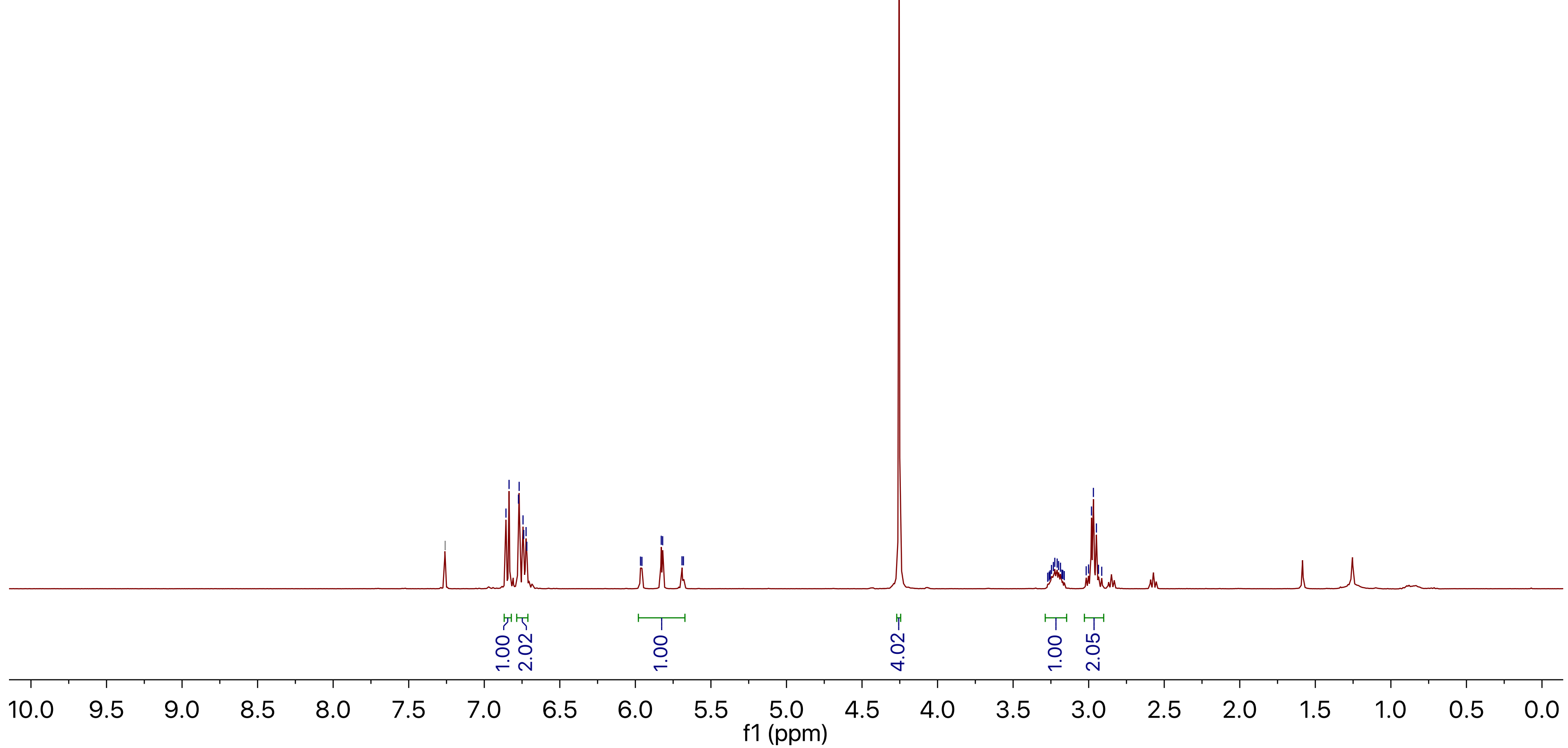

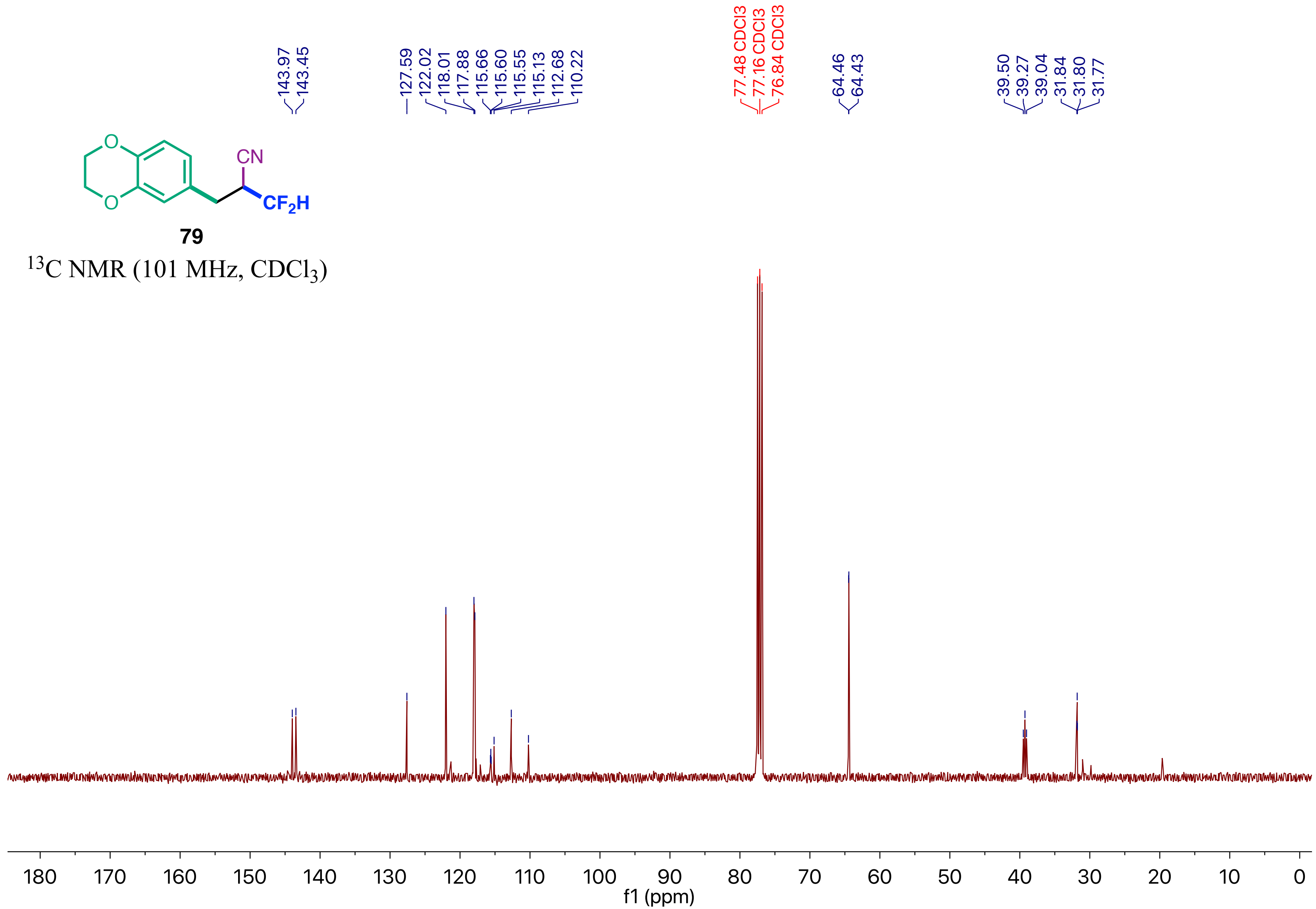
143.97
143.45
127.59
122.02
118.01
117.88
115.66
115.60
115.55
115.13
112.68
110.22
77.48 CDCl3
77.16 CDCl3
76.84 CDCl3
64.46
64.43
39.50
39.27
39.04
31.84
31.80
31.77
CN
CF2H
79
13C NMR (101 MHz, CDCl3)
f1 (ppm)

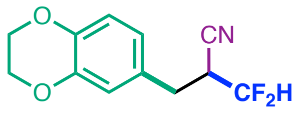

79

^{19}F NMR (376 MHz, $CDCl_3$)

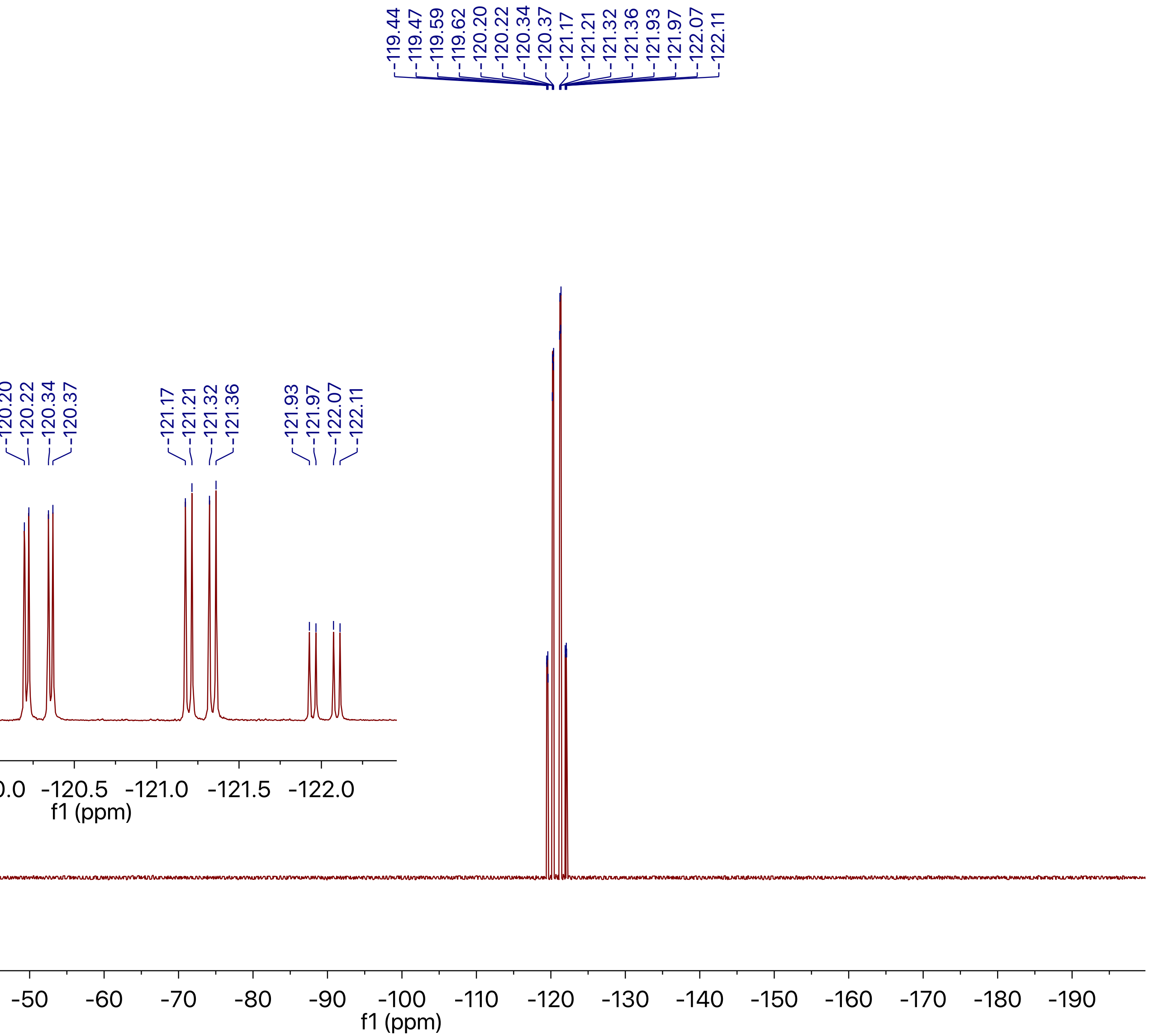

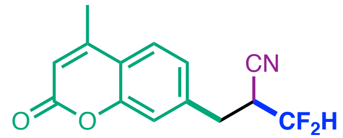

80

^{1}H NMR (400 MHz, $CDCl_3$)

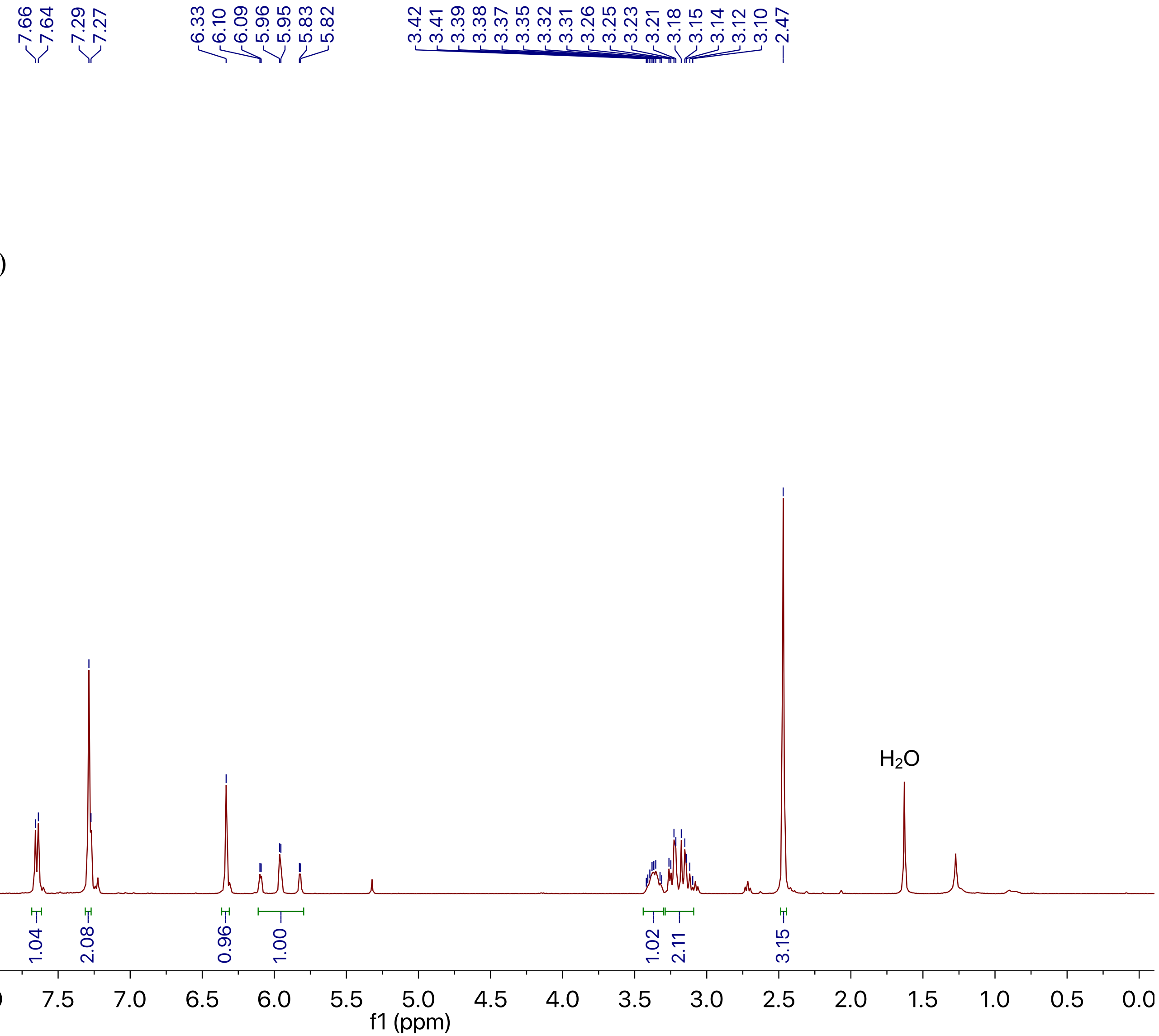

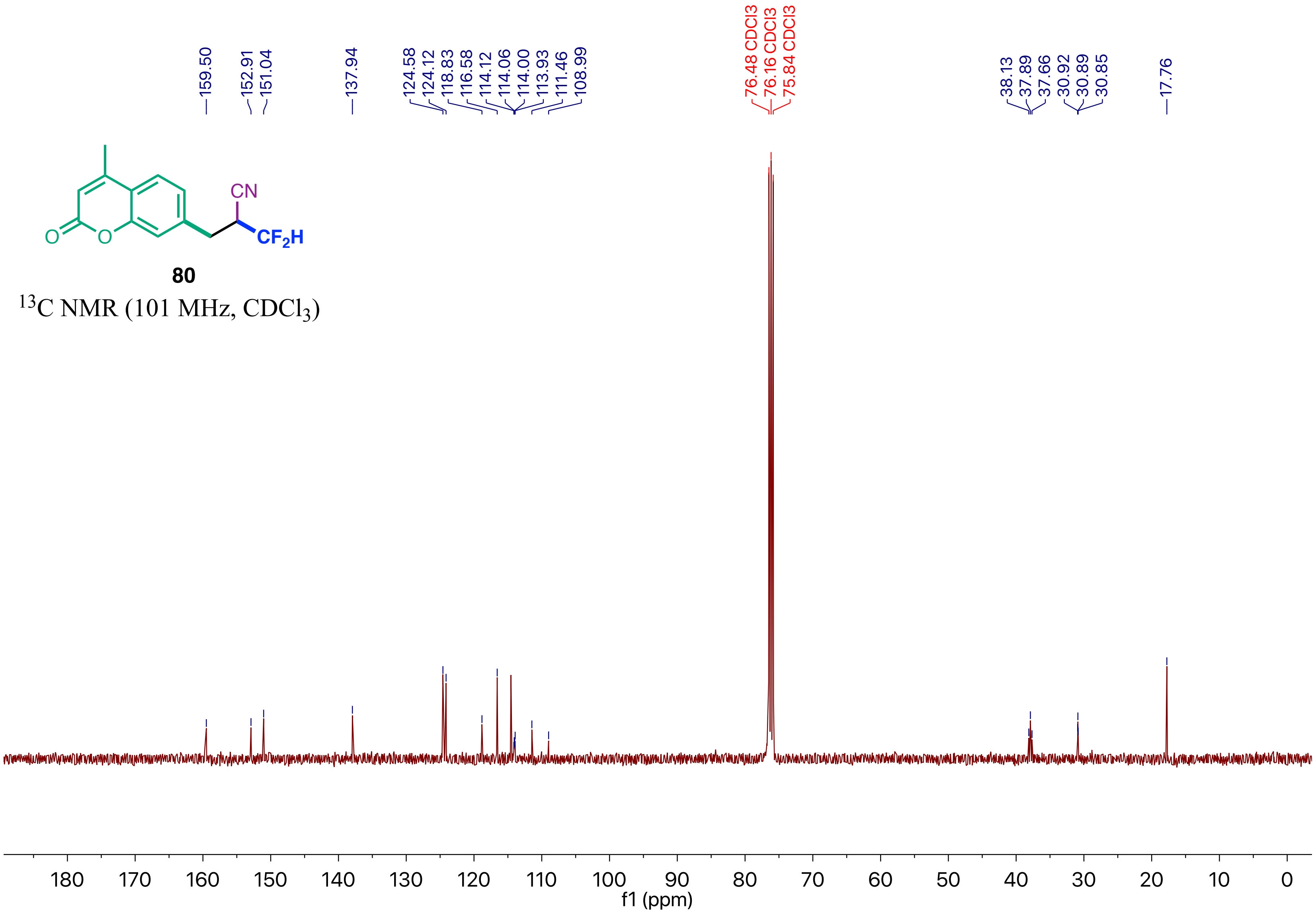

80

^{13}C NMR (101 MHz, $CDCl_3$)

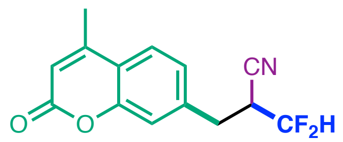

80

^{19}F NMR (376 MHz, $CDCl_3$)

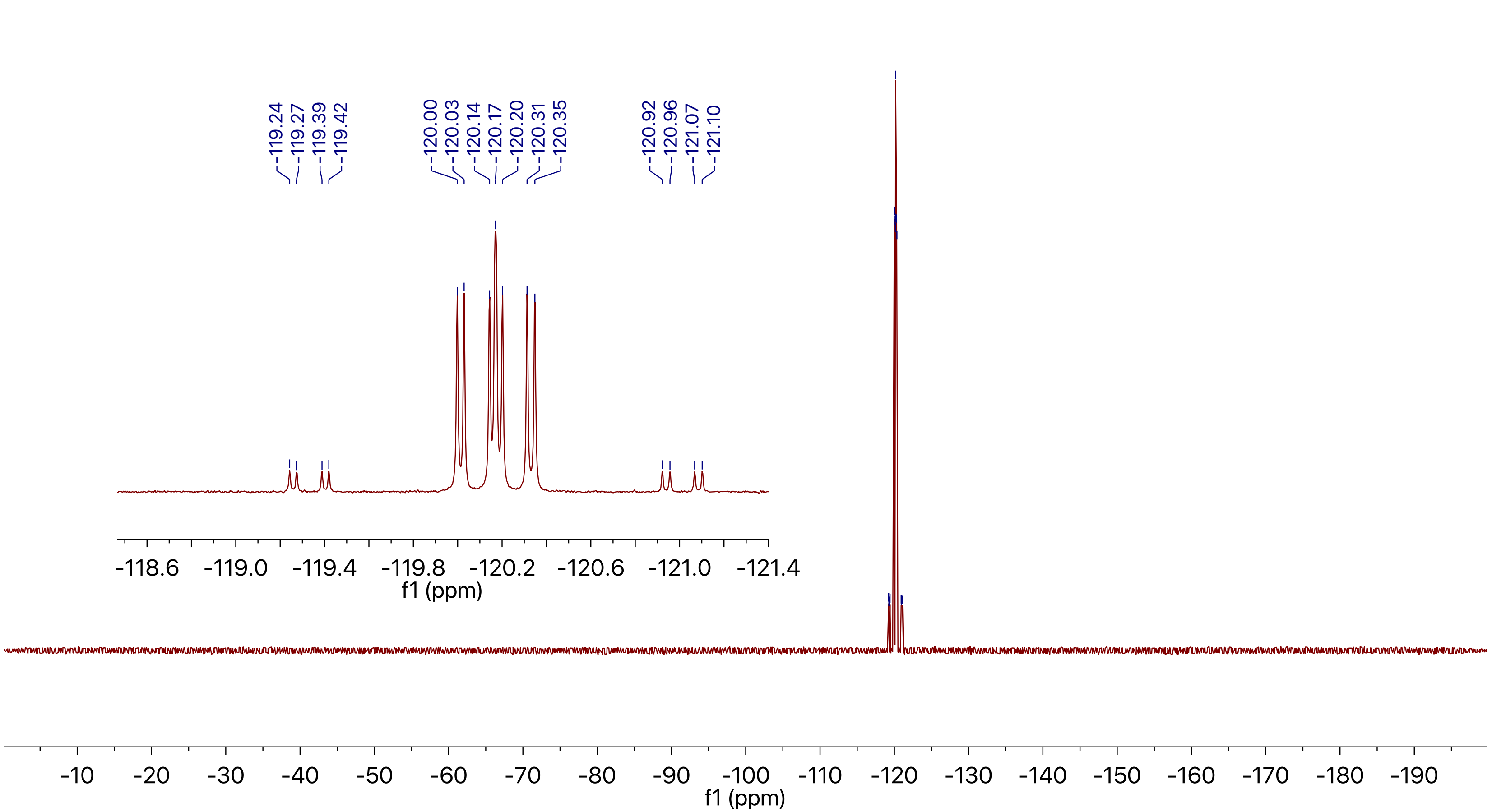

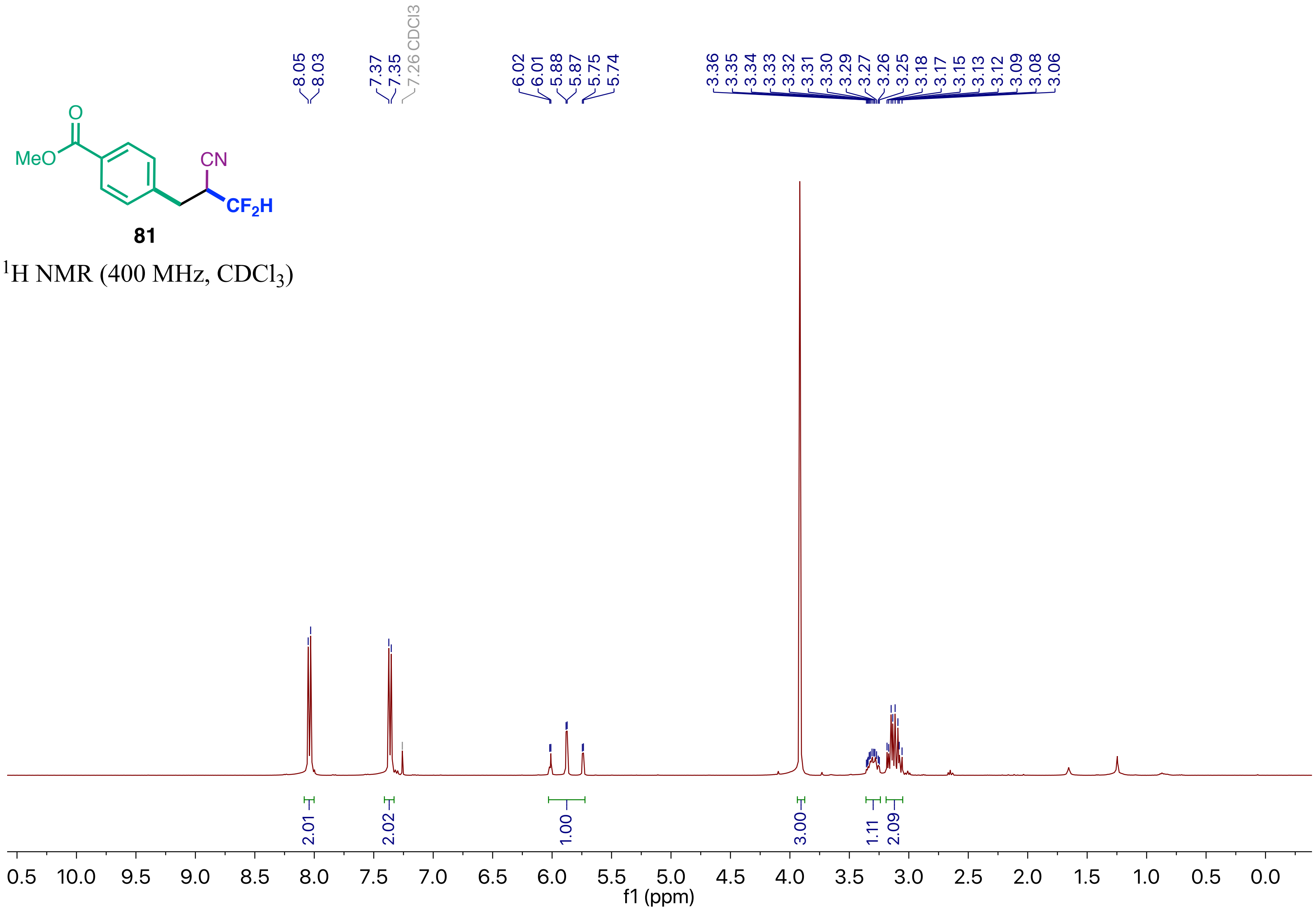

81

^{1}H NMR (400 MHz, $CDCl_3$)

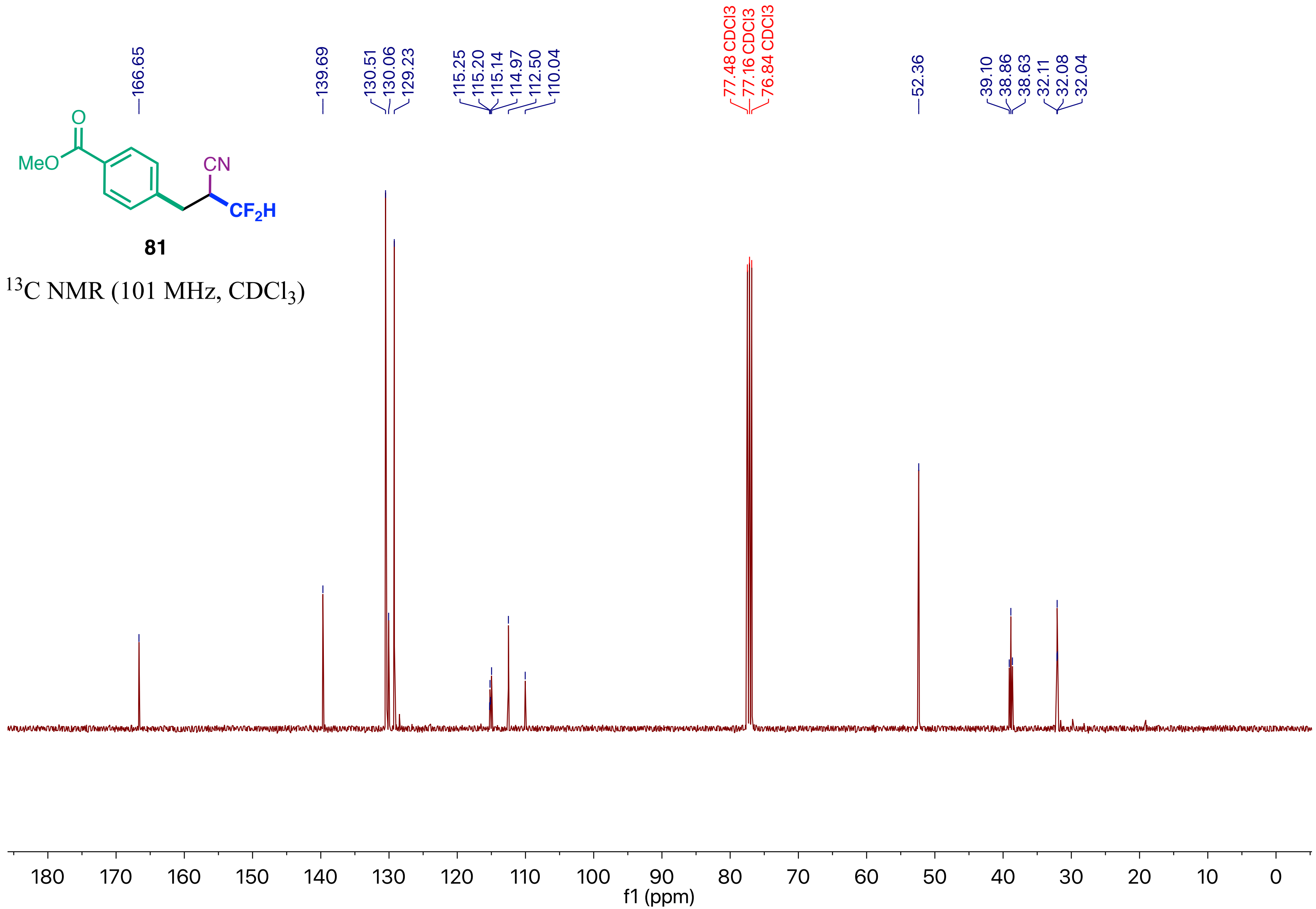

166.65
139.69
130.51
130.06
129.23
115.25
115.20
115.14
114.97
112.50
110.04
77.48 CDCl3
77.16 CDCl3
76.84 CDCl3
52.36
39.10
38.86
38.63
32.11
32.08
32.04
MeO
O
CN
CF2H
81
13C NMR (101 MHz, CDCl3)
180 170 160 150 140 130 120 110 100 90 80 70 60 50 40 30 20 10 0
f1 (ppm)

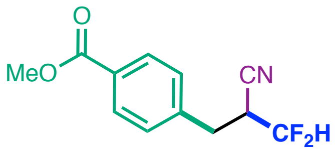

81

^{19}F NMR (376 MHz, $CDCl_3$)

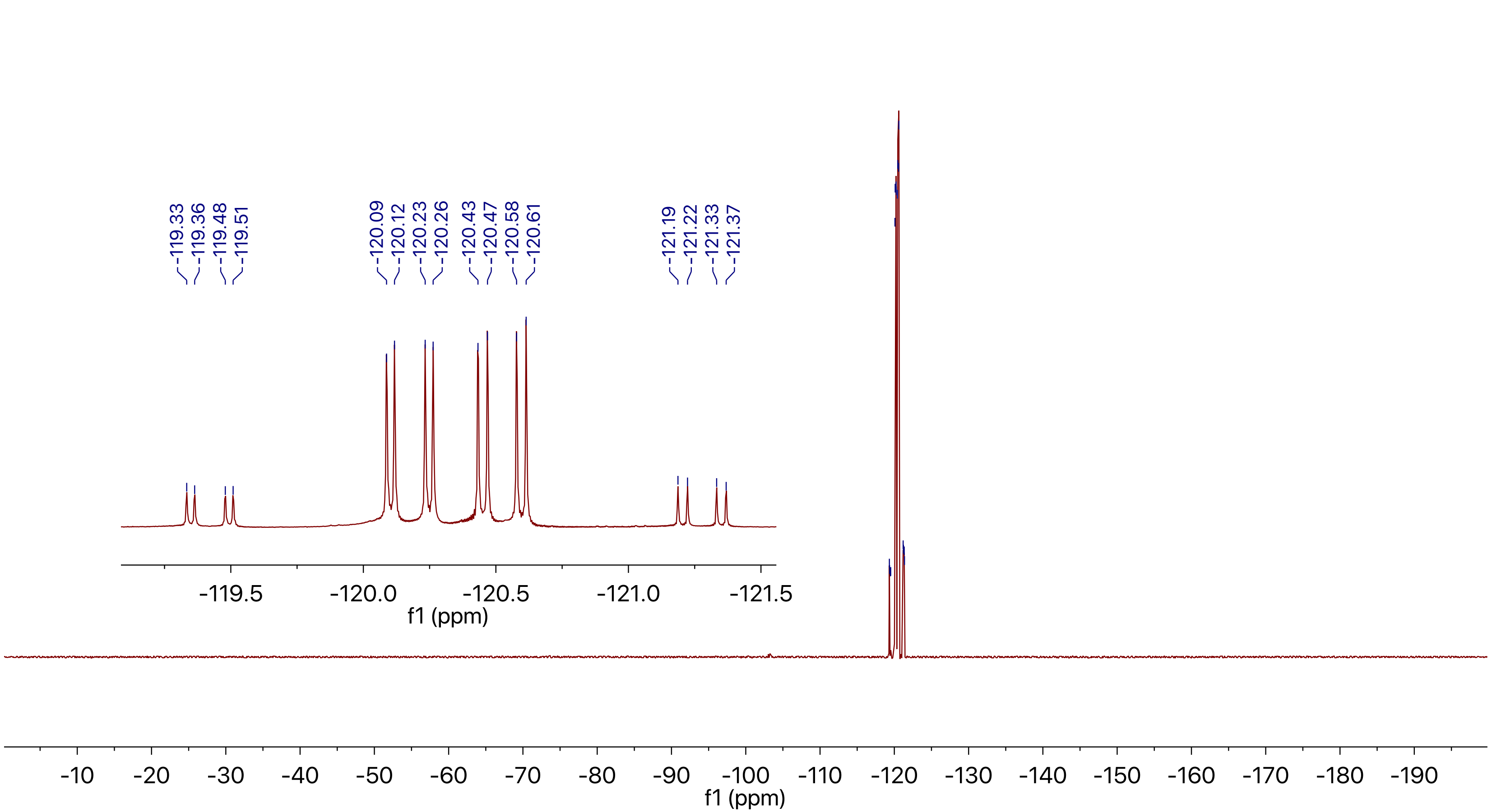

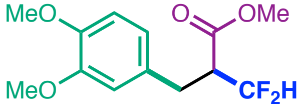

82

^{1}H NMR (400 MHz, $CDCl_3$)

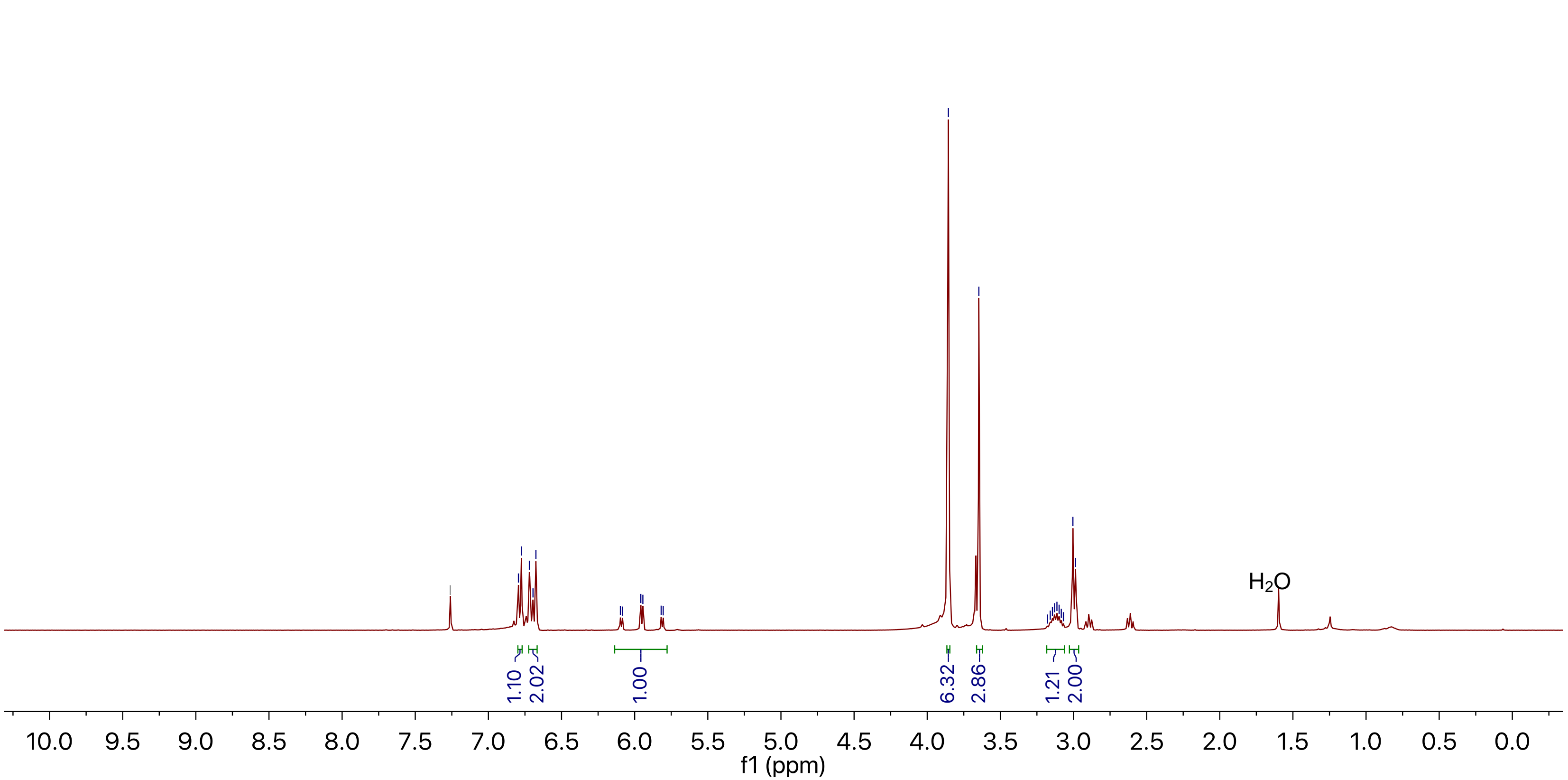

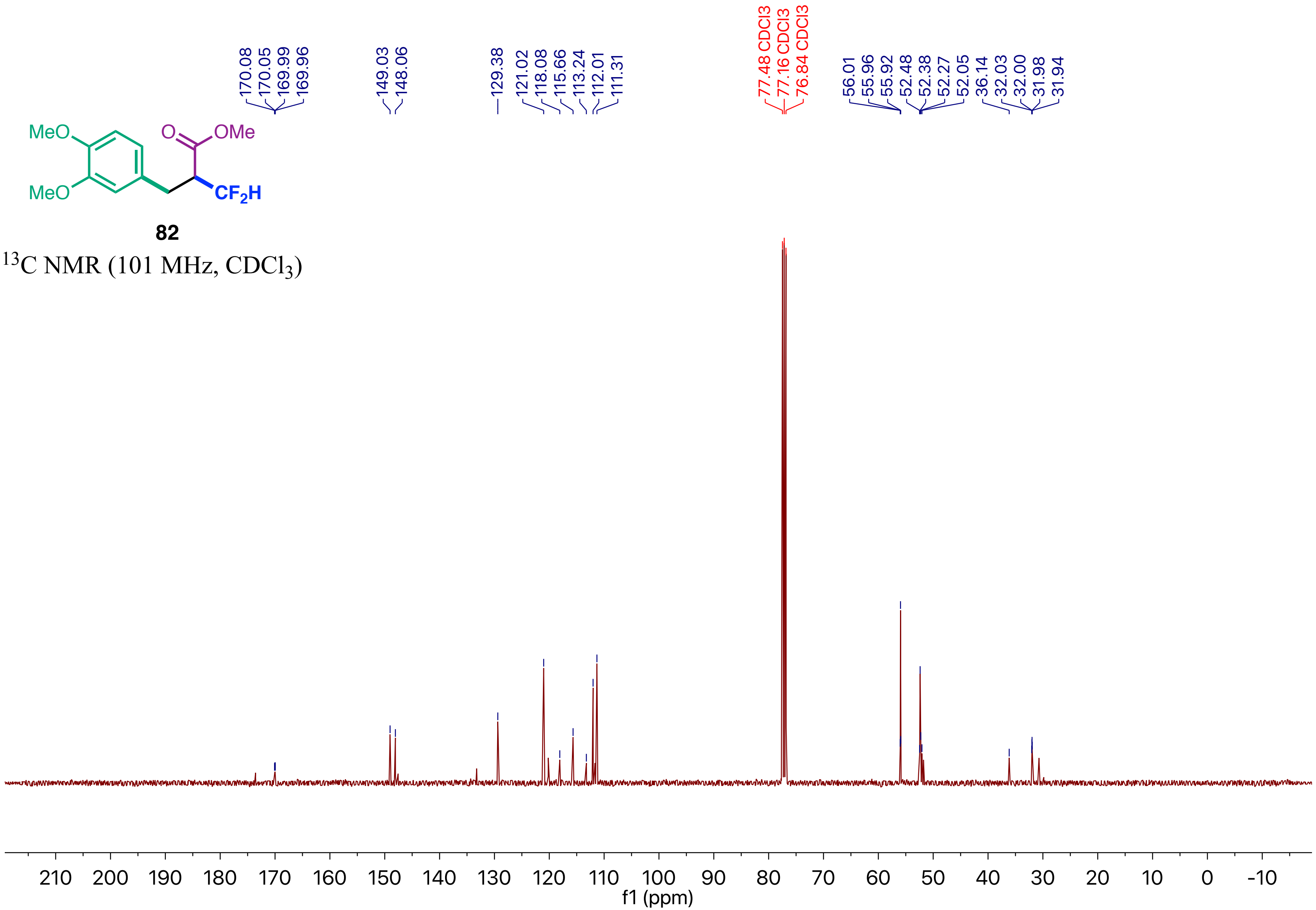

82

^{13}C NMR (101 MHz, $CDCl_3$)

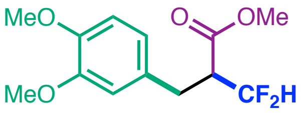

82

^{19}F NMR (376 MHz, $CDCl_3$)

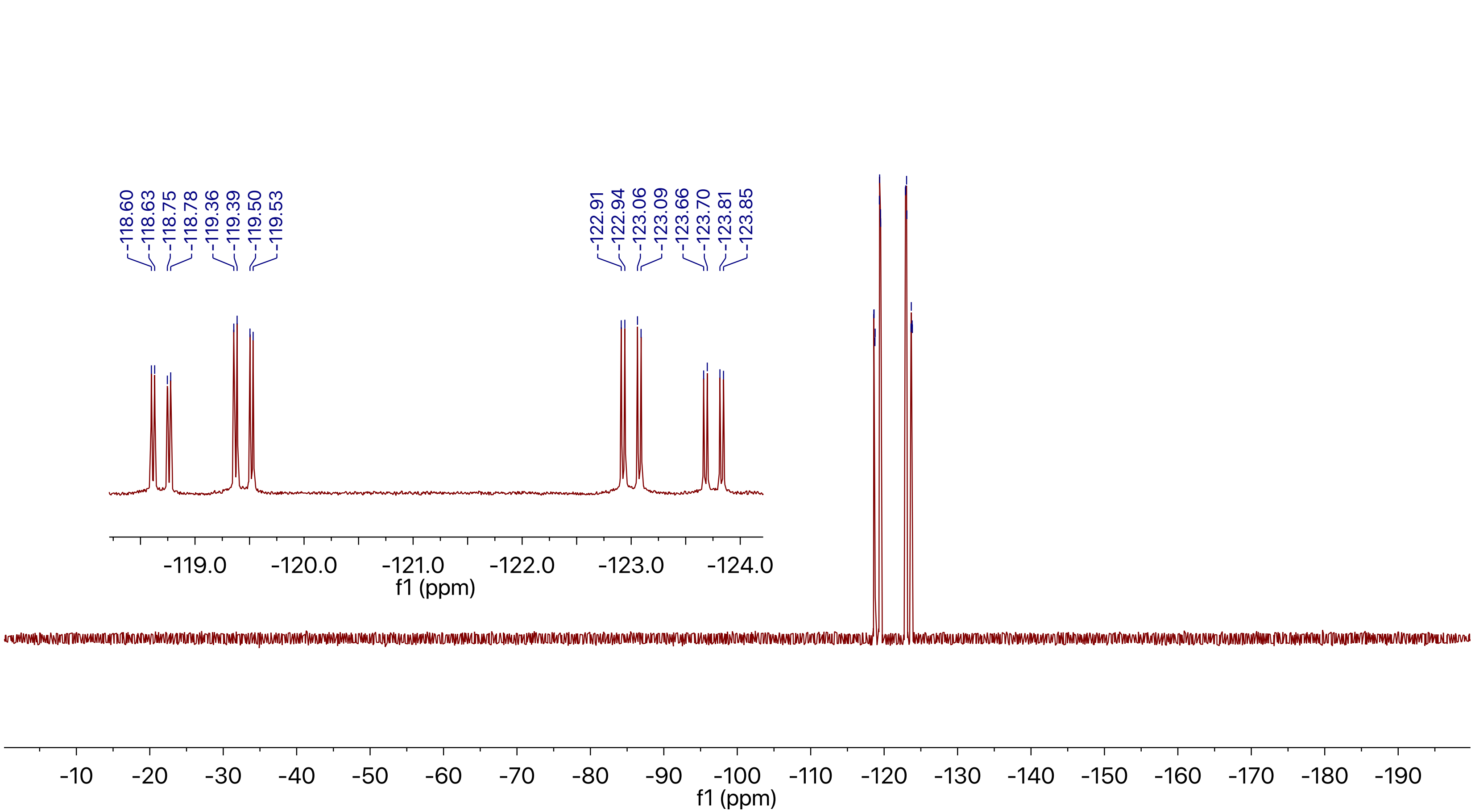

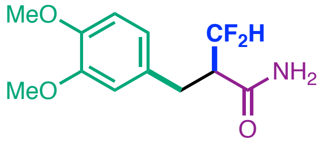

83

^{1}H NMR (400 MHz, $CDCl_3$)

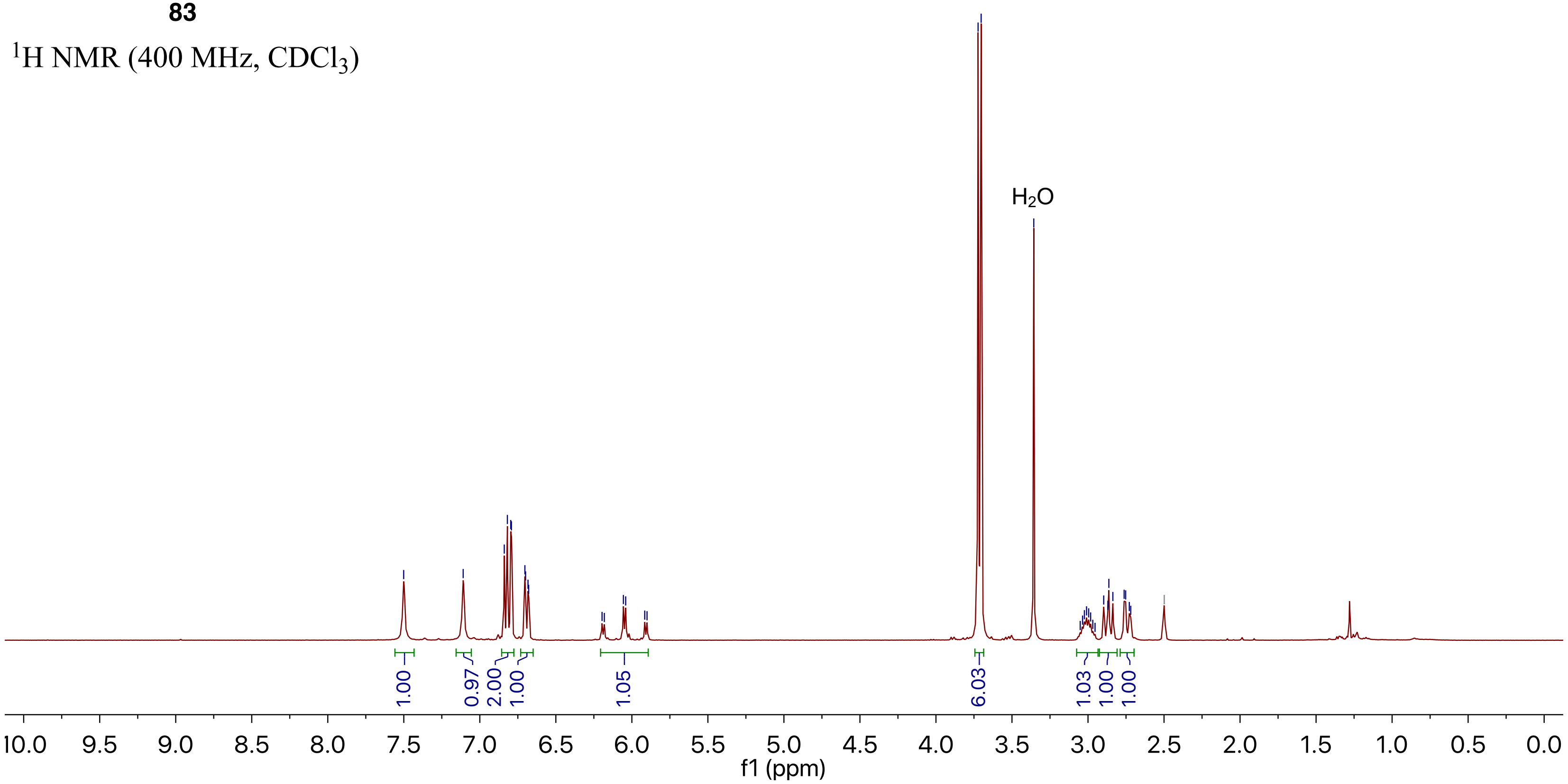

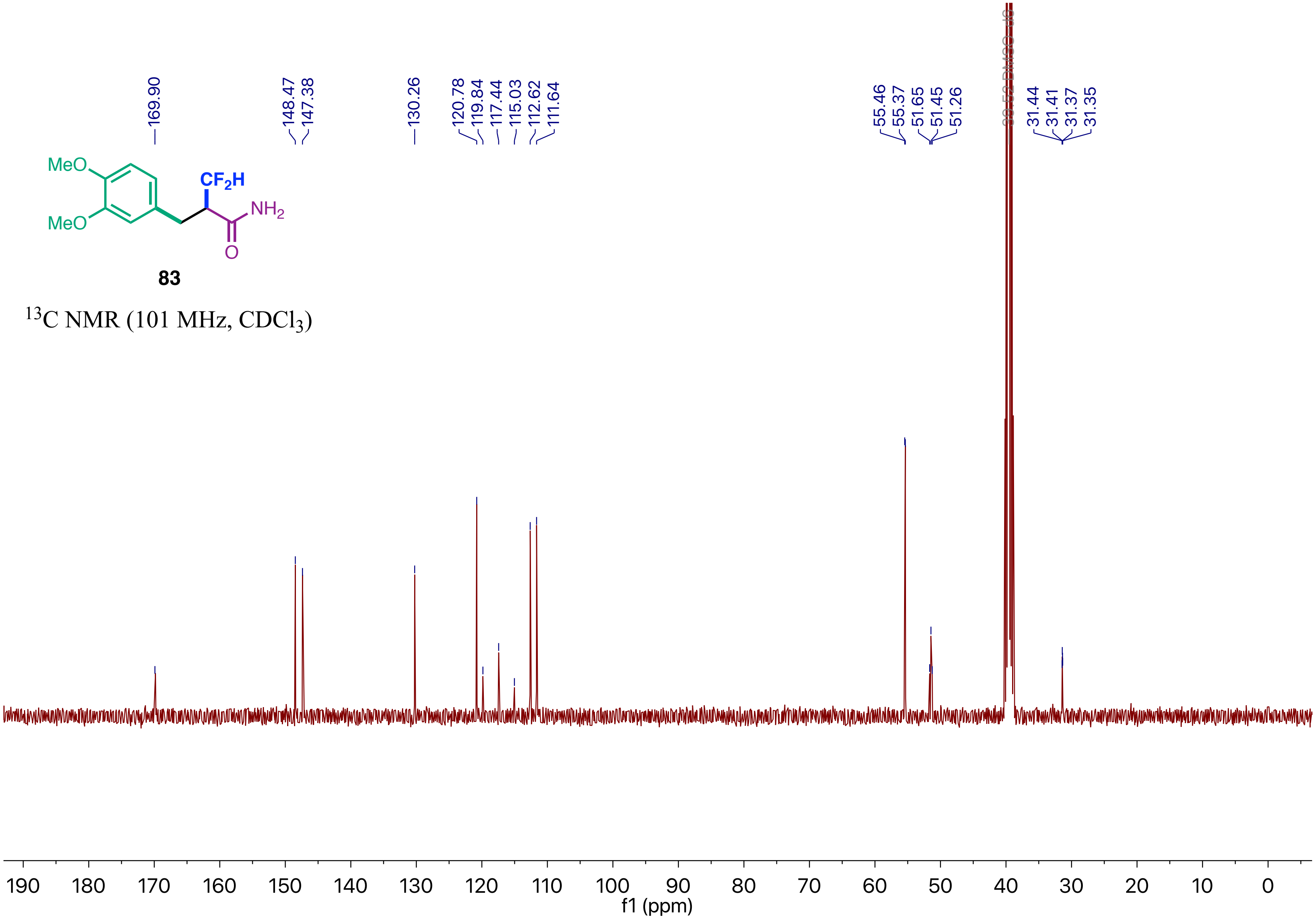

83

^{13}C NMR (101 MHz, $CDCl_3$)

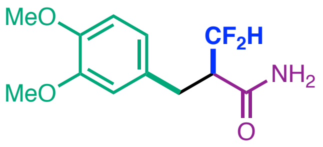

83

^{19}F NMR (376 MHz, $CDCl_3$)

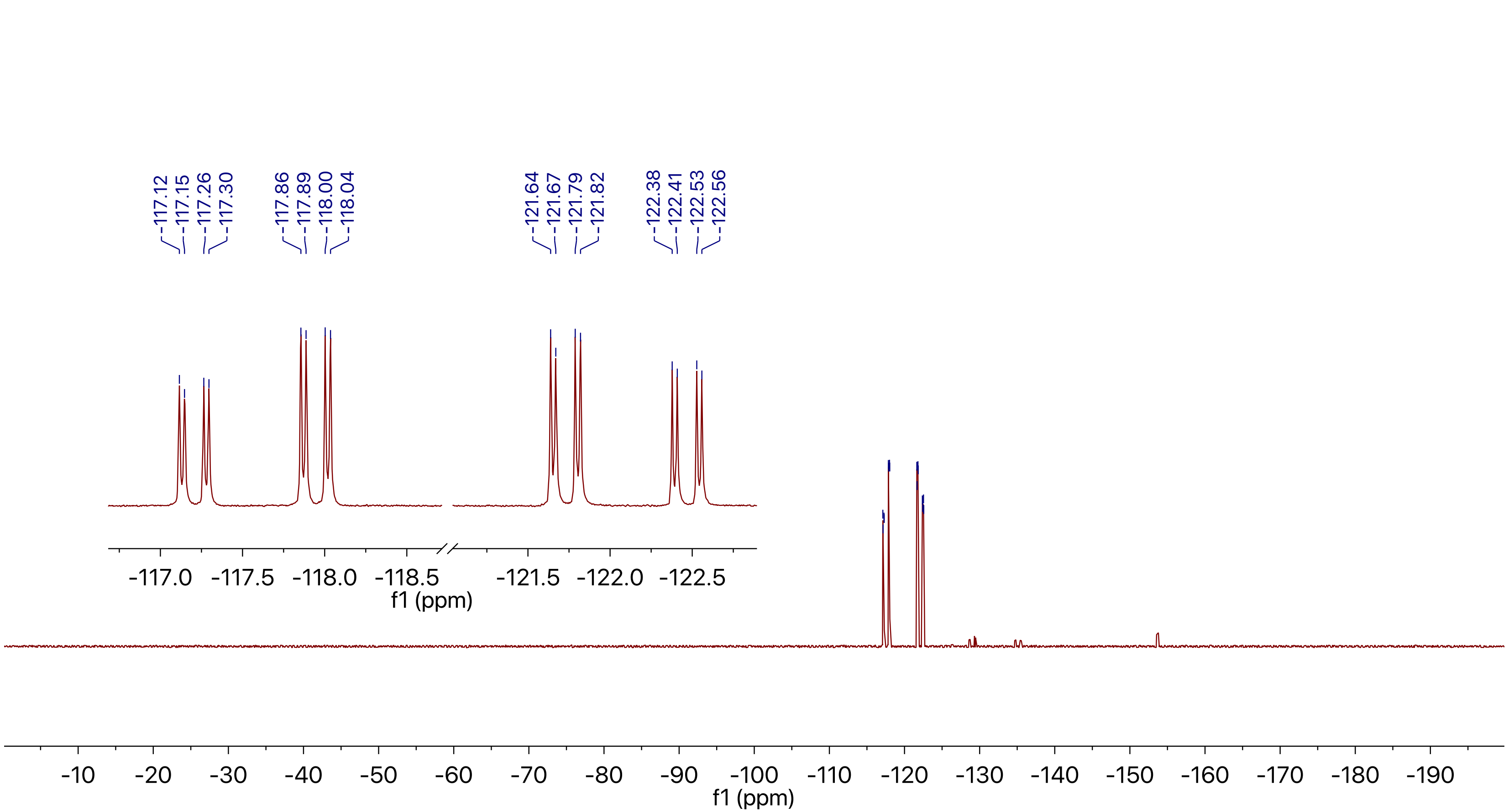

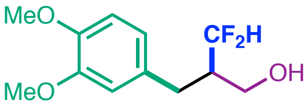

84

^{1}H NMR (400 MHz, $CDCl_3$)

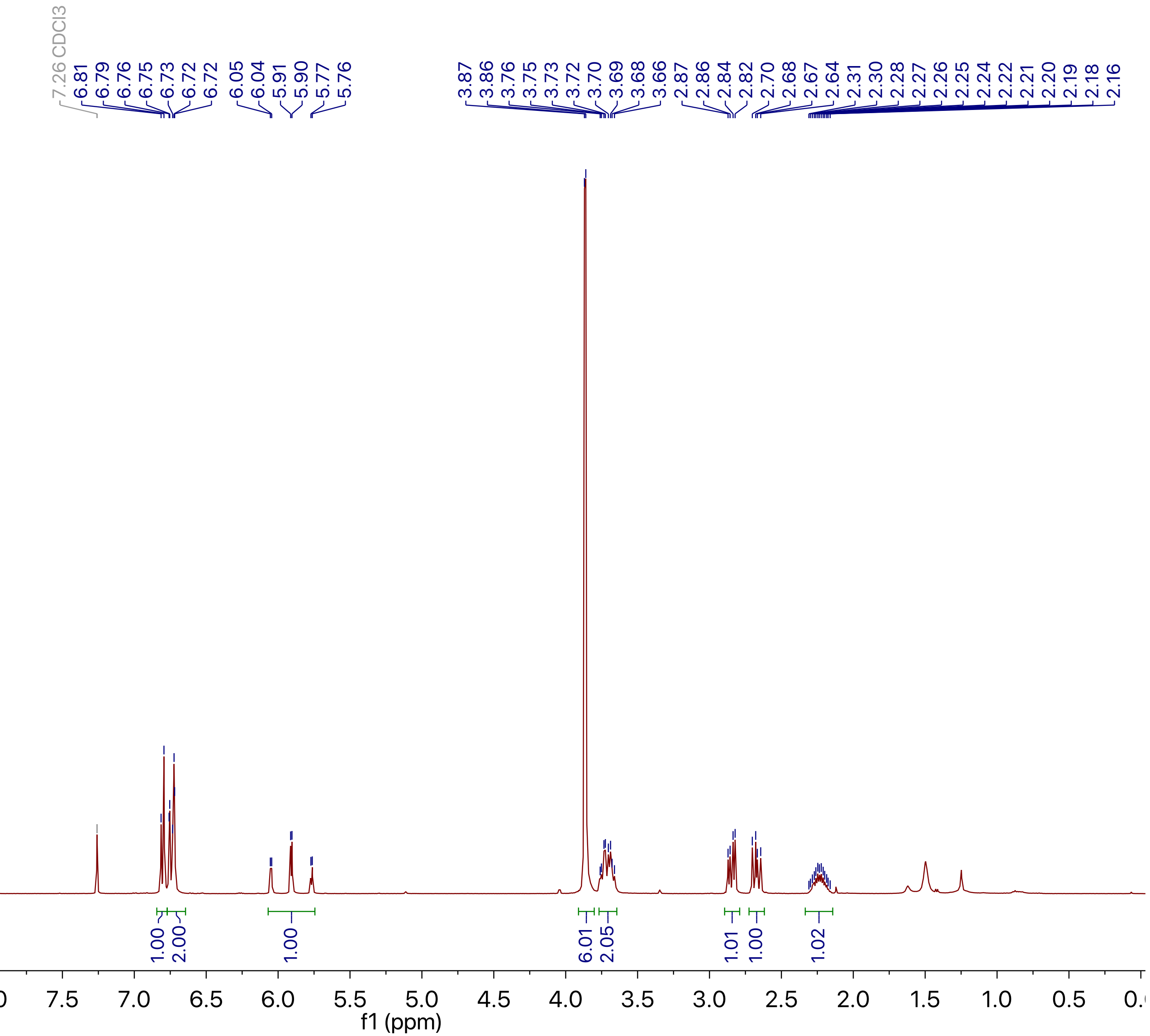

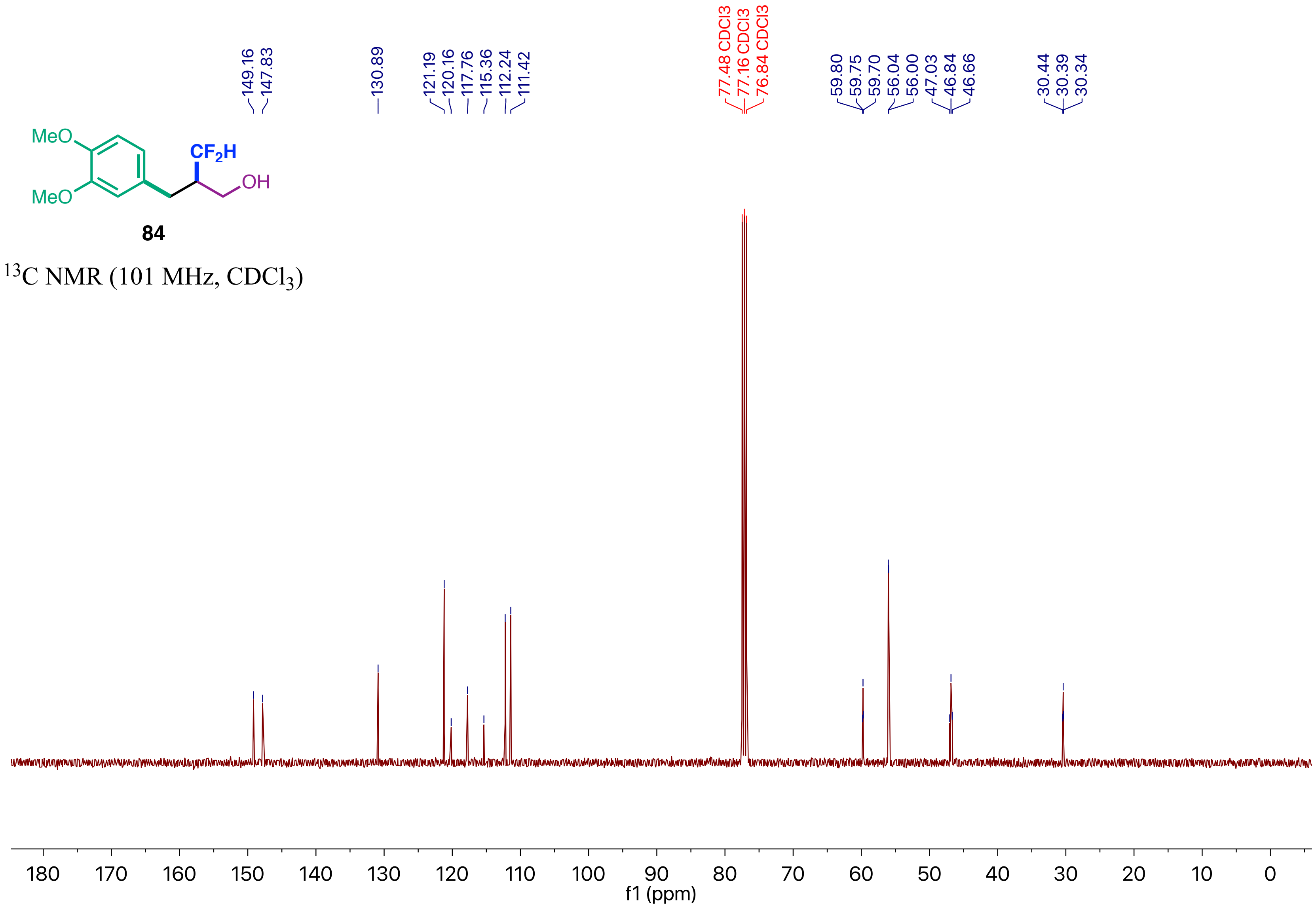
MeO
MeO
CF2H
OH
84
13C NMR (101 MHz, CDCl3)
149.16
147.83
130.89
121.19
120.16
117.76
115.36
112.24
111.42
77.48 CDCl3
77.16 CDCl3
76.84 CDCl3
59.80
59.75
59.70
56.04
56.00
47.03
46.84
46.66
30.44
30.39
30.34
180
170
160
150
140
130
120
110
100
90
80
70
60
50
40
30
20
10
0
f1 (ppm)

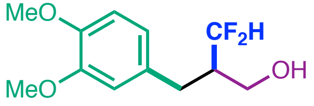

84

^{19}F NMR (376 MHz, $CDCl_3$)

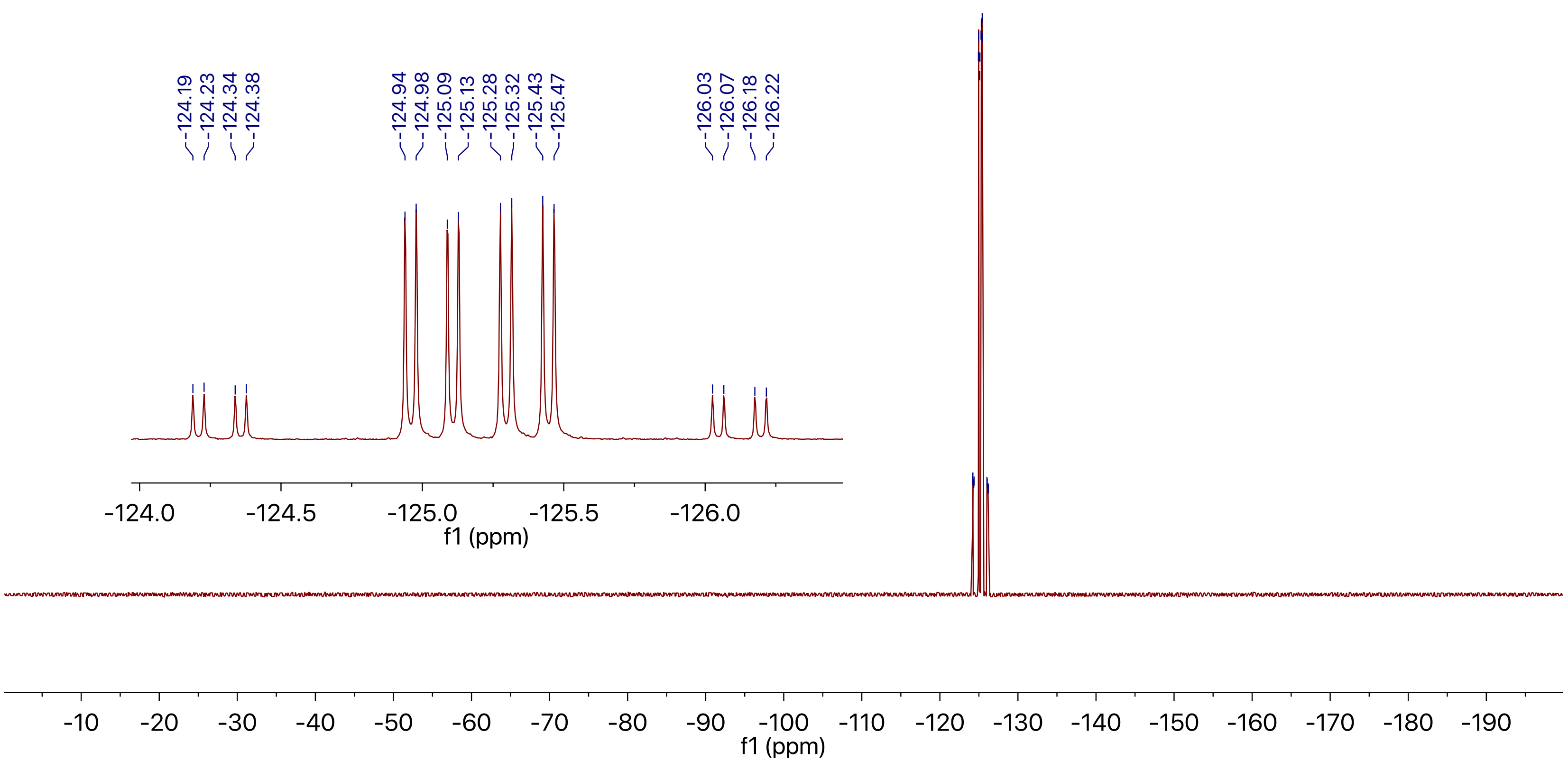

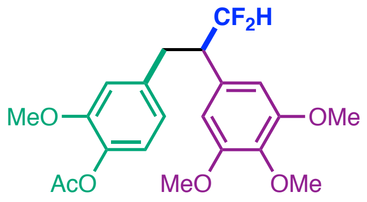

85

^{1}H NMR (400 MHz, $CDCl_3$)

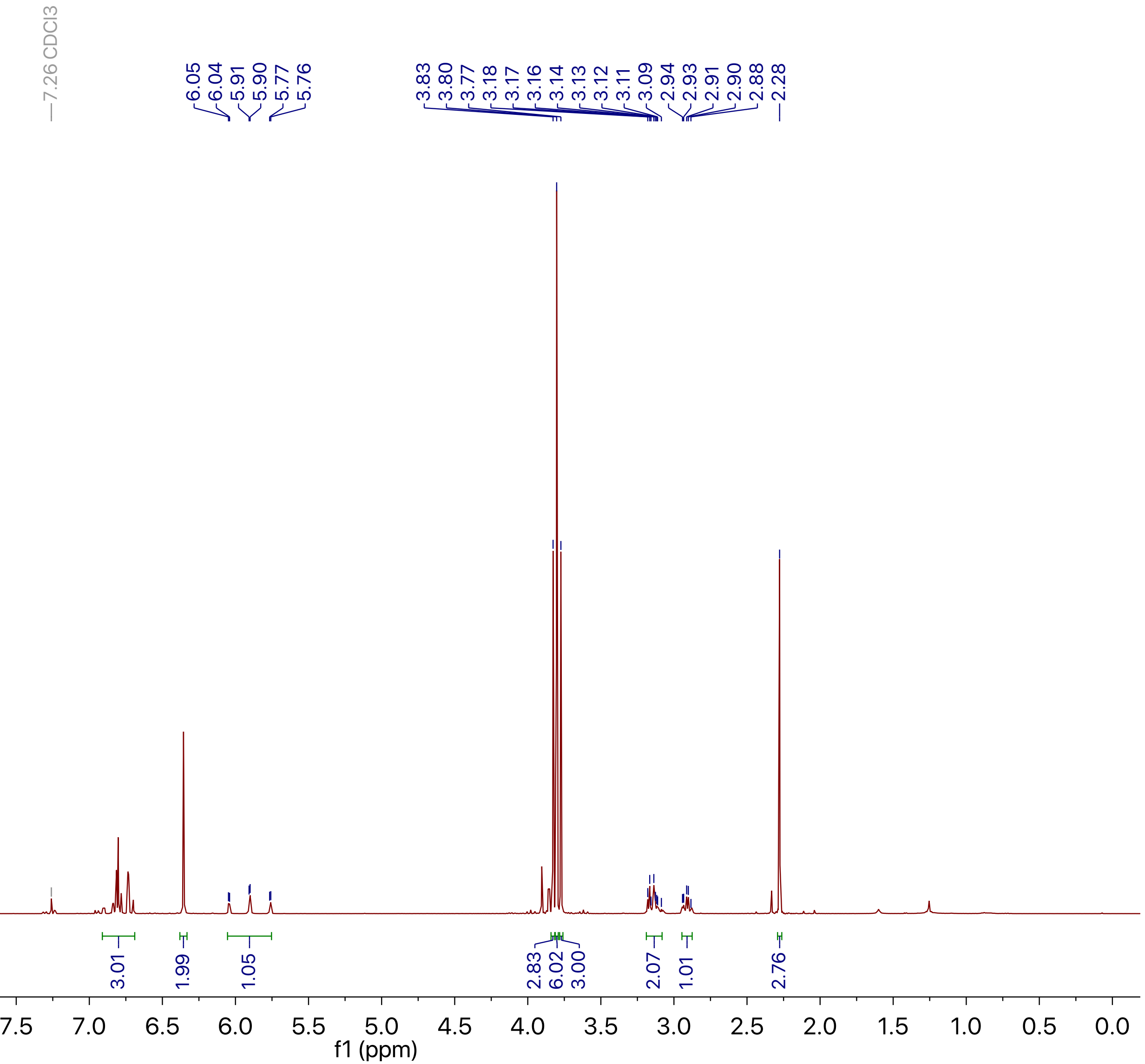

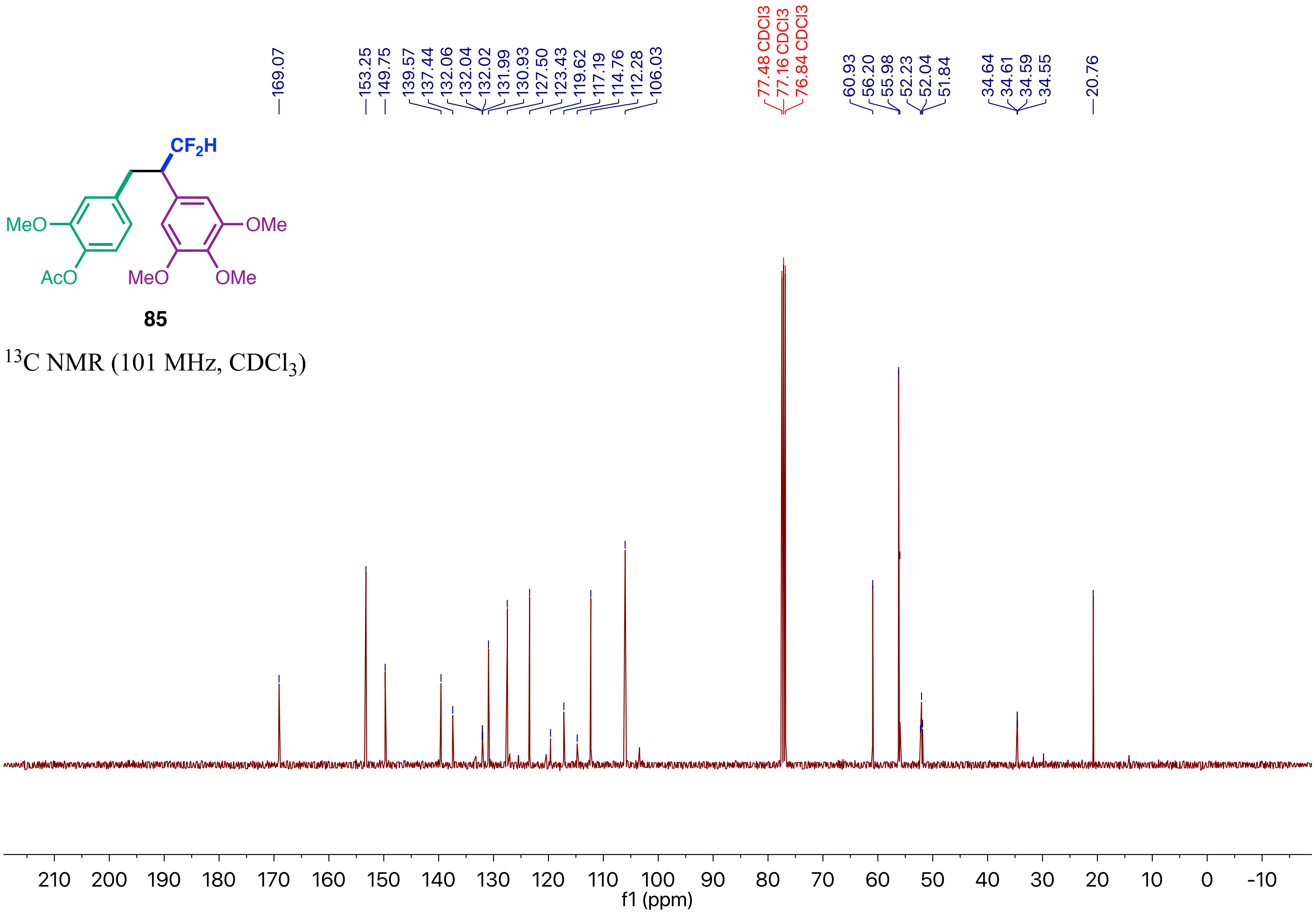

85

^{13}C NMR (101 MHz, $CDCl_3$)

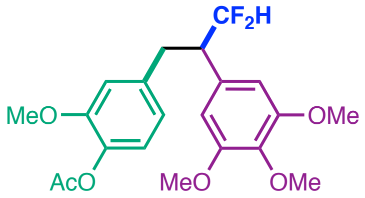

85

^{19}F NMR (376 MHz, $CDCl_3$)

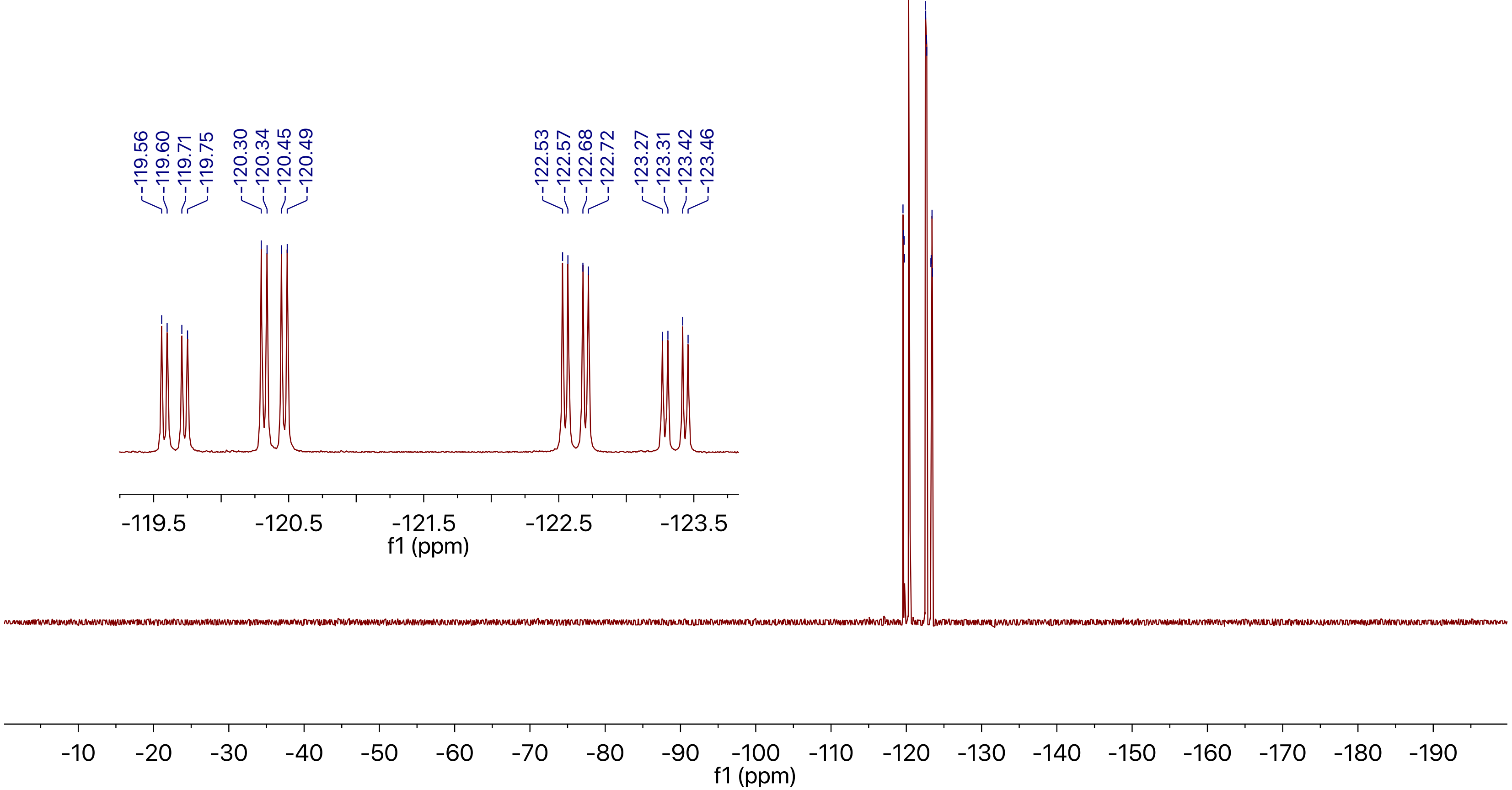

XIV. Supplementary References

1. J. M. Smith, T. Qin, R. R. Merchant, J. T. Edwards, L. R. Malins, Z. Liu, G. Che, Z. Shen, S. A. Shaw, M. D. Eastgate, P. S. Baran, *Angew. Chem. Int. Ed.* **2017**, *56*, 11906.
2. J. T. Edwards, R. R. Merchant, K. S. McClymont, K. W. Knouse, T. Qin, L. R. Malins, B. Vokits, S. A. Shaw, D.-H. Bao, F.-L. Wei, T. Zhou, M. D. Eastgate, P. S. Baran, *Nature* **2017**, *545*, 213.
3. T. Qin, J. Cornella, C. Li, L. R. Malins, J. T. Edwards, S. Kawamura, B. D. Maxwell, M. D. Eastgate, P. S. Baran, *Science* **2016**, *352*, 801.
4. C. Li, J. Wang, L. M. Barton, S. Yu, M. Tian, D. S. Peters, M. Kumar, A. W. Yu, K. A. Johnson, A. K. Chatterjee, M. Yan, P. S. Baran, *Science* **2017**, *356*, eaam7355.
5. F. Cong, Y. Wei, P. Tang, *Chem. Commun.,* **2018**, *54*, 4473.
6. M. D. Greenhalgh, S. P. Thomas, *J. Am. Chem. Soc*. **2012**, *134*, 11900.
7. Y. Liu, D. Yu, Y. Guo, J.-C. Xiao, Q.-Y. Chen, C. Liu, *Org. Lett*. **2020**, *22*, 2281.
8. H. Serizawa, K. Ishii, K. Ailawa, K. Mikami, *Org. Lett.* **2016**, *18*, 3686.
